# Some Dissimilarity Measures of Branching Processes and Optimal Decision Making in the Presence of Potential Pandemics

**DOI:** 10.3390/e22080874

**Published:** 2020-08-08

**Authors:** Niels B. Kammerer, Wolfgang Stummer

**Affiliations:** 1 Königinstrasse 75, 80539 Munich, Germany; nielskammerer@gmx.de; 2 Department of Mathematics, University of Erlangen–Nürnberg, Cauerstrasse 11, 91058 Erlangen, Germany

**Keywords:** Galton-Watson branching processes with immigration, Hellinger integrals, power divergences, Kullback-Leibler information distance/divergence, relative entropy, Renyi divergences, epidemiology, COVID-19 pandemic, Bayesian decision making, INARCH(1) model, GLM model, Bhattacharyya coefficient/distance

## Abstract

We compute exact values respectively bounds of dissimilarity/distinguishability measures–in the sense of the Kullback-Leibler information distance (relative entropy) and some transforms of more general power divergences and Renyi divergences–between two competing discrete-time *Galton-Watson branching processes with immigration* GWI for which the offspring as well as the immigration (importation) is arbitrarily Poisson-distributed; especially, we allow for arbitrary type of extinction-concerning criticality and thus for non-stationarity. We apply this to optimal decision making in the context of the spread of potentially pandemic infectious diseases (such as e.g., the current COVID-19 pandemic), e.g., covering different levels of dangerousness and different kinds of intervention/mitigation strategies. Asymptotic distinguishability behaviour and diffusion limits are investigated, too.

**Contents**


**1 Introduction**

**3**

**2 The Framework and Application Setups**

**5**
  2.1 Process Setup5  2.2 Connections to Time Series of Counts6  2.3 Applicability to Epidemiology8  2.4 Information Measures12  2.5 Decision Making under Uncertainty15  2.6 Asymptotical Distinguishability19
**3 Detailed Recursive Analyses of Hellinger Integrals**

**21**
  3.1 A First Basic Result21  3.2 Some Useful Facts for Deeper Analyses25  3.3 Detailed Analyses of the Exact Recursive Values, i.e., for the Cases $\left(\beta_{A},\beta_{H},\alpha_{A},\alpha_{H}\right)\in P_{\mathrm{NI}}\cup P_{\mathrm{SP},1}$27  3.4 Some Preparatory Basic Facts for the Remaining Cases $\left(\beta_{A},\beta_{H},\alpha_{A},\alpha_{H}\right)\in P_{\mathrm{SP}}\backslash P_{\mathrm{SP},1}$29  3.5 Lower Bounds for the Cases $\left(\beta_{A},\beta_{H},\alpha_{A},\alpha_{H},\lambda \right)\in \left(P_{\mathrm{SP}}\backslash P_{\mathrm{SP},1}\right)\times ]0,1[$31  3.6 Goals for Upper Bounds for the Cases $\left(\beta_{A},\beta_{H},\alpha_{A},\alpha_{H},\lambda \right)\in \left(P_{\mathrm{SP}}\backslash P_{\mathrm{SP},1}\right)\times ]0,1[$32  3.7 Upper Bounds for the Cases $\left(\beta_{A},\beta_{H},\alpha_{A},\alpha_{H},\lambda \right)\in P_{\mathrm{SP},2}\times ]0,1[$34  3.8 Upper Bounds for the Cases $\left(\beta_{A},\beta_{H},\alpha_{A},\alpha_{H},\lambda \right)\in P_{\mathrm{SP},3a}\times ]0,1[$35  3.9 Upper Bounds for the Cases $\left(\beta_{A},\beta_{H},\alpha_{A},\alpha_{H},\lambda \right)\in P_{\mathrm{SP},3b}\times ]0,1[$36  3.10 Upper Bounds for the Cases $\left(\beta_{A},\beta_{H},\alpha_{A},\alpha_{H},\lambda \right)\in P_{\mathrm{SP},3c}\times ]0,1[$37  3.11 Upper Bounds for the Cases $\left(\beta_{A},\beta_{H},\alpha_{A},\alpha_{H},\lambda \right)\in P_{\mathrm{SP},4a}\times ]0,1[$37  3.12 Upper Bounds for the Cases $\left(\beta_{A},\beta_{H},\alpha_{A},\alpha_{H},\lambda \right)\in P_{\mathrm{SP},4b}\times ]0,1[$37  3.13 Concluding Remarks on Alternative Upper Bounds for all Cases $\left(\beta_{A},\beta_{H},\alpha_{A},\alpha_{H},\lambda \right)\in$
$\left(P_{\mathrm{SP}}\backslash P_{\mathrm{SP},1}\right)\times ]0,1[$37  3.14 Intermezzo 1: Application to Asymptotical Distinguishability38  3.15 Intermezzo 2: Application to Decision Making under Uncertainty39   3.15.1 Bayesian Decision Making39   3.15.2. Neyman-Pearson Testing41  3.16 Goals for Lower Bounds for the Cases $\left(\beta_{A},\beta_{H},\alpha_{A},\alpha_{H},\lambda \right)\in \left(P_{\mathrm{SP}}\backslash P_{\mathrm{SP},1}\right)\times \left(R\backslash \left[0,1\right]\right)$41  3.17 Lower Bounds for the Cases $\left(\beta_{A},\beta_{H},\alpha_{A},\alpha_{H},\lambda \right)\in P_{\mathrm{SP},2}\times \left(R\backslash \left[0,1\right]\right)$44  3.18 Lower Bounds for the Cases $\left(\beta_{A},\beta_{H},\alpha_{A},\alpha_{H},\lambda \right)\in P_{\mathrm{SP},3a}\times \left(R\backslash \left[0,1\right]\right)$45  3.19 Lower Bounds for the Cases $\left(\beta_{A},\beta_{H},\alpha_{A},\alpha_{H},\lambda \right)\in P_{\mathrm{SP},3b}\times \left(R\backslash \left[0,1\right]\right)$46  3.20 Lower Bounds for the Cases $\left(\beta_{A},\beta_{H},\alpha_{A},\alpha_{H},\lambda \right)\in P_{\mathrm{SP},3c}\times \left(R\backslash \left[0,1\right]\right)$47  3.21 Lower Bounds for the Cases $\left(\beta_{A},\beta_{H},\alpha_{A},\alpha_{H},\lambda \right)\in P_{\mathrm{SP},4a}\times \left(R\backslash \left[0,1\right]\right)$47  3.22 Lower Bounds for the Cases $\left(\beta_{A},\beta_{H},\alpha_{A},\alpha_{H},\lambda \right)\in P_{\mathrm{SP},4b}\times \left(R\backslash \left[0,1\right]\right)$48  3.23 Concluding Remarks on Alternative Lower Bounds for all Cases $\left(\beta_{A},\beta_{H},\alpha_{A},\alpha_{H},\lambda \right)\in \left(P_{\mathrm{SP}}\backslash P_{\mathrm{SP},1}\right)\times \left(R\backslash \left[0,1\right]\right)$48  3.24 Upper Bounds for the Cases $\left(\beta_{A},\beta_{H},\alpha_{A},\alpha_{H},\lambda \right)\in \left(P_{\mathrm{SP}}\backslash P_{\mathrm{SP},1}\right)\times \left(R\backslash \left[0,1\right]\right)$48
**4 Power Divergences of Non-Kullback-Leibler-Information-Divergence Type**
49  4.1 A First Basic Result49  4.2 Detailed Analyses of the Exact Recursive Values of $I_{\lambda }\left(\cdot ∥\cdot \right)$, i.e., for the Cases $\left(\beta_{A},\beta_{H},\alpha_{A},\alpha_{H},\lambda \right)\in \left(P_{\mathrm{NI}}\cup P_{\mathrm{SP},1}\right)\times \left(R\backslash \left\{0,1\right\}\right)$51  4.3 Lower Bounds of $I_{\lambda }\left(\cdot ∥\cdot \right)$ for the Cases $\left(\beta_{A},\beta_{H},\alpha_{A},\alpha_{H},\lambda \right)\in \left(P_{\mathrm{SP}}\backslash P_{\mathrm{SP},1}\right)\times ]0,1[$52  4.4 Upper Bounds of $I_{\lambda }\left(\cdot ∥\cdot \right)$ for the Cases $\left(\beta_{A},\beta_{H},\alpha_{A},\alpha_{H},\lambda \right)\in \left(P_{\mathrm{SP}}\backslash P_{\mathrm{SP},1}\right)\times ]0,1[$53  4.5 Lower Bounds of $I_{\lambda }\left(\cdot ∥\cdot \right)$ for the Cases $\left(\beta_{\;A},\beta_{\;H},\alpha_{\;A},\alpha_{\;H},\lambda \right)\;\in \;\left(P_{\mathrm{SP}}\;\backslash P_{\mathrm{SP},1}\right)\;\times \;\left(R\backslash \;\left[0,\;1\right]\right)$53  4.6 Upper Bounds of $I_{\lambda }\left(\cdot ∥\cdot \right)$ for the Cases $\left(\beta_{A},\beta_{H},\alpha_{A},\alpha_{H},\lambda \right)\in \left(P_{\mathrm{SP}}\backslash P_{\mathrm{SP},1}\right)\times \left(R\backslash \left[0,1\right]\right)$54  4.7 Applications to Bayesian Decision Making55
**5 Kullback-Leibler Information Divergence (Relative Entropy)**

**55**
  5.1 Exact Values Respectively Upper Bounds of I(·||·)55  5.2 Lower Bounds of I(·||·) for the Cases $\left(\beta_{A},\beta_{H},\alpha_{A},\alpha_{H}\right)\in \left(P_{\mathrm{SP}}\backslash P_{\mathrm{SP},1}\right)$56  5.3 Applications to Bayesian Decision Making58
**6 Explicit Closed-Form Bounds of Hellinger Integrals**

**59**
  6.1 Principal Approach59  6.2 Explicit Closed-Form Bounds for the Cases $\left(\beta_{A},\beta_{H},\alpha_{A},\alpha_{H},\lambda \right)\in \left(P_{\mathrm{NI}}\cup P_{\mathrm{SP},1}\right)\times \left(R\backslash \left\{0,1\right\}\right)$63  6.3 Explicit Closed-Form Bounds for the Cases $\left(\beta_{A},\beta_{H},\alpha_{A},\alpha_{H},\lambda \right)\in \left(P_{\mathrm{SP}}\backslash P_{\mathrm{SP},1}\right)\times ]0,1[$64  6.4 Explicit Closed-Form Bounds for the Cases $\left(\beta_{A},\beta_{H},\alpha_{A},\alpha_{H},\lambda \right)\in \left(P_{\mathrm{SP}}\backslash P_{\mathrm{SP},1}\right)\times \left(R\backslash \left[0,1\right]\right)$67  6.5 Totally Explicit Closed-Form Bounds69  6.6 Closed-Form Bounds for Power Divergences of Non-Kullback-Leibler-Information-Divergence Type70  6.7 Applications to Decision Making71
**7 Hellinger Integrals and Power Divergences of Galton-Watson Type Diffusion Approximations**

**71**
  7.1 Branching-Type Diffusion Approximations71  7.2 Bounds of Hellinger Integrals for Diffusion Approximations74  7.3 Bounds of Power Divergences for Diffusion Approximations79  7.4 Applications to Decision Making80
**A Proofs and Auxiliary Lemmas**

**81**
  A.1. Proofs and Auxiliary Lemmas for Section 381  A.2 Proofs and Auxiliary Lemmas for Section 588  A.3 Proofs and Auxiliary Lemmas for Section 694  A.4 Proofs and Auxiliary Lemmas for Section 7101
**References**

**115**


## 1. Introduction

(This paper is a thoroughly revised, extended and retitled version of the preprint arXiv:1005.3758v1 of both authors) Over the past twenty years, *density-based divergences* D(P,Q) –also known as (dis)similarity measures, directed distances, disparities, distinguishability measures, proximity measures–between probability distributions *P* and *Q*, have turned out to be of substantial importance for decisive statistical tasks such as parameter estimation, testing for goodness-of-fit, Bayesian decision procedures, change-point detection, clustering, as well as for other research fields such as information theory, artificial intelligence, machine learning, signal processing (including image and speech processing), pattern recognition, econometrics, and statistical physics. For some comprehensive overviews on the divergence approach to statistics and probability, the reader is referred to the insightful books of e.g., Liese & Vajda [1], Read & Cressie [2], Vajda [3], Csiszár & Shields [4], Stummer [5], Pardo [6], Liese & Miescke [7], Basu et al. [8], Voinov et al. [9], the survey articles of e.g., Liese & Vajda [10], Vajda & van der Meulen [11], the structure-building papers of Stummer & Vajda [12], Kißlinger & Stummer [13] and Broniatowski & Stummer [14], and the references therein. Divergence-based bounds of minimal mean decision risks (e.g., Bayes risks in finance) can be found e.g., in Stummer & Vajda [15] and Stummer & Lao [16].

Amongst the above-mentioned dissimilarity measures, an important omnipresent subclass are the so-called f–divergences of Csiszar [17], Ali & Silvey [18] and Morimoto [19]; important special cases thereof are the total variation distance and the very frequently used *λ–order power divergences* $I_{\lambda }\left(P,Q\right)$ (also known as alpha-entropies, Cressie-Read measures, Tsallis cross-entropies) with λ∈R. The latter cover e.g., the very prominent Kullback-Leibler information divergence $I_{1}\left(P,Q\right)$ (also called relative entropy), the (squared) Hellinger distance $I_{1/2}\left(P,Q\right)$, as well as the Pearson chi-square divergence $I_{2}\left(P,Q\right)$. It is well known that the power divergences can be build with the help of the *λ–order Hellinger integrals* $H_{\lambda }\left(P,Q\right)$ (where e.g., the case λ=1/2 corresponds to the well-known Bhattacharyya coefficient), which are information measures of interest by their own and which are also the crucial ingredients of *λ–order Renyi divergences* $R_{\lambda }\left(P,Q\right)$ (see e.g., Liese & Vajda [1], van Erven & Harremoes [20]); the case $R_{1/2}\left(P,Q\right)$ corresponds to the well-known Bhattacharyya distance.

The above-mentioned information/dissimilarity measures have been also investigated in non-static, time-dynamic frameworks such as for various different contexts of *stochastic processes* like *processes with independent increments* (see e.g., Newman [21], Liese [22], Memin & Shiryaev [23], Jacod & Shiryaev [24], Liese & Vajda [1], Linkov & Shevlyakov [25]), *Poisson point processes* (see e.g., Liese [26], Jacod & Shiryaev [24], Liese & Vajda [1]), *diffusion prcoesses and solutions of stochastic differential equations with continuous paths* (see e.g., Kabanov et al. [27], Liese [28], Jacod & Shiryaev [24], Liese & Vajda [1], Vajda [29], Stummer [30,31,32], Stummer & Vajda [15]), and *generalized binomial processes* (see e.g., Stummer & Lao [16]); further related literature can be found e.g., in references of the aforementioned papers and books.

Another important class of time-dynamic models is given by *discrete-time integer-valued branching processes*, in particular *(Bienaymé-)Galton-Watson processes without immigration* GW respectively *with immigration (resp. importation, invasion)* GWI, which have numerous applications in biotechnology, population genetics, internet traffic research, clinical trials, asset price modelling, derivative pricing, and many others. As far as important terminology is concerned, we abbreviatingly subsume both models as GW(I) and, simply as GWI in case that GW appears as a parameter-special-case of GWI; recall that a GW(I) is called *subcritical* respectively *critical* respectively *supercritical* if its offspring mean is less than 1 respectively equal to 1 respectively larger than 1.

For applications of GW(I) in *epidemiology*, see e.g., the works of Bartoszynski [33], Ludwig [34], Becker [35,36], Metz [37], Heyde [38], von Bahr & Martin-Löf [39], Ball [40], Jacob [41], Barbour & Reinert [42], Section 1.2 of Britton & Pardoux [43]); for more details see Section 2.3 below.

For connections of GW(I) to *time series of counts* including GLM models, see e.g., Dion, Gauthier & Latour [44], Grunwald et al. [45], Kedem & Fokianos [46], Held, Höhle & Hofmann [47], and Weiß [48]; a more comprehensive discussion can be found in Section 2.2 below.

As far as the combined study of information measures and GW processes is concerned, let us first mention that (transforms of) power divergences have been used for supercritical Galton-Watson processes without immigration for instance as follows: Feigin & Passy [49] study the problem to find an offspring distribution which is closest (in terms of relative entropy type distance) to the original offspring distribution and under which ultimate extinction is certain. Furthermore, Mordecki [50] gives an equivalent characterization for the stable convergence of the corresponding log-likelihood process to a mixed Gaussian limit, in terms of conditions on Hellinger integrals of the involved offspring laws. Moreover, Sriram & Vidyashankar [51] study the properties of offspring-distribution-parameters which minimize the squared Hellinger distance between the model offspring distribution and the corresponding non-parametric maximum likelihood estimator of Guttorp [52]. For the setup of GWI with Poisson offspring and nonstochastic immigration of constant value 1, Linkov & Lunyova [53] investigate the asymptotics of Hellinger integrals in order to deduce large deviation assertions in hypotheses testing problems.

In contrast to the above-mentioned contexts, this paper pursues the following main goals:

(MG1) for any time horizon and any criticality scenario (allowing for non-stationarities), to compute lower and upper bounds–and sometimes even exact values–of the Hellinger integrals $H_{\lambda }\left(P_{A}∥P_{H}\right)$, power divergences $I_{\lambda }\left(P_{A}∥P_{H}\right)$ and Renyi divergences $R_{\lambda }\left(P_{A}∥P_{H}\right)$ of two alternative Galton-Watson branching processes $P_{A}$ and $P_{H}$ (on path/scenario space), where (i) $P_{A}$ has Poisson($\beta_{A}$) distributed offspring as well as Poisson($\alpha_{A}$) distributed immigration, and (ii) $P_{H}$ has Poisson($\beta_{H}$) distributed offspring as well as Poisson($\alpha_{H}$) distributed immigration; the non-immigration cases are covered as $\alpha_{A}=\alpha_{H}=0$; as a side effect, we also aim for corresponding asymptotic distinguishability results;
(MG2) to compute the corresponding limit quantities for the context in which (a proper rescaling of) the two alternative Galton-Watson processes with immigration converge to *Feller*-type branching diffusion processes, as the time-lags between the generation-size observations tend to zero;
(MG3) as an exemplary field of application, to indicate how to use the results of (MG1) for Bayesian decision making in the epidemiological context of an infectious-disease pandemic (e.g., the current COVID-19), where e.g., potential state-budgetary losses can be controlled by alternative public policies (such as e.g., different degrees of lockdown) for mitigations of the time-evolution of the number of infectious persons (being quantified by a GW(I)). Corresponding Neyman-Pearson testing will be treated, too.

Because of the involved Poisson distributions, these goals can be tackled with a high degree of tractability, which is worked out in detail with the following structure (see also the full table of contents after this paragraph): in Section 2, we first introduce (i) the basic ingredients of Galton-Watson processes together with their interpretations in the above-mentioned pandemic setup where it is essential to study *all* types of criticality (being connected with levels of reproduction numbers), (ii) the employed fundamental information measures such as Hellinger integrals, power divergences and Renyi divergences, (iii) the underlying decision-making framework, as well as (iv) connections to time series of counts and asymptotical distinguishability. Thereafter, we start our detailed technical analyses by giving *recursive* exact values respectively *recursive* bounds–as well as their applications–of Hellinger integrals $H_{\lambda }\left(P_{A}∥P_{H}\right)$ (see Section 3), power divergences $I_{\lambda }\left(P_{A}∥P_{H}\right)$ and Renyi divergences $R_{\lambda }\left(P_{A}∥P_{H}\right)$ (see Section 4 and Section 5). *Explicit closed-form* bounds of Hellinger integrals $H_{\lambda }\left(P_{A}∥P_{H}\right)$ will be worked out in Section 6, whereas Section 7 deals with Hellinger integrals and power divergences of the above-mentioned Galton-Watson type diffusion approximations.

## 2. The Framework and Application Setups

### 2.1. Process Setup

We investigate dissimilarity measures and apply them to decisions, in the following context. Let the integer-valued random variable $X_{n}$ ($n\in N_{0}$) denote the size of the *n*th generation of a population (of persons, organisms, spreading news, other kind of objects, etc.) with specified characteristics, and suppose that for the modelling of the time-evolution $n\mapsto X_{n}$ we have the choice between the following two (e.g., alternative, competing) models (H) and (A):

(H) a discrete-time homogeneous *Galton-Watson process with immigration GWI*, given by the recursive description

(1) $$X_{0}\in N;\;N_{0}∋\;X_{n}=\sum_{k=1}^{X_{n-1}}Y_{n-1,k}\;+\;{\tilde{Y}}_{n},\;n\in N,$$

where $Y_{n-1,k}$ is the number of offspring of the *k*th object (e.g., organism, person) within the (n−1)th generation, and ${\tilde{Y}}_{n}$ denotes the number of immigrating objects in the *n*th generation. Notice that we employ an arbitrary *deterministic* (i.e., degenerate random) initial generation size $X_{0}$. We always assume that under the corresponding dynamics-governing law $P_{H}$

(GWI1) the collection $Y:=\left\{Y_{n-1,k},\;n\in N,k\in N\right\}$ consists of independent and identically distributed (i.i.d.) random variables which are Poisson distributed with parameter $\beta_{H}>0$,
(GWI2) the collection $\tilde{Y}:=\left\{{\tilde{Y}}_{n},\;n\in N\right\}$ consists of i.i.d. random variables which are Poisson distributed with parameter $\alpha_{H}\geq 0$ (where $\alpha_{H}=0$ stands for the degenerate case of having no immigration),
(GWI3) *Y* and $\tilde{Y}$ are independent.

(A) a discrete-time homogeneous *Galton-Watson process with immigration GWI* given by the same recursive description (1), but with different dynamics-governing law $P_{A}$ under which (GWI1) holds with parameter $\beta_{A}>0$ (instead of $\beta_{H}>0$), (GWI2) holds with $\alpha_{A}\geq 0$ (instead of $\alpha_{H}\geq 0$), and (GWI3) holds. As a side remark, in some contexts the two models (H) and (A) may function as a “sandwich” of a more complicated not fully known model.

Basic and advanced facts on general GWI (introduced by Heathcote [54]) can be found e.g., in the monographs of Athreya & Ney [55], Jagers [56], Asmussen & Hering [57], Haccou [58]; see also e.g., Heyde & Seneta [59], Basawa & Rao [60], Basawa & Scott [61], Sankaranarayanan [62], Wei & Winnicki [63], Winnicki [64], Guttorp [52] as well as Yanev [65] (and also the references therein all those) for adjacent fundamental statistical issues including the involved technical and conceptual challenges.

For the sake of brevity, wherever we introduce or discuss corresponding quantities *simultaneously* for both models H and A, we will use the subscript • as a synonym for either the symbol H or A. For illustration, recall the well-known fact that the corresponding conditional probabilities $P_{•}\left(X_{n}=\cdot \;|X_{n-1}=k\right)$ are again Poisson-distributed, with parameter $\beta_{•}\cdot k+\alpha_{•}$.

In oder to achieve a transparently representable structure of our results, we subsume the involved parameters as follows:

(PS1) $P_{\mathrm{SP}}$ is the set of all constellations $\left(\beta_{A},\beta_{H},\alpha_{A},\alpha_{H}\right)$ of real-valued parameters $\beta_{A}>0$, $\beta_{H}>0$, $\alpha_{A}>0$, $\alpha_{H}>0$, such that $\beta_{A}\neq \beta_{H}$ or $\alpha_{A}\neq \alpha_{H}$ (or both); in other words, both models are non-identical and have non-vanishing immigration;
(PS2) $P_{\mathrm{NI}}$ is the set of all $\left(\beta_{A},\beta_{H},\alpha_{A},\alpha_{H}\right)$ of real-valued parameters $\beta_{A}>0$, $\beta_{H}>0$, $\alpha_{A}=\alpha_{H}=0$, such that $\beta_{A}\neq \beta_{H}$; this corresponds to the important special case that both models have no immigration and are non-identical;
(PS3) the resulting disjoint union will be denoted by $P=P_{\mathrm{SP}}\cup P_{\mathrm{NI}}$.

Notice that for (unbridgeable) technical reasons, we *do not allow for* “crossovers” between “immigration and no-immigration” (i.e., $\alpha_{A}=0$ and $\alpha_{H}\neq 0$, respectively, $\alpha_{A}\neq 0$ and $\alpha_{H}=0$). For practice, this is not a strong restriction, since one may take e.g., $\alpha_{A}=10^{-12}$ and $\alpha_{H}=1$.

For the non-immigration case $\alpha_{•}=0$ one has the following *extinction properties* (see e.g., Harris [66], Athreya & Ney [55]). As usual, let us define the extinction time $\tau :=\min\{i\in N:X_{\ell }=0$ for all integers ℓ≥i} if this minimum exists, and τ:=∞ else. Correspondingly, let B:={τ<∞} be the extinction set. If the *offspring mean* $\beta_{•}$ satisfies $\beta_{•}<1$—which is called the *subcritical* case– or $\beta_{•}=1$—which is known as the *critical* case–then extinction is certain, i.e., there holds $P(B\;|\;X_{0}=1)=1$. However, if the offspring mean satisfies $\beta_{•}>1$—which is called the *supercritical* case–then there is a probability greater than zero, that the population never dies out, i.e., $P\left(B\;|\;X_{0}=1\right)\in ]0,1[$. In the latter case, $X_{n}$ explodes (a.s.) to infinity as n→∞.

In contrast, for the (nondegenerate, nonvanishing) immigration case $\alpha_{•}\neq 0$ there is *no extinction*, viz. $P(B\;|\;X_{0}=1)=0$, although there may be zero population $X_{\ell_{0}}=0$ for some intermediate time $\ell_{0}\in N$; but due to the immigration, with probability one there is always a later time $\ell_{1}>\ell_{0}$, such that $X_{\ell_{1}}>0$. Nevertheless, also for the setup $\alpha_{•}\neq 0$ it is important to know whether $\beta_{•}⪌1$—which is still called (super-, sub-)criticality–since e.g., in the case $\beta_{•}<1$ the population size $X_{n}$ converges (as n→∞) to a stationary distribution on N whereas for $\beta_{•}>1$ the behaviour is non-stationary (non-ergodic), see e.g., Athreya & Ney [55].

At this point, let us emphasize that in our investigations (both for $\alpha_{•}=0$ and for $\alpha_{•}\neq 0$) we *do allow for* “crossovers” between “different criticalities”, i.e., we deal with all cases $\beta_{A}⪌1$ versus all cases $\beta_{H}⪌1$; as will be explained in the following, this unifying flexibility is especially important for corresponding epidemiological-model comparisons (e.g., for the sake of decision making).

One of our main goals is to quantitatively compare (the time-evolution of) two competing GWI models H and A with respective parameter sets $\left(\beta_{H},\alpha_{H}\right)$ and $\left(\beta_{A},\alpha_{A}\right)$, in terms of the information measures $H_{\lambda }\left(P_{A}∥P_{H}\right)$ (Hellinger intergrals), $I_{\lambda }\left(P_{A}∥P_{H}\right)$ (power divergences), $R_{\lambda }\left(P_{A}∥P_{H}\right)$ (Renyi divergences). The latter two express a distance (degree of dissimilarity) between H and A. From this, we shall particularly derive applications for decision making under uncertainty (including tests).

### 2.2. Connections to Time Series of Counts

It is well known that a Galton-Watson process with Poisson offspring (with parameter $\beta_{•}$) and Poisson immigration (with parameter $\alpha_{•}$) is “distributionally” equal to each of the following models (listed in “tree-type” chronological order):

(M1) a Poissonian *Generalized Integer-valued Autoregressive process* GINAR(1) in the sense of Gauthier & Latour [67] (see also Dion, Gauthier & Latour [44], Latour [68], as well as Grunwald et al. [45]), that is, a first-order autoregressive times series with Poissonian thinning (with parameter $\beta_{•}$) and Poissonian innovations (with parameter $\alpha_{•}$);
(M2) Poissonian *first order Conditional Linear Autoregressive model* (Poissonian CLAR(1)) in the sense of Grunwald et al. [45] (and earlier preprints thereof) (since the conditional expectation is $EP_{•}\left[X_{n}|F_{n-1}\right]=\alpha_{•}+\beta_{•}\cdot X_{n-1}$); this can be equally seen as Poissonian autoregressive *Generalized Linear Model* GLM with identity link function (cf. [45] as well as Chapter 4 of Kedem & Fokianos [46]), that is, an autoregressive GLM with Poisson distribution as random component and the identity link as systematic component;
  the same model was used (and generalized)
  (M2i) under the name BIN(1) by Rydberg & Shephard [69] for the description of the number $X_{n}$ of stock transactions/trades recorded up to time *n*;
  (M2ii) under the name *Poisson autoregressive model* PAR(1) by Brandt & Williams [70] for the description of event counts in political and other social science applications;
  (M2iii) under the name *Autoregressive Conditional Poisson model* ACP(1,0) by Heinen [71];
  (M2iv) by Held, Höhle & Hofmann [47] as well as Held et al. [72], as a description of the time-evolution of counts from infectious disease surveillance databases, where $\beta_{•}$ (respectively, $\alpha_{•}$) is interpreted as driving parameter of epidemic (respectively, endemic) component; in principle, this type of modelling can be also implicitly recovered as a special case of the epidemics-treating work of Finkenstädt, Bjornstad & Grenfell [73], by assuming trend- and season-neglecting (e.g., intra-year) measles data in urban areas of about 10 million people (provided that their population size approximation extends linearly);
  (M2v) under the name *integer-valued Generalized Autoregressive Conditional Heteroscedastic model* INGARCH(1,0) by Ferland, Latour & Oraichi [74] (since the conditional variance is $VarP_{•}\left[X_{n}|F_{n-1}\right]=\alpha_{•}+\beta_{•}\cdot X_{n-1}$), see also Weiß [75]; this has been refinely named as INARCH(1) model by Weiß [76,77], and frequently applied thereafter; for an “overlapping-generation type” interpretation of the INARCH(1) model, which is an adequate description for the time-evolution of overdispersed counts with an autoregressive serial dependence structure, see Weiß & Testik [78]; for a corresponding comprehensive recent survey (also to more general count time series), the reader is referred to the book of Weiß [48];

Moreover, according to the general considerations of Grunwald et al. [45], the Poissonian Galton-Watson model with immigration may possibly be “distributionally equal” to an integer-valued autoregressive model with random coefficient (thinning).

Nowadays, besides the name *homogeneous Galton-Watson model with immigration GWI*, the name *INARCH(1)* seems to be the most used one, and we follow this terminology (with emphasis on GWI). Typical features of the above-mentioned models (M1) to (M2v), are the use of Z as the set of times, and the assumptions $\alpha_{•}>0$ as well as $\beta_{•}\in ]0,1[$, which guarantee stationarity and ergodicity (see above). In contrast, we employ $N_{0}$ as the set of times, degenerate (and thus, non-equilibrium) starting distribution, and arbitrary $\alpha_{•}\geq 0$ as well as $\beta_{•}>0$. For such a situation, as explained above, we quantitatively compare two competing GWI models H and A with respective parameter sets $\left(\beta_{H},\alpha_{H}\right)$ and $\left(\beta_{A},\alpha_{A}\right)$. Since–as can be seen e.g., in (29) below—we basically employ only (conditionally) distributional ingredients, such as the corresponding likelihood ratio (see e.g., (13) to (15), (27) to (29) below), *all the results of the Section 3, Section 4, Section 5 and Section 6 can be immediately carried over to the above-mentioned time-series contexts* (where we even allow for non-stationarities, in fact we start with a one-point/Dirac distribution); for the sake of brevity, in the rest of the paper this will not be mentioned explicitly anymore.

Notice that a Poissonian GWI as well as all models (M1) and (M2) are–despite of their *conditional* Poisson law– typically overdispersed since

$$EP_{•}\left[X_{n}\right]=\alpha_{•}+\beta_{•}\cdot EP_{•}\left[X_{n-1}\right]\leq \alpha_{•}+\beta_{•}\cdot EP_{•}\left[X_{n-1}\right]+\beta_{•}^{2}\cdot VarP_{•}\left[X_{n-1}\right]=VarP_{•}\left[X_{n}\right],\;n\in N\backslash \left\{1\right\},$$

with equality iff (i.e., if and only if) $\alpha_{•}=0$ (NI) and $X_{n-2}=0$ (extinction at n−2 with n≥3).

### 2.3. Applicability to Epidemiology

The above-mentioned framework can be used for any of the numerous fields of applications of discrete-time branching processes, and of the closely related INARCH(1) models. For the sake of brevity, we explain this—as a kind of running-example—in detail for the currently highly important context of the epidemiology of infectious diseases. For insightful non-mathematical introductions to the latter, see e.g., Kaslow & Evans [79], Osterholm & Hedberg [80]; for a first entry as well as overviews on modelling, the reader is referred to e.g., Grassly & Fraser [81], Keeling & Rohani [82], Yan [83,84], Britton [85], Diekmann, Heesterbeek & Britton [86], Cummings & Lessler [87], Just et al. [88], Britton & Giardina [89], Britton & Pardoux [43]. A survey on the particular role of branching processes in epidemiology can be found e.g., in Jacob [41].

Undoubtedly, by nature, the spreading of an infectious disease through a (human, animal, plant) population is a branching process with possible immigration. Indeed, typically one has the following mechanism:

(D1) at some time $t_{k}^{E}$–called the time of exposure (moment of infection)—an individual *k* of a specified population is infected in a wide sense, i.e., entered/invaded/colonized by a number of transmissible disease-causative pathogens (etiologic agents such as viruses, bacteria, protozoans and other parasites, subviruses (e.g., prions and plant viroids), etc.); the individual is then a *host* (of pathogens);
(D2) depending on the level of immunity and some other factors, these pathogens may multiply/replicate within the host to an extent (over a threshold number) such that at time $t_{k}^{I}$ some of the pathogens start to leave their host (*shedding of pathogens*); in other words, the individual *k* becomes *infectious* at the time $t_{k}^{I}$ of *onset of infectiousness*. Ex post, one can then say that the individual became infected in the narrow sense at earlier time $t_{k}^{E}$ and call it a *primary case*. The time interval $[t_{k}^{E},t_{k}^{I}[$ is called the *latent/latency/pre-infectious period* of *k*, and $t_{k}^{I}-t_{k}^{E}$ its duration (in some literature, there is no verbal distinction between them); notice that $t_{k}^{I}$ may differ from the time $t_{k}^{OS}$ of *onset (first appearance) of symptoms*, which leads to the so-called *incubation period* $[t_{k}^{E},t_{k}^{OS}[$; if $t_{k}^{I}<t_{k}^{OS}$ then $[t_{k}^{I},t_{k}^{OS}[$ is called the *pre-symptomatic period*;
(D3) as long as the individual *k* stays infectious, by shedding of pathogens it may infect in a narrow sense a random number $Y_{k}\in N_{0}$ of other individuals which are *susceptible* (i.e., neither immune nor already infected in a narrow sense), where the distribution of $Y_{k}$ depends on the individual’s (natural, voluntary, forced) behaviour, its environment, as well as some other factors e.g., connected with the type of pathogen transmission; the newly infected individuals are called *offspring of k*, and *secondary cases* if they are from the same specified population or *exportations* if they are from a different population; from the view of the latter, these infections are *imported* cases and thus can be viewed as *immigrants*;
(D4) at the time $t_{k}^{R}$ of *cessation of infectiousness*, the individual stops being infectious (e.g., because of recovery, death, or total isolation); the time interval $[t_{k}^{I},t_{k}^{R}[$ is called the *period of infectiousness (also period of communicability, infectious/infective/shedding/contagious period)* of *k*, and $t_{k}^{R}-t_{k}^{I}$ its duration (in some literature, there is no verbal distinction between them); notice that $t_{k}^{R}$ may differ from the time $t_{k}^{CS}$ of *cessation (last appearance) of symptoms* which leads to the so-called *sickness period* $[t_{k}^{OS},t_{k}^{CS}[$;
(D5) this branching mechanism continues within the specified population until there are no infectious individuals and also no importations anymore (eradication, full extinction, total elimination)– up to a specified final time (which may be large or even infinite);

All the above-mentioned times $t_{k}^{\cdot }$ and time intervals are random, by nature. Two further connected quantities are also important for modelling (see e.g., Yan & Chowell [84] (p. 241ff), including a history of corresponding terminology). Firstly, the *generation interval* (generation time, transmission interval) is the time interval from the onset of infectiousness in a primary case (called the infector) to the onset of infectiousness in a secondary case (called the infectee) infected by the primary case; clearly, the generation interval is random, and so is its duration (often, the (population-)mean of the latter is also called generation interval). Typically, generation intervals are important ingredients of branching process models of infectious diseases. Secondly, the *serial interval* describes time interval from the onset of symptoms in a primary case to the onset of symptoms in a secondary case infected by the primary case. By nature, the serial interval is random, and so is its duration (often, the (population-)mean of the latter is also called serial interval). Typically, the serial interval is easier to observe than the generation interval, and thus, the latter is often approximately estimated from data of the former. For further investigations on generation and serial intervals, the reader is referred to e.g., Fine [90], Svensson [91,92], Wallinga & Lipsitch [93], Forsberg White & Pagano [94], Nishiura [95], Scalia Tomba et al. [96], Trichereau et al. [97], Vink, Bootsma & Wallinga [98], Champredon & Dushoff [99], Just et al. [88], and–especially for the novel COVID-19 pandemics—An der Heiden & Hamouda [100], Ferretti et al. [101], Ganyani et al. [102], Li et al. [103], Nishiura, Linton & Akhmetzhanov [104], Park et al. [105].

With the help of the above-mentioned *individual* ingredients, one can aggregatedly build numerous different *population-wide* models of infectious diseases in discrete time as well as in continuous time; the latter are typically observed only in discrete-time steps (discrete-time sampling), and hence in the following we concentrate on discrete-time modelling (of the real or the observational process). In fact, we confine ourselves to the important task of modelling the evolution $n\mapsto X_{n}$ of the number of *incidences* at “stage” *n*, where *incidence* refers to the number of *new* infected/infectious individuals. Here, *n* may be a generation number where, inductively, n=0 refers to the generation of the first appearing primary cases in the population (also called *initial importations*), and *n* refers to the generation of offsprings of all individuals of generation n−1. Alternatively, *n* may be the index of a physical (“calender”) point of time $t_{n}$, which may be deterministic or random; e.g., ${\left(t_{n}\right)}_{n\in N}$ may be a strictly increasing series of (i) equidistant deterministic time points (and thus, one can identify $t_{n}=n$ in appropriate time units such as days, weeks, bi-weeks, months), or (ii) non-equidistant deterministic time points, or (iii) random time points (as a side remark, let us mention that in some situations, $X_{n}$ may alternatively denote the number of *prevalences* at “stage” *n*, where *prevalence* refers to the total number of infected/infectious individuals (e.g., through some methodical tricks like “self-infection”)).

In the light of this, one can loosely define an *epidemic* as the rapid spread of an infectious disease within a specified population, where the numbers $X_{n}$ of incidences are high (or much higher than expected) for that kind of population. A *pandemic* is a geographically large-scale (e.g., multicontinental or worldwide) epidemic. An *outbreak/onset* of an epidemic in the narrow sense is the (time of) change where an infectious disease turns into an epidemic, which is typically quantified by exceedance over an threshold; analogously, an *outbreak/onset* of a pandemic is the (time of) change where the epidemic turns into a pandemic. Of course, one goal of infectious-disease modelling is to quantify “early enough” the potential danger of an emerging outbreak of an epidemic or a pandemic.

Returning to possible models of the incidence-evolution $n\mapsto X_{n}$, its description may be theoretically derived from more detailed, time-finer, highly sophisticated, individual-based “mechanistic” infectious-disease models such as e.g., continuous-time suscetible-exposed-infectious-recovered (SEIR) models (see the above-mentioned introductory texts); however, as e.g., pointed out in Held et al. [72], the estimation of the correspondingly involved numerous parameters may be too ambitious for routinely collected, non-detailed disease data, such as e.g., daily/weekly counts $X_{n}$ of incidences–especially in decisive emerging/early phases of a novel disease (such as the current COVID-19 pandemic). Accordingly, in the following we assume that $X_{n}$ can be approximately described by a Poissonian Galton-Watson process with immigration respectively a (“distributionally equal”) Poissonian autoregressive Generalized Linear Model in the sense of (M2). Depending on the situation, this can be quite reasonable, for the following arguments (apart from the usual “if the data say so”). Firstly, it is well known (see e.g., Bartoszynski [33], Ludwig [34], Becker [35,36], Metz [37], Heyde [38], von Bahr & Martin-Löf [39], Ball [40], Jacob [41], Barbour & Reinert [42], Section 1.2 of Britton & Pardoux [43]) that in populations with a relatively high number of susceptible individuals and a relatively low number of infectious individuals (e.g., in a large population and in decisive emerging/early phases of the disease spreading), the incidence-evolution $n\mapsto X_{n}$ can be well approximated by a (e.g., Poissonian) Galton-Watson process with possible immigration where *n* plays the role of a *generation number*. If the above-mentioned generation interval is “nearly” deterministic (leading to nearly synchronous, non-overlapping generations)—which is the case e.g., for (phases of) Influenza A(H1N1)pdm09, Influenza A(H3N2), Rubella (cf. Vink, Bootsma & Wallinga [98]), and COVID-19 (cf. Ferretti et al. [101])—and the length of the generation interval is approximated by its mean length and the latter is tuned to be equal to the unit time between consecutive observations, then *n* plays the role of an *observation* (*surveillance*) *time*. This effect is even more realistic if the period of infectiousness is nearly deterministic and relatively short. Secondly, as already mentioned above, the spreading of an infectious disease is intrinsically a (not necessarily Poissonian Galton-Watson) branching mechanism, which may be blurred by other effects in a way that a Poissonian autoregressive Generalized Linear Model is still a reasonably fitting model for the observational process in disease surveillance. The latter have been used e.g., by Finkenstädt, Bjornstad & Grenfell [73], Held, Höhle & Hofmann [47], and Held et al. [72]; they all use non-constant parameters (e.g., to describe seasonal effects, which are however unknown in early phases of a novel infectious disease such as COVID-19). In contrast, we employ different new–namely divergence-based–statistical techniques, for which we assume constant parameters but also indicate procedures for the detection of changes; the extension to non-constant parameters is straightforward.

Returning to Galton-Watson processes, let us mention as a *side remark* that they can be also used to model the above-mentioned within-host replication dynamics (D2) (e.g., in the time-interval $[t_{k}^{E},t_{k}^{I}[$ and beyond) on a sub-cellular level, see e.g., Spouge [106], as well as Taneyhill, Dunn & Hatcher [107] for parasitic pathogens; on the other hand, one can also employ Galton-Watson processes for quantifying snowball-effect (avalanche-effect, cascade-effect) type, economic-crisis triggered consequences of large epidemics and pandemics, such as e.g., the potential spread of transmissible (i) foreclosures of homes (cf. Parnes [108]), or clearly also (ii) company insolvencies, downsizings and credit-risk downgradings; moreover, the time-evolution of integer-valued indicators concerning the spread of (rational or unwarranted) fears resp. perceived threats may be modelled, too.

Summing up things, we model the evolution $n\mapsto X_{n}$ of the number of incidences at stage *n* by a Poissonian Galton Watson process with immigration GWI

$$X_{0}\in N;\;N_{0}∋\;X_{n}=\sum_{k=1}^{X_{n-1}}Y_{n-1,k}\;+\;{\tilde{Y}}_{n},\;n\in N,\;\mathrm{cf}.\;\left(1\right),\;\left(\mathrm{GWI}1\right)-\left(\mathrm{GWI}3\right)\;\mathrm{with}\;\mathrm{law}\;P_{•},$$

(where $Y_{n-1,k}$ corresponds to the $Y_{k}$ of (D3), equipped with an additional stage-index n−1), respectively by a corresponding “distributionally equal”–possibly non-stationary– Poissonian autoregressive Generalized Linear Model in the sense of (M2); depending on the situation, we may also fix a (deterministic or random) upper time horizon other than infinity. Recall that both models are overdispersed, which is consistent with the current debate on overdispersion in connection with the current COVID-19 pandemic. In infectious-disease language, the sum $\sum_{k=1}^{X_{n-1}}Y_{n-1,k}$ can also be loosely interpreted as *epidemic component* (in a narrow sense) driven by the parameter $\beta_{•}$, and ${\tilde{Y}}_{n}$ as *endemic component* driven by the parameter $\alpha_{•}$. In fact, the offspring mean (here, $\beta_{•}$) is called *reproduction number* and plays a major role–also e.g., in the current public debate about the COVID-19 pandemic–because it crucially determines the rapidity of the spread of the disease and—as already indicated above in the second and third paragraph after (PS3)–also the probability that the epidemic/pandemic becomes (maybe temporally) extinct or at least stationary at a low level (that is, *endemic*). For this to happen, $\beta_{•}$ should be subcritical, i.e., $\beta_{•}<1$, and even better, close to zero. Of course, the size of the *importation mean* $\alpha_{•}\geq 0$ matters, too, in a secondary order.

Keeping this in mind, let us discuss on which factors the reproduction number $\beta_{•}$ and the *importation mean* $\alpha_{•}$ depend upon, and how they can be influenced/controlled. To begin with, by recalling the above-mentioned points (D1) to (D5) and by adapting the considerations of e.g., Grassly & Fraser [81] to our model, one encounters the fact that the distribution of the offspring $Y_{n-1,k}$—here driven by the reproduction number (offspring mean) $\beta_{•}$—depends on the following factors:

(B1) the *degree of infectiousness* of the individual *k*, with three major components:
  (B1a) degree of *biological* infectiousness; this reflects the within-host dynamics (D2) of the “representative” individual *k*, in particular the duration and amount of the corresponding replication and shedding/excretion of the infectious pathogens; this degree depends thus on (i) the number of host-invading pathogens (called the *initial infectious dose*), (ii) the type of the pathogen with respect to e.g., its principal capabilities of replication speed, range of spread and drug-sensitivity, (iii) features of the immune system of the host *k* including the level of innate or acquired immunity, and (iv) the interaction between the genetic determinants of disease progression in both the pathogen and the host;
  (B1b) degree of *behavioural* infectiousness; this depends on the contact patterns of an infected/infectious individual (and, if relevant, the contact patterns of intermediate hosts or vectors), in relation to the disease-specific type of route(s) of transmission of the infectious pathogens (for an overview of the latter, see e.g., Table 3 of Kaslow & Evans [79]); a long-distance-travel behaviour may also lead to the disease exportation to another, outside population (and thus, for the latter to a disease importation);
  (B1c) degree of *environmental* infectiousness; this depends on the location and environment of the host *k*, which influences the duration of outside-host survival of the pathogens (and, if relevant, of the intermediate hosts or vectors) as well as the speed and range of their outside-host spread; for instance, high temperature may kill the pathogens, high airflow or rainfall dynamics may ease their spread, etc.
(B2) the *degree of susceptibility* of uninfected individuals who have contact with *k*, with the following three major components (with similar background as their infectiousness counterparts):
  (B2a) degree of *biological* susceptibility;
  (B2b) degree of *behavioural* susceptibility;
  (B2c) degree of *environmental* susceptibility.

All these factors (B1a) to (B2c) can be principally influenced/controlled to a certain–respective–extent. Let us briefly discuss this for *human* infectious diseases, where one major goal of epidemic risk management is to operate countermeasures/interventions in order to slow down the disease transmission (e.g., by reducing the reproduction number $\beta_{•}$ to less than 1) and eventually even break the chain of transmission, for the sake of containment or mitigation; preparedness and preparation are motives, too, for instance as a part of governmental pandemic risk management.

For instance, (B1a) can be reduced or even erased through pharmaceutical interventions such as medication (if available), and preventive strengthening of the immune system through non-extreme sports activities and healthy food.

Moreover, the following exemplary control measures for (B2) can be either put into action by common-sense self-behaviour, or by large-scale public recommendations (e.g., through mass media), or by rules/requirements from authorities:

(i) personal preventive measures such as frequent washing and disinfecting of hands; keeping hands away from face; covering coughs; avoidance of handshakes and hugs with non-family-members; maintaining physical distance (e.g., of two meters) from non-family-members; wearing a face-mask of respective security degree (such as homemade cloth face mask, particulate-filtering face-piece respirator, medical (non-surgical) mask, surgical mask); self-quarantine;
(ii) environmental measures, such as e.g., cleaning of surfaces;
(iii) community measures aimed at mild or stringent social distancing, such as e.g., prohibiting/cancelling/banning gatherings of more than *z* non-family members (e.g., z=2,5,10,100,1000 in various different phases and countries during the current COVID-19 pandemic); mask-wearing (see above); closing of schools, universities, some or even all nonessential (“system-irrelevant”) businesses and venues; home-officing/work ban; home isolation of disease cases; isolation of homes for the elderly/aged (nursing homes); stay-at-home orders with exemptions, household or even general quarantine; testing & tracing; lockdown of entire cities and beyond; restricting the degrees of travel freedom/allowed mobility (e.g., local, union-state, national, international including border and airport closure). The latter also affects the mean importation rate $\alpha_{•}$, which can be controlled by vaccination programs in “outside populations”, too.

As far as the degree of *biological* susceptibility (B2a) is concerned, one obvious therapeutic countermeasure is a mass vaccination program/campaign (if available).

In case of *highly virulent* infectious diseases causing epidemics and pandemics with substantial *fatality rates*, some of the above-mentioned control strategies and countermeasures may (have to) be “drastic” (e.g., lockdown), and thus imply considerable social and economic costs, with a huge impact and potential danger of triggering severe social, economic and political disruptions.

In order to prepare corresponding suggestions for decisions about appropriate control measures (e.g., public policies), it is therefore important–especially for a novel infectious disease such as the current COVID-19 pandemic–to have a model for the time-evolution of the incidences in (i) a natural (basically uncontrolled) set-up, as well as in (ii) the control set-ups under consideration. As already mentioned above, we assume that all these situations can be distilled into an incidence evolution $n\mapsto X_{n}$ which follows a Poissonian Galton-Watson process with respectively different parameter pairs $\left(\beta_{•},\alpha_{•}\right)$. Correspondingly, we always compare two alternative models (H) and (A) with parameter pairs $\left(\beta_{H},\alpha_{H}\right)$ and $\left(\beta_{A},\alpha_{A}\right)$ which reflect either a “pure” statistical uncertainty (under the *same* uncontrolled or controlled set-up), or the uncertainty between two *different* potential control set-ups (for the sake of assessing the potential impact/efficiency of some planned interventions, compared with alternative ones); the economic impact can be also taken into account, within a Bayesian decision framework discussed in Section 2.5 below. As will be explained in the next subsections, we achieve such comparisons by means of density-based dissimilarity distances/divergences and related quantities thereof.

From the above-mentioned detailed explanations, it is immediately clear that for the described epidemiological context one should investigate *all* types of criticality and importation means for the therein involved two Poissonian Galton-Watson processes with/without immigration (respectively the equally distributed INARCH(1) models); in particular, this motivates (or even “justifies”) the necessity of the very lengthy detailed studies in the Section 3, Section 4, Section 5, Section 6 and Section 7 below.

### 2.4. Information Measures

Having two competing models (H) and (A) at stake, it makes sense to study questions such as “how far are they apart?” and thus “how dissimilar are they?”. This can be quantified in terms of divergences in the sense of directed (i.e., not necessarily symmetric) distances, where usually the triangular inequality fails. Let us first discuss our employed divergence subclasses in a *general* set-up of two *equivalent* probability measures $P_{H}$, $P_{A}$ on a measurable space $\left(\Omega ,F\right)$. In terms of the parameter λ∈R, the *power divergences*—also known as Cressie-Read divergences, relative Tsallis entropies, or generalized cross-entropy family– are defined as (see e.g., Liese & Vajda [1,10])

(2) $$\begin{array}{c} 0\leq \;I_{\lambda }\left(P_{A}∥P_{H}\right)\;:=\;\left\{\begin{array}{cc} I\left(P_{A}∥P_{H}\right), & \;\mathrm{if}\;\;\lambda =1,\\ \frac{1}{\lambda (\lambda -1)}\left(H_{\lambda }\left(P_{A}∥P_{H}\right)-1\right), & \;\mathrm{if}\;\;\lambda \in R\backslash \{0,1\},\\ I\left(P_{H}∥P_{A}\right), & \;\mathrm{if}\;\;\lambda =0, \end{array}\right. \end{array}$$

where

(3) $$I\left(P_{A}∥P_{H}\right)\;:=\;\int p_{A}\;\log\frac{p_{A}}{p_{H}}\;d\mu \;\geq \;0$$

is the *Kullback-Leibler information divergence* (also known as *relative entropy*) and

(4) $$H_{\lambda }\left(P_{A}∥P_{H}\right)\;:=\;\int_{\Omega }p_{A}^{\lambda }\;p_{H}^{1-\lambda }\;d\mu \;\geq \;0$$

is the *Hellinger integral of order* λ∈R\{0,1}; for this, we assume as usual without loss of generality that the probability measures $P_{H}$, $P_{A}$ are dominated by some σ–finite measure μ, with densities

(5) $$p_{A}\;=\;\frac{dP_{A}}{d\mu }\;\mathrm{and}\;p_{H}\;=\;\frac{dP_{H}}{d\mu }$$

defined on Ω (the zeros of $p_{H}$, $p_{A}$ are handled in (3) and (4) with the usual conventions). Clearly, for λ∈{0,1} one trivially gets

$$H_{0}\left(P_{A}∥P_{H}\right)\;=\;H_{1}\left(P_{A}∥P_{H}\right)\;=\;1\;.$$

The Kullback-Leibler information divergences (relative entropies) in (2) and (3) can alternatively be expressed as (see, e.g., Liese & Vajda [1])

(6) $$I\left(P_{A}∥P_{H}\right)\;=\;\lim_{\lambda ↗1}\frac{1-H_{\lambda }\left(P_{A}∥P_{H}\right)}{\lambda (1-\lambda )},\;I\left(P_{H}∥P_{A}\right)\;=\;\lim_{\lambda ↘0}\frac{1-H_{\lambda }\left(P_{A}∥P_{H}\right)}{\lambda (1-\lambda )}\;.$$

Apart from the Kullback-Leibler information divergence (relative entropy), other prominent examples of power divergences are the squared Hellinger distance $\frac{1}{2}\;I_{1/2}\left(P_{A}∥P_{H}\right)$ and Pearson’s $\chi^{2}–$divergence $2\;I_{2}\left(P_{A}∥P_{H}\right)$; the Hellinger integral $H_{1/2}\left(P_{A}∥P_{H}\right)$ is also known as (multiple of) the *Bhattacharyya coefficent*. Extensive studies about basic and advanced general facts on power divergences, Hellinger integrals and the related Renyi divergences of order λ∈R\{0,1}

(7) $$0\leq \;R_{\lambda }\left(P_{A}∥P_{H}\right)\;:=\;\frac{1}{\lambda (\lambda -1)}\;\log H_{\lambda }\left(P_{A}∥P_{H}\right)\;,\;\mathrm{with}\log\;0=-\infty ,$$

can be found e.g., in Liese & Vajda [1,10], Jacod & Shiryaev [24], van Erven & Harremoes [20] (as a side remark, $R_{1/2}\left(P_{A}∥P_{H}\right)$ is also known as (multiple of) *Bhattacharyya distance*). For instance, the integrals in (3) and (4) do not depend on the choice of μ. Furthermore, one has the skew symmetries

(8) $$H_{\lambda }\left(P_{A}∥P_{H}\right)\;=\;H_{1-\lambda }\left(P_{H}∥P_{A}\right)\;,\;\mathrm{as}\;\mathrm{well}\;\mathrm{as}\;I_{\lambda }\left(P_{A}∥P_{H}\right)\;=\;I_{1-\lambda }\left(P_{H}∥P_{A}\right),$$

for all λ∈R (see e.g., Liese & Vajda [1]). As far as finiteness is concerned, for λ∈]0,1[ one gets the rudimentary bounds

(9) $$\begin{array}{c} 0\;<\;H_{\lambda }\left(P_{A}∥P_{H}\right)\;\leq \;1\;,\;\mathrm{and}\;\mathrm{equivalently}, \end{array}$$

(10) $$\begin{array}{c} 0\;\leq \;I_{\lambda }\left(P_{A}∥P_{H}\right)\;=\;\frac{1-H_{\lambda }\left(P_{A}∥P_{H}\right)}{\lambda (1-\lambda )}\;<\;\frac{1}{\lambda (1-\lambda )}\;, \end{array}$$

where the lower bound in (10) (upper bound in (9)) is achieved iff $P_{A}=P_{H}$. For λ∈R\]0,1[, one gets the bounds

(11) $$0\;\leq \;I_{\lambda }\left(P_{A}∥P_{H}\right)\;\leq \;\infty \;,\;\mathrm{and}\;\mathrm{equivalently},\;1\;\leq \;H_{\lambda }\left(P_{A}∥P_{H}\right)\;\leq \;\infty \;,$$

where, in contrast to above, both the lower bound of $H_{\lambda }\left(P_{A}∥P_{H}\right)$ and the lower bound of $I_{\lambda }\left(P_{A}∥P_{H}\right)$ is achieved iff $P_{A}=P_{H}$; however, the power divergence $I_{\lambda }\left(P_{A}∥P_{H}\right)$ and Hellinger integral $H_{\lambda }\left(P_{A}∥P_{H}\right)$ might be infinite, depending on the particular setup.

The Hellinger integrals can be also used for bounds of the well-known *total variation*

$$0\;\leq \;V(P_{A}∥P_{H})\;:=\;2\underset{A\in F}{\sup}\left\{P_{A}\left(A\right)-P_{H}\left(A\right)\right\}\;=\;\int_{\Omega }\left|p_{A}-p_{H}\right|d\mu \;,$$

with $p_{A}$ and $p_{H}$ defined in (5). Certainly, the total variation is one of the best known statistical distances, see e.g., Le Cam [109]. For arbitrary λ∈]0,1[ there holds (cf. Liese & Vajda [1])

$$1-\frac{V(P_{A}∥P_{H})}{2}\leq H_{\lambda }(P_{A}∥P_{H})\leq {\left(1+\frac{V(P_{A}∥P_{H})}{2}\right)}^{\max\{\lambda ,1-\lambda \}}{\left(1-\frac{V(P_{A}∥P_{H})}{2}\right)}^{\min\{\lambda ,1-\lambda \}}.$$

From this together with the particular choice $\lambda =\frac{1}{2}$, we can derive the fundamental universal bounds

(12) $$2\;\left(1-H_{\frac{1}{2}}(P_{A}∥P_{H})\right)\;\leq \;V(P_{A}∥P_{H})\;\leq \;2\;\sqrt{1-{\left(H_{\frac{1}{2}}(P_{A}∥P_{H})\right)}^{2}}\;.$$

We apply these concepts to our setup of Section 2.1 with two competing models (H) and (A) of Galton-Watson processes with immigration, where one can take $\Omega \subset N_{0}^{N_{0}}$ to be the space of all paths of ${\left(X_{n}\right)}_{n\in N}$. More detailed, in terms of the extinction set B:={τ<∞} and the parameter-set notation (PS1) to (PS3), it is known that for $P_{\mathrm{SP}}$ the two laws $P_{H}$ and $P_{A}$ are equivalent, whereas for $P_{\mathrm{NI}}$ the two restrictions ${\left.P_{H}\right|}_{B}$ and ${\left.P_{A}\right|}_{B}$ are equivalent (see e.g., Lemma 1.1.3 of Guttorp [52]); with a slight abuse of notation we shall henceforth omit ${\left.\;\right|}_{B}$. Consistently, for fixed time $n\in N_{0}$ we introduce $P_{A,n}:={\left.P_{A}\right|}_{F_{n}}$ and $P_{H,n}:={\left.P_{H}\right|}_{F_{n}}$ as well as the corresponding Radon-Nikodym-derivative (likelihood ratio)

(13) $$Z_{n}\;:=\;\frac{dP_{A,n}}{dP_{H,n}}\;,$$

where ${\left(F_{n}\right)}_{n\in N}$ denotes the corresponding canonical filtration generated by $X:={\left(X_{n}\right)}_{n\in N}$; in other words, $F_{n}$ reflects the “process-intrinsic” information known at stage *n*. Clearly, $Z_{0}=1$. By choosing the reference measure $\mu =P_{H,n}$ one obtains from (4) the Hellinger integral $H_{\lambda }\left(P_{A,0}∥P_{H,0}\right)=1$, as well as and for all n∈N

(14) $$\begin{array}{c} \;H_{\lambda }\left(P_{A,n}∥P_{H,n}\right)\;=\;EP_{H,n}\left[{\left(Z_{n}\right)}^{\lambda }\right], \end{array}$$

(15) $$\begin{array}{c} \;I\left(P_{A,n}∥P_{H,n}\right)\;=\;EP_{A,n}\left[\log Z_{n}\right], \end{array}$$

from which one can immediately build $I_{\lambda }\left(P_{A,n}∥P_{H,n}\right)$ (λ∈R) respectively $R_{\lambda }\left(P_{A,n}∥P_{H,n}\right)$ (λ∈R\{0,1}) respectively bounds of $V\left(P_{A,n}∥P_{H,n}\right)$ via (2) respectively (7) respectively (12).

The outcoming values (respectively bounds) of $H_{\lambda }\left(P_{A,n}∥P_{H,n}\right)$ are quite diverse and depend on the choice of the involved parameter pairs $\left(\beta_{H},\alpha_{H}\right)$, $\left(\beta_{A},\alpha_{A}\right)$ as well as λ; the exact details will be given in the Section 3 and Section 6 below.

Before we achieve this, in the following we explain how the outcoming dissimilarity results can be applied to Bayesian testing and more general Bayesian decision making, as well as to Neyman-Pearson testing.

### 2.5. Decision Making under Uncertainty

Within the above-mentioned context of two competing models (H) and (A) of Galton-Watson processes with immigration, let us briefly discuss how knowledge about the time-evolution of the Hellinger integrals $H_{\lambda }\left(P_{A,n}∥P_{H,n}\right)$–or equivalently, of the power divergences $I_{\lambda }\left(P_{A,n}∥P_{H,n}\right)$, cf. (2)—can be used in order to take decisions under uncertainty, within a framework of Bayesian decision making BDM, or alternatively, of Neyman-Pearson testing NPT.

In our context of BDM, we decide between an action $d_{H}$ “associated with” the (say) hypothesis law $P_{H}$ and an action $d_{A}$ “associated with” the (say) alternative law $P_{A}$, based on the sample path observation $X_{n}:=\left\{X_{l}:\;l\in \left\{0,1,\ldots ,n\right\}\;\right\}$ of the GWI-generation-sizes (e.g., infectious-disease incidences, cf. Section 2.3) up to observation horizon n∈N. Following the lines of Stummer & Vajda [15] (adapted to our branching process context), for our BDM let us consider as admissible decision rules $\delta_{n}:\Omega_{n}\mapsto \left\{d_{H},d_{A}\right\}$ the ones generated by all path sets $G_{n}\in \Omega_{n}$ (where $\Omega_{n}$ denotes the space of all possible paths of ${\left(X_{k}\right)}_{k\in \{1,\ldots ,n\}}$) through

$$\begin{array}{ccc} \delta_{n}\left(X_{n}\right)\;:=\;\delta_{G_{n}}\left(X_{n}\right) & := & \left\{\begin{array}{cc} d_{A}, & \mathrm{if}\;X_{n}\in G_{n},\\ d_{H}, & \mathrm{if}\;X_{n}\notin G_{n}, \end{array}\right. \end{array}$$

as well as loss functions of the form

(16) $$\left(\begin{array}{cc} L(d_{H},H) & L(d_{H},A)\\ L(d_{A},H) & L(d_{A},A) \end{array}\right)\;:=\;\left(\begin{array}{cc} 0 & L_{A}\\ L_{H} & 0 \end{array}\right)$$

with pregiven constants $L_{A}>0$, $L_{H}>0$ (e.g., arising as bounds from quantities in worst-case scenarios); notice that in (16), $d_{H}$ is assumed to be a zero-loss action under H and $d_{A}$ a zero-loss action under A. Per definition, the *Bayes decision rule* $\delta_{G_{n,\min}}$ minimizes–over $G_{n}$—the *mean decision loss*

(17) $$\begin{array}{cc} L(\delta_{G_{n}}):= & p_{H}^{\mathrm{prior}}\cdot L_{H}\cdot Pr\left(\delta_{G_{n}}\left(X_{n}\right)=d_{A}|H\right)\;+\;p_{A}^{\mathrm{prior}}\cdot L_{A}\cdot Pr\left(\delta_{G_{n}}\left(X_{n}\right)=d_{H}|A\right)\\ = & p_{H}^{\mathrm{prior}}\cdot L_{H}\cdot P_{H,n}\left(G_{n}\right)\;+\;p_{A}^{\mathrm{prior}}\cdot L_{A}\cdot P_{A,n}\left(\Omega_{n}-G_{n}\right) \end{array}$$

for given prior probabilities $p_{H}^{\mathrm{prior}}=Pr\left(H\right)\in ]0,1[$ for H and $p_{A}^{\mathrm{prior}}:=Pr\left(A\right)=1-p_{H}^{\mathrm{prior}}$ for A. As a side remark let us mention that, in a certain sense, the involved model (parameter) uncertainty expressed by the “superordinate” Bernoulli-type law $Pr=Bin(1,p_{H}^{\mathrm{prior}})$ can also be reinterpreted as a rudimentary static random environment caused e.g., by a random Bernoulli-type external static force. By straightforward calculations, one gets with (13) the minimizing path set $G_{n,\min}=\left\{Z_{n}\geq \frac{p_{H}^{\mathrm{prior}}L_{H}}{p_{A}^{\mathrm{prior}}L_{A}}\right\}$ leading to the *minimal mean decision loss*, i.e., the *Bayes risk*,

(18) $$\begin{array}{ccc} R_{n}\;:=\;\min_{G_{n}}L\left(\delta_{G_{n}}\right)\;=\;L\left(\delta_{G_{n,\min}}\right) & = & \int_{\Omega_{n}}\min\left\{p_{H}^{\mathrm{prior}}L_{H},p_{A}^{\mathrm{prior}}L_{A}\;Z_{n}\right\}dP_{H,n}\;. \end{array}$$

Notice that—by straightforward standard arguments—the *alternative* decision procedure

$$\mathrm{take}\;\mathrm{action}\;d_{A}\;(\mathrm{resp}.\;d_{H})\;\;\;\mathrm{if}\;\;\;L_{H}\cdot p_{H}^{\mathrm{post}}\;\left(X_{n}\right)\;\leq (\mathrm{resp}.\;>)\;\;L_{A}\cdot p_{A}^{\mathrm{post}}\;\left(X_{n}\right)$$

with posterior probabilities $p_{H}^{\mathrm{post}}\;\left(X_{n}\right):=\frac{p_{H}^{\mathrm{prior}}}{\left(1-p_{H}^{\mathrm{prior}}\right)\cdot Z_{n}\;\left(X_{n}\right)\;+\;p_{H}^{\mathrm{prior}}}=:1-p_{A}^{\mathrm{post}}\;\left(X_{n}\right)$, leads exactly to the same actions as $\delta_{G_{n,\min}}$. By adapting the Lemma 6.5 of Stummer & Vajda [15]—which on general probability spaces gives *fundamental universal* inequalities relating Hellinger integrals (or equivalently, power divergences) and Bayes risks—one gets for all $L_{H}>0$, $L_{A}>0$, $p_{H}^{\mathrm{prior}}\in ]0,1[$, λ∈]0,1[ and n∈N the upper bound

(19) $$R_{n}\;\leq \;\Lambda_{A}^{\lambda }\;\Lambda_{H}^{1-\lambda }\;H_{\lambda }\left(P_{A,n}∥P_{H,n}\right)\;,\;\mathrm{with}\Lambda_{H}:=p_{H}^{prior}L_{H},\;\Lambda_{A}:=\left(1-p_{H}^{prior}\right)L_{A},$$

as well as the lower bound

$${\left(R_{n}\right)}^{\min\{\lambda ,1-\lambda \}}\cdot {\left(\Lambda_{H}+\Lambda_{A}-R_{n}\right)}^{\max\{\lambda ,1-\lambda \}}\;\geq \;\Lambda_{A}^{\lambda }\;\Lambda_{H}^{1-\lambda }\;H_{\lambda }\left(P_{A,n}∥P_{H,n}\right)\;$$

which implies in particular the “direct” lower bound

(20) $$R_{n}\;\geq \;\frac{\Lambda_{A}^{\max\{1,\frac{\lambda }{1-\lambda }\}}\;\Lambda_{H}^{\max\{1,\frac{1-\lambda }{\lambda }\}}}{{\left(\Lambda_{A}+\Lambda_{H}\right)}^{\max\{\frac{\lambda }{1-\lambda },\frac{1-\lambda }{\lambda }\}}}\cdot {\left(H_{\lambda }\left(P_{A,n}∥P_{H,n}\right)\right)}^{\max\{\frac{1}{\lambda },\frac{1}{1-\lambda }\}}\;.$$

By using (19) (respectively (20)) together with the exact values and the upper (respectively lower) bounds of the Hellinger integrals $H_{\lambda }\left(P_{A,n}∥P_{H,n}\right)$ derived in the following sections, we end up with upper (respectively lower) bounds of the Bayes risk $R_{n}$. Of course, with the help of (2) the bounds (19) and (20) can be (i) immediately rewritten in terms of the power divergences $I_{\lambda }\left(P_{A,n}∥P_{H,n}\right)$ and (ii) thus be *directly* interpreted in terms of dissimilarity-size arguments. As a side-remark, in such a Bayesian context the λ–order Hellinger integral $H_{\lambda }\left(P_{A,n}∥P_{H,n}\right)\;=\;EP_{H,n}\left[{\left(Z_{n}\right)}^{\lambda }\right]$ (cf. (14)) can be also interpreted as λ–order Bayes-factor moment (with respect to $P_{H,n}$), since $Z_{n}=Z_{n}\;\left(X_{n}\right)=\frac{p_{A}^{\mathrm{post}}\;\left(X_{n}\right)}{p_{H}^{\mathrm{post}}\;\left(X_{n}\right)}/\frac{p_{A}^{\mathrm{prior}}}{p_{H}^{\mathrm{prior}}}$ is the Bayes factor (i.e., the posterior odds ratio of (A) to (H), divided by the prior odds ratio of (A) to (H)).

At this point, the potential applicant should be warned about the *usual way of* asynchronous decision making, where one first *tests* (A) versus (H) (i.e., $L_{A}=L_{H}=1$ which leads to 0–1 losses in (16)) and afterwards, based on the outcoming result (e.g., in favour of (A)), takes the attached economic decision (e.g., $d_{A}$); this can lead to distortions compared with synchronous decision making with “full” monetary losses $L_{A}$ and $L_{H}$, as is shown in Stummer & Lao [16] within an economic context in connection with discrete approximations of financial diffusion processes (they call this distortion effect a *non-commutativity between Bayesian statistical and investment decisions*).

For different types of–mainly parameter estimation (squared-error type loss function) concerning—Bayesian analyses based on GW(I) generation size observations, see e.g., Jagers [56], Heyde [38], Heyde & Johnstone [110], Johnson et al. [111], Basawa & Rao [60], Basawa & Scott [61], Scott [112], Guttorp [52], Yanev & Tsokos [113], Mendoza & Gutierrez-Pena [114], and the references therein.

Within our running-example epidemiological context of Section 2.3, let us briefly discuss the role of the above-mentioned losses $L_{A}$ and $L_{H}$. To begin with, as mentioned above the *unit-free* choice $L_{A}=L_{H}=1$ corresponds to *Bayesian testing*. Recall that this concerns with two alternative infectious-disease models (H) and (A) with parameter pairs (recall the interpretation of $\beta_{•}$ as reproduction number and $\alpha_{•}$ as importation mean) $\left(\beta_{H},\alpha_{H}\right)$ and $\left(\beta_{A},\alpha_{A}\right)$ which reflect either a “pure” statistical uncertainty (under the *same* uncontrolled or controlled set-up), or the uncertainty between two *different* potential control set-ups (for the sake of assessing the potential impact/efficiency of some planned interventions, compared with alternative ones). As far as *non-unit-free*–e.g., macroeconomic or monetary–losses is concerned, recall that some of the above-mentioned control strategies (countermeasures, public policies, governmental pandemic risk management plans) may imply considerable social and economic costs, with a huge impact and potential danger of triggering severe social, economic and political disruptions; a corresponding tradeoff between health and economic issues can be incorporated by choosing $L_{A}$ and $L_{H}$ to be (e.g., monetary) values which reflect estimates or upper bounds of losses due to wrong decisions, e.g., if at stage *n* due to the observed data one erroneously thinks (reinforced by fear) that a novel infectious disease (e.g., COVID-19) will lead (or re-emerge) to a severe pandemic and consequently decides for a lockdown with drastic future economic consequences, versus, if one erroneously thinks (reinforced by carelessness) that the infectious disease is (or stays) non-severe and consequently eases some/all control measures which will lead to extremely devastating future economic consequences. For the estimates/bounds of $L_{A}$ and $L_{H}$, one can e.g., employ (i) the comprehensive stochastic studies of Feicht & Stummer [115] on the quantitative degree of elasticity and speed of recovery of economies after a sudden macroeconomic disaster, or (ii) the more short-term, German-specific, scenario-type (basically non-stochastic) studies of Dorn et al. [116,117] in connection with the current COVID-19 pandemic.

Of course, the above-mentioned Bayesian decision procedure can be also operated in *sequential way*. For instance, suppose that we are encountered with a novel infectious disease (e.g., COVID-19) of non-negligible fatality rate and let (A) reflect a “potentially dangerous” infectious-disease-transmission situation (e.g., a reproduction number of substantially supercritical case $\beta_{A}=2$, and an importation mean of $\alpha_{A}=10$, for *weekly* appearing new incidence-generations) whereas (H) describes a “relatively harmless/mild” situation (e.g., a substantially subcritical $\beta_{H}=0.5$, $\alpha_{H}=0.2$). Moreover, let $d_{A}$ respectively $d_{H}$ denote (non-quantitatively) the decision/action to accept (A) respectively (H). It can then be reasonable to decide to stop the observation process $n\mapsto X_{n}$ (also called *surveillance* or *online-monitoring*) of incidence numbers at the first time at which $n\mapsto Z_{n}=Z_{n}\left(X_{n}\right)$ exceeds the threshold $p_{H}^{\mathrm{prior}}/p_{A}^{\mathrm{prior}}$; if this happens, one takes $d_{A}$ as decision (and e.g., declare the situation as *occurrence of an epidemic outbreak* and start with control/intervention measures (however, as explained above, one should synchronously involve also the potential economic losses)) whereas as long as this does not happen, one continues the observation (and implicitly takes $d_{H}$ as decision). This can be modelled in terms of the pair $\left(\tilde{\tau },d_{A}\right)$ with (random) stopping time $\tilde{\tau }:=\inf\left\{n\in N:\;Z_{n}\geq \frac{p_{H}^{\mathrm{prior}}}{p_{A}^{\mathrm{prior}}}\right\}$ (with the usual convention that the infimum of the empty set is infinity), and the corresponding decision $d_{A}$. After the time $\tilde{\tau }<\infty$ and e.g., immediate subsequent employment of some control/counter measures, one can e.g., take the old model (A) as new (H), declare a new target (A) for the desired quantification of the effectiveness of the employed control measures (e.g., a mitigation to a slightly subcritical case of $\beta_{A}=0.95$, $\alpha_{H}=0.8$), and starts to observe the new incidence numbers until the new target (A) has been reached. This can be interpreted as online-detection of a distributional change; a related comprehensive new framework for the use of divergences (even much beyond power divergences) for distributional change detection can be found e.g., in the recent work of Kißlinger & Stummer [118]. A completely different, SIR-model based, approach for the detection of change points in the spread of COVID-19 is given in Dehning et al. [119]. Moreover, other different surveillance methods can be also found e.g., in the corresponding overview of Frisen [120] and the Swedish epidemics outbreak investigations of Friesen & Andersson & Schiöler [121].

One can refine the above-mentioned sequential procedure via two (instead of one) appropriate thresholds $c_{1}<c_{2}$ and the pair $\left(\overset{˘}{\tau },\delta_{\overset{˘}{\tau }}\right)$, with the stopping time $\overset{˘}{\tau }:=\inf\left\{n\in N:\;Z_{n}\notin \left[c_{1},c_{2}\right]\right\}$ as well as corresponding decision rule

$$\begin{array}{ccc} \delta_{\overset{˘}{\tau }} & := & \;\left\{\begin{array}{c} d_{A},\;\mathrm{if}Z_{\overset{˘}{\tau }}>c_{2},\\ d_{H},\;\mathrm{if}Z_{\overset{˘}{\tau }}<c_{1}. \end{array}\right. \end{array}$$

An exact optimized treatment on the two above-mentioned sequential procedures, and their connection to Hellinger integrals (and power divergences) of Galton-Watson processes with immigration, is beyond the scope of this paper.

As a side remark, let us mention that our above-mentioned suggested method of Bayesian decision making with Hellinger integrals of GWIs differs completely from the very recent work of Brauner et al. [122] who use a Bayesian hierarchical model for the concrete, very comprehensive study on the effectiveness and burden of non-pharmaceutical interventions against COVID-19 transmission.

The power divergences $I_{\lambda }\left(P_{A,n}∥P_{H,n}\right)$ (λ∈R) can be employed also in other ways within Bayesian decision making, of statistical nature. Namely, by adapting the general lines of Österreicher & Vajda [123] (see also Liese & Vajda [10], as well as diffusion-process applications in Stummer [5,31,32]) to our context of Galton-Watson processes with immigration, we can proceed as follows. For the sake of comfortable notations, we first attach the value θ:=1 to the GWI model (A) (which has prior probability $p_{A}^{\mathrm{prior}}\in \;]0,1[$) and θ:=0 to (H) (which has prior probability $1-p_{A}^{\mathrm{prior}}$). Suppose we want to decide, in an optimal Bayesian way, which *degree of evidence* deg∈[0,1] we should attribute (according to a pregiven *loss function* LO) to the model (A). In order to achieve this goal, we choose a nonnegatively-valued loss function LO(θ,deg) defined on {0,1}×[0,1], of two types which will be specified below. The risk at stage 0 (i.e., prior to the GWI-path observations $X_{n}$), from the optimal decision about the degree of evidence deg concerning the decision parameter θ, is defined as

$${\mathrm{BR}}_{\mathrm{LO}}\left(p_{A}^{\mathrm{prior}}\right):=\min_{deg\in [0,1]}\{\;\left(1-p_{A}^{\mathrm{prior}}\right)\cdot \mathrm{LO}\left(0,deg\right)\;+\;p_{A}^{\mathrm{prior}}\cdot \mathrm{LO}\left(1,deg\right)\;\}\;,$$

which can be thus interpreted as a *minimal prior expected loss* (the minimum will always exist). The corresponding risk *posterior* to the GWI-path observations $X_{n}$, from the optimal decision about the degree of evidence deg concerning the parameter θ, is given by

$${\mathrm{BR}}_{\mathrm{LO}}^{\mathrm{post}}\left(p_{A}^{\mathrm{prior}}\right):=\int_{\Omega_{n}}{\mathrm{BR}}_{\mathrm{LO}}\left(p_{A}^{\mathrm{post}}\;\left(X_{n}\right)\right)\;(p_{A}^{\mathrm{prior}}\;dP_{A,n}\;+\;\left(1-p_{A}^{\mathrm{prior}}\right)\;dP_{H,n})\;,$$

which is achieved by the optimal decision rule (about the degree of evidence)

$$D^{*}\left(X_{n}\right)\;:=\;\arg\min_{deg\in [0,1]}\{\;\left(1-p_{A}^{\mathrm{post}}\;\left(X_{n}\right)\right)\cdot \mathrm{LO}\left(0,deg\right)\;+\;p_{A}^{\mathrm{post}}\;\left(X_{n}\right)\cdot \mathrm{LO}\left(1,deg\right)\;\}\;.$$

The corresponding *statistical information measure* (in the sense of De Groot [124])

$$\Delta {\mathrm{BR}}_{\mathrm{LO}}\left(p_{A}^{\mathrm{prior}}\right):={\mathrm{BR}}_{\mathrm{LO}}\left(p_{A}^{\mathrm{prior}}\right)-{\mathrm{BR}}_{\mathrm{LO}}^{post}\left(p_{A}^{\mathrm{prior}}\right)\;\geq 0$$

represents the *reduction of the decision risk* about the degree of evidence deg concerning the parameter θ, that can be attained by observing the GWI-path $X_{n}$ until stage *n*. For the first-type loss function $\tilde{\mathrm{LO}}\left(\theta ,deg\right):=deg-\left(2\;deg-1\right)\cdot 1_{\left\{1\right\}}\left(\theta \right)$, defined on {0,1}×[0,1] with the help of the indicator function $1_{A}\left(.\right)$ on the set *A*, one can show that

$$\begin{array}{ccc} D^{*}\left(X_{n}\right)\; & := & \;\left\{\begin{array}{c} 0,\;\mathrm{if}\;p_{A}^{\mathrm{post}}\;\left(X_{n}\right)\in \;[0,\frac{1}{2}[,\\ 1,\;\mathrm{if}\;p_{A}^{\mathrm{post}}\;\left(X_{n}\right)\in \;]\frac{1}{2},1[,\\ \mathrm{any}\;\mathrm{number}\;\mathrm{in}\;[0,1],\;\mathrm{if}\;p_{A}^{\mathrm{post}}\;\left(X_{n}\right)=\frac{1}{2}, \end{array}\right. \end{array}$$

as well as the representation formula

(21) $$I_{\lambda }\left(P_{A,n}∥P_{H,n}\right)\;=\;\int_{0}^{1}\Delta {\mathrm{BR}}_{\tilde{\mathrm{LO}}}\left(p_{A}^{\mathrm{prior}}\right)\cdot {\left(1-p_{A}^{\mathrm{prior}}\right)}^{\lambda -2}\cdot {\left(p_{A}^{\mathrm{prior}}\right)}^{-1-\lambda }\;dp_{A}^{\mathrm{prior}},\;\lambda \in R,$$

(cf. Österreicher & Vajda [123], Liese & Vajda [10], adapted to our GWI context); in other words, the power divergence $I_{\lambda }\left(P_{A,n}∥P_{H,n}\right)$ can be regarded as a *weighted-average statistical information measure* (*weighted-average decision risk reduction*). One can also use other weights of $p_{A}^{\mathrm{prior}}$ in order to get bounds of $I_{\lambda }\left(P_{A,n}∥P_{H,n}\right)$ (analogously to Stummer [5]).

For the second-type loss function ${\mathrm{LO}}_{\lambda ,\chi }\left(\theta ,deg\right):=\frac{\lambda^{\theta -1}\;{deg}^{\lambda -\theta }}{\chi^{\lambda }\;{\left(1-\chi \right)}^{1-\lambda }\;{\left(1-\lambda \right)}^{\theta }\;{\left(1-deg\right)}^{\lambda -\theta }}$ defined on {0,1}×[0,1] with parameters λ∈]0,1[ and χ∈]0,1[, one can derive the optimal decision rule

$$D^{*}\left(X_{n}\right)\;=\;p_{A}^{\mathrm{post}}\;\left(X_{n}\right)$$

as well as the representation formula as a *limit statistical information measure* (*limit decision risk reduction*)

(22) $$I_{\lambda }\left(P_{A,n}∥P_{H,n}\right)\;=\;\lim_{\chi \to p_{A}^{\mathrm{prior}}}\;\Delta {\mathrm{BR}}_{{\mathrm{LO}}_{\lambda ,\chi }}\left(p_{A}^{\mathrm{prior}}\right)\;=:\;\Delta {\mathrm{BR}}_{{\mathrm{LO}}_{\lambda ,p_{A}^{\mathrm{prior}}}}\left(p_{A}^{\mathrm{prior}}\right)$$

(cf. Österreicher & Vajda [123], Stummer [5], adapted to our GWI context).

As an alternative to the above-mentioned Bayesian-decision-making applications of Hellinger integrals $H_{\lambda }\left(P_{A,n}∥P_{H,n}\right)$, let us now briefly discuss the use of the latter for the corresponding *Neyman-Pearson* (NPT) framework with randomized tests $T_{n}:\Omega_{n}\mapsto \left[0,1\right]$ of the hypothesis $P_{H}$ against the alternative $P_{A}$, based on the GWI-generation-size sample path observations $X_{n}:=\left\{X_{l}:\;l\in \left\{0,1,\ldots ,n\right\}\;\right\}$. In contrast to (17) and (18) a Neyman-Pearson test minimizes—over $T_{n}$–the type II error probability $\int_{\Omega_{n}}\left(1-T_{n}\right)\;dP_{A,n}$ in the class of the tests for which the type I error probability $\int_{\Omega_{n}}T_{n}\;dP_{H,n}$ is at most ς∈]0,1[. The corresponding minimal type II error probability

$$E_{\varsigma }\left(P_{A,i}∥P_{H,i}\right)\;:=\;\underset{T_{i}:\int_{\Omega_{i}}\;T_{i}\;dP_{H,i}\leq \varsigma }{\inf}\;\int_{\Omega_{i}}\left(1-T_{i}\right)\;dP_{A,i}$$

can for all ς∈]0,1[, λ∈]0,1[, i∈I be bounded from above by

(23) $$E_{\varsigma }\left(P_{A,i}∥P_{H,i}\right)\leq E_{\varsigma }^{U}\left(P_{A,i}∥P_{H,i}\right):=\min\left\{\left(1-\lambda \right)\cdot {\left(\frac{\lambda }{\varsigma }\right)}^{\lambda /(1-\lambda )}\cdot {\left(H_{\lambda }\left(P_{A,i}∥P_{H,i}\right)\right)}^{1/(1-\lambda )},1\right\},$$

and for all λ>1, i∈I it can be bounded from below by

(24) $$E_{\varsigma }\left(P_{A,i}∥P_{H,i}\right)\;\geq \;E_{\varsigma }^{L}\left(P_{A,i}∥P_{H,i}\right)\;:=\;{\left(1-\varsigma \right)}^{\lambda /(\lambda -1)}\cdot {\left(H_{\lambda }\left(P_{A,i}∥P_{H,i}\right)\right)}^{1/(1-\lambda )}\;,$$

which is an adaption of a general result of Krafft & Plachky [125], see also Liese & Vajda [1] as well as Stummer & Vajda [15]. Hence, by combining (23) and (24) with the exact values respectively upper bounds of the Hellinger integrals $H_{1-\lambda }\left(P_{A,n}∥P_{H,n}\right)$ from the following sections, we obtain for our context of Galton-Watson processes with Poisson offspring and Poisson immigration (including the non-immigration case) some upper bounds of $E_{\varsigma }\left(P_{A,n}∥P_{H,n}\right)$, which can also be immediately rewritten as lower bounds for the power $1-E_{\varsigma }\left(P_{A,n}∥P_{H,n}\right)$ of a most powerful test at level ς. In contrast to such finite-time-horizon results, for the (to our context) incompatible setup of Galton-Watson processes with Poisson offspring but nonstochastic immigration of constant value 1, the asymptotic rates of decrease as n→∞ of the unconstrained type II error probabilities as well as the type I error probabilites were studied in Linkov & Lunyova [53] by a different approach employing also Hellinger integrals. Some other types of Galton-Watson-process concerning Neyman-Pearson testing investigations different to ours can be found e.g., in Basawa & Scott [126], Feigin [127], Sweeting [128], Basawa & Scott [61], and the references therein.

### 2.6. Asymptotical Distinguishability

The next two concepts deal with two general families ${\left(P_{A,i}\right)}_{i\in I}$ and ${\left(P_{H,i}\right)}_{i\in I}$ of probability measures on the measurable spaces ${\left(\Omega_{i},F_{i}\right)}_{i\in I}$, where the index set I is either $N_{0}$ or $R_{+}$. For them, the following two general types of asymptotical distinguishability are well known (see e.g., LeCam [109], Liese & Vajda [1], Jacod & Shiryaev [24], Linkov [129], and the references therein).

**Definition** **1.** *The family ${\left(P_{A,i}\right)}_{i\in I}$ is contiguous to the family ${\left(P_{H,i}\right)}_{i\in I}$ – in symbols, $\left(P_{A,i}\right)\;◃\;\left(P_{H,i}\right)$– if for all sets $A_{i}\in F_{i}$ with $\lim_{i\to \infty }P_{H,i}\left(A_{i}\right)=0$ there holds $\lim_{i\to \infty }P_{A,i}\left(A_{i}\right)=0$.*

**Definition** **2.** *Families of measures ${\left(P_{A,i}\right)}_{i\in I}$ and ${\left(P_{H,i}\right)}_{i\in I}$ are called entirely separated (completely asymptotically distinguishable)—in symbols, $\left(P_{A,i}\right)△\left(P_{H,i}\right)$–if there exist a sequence $i_{m}↑\infty$ as m↑∞ and for each $m\in N_{0}$ an $A_{i_{m}}\in F_{i_{m}}$ such that $\lim_{m\to \infty }P_{A,i_{m}}\left(A_{i_{m}}\right)=1$ and $\lim_{m\to \infty }P_{H,i_{m}}\left(A_{i_{m}}\right)=0$.*

It is clear that the notion of contiguity is the attempt to carry the concept of absolute continuity over to families of measures. Loosely speaking, $\left(P_{A,i}\right)$ is contiguous to $\left(P_{H,i}\right)$, if the limit $\lim_{i\to \infty }\left(P_{A,i}\right)$ (existence preconditioned) is absolute continuous to the limit $\lim_{i\to \infty }\left(P_{H,i}\right)$. However, for the definition of contiguity, we do not need to require the probability measures to converge to limiting probability measures. On the other hand, entire separation is the generalization of singularity to families of measures.

The corresponding negations will be denoted by $\bar{\;◃}$ and $\bar{\;△}$. One can easily check that a family $\left(P_{A,i}\right)$ cannot be both contiguous and entirely separated to a family $\left(P_{H,i}\right)$. In fact, as shown in Linkov [129], the relation between the families $\left(P_{A,i}\right)$ and $\left(P_{H,i}\right)$ can be uniquely classified into the following *distinguishability types*:

(a) $\left(P_{A,i}\right)\;◃▹\;\left(P_{H,i}\right)$;
(b) $\left(P_{A,i}\right)\;◃\;\left(P_{H,i}\right)$, $\left(P_{H,i}\right)\;\bar{\;◃}\;\left(P_{A,i}\right)$;
(c) $\left(P_{A,i}\right)\;\bar{\;◃}\;\left(P_{H,i}\right)$, $\left(P_{H,i}\right)\;◃\;\left(P_{A,i}\right)$;
(d) $\left(P_{A,i}\right)\;\bar{\;◃}\;\bar{\;▹}\;\left(P_{H,i}\right)$, $\left(P_{A,i}\right)\;\bar{\;△}\;\left(P_{H,i}\right)$;
(e) $\left(P_{A,i}\right)\;△\;\left(P_{H,i}\right)$.

As demonstrated in the above-mentioned references for a general context, one can conclude the type of distinguishability from the time-evolution of Hellinger integrals. Indeed, the following assertions can be found e.g., in Linkov [129], where part (c) was established in Liese & Vajda [1] and (f), (g) in Vajda [3].

**Proposition** **1.** *The following assertions are equivalent:*

(25) $$\begin{array}{ccc} \; & \left(a\right) & \left(P_{A,i}\right)\;△\;\left(P_{H,i}\right)\;,\\ \; & \left(b\right) & \underset{i\to \infty }{lim inf}H_{\lambda }(P_{A,i}∥P_{H,i})\;=\;0\;\;\mathrm{for}\;\mathrm{all}\;\lambda \in ]0,1[,\\ \; & \left(c\right) & \mathrm{there}\;\mathrm{exists}\;a\;\lambda \in \;]0,1[\;:\;\underset{i\to \infty }{lim inf}H_{\lambda }(P_{A,i}∥P_{H,i})\;=\;0\;,\\ \; & \left(d\right) & \mathrm{there}\;\mathrm{exists}\;a\;\pi \in \;]0,1[\;:\;\underset{i\to \infty }{lim inf}e_{\pi }(P_{A,i}∥P_{H,i})\;=\;0\;,\\ \; & \left(e\right) & \underset{i\to \infty }{lim sup}V(P_{A,i}∥P_{H,i})\;=\;2\;,\\ \; & \left(f\right) & \mathrm{there}\;\mathrm{exists}\;a\;\lambda \in \;]0,1[\;:\;\underset{i\to \infty }{lim sup}I_{\lambda }(P_{A,i}∥P_{H,i})\;=\;\frac{1}{\lambda \cdot (1-\lambda )}\;,\\ \; & \left(g\right) & \underset{i\to \infty }{lim sup}I_{\lambda }(P_{A,i}∥P_{H,i})\;=\;\frac{1}{\lambda \cdot (1-\lambda )}\;,\;\mathrm{for}\;\mathrm{all}\;\lambda \in ]0,1[. \end{array}$$

In combination with the discussion after Definition 2, one can thus interpret the λ–order Hellinger integral $H_{\lambda }(P_{A,i}∥P_{H,i})$ as a “measure” for the distinctness of the two families $P_{A,i}$ and $P_{H,i}$ up to a fixed finite time horizon i∈I.

Furthermore, for the contiguity we obtain the equivalence (see e.g., Liese & Vajda [1], Linkov [129])

(26) $$\begin{array}{ccc} \left(P_{A,i}\right)\;◃\;\left(P_{H,i}\right)\; & ⟺ & \;\underset{\lambda ↗1}{lim inf}\left\{\underset{i\to \infty }{lim inf}\;H_{\lambda }\left(P_{A,i}∥P_{H,i}\right)\right\}\;=\;1\;\\ & ⟺ & \;\underset{\lambda ↗1}{lim sup}\left\{\underset{i\to \infty }{lim sup}\;\lambda \cdot \left(1-\lambda \right)\cdot I_{\lambda }\left(P_{A,i}∥P_{H,i}\right)\right\}\;=\;0. \end{array}$$

All the above-mentioned general results can be applied to our context of two competing Poissonian Galton-Watson processes with immigration (GWI) (H) and (A) (reflected by the two different laws $P_{H}$ resp. $P_{A}$ with parameter pairs $\left(\beta_{H},\alpha_{H}\right)$ resp. $\left(\beta_{A},\alpha_{A}\right)$), by taking $P_{A,i}:={\left.P_{A}\right|}_{F_{i}}$ and $P_{H,i}:={\left.P_{H}\right|}_{F_{i}}$. Recall from the preceding subsections (by identifying *i* with *n*) that the latter two describe the stochastic dynamics of the respective GWI within the restricted time-/stage-frame {0,1,…,i}.

In the following, we study in detail the evolution of Hellinger integrals between two competing models of Galton-Watson processes with immigration, which turns out to be quite extensive.

## 3. Detailed Recursive Analyses of Hellinger Integrals

### 3.1. A First Basic Result

In terms of our notations (PS1) to (PS3), a typical situation for applications in our mind is that one particular constellation $\left(\beta_{A},\beta_{H},\alpha_{A},\alpha_{H}\right)\in P$ (e.g., obtained from theoretical or previous statistical investigations) is fixed, whereas–in contrast–the parameter λ∈R\{0,1} for the Hellinger integral or the power divergence might be chosen freely, e.g., depending on which (transform of a) dissimilarity measure one decides to choose for further analysis. At this point, let us emphasize that *in general* we will not make assumptions of the form $\beta_{•}⪌1$, i.e., upon the type of criticality.

To start with our investigations, in order to justify for all $n\in N_{0}$

$$Z_{n}\;:=\;\frac{dP_{A,n}}{dP_{H,n}}\;\;\left(\mathrm{cf}.\;(13)\right),$$

(14) and (15) (as well as $I_{\lambda }\left(P_{A,n}∥P_{H,n}\right)$ for λ∈R respectively $R_{\lambda }\left(P_{A,n}∥P_{H,n}\right)$ for λ∈R\{0,1}), we first mention the following straightforward facts: (i) if $\left(\beta_{A},\beta_{H},\alpha_{A},\alpha_{H}\right)\in P_{\mathrm{NI}}$, then $P_{A,n}$ and $P_{H,n}$ are equivalent (i.e., $P_{A,n}∼P_{H,n}$), as well as (ii) if $\left(\beta_{A},\beta_{H},\alpha_{A},\alpha_{H}\right)\in P_{\mathrm{SP}}$, then $P_{A,n}$ and $P_{H,n}$ are equivalent (i.e., $P_{A,n}∼P_{H,n}$). Moreover, by recalling $Z_{0}=1$ and using the “rate functions” $f_{•}\left(x\right)=\beta_{•}\;x+\alpha_{•}$ (x∈[0,∞[), a version of (13) can be easily determined by calculating for each $\vec{x}:=\left(x_{0},x_{1},x_{2},\cdots \right)\in \Omega :=N\times N_{0}\times N_{0}\times \cdots$

$$\begin{array}{cc} Z_{n}\left(\vec{x}\right)=\prod_{k=1}^{n}Z_{n,k}\left(\vec{x}\right) & \mathrm{with}\;Z_{n,k}\left(\vec{x}\right):=\exp\left\{-\left(f_{A}\left(x_{k-1}\right)-f_{H}\left(x_{k-1}\right)\right)\right\}{\left[\frac{f_{A}\left(x_{k-1}\right)}{f_{H}\left(x_{k-1}\right)}\right]}^{x_{k}}, \end{array}$$

where for the last term we use the convention ${\left(\frac{0}{0}\right)}^{x}=1$ for all $x\in N_{0}$. Furthermore, we define for each $\vec{x}\in \Omega$

(27) $$Z_{n,k}^{\left(\lambda \right)}\left(\vec{x}\right):=\exp\left\{-\left(\lambda f_{A}\left(x_{k-1}\right)+\left(1-\lambda \right)f_{H}\left(x_{k-1}\right)\right)\right\}\;\frac{{\left[{\left(f_{A}\left(x_{k-1}\right)\right)}^{\lambda }{\left(f_{H}\left(x_{k-1}\right)\right)}^{1-\lambda }\right]}^{x_{k}}}{x_{k}!}$$

with the convention $\frac{{\left(0\right)}^{0}}{0!}=1$ for the last term. Accordingly, one obtains from (14) the Hellinger integral $H_{\lambda }\left(P_{A,0}∥P_{H,0}\right)=1$, as well as for all $\left(\beta_{A},\beta_{H},\alpha_{A},\alpha_{H},\lambda \right)\in P\times \left(R\backslash \left\{0,1\right\}\right)$

(28) $$H_{\lambda }\left(P_{A,1}∥P_{H,1}\right)=\exp\left\{{\left(f_{A}\left(x_{0}\right)\right)}^{\lambda }{\left(f_{H}\left(x_{0}\right)\right)}^{\left(1-\lambda \right)}-\left(\lambda f_{A}\left(x_{0}\right)+\left(1-\lambda \right)f_{H}\left(x_{0}\right)\right)\right\}$$

for $x_{0}=X_{0}\in N$, and for all n∈N\{1}

(29) $$\begin{array}{c} \;H_{\lambda }\left(P_{A,n}∥P_{H,n}\right)\;=\;EP_{H,n}\left[{\left(Z_{n}\right)}^{\lambda }\right]\;=\;\sum_{x_{1}=0}^{\infty }\cdots \sum_{x_{n}=0}^{\infty }\prod_{k=1}^{n}Z_{n,k}^{\left(\lambda \right)}\left(\vec{x}\right)\\ \;=\;\sum_{x_{1}=0}^{\infty }\cdots \sum_{x_{n-1}=0}^{\infty }\prod_{k=1}^{n-1}Z_{n,k}^{\left(\lambda \right)}\left(\vec{x}\right)\cdot e^{-(\lambda f_{A}\left(x_{n-1}\right)+\left(1-\lambda \right)f_{H}\left(x_{n-1}\right))}\sum_{x_{n}=0}^{\infty }\frac{{\left[{\left(f_{A}\left(x_{n-1}\right)\right)}^{\lambda }{\left(f_{H}\left(x_{n-1}\right)\right)}^{1-\lambda }\right]}^{x_{n}}}{x_{n}!}\\ \;=\;\sum_{x_{1}=0}^{\infty }\cdots \sum_{x_{n-1}=0}^{\infty }\prod_{k=1}^{n-1}Z_{n,k}^{\left(\lambda \right)}\left(\vec{x}\right)\cdot \exp\left\{{\left(f_{A}\left(x_{n-1}\right)\right)}^{\lambda }{\left(f_{H}\left(x_{n-1}\right)\right)}^{1-\lambda }-\left(\lambda f_{A}\left(x_{n-1}\right)+\left(1-\lambda \right)f_{H}\left(x_{n-1}\right)\right)\right\}.\;\; \end{array}$$

From (29), one can see that a crucial role for the exact calculation (respectively the derivation of bounds) of the Hellinger integral is played by the functions defined for x∈[0,∞[

(30) $$\phi_{\lambda }\left(x\right)\;:=\;\phi \left(x,\beta_{A},\beta_{H},\alpha_{A},\alpha_{H},\lambda \right)\;:=\;\varphi_{\lambda }\left(x\right)-f_{\lambda }\left(x\right)\;,\;\mathrm{with}$$

(31) $$\varphi_{\lambda }\left(x\right)\;:=\;\varphi \left(x,\beta_{A},\beta_{H},\alpha_{A},\alpha_{H},\lambda \right)\;:=\;{\left(f_{A}\left(x\right)\right)}^{\lambda }{\left(f_{H}\left(x\right)\right)}^{1-\lambda }\;\mathrm{and}$$

(32) $$f_{\lambda }\left(x\right)\;:=\;f\left(x,\beta_{A},\beta_{H},\alpha_{A},\alpha_{H},\lambda \right)\;:=\;\lambda f_{A}\left(x\right)+\left(1-\lambda \right)f_{H}\left(x\right)\;=\;\alpha_{\lambda }+\beta_{\lambda }\;x\;,$$

where we have used the λ-*weighted-averages*

$$\alpha_{\lambda }:=\alpha \left(\alpha_{A},\alpha_{H},\lambda \right):=\lambda \cdot \alpha_{A}+\left(1-\lambda \right)\cdot \alpha_{H}\;\mathrm{and}\;\beta_{\lambda }:=\beta \left(\beta_{A},\beta_{H},\lambda \right):=\lambda \cdot \beta_{A}+\left(1-\lambda \right)\cdot \beta_{H}.$$

Since λ plays a special role, henceforth we typically use it as index and often omit $\left(\beta_{A},\beta_{H},\alpha_{A},\alpha_{H}\right)$. According to Lemma A1 in the Appendix A.1, it follows that for λ∈]0,1[ (respectively λ∈R\[0,1]) one gets $\phi_{\lambda }\left(x\right)\leq 0$ (respectively $\phi_{\lambda }\left(x\right)\geq 0$) for all x∈[0,∞[. Furthermore, in both cases there holds $\phi_{\lambda }\left(x\right)=0$ iff $f_{A}\left(x\right)=f_{H}\left(x\right)$, i.e., for $x=x^{*}:=\frac{\alpha_{A}-\alpha_{H}}{\beta_{H}-\beta_{A}}\geq 0$. This is consistent with the corresponding generally valid upper and lower bounds (cf. (9) and (11)) $0\;<\;H_{\lambda }\left(P_{A,n}∥P_{H,n}\right)\;\leq 1\;,\;\mathrm{for}\;\lambda \in ]0,1[\;,\;1\;\leq \;H_{\lambda }\left(P_{A,n}∥P_{H,n}\right)\;\leq \;\infty \;,\;\mathrm{for}\;\lambda \in R\backslash \left[0,1\right]\;$.

As a first indication for our proposed method, let us start by illuminating the simplest case λ∈R\{0,1} and $\gamma :=\alpha_{H}\beta_{A}-\alpha_{A}\beta_{H}=0$. This means that $\left(\beta_{A},\beta_{H},\alpha_{A},\alpha_{H}\right)\in P_{\mathrm{NI}}\cup P_{\mathrm{SP},1}$, where $P_{\mathrm{SP},1}$ is the set of all (componentwise) strictly positive $\left(\beta_{A},\beta_{H},\alpha_{A},\alpha_{H}\right)$ with $\beta_{A}\neq \beta_{H}$, $\alpha_{A}\neq \alpha_{H}$ and $\frac{\beta_{A}}{\beta_{H}}=\frac{\alpha_{A}}{\alpha_{H}}\neq 1$ (“the equal-fraction-case”). In this situation, *all* the three functions (30) to (32) are linear. Indeed,

(33) $$\varphi_{\lambda }\left(x\right)\;=\;p_{\lambda }^{E}+q_{\lambda }^{E}\;x$$

with $p_{\lambda }^{E}:=\alpha_{A}^{\lambda }\;\alpha_{H}^{1-\lambda }$ and $q_{\lambda }^{E}:=\beta_{A}^{\lambda }\;\beta_{H}^{1-\lambda }$ (where the index E stands for exact linearity). Clearly, $q_{\lambda }^{E}>0$ on $P_{\mathrm{NI}}\cup P_{\mathrm{SP},1}$, as well as $p_{\lambda }^{E}>0$ on $P_{\mathrm{SP},1}$ and $p_{\lambda }^{E}=0$ on $P_{\mathrm{NI}}$. Furthermore,

$$\phi_{\lambda }\left(x\right)\;=\;r_{\lambda }^{E}+s_{\lambda }^{E}\;x$$

with $r_{\lambda }^{E}:=p_{\lambda }^{E}-\alpha_{\lambda }=\alpha_{A}^{\lambda }\;\alpha_{H}^{1-\lambda }-\left(\lambda \alpha_{A}+\left(1-\lambda \right)\alpha_{H}\right)$ and $s_{\lambda }^{E}:=q_{\lambda }^{E}-\beta_{\lambda }=\beta_{A}^{\lambda }\;\beta_{H}^{1-\lambda }-\left(\lambda \beta_{A}+\left(1-\lambda \right)\beta_{H}\right)$. Due to Lemma A1 one knows that on $P_{\mathrm{NI}}\cup P_{\mathrm{SP},1}$ one gets $s_{\lambda }^{E}<0$ for λ∈]0,1[ and $s_{\lambda }^{E}>0$ for λ∈R\[0,1]. Furthermore, on $P_{\mathrm{SP},1}$ one gets $r_{\lambda }^{E}<0$ (resp. $r_{\lambda }^{E}>0$) for λ∈]0,1[ (resp. λ∈R\[0,1]), whereas on $P_{\mathrm{NI}}$, the no-immigration setup, we get for all λ∈R\{0,1} $r_{\lambda }^{E}=0$.

As it will be seen later on, such kind of linearity properties are useful for the recursive handling of the Hellinger integrals. However, only on the parameter set $P_{\mathrm{NI}}\cup P_{\mathrm{SP},1}$ the functions $\varphi_{\lambda }$ and $\phi_{\lambda }$ are linear. Hence, in the general case $\left(\beta_{A},\beta_{H},\alpha_{A},\alpha_{H},\lambda \right)\in P\times R\backslash \left\{0,1\right\}$ we aim for linear lower and upper bounds

(34) $$\varphi_{\lambda }^{L}\left(x\right):=p_{\lambda }^{L}+q_{\lambda }^{L}\;x\;\leq \;\varphi_{\lambda }\left(x\right)\;\leq \;\varphi_{\lambda }^{U}\left(x\right):=p_{\lambda }^{U}+q_{\lambda }^{U}\;x\;,$$

x∈[0,∞[ (ultimately, $x\in N_{0}$), which by (30) and (31) leads to

(35) $$\phi_{\lambda }\left(x\right)\;\left\{\begin{array}{cc} \leq & \phi_{\lambda }^{U}\left(x\right)\;:=\;r_{\lambda }^{U}\;+\;s_{\lambda }^{U}\cdot x\;:=\;\left(p_{\lambda }^{U}-\alpha_{\lambda }\right)\;+\;\left(q_{\lambda }^{U}-\beta_{\lambda }\right)\cdot x\;,\\ \; & \;\\ \geq & \phi_{\lambda }^{L}\left(x\right)\;:=\;r_{\lambda }^{L}\;+\;s_{\lambda }^{L}\cdot x\;:=\;\left(p_{\lambda }^{L}-\alpha_{\lambda }\right)\;+\;\left(q_{\lambda }^{L}-\beta_{\lambda }\right)\cdot x\;, \end{array}\right.$$

x∈[0,∞[ (ultimately, $x\in N_{0}$). Of course, the involved slopes and intercepts should satisfy reasonable restrictions. Later on, we shall impose further restrictions on the involved slopes and intercepts, in order to guarantee nice properties of the general Hellinger integral bounds given in Theorem 1 below (for instance, in consistency with the nonnegativity of $\varphi_{\lambda }$ we could require $p_{\lambda }^{U}\geq p_{\lambda }^{L}\geq 0$, $q_{\lambda }^{U}\geq q_{\lambda }^{L}\geq 0$ which nontrivially implies that these bounds possess certain monotonicity properties). For the formulation of our first assertions on Hellinger integrals, we make use of the following notation:

**Definition** **3.** *For all $\left(\beta_{A},\beta_{H},\alpha_{A},\alpha_{H},\lambda \right)\in P\times R\backslash \left\{0,1\right\}$ and all p,q∈R let us define the sequences ${\left(a_{n}^{\left(q\right)}\right)}_{n\in N_{0}}$ and ${\left(b_{n}^{\left(p,q\right)}\right)}_{n\in N_{0}}$ recursively by*

(36) $$\begin{array}{ccc} a_{0}^{\left(q\right)}:=0 & ; & \;a_{n}^{\left(q\right)}:=\;\xi_{\lambda }^{\left(q\right)}\left(a_{n-1}^{\left(q\right)}\right)\;\;:=\;q\cdot e^{a_{n-1}^{\left(q\right)}}-\beta_{\lambda },\;\;n\in N, \end{array}$$

(37) $$\begin{array}{ccc} b_{0}^{\left(p,q\right)}:=0 & ; & \;b_{n}^{\left(p,q\right)}\;:=\;p\cdot e^{a_{n-1}^{\left(q\right)}}-\alpha_{\lambda },\;\;n\in N. \end{array}$$

Notice the interrelation $a_{1}^{\left(q_{\lambda }^{A}\right)}=s_{\lambda }^{A}$ and $b_{1}^{\left(p_{\lambda }^{A},q_{\lambda }^{A}\right)}=r_{\lambda }^{A}$ for A∈{E,L,U}. Clearly, for all q∈R\{0} and p∈R one has the linear interrelation

(38) $$b_{n}^{\left(p,q\right)}=\frac{p}{q}\;a_{n}^{\left(q\right)}\;+\;\frac{p}{q}\;\beta_{\lambda }\;-\;\alpha_{\lambda },\;\;n\in N.$$

Accordingly, we obtain fundamental Hellinger integral evaluations:

**Theorem** **1.**

*(a)*  *For all $\left(\beta_{A},\beta_{H},\alpha_{A},\alpha_{H},\lambda \right)\in \left(P_{\mathrm{NI}}\cup P_{\mathrm{SP},1}\right)\times R\backslash \left\{0,1\right\}$, all initial population sizes $X_{0}\in N$ and all observation horizons n∈N one can recursively compute the **exact value***
  (39) $$H_{\lambda }(P_{A,n}∥P_{H,n})\;=\;\exp\left\{a_{n}^{\left(q_{\lambda }^{E}\right)}\;X_{0}\;+\;\frac{\alpha_{A}}{\beta_{A}}\;\sum_{k=1}^{n}a_{k}^{\left(q_{\lambda }^{E}\right)}\right\}\;=:\;V_{\lambda ,X_{0},n},$$
  *where $\frac{\alpha_{A}}{\beta_{A}}$ can be equivalently replaced by $\frac{\alpha_{H}}{\beta_{H}}$. Recall that $q_{\lambda }^{E}:=\beta_{A}^{\lambda }\;\beta_{H}^{1-\lambda }$. Notice that on $P_{\mathrm{NI}}\times \left(R\backslash \left\{0,1\right\}\right)$ the formula (39) simplifies significantly, since $\alpha_{A}=\alpha_{H}=0$.*
*(b)*  *For all $\left(\beta_{A},\beta_{H},\alpha_{A},\alpha_{H},\lambda \right)\in \left(P_{\mathrm{SP}}\backslash P_{\mathrm{SP},1}\right)\times \left(R\backslash \left\{0,1\right\}\right)$, all coefficients $p_{\lambda }^{L},\;p_{\lambda }^{U},\;q_{\lambda }^{L},\;q_{\lambda }^{U}\in R$ which satisfy (35) for all $x\in N_{0}$(and thus in particular $p_{\lambda }^{L}\leq p_{\lambda }^{U},\;q_{\lambda }^{L}\leq q_{\lambda }^{U}$), all initial population sizes $X_{0}\in N$ and all observation horizons n∈N one gets the following **recursive** (i.e., recursively computable) **bounds** for the Hellinger integrals:*
  (40) $$\mathrm{for}\;\lambda \in ]0,1[\;:\;B_{\lambda ,X_{0},n}^{L}\;:=\;{\tilde{B}}_{\lambda ,X_{0},n}^{\left(p_{\lambda }^{L},q_{\lambda }^{L}\right)}\;<\;H_{\lambda }(P_{A,n}∥P_{H,n})\;\leq \;\min\left\{{\tilde{B}}_{\lambda ,X_{0},n}^{\left(p_{\lambda }^{U},q_{\lambda }^{U}\right)}\;,\;1\right\}\;=:\;B_{\lambda ,X_{0},n}^{U}\;,$$
  (41) $$\mathrm{for}\;\lambda \in R\backslash \left[0,1\right]\;:\;B_{\lambda ,X_{0},n}^{L}\;:=\;\max\left\{{\tilde{B}}_{\lambda ,X_{0},n}^{\left(p_{\lambda }^{L},q_{\lambda }^{L}\right)}\;,\;1\right\}\;\leq \;H_{\lambda }(P_{A,n}∥P_{H,n})\;<\;{\tilde{B}}_{\lambda ,X_{0},n}^{\left(p_{\lambda }^{U},q_{\lambda }^{U}\right)}\;=:\;B_{\lambda ,X_{0},n}^{U}\;,$$
  *where for general λ∈R\{0,1}, p∈R,q∈R\{0} we use the definitions*
  (42) $${\tilde{B}}_{\lambda ,X_{0},n}^{\left(p,q\right)}\;:=\;\exp\left\{a_{n}^{\left(q\right)}\cdot X_{0}\;+\;\sum_{k=1}^{n}b_{k}^{\left(p,q\right)}\right\}\;=\;\exp\left\{a_{n}^{\left(q\right)}\cdot X_{0}\;+\;\frac{p}{q}\sum_{k=1}^{n}a_{k}^{\left(q\right)}\;+\;n\cdot \left(\frac{p}{q}\beta_{\lambda }-\alpha_{\lambda }\right)\right\},\;$$
  *as well as*
  $${\tilde{B}}_{\lambda ,X_{0},n}^{\left(p,0\right)}\;:=\;\exp\left\{-\beta_{\lambda }\cdot X_{0}\;+\;\left(p\cdot e^{-\beta_{\lambda }}-\alpha_{\lambda }\right)\cdot n\right\}\;.$$

**Remark** **1.**

*(a)*  *Notice that the expression ${\tilde{B}}_{\lambda ,X_{0},n}^{\left(p,q\right)}$ can analogously be defined on the parameter set $P_{\mathrm{NI}}\cup P_{\mathrm{SP},1}$. For the choices $q_{\lambda }^{E}:=\beta_{A}^{\lambda }\beta_{H}^{1-\lambda }>0$ and $p_{\lambda }^{E}:=\alpha_{A}^{\lambda }\alpha_{H}^{1-\lambda }=q_{\lambda }^{E}\cdot \frac{\alpha_{A}}{\beta_{A}}=q_{\lambda }^{E}\cdot \frac{\alpha_{H}}{\beta_{H}}\geq 0$ one gets $\left(p_{\lambda }^{E}/q_{\lambda }^{E}\right)\cdot \beta_{\lambda }-\alpha_{\lambda }=0$, and thus the characterization ${\tilde{B}}_{\lambda ,X_{0},n}^{\left(p_{\lambda }^{E},q_{\lambda }^{E}\right)}=V_{\lambda ,X_{0},n}$ as the exact value (rather than a lower/upper bound (component)).*
*(b)*  *In the case $q=\beta_{\lambda }$ one gets the explicit representation ${\tilde{B}}_{\lambda ,X_{0},n}^{\left(p,q\right)}=\exp\left\{\left(p-\alpha_{\lambda }\right)\cdot n\right\}$.*
*(c)*  *Using the skew symmetry (8), one can derive alternative bounds of the Hellinger integral by switching to the transformed parameter setup $\left(\overset{↔}{\beta_{A}},\overset{↔}{\beta_{H}},\overset{↔}{\alpha_{A}},\overset{↔}{\alpha_{H}},\overset{↔}{\lambda }\right):=\left(\beta_{H},\beta_{A},\alpha_{H},\alpha_{A},1-\lambda \right)$. However, this does not lead to different bounds: define ${\overset{↔}{\phi }}_{\overset{↔}{\lambda }}$, ${\overset{↔}{\varphi }}_{\overset{↔}{\lambda }}$ and ${\overset{↔}{f}}_{\overset{↔}{\lambda }}$ analogously to (30), (31) and (32) by replacing the parameters $\left(\beta_{A},\beta_{H},\alpha_{A},\alpha_{H},\lambda \right)$ with $\left(\overset{↔}{\beta_{A}},\overset{↔}{\beta_{H}},\overset{↔}{\alpha_{A}},\overset{↔}{\alpha_{H}},\overset{↔}{\lambda }\right)$. Then, there holds ${\overset{↔}{f}}_{\overset{↔}{\lambda }}\left(x\right)=f_{\lambda }\left(x\right),\;{\overset{↔}{\varphi }}_{\overset{↔}{\lambda }}\left(x\right)=\varphi_{\lambda }\left(x\right)$ and ${\overset{↔}{\phi }}_{\overset{↔}{\lambda }}\left(x\right)=\phi_{\lambda }\left(x\right)$, and the set of (lower and upper bound) parameters $p_{\lambda }^{L},q_{\lambda }^{L},p_{\lambda }^{U},q_{\lambda }^{U}$ satisfying (35) does not change under this transformation.*
*(d)*  *If there are no other restrictions on $p_{\lambda }^{L},\;p_{\lambda }^{U},\;q_{\lambda }^{L},\;q_{\lambda }^{U}$ than (35), the bounds in (40) and (41) can have some inconvenient features, e.g., being 1 for all (large enough) n∈N, having oscillating n-behaviour, being suboptimal in certain (other) senses. For a detailed discussion, the reader is referred to Section 3.16 ff. below.*
*(e)*  *For the (to our context) incompatible setup of GWI with Poisson offspring but nonstochastic immigration of constant value 1, the exact values of the corresponding Hellinger integrals (i.e., an “analogue” of part (a)) was established in Linkov & Lunyova [53].*

**Proof of Theorem** **1.** Let us fix $\left(\beta_{A},\beta_{H},\alpha_{A},\alpha_{H}\right)\in P$ as well as $x_{0}:=X_{0}\in N$, and start with arbitrary λ∈]0,1[. We first prove the upper bound $B_{\lambda ,X_{0},n}^{U}$ of part (b). Correspondingly, we suppose that the coefficients $p_{\lambda }^{U}$, $q_{\lambda }^{U}$ satisfy (35) for all $x\in N_{0}$. From (28), (30), (31), (32) and (35) one gets immediately $B_{\lambda ,X_{0},1}^{U}$ in terms of the first sequence-element $a_{1}^{\left(q_{\lambda }^{U}\right)}$ (cf. (36)). With the help of (29) for all observation horizons n∈N\{1} we get (with the obvious shortcut for n=2)

(43) $$\begin{array}{c} H_{\lambda }\left(P_{A,n}∥P_{H,n}\right)\;=\;\sum_{x_{1}=0}^{\infty }\cdots \sum_{x_{n-1}=0}^{\infty }\;\prod_{k=1}^{n-1}Z_{n,k}^{\left(\lambda \right)}\left(\vec{x}\right)\cdot \exp\left\{\varphi_{\lambda }\left(x_{n-1}\right)-f_{\lambda }\left(x_{n-1}\right)\right\}\\ <\;\sum_{x_{1}=0}^{\infty }\cdots \sum_{x_{n-1}=0}^{\infty }\;\prod_{k=1}^{n-1}Z_{n,k}^{\left(\lambda \right)}\left(\vec{x}\right)\cdot \exp\left\{\left(p_{\lambda }^{U}-\alpha_{\lambda }\right)+\left(q_{\lambda }^{U}-\beta_{\lambda }\right)\;x_{n-1}\right\}\\ =\;\sum_{x_{1}=0}^{\infty }\cdots \sum_{x_{n-1}=0}^{\infty }\;\prod_{k=1}^{n-1}Z_{n,k}^{\left(\lambda \right)}\left(\vec{x}\right)\cdot \exp\left\{b_{1}^{\left(p_{\lambda }^{U},q_{\lambda }^{U}\right)}+a_{1}^{\left(q_{\lambda }^{U}\right)}\;x_{n-1}\right\}\\ =\;\exp\left\{b_{1}^{\left(p_{\lambda }^{U},q_{\lambda }^{U}\right)}\right\}\;\sum_{x_{1}=0}^{\infty }\cdots \sum_{x_{n-2}=0}^{\infty }\;\prod_{k=1}^{n-2}Z_{n,k}^{\left(\lambda \right)}\left(\vec{x}\right)\cdot \exp\left\{\exp\left\{a_{1}^{\left(q_{\lambda }^{U}\right)}\right\}\;\varphi_{\lambda }\left(x_{n-2}\right)-f_{\lambda }\left(x_{n-2}\right)\right\}\\ <\;\exp\left\{b_{1}^{\left(p_{\lambda }^{U},q_{\lambda }^{U}\right)}\right\}\;\sum_{x_{1}=0}^{\infty }\cdots \sum_{x_{n-2}=0}^{\infty }\;\prod_{k=1}^{n-2}Z_{n,k}^{\left(\lambda \right)}\left(\vec{x}\right)\\ \cdot \exp\left\{\left(\exp\left\{a_{1}^{\left(q_{\lambda }^{U}\right)}\right\}\;p_{\lambda }^{U}-\alpha_{\lambda }\right)+\left(\exp\left\{a_{1}^{\left(q_{\lambda }^{U}\right)}\right\}\;q_{\lambda }^{U}-\beta_{\lambda }\right)\cdot x_{n-2}\right\}\\ <\;\exp\left\{b_{1}^{\left(p_{\lambda }^{U},q_{\lambda }^{U}\right)}\right\}\;\sum_{x_{1}=0}^{\infty }\cdots \sum_{x_{n-2}=0}^{\infty }\;\prod_{k=1}^{n-2}Z_{n,k}^{\left(\lambda \right)}\left(\vec{x}\right)\cdot \exp\left\{b_{2}^{\left(p_{\lambda }^{U},q_{\lambda }^{U}\right)}+a_{2}^{\left(q_{\lambda }^{U}\right)}\;x_{n-2}\right\}\\ <\;\cdots \;<\;\exp\left\{a_{n}^{\left(q_{\lambda }^{U}\right)}\;x_{0}\;+\;\sum_{k=1}^{n}b_{k}^{\left(p_{\lambda }^{U},q_{\lambda }^{U}\right)}\right\}\;. \end{array}$$

Notice that for the strictness of the above inequalities we have used the fact that $\phi_{\lambda }\left(x\right)<\phi_{\lambda }^{U}\left(x\right)$ for some (in fact, all but at most two) $x\in N_{0}$ (cf. Properties 3(P19) below). Since for some admissible choices of $p_{\lambda }^{U},q_{\lambda }^{U}$ and some n∈N the last term in (43) can become larger than 1, one needs to take into account the cutoff-point 1 arising from (9). The lower bound $B_{\lambda ,X_{0},n}^{L}$ of part (b), as well as the exact value of part (a) follow from (29) in an analogous manner by employing $p_{\lambda }^{L},q_{\lambda }^{L}$ and $p_{\lambda }^{E},q_{\lambda }^{E}$ respectively. Furthermore, we use the fact that for $\left(\beta_{A},\beta_{H},\alpha_{A},\alpha_{H},\lambda \right)\in \left(P_{\mathrm{NI}}\cup P_{\mathrm{SP},1}\right)\times ]0,1[$ one gets from (38) the relation $b_{n}^{\left(p_{\lambda }^{E},q_{\lambda }^{E}\right)}=\frac{\alpha_{A}}{\beta_{A}}\;a_{n}^{\left(q_{\lambda }^{E}\right)}$. For the sake of brevity, the corresponding straightforward details are omitted here. Although we take the minimum of the upper bound derived in (43) and 1, the inequality $B_{\lambda ,X_{0},n}^{L}<B_{\lambda ,X_{0},n}^{U}$ is nevertheless valid: the reason is that for constituting a lower bound, the parameters $p_{\lambda }^{L},q_{\lambda }^{L}$ must fulfill either the conditions $[p_{\lambda }^{L}-\alpha_{\lambda }<0$ and $q_{\lambda }^{L}-\beta_{\lambda }\leq 0]$ or $[p_{\lambda }^{L}-\alpha_{\lambda }\leq 0$ and $q_{\lambda }^{L}-\beta_{\lambda }<0]$ (or both), which guarantees that $B_{\lambda ,X_{0},n}^{L}<1$. The proof for all λ∈R\[0,1] works out completely analogous, by taking into account the generally valid lower bound $H_{\lambda }(P_{A,n}∥P_{H,n})\geq 1$ (cf. (11)). □

### 3.2. Some Useful Facts for Deeper Analyses

Theorem 1(b) and Remark 1(a) indicate the crucial role of the expression ${\tilde{B}}_{\lambda ,X_{0},n}^{\left(p,q\right)}$ and that the choice of the quantities p,q depends on the underlying (e.g., fixed) offspring-immigration parameter constellation $\left(\beta_{A},\beta_{H},\alpha_{A},\alpha_{H}\right)$ as well as on the (e.g., selectable) value of λ, i.e., $p_{\lambda }^{A}=p^{A}\left(\beta_{A},\beta_{H},\alpha_{A},\alpha_{H},\lambda \right)$ and $q_{\lambda }^{A}=q^{A}\left(\beta_{A},\beta_{H},\alpha_{A},\alpha_{H},\lambda \right)$ with A∈{E,L,U}. In order to study the desired time-behaviour $n\mapsto {\tilde{B}}_{\lambda ,X_{0},n}^{\left(\cdot ,\cdot \right)}$ of the Hellinger integral bounds resp. exact values, one therefore faces a six-dimensional (and thus highly non-obvious) detailed analysis, including the search for criteria (in addition to (35)) on good/optimal choices of $p_{\lambda }^{L},q_{\lambda }^{L},p_{\lambda }^{U},q_{\lambda }^{U}$. Since these criteria will (almost) always imply the nonnegativity of $p_{\lambda }^{A},\;q_{\lambda }^{A}$ (A∈{L,U}) and $p_{\lambda }^{E}\geq 0,\;q_{\lambda }^{E}>0$ (cf. Remark 1(a)), let us first present some fundamental properties of the underlying crucial sequences ${\left(a_{n}^{\left(q\right)}\right)}_{n\in N}$ and ${\left(b_{n}^{\left(p,q\right)}\right)}_{n\in N}$ for *general* p≥0,q≥0.

**Properties** **1.** *For all λ∈R the following holds:*

*(P1)*  *If $0<q<\beta_{\lambda }$, then the sequence ${\left(a_{n}^{\left(q\right)}\right)}_{n\in N}$ is strictly negative, strictly decreasing and converges to the unique negative solution $x_{0}^{\left(q\right)}\in ]-\beta_{\lambda },q-\beta_{\lambda }[$ of the equation*
  (44) $$\xi_{\lambda }^{\left(q\right)}\left(x\right)\;=\;q\cdot e^{x}-\beta_{\lambda }\;=\;x\;.$$
*(P2)*  *If $0<q=\beta_{\lambda }$, then $a_{n}^{\left(q\right)}\equiv 0$.*
*(P3)*  *If $q>\max\{0,\beta_{\lambda }\}$, then the sequence ${\left(a_{n}^{\left(q\right)}\right)}_{n\in N}$ is strictly positive and strictly increasing. Notice that in this setup, q=1 implies $\min\left\{1,e^{\beta_{\lambda }-1}\right\}=e^{\beta_{\lambda }-1}<q$.*
  *(P3a)*  *If additionally $q\leq \min\left\{1\;,\;e^{\beta_{\lambda }-1}\right\}$, then the sequence ${\left(a_{n}^{\left(q\right)}\right)}_{n\in N}$ converges to the smallest positive solution $x_{0}^{\left(q\right)}\in ]0,-\log q]$ of the Equation (44).*
  *(P3b)*  *If additionally $q>\min\left\{1\;,\;e^{\beta_{\lambda }-1}\right\}$, then the sequence ${\left(a_{n}^{\left(q\right)}\right)}_{n\in N}$ diverges to ∞, faster than exponentially (i.e., there do not exist constants $c_{1},c_{2}\in R$ such that $a_{n}^{\left(q\right)}\leq e^{c_{1}+c_{2}n}$ for all n∈N).*
*(P4)*  *If q=0, then one gets $a_{n}^{\left(0\right)}\equiv -\beta_{\lambda }$.*
*Due to the linear interrelation (38), these results directly carry over to the behaviour of the sequence ${\left(b_{n}^{\left(p,q\right)}\right)}_{n\in N}$:*
*(P5)*  *If p>0 and $0<q<\beta_{\lambda }$, then the sequence ${\left(b_{n}^{\left(p,q\right)}\right)}_{n\in N}$ is strictly decreasing and converges to $p\cdot e^{x_{0}^{\left(q\right)}}-\alpha_{\lambda }$. Trivially, $b_{1}^{\left(p,q\right)}=p-\alpha_{\lambda }$.*
  *(P5a)*  *If additionally $p<\alpha_{\lambda }$, then ${\left(b_{n}^{\left(p,q\right)}\right)}_{n\in N}$ is strictly negative for all n∈N.*
  *(P5b)*  *If additionally $p=\alpha_{\lambda }$, then ${\left(b_{n}^{\left(p,q\right)}\right)}_{n\in N}$ is strictly negative for all n∈N\{1}.*
  *(P5c)*  *If additionally $p>\alpha_{\lambda }$, then ${\left(b_{n}^{\left(p,q\right)}\right)}_{n\in N}$ is strictly positive for some (and possibly for all) n∈N.*
*(P6)*  *If $0<q=\beta_{\lambda }$, then $b_{n}^{\left(p,q\right)}\equiv p-\alpha_{\lambda }$.*
*(P7)*  *If p>0 and $q>\max\{0,\beta_{\lambda }\}$, then the sequence ${\left(b_{n}^{\left(p,q\right)}\right)}_{n\in N}$ is strictly increasing.*
  *(P7a)*  *If additionally $q\leq \min\left\{1\;,\;e^{\beta_{\lambda }-1}\right\}$, then the sequence ${\left(b_{n}^{\left(p,q\right)}\right)}_{n\in N}$ converges to $p\cdot e^{x_{0}^{\left(q\right)}}-\alpha_{\lambda }\in ]p-\alpha_{\lambda },p/q-\alpha_{\lambda }]$; this limit can take any sign, depending on the parameter constellation.*
  *(P7b)*  *If additionally $q>\min\left\{1\;,\;e^{\beta_{\lambda }-1}\right\}$, then the sequence ${\left(b_{n}^{\left(p,q\right)}\right)}_{n\in N}$ diverges to ∞, faster than exponentially.*
*(P8)*  *For the remaining cases we get: $b_{n}^{\left(0,q\right)}\equiv -\alpha_{\lambda }$ and $b_{n}^{\left(p,0\right)}\equiv p\cdot e^{-\beta_{\lambda }}-\alpha_{\lambda }$ (p∈R,q∈R).* *Moreover, in our investigations we will repeatedly make use of the function $\xi_{\lambda }^{\left(q\right)}\left(\cdot \right)$ from the definition (36) of $a_{n}^{\left(q\right)}$ (see also (44)), which has the following properties:*
*(P9)*  *For q∈]0,∞[ and all λ∈R\{0,1} the function $\xi_{\lambda }^{\left(q\right)}\left(\cdot \right)$ is strictly increasing, strictly convex and smooth, and there holds*
  *(P9a)*  $$\xi_{\lambda }^{\left(q\right)}\left(0\right)\;\left\{\begin{array}{cc} <\;0, & \mathrm{if}\;q<\beta_{\lambda },\\ =\;0, & \mathrm{if}\;q=\beta_{\lambda },\\ >\;0, & \mathrm{if}\;q>\beta_{\lambda }. \end{array}\right.$$
  *(P9b)*  $$\lim_{x\to -\infty }\;\xi_{\lambda }^{\left(q\right)}\left(x\right)\;=\;-\beta_{\lambda }\;,\;\mathrm{and}\;\lim_{x\to \infty }\;\xi_{\lambda }^{\left(q\right)}\left(x\right)\;=\;\infty \;.$$

The proof of these properties is provided in Appendix A.1. From Properties 1 (P1) to (P4) we can see, that the behaviour of the sequence ${\left(a_{n}^{\left(q\right)}\right)}_{n\in N}$ can be classified basically into four different types; besides the case (P2) where $a_{n}^{\left(q\right)}$ is *constant*, the sequence can be either (i) *strictly decreasing and convergent* (e.g., for the NI case $\left(\beta_{A},\beta_{H},\alpha_{A},\alpha_{H},\lambda \right)=\left(0.5,2,0,0,0.5\right)$ leading to $\beta_{\lambda }=\lambda \beta_{A}+\left(1-\lambda \right)\beta_{H}=1.25$ and to $q:=q_{\lambda }^{E}=\beta_{A}^{\lambda }\beta_{H}^{1-\lambda }=1$, cf. (33) resp. Theorem 1(a)), or (ii) *strictly increasing and convergent* (e.g., for $\left(\beta_{A},\beta_{H},\alpha_{A},\alpha_{H},\lambda \right)=\left(0.5,2,0,0,1.5\right)$ leading to $\beta_{\lambda }=-0.25$, $q:=q_{\lambda }^{E}=0.25$), or (iii) *strictly increasing and divergent* (e.g., for $\left(\beta_{A},\beta_{H},\alpha_{A},\alpha_{H},\lambda \right)=\left(0.5,2,0,0,2.7\right)$ leading to $\beta_{\lambda }=-2.05$, $q:=q_{\lambda }^{E}\approx 0.047366$). Within our running-example epidemiological context of Section 2.3, this corresponds to a “potentially dangerous” infectious-disease-transmission situation (H) (with supercritical reproduction number $\beta_{H}=2$), whereas (A) describes a “mild” situation (with “low” subcritical $\beta_{A}=0.5$).

As already mentioned before, the sequences ${\left(a_{n}^{\left(q\right)}\right)}_{n\in N}$ and ${\left(b_{n}^{\left(p,q\right)}\right)}_{n\in N}$–whose behaviours for general p≥0 and q≥0 were described by the Properties 1–have to be evaluated at setup-dependent choices $p=p_{\lambda }=p\left(\beta_{A},\beta_{H},\alpha_{A},\alpha_{H},\lambda \right)$ and $q=q_{\lambda }=q\left(\beta_{A},\beta_{H},\alpha_{A},\alpha_{H},\lambda \right)$. Hence, for fixed $\left(\beta_{A},\beta_{H},\alpha_{A},\alpha_{H}\right)$, one of the questions–which arises in the course of the desired investigations of the time-behaviour of the Hellinger integral bounds (resp. exact values)–is for which λ∈R the sequence ${\left(a_{n}^{\left(q_{\lambda }\right)}\right)}_{n\in N}$ converges. In the following, we illuminate this for the important special case $q_{\lambda }=\beta_{A}^{\lambda }\beta_{H}^{1-\lambda }$. Suppose at first that $\beta_{A}\neq \beta_{H}$. Properties 1 (P1) implies that for λ∈]0,1[ one has $\lim_{n\to \infty }a_{n}^{\left(q_{\lambda }\right)}=x_{0}^{\left(q_{\lambda }\right)}\in ]-\beta_{\lambda },q_{\lambda }-\beta_{\lambda }[$, and Lemma A1 states that $q_{\lambda }-\beta_{\lambda }<0$. For λ∈R\[0,1], there holds $q_{\lambda }>\max\left\{0,\beta_{\lambda }\right\}$, and from (P3) one can see that ${\left(a_{n}^{\left(q_{\lambda }\right)}\right)}_{n\in N}$ does not converge to $x_{0}^{\left(q_{\lambda }\right)}$ in general, but for $q_{\lambda }\leq \min\left\{1,e^{\beta_{\lambda }-1}\right\}$ which constitutes an implicit condition on λ. This can be made explicit, with the help of the auxiliary variables

$$\begin{array}{c} \;\lambda_{-}\;:=\;\lambda_{-}\left(\beta_{A},\beta_{H}\right)\;:=\;\left\{\begin{array}{c} \inf\left\{\lambda \leq 0\;:\;\beta_{A}^{\lambda }\beta_{H}^{1-\lambda }\leq \min\left\{1\;,\;\exp\{\lambda \beta_{A}+\left(1-\lambda \right)\beta_{H}-1\}\right\}\right\}\;,\\ \;\mathrm{in}\;\mathrm{case}\;\mathrm{that}\;\mathrm{the}\;\mathrm{set}\;\mathrm{is}\;\mathrm{nonempty},\\ 0,\;\mathrm{else}, \end{array}\right.\\ \;\lambda_{+}\;:=\;\lambda_{+}\left(\beta_{A},\beta_{H}\right)\;:=\;\left\{\begin{array}{c} \sup\left\{\lambda \geq 1\;:\;\beta_{A}^{\lambda }\beta_{H}^{1-\lambda }\leq \min\left\{1\;,\;\exp\{\lambda \beta_{A}+\left(1-\lambda \right)\beta_{H}-1\}\right\}\right\}\;,\\ \;\mathrm{in}\;\mathrm{case}\;\mathrm{that}\;\mathrm{the}\;\mathrm{set}\;\mathrm{is}\;\mathrm{nonempty},\\ 1,\;\mathrm{else}. \end{array}\right. \end{array}$$

For the constellation $\beta_{A}=\beta_{H}>0$ we clearly obtain $q_{\lambda }=\beta_{A}^{\lambda }\beta_{H}^{1-\lambda }=\beta_{A}=\beta_{H}=\beta_{\lambda }$. Hence, (P2) implies that the sequence ${\left(a_{n}^{\left(q_{\lambda }\right)}\right)}_{n\in N}$ converges *for all* λ∈R\{0,1} and we can set $\lambda_{-}:=-\infty$ as well as $\lambda_{+}:=\infty$. Incorporating this and by adapting a result of Linkov & Lunyova [53] on $\lambda_{-}\left(v_{1},v_{2}\right),\;\lambda_{+}\left(v_{1},v_{2}\right)$ for $\beta_{A}\neq \beta_{H}$, we end up with

**Lemma** **1.** *(a) For all $\beta_{A}>0,\;\beta_{H}>0$ with $\beta_{A}\neq \beta_{H}$ there holds*

$$\lambda_{-}\;=\;\lambda_{-}\left(\beta_{A},\beta_{H}\right)\;=\;\left\{\begin{array}{cc} 0, & \mathrm{if}\;\beta_{H}\geq 1,\;\\ \overset{˘}{\lambda }, & \mathrm{if}\;\beta_{H}<1\;\mathrm{and}\;\beta_{A}\notin \left[\beta_{H},\beta_{H}\;z\left(\beta_{H}\right)\right],\;\\ -\infty , & \mathrm{if}\;\beta_{H}<1\;\mathrm{and}\;\beta_{A}\in ]\beta_{H},\beta_{H}\;z\left(\beta_{H}\right)], \end{array}\right.$$

$$\lambda_{+}\;=\;\lambda_{+}\left(\beta_{A},\beta_{H}\right)\;=\;\left\{\begin{array}{cc} 1, & \mathrm{if}\;\beta_{A}\geq 1,\;\\ \overset{˘}{\lambda }, & \mathrm{if}\;\beta_{A}<1\;\mathrm{and}\;\beta_{H}\notin \left[\beta_{A},\beta_{A}\;z\left(\beta_{A}\right)\right],\;\\ \infty , & \mathrm{if}\;\beta_{A}<1\;\mathrm{and}\;\beta_{H}\in ]\beta_{A},\beta_{A}\;z\left(\beta_{A}\right)], \end{array}\right.$$

*where*

$$\overset{˘}{\lambda }\;:=\;\overset{˘}{\lambda }\left(\beta_{A},\beta_{H}\right)\;:=\;\frac{\beta_{H}-1-\log\left(\beta_{H}\right)}{\beta_{H}-\beta_{A}+\log\left(\frac{\beta_{A}}{\beta_{H}}\right)}\;\left\{\begin{array}{cc} <\;0, & \mathrm{if}\;\beta_{H}<1\;\mathrm{and}\;\beta_{A}\notin \left[\beta_{H},\beta_{H}\;z\left(\beta_{H}\right)\right],\;\\ >\;1, & \mathrm{if}\;\beta_{A}<1\;\mathrm{and}\;\beta_{H}\notin \left[\beta_{A},\beta_{A}\;z\left(\beta_{A}\right)\right]. \end{array}\right.$$

*Here, for fixed β∈]0,∞[\{1} we denote by z(β) the unique solution of the equation log(x)−β(x−1)=0, x∈]0,∞[\{1}. For β=1, z(β)=1 denotes the unique solution of log(x)−(x−1)=0,x∈]0,∞[.* *(b) For all $\beta_{A}=\beta_{H}>0$ one gets $\lambda_{-}=\lambda_{-}\left(\beta_{A},\beta_{H}\right)=-\infty$ as well as $\lambda_{+}=\lambda_{+}\left(\beta_{A},\beta_{H}\right)=\infty$.* *Notice that the relationship $\overset{˘}{\lambda }\left(\beta_{A},\beta_{H}\right)=1-\overset{˘}{\lambda }\left(\beta_{H},\beta_{A}\right)$ is consistent with the skew symmetry (8).*

A corresponding proof is given in Appendix A.1.

With these auxiliary basic facts in hand, let us now work out our detailed investigations of the time-behaviour $n\mapsto H_{\lambda }(P_{A,n}∥P_{H,n})$, where we start with the exactly treatable case (a) in Theorem 1.

### 3.3. Detailed Analyses of the Exact Recursive Values, i.e., for the Cases $\left(\beta_{A},\beta_{H},\alpha_{A},\alpha_{H}\right)\in P_{\mathrm{NI}}\cup P_{\mathrm{SP},1}$

In the no-immigration-case $\left(\beta_{A},\beta_{H},\alpha_{A},\alpha_{H}\right)\in P_{\mathrm{NI}}$ and in the equal-fraction-case $\left(\beta_{A},\beta_{H},\alpha_{A},\alpha_{H}\right)\in P_{\mathrm{SP},1}$, the Hellinger integral can be calculated exactly in terms of $H_{\lambda }(P_{A,n}∥P_{H,n})=V_{\lambda ,X_{0},n}$ (cf. (39)), as proposed in part (a) of Theorem 1. This quantity depends on the behaviour of the sequence ${\left(a_{n}^{\left(q_{\lambda }^{E}\right)}\right)}_{n\in N}$, with $q_{\lambda }^{E}:=\beta_{A}^{\lambda }\beta_{H}^{1-\lambda }>0$, and of the sum ${\left(\frac{\alpha_{A}}{\beta_{A}}\sum_{k=1}^{n}a_{k}^{\left(q_{\lambda }^{E}\right)}\right)}_{n\in N}$. The last expression is equal to zero on $P_{\mathrm{NI}}$. On $P_{\mathrm{SP},1}$, this sum is unequal to zero. Using Lemma A1 we conclude that $q_{\lambda }^{E}<\beta_{\lambda }$ (resp. $q_{\lambda }^{E}>\beta_{\lambda }$) iff λ∈]0,1[ (resp. λ∈R\[0,1]), since on $P_{\mathrm{NI}}\cup P_{\mathrm{SP},1}$ there holds $\beta_{A}\neq \beta_{H}$. Thus, from Properties 1 (P1) we can see that the sequence ${\left(a_{n}^{\left(q_{\lambda }^{E}\right)}\right)}_{n\in N}$ is strictly negative, strictly decreasing and it converges to the unique solution $x_{0}^{\left(q_{\lambda }^{E}\right)}\in ]-\beta_{\lambda },q_{\lambda }^{E}-\beta_{\lambda }[$ of the Equation (44) if λ∈]0,1[. For λ∈R\[0,1], (P3) implies that the sequence ${\left(a_{n}^{\left(q_{\lambda }^{E}\right)}\right)}_{n\in N}$ is strictly positive, strictly increasing and converges to the smallest positive solution $x_{0}^{\left(q_{\lambda }^{E}\right)}\in ]0,-\log\left(q_{\lambda }^{E}\right)]$ of the Equation (44) in case that (P3a) is satisfied, otherwise it diverges to *∞*. Thus, we have shown the following detailed behaviour of Hellinger integrals:

**Proposition** **2.** *For all $\left(\beta_{A},\beta_{H},\alpha_{A},\alpha_{H},\lambda \right)\in P_{\mathrm{NI}}\times ]0,1[$ and all initial population sizes $X_{0}\in N$ there holds*

$$\begin{array}{cc} \left(a\right) & \;H_{\lambda }(P_{A,1}∥P_{H,1})\;=\;\exp\left\{\left(\beta_{A}^{\lambda }\;\beta_{H}^{1-\lambda }-\lambda \beta_{A}-\left(1-\lambda \right)\beta_{H}\right)\;X_{0}\right\}\;<\;1,\;\;\\ \left(b\right) & \;\mathrm{the}\;\mathrm{sequence}{\left(H_{\lambda }(P_{A,n}∥P_{H,n})\right)}_{n\in N}\;\mathrm{given}\;\mathrm{by}\\ & \;H_{\lambda }(P_{A,n}∥P_{H,n})\;=\;\exp\left\{a_{n}^{\left(q_{\lambda }^{E}\right)}\;X_{0}\right\}\;=:\;V_{\lambda ,X_{0},n}\\ & \;\mathrm{is}\;\mathrm{strictly}\;\mathrm{decreasing},\\ \left(c\right) & \;\lim_{n\to \infty }\;H_{\lambda }(P_{A,n}∥P_{H,n})\;=\;\exp\left\{x_{0}^{\left(q_{\lambda }^{E}\right)}\;X_{0}\right\}\in \;]0,1[\;,\\ \left(d\right) & \;\lim_{n\to \infty }\;\frac{1}{n}\;\log H_{\lambda }(P_{A,n}∥P_{H,n})\;=\;0\;\\ \left(e\right) & \;\mathrm{the}\;\mathrm{map}\;X_{0}\;\mapsto \;V_{\lambda ,X_{0},n}\;\mathrm{is}\;\mathrm{strictly}\;\mathrm{decreasing}. \end{array}$$

**Proposition** **3.** *For all $\left(\beta_{A},\beta_{H},\alpha_{A},\alpha_{H},\lambda \right)\in P_{\mathrm{NI}}\times \left(R\backslash \left[0,1\right]\right)$ and all initial population sizes $X_{0}\in N$ there holds with $q_{\lambda }^{E}:=\beta_{A}^{\lambda }\beta_{H}^{1-\lambda }$*

$$\begin{array}{cc} \left(a\right) & \;H_{\lambda }(P_{A,1}∥P_{H,1})\;=\;\exp\left\{\left(\beta_{A}^{\lambda }\;\beta_{H}^{1-\lambda }-\beta_{\lambda }\right)\cdot X_{0}\right\}\;>\;1,\;\;\\ \left(b\right) & \;\mathrm{the}\;\mathrm{sequence}{\left(H_{\lambda }(P_{A,n}∥P_{H,n})\right)}_{n\in N}\;\mathrm{given}\;\mathrm{by}\\ & \;H_{\lambda }(P_{A,n}∥P_{H,n})\;=\;\exp\left\{a_{n}^{\left(q_{\lambda }^{E}\right)}\cdot X_{0}\right\}\;=:\;V_{\lambda ,X_{0},n}\\ & \;\mathrm{is}\;\mathrm{strictly}\;\mathrm{increasing},\\ \left(c\right) & \;\lim_{n\to \infty }\;H_{\lambda }(P_{A,n}∥P_{H,n})\;=\;\left\{\begin{array}{cc} \exp\left\{x_{0}^{\left(q_{\lambda }^{E}\right)}\cdot X_{0}\right\}>\;1, & \mathrm{if}\;\lambda \in \left[\lambda_{-},\lambda_{+}\right]\;\backslash \;\left[0,1\right],\\ \infty , & \mathrm{if}\;\lambda \in \;]-\infty ,\lambda_{-}\left[\;\cup \;\right]\lambda_{+},\infty [\;, \end{array}\right.\;\\ \left(d\right) & \;\lim_{n\to \infty }\;\frac{1}{n}\;\log H_{\lambda }(P_{A,n}∥P_{H,n})\;=\;\left\{\begin{array}{cc} 0, & \mathrm{if}\;\lambda \in \left[\lambda_{-},\lambda_{+}\right]\;\backslash \;\left[0,1\right],\\ \infty , & \mathrm{if}\;\lambda \in \;]-\infty ,\lambda_{-}\left[\;\cup \;\right]\lambda_{+},\infty [\;, \end{array}\right.\\ \left(e\right) & \;\mathrm{the}\;\mathrm{map}\;X_{0}\;\mapsto \;V_{\lambda ,X_{0},n}\;\mathrm{is}\;\mathrm{strictly}\;\mathrm{increasing}. \end{array}$$

In the case $\left(\beta_{A},\beta_{H},\alpha_{A},\alpha_{H}\right)\in P_{\mathrm{SP},1}$, the sequence ${\left(a_{n}^{\left(q_{\lambda }^{E}\right)}\right)}_{n\in N}$ under consideration is formally the same, with the parameter $q_{\lambda }^{E}:=\beta_{A}^{\lambda }\beta_{H}^{1-\lambda }>0$. However, in contrast to the case $P_{\mathrm{NI}}$, on $P_{\mathrm{SP},1}$ both the sequence ${\left(a_{n}^{\left(q_{\lambda }^{E}\right)}\right)}_{n\in N}$ and the sum ${\left(\frac{\alpha_{A}}{\beta_{A}}\sum_{k=1}^{n}a_{k}^{\left(q_{\lambda }^{E}\right)}\right)}_{n\in N}$ are strictly decreasing in case that λ∈]0,1[, and strictly increasing in case that λ∈R\[0,1]. The respective convergence behaviours are given in Properties 1 (P1) and (P3). We thus obtain

**Proposition** **4.** *For all $\left(\beta_{A},\beta_{H},\alpha_{A},\alpha_{H},\lambda \right)\in P_{\mathrm{SP},1}\times ]0,1[$ and all initial population sizes $X_{0}\in N$ there holds with $q_{\lambda }^{E}:=\beta_{A}^{\lambda }\beta_{H}^{1-\lambda }$*

$$\begin{array}{cc} \left(a\right) & H_{\lambda }(P_{A,1}∥P_{H,1})\;=\;\exp\left\{\left(\beta_{A}^{\lambda }\;\beta_{H}^{1-\lambda }-\beta_{\lambda }\right)\cdot \left(X_{0}+\frac{\alpha_{A}}{\beta_{A}}\right)\right\}\;<\;1,\\ \left(b\right) & \;\mathrm{the}\;\mathrm{sequence}{\left(H_{\lambda }(P_{A,n}∥P_{H,n})\right)}_{n\in N}\;\mathrm{given}\;\mathrm{by}\\ & \;H_{\lambda }(P_{A,n}∥P_{H,n})\;=\;\exp\left\{a_{n}^{\left(q_{\lambda }^{E}\right)}\cdot X_{0}+\frac{\alpha_{A}}{\beta_{A}}\sum_{k=1}^{n}a_{k}^{\left(q_{\lambda }^{E}\right)}\right\}\;=:\;V_{\lambda ,X_{0},n}\;\\ & \;\mathrm{is}\;\mathrm{strictly}\;\mathrm{decreasing},\\ \left(c\right) & \;\lim_{n\to \infty }\;H_{\lambda }(P_{A,n}∥P_{H,n})\;=\;0\;,\\ \left(d\right) & \;\lim_{n\to \infty }\;\frac{1}{n}\;\log H_{\lambda }(P_{A,n}∥P_{H,n})\;=\;\frac{\alpha_{A}}{\beta_{A}}\cdot x_{0}^{\left(q_{\lambda }^{E}\right)}\;<\;0\;,\\ \left(e\right) & \;\mathrm{the}\;\mathrm{map}\;X_{0}\;\mapsto \;V_{\lambda ,X_{0},n}\;\mathrm{is}\;\mathrm{strictly}\;\mathrm{decreasing}. \end{array}$$

**Proposition** **5.** *For all $\left(\beta_{A},\beta_{H},\alpha_{A},\alpha_{H},\lambda \right)\in P_{\mathrm{SP},1}\times \left(R\backslash \left[0,1\right]\right)$ and all initial population sizes $X_{0}\in N$ there holds with $q_{\lambda }^{E}:=\beta_{A}^{\lambda }\beta_{H}^{1-\lambda }$*

$$\begin{array}{cc} \left(a\right) & \;H_{\lambda }(P_{A,1}∥P_{H,1})\;=\;\exp\left\{\left(\beta_{A}^{\lambda }\;\beta_{H}^{1-\lambda }-\beta_{\lambda }\right)\cdot \left(X_{0}+\frac{\alpha_{A}}{\beta_{A}}\right)\right\}\;>\;1,\;\;\\ \left(b\right) & \;\mathrm{the}\;\mathrm{sequence}{\left(H_{\lambda }(P_{A,n}∥P_{H,n})\right)}_{n\in N}\;\mathrm{given}\;\mathrm{by}\\ & \;H_{\lambda }(P_{A,n}∥P_{H,n})\;=\;\exp\left\{a_{n}^{\left(q_{\lambda }^{E}\right)}\cdot X_{0}+\frac{\alpha_{A}}{\beta_{A}}\sum_{k=1}^{n}a_{k}^{\left(q_{\lambda }^{E}\right)}\right\}\;=:\;V_{\lambda ,X_{0},n}\\ & \;\mathrm{is}\;\mathrm{strictly}\;\mathrm{increasing},\\ \left(c\right) & \;\lim_{n\to \infty }\;H_{\lambda }(P_{A,n}∥P_{H,n})\;=\;\infty ,\\ \left(d\right) & \;\lim_{n\to \infty }\;\frac{1}{n}\;\log H_{\lambda }(P_{A,n}∥P_{H,n})\;=\;\left\{\begin{array}{cc} \frac{\alpha_{A}}{\beta_{A}}\cdot x_{0}^{\left(q_{\lambda }^{E}\right)}\;>\;0, & \mathrm{if}\;\lambda \in \left[\lambda_{-},\lambda_{+}\right]\;\backslash \;\left[0,1\right]\;,\;\\ \infty , & \mathrm{if}\;\lambda \in \;]-\infty ,\lambda_{-}\left[\;\cup \;\right]\lambda_{+},\infty [\;, \end{array}\right.\\ \left(e\right) & \;\mathrm{the}\;\mathrm{map}\;X_{0}\;\mapsto \;V_{\lambda ,X_{0},n}\;\mathrm{is}\;\mathrm{strictly}\;\mathrm{increasing}. \end{array}$$

Due to the nature of the equal-fraction-case $P_{\mathrm{SP},1}$, in the assertions (a), (b), (d) of the Propositions 4 and 5, the fraction $\alpha_{A}/\beta_{A}$ can be equivalently replaced by $\alpha_{H}/\beta_{H}$.

**Remark** **2.** *For the (to our context) incompatible setup of GWI with Poisson offspring but nonstochastic immigration of constant value 1, an “analogue” of part (d) of the Propositions 4 resp. 5 was established in Linkov & Lunyova [53].*

### 3.4. Some Preparatory Basic Facts for the Remaining Cases $\left(\beta_{A},\beta_{H},\alpha_{A},\alpha_{H}\right)\in P_{\mathrm{SP}}\backslash P_{\mathrm{SP},1}$

The bounds $B_{\lambda ,X_{0},n}^{L},\;B_{\lambda ,X_{0},n}^{U}$ for the Hellinger integral introduced in formula (40) in Theorem 1 can be chosen arbitrarily from a $\left(p_{\lambda }^{L},q_{\lambda }^{L},p_{\lambda }^{U},q_{\lambda }^{U}\right)$-indexed set of context-specific parameters satisfying (34), or equivalently (35).

In order to derive bounds which are optimal, with respect to goals that will be discussed later, the following monotonicity properties of the sequences ${\left(a_{n}^{\left(q\right)}\right)}_{n\in N}$ and ${\left(b_{n}^{\left(p,q\right)}\right)}_{n\in N}$ (cf. (36), (37)) for general, context-independent parameters *q* and *p*, will turn out to be very useful:

**Properties** **2.**

*(P10)*  *For $0\leq q_{1}<q_{2}<\infty$ there holds $a_{n}^{\left(q_{1}\right)}<a_{n}^{\left(q_{2}\right)}$ for all n∈N.*
*(P11)*  *For each fixed q≥0 and $0\leq p_{1}<p_{2}<\infty$ there holds $b_{n}^{\left(p_{1},q\right)}<b_{n}^{\left(p_{2},q\right)}$, for all n∈N.*
*(P12)*  *For fixed p>0 and $0\leq q_{1}<q_{2}$ it follows $b_{n}^{\left(p,q_{1}\right)}<b_{n}^{\left(p,q_{2}\right)}$ for all n∈N.*
*(P13)*  *Suppose that $0\leq p_{1}<p_{2}$ and $0\leq q_{2}<q_{1}$. For fixed n∈N, no dominance assertion can be conjectured for $b_{n}^{\left(p_{1},q_{1}\right)},b_{n}^{\left(p_{2},q_{2}\right)}$. As an example, consider the setup $\left(\beta_{A},\beta_{H},\alpha_{A},\alpha_{H},\lambda \right)=\left(0.4,0.8,5,3,0.5\right)$; within our running-example epidemiological context of Section 2.3, this corresponds to a “nearly dangerous” infectious-disease-transmission situation (H) (with nearly critical reproduction number $\beta_{H}=0.8$ and importation mean of $\alpha_{H}=3$), whereas (A) describes a “mild” situation (with “low” subcritical $\beta_{A}=0.4$ and $\alpha_{A}=5$). On the nonnegative real line, the function $\phi_{\lambda }\left(x\right)$ can be bounded from above by the linear functions $\phi_{\lambda }^{U,1}\left(x\right):=p_{1}+q_{1}x:=4.040+0.593\cdot x$ as well as by $\phi_{\lambda }^{U,2}\left(x\right):=p_{2}+q_{2}x:=4.110+0.584\cdot x$. Clearly, $p_{1}<p_{2}$ and $q_{1}>q_{2}$. Let us show the first eight elements and the respective limits of the corresponding sequences $b_{n}^{\left(p_{1},q_{1}\right)},b_{n}^{\left(p_{2},q_{2}\right)}$:*

*n*
12345678⋯
*∞*

$b_{n}^{\left(p_{1},q_{1}\right)}$
0.0400.011−0.005−0.015−0.021−0.024−0.026−0.028⋯−0.029
$b_{n}^{\left(p_{2},q_{2}\right)}$
0.1100.0450.007−0.014−0.026−0.033−0.036−0.039⋯−0.041
*(P14)*  *For arbitrary $0<p_{1},p_{2}$ and $0\leq q_{1},q_{2}\leq \min\left\{1,e^{\beta_{\lambda }-1}\right\}$ suppose that $\log\left(p_{1}\right)+x_{0}^{\left(q_{1}\right)}\;<\;\log\left(p_{2}\right)+x_{0}^{\left(q_{2}\right)}$. Then there holds*
  $$p_{1}\cdot e^{x_{0}^{\left(q_{1}\right)}}-\alpha_{\lambda }\;=\;\lim_{n\to \infty }\frac{1}{n}\sum_{k=1}^{n}b_{k}^{\left(p_{1},q_{1}\right)}\;<\;\lim_{n\to \infty }\frac{1}{n}\sum_{k=1}^{n}b_{k}^{\left(p_{2},q_{2}\right)}\;=\;p_{2}\cdot e^{x_{0}^{\left(q_{2}\right)}}-\alpha_{\lambda }\;.$$

From (P10) to (P12) one deduces that both sequences ${\left(a_{n}^{\left(q\right)}\right)}_{n\in N}$ and ${\left(b_{n}^{\left(p,q\right)}\right)}_{n\in N}$ are monotone in the general parameters p,q≥0. Thus, for the upper bound of the Hellinger integral $B_{\lambda ,X_{0},n}^{U}$ we should use nonnegative context-specific parameters $p_{\lambda }^{U}=p^{U}\left(\beta_{A},\beta_{H},\alpha_{A},\alpha_{H},\lambda \right)$ and $q_{\lambda }^{U}=q^{U}\left(\beta_{A},\beta_{H},\alpha_{A},\alpha_{H},\lambda \right)$ which are as small as possible, and for the lower bound $B_{\lambda ,X_{0},n}^{L}$ we should use nonnegative context-specific parameters $p_{\lambda }^{L}=p^{L}\left(\beta_{A},\beta_{H},\alpha_{A},\alpha_{H},\lambda \right)$ and $q_{\lambda }^{L}=q^{L}\left(\beta_{A},\beta_{H},\alpha_{A},\alpha_{H},\lambda \right)$ which are as large as possible, of course, subject to the (equivalent) restrictions (34) and (35).

To find “optimal” parameter pairs, we have to study the following properties of the function $\phi_{\lambda }\left(\cdot \right)=\phi \left(\cdot ,\beta_{A},\beta_{H},\alpha_{A},\alpha_{H},\lambda \right)$ defined on [0,∞[ in (30) (which are also valid for the previous parameter context $\left(\beta_{A},\beta_{H},\alpha_{A},\alpha_{H}\right)\in \left(P_{\mathrm{NI}}\cup P_{\mathrm{SP},1}\right)$):

**Properties** **3.**

*(P15)*  *One has*
  $$\phi_{\lambda }\left(x\right)={\left(\alpha_{A}+\beta_{A}x\right)}^{\lambda }{\left(\alpha_{H}+\beta_{H}x\right)}^{1-\lambda }\;-\;\lambda \left(\alpha_{A}+\beta_{A}x\right)+\left(1-\lambda \right)\left(\alpha_{H}+\beta_{H}x\right)\left\{\begin{array}{cc} \leq 0, & \mathrm{if}\;\lambda \;\in \;]0,1[,\;\\ \geq 0, & \mathrm{if}\;\lambda \in R\backslash [0,1], \end{array}\right.$$
  where equality holds iff $f_{A}\left(x\right)=f_{H}\left(x\right)$ for some x∈[0,∞[ iff $x=x^{*}:=\frac{\alpha_{A}-\alpha_{H}}{\beta_{H}-\beta_{A}}\in [0,\infty [$.
*(P16)*  *There holds*
  $$\phi_{\lambda }\left(0\right)=\alpha_{A}^{\lambda }\alpha_{H}^{1-\lambda }-\alpha_{\lambda }\left\{\begin{array}{cc} \leq 0, & \mathrm{if}\;\lambda \;\in \;]0,1[,\;\\ \geq 0, & \mathrm{if}\;\lambda \in R\backslash [0,1], \end{array}\right.$$
  *with equality iff $\alpha_{A}=\alpha_{H}$ together with $\beta_{A}\neq \beta_{H}$ (cf. Lemma A1).*
*(P17)*  *For all λ∈R\{0,1} one gets*
  $$\phi_{\lambda }^{\prime }\left(x\right)\;=\;\lambda \beta_{A}{\left(f_{A}\left(x\right)\right)}^{\lambda -1}{\left(f_{H}\left(x\right)\right)}^{1-\lambda }+\left(1-\lambda \right)\beta_{H}{\left(f_{A}\left(x\right)\right)}^{\lambda }{\left(f_{H}\left(x\right)\right)}^{-\lambda }-\beta_{\lambda }\;.$$
*(P18)*  *There holds*
  $$\lim_{x\to \infty }\phi_{\lambda }^{\prime }\left(x\right)=\beta_{A}^{\lambda }\beta_{H}^{1-\lambda }-\beta_{\lambda }\left\{\begin{array}{cc} \leq 0, & \mathrm{if}\;\lambda \;\in \;]0,1[,\;\\ \geq 0, & \mathrm{if}\;\lambda \in R\backslash [0,1], \end{array}\right.$$
  *with equality iff $\beta_{A}=\beta_{H}$ together with $\alpha_{A}\neq \alpha_{H}$ (cf. Lemma A1).*
*(P19)*  *There holds*
  $$\phi_{\lambda }^{\prime \prime }\left(x\right)\;=\;-\lambda \left(1-\lambda \right){\left(f_{A}\left(x\right)\right)}^{\lambda -2}{\left(f_{H}\left(x\right)\right)}^{-\lambda -1}{\left(\alpha_{A}\beta_{H}-\alpha_{H}\beta_{A}\right)}^{2}\left\{\begin{array}{cc} \leq 0, & \mathrm{if}\;\lambda \;\in \;]0,1[,\;\\ \geq 0, & \mathrm{if}\;\lambda \in R\backslash [0,1], \end{array}\right.$$
  *with equality iff $\left(\beta_{A},\beta_{H},\alpha_{A},\alpha_{H}\right)\in \left(P_{\mathrm{NI}}\cup P_{\mathrm{SP},1}\right)$. Hence, for $\left(\beta_{A},\beta_{H},\alpha_{A},\alpha_{H}\right)\in P_{\mathrm{SP}}\backslash P_{\mathrm{SP},1}$, the function $\phi_{\lambda }$ is strictly concave (convex) for λ∈]0,1[ (λ∈R\[0,1]). Notice that $\phi_{\lambda }^{\prime }\left(0\right)=\lambda \beta_{A}{\left(\frac{\alpha_{A}}{\alpha_{H}}\right)}^{\lambda -1}+\left(1-\lambda \right)\beta_{H}{\left(\frac{\alpha_{A}}{\alpha_{H}}\right)}^{\lambda }-\beta_{\lambda }$ can be either negative (e.g., for the setup $\left(\beta_{A},\beta_{H},\alpha_{A},\alpha_{H},\lambda \right)\in \{\left(4,2,3,1,0.5\right)$, (4,2,5,1,2)}, or zero (e.g., for $\left(\beta_{A},\beta_{H},\alpha_{A},\alpha_{H},\lambda \right)\in \left\{(4,2,4,1,0.5),(4,2,3,1,2)\right\}$), or positive (e.g., for $\left(\beta_{A},\beta_{H},\alpha_{A},\alpha_{H},\lambda \right)\;\in \;\{\left(4,2,5,1,0.5\right)$, (4,2,2,1,2)}), where the exemplary parameter constellations have concrete interpretations in our running-example epidemiological context of Section 2.3. Accordingly, for λ∈]0,1[, due to concavity and (P17), the function $\phi_{\lambda }\left(\cdot \right)$ can be either strictly decreasing, or can obtain its global maximum in ]0,∞[, or–only in the case $\beta_{A}=\beta_{H}$—can be strictly increasing. Analogously, for λ∈R\[0,1], the function $\phi_{\lambda }\left(\cdot \right)$ can be either strictly increasing, or can obtain its global minimum in ]0,∞[, or–only in the case $\beta_{A}=\beta_{H}$—can be strictly decreasing.*
(P20) *For all λ∈R\{0,1} one has*
  $$\begin{array}{c} \lim_{x\to \infty }\left(\phi_{\lambda }\left(x\right)-\left(\tilde{r_{\lambda }}+\tilde{s_{\lambda }}\;x\right)\right)\;=\;0\;,\\ \mathrm{for}\;\tilde{r_{\lambda }}\;:=\;\tilde{p_{\lambda }}-\alpha_{\lambda }\;:=\;\lambda \alpha_{A}{\left(\frac{\beta_{A}}{\beta_{H}}\right)}^{\lambda -1}+\left(1-\lambda \right)\alpha_{H}{\left(\frac{\beta_{A}}{\beta_{H}}\right)}^{\lambda }\;-\;\alpha_{\lambda }\\ \mathrm{and}\;\tilde{s_{\lambda }}\;:=\;\tilde{q_{\lambda }}-\beta_{\lambda }\;:=\;\beta_{A}^{\lambda }\beta_{H}^{1-\lambda }-\beta_{\lambda }\;.\; \end{array}$$
  *The linear function $\tilde{\phi_{\lambda }}\left(x\right):=\tilde{r_{\lambda }}+\tilde{s_{\lambda }}\cdot x$ constitutes the* asymptote *of $\phi_{\lambda }\left(\cdot \right)$. Notice that if $\beta_{A}=\beta_{H}$ one has ${\tilde{s}}_{\lambda }=0={\tilde{r}}_{\lambda }$; if $\beta_{A}\neq \beta_{H}$ we have ${\tilde{s}}_{\lambda }<0$ in the case λ∈]0,1[ and ${\tilde{s}}_{\lambda }>0$ if λ∈R\[0,1]. Furthermore, $\phi_{\lambda }\left(0\right)<\tilde{r_{\lambda }}$ if λ∈]0,1[ and $\phi_{\lambda }\left(0\right)>\tilde{r_{\lambda }}$ if λ∈R\[0,1], (cf. Lemma A1(c1) and (c2)). If $\alpha_{A}=\alpha_{H}$ (and thus $\beta_{A}\neq \beta_{H}$), then the intercept $\tilde{r_{\lambda }}$ is strictly positive if λ∈]0,1[ resp. strictly negative if λ∈R\[0,1]. In contrast, for the case $\alpha_{A}\neq \alpha_{H}$, the intercept $\tilde{r_{\lambda }}$ can assume any sign, take e.g., $\left(\beta_{A},\beta_{H},\alpha_{A},\alpha_{H},\lambda \right)\in \left\{\left(3.7,0.9,2.0,1.0,0.5\right),\left(4,2,1.6,1,2\right)\right\}$ for $\tilde{r_{\lambda }}>0$, $\left(\beta_{A},\beta_{H},\alpha_{A},\alpha_{H},\lambda \right)\in \left\{\left(3.6,0.9,2.0,1.0,0.5\right),\left(4,2,1.5,1,2\right)\right\}$ for $\tilde{r_{\lambda }}=0$, and $\left(\beta_{A},\beta_{H},\alpha_{A},\alpha_{H},\lambda \right)\in \left\{\left(3.5,0.9,2.0,1.0,0.5\right),\left(4,2,1.4,1,2\right)\right\}$ for $\tilde{r_{\lambda }}<0$; again, the exemplary parameter constellations have concrete interpretations in our running-example epidemiological context of Section 2.3.*

The properties (P15) to (P20) above describe in detail the characteristics of the function $\phi_{\lambda }\left(\cdot \right)\;=\;\phi \left(\cdot ,\beta_{A},\beta_{H},\alpha_{A},\alpha_{H},\lambda \right)$. In the previous parameter setup $P_{\mathrm{NI}}\cup P_{\mathrm{SP},1}$, this function is linear, which can be seen from (P19). In the current parameter setup $P_{\mathrm{SP}}\backslash P_{\mathrm{SP},1}$, this function can basically be classified into four different types. From (P16) to (P20) it is easy to see that for all current parameter constellations the particular choices

(45) $$p_{\lambda }^{A}:=\alpha_{A}^{\lambda }\alpha_{H}^{1-\lambda }>0,\;q_{\lambda }^{A}:=\beta_{A}^{\lambda }\beta_{H}^{1-\lambda }>0,$$

which correspond to the following choices in (35)

$$r_{\lambda }^{A}:=\alpha_{A}^{\lambda }\alpha_{H}^{1-\lambda }-\alpha_{\lambda }\;\leq 0\;\left(resp.\geq 0\right),\;s_{\lambda }^{A}:=\beta_{A}^{\lambda }\beta_{H}^{1-\lambda }-\beta_{\lambda }\;\leq 0\;\left(resp.\geq 0\right),$$

– where A=L (resp. A=U)–lead to the tightest lower bound $B_{\lambda ,X_{0},n}^{L}$ (resp. upper bound $B_{\lambda ,X_{0},n}^{U}$) for $H_{\lambda }(P_{A,n}∥P_{H,n})$ in (40) in the case λ∈]0,1[ (resp. λ∈R\[0,1]). Notice that for the previous parameter setup $\left(\beta_{A},\beta_{H},\alpha_{A},\alpha_{H}\right)\in \left(P_{\mathrm{NI}}\cup P_{\mathrm{SP},1}\right)$ these choices led to the exact values of the Hellinger integral and to the simplification $\left(p_{\lambda }^{E}/q_{\lambda }^{E}\right)\cdot \beta_{\lambda }-\alpha_{\lambda }=0$, which implies $b_{n}^{\left(p_{\lambda }^{E},q_{\lambda }^{E}\right)}=\left(\alpha_{A}/\beta_{A}\right)\cdot a_{n}^{\left(q_{\lambda }^{E}\right)}$. In contrast, in the current parameter setup $\left(\beta_{A},\beta_{H},\alpha_{A},\alpha_{H}\right)\in P_{\mathrm{SP}}\backslash P_{\mathrm{SP},1}$ we only derive the *optimal* lower (resp. upper) bound for λ∈]0,1[ (resp. λ∈R\[0,1]) by using the parameters $p_{\lambda }^{A},\;q_{\lambda }^{A}$ for A=L (resp. A=U) and $\left(p_{\lambda }^{A}/q_{\lambda }^{A}\right)\cdot \beta_{\lambda }-\alpha_{\lambda }\neq 0$. For a better distinguishability and easier reference we thus stick to the L–notation (resp. U–notation) here.

### 3.5. Lower Bounds for the Cases $\left(\beta_{A},\beta_{H},\alpha_{A},\alpha_{H},\lambda \right)\in \left(P_{\mathrm{SP}}\backslash P_{\mathrm{SP},1}\right)\times ]0,1[$

The discussion above implies that the lower bound $B_{\lambda ,X_{0},n}^{L}$ for the Hellinger integral $H_{\lambda }(P_{A,n}∥P_{H,n})$ in (40) is optimal for the choices $p_{\lambda }^{L},\;q_{\lambda }^{L}>0$ defined in (45). If $\beta_{A}\neq \beta_{H}$, due to Properties 1 (P1) and Lemma A1, the sequence ${\left(a_{n}^{\left(q_{\lambda }^{L}\right)}\right)}_{n\in N}$ is strictly negative and strictly decreasing and converges to the unique negative solution of the Equation (44). Furthermore, due to (P5), the sequence ${\left(b_{n}^{\left(p_{\lambda }^{L},q_{\lambda }^{L}\right)}\right)}_{n\in N}$, as defined in (37), is strictly decreasing. Since $b_{1}^{\left(p_{\lambda }^{L},q_{\lambda }^{L}\right)}=p_{\lambda }^{L}-\alpha_{\lambda }\leq 0$ by Lemma A1, with equality iff $\alpha_{A}=\alpha_{H}$, the sequence ${\left(b_{n}^{\left(p_{\lambda }^{L},q_{\lambda }^{L}\right)}\right)}_{n\in N}$ is also strictly negative (with the exception $b_{1}^{\left(p_{\lambda }^{L},q_{\lambda }^{L}\right)}=0$ for $\alpha_{A}=\alpha_{H}$) and strictly decreasing. If $\beta_{A}=\beta_{H}$ and thus $\alpha_{A}\neq \alpha_{H}$, due to (P2), (P6) and Lemma A1, there holds $a_{n}^{\left(q_{\lambda }^{L}\right)}\equiv 0$ and $b_{n}^{\left(q_{\lambda }^{L}\right)}\equiv p_{\lambda }^{L}-\alpha_{\lambda }<0$. Thus, analogously to the cases $P_{\mathrm{NI}}\cup P_{\mathrm{SP},1}$ we obtain

**Proposition** **6.** *For all $\left(\beta_{A},\beta_{H},\alpha_{A},\alpha_{H},\lambda \right)\in \left(P_{\mathrm{SP}}\backslash P_{\mathrm{SP},1}\right)\times ]0,1[$ and all initial population sizes $X_{0}\in N$ there holds with $p_{\lambda }^{L}:=\alpha_{A}^{\lambda }\alpha_{H}^{1-\lambda },\;q_{\lambda }^{L}:=\beta_{A}^{\lambda }\beta_{H}^{1-\lambda }$*

$$\begin{array}{cc} \left(a\right) & \;B_{\lambda ,X_{0},1}^{L}\;=\;\exp\left\{\left(\beta_{A}^{\lambda }\;\beta_{H}^{1-\lambda }-\beta_{\lambda }\right)\cdot X_{0}\;+\;\alpha_{A}^{\lambda }\alpha_{H}^{1-\lambda }-\alpha_{\lambda }\right\}\;<\;1,\\ \left(b\right) & \;\mathrm{the}\;\mathrm{sequence}\;\mathrm{of}\;\mathrm{lower}\;\mathrm{bounds}\;{\left(B_{\lambda ,X_{0},n}^{L}\right)}_{n\in N}\;\mathrm{for}\;H_{\lambda }(P_{A,n}∥P_{H,n})\;\mathrm{given}\;\mathrm{by}\\ & \;B_{\lambda ,X_{0},n}^{L}\;=\;\exp\left\{a_{n}^{\left(q_{\lambda }^{L}\right)}\cdot X_{0}+\frac{p_{\lambda }^{L}}{q_{\lambda }^{L}}\sum_{k=1}^{n}a_{k}^{\left(q_{\lambda }^{L}\right)}+n\cdot \left(\frac{p_{\lambda }^{L}}{q_{\lambda }^{L}}\cdot \beta_{\lambda }-\alpha_{\lambda }\right)\right\}\;\mathrm{is}\;\mathrm{strictly}\;\mathrm{decreasing},\\ \left(c\right) & \;\lim_{n\to \infty }B_{\lambda ,X_{0},n}^{L}\;=\;0\;,\\ \left(d\right) & \;\lim_{n\to \infty }\;\frac{1}{n}\;\log B_{\lambda ,X_{0},n}^{L}\;=\;\frac{p_{\lambda }^{L}}{q_{\lambda }^{L}}\cdot \left(x_{0}^{\left(q_{\lambda }^{L}\right)}+\beta_{\lambda }\right)-\alpha_{\lambda }\;=\;p_{\lambda }^{L}\cdot e^{x_{0}^{\left(q_{\lambda }^{L}\right)}}-\alpha_{\lambda }\;<\;0\;.\\ \left(e\right) & \;\mathrm{the}\;\mathrm{map}\;\;X_{0}\;\mapsto \;B_{\lambda ,X_{0},n}^{L}\;\mathrm{is}\;\mathrm{strictly}\;\mathrm{decreasing}. \end{array}$$

### 3.6. Goals for Upper Bounds for the Cases $\left(\beta_{A},\beta_{H},\alpha_{A},\alpha_{H},\lambda \right)\in \left(P_{\mathrm{SP}}\backslash P_{\mathrm{SP},1}\right)\times ]0,1[$

For parameter constellations $\left(\beta_{A},\beta_{H},\alpha_{A},\alpha_{H},\lambda \right)\in \left(P_{\mathrm{SP}}\backslash P_{\mathrm{SP},1}\right)\times ]0,1[$, in contrast to the treatment of the lower bounds (cf. the previous Section 3.5), the fine-tuning of the *upper bounds* of the Hellinger integrals $H_{\lambda }(P_{A,n}∥P_{H,n})$ is much more involved. To begin with, let us mention that the monotonicity-concerning Properties 2 (P10) to (P12) imply that for a tight upper bound $B_{\lambda ,X_{0},n}^{U}$ (cf. (40)) one should choose parameters $p_{\lambda }^{U}\geq p_{\lambda }^{L}>0,\;q_{\lambda }^{U}\geq q_{\lambda }^{L}>0$ as small as possible. Due to the concavity (cf. Properties 3 (P19)) of the function $\phi_{\lambda }\left(\cdot \right)$, the linear upper bound $\phi_{\lambda }^{U}\left(\cdot \right)$ (on the ultimately relevant subdomain $N_{0}$) thus must hit the function $\phi_{\lambda }\left(\cdot \right)$ in at least one point $x\in N_{0}$, which corresponds to some “discrete tangent line” of $\phi_{\lambda }\left(\cdot \right)$ in *x*, or in at most two points $x,x+1\in N_{0}$, which corresponds to the secant line of $\phi_{\lambda }\left(\cdot \right)$ across its arguments *x* and x+1. Accordingly, there is in general *no overall best* upper bound; of course, one way to obtain “good” upper bounds for $H_{\lambda }(P_{A,n}∥P_{H,n})$ is to solve the optimization problem

(46) $$\left(\bar{p_{\lambda }^{U}},\bar{q_{\lambda }^{U}}\right)\;:=\;\arg\min_{\;(p_{\lambda }^{U},q_{\lambda }^{U})}\left\{\exp\left\{a_{n}^{\left(q_{\lambda }^{U}\right)}\cdot X_{0}\;+\;\sum_{k=1}^{n}b_{k}^{\left(p_{\lambda }^{U},q_{\lambda }^{U}\right)}\right\}\right\},$$

subject to the constraint (35). However, the corresponding result generally depends on the particular choice of the initial population $X_{0}\in N$ and on the observation time horizon n∈N. Hence, there is in general no overall optimal choice of $p_{\lambda }^{U},q_{\lambda }^{U}$ without the incorporation of further goal-dependent constraints such as $\lim_{n\to \infty }B_{\lambda ,X_{0},n}^{U}=0$ in case of $\lim_{n\to \infty }H_{\lambda }(P_{A,n}∥P_{H,n})=0$. By the way, mainly because of the non-explicitness of the sequence ${\left(a_{n}^{\left(q_{\lambda }^{U}\right)}\right)}_{n\in N}$ (due to the generally not explicitly solvable recursion (36)) and the discreteness of the constraint (35), this optimization problem seems to be not straightforward to solve, anyway. The choice of parameters $p_{\lambda }^{U},q_{\lambda }^{U}$ for the upper bound $B_{\lambda ,X_{0},n}^{U}\geq H_{\lambda }(P_{A,n}∥P_{H,n})$ can be made according to different, partially incompatible (“optimality-” resp. “goodness-”) criteria and goals, such as:

(G1) the validity of $B_{\lambda ,X_{0},n}^{U}<1$ *simultaneously* for all initial configurations $X_{0}\in N$, all observation horizons n∈N and all λ∈]0,1[, which leads to a *strict* improvement of the general upper bound $H_{\lambda }(P_{A,n}∥P_{H,n})<1$ (cf. (9));
(G2) the determination of the long-term-limits $\lim_{n\to \infty }H_{\lambda }(P_{A,n}∥P_{H,n})$ respectively $\lim_{n\to \infty }B_{\lambda ,X_{0},n}^{U}$ for all $X_{0}\in N$ and all λ∈]0,1[; in particular, one would like to check whether $\lim_{n\to \infty }H_{\lambda }(P_{A,n}∥P_{H,n})=0$, which implies that the families of probability distributions ${\left(P_{A,n}\right)}_{n\in N}$ and ${\left(P_{H,n}\right)}_{n\in N}$ are *asymptotically distinguishable* (entirely separated), cf. (25);
(G3) the determination of the time-asymptotical growth rates $\lim_{n\to \infty }\frac{1}{n}\log\left(H_{\lambda }(P_{A,n}∥P_{H,n})\right)$ resp. $\lim_{n\to \infty }\frac{1}{n}\log\left(B_{\lambda ,X_{0},n}^{U}\right)$ for all $X_{0}\in N$ and all λ∈]0,1[.

Further goals–with which we do not deal here for the sake of brevity–are for instance (i) a very good tightness of the upper bound $B_{\lambda ,X_{0},n}^{U}$ for n≥N for some fixed large N∈N, or (ii) the criterion (G1) with *fixed* (rather than arbitrary) initial population size $X_{0}\in N$.

Let us briefly discuss the three Goals (G1) to (G3) and their challenges: due to Theorem 1, Goal (G1) can only be achieved if the sequence ${\left(a_{n}^{\left(q_{\lambda }^{U}\right)}\right)}_{n\in N}$ is non-increasing, since otherwise, for each fixed observation horizon n∈N there is a large enough initial population size $X_{0}$ such that the upper bound component ${\tilde{B}}_{\lambda ,X_{0},n}^{\left(p_{\lambda }^{U},q_{\lambda }^{U}\right)}$ becomes larger than 1, and thus $B_{\lambda ,X_{0},n}^{U}=1$ (cf. (40)). Hence, Properties 1 (P1) and (P2) imply that one should have $q_{\lambda }^{U}\leq \beta_{\lambda }$. Then, the sequence ${\left(b_{n}^{\left(p_{\lambda }^{U},q_{\lambda }^{U}\right)}\right)}_{n\in N}$ is also non-increasing. However, since $b_{n}^{\left(p_{\lambda }^{U},q_{\lambda }^{U}\right)}$ might be positive for some (even all) n∈N, the sum ${\left(\sum_{k=1}^{n}b_{k}^{\left(p_{\lambda }^{U},q_{\lambda }^{U}\right)}\right)}_{n\in N}$ is not necessarily decreasing. Nevertheless, the restriction

(47) $$q_{\lambda }^{U}-\beta_{\lambda }\leq 0\;\mathrm{and}\;p_{\lambda }^{U}-\alpha_{\lambda }\leq 0,\;\mathrm{where}\;\mathrm{at}\;\mathrm{least}\;\mathrm{one}\;\mathrm{of}\;\mathrm{the}\;\mathrm{inequalities}\;\mathrm{is}\;\mathrm{strict},$$

ensures that both sequences ${\left(a_{n}^{\left(q_{\lambda }^{U}\right)}\right)}_{n\in N}$ and ${\left(b_{n}^{\left(p_{\lambda }^{L},q_{\lambda }^{U}\right)}\right)}_{n\in N}$ are nonpositive and decreasing, where at least one sequence is strictly negative, implying that the sum ${\left(\sum_{k=1}^{n}b_{k}^{\left(p_{\lambda }^{U},q_{\lambda }^{U}\right)}\right)}_{n\in N}$ is strictly negative for n≥2 and strictly decreasing. To see this, suppose that (47) is satisfied with two strict inequalities. Then, ${\left(a_{n}^{\left(q_{\lambda }^{U}\right)}\right)}_{n\in N}$ as well as ${\left(b_{n}^{\left(p_{\lambda }^{L},q_{\lambda }^{U}\right)}\right)}_{n\in N}$ are strictly negative and strictly decreasing. If $q_{\lambda }^{U}=\beta_{\lambda }$ and $p_{\lambda }^{U}<\alpha_{\lambda }$, we see from (P2) and (P6) that $a_{n}^{\left(q_{\lambda }^{U}\right)}\equiv 0$ and that $b_{n}^{\left(p_{\lambda }^{U},q_{\lambda }^{U}\right)}\equiv p_{\lambda }^{U}-\alpha_{\lambda }<0$ (notice that $\alpha_{\lambda }=0$ is not possible in the current setup $P_{\mathrm{SP}}\backslash P_{\mathrm{SP},1}$ and for λ∈]0,1[). In the last case $q_{\lambda }^{U}<\beta_{\lambda }$ and $p_{\lambda }^{U}=\alpha_{\lambda }$, from (P1) and (P5) it follows that ${\left(a_{n}^{\left(q_{\lambda }^{U}\right)}\right)}_{n\in N}$ is strictly negative and strictly decreasing, as well as that $b_{1}^{\left(p_{\lambda }^{U},q_{\lambda }^{U}\right)}=0$ and ${\left(b_{n}^{\left(p_{\lambda }^{L},q_{\lambda }^{U}\right)}\right)}_{n\in N}$ is strictly decreasing and strictly negative for n≥2. Thus, whenever (47) is satisfied, the sum ${\left(\sum_{k=1}^{n}b_{k}^{\left(p_{\lambda }^{U},q_{\lambda }^{U}\right)}\right)}_{n\in N}$ is strictly negative for n≥2 and strictly decreasing.

To achieve Goal (G2), we have to require that the sequence ${\left(a_{n}^{\left(q_{\lambda }^{U}\right)}\right)}_{n\in N}$ converges, which is the case if either $q_{\lambda }^{U}\leq \beta_{\lambda }$ or $\beta_{\lambda }<q_{\lambda }^{U}\leq \min\left\{1,e^{\beta_{\lambda }-1}\right\}$ (cf. Properties 1 (P1) to (P3)). From the upper bound component ${\tilde{B}}_{\lambda ,X_{0},n}^{\left(p_{\lambda }^{U},q_{\lambda }^{U}\right)}$ (42) we conclude that Goal (G2) is met if the sequence ${\left(b_{n}^{\left(p_{\lambda }^{U},q_{\lambda }^{U}\right)}\right)}_{n\in N}$ converges to a negative limit, i.e., $\lim_{n\to \infty }b_{n}^{\left(p_{\lambda }^{U},q_{\lambda }^{U}\right)}=p_{\lambda }^{U}\cdot e^{x_{0}^{\left(q_{\lambda }^{U}\right)}}-\alpha_{\lambda }<0$. Notice that this condition holds true if (47) is satisfied: suppose that $q_{\lambda }^{U}<\beta_{\lambda }$, then $x_{0}^{\left(q_{\lambda }^{U}\right)}<0$ and $p_{\lambda }^{U}\cdot e^{x_{0}^{\left(q_{\lambda }^{U}\right)}}-\alpha_{\lambda }<p_{\lambda }^{U}-\alpha_{\lambda }\leq 0$. On the other hand, if $p_{\lambda }^{U}-\alpha_{\lambda }<0$, one obtains $x_{0}^{\left(q_{\lambda }^{U}\right)}\leq 0$ leading to $p_{\lambda }^{U}\cdot e^{x_{0}^{\left(q_{\lambda }^{U}\right)}}-\alpha_{\lambda }\leq p_{\lambda }^{U}-\alpha_{\lambda }<0$.

The examination of Goal (G2) above enters into the discussion of Goal (G3): if the sequence ${\left(a_{n}^{\left(q_{\lambda }^{U}\right)}\right)}_{n\in N}$ converges and $\lim_{n\to \infty }B_{\lambda ,X_{0},n}^{U}=0$, then there holds

(48) $$\lim_{n\to \infty }\frac{1}{n}\log\left(B_{\lambda ,X_{0},n}^{U}\right)\;=\;\lim_{n\to \infty }\frac{1}{n}\log\left({\tilde{B}}_{\lambda ,X_{0},n}^{\left(p_{\lambda }^{U},q_{\lambda }^{U}\right)}\right)\;=\;p_{\lambda }^{U}\cdot e^{x_{0}^{\left(q_{\lambda }^{U}\right)}}-\alpha_{\lambda }\;.$$

For the case $\left(\beta_{A},\beta_{H},\alpha_{A},\alpha_{H},\lambda \right)\in \left(P_{\mathrm{SP}}\backslash P_{\mathrm{SP},1}\right)\times ]0,1[$, let us now start with our comprehensive investigations of the upper bounds, where we focus on fulfilling the condition (47) which tackles Goals (G1) and (G2) simultaneously; then, the Goal (G3) can be achieved by (48). As indicated above, various different parameter subcases can lead to different Hellinger-integral-upper-bound details, which we work out in the following. For better transparency, we employ the following notations (where the first four are just reminders of sets which were already introduced above)

(49) $$\begin{array}{ccc} P_{\mathrm{NI}} & := & \left\{\;\left(\beta_{A},\beta_{H},\alpha_{A},\alpha_{H}\right)\in {[0,\infty [}^{4}\;:\;\alpha_{A}=\alpha_{H}=0;\;\beta_{A}>0;\;\beta_{H}>0;\;\beta_{A}\neq \beta_{H}\;\right\},\\ P_{\mathrm{SP}} & := & \left\{\;\left(\beta_{A},\beta_{H},\alpha_{A},\alpha_{H}\right)\in \;{]0,\infty [}^{4}\;:\;\left(\alpha_{A}\neq \alpha_{H}\right)\;\;\mathrm{or}\;\;\left(\beta_{A}\neq \beta_{H}\right)\;\;\mathrm{or}\mathrm{both}\;\right\},\\ P & := & P_{\mathrm{NI}}\cup P_{\mathrm{SP}},\\ P_{\mathrm{SP},1} & := & \left\{\;\left(\beta_{A},\beta_{H},\alpha_{A},\alpha_{H}\right)\in P_{\mathrm{SP}}\;:\;\alpha_{A}\neq \alpha_{H},\;\beta_{A}\neq \beta_{H},\;\frac{\alpha_{A}}{\beta_{A}}=\frac{\alpha_{H}}{\beta_{H}}\;\right\},\\ P_{\mathrm{SP},2} & := & \left\{\;\left(\beta_{A},\beta_{H},\alpha_{A},\alpha_{H}\right)\in P_{\mathrm{SP}}\;:\;\alpha_{A}=\alpha_{H},\;\beta_{A}\neq \beta_{H}\;\right\},\\ P_{\mathrm{SP},3} & := & \left\{\;\left(\beta_{A},\beta_{H},\alpha_{A},\alpha_{H}\right)\in P_{\mathrm{SP}}\;:\;\alpha_{A}\neq \alpha_{H},\;\beta_{A}\neq \beta_{H},\;\frac{\alpha_{A}}{\beta_{A}}\neq \frac{\alpha_{H}}{\beta_{H}}\;\right\}\;=\;P_{\mathrm{SP},3a}\cup P_{\mathrm{SP},3b}\cup P_{\mathrm{SP},3c}\;,\\ P_{\mathrm{SP},3a} & := & \left\{\;\left(\beta_{A},\beta_{H},\alpha_{A},\alpha_{H}\right)\in P_{\mathrm{SP}}\;:\;\alpha_{A}\neq \alpha_{H},\;\beta_{A}\neq \beta_{H},\;\frac{\alpha_{A}}{\beta_{A}}\neq \frac{\alpha_{H}}{\beta_{H}},\;\frac{\alpha_{A}-\alpha_{H}}{\beta_{H}-\beta_{A}}\in \;]-\infty ,0[\;\right\},\\ P_{\mathrm{SP},3b} & := & \left\{\;\left(\beta_{A},\beta_{H},\alpha_{A},\alpha_{H}\right)\in P_{\mathrm{SP}}\;:\;\alpha_{A}\neq \alpha_{H},\;\beta_{A}\neq \beta_{H},\;\frac{\alpha_{A}}{\beta_{A}}\neq \frac{\alpha_{H}}{\beta_{H}},\;\frac{\alpha_{A}-\alpha_{H}}{\beta_{H}-\beta_{A}}\in \;]0,\infty [\backslash N\;\right\},\\ P_{\mathrm{SP},3c} & := & \left\{\;\left(\beta_{A},\beta_{H},\alpha_{A},\alpha_{H}\right)\in P_{\mathrm{SP}}\;:\;\alpha_{A}\neq \alpha_{H},\;\beta_{A}\neq \beta_{H},\;\frac{\alpha_{A}}{\beta_{A}}\neq \frac{\alpha_{H}}{\beta_{H}},\;\frac{\alpha_{A}-\alpha_{H}}{\beta_{H}-\beta_{A}}\in N\;\right\},\\ P_{\mathrm{SP},4} & := & \left\{\;\left(\beta_{A},\beta_{H},\alpha_{A},\alpha_{H}\right)\in P_{\mathrm{SP}}\;:\;\alpha_{A}\neq \alpha_{H}>0,\;\beta_{A}=\beta_{H}\;\right\}=P_{\mathrm{SP},4a}\cup P_{\mathrm{SP},4b}\;,\\ P_{\mathrm{SP},4a} & := & \left\{\;\left(\beta_{A},\beta_{H},\alpha_{A},\alpha_{H}\right)\in P_{\mathrm{SP}}\;:\;\alpha_{A}\neq \alpha_{H}>0,\;\beta_{A}=\beta_{H}\in \;]0,1[\;\right\},\\ P_{\mathrm{SP},4b} & := & \left\{\;\left(\beta_{A},\beta_{H},\alpha_{A},\alpha_{H}\right)\in P_{\mathrm{SP}}\;:\;\alpha_{A}\neq \alpha_{H}>0,\;\beta_{A}=\beta_{H}\in \;[1,\infty [\;\right\}; \end{array}$$

notice that because of Lemma A1 and of the Properties 3 (P15) one gets on the domain ]0,∞[ the relation $\phi_{\lambda }\left(x\right)=0$ iff $f_{A}\left(x\right)=f_{H}\left(x\right)$ iff $x=x^{*}:=\frac{\alpha_{H}-\alpha_{A}}{\beta_{A}-\beta_{H}}\in \;]0,\infty [$.

### 3.7. Upper Bounds for the Cases $\left(\beta_{A},\beta_{H},\alpha_{A},\alpha_{H},\lambda \right)\in P_{\mathrm{SP},2}\times ]0,1[$

For this parameter constellation, one has $\phi_{\lambda }\left(0\right)=0$ and $\phi_{\lambda }^{\prime }\left(0\right)=0$ (cf. Properties 3 (P16), (P17)). Thus, the only admissible intercept choice satisfying (47) is $r_{\lambda }^{U}=0=p_{\lambda }^{U}-\alpha_{\lambda }$(i.e., $p_{\lambda }^{U}=p^{U}\left(\beta_{A},\beta_{H},\alpha_{A},\alpha_{H},\lambda \right)=\alpha_{\lambda }=\alpha >0$), and the minimal admissible slope which implies (35) for all $x\in N_{0}$ is given by $s_{\lambda }^{U}=\frac{\phi_{\lambda }\left(1\right)-\phi_{\lambda }\left(0\right)}{1-0}=q_{\lambda }^{U}-\beta_{\lambda }=a_{1}^{\left(q_{\lambda }^{U}\right)}<0$(i.e., $q_{\lambda }^{U}=q^{U}\left(\beta_{A},\beta_{H},\alpha_{A},\alpha_{H},\lambda \right)={\left(\alpha +\beta_{A}\right)}^{\lambda }{\left(\alpha +\beta_{H}\right)}^{1-\lambda }-\alpha >0$). Analogously to the investigation for $P_{\mathrm{SP},1}$ in the above-mentioned Section 3.3, one can derive that ${\left(a_{n}^{\left(q_{\lambda }^{U}\right)}\right)}_{n\in N}$ is strictly negative, strictly decreasing, and converges to $x_{0}^{\left(q_{\lambda }^{U}\right)}\in ]-\beta_{\lambda },q_{\lambda }^{U}-\beta_{\lambda }[$ as indicated in Properties 1 (P1). Moreover, in the same manner as for the case $P_{\mathrm{SP},1}$ this leads to

**Proposition** **7.** *For all $\left(\beta_{A},\beta_{H},\alpha_{A},\alpha_{H},\lambda \right)\in P_{\mathrm{SP},2}\times ]0,1[$ and all initial population sizes $X_{0}\in N$ there holds with $p_{\lambda }^{U}=\alpha ,\;q_{\lambda }^{U}={\left(\alpha +\beta_{A}\right)}^{\lambda }{\left(\alpha +\beta_{H}\right)}^{1-\lambda }-\alpha$*

$$\begin{array}{cc} \left(a\right) & \;B_{\lambda ,X_{0},1}^{U}\;=\;\exp\left\{\left(q_{\lambda }^{U}-\beta_{\lambda }\right)\cdot X_{0}\right\}\;<\;1,\;\;\\ \left(b\right) & \;\mathrm{the}\;\mathrm{sequence}{\left(B_{\lambda ,X_{0},n}^{U}\right)}_{n\in N}\;\mathrm{of}\;\mathrm{upper}\;\mathrm{bounds}\;\mathrm{for}\;H_{\lambda }(P_{A,n}∥P_{H,n})\;\mathrm{given}\;\mathrm{by}\\ & \;B_{\lambda ,X_{0},n}^{U}\;=\;\exp\left\{a_{n}^{\left(q_{\lambda }^{U}\right)}\cdot X_{0}\;+\;\sum_{k=1}^{n}b_{k}^{\left(p_{\lambda }^{U},q_{\lambda }^{U}\right)}\right\}\\ & \;\mathrm{is}\;\mathrm{strictly}\;\mathrm{decreasing},\\ \left(c\right) & \;\lim_{n\to \infty }B_{\lambda ,X_{0},n}^{U}\;=\;0\;=\;\lim_{n\to \infty }H_{\lambda }(P_{A,n}∥P_{H,n})\;,\\ \left(d\right) & \;\lim_{n\to \infty }\;\frac{1}{n}\;\log B_{\lambda ,X_{0},n}^{U}\;=\;p_{\lambda }^{U}\cdot e^{x_{0}^{\left(q_{\lambda }^{U}\right)}}-\alpha_{\lambda }\;=\;\alpha \left(e^{x_{0}^{\left(q_{\lambda }^{U}\right)}}-1\right)\;<\;0\;.\\ \left(e\right) & \;\mathrm{the}\;\mathrm{map}\;\;X_{0}\;\mapsto \;B_{\lambda ,X_{0},n}^{U}\;\mathrm{is}\;\mathrm{strictly}\;\mathrm{decreasing}. \end{array}$$

### 3.8. Upper Bounds for the Cases $\left(\beta_{A},\beta_{H},\alpha_{A},\alpha_{H},\lambda \right)\in P_{\mathrm{SP},3a}\times ]0,1[$

From Properties 3 (P16) one gets $\phi_{\lambda }\left(0\right)<0$, whereas $\phi_{\lambda }^{\prime }\left(0\right)$ can assume any sign, take e.g., the parameters $\left(\beta_{A},\beta_{H},\alpha_{A},\alpha_{H},\lambda \right)=\left(1.8,0.9,2.7,0.7,0.5\right)$ for $\phi_{\lambda }^{\prime }\left(0\right)<0$, $\left(\beta_{A},\beta_{H},\alpha_{A},\alpha_{H},\lambda \right)=\left(1.8,0.9,2.8,0.7,0.5\right)$ for $\phi_{\lambda }^{\prime }\left(0\right)=0$ and $\left(\beta_{A},\beta_{H},\alpha_{A},\alpha_{H},\lambda \right)=\left(1.8,0.9,2.9,0.7,0.5\right)$ for $\phi_{\lambda }^{\prime }\left(0\right)>0$; within our running-example epidemiological context of Section 2.3, this corresponds to a “nearly dangerous” infectious-disease-transmission situation (H) (with nearly critical reproduction number $\beta_{H}=0.9$ and importation mean of $\alpha_{H}=0.7$), whereas (A) describes a “dangerous” situation (with supercritical $\beta_{A}=1.8$ and $\alpha_{A}=2.7,2.8,2.9$). However, in all three subcases there holds $\max_{x\in N_{0}}\phi_{\lambda }\left(x\right)\leq \max_{x\in [0,\infty [}\phi_{\lambda }\left(x\right)<0$. Thus, there clearly exist parameters $p_{\lambda }^{U}=p^{U}\left(\beta_{A},\beta_{H},\alpha_{A},\alpha_{H},\lambda \right),$ $q_{\lambda }^{U}=q^{U}\left(\beta_{A},\beta_{H},\alpha_{A},\alpha_{H},\lambda \right)$ with $p_{\lambda }^{U}\in [\alpha_{A}^{\lambda }\alpha_{H}^{1-\lambda },\alpha_{\lambda }[$ and $q_{\lambda }^{U}\in [\beta_{A}^{\lambda }\beta_{H}^{1-\lambda },\beta_{\lambda }[$ (implying (47)) such that (35) is satisfied. As explained above, we get the following

**Proposition** **8.** *For all $\left(\beta_{A},\beta_{H},\alpha_{A},\alpha_{H},\lambda \right)\in P_{\mathrm{SP},3a}\times ]0,1[$ there exist parameters $p_{\lambda }^{U},\;q_{\lambda }^{U}$ which satisfy $p_{\lambda }^{U}\in [\alpha_{A}^{\lambda }\alpha_{H}^{1-\lambda },\alpha_{\lambda }[$ and $q_{\lambda }^{U}\in [\beta_{A}^{\lambda }\beta_{H}^{1-\lambda },\beta_{\lambda }[$ as well as (35) for all $x\in N_{0}$, and for all such pairs $\left(p_{\lambda }^{U},q_{\lambda }^{U}\right)$ and all initial population sizes $X_{0}\in N$ there holds*

$$\begin{array}{cc} \left(a\right) & \;B_{\lambda ,X_{0},1}^{U}\;=\;\exp\left\{\left(q_{\lambda }^{U}-\beta_{\lambda }\right)\cdot X_{0}\;+\;p_{\lambda }^{U}-\alpha_{\lambda }\right\}\;<\;1,\\ \left(b\right) & \;\mathrm{the}\;\mathrm{sequence}{\left(B_{\lambda ,X_{0},n}^{U}\right)}_{n\in N}\;\mathrm{of}\;\mathrm{upper}\;\mathrm{bounds}\;\mathrm{for}\;H_{\lambda }(P_{A,n}∥P_{H,n})\;\mathrm{given}\;\mathrm{by}\\ & \;B_{\lambda ,X_{0},n}^{U}\;=\;\exp\left\{a_{n}^{\left(q_{\lambda }^{U}\right)}\;X_{0}\;+\;\sum_{k=1}^{n}b_{k}^{\left(p_{\lambda }^{U},q_{\lambda }^{U}\right)}\right\}\\ & \;\mathrm{is}\;\mathrm{strictly}\;\mathrm{decreasing},\\ \left(c\right) & \;\lim_{n\to \infty }B_{\lambda ,X_{0},n}^{U}\;=\;0\;=\;\lim_{n\to \infty }H_{\lambda }(P_{A,n}∥P_{H,n})\;,\\ \left(d\right) & \;\lim_{n\to \infty }\;\frac{1}{n}\;\log B_{\lambda ,X_{0},n}^{U}\;=\;p_{\lambda }^{U}\cdot e^{x_{0}^{\left(q_{\lambda }^{U}\right)}}-\alpha_{\lambda }\;<\;0\;,\\ \left(e\right) & \;\mathrm{the}\;\mathrm{map}\;X_{0}\;\mapsto \;B_{\lambda ,X_{0},n}^{U}\;\mathrm{is}\;\mathrm{strictly}\;\mathrm{decreasing}. \end{array}$$

Notice that all parts of this proposition also hold true for parameter pairs $\left(p_{\lambda }^{U},q_{\lambda }^{U}\right)$ satisfying (35) and additionally either $p_{\lambda }^{U}=\alpha_{\lambda }$, $q_{\lambda }^{U}<\beta_{\lambda }$ or $p_{\lambda }^{U}<\alpha_{\lambda }$, $q_{\lambda }^{U}=\beta_{\lambda }$.

Let us briefly illuminate the above-mentioned possible parameter choices, where we begin with the case of $\phi_{\lambda }^{\prime }\left(0\right)\leq 0$, which corresponds to $\lambda \beta_{A}{\left(\alpha_{A}/\alpha_{H}\right)}^{\lambda -1}+\left(1-\lambda \right)\beta_{H}{\left(\alpha_{A}/\alpha_{H}\right)}^{\lambda }-\beta_{\lambda }\leq 0$ (cf. (P17)); then, the function $\phi_{\lambda }\left(\cdot \right)$ is strictly negative, strictly decreasing, and–due to (P19)–strictly concave (and thus, the assumption $\frac{\alpha_{H}-\alpha_{A}}{\beta_{A}-\beta_{H}}<0$ is superfluous here). One pragmatic but yet reasonable parameter choice is the following: take any intercept $p_{\lambda }^{U}\in \left[\alpha_{A}^{\lambda }\alpha_{H}^{1-\lambda },\alpha_{\lambda }\right]$ such that $\left(p_{\lambda }^{U}-\alpha_{\lambda }\right)+2\left(\phi_{\lambda }\left(1\right)-\left(p_{\lambda }^{U}-\alpha_{\lambda }\right)\right)\geq \phi_{\lambda }\left(2\right)$ (i.e., $2{\left(\alpha_{A}+\beta_{A}\right)}^{\lambda }\;{\left(\alpha_{H}+\beta_{H}\right)}^{1-\lambda }-p_{\lambda }^{U}+\alpha_{\lambda }\geq {\left(\alpha_{A}+2\beta_{A}\right)}^{\lambda }\;{\left(\alpha_{H}+2\beta_{H}\right)}^{1-\lambda }$) and $q_{\lambda }^{U}:=\phi_{\lambda }\left(1\right)-\left(p_{\lambda }^{U}-\alpha_{\lambda }\right)+\beta_{\lambda }={\left(\alpha_{A}+\beta_{A}\right)}^{\lambda }\;{\left(\alpha_{H}+\beta_{H}\right)}^{1-\lambda }-p_{\lambda }^{U}$, which corresponds to a linear function $\phi_{\lambda }^{U}$ which is (i) nonpositive on $N_{0}$ and strictly negative on N, and (ii) larger than or equal to $\phi_{\lambda }$ on $N_{0}$, strictly larger than $\phi_{\lambda }$ on N\{1,2}, and equal to $\phi_{\lambda }$ at the point x=1 (“discrete tangent or secant line through x=1”). One can easily see that (due to the restriction (34)) not all $p_{\lambda }^{U}\in \left[\alpha_{A}^{\lambda }\alpha_{H}^{1-\lambda },\alpha_{\lambda }\right]$ might qualify for the current purpose. For the particular choice $p_{\lambda }^{U}=\alpha_{A}^{\lambda }\alpha_{H}^{1-\lambda }$ and $q_{\lambda }^{U}={\left(\alpha_{A}+\beta_{A}\right)}^{\lambda }\;{\left(\alpha_{H}+\beta_{H}\right)}^{1-\lambda }-\alpha_{A}^{\lambda }\alpha_{H}^{1-\lambda }$ one obtains $r_{\lambda }^{U}=p_{\lambda }^{U}-\alpha_{\lambda }=b_{1}^{\left(p_{\lambda }^{U},q_{\lambda }^{U}\right)}<0$ (cf. Lemma A1) and $s_{\lambda }^{U}=q_{\lambda }^{U}-\beta_{\lambda }=\phi_{\lambda }\left(1\right)-\phi_{\lambda }\left(0\right)=a_{1}^{\left(q_{\lambda }^{U}\right)}<0$ (secant line through $\phi_{\lambda }\left(0\right)$ and $\phi_{\lambda }\left(1\right)$).

For the remaining case $\phi_{\lambda }^{\prime }\left(0\right)>0$, which corresponds to $\lambda \beta_{A}{\left(\alpha_{A}/\alpha_{H}\right)}^{\lambda -1}+\left(1-\lambda \right)\beta_{H}{\left(\alpha_{A}/\alpha_{H}\right)}^{\lambda }-\beta_{\lambda }>0$, the function $\phi_{\lambda }\left(\cdot \right)$ is strictly negative, strictly concave and hump-shaped (cf. (P18)). For the derivation of the parameter choices, we employ $x_{\max}:={\mathrm{argmax}}_{x\in ]0,\infty [}\phi_{\lambda }\left(x\right)$ which is the unique solution of

(50) $$\lambda \beta_{A}\left[{\left(\frac{f_{A}\left(x\right)}{f_{H}\left(x\right)}\right)}^{\lambda -1}-1\right]\;+\;\left(1-\lambda \right)\beta_{H}\left[{\left(\frac{f_{A}\left(x\right)}{f_{H}\left(x\right)}\right)}^{\lambda }-1\right]\;=\;0\;,\;x\in ]0,\infty [\;,$$

(cf. (P17), (P19)); notice that $x=x^{*}:=\frac{\alpha_{H}-\alpha_{A}}{\beta_{A}-\beta_{H}}\in \;]0,\infty [$ formally satisfies the Equation (50) but does not qualify because of the current restriction $x^{*}<0$.

Let us first inspect the case $\phi_{\lambda }\left(\left\lfloor x_{\max}\right\rfloor \right)>\phi_{\lambda }\left(\left\lfloor x_{\max}\right\rfloor +1\right)$, where ⌊x⌋ denotes the integer part of *x*. Consider the subcase $\phi_{\lambda }\left(\left\lfloor x_{\max}\right\rfloor \right)+\left\lfloor x_{\max}\right\rfloor \left(\phi_{\lambda }\left(\left\lfloor x_{\max}\right\rfloor \right)-\phi_{\lambda }\left(\left\lfloor x_{\max}\right\rfloor +1\right)\right)\leq 0$, which means that the secant line through $\phi_{\lambda }\left(\left\lfloor x_{\max}\right\rfloor \right)$ and $\phi_{\lambda }\left(\left\lfloor x_{\max}\right\rfloor +1\right)$ possesses a non-positive intercept. In this situation it is reasonable to choose as *intercept* any $p_{\lambda }^{U}-\alpha_{\lambda }=b_{1}^{\left(p_{\lambda }^{U},q_{\lambda }^{U}\right)}=r_{\lambda }^{U}\in \left[\phi_{\lambda }\left(\left\lfloor x_{\max}\right\rfloor \right),\phi_{\lambda }\left(\left\lfloor x_{\max}\right\rfloor \right)+\left\lfloor x_{\max}\right\rfloor \left(\phi_{\lambda }\left(\left\lfloor x_{\max}\right\rfloor \right)-\phi_{\lambda }\left(\left\lfloor x_{\max}\right\rfloor +1\right)\right)\right]$, and as corresponding *slope* $q_{\lambda }^{U}-\alpha_{\lambda }=a_{1}^{\left(q_{\lambda }^{U}\right)}=s_{\lambda }^{U}=\frac{\phi_{\lambda }\left(\left\lfloor x_{\max}\right\rfloor \right)-r_{\lambda }^{U}}{(\left\lfloor x_{\max}\right\rfloor )-0}\;\leq 0$. A larger intercept would lead to a linear function $\phi_{\lambda }^{U}$ for which (35) is not valid at $\lfloor x_{\max}\rfloor +1$. In the other subcase $\phi_{\lambda }\left(\left\lfloor x_{\max}\right\rfloor \right)+x_{\max}\left(\phi_{\lambda }\left(\left\lfloor x_{\max}\right\rfloor \right)-\phi_{\lambda }\left(\left\lfloor x_{\max}\right\rfloor +1\right)\right)>0$, one can choose any intercept $p_{\lambda }^{U}-\alpha_{\lambda }=b_{1}^{\left(p_{\lambda }^{U},q_{\lambda }^{U}\right)}=r_{\lambda }^{U}\in \left[\phi_{\lambda }\left(\left\lfloor x_{\max}\right\rfloor \right),0\right]$ and as corresponding slope $q_{\lambda }^{U}-\alpha_{\lambda }=a_{1}^{\left(q_{\lambda }^{U}\right)}=s_{\lambda }^{U}=\frac{\phi_{\lambda }\left(\left\lfloor x_{\max}\right\rfloor \right)-r_{\lambda }^{U}}{(\left\lfloor x_{\max}\right\rfloor )-0}\;\leq 0$ (notice that the corresponding line $\phi_{\lambda }^{U}$ is on $]\left\lfloor x_{\max}\right\rfloor ,\infty [$ strictly larger than the secant line through $\phi_{\lambda }\left(\left\lfloor x_{\max}\right\rfloor \right)$ and $\phi_{\lambda }\left(\left\lfloor x_{\max}\right\rfloor +1\right)$).

If $\phi_{\lambda }\left(\left\lfloor x_{\max}\right\rfloor \right)\leq \phi_{\lambda }\left(\left\lfloor x_{\max}\right\rfloor +1\right)$, one can proceed as above by substituting the crucial pair of points $\left(\left\lfloor x_{\max}\right\rfloor ,\left\lfloor x_{\max}\right\rfloor +1\right)$ with $\left(\left\lfloor x_{\max}\right\rfloor +1,\left\lfloor x_{\max}\right\rfloor +2\right)$ and examining the analogous two subcases.

### 3.9. Upper Bounds for the Cases $\left(\beta_{A},\beta_{H},\alpha_{A},\alpha_{H},\lambda \right)\in P_{\mathrm{SP},3b}\times ]0,1[$

The only difference to the preceding Section 3.8 is that–due to Properties 3 (P15)–the maximum value of $\phi_{\lambda }\left(\cdot \right)$ now achieves 0, at the positive *non-integer* point $x_{\max}=x^{*}=\frac{\alpha_{H}-\alpha_{A}}{\beta_{A}-\beta_{H}}\in ]0,\infty [\backslash N$ (take e.g., $\left(\beta_{A},\beta_{H},\alpha_{A},\alpha_{H},\lambda \right)=\left(1.8,0.9,1.1,3.0,0.5\right)$ as an example, which within our running-example epidemiological context of Section 2.3 corresponds to a “nearly dangerous” infectious-disease-transmission situation (H) (with nearly critical reproduction number $\beta_{H}=0.9$ and importation mean of $\alpha_{H}=3$), whereas (A) describes a “dangerous” situation (with supercritical $\beta_{A}=1.8$ and $\alpha_{A}=1.1$)); this implies that $\phi_{\lambda }\left(x\right)<0$ for all *x* on the relevant subdomain $N_{0}$. Due to (P16), (P17) and (P19) one gets automatically $\lambda \beta_{A}{\left(\alpha_{A}/\alpha_{H}\right)}^{\lambda -1}+\left(1-\lambda \right)\beta_{H}{\left(\alpha_{A}/\alpha_{H}\right)}^{\lambda }-\beta_{\lambda }>0$ for all λ∈]0,1[. Analogously to Section 3.8, there exist parameter $p_{\lambda }^{U}\in \left[\alpha_{A}^{\lambda }\alpha_{H}^{1-\lambda },\alpha_{\lambda }\right]$ and $q_{\lambda }^{U}\in \left[\beta_{A}^{\lambda }\beta_{H}^{1-\lambda },\beta_{\lambda }\right]$ such that (47) and (35) are satisfied. Thus, all the assertions (a) to (e) of Proposition 8 also hold true for the current parameter constellations.

### 3.10. Upper Bounds for the Cases $\left(\beta_{A},\beta_{H},\alpha_{A},\alpha_{H},\lambda \right)\in P_{\mathrm{SP},3c}\times ]0,1[$

The only difference to the preceding Section 3.9 is that the maximum value of $\phi_{\lambda }\left(\cdot \right)$ now achieves 0 at the *integer* point $x_{\max}=x^{*}=\frac{\alpha_{H}-\alpha_{A}}{\beta_{A}-\beta_{H}}\in N$ (take e.g., $\left(\beta_{A},\beta_{H},\alpha_{A},\alpha_{H},\lambda \right)=\left(1.8,0.9,1.2,3.0,0.5\right)$ as an example). Accordingly, there do not exist parameters $p_{\lambda }^{U},q_{\lambda }^{U}$, such that (35) and (47) are satisfied simultaneously. The only parameter pair that ensures $\exp\left\{a_{n}^{\left(q_{\lambda }^{U}\right)}\cdot X_{0}\;+\;\sum_{k=1}^{n}b_{k}^{\left(p_{\lambda }^{U},q_{\lambda }^{U}\right)}\right\}\leq 1$ for all n∈N and all $X_{0}\in N$ without further investigations, leads to the choices $p_{\lambda }^{U}=\alpha_{\lambda }$ as well as $q_{\lambda }^{U}=\beta_{\lambda }$. Consequently, $B_{\lambda ,X_{0},n}^{U}\equiv 1$, which coincides with the general upper bound (9), but violates the above-mentioned desired Goal (G1). However, there might exist parameters $p_{\lambda }^{U}<\alpha_{\lambda },\;q_{\lambda }^{U}>\beta_{\lambda }$ or $p_{\lambda }^{U}>\alpha_{\lambda },\;q_{\lambda }^{U}<\beta_{\lambda }$, such that at least the parts (c) and (d) of Proposition 8 are satisfied. Nevertheless, by using a conceptually different method we can prove

(51) $$H_{\lambda }(P_{A,n}∥P_{H,n})\;<\;1\;\forall \;n\in N\backslash \left\{1\right\}\;\mathrm{as}\;\mathrm{well}\;\mathrm{as}\;\mathrm{the}\;\mathrm{convergence}\;\lim_{n\to \infty }\;H_{\lambda }(P_{A,n}∥P_{H,n})\;=\;0$$

which will be used for the study of complete asymptotical distinguishability (entire separation) below. This proof is provided in Appendix A.1.

### 3.11. Upper Bounds for the Cases $\left(\beta_{A},\beta_{H},\alpha_{A},\alpha_{H},\lambda \right)\in P_{\mathrm{SP},4a}\times ]0,1[$

This setup and the remaining setup $\left(\beta_{A},\beta_{H},\alpha_{A},\alpha_{H},\lambda \right)\in P_{\mathrm{SP},4b}\times ]0,1[$ (see the next Section 3.12) are the only constellations where $\phi_{\lambda }\left(\cdot \right)$ is strictly negative and strictly increasing, with $\lim_{x\to \infty }\phi_{\lambda }\left(x\right)=\lim_{x\to \infty }\phi_{\lambda }^{\prime }\left(x\right)=0$, leading to the choices $p_{\lambda }^{U}=\alpha_{\lambda }$ as well as $q_{\lambda }^{U}=\beta_{\lambda }=\beta$ under the restriction that $\exp\left\{a_{n}^{\left(q_{\lambda }^{U}\right)}\cdot X_{0}\;+\;\sum_{k=1}^{n}b_{k}^{\left(p_{\lambda }^{U},q_{\lambda }^{U}\right)}\right\}\leq 1$ for all n∈N and all $X_{0}\in N$. Consequently, one has $B_{\lambda ,X_{0},n}^{U}\equiv 1$, which is consistent with the general upper bound (9) but violates the above-mentioned desired Goal (G1). Unfortunately, the proof method of (51) (cf. Appendix A.1) can’t be carried over to the current setup. The following proposition states two of the above-mentioned desired assertions which can be verified by a completely different proof method, which is also given in Appendix A.1.

**Proposition** **9.** *For all $\left(\beta_{A},\beta_{H},\alpha_{A},\alpha_{H},\lambda \right)\in P_{\mathrm{SP},4a}\times ]0,1[$ there exist parameters $p_{\lambda }^{U}<\alpha_{\lambda }$, $1>q_{\lambda }^{U}>\beta_{\lambda }=\beta$ such that (35) is satisfied for all x∈[0,∞[ and such that for all initial population sizes $X_{0}\in N$ the parts (c) and (d) of Proposition 8 hold true.*

### 3.12. Upper Bounds for the Cases $\left(\beta_{A},\beta_{H},\alpha_{A},\alpha_{H},\lambda \right)\in P_{\mathrm{SP},4b}\times ]0,1[$

The assertions preceding Proposition 9 remain valid. However, any linear upper bound of the function $\phi_{\lambda }\left(\cdot \right)$ on the domain $N_{0}$ possesses the slope $q_{\lambda }^{U}-\beta_{\lambda }\geq 0$. If $q_{\lambda }^{U}=\beta_{\lambda }$, then the intercept is $p_{\lambda }^{U}-\alpha_{\lambda }=0$ leading to $B_{\lambda ,X_{0},n}^{U}\equiv 1$ and thus Goal (G1) is violated. If we use a slope $q_{\lambda }^{U}-\beta_{\lambda }>0$, then both the sequences ${\left(a_{n}^{\left(q_{\lambda }^{U}\right)}\right)}_{n\in N}$ and ${\left(b_{n}^{\left(p_{\lambda }^{U},q_{\lambda }^{U}\right)}\right)}_{n\in N}$ are strictly increasing and diverge to *∞*. This comes from Properties 1 (P3b) and (P7b) since $q_{\lambda }^{U}>\beta_{\lambda }=\beta \geq 1$. Altogether, this implies that the corresponding upper bound component ${\tilde{B}}_{\lambda ,X_{0},n}^{\left(p_{\lambda }^{U},q_{\lambda }^{U}\right)}$ (cf. (42)) diverges to *∞* as well. This leads to

**Proposition** **10.** *For all $\left(\beta_{A},\beta_{H},\alpha_{A},\alpha_{H},\lambda \right)\in P_{\mathrm{SP},4b}\times ]0,1[$ and all initial population sizes $X_{0}\in N$ there do not exist parameters $p_{\lambda }^{U}\geq 0$, $q_{\lambda }^{U}\geq 0$ such that (35) is satisfied and such that the parts (c) and (d) of Proposition 8 hold true.*

### 3.13. Concluding Remarks on Alternative Upper Bounds for all Cases $\left(\beta_{A},\beta_{H},\alpha_{A},\alpha_{H},\lambda \right)\in$ $\left(P_{\mathrm{SP}}\backslash P_{\mathrm{SP},1}\right)\times ]0,1[$

As mentioned earlier on, starting from Section 3.6 we have principally focused on constructing upper bounds $B_{\lambda ,X_{0},n}^{U}$ of the Hellinger integrals, starting from $p_{\lambda }^{U},q_{\lambda }^{U}$ which fulfill (35) as well as further constraints depending on the Goals (G1) and (G2). For the setups in the Section 3.7, Section 3.8 and Section 3.9, we have proved the existence of *special parameter choices* $p_{\lambda }^{U},q_{\lambda }^{U}$ which were consistent with (G1) and (G2). Furthermore, for the constellation in the Section 3.11 we have found parameters such that at least (G2) is satisfied. In contrast, for the setup of Section 3.12 we have not found any choices which are consistent with (G1) and (G2), leading to the “cut-off bound” $B_{\lambda ,X_{0},n}^{U}\equiv 1$ which gives no improvement over the generally valid upper bound (9).

In the following, we present some *alternative choices* of $p_{\lambda }^{U},q_{\lambda }^{U}$ which–depending on the parameter constellation $\left(\beta_{A},\beta_{H},\alpha_{A},\alpha_{H},\lambda \right)\in \left(P_{\mathrm{SP}}\backslash P_{\mathrm{SP},1}\right)\times ]0,1[$–may or may not lead to upper bounds $B_{\lambda ,X_{0},n}^{U}$ which are consistent with Goal (G1) or with (G2) (and which are maybe weaker or better than resp. incomparable with the previous upper bounds when dealing with some relaxations of (G1), such as e.g., $H_{\lambda }(P_{A,n}∥P_{H,n})<1$ for all but finitely many n∈N).

As a first alternative choice for a linear upper bound of $\phi_{\lambda }\left(\cdot \right)$ (cf. (35)) one could use the asymptote $\tilde{\phi_{\lambda }}\left(\cdot \right)$ (cf. Properties 3 (P20)) with the parameters $p_{\lambda }^{U}:=\tilde{p_{\lambda }}=\lambda \alpha_{A}{\left(\beta_{A}/\beta_{H}\right)}^{\lambda -1}+\left(1-\lambda \right)\alpha_{H}{\left(\beta_{A}/\beta_{H}\right)}^{\lambda }$ and $q_{\lambda }^{U}:=\tilde{q_{\lambda }}=\beta_{A}^{\lambda }\beta_{H}^{1-\lambda }$. Another important linear upper bound of $\phi_{\lambda }\left(\cdot \right)$ is the tangent line $\phi_{\lambda ,y}^{\tan}\left(\cdot \right)$ on $\phi_{\lambda }\left(\cdot \right)$ at an arbitrarily fixed point y∈[0,∞[, which amounts to

(52) $$\phi_{\lambda ,y}^{\tan}\left(x\right)\;:=\;r_{\lambda ,y}^{\tan}+s_{\lambda ,y}^{\tan}\cdot x\;:=\;\left(p_{\lambda ,y}^{\tan}-\alpha_{\lambda }\right)+\left(q_{\lambda ,y}^{\tan}-\beta_{\lambda }\right)\cdot x\;:=\;\left(\phi_{\lambda }\left(y\right)-y\cdot \phi_{\lambda }^{\prime }\left(y\right)\right)\;+\;\phi_{\lambda }^{\prime }\left(y\right)\cdot x\;,$$

where $\phi_{\lambda }^{\prime }\left(\cdot \right)$ is given by (P17). Notice that this upper bound is for y∈]0,∞[\N “not tight” in the sense that $\phi_{\lambda ,y}^{\tan}\left(\cdot \right)$ does not hit the function $\phi_{\lambda }\left(\cdot \right)$ on $N_{0}$ (where the generation sizes “live”); moreover, $\phi_{\lambda ,y}^{\tan}\left(x\right)$ might take on strictly positive values for large enough points *x* which is counter-productive for Goal (G1). Another alternative choice of a linear upper bound for $\phi_{\lambda }\left(\cdot \right)$, which in contrast to the tangent line is “tight” (but not necessarily avoiding the strict positivity), is the secant line $\phi_{\lambda ,k}^{\sec}\left(\cdot \right)$ across its arguments *k* and k+1, given by

(53) $$\begin{array}{ccc} \phi_{\lambda ,k}^{\sec}\left(x\right) & := & r_{\lambda ,k}^{\sec}+s_{\lambda ,k}^{\sec}\cdot x\;:=\;\left(p_{\lambda ,k}^{\sec}-\alpha_{\lambda }\right)+\left(q_{\lambda ,k}^{\sec}-\beta_{\lambda }\right)\cdot x\\ & := & \left[\phi_{\lambda }\left(k\right)-k\cdot \left(\phi_{\lambda }\left(k+1\right)-\phi_{\lambda }\left(k\right)\right)\right]\;+\;\left(\phi_{\lambda }\left(k+1\right)-\phi_{\lambda }\left(k\right)\right)\cdot x\;. \end{array}$$

Another alternative choice is the horizontal line

(54) $$\phi_{\lambda }^{\mathrm{hor}}\left(x\right)\;\equiv \;\max\left\{\phi_{\lambda }\left(y\right),\;y\in N_{0}\right\}\;.$$

For $p_{\lambda }^{U}\in \left\{\tilde{p_{\lambda }}\;,\;p_{\lambda ,y}^{\tan}\;,\;p_{\lambda ,y}^{\sec}\right\}$ and $q_{\lambda }^{U}\in \left\{q_{\lambda ,y}^{\tan}\;,\;q_{\lambda ,y}^{\sec}\right\}$ it is possible that in some parameter cases $\left(\beta_{A},\beta_{H},\alpha_{A},\alpha_{H}\right)$ either the intercept $r_{\lambda }^{U}=p_{\lambda }^{U}-\alpha_{\lambda }$ is strictly larger than zero or the slope $s_{\lambda }^{U}=q_{\lambda }^{U}-\beta_{\lambda }$ is strictly larger than zero. Thus, it can happen that ${\tilde{B}}_{\lambda ,X_{0},n}^{\left(p_{\lambda }^{U},q_{\lambda }^{U}\right)}>1$ for some (and even for all) n∈N, such that the corresponding upper bound $B_{\lambda ,X_{0},n}^{U}$ for the Hellinger integral $H_{\lambda }(P_{A,n}∥P_{H,n})$ amounts to the cut-off at 1. However, due to Properties 1 (P5) and (P7a), the sequence ${\left({\tilde{B}}_{\lambda ,X_{0},n}^{\left(p_{\lambda }^{U},q_{\lambda }^{U}\right)}\right)}_{n\in N}$ may become smaller than 1 and may finally converge to zero. Due to Properties 2 (P14), this upper bound can even be tighter (smaller) than those bounds derived from parameters $p_{\lambda }^{U},\;q_{\lambda }^{U}$ fulfilling (47).

As far as our desired Hellinger integral bounds are concerned, in the setup of Section 3.11—where $\lim_{y\to \infty }\phi_{\lambda ,y}^{\tan}\left(\cdot \right)\equiv 0$–for the proof of Proposition 9 in Appendix A.1 we shall employ the mappings $y\mapsto \phi_{\lambda ,y}^{\tan}$ resp. $y\mapsto p_{\lambda ,y}^{\tan}$ resp. $y\mapsto q_{\lambda ,y}^{\tan}$. These will also be used for the proof of the below-mentioned Theorem 4.

### 3.14. Intermezzo 1: Application to Asymptotical Distinguishability

The above-mentioned investigations can be applied to the context of Section 2.6 on asymptotical distinguishability. Indeed, with the help of the Definitions 1 and 2 as well as the equivalence relations (25) and (26) we obtain the following

**Corollary** **1.**

*(a)*  *For all $\left(\beta_{A},\beta_{H},\alpha_{A},\alpha_{H}\right)\in P_{\mathrm{SP}}\backslash P_{\mathrm{SP},4b}$ and all initial population sizes $X_{0}\in N$, the corresponding sequences ${\left(P_{A,n}\right)}_{n\in N_{0}}$ and ${\left(P_{H,n}\right)}_{n\in N_{0}}$ are entirely separated (completely asymptotically distinguishable).*
*(b)*  *For all $\left(\beta_{A},\beta_{H},\alpha_{A},\alpha_{H}\right)\in P_{\mathrm{NI}}$ with $\beta_{A}\leq 1$ and all initial population sizes $X_{0}\in N$, the sequence ${\left(P_{A,n}\right)}_{n\in N_{0}}$ is contiguous to ${\left(P_{H,n}\right)}_{n\in N_{0}}$.*
*(c)*  *For all $\left(\beta_{A},\beta_{H},\alpha_{A},\alpha_{H}\right)\in P_{\mathrm{NI}}$ with $\beta_{A}>1$ and all initial population sizes $X_{0}\in N$, the sequence ${\left(P_{A,n}\right)}_{n\in N_{0}}$ is neither contiguous to nor entirely separated to ${\left(P_{H,n}\right)}_{n\in N_{0}}$.*

The proof of Corollary 1 will be given in Appendix A.1.

**Remark** **3.**

*(a)*  *Assertion (c) of Corollary 1 contrasts the case of Gaussian processes with independent increments where one gets either entire separation or mutual contiguity (see e.g., Liese & Vajda [1]).*
*(b)*  *By putting Corollary 1(b) and (c) together, we obtain for different “criticality pairs” in the non-immigration case $P_{\mathrm{NI}}$ the following asymptotical distinguishability types:* *$\left(P_{A,n}\right)◃▹\left(P_{H,n}\right)$ if $\beta_{A}\leq 1$, $\beta_{H}\leq 1$; $\left(P_{A,n}\right)◃\bar{▹}\;\left(P_{H,n}\right)$ if $\beta_{A}\leq 1$, $\beta_{H}>1$;* *$\left(P_{A,n}\right)\;\bar{◃}▹\left(P_{H,n}\right)$ if $\beta_{A}>1$, $\beta_{H}\leq 1$; $\left(P_{A,n}\right)\;\bar{◃}\;\bar{▹}\;\left(P_{H,n}\right)$ and $\left(P_{A,n}\right)\bar{△}\left(P_{H,n}\right)$ if $\beta_{A}>1$, $\beta_{H}>1$;* *in particular, for $P_{\mathrm{NI}}$ the sequences ${\left(P_{A,n}\right)}_{n\in N_{0}}$ and ${\left(P_{H,n}\right)}_{n\in N_{0}}$ are not completely asymptotically inseparable (indistinguishable).*
*(c)*  *In the light of the above-mentioned characterizations of contiguity resp. entire separation by means of Hellinger integral limits, the finite-time-horizon results on Hellinger integrals given in the “λ∈]0,1[ parts” of Theorem 1, the Section 3.3, Section 3.4, Section 3.5, Section 3.6, Section 3.7, Section 3.8, Section 3.9, Section 3.10, Section 3.11, Section 3.12, Section 3.13 and also in the below-mentioned Section 6 can loosely be interpreted as “finite-sample (rather than asymptotical) distinguishability” assertions.*

### 3.15. Intermezzo 2: Application to Decision Making under Uncertainty

#### 3.15.1. Bayesian Decision Making

The above-mentioned investigations can be applied to the context of Section 2.5 on *dichotomous* Bayesian decision making on the space of all possible path scenarios (path space) of Poissonian Galton-Watson processes without/with immigration GW(I) (e.g., in combination with our running-example epidemiological context of Section 2.3). More detailed, for the minimal mean decision loss (Bayes risk) $R_{n}$ defined by (18) we can derive upper (respectively lower) bounds by using (19) respectively (20) together with the exact values or the upper (respectively lower) bounds of the Hellinger integrals $H_{\lambda }(P_{A,n}∥P_{H,n})$ derived in the “λ∈]0,1[ parts” of Theorem 1, the Section 3.3, Section 3.4, Section 3.5, Section 3.6, Section 3.7, Section 3.8, Section 3.9, Section 3.10, Section 3.11, Section 3.12, Section 3.13 (and also in the below-mentioned Section 6); instead of providing the corresponding outcoming formulas–which is merely repetitive–we give the illustrative

**Example** **1.** *Based on a sample path observation $X_{n}:=\left\{X_{\ell }:\;\ell =1,...,n\right\}$ of a GWI, which is either governed by a hypothesis law $P_{H}$ or an alternative law $P_{A}$, we want to make a dichotomous optimal Bayesian decision described in Section 2.5, namely, decide between an action $d_{H}$ “associated with” $P_{H}$ and an action $d_{A}$ “associated with” $P_{A}$, with pregiven loss function (16) involving constants $L_{A}>0$, $L_{H}>0$ which e.g., arise as bounds from quantities in worst-case scenarios.*

*For this, let us exemplarily deal with initial population $X_{0}=5$ as well as parameter setup $\left(\beta_{A},\beta_{H},\alpha_{A},\alpha_{H}\right)=\left(1.2,0.9,4,3\right)\in P_{\mathrm{SP},1}$; within our running-example epidemiological context of Section 2.3, this corresponds e.g., to a setup where one is encountered with a novel infectious disease (such as COVID-19) of non-negligible fatality rate, and (A) reflects a “potentially dangerous” infectious-disease-transmission situation (with supercritical reproduction number $\beta_{A}=1.2$ and importation mean of $\alpha_{A}=4$, for weekly appearing new incidence-generations) whereas (H) describes a “milder” situation (with subcritical $\beta_{H}=0.9$ and $\alpha_{H}=3$). Moreover, let $d_{H}$ and $d_{A}$ reflect two possible sets of interventions (control measures) in the course of pandemic risk management, with respective “worst-case type” decision losses $L_{A}=600$ and $L_{H}=300$ (e.g., in units of billion Euros or U.S. Dollars). Additionally we assume the prior probabilities π=Pr(H)=1−Pr(A)=0.5, which results in the prior-loss constants $L_{A}=300$ and $L_{H}=150$. In order to obtain bounds for the corresponding minimal mean decision loss (Bayes Risk) $R_{n}$ defined in (18) we can employ the general Stummer-Vajda bounds (cf. [15]) (19) and (20) in terms of the Hellinger integral $H_{\lambda }(P_{A,n}∥P_{H,n})$ (with arbitrary λ∈]0,1[), and combine this with the appropriate detailed results on the latter from the preceding subsections. To demonstrate this, let us choose λ=0.5 (for which $H_{1/2}(P_{A,n}∥P_{H,n})$ can be interpreted as a multiple of the Bhattacharyya coefficient between the two competing GWI) respectively λ=0.9, leading to the parameters $p_{0.5}^{E}=3.464,\;q_{0.5}^{E}=1.039$ respectively $p_{0.9}^{E}=3.887$, $q_{0.9}^{E}=1.166$ (cf. (33)). Combining (19) and (20) with Theorem 1 (a)– which provides us with the exact recursive values of $H_{\lambda }(P_{A,n}∥P_{H,n})$ in terms of the sequence $a_{n}^{\left(q_{\lambda }^{E}\right)}$ (cf. (36))– we obtain for λ=0.5 the bounds*

$$\begin{array}{ccc} R_{n} & \leq & R_{n}^{U}\;:=\;2.121\cdot 10^{2}\cdot \exp\left\{5\cdot a_{n}^{\left(1.039\right)}+\;\frac{10}{3}\cdot \sum_{k=1}^{n}a_{k}^{\left(1.039\right)}\right\},\\ R_{n} & \geq & R_{n}^{L}\;:=\;100\cdot \exp\left\{10\cdot a_{n}^{\left(1.039\right)}+\;\frac{20}{3}\cdot \sum_{k=1}^{n}a_{k}^{\left(1.039\right)}\right\}, \end{array}$$

*whereas for λ=0.9 we get*

$$\begin{array}{ccc} R_{n} & \leq & R_{n}^{U}\;:=\;2.799\cdot 10^{2}\cdot \exp\left\{5\cdot a_{n}^{\left(1.166\right)}+\frac{10}{3}\cdot \sum_{k=1}^{n}a_{k}^{\left(1.166\right)}\right\},\\ R_{n} & \geq & R_{n}^{L}\;:=\;3.902\cdot \exp\left\{50\cdot a_{n}^{\left(1.166\right)}+\frac{100}{3}\cdot \sum_{k=1}^{n}a_{k}^{\left(1.166\right)}\right\}. \end{array}$$

**Figure 1:**
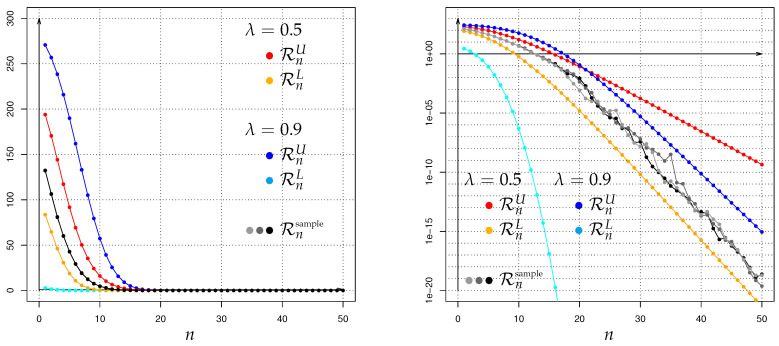
Bayes risk bounds (using λ=0.5 (red/orange) resp. λ=0.9 (blue/cyan)) and Bayes risk simulations (lightgrey/grey/black) on a unit (**left graph**) and logarithmic (**right graph**) scale in the parameter setup $\left(\beta_{A},\beta_{H},\alpha_{A},\alpha_{H}\right)=\left(1.2,0.9,4,3\right)\in P_{\mathrm{SP},1}$, with initial population $X_{0}=5$ and prior-loss constants $L_{A}=300$ and $L_{H}=150$.

*Figure 1 illustrates the lower (orange resp. cyan) and upper (red resp. blue) bounds $R_{n}^{L}$ resp. $R_{n}^{U}$ of the Bayes Risk $R_{n}$ employing λ=0.5 resp. λ=0.9 on both a unit scale (left graph) and a logarithmic scale (right graph). The lightgrey/grey/black curves correspond to the (18)-based empirical evaluation of the Bayes risk sequence ${\left(R_{n}^{\mathrm{sample}}\right)}_{n=1,...,50}$ from three independent Monte Carlo simulations of 10000 GWI sample paths (each) up to time horizon 50.*

#### 3.15.2. Neyman-Pearson Testing

By combining (23) with the exact values resp. upper bounds of the Hellinger integrals $H_{\lambda }\left(P_{A,n}∥P_{H,n}\right)$ from the preceding subsections, we obtain for our context of GW(I) with Poisson offspring and Poisson immigration (including the non-immigration case) some upper bounds of the *minimal* type II error probability $E_{\varsigma }\left(P_{A,n}∥P_{H,n}\right)$ in the class of the tests for which the type I error probability is at most ς∈]0,1[, which can also be immediately rewritten as lower bounds for the power $1-E_{\varsigma }\left(P_{A,n}∥P_{H,n}\right)$ of a most powerful test at level ς. As for the Bayesian context of Section 3.15.1, instead of providing the–merely repetitive–outcoming formulas for the bounds of $E_{\varsigma }\left(P_{A,n}∥P_{H,n}\right)$ we give the illustrative

**Figure 2:**
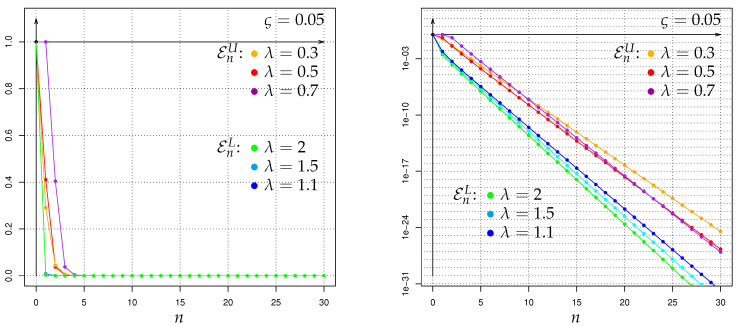
Different lower bounds $E_{n}^{L}$(using λ∈{1.1,1.5,2}) and upper bounds $E_{n}^{U}$(using λ∈{0.3,0.5,0.7}) of the minimal type II error probability $E_{\varsigma }\left(P_{A,n}∥P_{H,n}\right)$ for fixed level ς=0.05 in the parameter setup $\left(\beta_{A},\beta_{H},\alpha_{A},\alpha_{H}\right)=\left(0.3,1.2,1,4\right)\in P_{\mathrm{SP},1}$ together with initial population $X_{0}=5$ on both a unit scale (**left graph**) and a logarithmic scale (**right graph**).

**Figure 3:**
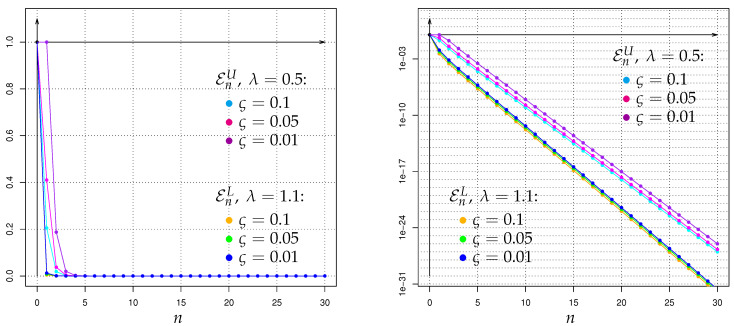
The lower bound $E_{n}^{L}$ (using λ=1.1) and the upper bound $E_{n}^{U}$ (using λ=0.5) of the minimal type II error probability $E_{\varsigma }\left(P_{A,n}∥P_{H,n}\right)$ for different levels ς∈{0.01,0.05,0.1} in the parameter setup $\left(\beta_{A},\beta_{H},\alpha_{A},\alpha_{H}\right)=\left(0.3,1.2,1,4\right)\in P_{\mathrm{SP},1}$ together with initial population $X_{0}=5$ on both a unit scale (**left graph**) and a logarithmic scale (**right graph**).

**Example** **2.** *Consider the Figure 2 and Figure 3 which deal with initial population $X_{0}=5$ and the parameter setup $\left(\beta_{A},\beta_{H},\alpha_{A},\alpha_{H}\right)=\left(0.3,1.2,1,4\right)\in P_{\mathrm{SP},1}$; within our running-example epidemiological context of Section 2.3, this corresponds to a “potentially dangerous” infectious-disease-transmission situation (H) (with supercritical reproduction number $\beta_{H}=1.2$ and importation mean of $\alpha_{H}=4$), whereas (A) describes a “very mild” situation (with “low” subcritical $\beta_{A}=0.3$ and $\alpha_{A}=1$). Figure 2 shows the lower and upper bounds of $E_{\varsigma }\left(P_{A,n}∥P_{H,n}\right)$ with ς=0.05, evaluated from the Formulas (23) and (24), together with the exact values of the Hellinger integral $H_{\lambda }\left(P_{A,n}∥P_{H,n}\right)$, cf. Theorem 1 (recall that we are in the setup $P_{\mathrm{SP},1}$) on both a unit scale (left graph) and a logarithmic scale (right graph). The orange resp. red resp. purple curves correspond to the outcoming upper bounds $E_{n}^{U}:=E_{n}^{U}(P_{A,n}∥P_{H,n})$ (cf. (23)) with parameters λ=0.3 resp. λ=0.5 resp. λ=0.7. The green resp. cyan resp. blue curves correspond to the lower bounds $E_{n}^{L}:=E_{n}^{L}(P_{A,n}∥P_{H,n})$ (cf. (24)) with parameters λ=2 resp. λ=1.5 resp. λ=1.1. Notice the different λ-ranges in (23) and (24). In contrast, Figure 3 compares the lower bound $E_{n}^{L}$ (for fixed λ=1.1) with the upper bound $E_{n}^{U}$ (for fixed λ=0.5) of the minimal type II error probability $E_{\varsigma }(P_{A,n}∥P_{H,n})$ for different levels ς=0.1 (orange for the lower and cyan for the upper bound), ς=0.05 (green and magenta) and ς=0.01 (blue and purple) on both a unit scale (left graph) and a logarithmic scale (right graph).*

### 3.16. Goals for Lower Bounds for the Cases $\left(\beta_{A},\beta_{H},\alpha_{A},\alpha_{H},\lambda \right)\in \left(P_{\mathrm{SP}}\backslash P_{\mathrm{SP},1}\right)\times \left(R\backslash \left[0,1\right]\right)$

Recall from (49) the set $P_{\mathrm{SP}}:=\left\{\;\left(\beta_{A},\beta_{H},\alpha_{A},\alpha_{H}\right)\in \;{]0,\infty [}^{4}\;:\;\left(\alpha_{A}\neq \alpha_{H}\right)\;\;\mathrm{or}\;\;\left(\beta_{A}\neq \beta_{H}\right)\;\;\mathrm{or}\mathrm{both}\;\right\}$ and the “equal-fraction-case” set $P_{\mathrm{SP},1}:=\left\{\;\left(\beta_{A},\beta_{H},\alpha_{A},\alpha_{H}\right)\in P_{\mathrm{SP}}\;:\;\alpha_{A}\neq \alpha_{H},\;\beta_{A}\neq \beta_{H},\;\frac{\alpha_{A}}{\beta_{A}}=\frac{\alpha_{H}}{\beta_{H}}\;\right\}$, where for the latter we have derived in Theorem 1(a) and in Proposition 5 the *exact* recursive values for the time-behaviour of the Hellinger integrals $H_{\lambda }(P_{A,1}∥P_{H,1})$ of order λ∈R\[0,1]. Moreover, recall that for the case $\left(\beta_{A},\beta_{H},\alpha_{A},\alpha_{H},\lambda \right)\in \left(P_{\mathrm{SP}}\backslash P_{\mathrm{SP},1}\right)\times ]0,1[$ we have obtained in the Section 3.4 and Section 3.5 some “optimal” linear lower bounds $\phi_{\lambda }^{L}\left(\cdot \right)$ for the strictly concave function $\phi_{\lambda }\left(x\right):=\phi \left(x,\beta_{A},\beta_{H},\alpha_{A},\alpha_{H},\lambda \right)$ on the domain x∈[0,∞[; due to the monotonicity Properties 2 (P10) to (P12) of the sequences ${\left(a_{n}^{\left(q_{\lambda }^{L}\right)}\right)}_{n\in N}$ and ${\left(b_{n}^{\left(p_{\lambda }^{L},q_{\lambda }^{L}\right)}\right)}_{n\in N}$, these bounds have led to the “optimal” recursive lower bound $B_{\lambda ,X_{0},n}^{L}$ of the Hellinger integral $H_{\lambda }(P_{A,n}∥P_{H,n})$ in (40) of Theorem 1(b)).

In contrast, the strict *convexity* of the function $\phi_{\lambda }\left(\cdot \right)$ in the case $\left(\beta_{A},\beta_{H},\alpha_{A},\alpha_{H},\lambda \right)\in \left(P_{\mathrm{SP}}\backslash P_{\mathrm{SP},1}\right)\times \left(R\backslash \left[0,1\right]\right)$ implies that we cannot maximize both parameters $p_{\lambda }^{L},\;q_{\lambda }^{L}\in R$*simultaneously* subject to the constraint (35). This effect carries over to the lower bounds $B_{\lambda ,X_{0},n}^{L}$ of the Hellinger integrals $H_{\lambda }(P_{A,n}∥P_{H,n})$ (cf. (41)); in general, these bounds cannot be maximized *simultaneously* for all initial population sizes $X_{0}\in N$ and all observation horizons n∈N.

Analogously to (46), one way to obtain “good” recursive lower bounds for $H_{\lambda }(P_{A,n}∥P_{H,n})$ from (41) in Theorem 1 (b) is to solve the optimization problem,

(55) $$\left(\bar{p_{\lambda }^{L}},\bar{q_{\lambda }^{L}}\right)\;:=\;\arg\max_{\;\left(p_{\lambda }^{L},q_{\lambda }^{L}\right)\in R^{2}}\left\{\exp\left\{a_{n}^{\left(q_{\lambda }^{L}\right)}\cdot X_{0}\;+\;\sum_{k=1}^{n}b_{k}^{\left(p_{\lambda }^{L},q_{\lambda }^{L}\right)}\right\}\right\}\;\mathrm{such}\;\mathrm{that}\;(35)\;\mathrm{is}\;\mathrm{satisfied},$$

for each fixed initial population size $X_{0}\in N$ and observation horizon n∈N. But due to the same reasons as explained right after (46), the optimization problem (55) seems to be not straightforward to solve explicitly. In a congeneric way as in the discussion of the upper bounds for the case λ∈]0,1[ above, we now have to look for suitable parameters $p_{\lambda }^{L},\;q_{\lambda }^{L}$ for the lower bound $B_{\lambda ,X_{0},n}^{L}\leq H_{\lambda }(P_{A,n}∥P_{H,n})$ that fulfill (35) and that guarantee certain reasonable criteria and goals; these are similar to the goals (G1) to (G3) from Section 3.6, and are therefore supplemented by an additional “ ${}^{\prime }$ ”:

(G1${}^{\prime }$) the validity of $B_{\lambda ,X_{0},n}^{L}>1$ *simultaneously* for all initial configurations $X_{0}\in N$, all observation horizons n∈N and all λ∈R\[0,1], which leads to a *strict* improvement of the general upper bound $H_{\lambda }(P_{A,n}∥P_{H,n})>1$ (cf. (11));
(G2${}^{\prime }$) the determination of the long-term-limits $\lim_{n\to \infty }H_{\lambda }(P_{A,n}∥P_{H,n})$ respectively $\lim_{n\to \infty }B_{\lambda ,X_{0},n}^{L}$ for all $X_{0}\in N$ and all λ∈R\[0,1]; in particular, one would like to check whether $\lim_{n\to \infty }H_{\lambda }(P_{A,n}∥P_{H,n})=\infty$;
(G3${}^{\prime }$) the determination of the time-asymptotical growth rates $\lim_{n\to \infty }\frac{1}{n}\log\left(H_{\lambda }(P_{A,n}∥P_{H,n})\right)$ resp. $\lim_{n\to \infty }\frac{1}{n}\log\left(B_{\lambda ,X_{0},n}^{L}\right)$ for all $X_{0}\in N$ and all λ∈R\[0,1].

In the following, let us briefly discuss how these three goals can be achieved in principle, where we confine ourselves to parameters $p_{\lambda }^{L},\;q_{\lambda }^{L}$ which–in addition to (35)–fulfill the requirement

(56) $$\left\{\;q_{\lambda }^{L}\geq \max\left\{0,\beta_{\lambda }\right\}\;\wedge \;p_{\lambda }^{L}>\max\left\{0,\alpha_{\lambda }\right\}\;\right\}\;\;\vee \;\left\{\;q_{\lambda }^{L}>\max\left\{0,\beta_{\lambda }\right\}\;\wedge \;p_{\lambda }^{L}\geq \max\left\{0,\alpha_{\lambda }\right\}\;\right\}\;,$$

where ∧ is the logical “AND” and ∨ the logical “OR” operator. This is sufficient to tackle all three Goals (G1${}^{\prime }$) to (G3${}^{\prime }$). To see this, assume that $p_{\lambda }^{L},\;q_{\lambda }^{L}$ satisfy (35). Let us begin with the two “extremal” cases in (56), i.e., with (i) $q_{\lambda }^{L}=\max\left\{0,\beta_{\lambda }\right\},\;p_{\lambda }^{L}>\max\left\{0,\alpha_{\lambda }\right\}$, respectively (ii) $q_{\lambda }^{L}>\max\left\{0,\beta_{\lambda }\right\},\;p_{\lambda }^{L}=\max\left\{0,\alpha_{\lambda }\right\}$.

Suppose in the first extremal case (i) that $\beta_{\lambda }\leq 0$. Then, $q_{\lambda }^{L}=0$ and Properties 1 (P4) implies that $a_{n}^{\left(q_{\lambda }^{L}\right)}=-\beta_{\lambda }\geq 0$ and hence $b_{n}^{\left(p_{\lambda }^{L},q_{\lambda }^{L}\right)}=p_{\lambda }^{L}e^{-\beta_{\lambda }}-\alpha_{\lambda }\geq p_{\lambda }^{L}-\alpha_{\lambda }>0$ for all n∈N. This enters into (41) as follows: the Hellinger integral lower bound becomes $B_{\lambda ,X_{0},n}^{L}\geq {\tilde{B}}_{\lambda ,X_{0},n}^{\left(p_{\lambda }^{L},q_{\lambda }^{L}\right)}=\exp\left\{-\beta_{\lambda }\cdot X_{0}+\left(p_{\lambda }^{L}e^{-\beta_{\lambda }}-\alpha_{\lambda }\right)\cdot n\right\}>1$. Furthermore, one clearly has $\lim_{n\to \infty }B_{\lambda ,X_{0},n}^{L}=\infty$ as well as $\lim_{n\to \infty }\frac{1}{n}\log\left(B_{\lambda ,X_{0},n}^{L}\right)=p_{\lambda }^{L}e^{-\beta_{\lambda }}-\alpha_{\lambda }>0$. Assume now that $\beta_{\lambda }>0$. Then, $q_{\lambda }^{L}=\beta_{\lambda }>0$, $a_{n}^{\left(q_{\lambda }^{L}\right)}=0$ (cf. (P2)), $b_{n}^{\left(p_{\lambda }^{L},q_{\lambda }^{L}\right)}=p_{\lambda }^{L}-\alpha_{\lambda }>0$ and thus $B_{\lambda ,X_{0},n}^{L}=\exp\left\{\left(p_{\lambda }^{L}-\alpha_{\lambda }\right)\cdot n\right\}>1$ for all n∈N. Furthermore, one gets $\lim_{n\to \infty }B_{\lambda ,X_{0},n}^{L}=\infty$ as well as $\lim_{n\to \infty }\frac{1}{n}\log\left(B_{\lambda ,X_{0},n}^{L}\right)=p_{\lambda }^{L}-\alpha_{\lambda }>0$.

Let us consider the other above-mentioned extremal case (ii). Suppose that $q_{\lambda }^{L}>\max\left\{0,\beta_{\lambda }\right\}$ together with $q_{\lambda }^{L}>\min\left\{1,e^{\beta_{\lambda }-1}\right\}$ which implies that the sequence ${\left(a_{n}^{\left(q_{\lambda }^{L}\right)}\right)}_{n\in N}$ is strictly positive, strictly increasing and grows to infinity faster than exponentially, cf. (P3b). Hence, $B_{\lambda ,X_{0},n}^{L}\geq \exp\left\{a_{n}^{\left(q_{\lambda }^{L}\right)}\cdot X_{0}\right\}>1$, $\lim_{n\to \infty }B_{\lambda ,X_{0},n}^{L}=\infty$ as well as $\lim_{n\to \infty }\frac{1}{n}\log\left(B_{\lambda ,X_{0},n}^{L}\right)=\infty$. If $\max\left\{0,\beta_{\lambda }\right\}<q_{\lambda }^{L}\leq \min\left\{1,e^{\beta_{\lambda }-1}\right\}$, then ${\left(a_{n}^{\left(q_{\lambda }^{L}\right)}\right)}_{n\in N}$ is strictly positive, strictly increasing and converges to $x_{0}^{\left(q_{\lambda }\right)}\in ]0,-\log\left(q_{\lambda }^{L}\right)]$ (cf. (P3a)). This carries over to the sequence ${\left(b_{n}^{\left(p_{\lambda }^{L},q_{\lambda }^{L}\right)}\right)}_{n\in N}$: one gets $b_{1}^{\left(p_{\lambda }^{L},q_{\lambda }^{L}\right)}=p_{\lambda }^{L}-\alpha_{\lambda }\geq 0$ and $b_{n}^{\left(p_{\lambda }^{L},q_{\lambda }^{L}\right)}>0$ for all n≥2. Furthermore, $b_{n}^{\left(p_{\lambda }^{L},q_{\lambda }^{L}\right)}$ is strictly increasing and converges to $p_{\lambda }^{L}\cdot e^{x_{0}^{\left(q_{\lambda }^{L}\right)}}-\alpha_{\lambda }>0$, leading to $B_{\lambda ,X_{0},n}^{L}>1$ for all n∈N, to $\lim_{n\to \infty }B_{\lambda ,X_{0},n}^{L}=\infty$ as well as to $\lim_{n\to \infty }\frac{1}{n}\log\left(B_{\lambda ,X_{0},n}^{L}\right)=p_{\lambda }^{L}\cdot e^{x_{0}^{\left(q_{\lambda }^{L}\right)}}-\alpha_{\lambda }>0$.

It remains to look at the cases where $p_{\lambda }^{L},\;q_{\lambda }^{L}$ satisfy (35), and (56) with two strict inequalities. For this situation, one gets

- ${\left(a_{n}^{\left(q_{\lambda }^{L}\right)}\right)}_{n\in N}$ is strictly positive, strictly increasing and–iff $q_{\lambda }^{L}\leq \min\left\{1,e^{\beta_{\lambda }-1}\right\}$–convergent (namely to the smallest positive solution $x_{0}^{\left(q_{\lambda }^{L}\right)}\in ]0,-\log\left(q_{\lambda }^{L}\right)]$ of (44)), cf. (P3);
- ${\left(b_{n}^{\left(p_{\lambda }^{L},q_{\lambda }^{L}\right)}\right)}_{n\in N}$ is strictly increasing, strictly positive (since $b_{1}^{\left(p_{\lambda }^{L},q_{\lambda }^{L}\right)}=p_{\lambda }^{L}-\alpha_{\lambda }>0$) and–iff $q_{\lambda }^{L}\leq \min\left\{1,e^{\beta_{\lambda }-1}\right\}$–convergent (namely to $p_{\lambda }^{L}e^{x_{0}^{\left(q_{\lambda }^{L}\right)}}\;\;\;-\;\alpha_{\lambda }\in \left[p_{\lambda }^{L}-\alpha_{\lambda },p_{\lambda }^{L}/q_{\lambda }^{L}-\alpha_{\lambda }\right]$), cf (P7).

Hence, under the assumptions (35) and $\left(p_{\lambda }^{L}>\max\left\{0,\alpha_{\lambda }\right\}\right)\wedge \left(q_{\lambda }^{L}>\max\left\{0,\beta_{\lambda }\right\}\right)$ the corresponding lower bounds $B_{\lambda ,X_{0},n}^{L}$ of the Hellinger integral $H_{\lambda }(P_{A,n}∥P_{H,n})$ fulfill for all $X_{0}\in N$

- $B_{\lambda ,X_{0},n}^{L}>1$ for all n∈N,
- $\lim_{n\to \infty }B_{\lambda ,X_{0},n}^{L}=\infty$,
- $\lim_{n\to \infty }\frac{1}{n}\log\left(B_{\lambda ,X_{0},n}^{L}\right)=p_{\lambda }^{L}e^{x_{0}^{\left(q_{\lambda }^{L}\right)}}-\alpha_{\lambda }>0$ for the case $q_{\lambda }^{L}\in \;]\max\left\{0,\beta_{\lambda }\right\},\min\left\{1,e^{\beta_{\lambda }-1}\right\}]$, respectively $\lim_{n\to \infty }\frac{1}{n}\log\left(B_{\lambda ,X_{0},n}^{L}\right)=\infty$ for the remaining case $q_{\lambda }^{L}>\min\left\{1,e^{\beta_{\lambda }-1}\right\}$.

Putting these considerations together we conclude that the constraints (35) and (56) are sufficient to achieve the Goals (G1${}^{\prime }$) to (G3${}^{\prime }$). Hence, for fixed parameter constellation $\left(\beta_{A},\beta_{H},\alpha_{A},\alpha_{H},\lambda \right)$, we aim for finding $p_{\lambda }^{L}=p^{L}\left(\beta_{A},\beta_{H},\alpha_{A},\alpha_{H},\lambda \right)$ and $q_{\lambda }^{L}=q^{L}\left(\beta_{A},\beta_{H},\alpha_{A},\alpha_{H},\lambda \right)$ which satisfy (35) and (56). This can be achieved mostly, but not always, as we shall show below. As an auxiliary step for further investigations, it is useful to examine the set of all λ∈R\[0,1] for which $\alpha_{\lambda }\leq 0$ or $\beta_{\lambda }\leq 0$ (or both). By straightforward calculations, we see that

(57) $$\alpha_{\lambda }\leq 0\;⟺\;\lambda \;\left\{\begin{array}{ccc} \leq & \frac{-\alpha_{H}}{\alpha_{A}-\alpha_{H}}, & \mathrm{if}\;\alpha_{A}>\alpha_{H},\\ \geq & \frac{\alpha_{H}}{\alpha_{H}-\alpha_{A}}, & \mathrm{if}\;\alpha_{A}<\alpha_{H}, \end{array}\right.\;\mathrm{and}\;\beta_{\lambda }\leq 0\;⟺\;\lambda \;\left\{\begin{array}{ccc} \leq & \frac{-\beta_{H}}{\beta_{A}-\beta_{H}}, & \mathrm{if}\;\beta_{A}>\beta_{H},\\ \geq & \frac{\beta_{H}}{\beta_{H}-\beta_{A}}, & \mathrm{if}\;\beta_{A}<\beta_{H}. \end{array}\right.$$

Furthermore, recall that (35) implies the general bounds $p_{\lambda }^{L}\leq \alpha_{A}^{\lambda }\alpha_{H}^{1-\lambda }=\varphi_{\lambda }\left(0\right)$ (being equivalent to the requirement $\phi_{\lambda }^{L}\left(0\right)=\phi_{\lambda }\left(0\right)$) and $q_{\lambda }^{L}\leq \beta_{A}^{\lambda }\beta_{H}^{1-\lambda }={\tilde{q}}_{\lambda }$ (the latter being the maximal slope due to Properties 3 (P19), (P20)).

Let us now undertake the desired *detailed* investigations on lower and upper bounds of the Hellinger integrals $H_{\lambda }(P_{A,n}∥P_{H,n})$ of order λ∈R\[0,1], for the various different subclasses of $P_{\mathrm{SP}}\backslash P_{\mathrm{SP},1}$.

### 3.17. Lower Bounds for the Cases $\left(\beta_{A},\beta_{H},\alpha_{A},\alpha_{H},\lambda \right)\in P_{\mathrm{SP},2}\times \left(R\backslash \left[0,1\right]\right)$

In such a constellation, where $P_{\mathrm{SP},2}:=\left\{\;\left(\beta_{A},\beta_{H},\alpha_{A},\alpha_{H}\right)\in P_{\mathrm{SP}}\;:\;\alpha_{A}=\alpha_{H},\;\beta_{A}\neq \beta_{H}\;\right\}$ (cf. (49)), one gets $\phi_{\lambda }\left(0\right)=0$ (cf. Properties 3 (P16)), $\phi_{\lambda }^{\prime }\left(0\right)=0$ (cf. (P17)). Thus, the only choice for the intercept and the slope of the linear lower bound $\phi_{\lambda }^{L}\left(\cdot \right)$ for $\phi_{\lambda }\left(\cdot \right)$, which satisfies (35) for all x∈N and (potentially) (56), is $r_{\lambda }^{L}=0=p_{\lambda }^{L}-\alpha_{\lambda }$ (i.e., $p_{\lambda }^{L}=\alpha_{\lambda }=\alpha >0$) and $s_{\lambda }^{L}=\frac{\phi_{\lambda }\left(1\right)-\phi_{\lambda }\left(0\right)}{1-0}=q_{\lambda }^{L}-\beta_{\lambda }=a_{1}^{\left(q_{\lambda }^{L}\right)}>0$ (i.e., $q_{\lambda }^{L}={\left(\alpha +\beta_{A}\right)}^{\lambda }{\left(\alpha +\beta_{H}\right)}^{1-\lambda }-\alpha$). However, since $p_{\lambda }^{L}=\alpha_{\lambda }=\alpha >0$, the restriction (56) is fulfilled iff $q_{\lambda }^{L}>0$, which is equivalent to

(58) $$\lambda \;\in \;I_{\mathrm{SP},2}\;:=\;\left\{\begin{array}{cc} ]\frac{\log\left(\frac{\alpha }{\alpha +\beta_{H}}\right)}{\log\left(\frac{\alpha +\beta_{A}}{\alpha +\beta_{H}}\right)}\;,\;0\left[\;\cup \;\right]1,\infty [\;, & \mathrm{if}\;\beta_{A}\;>\;\beta_{H},\\ ]-\infty ,0\left[\;\cup \;\right]1\;,\;\frac{\log\left(\frac{\alpha }{\alpha +\beta_{H}}\right)}{\log\left(\frac{\alpha +\beta_{A}}{\alpha +\beta_{H}}\right)}[\;, & \mathrm{if}\;\beta_{A}\;<\;\beta_{H}. \end{array}\right.$$

Suppose that $\lambda \in I_{\mathrm{SP},2}$. As we have seen above, from Properties 1 (P3a) and (P3b) one can derive that ${\left(a_{n}^{\left(q_{\lambda }^{L}\right)}\right)}_{n\in N}$ is strictly positive, strictly increasing, and converges to $x_{0}^{\left(q_{\lambda }^{L}\right)}\in ]0,-\log\left(q_{\lambda }^{L}\right)]$ iff $q_{\lambda }^{L}\leq \min\left\{1\;,\;e^{\beta_{\lambda }-1}\right\}$, and otherwise it diverges to *∞*. Notice that both cases can occur: consider the parameter setup $\left(\beta_{A},\beta_{H},\alpha_{A},\alpha_{H}\right)=\left(1.5,0.5,0.5,0.5\right)\in P_{\mathrm{SP},2}$, which leads to $I_{\mathrm{SP},2}=]-1,0\left[\;\cup \;\right]1,\infty [$; within our running-example epidemiological context of Section 2.3, this corresponds to a “mild” infectious-disease-transmission situation (H) (with “low” reproduction number $\beta_{H}=0.5$ and importation mean of $\alpha_{H}=0.5$), whereas (A) describes a “dangerous” situation (with supercritical $\beta_{A}=1.5$ and $\alpha_{A}=0.5$). For $\lambda =-0.5\in I_{\mathrm{SP},2}$ one obtains $q_{\lambda }^{L}\approx 0.207\leq \min\left\{1\;,\;e^{\beta_{\lambda }-1}\right\}\approx 0.368$, whereas for $\lambda =2\in I_{\mathrm{SP},2}$ one gets $q_{\lambda }^{L}=3.5>\min\left\{1\;,\;e^{\beta_{\lambda }-1}\right\}=1$. Altogether, this leads to

**Proposition** **11.** *For all $\left(\beta_{A},\beta_{H},\alpha_{A},\alpha_{H},\lambda \right)\in P_{\mathrm{SP},2}\times I_{\mathrm{SP},2}$ and all initial population sizes $X_{0}\in N$ there holds with $p_{\lambda }^{L}=\alpha_{A}=\alpha_{H}=\alpha ,\;q_{\lambda }^{L}={\left(\alpha +\beta_{A}\right)}^{\lambda }{\left(\alpha +\beta_{H}\right)}^{1-\lambda }-\alpha$*

$$\begin{array}{cc} \left(a\right) & \;B_{\lambda ,X_{0},1}^{L}\;=\;{\tilde{B}}_{\lambda ,X_{0},1}^{\left(p_{\lambda }^{L},q_{\lambda }^{L}\right)}\;=\;\exp\left\{\left(q_{\lambda }^{L}-\beta_{\lambda }\right)\cdot X_{0}\right\}\;>\;1,\;\;\\ \left(b\right) & \;\mathrm{the}\;\mathrm{sequence}{\left(B_{\lambda ,X_{0},n}^{L}\right)}_{n\in N}\;\mathrm{of}\;\mathrm{lower}\;\mathrm{bounds}\;\mathrm{for}\;H_{\lambda }(P_{A,n}∥P_{H,n})\;\mathrm{given}\;\mathrm{by}\\ & \;B_{\lambda ,X_{0},n}^{L}\;=\;{\tilde{B}}_{\lambda ,X_{0},n}^{\left(p_{\lambda }^{L},q_{\lambda }^{L}\right)}\;=\;\exp\left\{a_{n}^{\left(q_{\lambda }^{L}\right)}\cdot X_{0}\;+\;\sum_{k=1}^{n}b_{k}^{\left(p_{\lambda }^{L},q_{\lambda }^{L}\right)}\right\}\\ & \;\mathrm{is}\;\mathrm{strictly}\;\mathrm{increasing},\\ \left(c\right) & \;\lim_{n\to \infty }B_{\lambda ,X_{0},n}^{L}\;=\;\infty \;=\;\lim_{n\to \infty }H_{\lambda }(P_{A,n}∥P_{H,n})\;,\\ \left(d\right) & \lim_{n\to \infty }\;\frac{1}{n}\;\log B_{\lambda ,X_{0},n}^{L}\;=\;\left\{\begin{array}{cc} p_{\lambda }^{L}\cdot \exp\left\{x_{0}^{\left(q_{\lambda }^{L}\right)}\right\}-\alpha \;>\;0,\; & \mathrm{if}\;q_{\lambda }^{L}\;\leq \;\min\left\{1,e^{\beta_{\lambda }-1}\right\}\;,\\ \infty , & \mathrm{if}\;q_{\lambda }^{L}\;>\;\min\left\{1,e^{\beta_{\lambda }-1}\right\}\;, \end{array}\right.\\ \left(e\right) & \;\mathrm{the}\;\mathrm{map}\;\;X_{0}\;\mapsto \;B_{\lambda ,X_{0},n}^{L}={\tilde{B}}_{\lambda ,X_{0},n}^{\left(p_{\lambda }^{L},q_{\lambda }^{L}\right)}\;\mathrm{is}\;\mathrm{strictly}\;\mathrm{increasing}. \end{array}$$

Nevertheless, for the remaining constellations $\left(\beta_{A},\beta_{H},\alpha_{A},\alpha_{H},\lambda \right)\in P_{\mathrm{SP},2}\times R\backslash \left(I_{\mathrm{SP},2}\cup \left[0,1\right]\right)$, all observation time horizons n∈N and all initial population sizes $X_{0}\in N$ one can still prove

(59) $$1\;<\;H_{\lambda }\left(P_{A,n}∥P_{H,n}\right)\;\;\mathrm{and}\;\lim_{n\to \infty }H_{\lambda }\left(P_{A,n}∥P_{H,n}\right)\;=\;\infty \;,$$

(i.e., the achievement of the Goals (G1${}^{\prime }$), (G2${}^{\prime }$)), which is done by a conceptually different method (without involving $p_{\lambda }^{L},\;q_{\lambda }^{L}$) in Appendix A.1.

### 3.18. Lower Bounds for the Cases $\left(\beta_{A},\beta_{H},\alpha_{A},\alpha_{H},\lambda \right)\in P_{\mathrm{SP},3a}\times \left(R\backslash \left[0,1\right]\right)$

In the current setup, where $P_{\mathrm{SP},3a}:=\left\{\;\left(\beta_{A},\beta_{H},\alpha_{A},\alpha_{H}\right)\in P_{\mathrm{SP}}\;:\;\alpha_{A}\neq \alpha_{H},\;\beta_{A}\neq \beta_{H},\;\frac{\alpha_{A}}{\beta_{A}}\neq \frac{\alpha_{H}}{\beta_{H}},\;\frac{\alpha_{A}-\alpha_{H}}{\beta_{H}-\beta_{A}}\in \;]-\infty ,0[\;\right\}$ (cf. (49)), we *always* have either $\left(\alpha_{A}>\alpha_{H}\right)\wedge \left(\beta_{A}>\beta_{H}\right)$ or $\left(\alpha_{A}<\alpha_{H}\right)\wedge \left(\beta_{A}<\beta_{H}\right)$. Furthermore, from Properties 3 (P16) we obtain $\phi_{\lambda }\left(0\right)>0$. As in the case λ∈]0,1[, the derivative $\phi_{\lambda }^{\prime }\left(0\right)$ can assume any sign on $P_{\mathrm{SP},3a}$, take e.g., $\left(\beta_{A},\beta_{H},\alpha_{A},\alpha_{H},\lambda \right)=\left(2.2,4.5,1,3,2\right)$ for $\phi_{\lambda }^{\prime }\left(0\right)<0$, $\left(\beta_{A},\beta_{H},\alpha_{A},\alpha_{H},\lambda \right)=\left(2.25,4.5,1,3,2\right)$ for $\phi_{\lambda }^{\prime }\left(0\right)=0$ and $\left(\beta_{A},\beta_{H},\alpha_{A},\alpha_{H},\lambda \right)=\left(2.3,4.5,1,3,2\right)$ for $\phi_{\lambda }^{\prime }\left(0\right)>0$ (these parameter constellations reflect “dangerous” (A) versus “highly dangerous” (H) situations within our running-example epidemiological context of Section 2.3). Nevertheless, in all three subcases one gets $\min_{x\in N_{0}}\phi_{\lambda }\left(x\right)\geq \min_{x\geq 0}\phi_{\lambda }\left(x\right)>0$. Thus, there exist parameters $p_{\lambda }^{L}\in ]\alpha_{\lambda },\alpha_{A}^{\lambda }\alpha_{H}^{1-\lambda }]$ and $q_{\lambda }^{L}\in ]\beta_{\lambda },\beta_{A}^{\lambda }\beta_{H}^{1-\lambda }]$ which satisfy (35) (in particular, $p_{\lambda }^{L}-\alpha_{\lambda }>0,\;q_{\lambda }^{L}-\beta_{\lambda }>0$). We now have to look for a condition which guarantees that these parameters *additionally* fulfill (56); such a condition is clearly that both $\alpha_{\lambda }\geq 0$ and $\beta_{\lambda }\geq 0$ hold, which is equivalent (cf. (57)) with

$$\lambda \;\in \;I_{\mathrm{SP},3a}^{\left(\geq \right)}\;:=\;\left\{\begin{array}{cc} {}[\max\left\{\frac{-\alpha_{H}}{\alpha_{A}-\alpha_{H}},\frac{-\beta_{H}}{\beta_{A}-\beta_{H}}\right\}\;,\;0\left[\;\cup \;\right]1,\infty [, & \mathrm{if}\;\left(\alpha_{A}>\alpha_{H}\right)\wedge \left(\beta_{A}>\beta_{H}\right),\\ \left[-\infty ,0\left[\;\cup \;\right]1,\min\left\{\frac{\alpha_{H}}{\alpha_{H}-\alpha_{A}},\frac{\beta_{H}}{\beta_{H}-\beta_{A}}\right\}\right], & \mathrm{if}\;\left(\alpha_{A}<\alpha_{H}\right)\wedge \left(\beta_{A}<\beta_{H}\right); \end{array}\right.$$

recall that $\alpha_{\lambda }=0$ and $\beta_{\lambda }=0$ cannot occur simultaneously in the current setup. If $\alpha_{\lambda }\leq 0$ and $\beta_{\lambda }\leq 0$, i.e., if

$$\lambda \;\in \;I_{\mathrm{SP},3a}^{\left(<\right)}\;:=\;\left\{\begin{array}{cc} ]-\infty \;,\;\min\left\{\frac{-\alpha_{H}}{\alpha_{A}-\alpha_{H}};\frac{-\beta_{H}}{\beta_{A}-\beta_{H}}\right\}], & \mathrm{if}\;\left(\alpha_{A}>\alpha_{H}\right)\wedge \left(\beta_{A}>\beta_{H}\right),\\ {}[\max\left\{\frac{\alpha_{H}}{\alpha_{H}-\alpha_{A}};\frac{\beta_{H}}{\beta_{H}-\beta_{A}}\right\}\;,\;\infty [, & \mathrm{if}\;\left(\alpha_{A}<\alpha_{H}\right)\wedge \left(\beta_{A}<\beta_{H}\right), \end{array}\right.$$

then–due to the strict positivity of the function $\varphi_{\lambda }\left(\cdot \right)$ (cf. (31))–there exist parameters $p_{\lambda }^{L}>0=\max\left\{0,\alpha_{\lambda }\right\}$ and $q_{\lambda }^{L}>0=\max\left\{0,\beta_{\lambda }\right\}$ which satisfy (56) and (34) (where the latter implies (35) and thus $p_{\lambda }^{L}\leq \alpha_{A}^{\lambda }\alpha_{H}^{1-\lambda },\;q_{\lambda }^{L}\leq \beta_{A}^{\lambda }\beta_{H}^{1-\lambda }$). With

(60) $$I_{\mathrm{SP},3a}\;:=\;I_{\mathrm{SP},3a}^{\left(\geq \right)}\;\cup \;I_{\mathrm{SP},3a}^{\left(<\right)}\;$$

and with the discussion below (56), we thus derive the following

**Proposition** **12.** *For all $\left(\beta_{A},\beta_{H},\alpha_{A},\alpha_{H},\lambda \right)\in P_{\mathrm{SP},3a}\times I_{\mathrm{SP},3a}$ there exist parameters $p_{\lambda }^{L},\;q_{\lambda }^{L}$ which satisfy $\max\left\{0,\alpha_{\lambda }\right\}<p_{\lambda }^{L}\leq \alpha_{A}^{\lambda }\alpha_{H}^{1-\lambda },\;\max\left\{0,\beta_{\lambda }\right\}<q_{\lambda }^{L}\leq \beta_{A}^{\lambda }\beta_{H}^{1-\lambda }$ as well as (35) for all $x\in N_{0}$, and for all such pairs $\left(p_{\lambda }^{L},\;q_{\lambda }^{L}\right)$ and all initial population sizes $X_{0}\in N$ one gets*

$$\begin{array}{cc} \left(a\right) & \;B_{\lambda ,X_{0},1}^{L}\;=\;{\tilde{B}}_{\lambda ,X_{0},1}^{\left(p_{\lambda }^{L},q_{\lambda }^{L}\right)}\;=\;\exp\left\{\left(q_{\lambda }^{L}-\beta_{\lambda }\right)\cdot X_{0}\;+\;p_{\lambda }^{L}-\alpha_{\lambda }\right\}\;>\;1,\;\;\\ \left(b\right) & \;\mathrm{the}\;\mathrm{sequence}{\left(B_{\lambda ,X_{0},n}^{L}\right)}_{n\in N}\;\mathrm{of}\;\mathrm{lower}\;\mathrm{bounds}\;\mathrm{for}\;H_{\lambda }(P_{A,n}∥P_{H,n})\;\mathrm{given}\;\mathrm{by}\\ & \;B_{\lambda ,X_{0},n}^{L}\;=\;{\tilde{B}}_{\lambda ,X_{0},n}^{\left(p_{\lambda }^{L},q_{\lambda }^{L}\right)}\;=\;\exp\left\{a_{n}^{\left(q_{\lambda }^{L}\right)}\cdot X_{0}\;+\;\sum_{k=1}^{n}b_{k}^{\left(p_{\lambda }^{L},q_{\lambda }^{L}\right)}\right\}\\ & \;\mathrm{is}\;\mathrm{strictly}\;\mathrm{increasing},\\ \left(c\right) & \;\lim_{n\to \infty }B_{\lambda ,X_{0},n}^{L}\;=\;\infty \;=\;\lim_{n\to \infty }H_{\lambda }(P_{A,n}∥P_{H,n})\;,\\ \left(d\right) & \;\lim_{n\to \infty }\;\frac{1}{n}\;\log B_{\lambda ,X_{0},n}^{L}\;=\;\left\{\begin{array}{cc} p_{\lambda }^{L}\cdot \exp\left\{x_{0}^{\left(q_{\lambda }^{L}\right)}\right\}-\alpha_{\lambda }\;>\;0, & \mathrm{if}\;q_{\lambda }^{L}\;\leq \;\min\left\{1,e^{\beta_{\lambda }-1}\right\}\;,\\ \infty , & \mathrm{if}\;q_{\lambda }^{L}\;>\;\min\left\{1,e^{\beta_{\lambda }-1}\right\}\;, \end{array}\right.\\ \left(e\right) & \;\mathrm{the}\;\mathrm{map}\;X_{0}\;\mapsto \;B_{\lambda ,X_{0},n}^{L}={\tilde{B}}_{\lambda ,X_{0},n}^{\left(p_{\lambda }^{L},q_{\lambda }^{L}\right)}\;\mathrm{is}\;\mathrm{strictly}\;\mathrm{increasing}. \end{array}$$

Notice that the assertions (a) to (e) of Proposition 12 hold true for parameter pairs $\left(p_{\lambda }^{L},q_{\lambda }^{L}\right)$ *whenever* they satisfy (35) and (56); in particular, we may allow either $p_{\lambda }^{L}=\max\left\{0,\alpha_{\lambda }\right\}$ or $q_{\lambda }^{L}=\max\left\{0,\beta_{\lambda }\right\}$. Let us furthermore mention that in part (d) both asymptotical behaviours can occur: consider e.g., the parameter setup $\left(\beta_{A},\beta_{H},\alpha_{A},\alpha_{H}\right)=\left(0.3,0.2,4,3\right)\in P_{\mathrm{SP},3a}$, leading to $]1,\infty [\;⊊I_{\mathrm{SP},3a}^{\left(\geq \right)}⊊I_{\mathrm{SP},3a}$. For $\lambda =2\in I_{\mathrm{SP},3a}$, the parameters $p_{\lambda }^{L}:={\tilde{p}}_{\lambda }:=5.25,\;\;q_{\lambda }^{L}:={\tilde{q}}_{\lambda }:=0.45$ (corresponding to the asymptote ${\tilde{\phi }}_{\lambda }\left(\cdot \right)$, cf. (P20)) fulfill (35), (56) and additionally $q_{\lambda }^{L}=0.45<\min\left\{1,e^{\beta_{\lambda }-1}\right\}\approx 0.549$. Analogously, in the setup $\left(\beta_{A},\beta_{H},\alpha_{A},\alpha_{H},\lambda \right)=\left(3,2,4,3,2\right)\in P_{\mathrm{SP},3a}\times I_{\mathrm{SP},3a}$, the choices $p_{\lambda }^{L}:={\tilde{p}}_{\lambda }:=5.25,\;\;q_{\lambda }^{L}:={\tilde{q}}_{\lambda }:=4.5$ satisfy (35), (56) and there holds $q_{\lambda }^{L}=4.5>\min\left\{1,e^{\beta_{\lambda }-1}\right\}=1$.

For the remaining two cases $\left(\alpha_{\lambda }\leq 0\right)\wedge \left(\beta_{\lambda }>0\right)$ (e.g., $\left(\beta_{A},\beta_{H},\alpha_{A},\alpha_{H},\lambda \right)=\left(6,5,3,2,-3\right)$) and $\left(\alpha_{\lambda }>0\right)\wedge \left(\beta_{\lambda }\leq 0\right)$ (e.g., $\left(\beta_{A},\beta_{H},\alpha_{A},\alpha_{H},\lambda \right)=\left(3,2,6,5,-3\right)$), one has to proceed differently. Indeed, for all parameter constellations $\left(\beta_{A},\beta_{H},\alpha_{A},\alpha_{H},\lambda \right)\in P_{\mathrm{SP},3a}\times R\backslash \left(I_{\mathrm{SP},3a}\cup \left[0,1\right]\right)$, all observation time horizons n∈N and all initial population sizes $X_{0}\in N$ one can still prove

(61) $$1\;<\;H_{\lambda }\left(P_{A,n}∥P_{H,n}\right)\;,\;\mathrm{and}\;\lim_{n\to \infty }H_{\lambda }\left(P_{A,n}∥P_{H,n}\right)\;=\;\infty \;,$$

which is done in Appendix A.1, using a similar method as in the proof of assertion (59).

### 3.19. Lower Bounds for the Cases $\left(\beta_{A},\beta_{H},\alpha_{A},\alpha_{H},\lambda \right)\in P_{\mathrm{SP},3b}\times \left(R\backslash \left[0,1\right]\right)$

Within such a constellation, where $P_{\mathrm{SP},3b}:=\left\{\;\left(\beta_{A},\beta_{H},\alpha_{A},\alpha_{H}\right)\in P_{\mathrm{SP}}\;:\;\alpha_{A}\neq \alpha_{H},\;\beta_{A}\neq \beta_{H},\;\frac{\alpha_{A}}{\beta_{A}}\neq \frac{\alpha_{H}}{\beta_{H}},\;\frac{\alpha_{A}-\alpha_{H}}{\beta_{H}-\beta_{A}}\in \;]0,\infty [\backslash N\;\right\}$ (cf. (49)), one *always* has either $\left(\alpha_{A}<\alpha_{H}\right)\wedge \left(\beta_{A}>\beta_{H}\right)$ or $\left(\alpha_{A}>\alpha_{H}\right)\wedge \left(\beta_{A}<\beta_{H}\right)$. Moreover, from Properties 3 (P15) one can see that $\phi_{\lambda }\left(x\right)=0$ for $x=x^{*}=\frac{\alpha_{H}-\alpha_{A}}{\beta_{A}-\beta_{H}}>0$. However, $x^{*}\notin N_{0}$, which implies $\phi_{\lambda }\left(x\right)>0$ for all *x* on the relevant subdomain $N_{0}$. Again, we incorporate (57) and consider the set of all λ∈R\[0,1] such that $\alpha_{\lambda }\geq 0$ and $\beta_{\lambda }\geq 0$ (where $\alpha_{\lambda }=0\wedge \beta_{\lambda }=0$ cannot appear), i.e.,

(62) $$\lambda \;\in \;I_{\mathrm{SP},3b}^{\left(\geq \right)}\;:=\;\left\{\begin{array}{cc} \left[\frac{-\beta_{H}}{\beta_{A}-\beta_{H}}\;,\;0\left[\;\cup \;\right]1\;,\;\frac{\alpha_{H}}{\alpha_{H}-\alpha_{A}}\right], & \mathrm{if}\;\left(\alpha_{A}<\alpha_{H}\right)\wedge \left(\beta_{A}>\beta_{H}\right),\\ \left[\frac{-\alpha_{H}}{\alpha_{A}-\alpha_{H}}\;,\;0\left[\;\cup \;\right]1\;,\;\frac{\beta_{H}}{\beta_{H}-\beta_{A}}\right], & \mathrm{if}\;\left(\alpha_{A}>\alpha_{H}\right)\wedge \left(\beta_{A}<\beta_{H}\right). \end{array}\right.$$

As above in Section 3.18, if $\lambda \in I_{\mathrm{SP},3b}^{\left(\geq \right)}$ then there exist parameters $p_{\lambda }^{L}\in ]\alpha_{\lambda },\alpha_{A}^{\lambda }\alpha_{H}^{1-\lambda }]$, $q_{\lambda }^{L}\in ]\beta_{\lambda },\beta_{A}^{\lambda }\beta_{H}^{1-\lambda }]$ (which thus fulfill (56)) such that (35) is satisfied for all $x\in N_{0}$. Hence, for all $\lambda \in I_{\mathrm{SP},3b}:=I_{\mathrm{SP},3b}^{\left(\geq \right)}$, all assertions (a) to (e) of Proposition 12 hold true. Notice that for the current setup $P_{\mathrm{SP},3b}$ one cannot have $\alpha_{\lambda }\leq 0$ and $\beta_{\lambda }\leq 0$ simultaneously. Furthermore, in each of the two remaining cases $\left(\alpha_{\lambda }<0\right)\wedge \left(\beta_{\lambda }>0\right)$ respectively $\left(\alpha_{\lambda }>0\right)\wedge \left(\beta_{\lambda }<0\right)$ it can happen that there do not exist parameters $p_{\lambda }^{L},\;q_{\lambda }^{L}>0$ which satisfy both (35) and (56). However, as in the case $P_{\mathrm{SP},3a}$ above, for all $\lambda \notin I_{\mathrm{SP},3b}$ we prove in Appendix A.1 (by a method without $p_{\lambda }^{L},\;q_{\lambda }^{L}$) that for all observation times n∈N and all initial population sizes $X_{0}\in N$ there holds

(63) $$1\;<\;H_{\lambda }\left(P_{A,n}∥P_{H,n}\right)\;\;\mathrm{and}\;\lim_{n\to \infty }H_{\lambda }\left(P_{A,n}∥P_{H,n}\right)\;=\;\infty \;.$$

### 3.20. Lower Bounds for the Cases $\left(\beta_{A},\beta_{H},\alpha_{A},\alpha_{H},\lambda \right)\in P_{\mathrm{SP},3c}\times \left(R\backslash \left[0,1\right]\right)$

Since in this subcase one has $P_{\mathrm{SP},3c}:=\left\{\;\left(\beta_{A},\beta_{H},\alpha_{A},\alpha_{H}\right)\in P_{\mathrm{SP}}\;:\;\alpha_{A}\neq \alpha_{H},\;\beta_{A}\neq \beta_{H},\;\frac{\alpha_{A}}{\beta_{A}}\neq \frac{\alpha_{H}}{\beta_{H}},\;\frac{\alpha_{A}-\alpha_{H}}{\beta_{H}-\beta_{A}}\in N\;\right\}$ (cf. (49)) and thus $\phi_{\lambda }\left(x^{*}\right)=0$ for $x^{*}\in N$, there do not exist parameters $p_{\lambda }^{L},\;q_{\lambda }^{L}$ such that (35) and (56) are satisfied. The only parameter pair that ensures $\exp\left\{a_{n}^{\left(q_{\lambda }^{L}\right)}\cdot X_{0}\;+\;\sum_{k=1}^{n}b_{k}^{\left(p_{\lambda }^{L},q_{\lambda }^{L}\right)}\right\}\geq 1$ for all n∈N and all $X_{0}\in N$ within our proposed method, is the choice $p_{\lambda }^{L}=\alpha_{\lambda },\;q_{\lambda }^{L}=\beta_{\lambda }$. Consequently, $B_{\lambda ,X_{0},n}^{L}\equiv 1$, which coincides with the general lower bound (11) but violates the above-mentioned desired Goal (G1${}^{\prime }$). However, in some constellations there exist *nonnegative* parameters $p_{\lambda }^{L}<\alpha_{\lambda },\;q_{\lambda }^{L}>\beta_{\lambda }$ or $p_{\lambda }^{L}>\alpha_{\lambda },\;q_{\lambda }^{L}<\beta_{\lambda }$, such that at least the parts (c) and (d) of Proposition 12 are satisfied. As in Section 3.19 above, by using a conceptually different method (without $p_{\lambda }^{L},\;q_{\lambda }^{L}$) we prove in Appendix A.1 that for all λ∈R\[0,1], all observation times n∈N and all initial population sizes $X_{0}\in N$ there holds

(64) $$1\;<\;H_{\lambda }\left(P_{A,n}∥P_{H,n}\right)\;\;\mathrm{and}\;\lim_{n\to \infty }H_{\lambda }\left(P_{A,n}∥P_{H,n}\right)\;=\;\infty \;.$$

### 3.21. Lower Bounds for the Cases $\left(\beta_{A},\beta_{H},\alpha_{A},\alpha_{H},\lambda \right)\in P_{\mathrm{SP},4a}\times \left(R\backslash \left[0,1\right]\right)$

In the current setup, where $P_{\mathrm{SP},4a}:=\left\{\;\left(\beta_{A},\beta_{H},\alpha_{A},\alpha_{H}\right)\in P_{\mathrm{SP}}\;:\;\alpha_{A}\neq \alpha_{H}>0,\;\beta_{A}=\beta_{H}\in \;]0,1[\;\right\}$ (cf. (49)), the function $\phi_{\lambda }\left(\cdot \right)$ is strictly positive and strictly decreasing, with $\lim_{x\to \infty }\phi_{\lambda }\left(x\right)=\lim_{x\to \infty }\phi_{\lambda }^{\prime }\left(x\right)=0$. The only choice of parameters $p_{\lambda }^{L},\;q_{\lambda }^{L}$ which fulfill (35) and $\exp\left\{a_{n}^{\left(q_{\lambda }^{L}\right)}\cdot X_{0}\;+\;\sum_{k=1}^{n}b_{k}^{\left(p_{\lambda }^{L},q_{\lambda }^{L}\right)}\right\}\geq 1$ for all n∈N and all $X_{0}\in N$, is the choice $p_{\lambda }^{L}=\alpha_{\lambda }$ as well as $q_{\lambda }^{L}=\beta_{\lambda }=\beta_{•}$, where $\beta_{•}$ stands for both (equal) $\beta_{H}$ and $\beta_{A}$. Of course, this leads to $B_{\lambda ,X_{0},n}^{L}\equiv 1$, which is consistent with the general lower bound (11), but violates the above-mentioned desired Goal (G1${}^{\prime }$). Nevertheless, in Appendix A.1 we prove the following

**Proposition** **13.** *For all $\left(\beta_{A},\beta_{H},\alpha_{A},\alpha_{H},\lambda \right)\in P_{\mathrm{SP},4a}\times R\backslash \left[0,1\right]$ there exist parameters $p_{\lambda }^{L}>\alpha_{\lambda }$ (not necessarily satisfying $p_{\lambda }^{L}\geq 0$) and $0<q_{\lambda }^{L}<\beta_{\lambda }=\beta_{•}<\min\left\{1,e^{\beta_{•}-1}\right\}=e^{\beta_{•}-1}$ such that (35) holds for all x∈[0,∞[ and such that for all initial population sizes $X_{0}\in N$ the parts (c) and (d) of Proposition 12 hold true.*

### 3.22. Lower Bounds for the Cases $\left(\beta_{A},\beta_{H},\alpha_{A},\alpha_{H},\lambda \right)\in P_{\mathrm{SP},4b}\times \left(R\backslash \left[0,1\right]\right)$

By recalling $P_{\mathrm{SP},4b}:=\left\{\;\left(\beta_{A},\beta_{H},\alpha_{A},\alpha_{H}\right)\in P_{\mathrm{SP}}\;:\;\alpha_{A}\neq \alpha_{H}>0,\;\beta_{A}=\beta_{H}\in \;[1,\infty [\;\right\}$ (cf.(49)), the assertions preceding Proposition 13 remain valid. However, the proof of Proposition 13 in Appendix A.1 contains details which explain why it cannot be carried over to the current case $P_{\mathrm{SP},4b}$. Thus, the generally valid lower bound $B_{\lambda ,X_{0},n}^{L}\equiv 1$ cannot be improved with our methods.

### 3.23. Concluding Remarks on Alternative Lower Bounds for all Cases $\left(\beta_{A},\beta_{H},\alpha_{A},\alpha_{H},\lambda \right)\in \left(P_{\mathrm{SP}}\backslash P_{\mathrm{SP},1}\right)\times \left(R\backslash \left[0,1\right]\right)$

To achieve the Goals (G1${}^{\prime }$) to (G3${}^{\prime }$), in the above-mentioned investigations about lower bounds of the Hellinger integral $H_{\lambda }(P_{A,n}∥P_{H,n})$, λ∈R\[0,1], we have mainly focused on parameters $p_{\lambda }^{L},\;q_{\lambda }^{L}$ which satisfy (35) and additionally (56). Nevertheless, Theorem 1 (b) gives lower bounds $B_{\lambda ,X_{0},n}^{L}$*whenever* (35) is fulfilled. However, this lower bound can be the trivial one, $B_{\lambda ,X_{0},n}^{L}\equiv 1$. Let us remark here that for the parameter constellations $\left(\beta_{A},\beta_{H},\alpha_{A},\alpha_{H},\lambda \right)\in \left(P_{\mathrm{SP},2}\times R\backslash \left(\left[0,1\right]\cup I_{\mathrm{SP},2}\right)\right)\cup \left(P_{\mathrm{SP},3a}\times R\backslash \left(\left[0,1\right]\cup I_{\mathrm{SP},3a}\right)\right)\cup \left(P_{\mathrm{SP},3b}\times R\backslash \left(\left[0,1\right]\cup I_{\mathrm{SP},3b}\right)\right)$ one can prove that there exist $p_{\lambda }^{L},\;q_{\lambda }^{L}$ which satisfy (35) for all $x\in N_{0}$ as well as the condition (generalizing (56))

$$p_{\lambda }^{L}\;\geq \;\alpha_{\lambda }\;,\;q_{\lambda }^{L}\;\geq \;\beta_{\lambda }\;,\;\left(\mathrm{where}\;\mathrm{at}\;\mathrm{least}\;\mathrm{one}\;\mathrm{of}\;\mathrm{the}\;\mathrm{inequalities}\;\mathrm{is}\;\mathrm{strict}\;\right)\;,$$

and that for such $p_{\lambda }^{L},\;q_{\lambda }^{L}$ one gets the validity of $H_{\lambda }(P_{A,n}∥P_{H,n})\geq B_{\lambda ,X_{0},n}^{L}={\tilde{B}}_{\lambda ,X_{0},n}^{\left(p_{\lambda }^{L},q_{\lambda }^{L}\right)}>1$ for all $X_{0}\in N$ and all n∈N; consequently, Goal (G1${}^{\prime }$) is achieved. However, in these parameter constellations it can unpleasantly happen that $n\mapsto B_{\lambda ,X_{0},n}^{L}$ is oscillating (in contrast to the monotone behaviour in the Propositions 11 (b), 12 (b)).

As a final general remark, let us mention that the functions $\phi_{\lambda ,y}^{\tan}\left(\cdot \right)$, $\phi_{\lambda ,k}^{\sec}\left(\cdot \right)$, $\phi_{\lambda }^{\mathrm{hor}}\left(\cdot \right)$, $\tilde{\phi_{\lambda }}\left(\cdot \right)$ –defined in (52)–(54) and Properties 3 (P20)–constitute linear lower bounds for $\phi_{\lambda }\left(\cdot \right)$ on the domain $N_{0}$ in the case λ∈R\[0,1]. Their parameters $p_{\lambda }^{L}\in \left\{p_{\lambda ,y}^{\tan},p_{\lambda ,y}^{\sec},p_{\lambda ,y}^{\mathrm{hor}},\tilde{p_{\lambda }}\right\}$ and $q_{\lambda }^{L}\in \left\{q_{\lambda ,y}^{\tan},q_{\lambda ,y}^{\sec},q_{\lambda ,y}^{\mathrm{hor}},\tilde{q_{\lambda }}\right\}$ lead to lower bounds $B_{\lambda ,X_{0},n}^{L}$ of the Hellinger integrals that may or may not be consistent with Goals (G1${}^{\prime }$) to (G3${}^{\prime }$), and which may be possibly better respectively weaker respectively incomparable with the previous lower bounds when adding some relaxation of (G1${}^{\prime }$), such as e.g., the validity of $H_{\lambda }(P_{A,n}∥P_{H,n})>1$ for all but finitely many n∈N.

### 3.24. Upper Bounds for the Cases $\left(\beta_{A},\beta_{H},\alpha_{A},\alpha_{H},\lambda \right)\in \left(P_{\mathrm{SP}}\backslash P_{\mathrm{SP},1}\right)\times \left(R\backslash \left[0,1\right]\right)$

For the cases λ∈R\[0,1], the investigation of upper bounds for the Hellinger integral $H_{\lambda }(P_{A,n}∥P_{H,n})$ is much easier than the above-mentioned derivations of lower bounds. In fact, we face a situation which is similar to the lower-bounds-studies for the cases λ∈]0,1[: due to Properties 3 (P19), the function $\phi_{\lambda }\left(\cdot \right)$ is strictly convex on the nonnegative real line. Furthermore, it is asymptotically linear, as stated in (P20). The monotonicity Properties 2 (P10) to (P12) imply that for the tightest upper bound (within our framework) one should use the parameters $p_{\lambda }^{U}:=\alpha_{A}^{\lambda }\alpha_{H}^{1-\lambda }>0$ and $q_{\lambda }^{U}:=\beta_{A}^{\lambda }\beta_{H}^{1-\lambda }>0$. Lemma A1 states that $p_{\lambda }^{U}\geq \alpha_{\lambda }$ resp. $q_{\lambda }^{U}\geq \beta_{\lambda }$, with equality iff $\alpha_{A}=\alpha_{H}$ resp. iff $\beta_{A}=\beta_{H}$. From Properties 1 (P3a) we see that for $\beta_{A}\neq \beta_{H}$ the corresponding sequence ${\left(a_{n}^{\left(q_{\lambda }^{U}\right)}\right)}_{n\in N}$ is convergent to $x_{0}^{\left(q_{\lambda }^{U}\right)}\in \;]0,-\log\left(q_{\lambda }^{U}\right)]$ if $q_{\lambda }^{U}\leq \min\left\{1\;,\;e^{\beta_{\lambda }-1}\right\}$ (i.e., if $\lambda \in [\lambda_{-},\lambda_{+}]$, cf. Lemma 1 (a)), and otherwise it diverges to *∞* faster than exponentially (cf. (P3b)). If $\beta_{A}=\beta_{H}$ (i.e., if $\left(\beta_{A},\beta_{H},\alpha_{A},\alpha_{H}\right)\in P_{\mathrm{SP},4}=P_{\mathrm{SP},4a}\cup P_{\mathrm{SP},4b}$), then one gets $q_{\lambda }^{U}=\beta_{\lambda }$ and $a_{n}^{\left(q_{\lambda }^{U}\right)}=0=x_{0}^{\left(q_{\lambda }^{U}\right)}$ for all n∈N (cf. (P2)). Altogether, this leads to

**Proposition** **14.** *For all $\left(\beta_{A},\beta_{H},\alpha_{A},\alpha_{H},\lambda \right)\in \left(P_{\mathrm{SP}}\backslash P_{\mathrm{SP},1}\right)\times \left(R\backslash \left[0,1\right]\right)$ and all initial population sizes $X_{0}\in N$ there holds with $p_{\lambda }^{U}:=\alpha_{A}^{\lambda }\alpha_{H}^{1-\lambda },\;q_{\lambda }^{U}:=\beta_{A}^{\lambda }\beta_{H}^{1-\lambda }$*

$$\begin{array}{cc} \left(a\right) & \;B_{\lambda ,X_{0},1}^{U}\;=\;{\tilde{B}}_{\lambda ,X_{0},1}^{\left(p_{\lambda }^{U},q_{\lambda }^{U}\right)}\;=\;\exp\left\{\left(\beta_{A}^{\lambda }\beta_{H}^{1-\lambda }-\beta_{\lambda }\right)\cdot X_{0}\;+\;\alpha_{A}^{\lambda }\alpha_{H}^{1-\lambda }-\alpha_{\lambda }\right\}\;>\;1,\;\;\\ \left(b\right) & \;\mathrm{the}\;\mathrm{sequence}{\left(B_{\lambda ,X_{0},n}^{U}\right)}_{n\in N}\;\mathrm{of}\;\mathrm{upper}\;\mathrm{bounds}\;\mathrm{for}\;H_{\lambda }(P_{A,n}∥P_{H,n})\;\mathrm{given}\;\mathrm{by}\\ & \;B_{\lambda ,X_{0},n}^{U}\;=\;{\tilde{B}}_{\lambda ,X_{0},n}^{\left(p_{\lambda }^{U},q_{\lambda }^{U}\right)}\;=\;\exp\left\{a_{n}^{\left(q_{\lambda }^{U}\right)}\cdot X_{0}\;+\;\sum_{k=1}^{n}b_{k}^{\left(p_{\lambda }^{U},q_{\lambda }^{U}\right)}\right\}\\ & \;\mathrm{is}\;\mathrm{strictly}\;\mathrm{increasing},\\ \left(c\right) & \;\lim_{n\to \infty }B_{\lambda ,X_{0},n}^{U}\;=\;\infty \;,\\ \left(d\right) & \lim_{n\to \infty }\;\frac{1}{n}\;\log B_{\lambda ,X_{0},n}^{U}\;=\;\left\{\begin{array}{cc} p_{\lambda }^{U}\cdot \exp\left\{x_{0}^{\left(q_{\lambda }^{U}\right)}\right\}-\alpha_{\lambda }\;>\;0,\; & \mathrm{if}\;\lambda \in \left[\lambda_{-},\lambda_{+}\right]\;\backslash \;\left[0,1\right]\;,\\ \infty , & \mathrm{if}\;\lambda \in \;]-\infty ,\lambda_{-}\left[\;\cup \;\right]\lambda_{+},\infty [\;, \end{array}\right.\\ \left(e\right) & \;\mathrm{the}\;\mathrm{map}\;X_{0}\;\mapsto \;B_{\lambda ,X_{0},n}^{U}={\tilde{B}}_{\lambda ,X_{0},n}^{\left(p_{\lambda }^{U},q_{\lambda }^{U}\right)}\;\mathrm{is}\;\mathrm{strictly}\;\mathrm{increasing}. \end{array}$$

## 4. Power Divergences of Non-Kullback-Leibler-Information-Divergence Type

### 4.1. A First Basic Result

For orders λ∈R\{0,1}, all the results of the previous Section 3 carry correspondingly over from the Hellinger integrals $H_{\lambda }\left(\cdot ∥\cdot \right)$ to the total variation distance V(·||·), by virtue of the relation (cf. (12))

$$2\;\left(1-H_{\frac{1}{2}}(P_{A,n}∥P_{H,n})\right)\;\leq \;V(P_{A,n}∥P_{H,n})\;\leq \;2\;\sqrt{1-{\left(H_{\frac{1}{2}}(P_{A,n}∥P_{H,n})\right)}^{2}}\;,$$

to the Renyi divergences $R_{\lambda }\left(\cdot ∥\cdot \right)$, by virtue of the relation (cf. (7))

$$0\leq \;R_{\lambda }\left(P_{A,n}∥P_{H,n}\right)\;=\;\frac{1}{\lambda (\lambda -1)}\;\log H_{\lambda }\left(P_{A,n}∥P_{H,n}\right)\;,\;\mathrm{with}\log\;0:=-\infty ,$$

as well as to the power divergences $I_{\lambda }\left(\cdot ∥\cdot \right)$, by virtue of the relation (cf. (2))

$$I_{\lambda }\left(P_{A,n}∥P_{H,n}\right)\;=\;\frac{1-H_{\lambda }(P_{A,n}∥P_{H,n})}{\lambda \cdot (1-\lambda )}\;,\;n\in N\;;$$

in the following, we concentrate on the latter. In particular, the above-mentioned carrying-over procedure leads to bounds on $I_{\lambda }\left(P_{A}∥P_{H}\right)$ which are tighter than the general rudimentary bounds (cf. (10) and (11))

$$0\;\leq \;I_{\lambda }\left(P_{A,n}∥P_{H,n}\right)\;<\;\frac{1}{\lambda (1-\lambda )},\;\mathrm{for}\;\lambda \in \;]0,1[\;,\;0\;\leq \;I_{\lambda }\left(P_{A,n}∥P_{H,n}\right)\;\leq \;\infty ,\;\mathrm{for}\;\lambda \in R\backslash \left[0,1\right]\;.$$

Because power divergences have a *very insightful interpretation* as “directed distances” between two probability distributions (e.g., within our running-example epidemiological context), and function as important tools in statistics, information theory, machine learning, and artificial intelligence, we present explicitly the outcoming exact values respectively bounds of $I_{\lambda }\left(P_{A}∥P_{H}\right)$ (λ∈R\{0,1}, n∈N), in the current and the following subsections. For this, recall the case-dependent parameters $p^{A}=p_{\lambda }^{A}=p^{A}\left(\beta_{A},\beta_{H},\alpha_{A},\alpha_{H},\lambda \right)$ and $q^{A}=q_{\lambda }^{A}=q^{A}\left(\beta_{A},\beta_{H},\alpha_{A},\alpha_{H},\lambda \right)$ (A∈{E,L,U}). To begin with, we can deduce from Theorem 1

**Theorem** **2.**

*(a)*  *For all $\left(\beta_{A},\beta_{H},\alpha_{A},\alpha_{H}\right)\in \left(P_{\mathrm{NI}}\cup P_{\mathrm{SP},1}\right)$, all initial population sizes $X_{0}\in N_{0}$, all observation horizons n∈N and all λ∈R\{0,1} one can recursively compute the **exact value***
  (65) $$I_{\lambda }(P_{A,n}∥P_{H,n})\;=\;\frac{1}{\lambda (\lambda -1)}\cdot \left[\exp\left\{a_{n}^{\left(q_{\lambda }^{E}\right)}\cdot X_{0}\;+\;\frac{\alpha_{A}}{\beta_{A}}\;\sum_{k=1}^{n}a_{k}^{\left(q_{\lambda }^{E}\right)}\right\}\;-\;1\right]\;=:\;V_{\lambda ,X_{0},n}^{I}\;,$$
  *where $\frac{\alpha_{A}}{\beta_{A}}$ can be equivalently replaced by $\frac{\alpha_{H}}{\beta_{H}}$ and $q_{\lambda }^{E}:=\beta_{A}^{\lambda }\;\beta_{H}^{1-\lambda }$. Notice that on $P_{\mathrm{NI}}$ the formula (65) simplifies significantly, since $\alpha_{A}=\alpha_{H}=0$.*
*(b)*  *For general parameters p∈R, q≠0 recall the general expression (cf. (42))*
  $${\tilde{B}}_{\lambda ,X_{0},n}^{\left(p,q\right)}\;:=\;\exp\left\{a_{n}^{\left(q\right)}\cdot X_{0}\;+\;\frac{p}{q}\sum_{k=1}^{n}a_{k}^{\left(q\right)}\;+\;n\cdot \left(\frac{p}{q}\beta_{\lambda }-\alpha_{\lambda }\right)\right\}$$
  *as well as*
  $${\tilde{B}}_{\lambda ,X_{0},n}^{\left(p,0\right)}\;:=\;\exp\left\{-\beta_{\lambda }\cdot X_{0}\;+\;\left(p\cdot e^{-\beta_{\lambda }}-\alpha_{\lambda }\right)\cdot n\right\}\;.$$
  *Then, for all $\left(\beta_{A},\beta_{H},\alpha_{A},\alpha_{H}\right)\in P_{\mathrm{SP}}\backslash P_{\mathrm{SP},1}$, all λ∈R\{0,1}, all coefficients $p_{\lambda }^{L},\;p_{\lambda }^{U},\;q_{\lambda }^{L},\;q_{\lambda }^{U}\in R$ which satisfy (35) for all $x\in N_{0}$, all initial population sizes $X_{0}\in N$ and all observation horizons n∈N one gets the following recursive bounds for the power divergences: for λ∈]0,1[ there holds*
  $$I_{\lambda }(P_{A,n}∥P_{H,n})\;\left\{\begin{array}{cc} < & \frac{1}{\lambda (1-\lambda )}\cdot \left(1-B_{\lambda ,X_{0},n}^{L}\right)\;=\;\frac{1}{\lambda (1-\lambda )}\cdot \left(1-{\tilde{B}}_{\lambda ,X_{0},n}^{\left(p_{\lambda }^{L},q_{\lambda }^{L}\right)}\right)\;=:\;B_{\lambda ,X_{0},n}^{I,U}\;,\\ \; & \;\\ \geq & \frac{1}{\lambda (1-\lambda )}\cdot \left(1-B_{\lambda ,X_{0},n}^{U}\right)\;=\;\frac{1}{\lambda (1-\lambda )}\cdot \left(1-\min\left\{{\tilde{B}}_{\lambda ,X_{0},n}^{\left(p_{\lambda }^{U},q_{\lambda }^{U}\right)}\;,\;1\right\}\right)\;=:\;B_{\lambda ,X_{0},n}^{I,L}\;, \end{array}\right.$$
  *whereas for λ∈R\[0,1] there holds*
  $$I_{\lambda }(P_{A,n}∥P_{H,n})\;\left\{\begin{array}{cc} < & \frac{1}{\lambda (\lambda -1)}\cdot \left(B_{\lambda ,X_{0},n}^{U}-1\right)\;=\;\frac{1}{\lambda (\lambda -1)}\cdot \left({\tilde{B}}_{\lambda ,X_{0},n}^{\left(p_{\lambda }^{U},q_{\lambda }^{U}\right)}-1\right)\;=:\;B_{\lambda ,X_{0},n}^{I,U}\;,\\ \; & \;\\ \geq & \frac{1}{\lambda (\lambda -1)}\cdot \left(B_{\lambda ,X_{0},n}^{L}-1\right)\;=\;\frac{1}{\lambda (\lambda -1)}\cdot \left(\max\left\{{\tilde{B}}_{\lambda ,X_{0},n}^{\left(p_{\lambda }^{L},q_{\lambda }^{L}\right)}\;,\;1\right\}-1\right)\;=:\;B_{\lambda ,X_{0},n}^{I,L}\;. \end{array}\right.$$

In order to deduce the subsequent *detailed* recursive analyses of power divergences, we also employ the obvious relations

(66) $$\begin{array}{cc} & \lim_{n\to \infty }\frac{1}{n}\log\left(\frac{1}{\lambda (1-\lambda )}-I_{\lambda }(P_{A,n}∥P_{H,n})\right)\;=\;\lim_{n\to \infty }\frac{1}{n}\left[-\log\left(\lambda (1-\lambda )\right)+\log\left(H_{\lambda }(P_{A,n}∥P_{H,n})\right)\right]\\ = & \lim_{n\to \infty }\frac{1}{n}\log\left(H_{\lambda }(P_{A,n}∥P_{H,n})\right)\;,\;\mathrm{for}\;\lambda \in ]0,1[\;, \end{array}$$

as well as

(67) $$\begin{array}{cc} & \lim_{n\to \infty }\frac{1}{n}\log\left(I_{\lambda }(P_{A,n}∥P_{H,n})\right)\;=\;\lim_{n\to \infty }\frac{1}{n}\left[-\log\left(\lambda (\lambda -1)\right)+\log\left(H_{\lambda }(P_{A,n}∥P_{H,n})-1\right)\right]\\ = & \lim_{n\to \infty }\frac{1}{n}\left[\log\left(1-\frac{1}{H_{\lambda }(P_{A,n}\left|\right|P_{H,n})}\right)+\log\left(H_{\lambda }(P_{A,n}∥P_{H,n})\right)\right]\;=\;\lim_{n\to \infty }\frac{1}{n}\log\left(H_{\lambda }(P_{A,n}∥P_{H,n})\right)\;, \end{array}$$

for λ∈R\[0,1] (provided that ${lim inf}_{n\to \infty }H_{\lambda }(P_{A,n}∥P_{H,n})>1$).

### 4.2. Detailed Analyses of the Exact Recursive Values of $I_{\lambda }\left(\cdot ∥\cdot \right)$, i.e., for the Cases $\left(\beta_{A},\beta_{H},\alpha_{A},\alpha_{H},\lambda \right)\in \left(P_{\mathrm{NI}}\cup P_{\mathrm{SP},1}\right)\times \left(R\backslash \left\{0,1\right\}\right)$

**Corollary** **2.** *For all $\left(\beta_{A},\beta_{H},\alpha_{A},\alpha_{H},\lambda \right)\in P_{\mathrm{NI}}\times ]0,1[$ and all initial population sizes $X_{0}\in N$ there holds with $q_{\lambda }^{E}:=\beta_{A}^{\lambda }\;\beta_{H}^{1-\lambda }$*

$$\begin{array}{cc} \left(a\right) & \;I_{\lambda }(P_{A,1}∥P_{H,1})\;=\;\frac{1}{\lambda (1-\lambda )}\cdot \left(1-\exp\left\{\left(\beta_{A}^{\lambda }\;\beta_{H}^{1-\lambda }-\beta_{\lambda }\right)\cdot X_{0}\right\}\right)\;>\;0\;,\\ \left(b\right) & \;\mathrm{the}\;\mathrm{sequence}{\left(I_{\lambda }(P_{A,n}∥P_{H,n})\right)}_{n\in N}\;\mathrm{given}\;\mathrm{by}\\ & \;I_{\lambda }(P_{A,n}∥P_{H,n})\;=\;\frac{1}{\lambda (1-\lambda )}\cdot \left(1-\exp\left\{a_{n}^{\left(q_{\lambda }^{E}\right)}\cdot X_{0}\right\}\right)\;=:\;V_{\lambda ,X_{0},n}^{I}\\ & \;\mathrm{is}\;\mathrm{strictly}\;\mathrm{increasing},\\ \left(c\right) & \;\lim_{n\to \infty }\;I_{\lambda }(P_{A,n}∥P_{H,n})\;=\;\frac{1}{\lambda (1-\lambda )}\cdot \left(1-\exp\left\{x_{0}^{\left(q_{\lambda }^{E}\right)}\cdot X_{0}\right\}\right)\in \;]0,\frac{1}{\lambda (1-\lambda )}[\;,\\ \left(d\right) & \;\lim_{n\to \infty }\frac{1}{n}\log\left(\frac{1}{\lambda (1-\lambda )}-I_{\lambda }(P_{A,n}∥P_{H,n})\right)\;=\;\lim_{n\to \infty }\;\frac{1}{n}\;\log H_{\lambda }(P_{A,n}∥P_{H,n})\;=\;0\;,\\ \left(e\right) & \;\mathrm{the}\;\mathrm{map}\;X_{0}\;\mapsto \;V_{\lambda ,X_{0},n}^{I}\;\mathrm{is}\;\mathrm{strictly}\;\mathrm{increasing}. \end{array}$$

**Corollary** **3.** *For all $\left(\beta_{A},\beta_{H},\alpha_{A},\alpha_{H},\lambda \right)\in P_{\mathrm{NI}}\times \left(R\backslash \left[0,1\right]\right)$ and all initial population sizes $X_{0}\in N$ there holds with $q_{\lambda }^{E}:=\beta_{A}^{\lambda }\;\beta_{H}^{1-\lambda }$*

$$\begin{array}{cc} \left(a\right) & \;I_{\lambda }(P_{A,1}∥P_{H,1})\;=\;\frac{1}{\lambda (\lambda -1)}\cdot \left(\exp\left\{\left(\beta_{A}^{\lambda }\;\beta_{H}^{1-\lambda }-\beta_{\lambda }\right)\cdot X_{0}\right\}-1\right)\;>\;0\;,\\ \left(b\right) & \;\mathrm{the}\;\mathrm{sequence}{\left(I_{\lambda }(P_{A,n}∥P_{H,n})\right)}_{n\in N}\;\mathrm{given}\;\mathrm{by}\\ & \;I_{\lambda }(P_{A,n}∥P_{H,n})\;=\;\frac{1}{\lambda (\lambda -1)}\cdot \left(\exp\left\{a_{n}^{\left(q_{\lambda }^{E}\right)}\cdot X_{0}\right\}-1\right)\;=:\;V_{\lambda ,X_{0},n}^{I}\\ & \;\mathrm{is}\;\mathrm{strictly}\;\mathrm{increasing},\\ \left(c\right) & \;\lim_{n\to \infty }\;I_{\lambda }(P_{A,n}∥P_{H,n})\;=\;\left\{\begin{array}{cc} \frac{1}{\lambda (\lambda -1)}\cdot \left(\exp\left\{x_{0}^{\left(q_{\lambda }^{E}\right)}\cdot X_{0}\right\}-1\right)>0, & \mathrm{if}\;\lambda \in \left[\lambda_{-},\lambda_{+}\right]\;\backslash \;\left[0,1\right]\;,\\ \infty , & \mathrm{if}\;\lambda \in \;]-\infty ,\lambda_{-}\left[\;\cup \;\right]\lambda_{+},\infty [\;, \end{array}\right.\\ \left(d\right) & \;\lim_{n\to \infty }\;\frac{1}{n}\;\log I_{\lambda }(P_{A,n}∥P_{H,n})\;=\;\left\{\begin{array}{cc} 0, & \mathrm{if}\;\lambda \in \left[\lambda_{-},\lambda_{+}\right]\;\backslash \;\left[0,1\right],\\ \infty , & \mathrm{if}\;\lambda \in \;]-\infty ,\lambda_{-}\left[\;\cup \;\right]\lambda_{+},\infty [\;, \end{array}\right.\\ \left(e\right) & \;\mathrm{the}\;\mathrm{map}\;X_{0}\;\mapsto \;V_{\lambda ,X_{0},n}^{I}\;\mathrm{is}\;\mathrm{strictly}\;\mathrm{increasing}. \end{array}$$

**Corollary** **4.** *For all $\left(\beta_{A},\beta_{H},\alpha_{A},\alpha_{H},\lambda \right)\in P_{\mathrm{SP},1}\times ]0,1[$ and all initial population sizes $X_{0}\in N$ there holds with $q_{\lambda }^{E}:=\beta_{A}^{\lambda }\;\beta_{H}^{1-\lambda }$*

$$\begin{array}{cc} \left(a\right) & \;I_{\lambda }(P_{A,1}∥P_{H,1})\;=\;\frac{1}{\lambda (1-\lambda )}\cdot \left(1-\exp\left\{\left(\beta_{A}^{\lambda }\beta_{H}^{1-\lambda }-\beta_{\lambda }\right)\cdot \left(X_{0}+\frac{\alpha_{A}}{\beta_{A}}\right)\right\}\right)\;>\;0\;,\\ \left(b\right) & \;\mathrm{the}\;\mathrm{sequence}{\left(I_{\lambda }(P_{A,n}∥P_{H,n})\right)}_{n\in N}\;\mathrm{given}\;\mathrm{by}\\ & \;I_{\lambda }(P_{A,n}∥P_{H,n})\;=\;\frac{1}{\lambda (1-\lambda )}\cdot \left(1-\exp\left\{a_{n}^{\left(q_{\lambda }^{E}\right)}\cdot X_{0}+\frac{\alpha_{A}}{\beta_{A}}\sum_{k=1}^{n}a_{k}^{\left(q_{\lambda }^{E}\right)}\right\}\right)\;=:\;V_{\lambda ,X_{0},n}^{I}\\ & \;\mathrm{is}\;\mathrm{strictly}\;\mathrm{increasing},\\ \left(c\right) & \;\lim_{n\to \infty }\;I_{\lambda }(P_{A,n}∥P_{H,n})\;=\;\frac{1}{\lambda (1-\lambda )}\;,\\ \left(d\right) & \;\lim_{n\to \infty }\frac{1}{n}\log\left(\frac{1}{\lambda (1-\lambda )}-I_{\lambda }(P_{A,n}∥P_{H,n})\right)\;=\;\frac{\alpha_{A}}{\beta_{A}}\cdot x_{0}^{\left(q_{\lambda }^{E}\right)}\;<\;0\;,\\ \left(e\right) & \;\mathrm{the}\;\mathrm{map}\;X_{0}\;\mapsto \;V_{\lambda ,X_{0},n}^{I}\;\mathrm{is}\;\mathrm{strictly}\;\mathrm{increasing}. \end{array}$$

**Corollary** **5.** *For all $\left(\beta_{A},\beta_{H},\alpha_{A},\alpha_{H},\lambda \right)\in P_{\mathrm{SP},1}\times \left(R\backslash \left[0,1\right]\right)$ and all initial population sizes $X_{0}\in N$ there holds with $q_{\lambda }^{E}:=\beta_{A}^{\lambda }\;\beta_{H}^{1-\lambda }$*

$$\begin{array}{cc} \left(a\right) & \;I_{\lambda }(P_{A,1}∥P_{H,1})\;=\;\frac{1}{\lambda (\lambda -1)}\cdot \left(\exp\left\{\left(\beta_{A}^{\lambda }\beta_{H}^{1-\lambda }-\beta_{\lambda }\right)\cdot \left(X_{0}+\frac{\alpha_{A}}{\beta_{A}}\right)\right\}-1\right)\;>\;0,\\ \left(b\right) & \;\mathrm{the}\;\mathrm{sequence}{\left(I_{\lambda }(P_{A,n}∥P_{H,n})\right)}_{n\in N}\;\mathrm{given}\;\mathrm{by}\\ & \;I_{\lambda }(P_{A,n}∥P_{H,n})\;=\;\frac{1}{\lambda (\lambda -1)}\cdot \left(\exp\left\{a_{n}^{\left(q_{\lambda }^{E}\right)}\cdot X_{0}+\frac{\alpha_{A}}{\beta_{A}}\sum_{k=1}^{n}a_{k}^{\left(q_{\lambda }^{E}\right)}\right\}-1\right)\;=:\;V_{\lambda ,X_{0},n}^{I}\\ & \;\mathrm{is}\;\mathrm{strictly}\;\mathrm{increasing},\\ \left(c\right) & \;\lim_{n\to \infty }\;I_{\lambda }(P_{A,n}∥P_{H,n})\;=\;\infty ,\\ \left(d\right) & \;\lim_{n\to \infty }\;\frac{1}{n}\;\log I_{\lambda }(P_{A,n}∥P_{H,n})\;=\;\left\{\begin{array}{cc} \frac{\alpha_{A}}{\beta_{A}}\cdot x_{0}^{\left(q_{\lambda }^{E}\right)}\;>0, & \mathrm{if}\;\lambda \in \left[\lambda_{-},\lambda_{+}\right]\;\backslash \;\left[0,1\right]\;,\;\\ \infty , & \mathrm{if}\;\lambda \in \;]-\infty ,\lambda_{-}\left[\;\cup \;\right]\lambda_{+},\infty [\;, \end{array}\right.\;\\ \left(e\right) & \;\mathrm{the}\;\mathrm{map}\;X_{0}\;\mapsto \;V_{\lambda ,X_{0},n}^{I}\;\mathrm{is}\;\mathrm{strictly}\;\mathrm{increasing}. \end{array}$$

In the assertions (a), (b), (d) of the Corollaries 4 and 5 the fraction $\alpha_{A}/\beta_{A}$ can be equivalently replaced by $\alpha_{H}/\beta_{H}$.

Let us now derive the corresponding detailed results for the bounds of the power divergences for the parameter cases $P_{\mathrm{SP}}\backslash P_{\mathrm{SP},1}$, where the Hellinger integral, and thus $I_{\lambda }(P_{A,n}∥P_{H,n})$, cannot be determined exactly. The extensive discussion on the Hellinger-integral bounds in the Section 3.4, Section 3.5, Section 3.6, Section 3.7, Section 3.8, Section 3.9, Section 3.10, Section 3.11, Section 3.12 and Section 3.13, as well as in the Section 3.16, Section 3.17, Section 3.18, Section 3.19, Section 3.20, Section 3.21, Section 3.22, Section 3.23 and Section 3.24 can be carried over directly to obtain power-divergence bounds. In the following, we summarize the outcoming key results, referring a detailed discussion on the possible choices of $p_{\lambda }^{A}=p^{A}\left(\beta_{A},\beta_{H},\alpha_{A},\alpha_{H},\lambda \right)$ and $q_{\lambda }^{A}=q^{A}\left(\beta_{A},\beta_{H},\alpha_{A},\alpha_{H},\lambda \right)$ (A∈{L,U}) to the corresponding above-mentioned subsections.

### 4.3. Lower Bounds of $I_{\lambda }\left(\cdot ∥\cdot \right)$ for the Cases $\left(\beta_{A},\beta_{H},\alpha_{A},\alpha_{H},\lambda \right)\in \left(P_{\mathrm{SP}}\backslash P_{\mathrm{SP},1}\right)\times ]0,1[$

**Corollary** **6.** *For all $\left(\beta_{A},\beta_{H},\alpha_{A},\alpha_{H},\lambda \right)\in \left(P_{\mathrm{SP},2}\cup P_{\mathrm{SP},3a}\cup P_{\mathrm{SP},3b}\right)\times ]0,1[$ there exist parameters $p_{\lambda }^{U},\;q_{\lambda }^{U}$ which satisfy $p_{\lambda }^{U}\in \left[\alpha_{A}^{\lambda }\alpha_{H}^{1-\lambda },\alpha_{\lambda }\right]$ and $q_{\lambda }^{U}\in [\beta_{A}^{\lambda }\beta_{H}^{1-\lambda },\beta_{\lambda }[$ as well as (35) for all $x\in N_{0}$, and for all such pairs $\left(p_{\lambda }^{U},q_{\lambda }^{U}\right)$ and all initial population sizes $X_{0}\in N$ there holds*

$$\begin{array}{cc} \left(a\right) & \;B_{\lambda ,X_{0},1}^{I,L}\;=\;\frac{1}{\lambda (1-\lambda )}\cdot \left(1-\exp\left\{\left(q_{\lambda }^{U}-\beta_{\lambda }\right)\cdot X_{0}\;+\;p_{\lambda }^{U}-\alpha_{\lambda }\right\}\right)\;>\;0,\;\;\\ \left(b\right) & \;\mathrm{the}\;\mathrm{sequence}{\left(B_{\lambda ,X_{0},n}^{I,L}\right)}_{n\in N}\;\mathrm{of}\;\mathrm{lower}\;\mathrm{bounds}\;\mathrm{for}\;I_{\lambda }(P_{A,n}∥P_{H,n})\;\mathrm{given}\;\mathrm{by}\\ & \;B_{\lambda ,X_{0},n}^{I,L}\;=\;\frac{1}{\lambda (1-\lambda )}\cdot \left(1-\exp\left\{a_{n}^{\left(q_{\lambda }^{U}\right)}\cdot X_{0}\;+\;\sum_{k=1}^{n}b_{k}^{\left(p_{\lambda }^{U},q_{\lambda }^{U}\right)}\right\}\right)\\ & \;\mathrm{is}\;\mathrm{strictly}\;\mathrm{increasing},\\ \left(c\right) & \;\lim_{n\to \infty }B_{\lambda ,X_{0},n}^{I,L}\;=\;\lim_{n\to \infty }I_{\lambda }(P_{A,n}∥P_{H,n})\;=\;\frac{1}{\lambda (1-\lambda )}\;,\\ \left(d\right) & \;\lim_{n\to \infty }\;\frac{1}{n}\;\log\left(\frac{1}{\lambda (1-\lambda )}-B_{\lambda ,X_{0},n}^{I,L}\right)\;=\;p_{\lambda }^{U}\cdot e^{x_{0}^{\left(q_{\lambda }^{U}\right)}}-\alpha_{\lambda }\;<\;0\;,\\ \left(e\right) & \;\mathrm{the}\;\mathrm{map}\;X_{0}\;\mapsto \;B_{\lambda ,X_{0},n}^{I,L}\;\mathrm{is}\;\mathrm{strictly}\;\mathrm{increasing}. \end{array}$$

**Remark** **4.**

*(a)*  *Notice that in the case $\left(\beta_{A},\beta_{H},\alpha_{A},\alpha_{H},\lambda \right)\in P_{\mathrm{SP},2}\times \;]0,1[$–where $\alpha_{A}^{\lambda }\alpha_{H}^{1-\lambda }=\alpha_{\lambda }=\alpha_{A}=\alpha_{H}=\alpha$–we get the special choice $p_{\lambda }^{U}=\alpha$ and $q_{\lambda }^{U}={\left(\alpha +\beta_{A}\right)}^{\lambda }{\left(\alpha +\beta_{H}\right)}^{1-\lambda }-\alpha$ (cf. Section 3.7). For the constellations $\left(\beta_{A},\beta_{H},\alpha_{A},\alpha_{H},\lambda \right)\in \left(P_{\mathrm{SP},3a}\cup P_{\mathrm{SP},3b}\right)\times ]0,1[$ there exist parameters $p_{\lambda }^{U}\in [\alpha_{A}^{\lambda }\alpha_{H}^{1-\lambda },\alpha_{\lambda }[$, $q_{\lambda }^{U}\in [\beta_{A}^{\lambda }\beta_{H}^{1-\lambda },\beta_{\lambda }[$ which satisfy (35) for all $x\in N_{0}$.*
*(b)*  *For the parameter setups $\left(\beta_{A},\beta_{H},\alpha_{A},\alpha_{H},\lambda \right)\in \left(P_{\mathrm{SP},2}\cup P_{\mathrm{SP},3a}\cup P_{\mathrm{SP},3b}\right)\times ]0,1[$ there might exist parameter pairs $\left(p_{\lambda }^{U},q_{\lambda }^{U}\right)$ satisfying (35) and either $p_{\lambda }^{U}=\alpha_{\lambda }$ or $q_{\lambda }^{U}=\beta_{\lambda }$, for which all assertions of Corollary 6 still hold true.*
*(c)*  *Following the discussion in Section 3.10 for all $\left(\beta_{A},\beta_{H},\alpha_{A},\alpha_{H},\lambda \right)\in P_{\mathrm{SP},3c}\times ]0,1[$ at least part (c) still holds true.*

**Corollary** **7.** *For all $\left(\beta_{A},\beta_{H},\alpha_{A},\alpha_{H},\lambda \right)\in P_{\mathrm{SP},4a}\times ]0,1[$ there exist parameters $p_{\lambda }^{U}<\alpha_{\lambda }$, $1>q_{\lambda }^{U}>\beta_{\lambda }=\beta$ such that (35) is satisfied for all x∈[0,∞[ and such that for all initial population sizes $X_{0}\in N$ at least the parts (c) and (d) of Corollary 6 hold true.*

As in Section 3.12, for the parameter setup $\left(\beta_{A},\beta_{H},\alpha_{A},\alpha_{H},\lambda \right)\in P_{\mathrm{SP},4b}\times ]0,1[$ we cannot derive a lower bound for the power divergences which improves the generally valid lower bound $I_{\lambda }(P_{A,n}∥P_{H,n})\geq 0$ (cf. (10)) by employing our proposed ($p_{\lambda }^{U},q_{\lambda }^{U}$)-method.

### 4.4. Upper Bounds of $I_{\lambda }\left(\cdot ∥\cdot \right)$ for the Cases $\left(\beta_{A},\beta_{H},\alpha_{A},\alpha_{H},\lambda \right)\in \left(P_{\mathrm{SP}}\backslash P_{\mathrm{SP},1}\right)\times ]0,1[$

Since in this setup the upper bounds of the power divergences can be derived from the lower bounds of the Hellinger integrals, we here appropriately adapt the results of Proposition 6.

**Corollary** **8.** *For all $\left(\beta_{A},\beta_{H},\alpha_{A},\alpha_{H},\lambda \right)\in \left(P_{\mathrm{SP}}\backslash P_{\mathrm{SP},1}\right)\times ]0,1[$ and all initial population sizes $X_{0}\in N$ there holds with $p_{\lambda }^{L}:=\alpha_{A}^{\lambda }\alpha_{H}^{1-\lambda }$ and $q_{\lambda }^{L}:=\beta_{A}^{\lambda }\beta_{H}^{1-\lambda }$*

$$\begin{array}{cc} \left(a\right) & \;B_{\lambda ,X_{0},1}^{I,U}\;=\;\frac{1}{\lambda (1-\lambda )}\cdot \left(1-\exp\left\{\left(\beta_{A}^{\lambda }\;\beta_{H}^{1-\lambda }-\beta_{\lambda }\right)\cdot X_{0}\;+\;\alpha_{A}^{\lambda }\alpha_{H}^{1-\lambda }-\alpha_{\lambda }\right\}\right)\;>\;0,\\ \left(b\right) & \;\mathrm{the}\;\mathrm{sequence}\;\mathrm{of}\;\mathrm{upper}\;\mathrm{bounds}{\left(B_{\lambda ,X_{0},n}^{I,U}\right)}_{n\in N}\;\mathrm{for}\;I_{\lambda }(P_{A,n}∥P_{H,n})\;\mathrm{given}\;\mathrm{by}\\ & \;B_{\lambda ,X_{0},n}^{I,U}\;=\;\frac{1}{\lambda (1-\lambda )}\cdot \left(1-\exp\left\{a_{n}^{\left(q_{\lambda }^{L}\right)}\cdot X_{0}+\frac{p_{\lambda }^{L}}{q_{\lambda }^{L}}\sum_{k=1}^{n}a_{k}^{\left(q_{\lambda }^{L}\right)}+n\cdot \left(\frac{p_{\lambda }^{L}}{q_{\lambda }^{L}}\cdot \beta_{\lambda }-\alpha_{\lambda }\right)\right\}\right)\\ & \;\mathrm{is}\;\mathrm{strictly}\;\mathrm{increasing},\\ \left(c\right) & \;\lim_{n\to \infty }B_{\lambda ,X_{0},n}^{I,U}\;=\;\frac{1}{\lambda (1-\lambda )}\;,\\ \left(d\right) & \;\lim_{n\to \infty }\;\frac{1}{n}\;\log\left(\frac{1}{\lambda (1-\lambda )}-B_{\lambda ,X_{0},n}^{I,U}\right)\;=\;\frac{p_{\lambda }^{L}}{q_{\lambda }^{L}}\cdot \left(x_{0}^{\left(q_{\lambda }^{L}\right)}+\beta_{\lambda }\right)-\alpha_{\lambda }\;=\;p_{\lambda }^{L}\cdot e^{x_{0}^{\left(q_{\lambda }^{L}\right)}}-\alpha_{\lambda }\;<\;0\;,\\ \left(e\right) & \;\mathrm{the}\;\mathrm{map}\;X_{0}\;\mapsto \;B_{\lambda ,X_{0},n}^{I,U}\;\mathrm{is}\;\mathrm{strictly}\;\mathrm{increasing}.\; \end{array}$$

### 4.5. Lower Bounds of $I_{\lambda }\left(\cdot ∥\cdot \right)$ for the Cases $\left(\beta_{\;A},\beta_{\;H},\alpha_{\;A},\alpha_{\;H},\lambda \right)\;\in \;\left(P_{\mathrm{SP}}\;\backslash P_{\mathrm{SP},1}\right)\;\times \;\left(R\backslash \;\left[0,\;1\right]\right)$

In order to derive detailed results on lower bounds of the power divergences in the case λ∈R\[0,1], we have to subsume and adapt the Hellinger-integral concerning lower-bounds investigations from the Section 3.16, Section 3.17, Section 3.18, Section 3.19, Section 3.20, Section 3.21, Section 3.22 and Section 3.23. Recall the λ-sets $I_{\mathrm{SP},2},\;I_{\mathrm{SP},3a},\;I_{\mathrm{SP},3b}$ (cf. (58), (60), (62)). For the constellations $P_{\mathrm{SP},2}\times I_{\mathrm{SP},2}$ we employ the special choice $p_{\lambda }^{L}=\alpha_{A}^{\lambda }\alpha_{H}^{1-\lambda }=\alpha_{\lambda }=\alpha_{A}=\alpha_{H}=\alpha$ together with $q_{\lambda }^{L}={\left(\alpha +\beta_{A}\right)}^{\lambda }{\left(\alpha +\beta_{H}\right)}^{1-\lambda }-\alpha >\max\left\{0,\beta_{\lambda }\right\}$ (cf. (58)) which satisfy (35) for all $x\in N_{0}$ and (56), whereas for the constellations $\left(P_{\mathrm{SP},3a}\times I_{\mathrm{SP},3a}\right)$ $\cup (P_{\mathrm{SP},3b}\times I_{\mathrm{SP},3b})$ we have proved the existence of parameters $p_{\lambda }^{L},\;q_{\lambda }^{L}$ satisfying both (35) for all $x\in N_{0}$ and (56) with two strict inequalities. Subsuming this, we obtain

**Corollary** **9.** *For all $\left(\beta_{A},\beta_{H},\alpha_{A},\alpha_{H},\lambda \right)\in \left(P_{\mathrm{SP},2}\times I_{\mathrm{SP},2}\right)$$\cup (P_{\mathrm{SP},3a}\times I_{\mathrm{SP},3a})$$\cup (P_{\mathrm{SP},3b}\times I_{\mathrm{SP},3b})$ there exist parameters $p_{\lambda }^{L},\;q_{\lambda }^{L}$ which satisfy $\max\left\{0,\alpha_{\lambda }\right\}\leq p_{\lambda }^{L}\leq \alpha_{A}^{\lambda }\alpha_{H}^{1-\lambda },\;\max\left\{0,\beta_{\lambda }\right\}<q_{\lambda }^{L}\leq \beta_{A}^{\lambda }\beta_{H}^{1-\lambda }$ as well as (35) for all $x\in N_{0}$, and for all such pairs $\left(p_{\lambda }^{L},\;q_{\lambda }^{L}\right)$ and all initial population sizes $X_{0}\in N$ one gets*

$$\begin{array}{cc} \left(a\right) & \;B_{\lambda ,X_{0},1}^{I,L}\;=\;\frac{1}{\lambda (\lambda -1)}\cdot \left(\exp\left\{\left(q_{\lambda }^{L}-\beta_{\lambda }\right)\cdot X_{0}\;+\;p_{\lambda }^{L}-\alpha_{\lambda }\right\}-1\right)\;>\;0,\;\;\\ \left(b\right) & \;\mathrm{the}\;\mathrm{sequence}{\left(B_{\lambda ,X_{0},n}^{I,L}\right)}_{n\in N}\;\mathrm{of}\;\mathrm{lower}\;\mathrm{bounds}\;\mathrm{for}\;I_{\lambda }(P_{A,n}∥P_{H,n})\;\mathrm{given}\;\mathrm{by}\\ & \;B_{\lambda ,X_{0},n}^{I,L}\;=\;\frac{1}{\lambda (\lambda -1)}\cdot \left(\exp\left\{a_{n}^{\left(q_{\lambda }^{L}\right)}\cdot X_{0}\;+\;\sum_{k=1}^{n}b_{k}^{\left(p_{\lambda }^{L},q_{\lambda }^{L}\right)}\right\}-1\right)\\ & \;\mathrm{is}\;\mathrm{strictly}\;\mathrm{increasing},\\ \left(c\right) & \;\lim_{n\to \infty }B_{\lambda ,X_{0},n}^{I,L}\;=\;\lim_{n\to \infty }I_{\lambda }(P_{A,n}∥P_{H,n})\;=\;\infty \;,\;\\ \left(d\right) & \;\lim_{n\to \infty }\;\frac{1}{n}\;\log B_{\lambda ,X_{0},n}^{I,L}\;=\;\left\{\begin{array}{cc} p_{\lambda }^{L}\cdot \exp\left\{x_{0}^{\left(q_{\lambda }^{L}\right)}\right\}-\alpha_{\lambda }\;>\;0, & \mathrm{if}\;q_{\lambda }^{L}\;\leq \;\min\left\{1;e^{\beta_{\lambda }-1}\right\},\\ \infty , & \mathrm{if}\;q_{\lambda }^{L}\;>\;\min\left\{1;e^{\beta_{\lambda }-1}\right\}, \end{array}\right.\\ \left(e\right) & \;\mathrm{the}\;\mathrm{map}\;X_{0}\;\mapsto \;B_{\lambda ,X_{0},n}^{I,L}\;\mathrm{is}\;\mathrm{strictly}\;\mathrm{increasing}. \end{array}$$

Analogously to the discussions in the Section 3.17, Section 3.18, Section 3.19 and Section 3.20, for the parameter setups $\left(P_{\mathrm{SP},2}\times R\backslash \left(I_{\mathrm{SP},2}\cup \left[0,1\right]\right)\right)$ $\cup \;\left(P_{\mathrm{SP},3a}\times R\backslash \left(I_{\mathrm{SP},3a}\cup \left[0,1\right]\right)\right)\;\cup \;\left(P_{\mathrm{SP},3b}\times R\backslash \left(I_{\mathrm{SP},3b}\cup \left[0,1\right]\right)\right)\;\cup \;\left(P_{\mathrm{SP},3c}\times R\backslash \left[0,1\right]\right)$ and for all initial population sizes $X_{0}\in N$ one can still show

$$0\;<\;I_{\lambda }(P_{A,n}∥P_{H,n})\;,\;\mathrm{and}\;\lim_{n\to \infty }I_{\lambda }(P_{A,n}∥P_{H,n})\;=\;\infty \;.$$

For the penultimate case we obtain

**Corollary** **10.** *For all $\left(\beta_{A},\beta_{H},\alpha_{A},\alpha_{H},\lambda \right)\in P_{\mathrm{SP},4a}\times \left(R\backslash \left[0,1\right]\right)$ there exist parameters $p_{\lambda }^{L}>\alpha_{\lambda }$ (where not necessarily $p_{\lambda }^{L}\geq 0$) and $0<q_{\lambda }^{L}<\beta_{\lambda }=\beta_{•}<\min\left\{1,e^{\beta_{•}-1}\right\}=e^{\beta_{•}-1}$ such that (35) is satisfied for all x∈[0,∞[ and such that for all initial population sizes $X_{0}\in N$ at least the parts (c) and (d) of Corollary 9 hold true.*

Notice that for the last case $\left(\beta_{A},\beta_{H},\alpha_{A},\alpha_{H},\lambda \right)\in P_{\mathrm{SP},4b}\times R\backslash \left[0,1\right]$ (where ($\beta_{A}=\beta_{H}\geq 1$) we cannot derive lower bounds of the power divergences which improve the generally valid lower bound $I_{\lambda }(P_{A,n}∥P_{H,n})\geq 0$ (cf. (11)) by employing our proposed ($p_{\lambda }^{U},q_{\lambda }^{U}$)-method.

### 4.6. Upper Bounds of $I_{\lambda }\left(\cdot ∥\cdot \right)$ for the Cases $\left(\beta_{A},\beta_{H},\alpha_{A},\alpha_{H},\lambda \right)\in \left(P_{\mathrm{SP}}\backslash P_{\mathrm{SP},1}\right)\times \left(R\backslash \left[0,1\right]\right)$

For these constellations we adapt Proposition 14, which after modulation becomes

**Corollary** **11.** *For all $\left(\beta_{A},\beta_{H},\alpha_{A},\alpha_{H},\lambda \right)\in \left(P_{\mathrm{SP}}\backslash P_{\mathrm{SP},1}\right)\times \left(R\backslash \left[0,1\right]\right)$ and all initial population sizes $X_{0}\in N$ there holds with $p_{\lambda }^{U}:=\alpha_{A}^{\lambda }\alpha_{H}^{1-\lambda }$ and $q_{\lambda }^{U}:=\beta_{A}^{\lambda }\beta_{H}^{1-\lambda }$*

$$\begin{array}{cc} \left(a\right) & \;B_{\lambda ,X_{0},1}^{I,U}\;=\;\frac{1}{\lambda (\lambda -1)}\cdot \left(\exp\left\{\left(\beta_{A}^{\lambda }\beta_{H}^{1-\lambda }-\beta_{\lambda }\right)\cdot X_{0}\;+\;\alpha_{A}^{\lambda }\alpha_{H}^{1-\lambda }-\alpha_{\lambda }\right\}-1\right)\;>\;0,\;\;\\ \left(b\right) & \;\mathrm{the}\;\mathrm{sequence}{\left(B_{\lambda ,X_{0},n}^{I,U}\right)}_{n\in N}\;\mathrm{of}\;\mathrm{upper}\;\mathrm{bounds}\;\mathrm{for}\;I_{\lambda }(P_{A,n}∥P_{H,n})\;\mathrm{given}\;\mathrm{by}\\ & \;B_{\lambda ,X_{0},n}^{I,U}\;=\;\frac{1}{\lambda (\lambda -1)}\cdot \left(\exp\left\{a_{n}^{\left(q_{\lambda }^{U}\right)}\cdot X_{0}\;+\;\sum_{k=1}^{n}b_{k}^{\left(p_{\lambda }^{U},q_{\lambda }^{U}\right)}\right\}-1\right)\\ & \;\mathrm{is}\;\mathrm{strictly}\;\mathrm{increasing},\\ \left(c\right) & \;\lim_{n\to \infty }B_{\lambda ,X_{0},n}^{I,U}\;=\;\infty \;,\\ \left(d\right) & \;\lim_{n\to \infty }\;\frac{1}{n}\;\log B_{\lambda ,X_{0},n}^{I,U}\;=\;\left\{\begin{array}{cc} p_{\lambda }^{U}\cdot \exp\left\{x_{0}^{\left(q_{\lambda }^{U}\right)}\right\}-\alpha_{\lambda }\;\;>\;0, & \mathrm{if}\;\lambda \in \left[\lambda_{-},\lambda_{+}\right]\;\backslash \;\left[0,1\right]\;,\\ \infty , & \mathrm{if}\;\lambda \in \;]-\infty ,\lambda_{-}\left[\;\cup \;\right]\lambda_{+},\infty [\;, \end{array}\right.\\ \left(e\right) & \;\mathrm{the}\;\mathrm{map}\;X_{0}\;\mapsto \;B_{\lambda ,X_{0},n}^{I,U}\;\mathrm{is}\;\mathrm{strictly}\;\mathrm{increasing}. \end{array}$$

### 4.7. Applications to Bayesian Decision Making

As explained in Section 2.5, the power divergences fulfill

$$I_{\lambda }\left(P_{A,n}∥P_{H,n}\right)\;=\;\int_{0}^{1}\Delta {\mathrm{BR}}_{\tilde{\mathrm{LO}}}\left(p_{A}^{\mathrm{prior}}\right)\cdot {\left(1-p_{A}^{\mathrm{prior}}\right)}^{\lambda -2}\cdot {\left(p_{A}^{\mathrm{prior}}\right)}^{-1-\lambda }\;dp_{A}^{\mathrm{prior}},\;\lambda \in R,\;\left(\mathrm{cf}.(21)\right),$$

and

$$I_{\lambda }\left(P_{A,n}∥P_{H,n}\right)\;=\;\lim_{\chi \to p_{A}^{\mathrm{prior}}}\;\Delta {\mathrm{BR}}_{{\mathrm{LO}}_{\lambda ,\chi }}\left(p_{A}^{\mathrm{prior}}\right),\;\lambda \in \;]0,1[,\;\left(\mathrm{cf}.(22)\right),$$

and thus can be interpreted as (i) *weighted-average* decision risk reduction (weighted-average statistical information measure) about the degree of evidence deg concerning the parameter θ that can be attained by observing the GWI-path $X_{n}$ until stage *n*, and as (ii) *limit* decision risk reduction (limit statistical information measure). Hence, by combining (21) and (22) with the investigations in the previous Section 4.1, Section 4.2, Section 4.3, Section 4.4, Section 4.5 and Section 4.6, we obtain exact recursive values respectively recursive bounds of the above-mentioned decision risk reductions. For the sake of brevity, we omit the details here.

## 5. Kullback-Leibler Information Divergence (Relative Entropy)

### 5.1. Exact Values Respectively Upper Bounds of I(·||·)

From (2), (3) and (6) in Section 2.4, one can immediately see that the Kullback-Leibler information divergence (relative entropy) between two competing Galton-Watson processes without/with immigration can be obtained by the limit

(68) $$I(P_{A,n}∥P_{H,n})\;=\;\lim_{\lambda ↗1}\;I_{\lambda }\left(P_{A,n}∥P_{H,n}\right)\;,$$

and the reverse Kullback-Leibler information divergence (reverse relative entropy) by $I\left(P_{H,n}∥P_{A,n}\right)=\lim_{\lambda ↘0}I_{\lambda }\left(P_{A,n}∥P_{H,n}\right)$. Hence, in the following we concentrate only on (68), the reverse case works analogously. Accordingly, we can use (68) in appropriate combination with the λ∈]0,1[-parts of the previous Section 4 (respectively, the corresponding parts of Section 3) in order to obtain detailed analyses for $I\left(P_{H,n}∥P_{A,n}\right)$. Let us start with the following assertions on exact values respectively upper bounds, which will be proved in Appendix A.2:

**Theorem** **3.**

*(a)*  *For all $\left(\beta_{A},\beta_{H},\alpha_{A},\alpha_{H}\right)\in \left(P_{\mathrm{NI}}\cup P_{\mathrm{SP},1}\right)$, all initial population sizes $X_{0}\in N$ and all observation horizons n∈N the Kullback-Leibler information divergence (relative entropy) is given by*
  (69) $$I(P_{A,n}∥P_{H,n})\;=\;I_{X_{0},n}\;:=\;\left\{\begin{array}{cc} \frac{\beta_{A}\cdot \left(\log\left(\frac{\beta_{A}}{\beta_{H}}\right)-1\right)+\beta_{H}}{1-\beta_{A}}\cdot \left[X_{0}-\frac{\alpha_{A}}{1-\beta_{A}}\right]\cdot \left(1-{\left(\beta_{A}\right)}^{n}\right) & \;\\ \;+\;\frac{\alpha_{A}\cdot \left[\beta_{A}\cdot \left(\log\left(\frac{\beta_{A}}{\beta_{H}}\right)-1\right)+\beta_{H}\right]}{\beta_{A}\left(1-\beta_{A}\right)}\cdot n\;, & \mathrm{if}\;\beta_{A}\neq 1,\\ \; & \;\\ \left[\beta_{H}-\log\beta_{H}-1\right]\cdot \left[\frac{\alpha_{A}}{2}\cdot n^{2}+\left(X_{0}+\frac{\alpha_{A}}{2}\right)\cdot n\right]\;, & \mathrm{if}\;\beta_{A}=1. \end{array}\right.$$
*(b)*  *For all $\left(\beta_{A},\beta_{H},\alpha_{A},\alpha_{H}\right)\in P_{\mathrm{SP}}\backslash P_{\mathrm{SP},1}$, all initial population sizes $X_{0}\in N$ and all observation horizons n∈N there holds $\;I(P_{A,n}∥P_{H,n})\;\leq \;E_{X_{0},n}^{U}$, where*
  (70) $$E_{X_{0},n}^{U}:=\left\{\begin{array}{cc} \frac{\beta_{A}\cdot \left(\log\left(\frac{\beta_{A}}{\beta_{H}}\right)-1\right)+\beta_{H}}{1-\beta_{A}}\cdot \left[X_{0}-\frac{\alpha_{A}}{1-\beta_{A}}\right]\cdot \left(1-{\left(\beta_{A}\right)}^{n}\right) & \;\\ \;+\;\left[\frac{\alpha_{A}\cdot \left[\beta_{A}\cdot \left(\log\left(\frac{\beta_{A}}{\beta_{H}}\right)-1\right)+\beta_{H}\right]}{\beta_{A}\left(1-\beta_{A}\right)}+\alpha_{A}\left[\log\left(\frac{\alpha_{A}\beta_{H}}{\alpha_{H}\beta_{A}}\right)-\frac{\beta_{H}}{\beta_{A}}\right]+\alpha_{H}\right]\cdot n\;, & \mathrm{if}\;\beta_{A}\neq 1,\\ \; & \;\\ \left[\beta_{H}-\log\beta_{H}-1\right]\cdot \left[\frac{\alpha_{A}}{2}\cdot n^{2}+\left(X_{0}+\frac{\alpha_{A}}{2}\right)\cdot n\right]\\ +\;\left[\alpha_{A}\left[\log\left(\frac{\alpha_{A}\beta_{H}}{\alpha_{H}}\right)-\beta_{H}\right]+\alpha_{H}\right]\cdot n\;, & \mathrm{if}\;\beta_{A}=1. \end{array}\right.$$

**Remark** **5.** *(i) Notice that the exact values respectively upper bounds are in* closed form *(rather than in recursive form).*

*(ii) The n–behaviour of (the bounds of) the Kullback-Leibler information divergence/relative entropy $I(P_{A,n}∥P_{H,n})$ in Theorem 3 is influenced by the following facts:*

*(a)*  *$\beta_{A}\cdot \left(\log\left(\frac{\beta_{A}}{\beta_{H}}\right)-1\right)+\beta_{H}\geq 0$ with equality iff $\beta_{A}=\beta_{H}$.*
*(b)*  *In the case $\beta_{A}\neq 1$ of (70), there holds $\frac{\alpha_{A}\cdot \left[\beta_{A}\cdot \left(\log\left(\frac{\beta_{A}}{\beta_{H}}\right)-1\right)+\beta_{H}\right]}{\beta_{A}\left(1-\beta_{A}\right)}+\alpha_{A}\left[\log\left(\frac{\alpha_{A}\beta_{H}}{\alpha_{H}\beta_{A}}\right)-\frac{\beta_{H}}{\beta_{A}}\right]+\alpha_{H}\geq 0$, with equality iff $\alpha_{A}=\alpha_{H}$ and $\beta_{A}=\beta_{H}$.*

### 5.2. Lower Bounds of I(·||·) for the Cases $\left(\beta_{A},\beta_{H},\alpha_{A},\alpha_{H}\right)\in \left(P_{\mathrm{SP}}\backslash P_{\mathrm{SP},1}\right)$

Again by using (68) in appropriate combination with the “λ∈]0,1[-parts” of the previous Section 4 (respectively, the corresponding parts of Section 3), we obtain the following *(semi-)closed-form* lower bounds of $I\left(P_{H,n}∥P_{A,n}\right)$:

**Theorem** **4.** *For all $\left(\beta_{A},\beta_{H},\alpha_{A},\alpha_{H}\right)\in P_{\mathrm{SP}}\backslash P_{\mathrm{SP},1}$, all initial population sizes $X_{0}\in N$ and all observation horizons n∈N*

(71) $$I(P_{A,n}∥P_{H,n})\;\geq \;E_{X_{0},n}^{L}\;:=\;\underset{k\in N_{0},\;y\in [0,\infty [}{\sup}\left\{E_{y,X_{0},n}^{L,tan}\;,\;E_{k,X_{0},n}^{L,sec}\;,\;E_{X_{0},n}^{L,hor}\right\}\in [0,\infty [\;,$$

*where for all y∈[0,∞[ we define the – possibly negatively valued– finite bound component*

(72) $$E_{y,X_{0},n}^{L,\tan}\;:=\;\left\{\begin{array}{cc} \left[\beta_{A}\log\left(\frac{\alpha_{A}+\beta_{A}y}{\alpha_{H}+\beta_{H}y}\right)+\beta_{H}\left(1-\frac{\alpha_{A}+\beta_{A}y}{\alpha_{H}+\beta_{H}y}\right)\right]\cdot \frac{1-{\left(\beta_{A}\right)}^{n}}{1-\beta_{A}}\cdot \left[X_{0}-\frac{\alpha_{A}}{1-\beta_{A}}\right]\\ +\;[\frac{\alpha_{A}}{\beta_{A}\left(1-\beta_{A}\right)}\left[\beta_{A}\log\left(\frac{\alpha_{A}+\beta_{A}y}{\alpha_{H}+\beta_{H}y}\right)+\beta_{H}\left(1-\frac{\alpha_{A}+\beta_{A}y}{\alpha_{H}+\beta_{H}y}\right)\right]\\ \;+\;\left(\alpha_{H}-\alpha_{A}\frac{\beta_{H}}{\beta_{A}}\right)\left(1-\frac{\alpha_{A}+\beta_{A}y}{\alpha_{H}+\beta_{H}y}\right)]\cdot n\;, & \mathrm{if}\;\beta_{A}\neq 1,\;\\ \left[\log\left(\frac{\alpha_{A}+y}{\alpha_{H}+\beta_{H}y}\right)+\beta_{H}\left(1-\frac{\alpha_{A}+y}{\alpha_{H}+\beta_{H}y}\right)\right]\cdot \left[\frac{\alpha_{A}}{2}\cdot n^{2}+\left(X_{0}+\frac{\alpha_{A}}{2}\right)\cdot n\right]\\ +\;\left(\alpha_{H}-\alpha_{A}\beta_{H}\right)\left(1-\frac{\alpha_{A}+y}{\alpha_{H}+\beta_{H}y}\right)\cdot n\;, & \mathrm{if}\;\beta_{A}=1, \end{array}\right.$$

*and for all $k\in N_{0}$ the – possibly negatively valued– finite bound component*

(73) $$E_{k,X_{0},n}^{L,\sec}\;:=\;\left\{\begin{array}{cc} \left[f_{A}\left(k+1\right)\log\left(\frac{f_{A}\left(k+1\right)}{f_{H}\left(k+1\right)}\right)-f_{A}\left(k\right)\log\left(\frac{f_{A}\left(k\right)}{f_{H}\left(k\right)}\right)+\beta_{H}-\beta_{A}\right]\cdot \frac{1-{\left(\beta_{A}\right)}^{n}}{1-\beta_{A}}\cdot \left[X_{0}-\frac{\alpha_{A}}{1-\beta_{A}}\right] & \;\\ +\;[\frac{\alpha_{A}}{\beta_{A}\left(1-\beta_{A}\right)}\left(f_{A}\left(k+1\right)\log\left(\frac{f_{A}\left(k+1\right)}{f_{H}\left(k+1\right)}\right)-f_{A}\left(k\right)\log\left(\frac{f_{A}\left(k\right)}{f_{H}\left(k\right)}\right)+\beta_{H}-\beta_{A}\right) & \;\\ \;-\left(f_{A}\left(k+1\right)\log\left(\frac{f_{A}\left(k+1\right)}{f_{H}\left(k+1\right)}\right)-f_{A}\left(k\right)\log\left(\frac{f_{A}\left(k\right)}{f_{H}\left(k\right)}\right)\right)\cdot \left(k+\frac{\alpha_{A}}{\beta_{A}}\right) & \;\\ \;+f_{A}\left(k\right)\log\left(\frac{f_{A}\left(k\right)}{f_{H}\left(k\right)}\right)-\frac{\alpha_{A}\beta_{H}}{\beta_{A}}+\alpha_{H}]\cdot n\;, & \;\mathrm{if}\;\beta_{A}\neq 1,\;\\ \left[f_{A}\left(k+1\right)\log\left(\frac{f_{A}\left(k+1\right)}{f_{H}\left(k+1\right)}\right)-f_{A}\left(k\right)\log\left(\frac{f_{A}\left(k\right)}{f_{H}\left(k\right)}\right)+\beta_{H}-1\right]\cdot \left[\frac{\alpha_{A}}{2}\cdot n^{2}+\left(X_{0}+\frac{\alpha_{A}}{2}\right)\cdot n\right] & \;\\ -[\left(f_{A}\left(k+1\right)\log\left(\frac{f_{A}\left(k+1\right)}{f_{H}\left(k+1\right)}\right)-f_{A}\left(k\right)\log\left(\frac{f_{A}\left(k\right)}{f_{H}\left(k\right)}\right)\right)\left(k+\alpha_{A}\right) & \;\\ \;-f_{A}\left(k\right)\log\left(\frac{f_{A}\left(k\right)}{f_{H}\left(k\right)}\right)+\alpha_{A}\beta_{H}-\alpha_{H}]\cdot n\;, & \;\mathrm{if}\;\beta_{A}=1. \end{array}\right.$$

*Furthermore, on $P_{\mathrm{SP},4}$ we set $E_{X_{0},n}^{L,hor}:=0$ for all n∈N whereas on $P_{\mathrm{SP}}\backslash \left(P_{\mathrm{SP},1}\cup P_{\mathrm{SP},4}\right)$ we define*

(74) $$E_{X_{0},n}^{L,hor}\;:=\;\left[\left(\alpha_{A}+\beta_{A}z^{*}\right)\cdot \left[\log\left(\frac{\alpha_{A}+\beta_{A}z^{*}}{\alpha_{H}+\beta_{H}z^{*}}\right)-1\right]+\alpha_{H}+\beta_{H}z^{*}\right]\cdot n,\;,n\in N,$$

with $z^{*}\;:=\;\arg\max_{x\in N_{0}}\left\{\left(\alpha_{A}+\beta_{A}x\right)\left[-\log\left(\frac{\alpha_{A}+\beta_{A}x}{\alpha_{H}+\beta_{H}x}\right)+1\right]-\left(\alpha_{H}+\beta_{H}x\right)\right\}$.

*On $P_{\mathrm{SP}}\backslash \left(P_{\mathrm{SP},1}\cup P_{\mathrm{SP},3c}\right)$ one even gets $E_{X_{0},n}^{L}>0$ for all $X_{0}\in N$ and all n∈N.*

For the subcase $P_{\mathrm{SP},3c}$, one obtains for each fixed n∈N and each fixed $X_{0}\in N$ the strict positivity $E_{X_{0},n}^{L}>0$ if $\left(\frac{\partial }{\partial y}E_{y,n}^{L,tan}\right)\left(y^{*}\right)\neq 0$, where $y^{*}:=\frac{\alpha_{A}-\alpha_{H}}{\beta_{H}-\beta_{A}}\in N$ and hence

(75) $$\begin{array}{c} \;\left(\frac{\partial }{\partial y}E_{y,X_{0},n}^{L,tan}\right)\left(y^{*}\right)\\ \;=\left\{\begin{array}{cc} \;-\frac{{\left(\beta_{A}-\beta_{H}\right)}^{3}}{\alpha_{A}\beta_{H}-\alpha_{H}\beta_{A}}\cdot \frac{1-{\left(\beta_{A}\right)}^{n}}{1-\beta_{A}}\cdot \left[X_{0}-\frac{\alpha_{A}}{1-\beta_{A}}\right]-\frac{{\left(\beta_{A}-\beta_{H}\right)}^{2}}{\beta_{A}}\left(1+\frac{\alpha_{A}\left(\beta_{A}-\beta_{H}\right)}{\left(1-\beta_{A}\right)\left(\alpha_{A}\beta_{H}-\alpha_{H}\beta_{A}\right)}\right)\cdot n\;, & \mathrm{if}\;\beta_{A}\neq 1,\;\\ -\;\frac{{\left(1-\beta_{H}\right)}^{3}}{\alpha_{A}\beta_{H}-\alpha_{H}}\cdot \left[\frac{\alpha_{A}}{2}\cdot n^{2}+\left(X_{0}+\frac{\alpha_{A}}{2}\right)\cdot n\right]-{\left(1-\beta_{H}\right)}^{2}\cdot n\;, & \mathrm{if}\;\beta_{A}=1. \end{array}\right. \end{array}$$

A proof of this theorem is given in in Appendix A.2.

**Remark** **6.** Consider the exemplary parameter setup $\left(\beta_{A},\beta_{H},\alpha_{A},\alpha_{H}\right)=\left(\frac{1}{3},\frac{2}{3},2,1\right)\in P_{\mathrm{SP},3c}$; within our running-example epidemiological context of Section 2.3, this corresponds to a “semi-mild” infectious-disease-transmission situation (H) (with subcritical reproduction number $\beta_{H}=\frac{2}{3}$ and importation mean of $\alpha_{H}=1$), whereas (A) describes a “mild” situation (with “low” subcritical $\beta_{A}=\frac{1}{3}$ and $\alpha_{A}=2$). In the case of $X_{0}=3$ there holds $\left(\frac{\partial }{\partial y}E_{y,X_{0},n}^{L,tan}\right)\left(y^{*}\right)=0$ for all n∈N, whereas for $X_{0}\neq 3$ one obtains $\left(\frac{\partial }{\partial y}E_{y,X_{0},n}^{L,tan}\right)\left(y^{*}\right)\neq 0$ for all n∈N.

It seems that the optimization problem in (71) admits in general only an implicitly representable solution, and thus we have used the prefix “(semi-)” above. Of course, as a less tight but less involved *explicit* lower bound of the Kullback-Leibler information divergence (relative entropy) $I(P_{A,n}||P_{H,n})$ one can use any term of the form $\max\left\{E_{y,X_{0},n}^{L,tan}\;,\;E_{k,X_{0},n}^{L,sec}\;,\;E_{X_{0},n}^{L,hor}\right\}$ (y∈[0,∞[, $k\in N_{0}$), as well as the following

**Corollary** **12.** *(a) For all $\left(\beta_{A},\beta_{H},\alpha_{A},\alpha_{H}\right)\in P_{\mathrm{SP}}\backslash P_{\mathrm{SP},1}$, all initial population sizes $X_{0}\in N$ and all observation horizons n∈N*

$$I(P_{A,n}∥P_{H,n})\;\geq \;E_{X_{0},n}^{L}\;\geq \;{\tilde{E}}_{X_{0},n}^{L}:=\max\left\{E_{\infty ,X_{0},n}^{L,tan}\;,\;E_{0,X_{0},n}^{L,sec}\;,\;E_{X_{0},n}^{L,hor}\right\}\in [0,\infty [\;,$$

*with $E_{X_{0},n}^{L,hor}$ defined by (74), with – possibly negatively valued– finite bound component $\;E_{\infty ,X_{0},n}^{L,tan}\;:=\;\lim_{y\to \infty }E_{y,X_{0},n}^{L,tan}$, where*

$$E_{\infty ,X_{0},n}^{L,tan}\;:=\;\left\{\begin{array}{cc} \frac{\beta_{A}\cdot \left(\log\left(\frac{\beta_{A}}{\beta_{H}}\right)-1\right)+\beta_{H}}{1-\beta_{A}}\cdot \left[X_{0}-\frac{\alpha_{A}}{1-\beta_{A}}\right]\cdot \left(1-{\left(\beta_{A}\right)}^{n}\right) & \;\\ +\;\left[\frac{\alpha_{A}\cdot \left[\beta_{A}\cdot \left(\log\left(\frac{\beta_{A}}{\beta_{H}}\right)-1\right)+\beta_{H}\right]}{\beta_{A}\left(1-\beta_{A}\right)}+\alpha_{A}\left(1-\frac{\beta_{H}}{\beta_{A}}\right)+\alpha_{H}\left(1-\frac{\beta_{A}}{\beta_{H}}\right)\right]\cdot n\;, & \mathrm{if}\beta_{A}\neq 1,\\ \; & \;\\ \left[\beta_{H}-\log\beta_{H}-1\right]\cdot \left[\frac{\alpha_{A}}{2}\cdot n^{2}+\left(X_{0}+\frac{\alpha_{A}}{2}\right)\cdot n\right]\\ +\;\left[\alpha_{A}\left(1-\beta_{H}\right)+\alpha_{H}\left(1-\frac{1}{\beta_{H}}\right)\right]\cdot n\;, & \mathrm{if}\beta_{A}=1, \end{array}\right.$$

*and –possibly negatively valued–finite bound component*

$$E_{0,X_{0},n}^{L,sec}\;=\;\left\{\begin{array}{cc} \left[\left(\alpha_{A}+\beta_{A}\right)\cdot \log\left(\frac{\alpha_{A}+\beta_{A}}{\alpha_{H}+\beta_{H}}\right)-\alpha_{A}\cdot \log\left(\frac{\alpha_{A}}{\alpha_{H}}\right)+\beta_{H}-\beta_{A}\right]\cdot \frac{1-{\left(\beta_{A}\right)}^{n}}{1-\beta_{A}}\cdot \left[X_{0}-\frac{\alpha_{A}}{1-\beta_{A}}\right] & \;\\ +\{\frac{\alpha_{A}}{\beta_{A}\left(1-\beta_{A}\right)}\left(\left(\alpha_{A}+\beta_{A}\right)\cdot \log\left(\frac{\alpha_{A}+\beta_{A}}{\alpha_{H}+\beta_{H}}\right)-\alpha_{A}\cdot \log\left(\frac{\alpha_{A}}{\alpha_{H}}\right)\right)-\frac{\alpha_{A}}{1-\beta_{A}}\left(1-\beta_{H}\right) & \;\\ \;-\alpha_{A}\left(1+\frac{\alpha_{A}}{\beta_{A}}\right)\cdot \log\left(\frac{\alpha_{H}\left(\alpha_{A}+\beta_{A}\right)}{\alpha_{A}\left(\alpha_{H}+\beta_{H}\right)}\right)+\alpha_{H}\}\cdot n\;, & \;\mathrm{if}\beta_{A}\neq 1,\\ \; & \;\\ \left[\left(\alpha_{A}+1\right)\cdot \log\left(\frac{\alpha_{A}+1}{\alpha_{H}+\beta_{H}}\right)-\alpha_{A}\cdot \log\left(\frac{\alpha_{A}}{\alpha_{H}}\right)+\beta_{H}-1\right]\cdot \left[n\cdot X_{0}+\frac{\alpha_{A}}{2}\cdot n^{2}\right]\\ +\{\frac{\alpha_{A}}{2}\left[\left(\alpha_{A}+1\right)\cdot \log\left(\frac{\alpha_{A}+1}{\alpha_{H}+\beta_{H}}\right)-\alpha_{A}\cdot \log\left(\frac{\alpha_{A}}{\alpha_{H}}\right)-\beta_{H}-1\right] & \;\\ \;-\alpha_{A}\left(1+\alpha_{A}\right)\cdot \log\left(\frac{\alpha_{H}\left(\alpha_{A}+1\right)}{\alpha_{A}\left(\alpha_{H}+\beta_{H}\right)}\right)+\alpha_{H}\}\cdot n\;, & \;\mathrm{if}\beta_{A}=1. \end{array}\right.$$

*For the cases $P_{\mathrm{SP},2}\cup P_{\mathrm{SP},3a}\cup P_{\mathrm{SP},3b}$ one gets even ${\tilde{E}}_{X_{0},n}^{L}>0$ for all $X_{0}\in N$ and all n∈N.*

### 5.3. Applications to Bayesian Decision Making

As explained in Section 2.5, the Kullback-Leibler information divergence fulfills

$$I\left(P_{A,n}∥P_{H,n}\right)\;=\;\int_{0}^{1}\Delta {\mathrm{BR}}_{\tilde{\mathrm{LO}}}\left(p_{A}^{\mathrm{prior}}\right)\cdot {\left(1-p_{A}^{\mathrm{prior}}\right)}^{-1}\cdot {\left(p_{A}^{\mathrm{prior}}\right)}^{-2}\;dp_{A}^{\mathrm{prior}},\;\left(\mathrm{cf}.\;(21)\;\mathrm{with}\;\lambda =1\right),$$

and thus can be interpreted as *weighted-average* decision risk reduction (weighted-average statistical information measure) about the degree of evidence deg concerning the parameter θ that can be attained by observing the GWI-path $X_{n}$ until stage *n*. Hence, by combining (21) with the investigations in the previous Section 5.1 and Section 5.2, we obtain exact values respectively bounds of the above-mentioned decision risk reductions. For the sake of brevity, we omit the details here.

## 6. Explicit Closed-Form Bounds of Hellinger Integrals

### 6.1. Principal Approach

Depending on the parameter constellation $\left(\beta_{A},\beta_{H},\alpha_{A},\alpha_{H},\lambda \right)\in P\times \left(R\backslash \left\{0,1\right\}\right)$, for the Hellinger integrals $H_{\lambda }\left(P_{A,n}∥P_{H,n}\right)$ we have derived in Section 3 corresponding lower/upper bounds respectively exact values–of recursive nature– which can be obtained by choosing appropriate $p=p_{\lambda }^{A}=p^{A}\left(\beta_{A},\beta_{H},\alpha_{A},\alpha_{H},\lambda \right),\;q=q_{\lambda }^{A}=q^{A}\left(\beta_{A},\beta_{H},\alpha_{A},\alpha_{H},\lambda \right)$ (A∈{E,L,U}) and by using those together with the recursion ${\left(a_{n}^{\left(q\right)}\right)}_{n\in N}$ defined by (36) as well as the sequence ${\left(b_{n}^{\left(p,q\right)}\right)}_{n\in N}$ obtained from ${\left(a_{n}^{\left(q\right)}\right)}_{n\in N}$ by the linear transformation (38). Both sequences are “stepwise fully evaluable” but generally seem not to admit a closed-form representation in the observation horizons *n*; consequently, the time-evolution $n\mapsto H_{\lambda }\left(P_{A,n}∥P_{H,n}\right)$–respectively the time-evolution of the corresponding recursive bounds– can generally *not be seen explicitly*. On order to avoid this *intransparency* (at the expense of losing some precision) one can approximate (36) by a recursion that allows for a closed-form representation; by the way, this will also turn out to be useful for investigations concerning diffusion limits (cf. the next Section 7).

To explain the basic underlying principle, let us first assume some *general* $q\in ]0,\beta_{\lambda }[$ and λ∈]0,1[. With Properties 1 (P1) we see that the sequence ${\left(a_{n}^{\left(q\right)}\right)}_{n\in N}$ is strictly negative, strictly decreasing and converges to $x_{0}^{\left(q\right)}\in ]-\beta_{\lambda },q-\beta_{\lambda }[$. Recall that this sequence is obtained by the recursive application of the function $\xi_{\lambda }^{\left(q\right)}\left(x\right):=q\cdot e^{x}-\beta_{\lambda }$, through $a_{1}^{\left(q\right)}=\xi_{\lambda }^{\left(q\right)}\left(0\right)=q-\beta_{\lambda }<0$, $a_{n}^{\left(q\right)}=\xi_{\lambda }^{\left(q\right)}\left(a_{n-1}^{\left(q\right)}\right)=qe^{a_{n-1}^{\left(q\right)}}-\beta_{\lambda }$ (cf. (36)). As a first step, we want to approximate $\xi_{\lambda }^{\left(q\right)}\left(\cdot \right)$ by a linear function on the interval $\left[x_{0}^{\left(q\right)},0\right]$. Due to convexity (P9), this is done by using the tangent line of $\xi_{\lambda }^{\left(q\right)}\left(\cdot \right)$ at $x_{0}^{\left(q\right)}$

(76) $$\xi_{\lambda }^{(q),T}\left(x\right)\;:=\;c^{(q),T}\;+\;d^{(q),T}\cdot x\;:=\;x_{0}^{\left(q\right)}\left(1-q\cdot e^{x_{0}^{\left(q\right)}}\right)\;+\;q\cdot e^{x_{0}^{\left(q\right)}}\cdot x\;,$$

as a linear lower bound, and the secant line of $\xi_{\lambda }^{\left(q\right)}\left(\cdot \right)$ across its arguments 0 and $x_{0}^{\left(q\right)}$

(77) $$\xi_{\lambda }^{(q),S}\left(x\right)\;:=\;c^{(q),S}\;+\;d^{(q),S}\cdot x\;:=\;q-\beta_{\lambda }\;+\;\frac{x_{0}^{\left(q\right)}-\left(q-\beta_{\lambda }\right)}{x_{0}^{\left(q\right)}}\cdot x\;,$$

as a linear upper bound. With the help of these functions, we can define the *linear* recursions

(78) $$\begin{array}{cc} & a_{0}^{(q),T}\;:=\;0\;,\;a_{n}^{(q),T}\;:=\;\xi_{\lambda }^{(q),T}\left(a_{n-1}^{(q),T}\right)\;,\;n\in N\;, \end{array}$$

(79) $$\begin{array}{cc} \mathrm{as}\;\mathrm{well}\;\mathrm{as}\; & a_{0}^{(q),S}\;:=\;0\;,\;a_{n}^{(q),S}\;:=\;\xi_{\lambda }^{(q),S}\left(a_{n-1}^{(q),S}\right)\;,\;n\in N\;.\; \end{array}$$

In the following, we will refer to these sequences as the *rudimentary closed-form sequence-bounds*.

Clearly, both sequences are strictly negative (on N), strictly decreasing, and one gets the sandwiching

(80) $$a_{n}^{(q),T}\;<\;a_{n}^{\left(q\right)}\;\leq \;a_{n}^{(q),S}\;$$

for all n∈N, with equality on the right side iff n=1 (where $a_{1}^{\left(q\right)}=q-\beta_{\lambda }<0$); moreover,

(81) $$\lim_{n\to \infty }a_{n}^{(q),T}\;=\;\lim_{n\to \infty }a_{n}^{(q),S}\;=\;\lim_{n\to \infty }a_{n}^{\left(q\right)}\;=\;x_{0}^{\left(q\right)}\;.$$

Furthermore, such linear recursions allow for a closed-form representation, namely

(82) $$a_{n}^{(q),*}\;=\;\frac{c^{(q),*}}{1-d^{(q),*}}\cdot \left(1-{\left(d^{(q),*}\right)}^{n}\right)\;=\;x_{0}^{\left(q\right)}\cdot \left(1-{\left(d^{(q),*}\right)}^{n}\right)\;,$$

where the “ * ” stands for either *S* or *T*. Notice that this representation is valid due to $d^{(q),T},d^{(q),S}\in ]0,1[$. So far, we have considered the case $q\in ]0,\beta_{\lambda }[$. If $q=\beta_{\lambda }$, then one can see from Properties 1 (P2) that $a_{n}^{\left(q\right)}\equiv 0$, which is also an explicitly given (though trivial) sequence. For the remaining case, where $q>\beta_{\lambda }$ and thus $\xi_{\lambda }^{\left(q\right)}\left(0\right)=a_{1}^{\left(q\right)}=q-\beta_{\lambda }>0$), we want to exclude $q\geq \min\left\{1\;,\;e^{\beta_{\lambda }-1}\right\}$ for the following reasons. Firstly, if $q>\min\left\{1\;,\;e^{\beta_{\lambda }-1}\right\}$, then from (P3) we see that the sequence ${\left(a_{n}^{\left(q\right)}\right)}_{n\in N}$ is strictly increasing and divergent to *∞*, at a rate faster than exponentially (P3b); but a linear recursion is too weak to approximate such a growth pattern. Secondly, if $q=\min\left\{1\;,\;e^{\beta_{\lambda }-1}\right\}$, then one necessarily gets $q=e^{\beta_{\lambda }-1}<1$ (since we have required $q>\beta_{\lambda }$, and otherwise one obtains the contradiction $\beta_{\lambda }<q=1\leq e^{\beta_{\lambda }-1}$). This means that the function $\xi_{\lambda }^{\left(q\right)}\left(\cdot \right)$ now touches the straight line id(·) in the point −log(q), i.e., $\xi_{\lambda }^{\left(q\right)}\left(-\log(q)\right)=-\log\left(q\right)$. Our above-proposed method, namely to use the tangent line of $\xi_{\lambda }^{\left(q\right)}\left(\cdot \right)$ at $x=x_{0}^{\left(q\right)}=-\log\left(q\right)$ as a linear lower bound for $\xi_{\lambda }^{\left(q\right)}\left(\cdot \right)$, leads then to the recursion $a_{n}^{(q),T}\equiv 0$ (cf. (78)). This is due to the fact that the tangent line $\xi_{\lambda }^{(q),T}\left(\cdot \right)$ is in the current case equivalent with the straight line id(·). Consequently, (81) would not be satisfied.

Notice that in the case $\beta_{\lambda }<q<\min\left\{1\;,\;e^{\beta_{\lambda }-1}\right\}$, the above-introduced functions $\xi_{\lambda }^{(q),T}\left(\cdot \right),\;\xi_{\lambda }^{(q),S}\left(\cdot \right)$ constitute again linear lower and upper bounds for $\xi_{\lambda }^{\left(q\right)}\left(\cdot \right)$, however, this time on the interval $\left[0,x_{0}^{\left(q\right)}\right]$. The sequences defined in (78) and (79) still fulfill the assertions (80) and (81), and additionally allow for the closed-form representation (82). Furthermore, let us mention that these rudimentary closed-form sequence-bounds can be defined analogously for λ∈R\[0,1] and either $0<q<\beta_{\lambda }$, or $q=\beta_{\lambda }$, or $\max\left\{0,\beta_{\lambda }\right\}<q<\min\left\{1,e^{\beta_{\lambda }-1}\right\}$.

In a second step, we want to *improve* the above-mentioned linear (lower and upper) approximations of the sequence $a_{n}^{\left(q\right)}$ by reducing the faced error within each iteration. To do so, in both cases of lower and upper approximates we shall employ context-adapted linear *inhomogeneous difference equations* of the form

(83) $$\begin{array}{ccc} {\tilde{a}}_{0}:=\;0 & ; & \;{\tilde{a}}_{n}\;:=\;\tilde{\xi }\left({\tilde{a}}_{n-1}\right)\;+\;\rho_{n-1},\;\;n\in N, \end{array}$$

with

(84) $$\begin{array}{ccc} \tilde{\xi }\left(x\right) & := & c\;+\;d\cdot x\;,\;x\in R\;, \end{array}$$

(85) $$\begin{array}{ccc} \rho_{n-1} & := & K_{1}\cdot \varkappa^{n-1}\;+\;K_{2}\cdot \nu^{n-1}\;,\;n\in N, \end{array}$$

for some constants c∈R, d∈]0,1[, $K_{1},K_{2},\varkappa ,\nu \in R$ with 0≤ν<ϰ≤d. This will be applied to $c:=c^{(q),S}$, $c:=c^{(q),T}$, $d:=d^{(q),S}$ and $d:=d^{(q),T}$ later on. Meanwhile, let us first present some facts and expressions which are insightful for further formulations and analyses.

**Lemma** **2.** *Consider the sequence ${\left({\tilde{a}}_{n}\right)}_{n\in N_{0}}$ defined in (83) to (85). If 0≤ν<ϰ<d, then one gets the* closed-form representation

(86) $${\tilde{a}}_{n}\;=\;{\tilde{a}}_{n}^{hom}+{\tilde{c}}_{n}\;\mathrm{with}\;{\tilde{a}}_{n}^{hom}=c\cdot \frac{1-d^{n}}{1-d}\;\;\;\mathrm{and}\;{\tilde{c}}_{n}=K_{1}\cdot \frac{d^{n}-\varkappa^{n}}{d-\varkappa }\;+\;K_{2}\cdot \frac{d^{n}-\nu^{n}}{d-\nu },$$

*which leads for all n∈N to*

(87) $$\sum_{k=1}^{n}{\tilde{a}}_{k}\;=\;\left(\frac{K_{1}}{d-\varkappa }+\frac{K_{2}}{d-\nu }-\frac{c}{1-d}\right)\cdot \frac{d\cdot \left(1-d^{n}\right)}{1-d}-\frac{K_{1}\cdot \varkappa \cdot \left(1-\varkappa^{n}\right)}{\left(d-\varkappa )(1-\varkappa \right)}-\frac{K_{2}\cdot \nu \cdot \left(1-\nu^{n}\right)}{\left(d-\nu )(1-\nu \right)}+\frac{c}{1-d}\cdot n\;.$$

*If 0≤ν<ϰ=d, then one gets the* closed-form representation

(88) $${\tilde{a}}_{n}\;=\;{\tilde{a}}_{n}^{hom}+{\tilde{c}}_{n}\;\mathrm{with}\;\;{\tilde{a}}_{n}^{hom}=c\cdot \frac{1-d^{n}}{1-d}\;\;\;\mathrm{and}\;{\tilde{c}}_{n}=K_{1}\cdot n\cdot d^{n-1}+K_{2}\cdot \frac{d^{n}-\nu^{n}}{d-\nu },$$

*which leads for all n∈N to*

(89) $$\sum_{k=1}^{n}{\tilde{a}}_{k}\;=\;\left(\frac{K_{1}}{d(1-d)}+\frac{K_{2}}{d-\nu }-\frac{c}{1-d}\right)\cdot \frac{d\cdot \left(1-d^{n}\right)}{1-d}-\frac{K_{2}\cdot \nu \cdot \left(1-\nu^{n}\right)}{\left(d-\nu )(1-\nu \right)}+\left(\frac{c}{1-d}-\frac{K_{1}\cdot d^{n}}{1-d}\right)\cdot n\;.$$

Lemma 2 will be proved in Appendix A.3. Notice that (88) is consistent with taking the limit ϰ↗d in (86). Furthermore, for the special case $K_{2}=-K_{1}>0$ one has from (85) for all integers n≥2 the relation $\rho_{n-1}<0$ and thus ${\tilde{a}}_{n}-{\tilde{a}}_{n}^{hom}<0$, leading to

(90) $${\tilde{c}}_{n}<0\;\mathrm{and}\;\sum_{k=1}^{n}{\tilde{c}}_{n}<0\;.$$

Lemma 2 gives explicit expressions for a linear inhomogeneous recursion of the form (83) possessing the extra term given by (85). Therefrom we derive lower and upper bounds for the sequence ${\left(a_{n}^{\left(q\right)}\right)}_{n\in N}$ by employing $a_{n}^{(q),T}$ resp. $a_{n}^{(q),S}$ as the homogeneous solution of (83), i.e., by setting ${\tilde{a}}_{n}^{hom}:=a_{n}^{(q),T}$ resp. ${\tilde{a}}_{n}^{hom}:=a_{n}^{(q),S}$. Moreover, our concrete approximation-error-reducing “correction terms” $\rho_{n}$ will have different form, depending on whether $0<q<\beta_{\lambda }$ or $q>\max\{0,\beta_{\lambda }\}$. In both cases, we express $\rho_{n}$ by means of the slopes $d^{(q),T}=qe^{x_{0}^{\left(q\right)}}$ resp. $d^{(q),S}=\frac{x_{0}^{\left(q\right)}-\left(q-\beta_{\lambda }\right)}{x_{0}^{\left(q\right)}}$ of the tangent line $\xi_{\lambda }^{(q),T}\left(\cdot \right)$ (cf. (76)) resp. the secant line $\xi_{\lambda }^{(q),S}\left(\cdot \right)$ (cf. (77)), as well as in terms of the parameters

(91) $$\Gamma_{<}^{\left(q\right)}\;:=\;\frac{1}{2}\cdot {\left(x_{0}^{\left(q\right)}\right)}^{2}\cdot q\cdot e^{x_{0}^{\left(q\right)}}\;,\;\mathrm{for}\;0<q<\beta_{\lambda }\;,\;\mathrm{and}\;\Gamma_{>}^{\left(q\right)}\;:=\;\frac{q}{2}\cdot {\left(x_{0}^{\left(q\right)}\right)}^{2}\;,\;\mathrm{for}\;q>\max\left\{0,\beta_{\lambda }\right\}\;.$$

In detail, let us first define the lower approximate by

(92) $${\underline{a}}_{0}^{\left(q\right)}\;:=\;0\;,\;{\underline{a}}_{n}^{\left(q\right)}\;:=\;\xi_{\lambda }^{(q),T}\left({\underline{a}}_{n-1}^{\left(q\right)}\right)\;+\;{\underline{\rho }}_{n-1}^{\left(q\right)}\;,\;n\in N,$$

where

(93) $${\underline{\rho }}_{n-1}^{\left(q\right)}\;:=\;\left\{\begin{array}{cc} \Gamma_{<}^{\left(q\right)}\cdot {\left(d^{(q),T}\right)}^{2(n-1)}, & \mathrm{if}\;0<q<\beta_{\lambda }\;,\\ \; & \;\\ \Gamma_{>}^{\left(q\right)}\cdot {\left(d^{(q),S}\right)}^{2(n-1)}, & \mathrm{if}\;\max\left\{0,\beta_{\lambda }\right\}<q<\min\left\{1,e^{\beta_{\lambda }-1}\right\}\;. \end{array}\right.$$

The upper approximate is defined by

(94) $${\bar{a}}_{0}^{\left(q\right)}\;:=\;0\;,\;{\bar{a}}_{n}^{\left(q\right)}\;:=\;\xi_{\lambda }^{(q),S}\left({\bar{a}}_{n-1}^{\left(q\right)}\right)\;+\;{\bar{\rho }}_{n-1}^{\left(q\right)}\;,\;n\in N,$$

where

(95) $${\bar{\rho }}_{n-1}^{\left(q\right)}\;:=\;\left\{\begin{array}{cc} -\;\Gamma_{<}^{\left(q\right)}\cdot {\left(d^{(q),T}\right)}^{n-1}\cdot \left[1-{\left(d^{(q),S}\right)}^{n-1}\right], & \mathrm{if}\;0<q<\beta_{\lambda }\;,\\ \; & \;\\ -\;\Gamma_{>}^{\left(q\right)}\cdot {\left(d^{(q),S}\right)}^{n-1}\cdot \left[1-{\left(d^{(q),T}\right)}^{n-1}\right], & \mathrm{if}\;\max\left\{0,\beta_{\lambda }\right\}<q<\min\left\{1,e^{\beta_{\lambda }-1}\right\}\;. \end{array}\right.$$

In terms of (85), we use for ${\underline{\rho }}_{n}^{\left(q\right)}$ the constants $K_{2}=\nu =0$ as well as $K_{1}=\Gamma_{<}^{\left(q\right)},\;\varkappa ={\left(d^{(q),T}\right)}^{2}$ for $0<q<\beta_{\lambda }$ respectively $K_{1}=\Gamma_{>}^{\left(q\right)},\;\varkappa ={\left(d^{(q),S}\right)}^{2}$ for $\max\left\{0,\beta_{\lambda }\right\}<q<\min\left\{1,e^{\beta_{\lambda }-1}\right\}$. For ${\bar{\rho }}_{n}^{\left(q\right)}$ we shall employ the constants $-K_{1}=K_{2}=\Gamma_{<}^{\left(q\right)},\;\varkappa =d^{(q),T},\;\nu =d^{(q),S}d^{(q),T}$ for $0<q<\beta_{\lambda }$, and $-K_{1}=K_{2}=\Gamma_{>}^{\left(q\right)},\;\varkappa =d^{(q),S},\;\nu =d^{(q),S}d^{(q),T}$ for $\max\left\{0,\beta_{\lambda }\right\}<q<\min\left\{1,e^{\beta_{\lambda }-1}\right\}$. Recall from (76) the constants $c^{(q),T}:=x_{0}^{\left(q\right)}\left(1-qe^{x_{0}^{\left(q\right)}}\right)$, $d^{(q),T}:=qe^{x_{0}^{\left(q\right)}}$ and from (77) $c^{(q),S}:=q-\beta_{\lambda }$, $d^{(q),S}:=\frac{x_{0}^{\left(q\right)}-\left(q-\beta_{\lambda }\right)}{x_{0}^{\left(q\right)}}$. In the following, we will refer to the sequences ${\underline{a}}_{n}^{\left(q\right)}$ resp. ${\bar{a}}_{n}^{\left(q\right)}$ as the *improved closed-form sequence-bounds*. Putting all ingredients together, we arrive at the

**Lemma** **3.** *For all $\left(\beta_{A},\beta_{H},\alpha_{A},\alpha_{H}\right)\in P$ there holds with $d^{(q),T}=qe^{x_{0}^{\left(q\right)}}$ and $d^{(q),S}=\frac{x_{0}^{\left(q\right)}-\left(q-\beta_{\lambda }\right)}{x_{0}^{\left(q\right)}}$*

*(a)*  *in the case $0<q<\beta_{\lambda }$:*
  *(i)*  $${\underline{a}}_{n}^{\left(q\right)}\;<\;a_{n}^{\left(q\right)}\;\leq \;{\bar{a}}_{n}^{\left(q\right)}\;\mathrm{for}\mathrm{all}\;n\in N,$$
    *with equality on the right-hand side iff n=1, where*
    $$\begin{array}{cc} \; & {\underline{a}}_{n}^{\left(q\right)}=x_{0}^{\left(q\right)}\cdot \left(1-{\left(d^{(q),T}\right)}^{n}\right)\;+\;\Gamma_{<}^{\left(q\right)}\cdot \frac{{\left(d^{(q),T}\right)}^{n-1}}{1-d^{(q),T}}\cdot \left(1-{\left(d^{(q),T}\right)}^{n}\right)\;>\;a_{n}^{(q),T},\;\mathrm{and}\\ \; & {\bar{a}}_{n}^{\left(q\right)}=x_{0}^{\left(q\right)}\cdot \left(1-{\left(d^{(q),S}\right)}^{n}\right)-\Gamma_{<}^{\left(q\right)}\cdot \;\left[\frac{{\left(d^{(q),S}\right)}^{n}-{\left(d^{(q),T}\right)}^{n}}{d^{(q),S}-d^{(q),T}}-{\left(d^{(q),S}\right)}^{n-1}\frac{1-{\left(d^{(q),T}\right)}^{n}}{1-d^{(q),T}}\right]\;\leq a_{n}^{(q),S}, \end{array}$$
    *with $a_{n}^{(q),T}$ and $a_{n}^{(q),S}$ defined by (78) and (79).*
  *(ii)*  *Both sequences ${\left({\underline{a}}_{n}^{\left(q\right)}\right)}_{n\in N}$ and ${\left({\bar{a}}_{n}^{\left(q\right)}\right)}_{n\in N}$ are strictly decreasing.*
  *(iii)*  $$\lim_{n\to \infty }{\underline{a}}_{n}^{\left(q\right)}\;=\;\lim_{n\to \infty }{\bar{a}}_{n}^{\left(q\right)}\;=\;\lim_{n\to \infty }a_{n}^{\left(q\right)}\;=\;x_{0}^{\left(q\right)}\;\in ]-\beta_{\lambda },q-\beta_{\lambda }[.$$
*(b)*  *in the case $\max\left\{0,\beta_{\lambda }\right\}<q<\min\left\{1\;,\;e^{\beta_{\lambda }-1}\right\}$:*
  *(i)*  $${\underline{a}}_{n}^{\left(q\right)}\;<\;a_{n}^{\left(q\right)}\;\leq \;{\bar{a}}_{n}^{\left(q\right)},\;\mathrm{for}\mathrm{all}\;n\in N,$$
    *with equality on the right-hand side iff n=1, where*
    $$\begin{array}{cc} \; & {\underline{a}}_{n}^{\left(q\right)}=x_{0}^{\left(q\right)}\cdot \left(1-{\left(d^{(q),T}\right)}^{n}\right)\;+\;\Gamma_{>}^{\left(q\right)}\cdot \frac{{\left(d^{(q),T}\right)}^{n}-{\left(d^{(q),S}\right)}^{2n}}{d^{(q),T}-{\left(d^{(q),S}\right)}^{2}}\;>\;a_{n}^{(q),T}\;\mathrm{and}\\ \; & {\bar{a}}_{n}^{\left(q\right)}=x_{0}^{\left(q\right)}\cdot \left(1-{\left(d^{(q),S}\right)}^{n}\right)\;-\;\Gamma_{>}^{\left(q\right)}\cdot {\left(d^{(q),S}\right)}^{n-1}\left[n\;-\;\frac{1-{\left(d^{(q),T}\right)}^{n}}{1-d^{(q),T}}\right]\;\leq \;a_{n}^{(q),S},\; \end{array}$$
    *with $a_{n}^{(q),T}$ and $a_{n}^{(q),S}$ defined by (78) and (79).*
  *(ii)*  *Both sequences ${\left({\underline{a}}_{n}^{\left(q\right)}\right)}_{n\in N}$ and ${\left({\bar{a}}_{n}^{\left(q\right)}\right)}_{n\in N}$ are strictly increasing.*
  *(iii)*  $$\lim_{n\to \infty }{\underline{a}}_{n}^{\left(q\right)}\;=\;\lim_{n\to \infty }{\bar{a}}_{n}^{\left(q\right)}\;=\;\lim_{n\to \infty }a_{n}^{\left(q\right)}\;=\;x_{0}^{\left(q\right)}\;\in ]q-\beta_{\lambda },-\log\left(q\right)[.$$

A detailed proof of Lemma 3 is provided in Appendix A.3. In the following, we employ the above-mentioned investigations in order to derive the desired closed-form bounds of the Hellinger integrals $H_{\lambda }(P_{A,n}∥P_{H,n})$.

### 6.2. Explicit Closed-Form Bounds for the Cases $\left(\beta_{A},\beta_{H},\alpha_{A},\alpha_{H},\lambda \right)\in \left(P_{\mathrm{NI}}\cup P_{\mathrm{SP},1}\right)\times \left(R\backslash \left\{0,1\right\}\right)$

Recall that in this setup, we have obtained the recursive, non-explicit *exact* values $V_{\lambda ,X_{0},n}=H_{\lambda }(P_{A,n}∥P_{H,n})$ given in (39) of Theorem 1, where we used $q=q_{\lambda }^{E}=q^{E}\left(\beta_{A},\beta_{H},\lambda \right)=\beta_{A}^{\lambda }\beta_{H}^{1-\lambda }\in ]0,\beta_{\lambda }[$ in the case λ∈]0,1[ respectively $q=q_{\lambda }^{E}=\beta_{A}^{\lambda }\beta_{H}^{1-\lambda }>\max\left\{0,\beta_{\lambda }\right\}$ in the case λ∈R\[0,1]. For the latter, Lemma 1 implies that $q_{\lambda }^{E}<\min\left\{1,e^{\beta_{\lambda }-1}\right\}$ iff $\lambda \in ]\lambda_{-},\lambda_{+}[\;\backslash \left[0,1\right]$. This—together with (39) from Theorem 1, Lemma 2 and with the quantities $d^{(q),T},\;d^{(q),S}$, $\Gamma_{<}^{\left(q\right)}$ and $\Gamma_{>}^{\left(q\right)}$ as defined in (76) and (77) resp. (91) –leads to

**Theorem** **5.** *Let $p_{\lambda }^{E}:=\alpha_{A}^{\lambda }\alpha_{H}^{1-\lambda }$ and $q_{\lambda }^{E}:=\beta_{A}^{\lambda }\beta_{H}^{1-\lambda }$. For all $\left(\beta_{A},\beta_{H},\alpha_{A},\alpha_{H},\lambda \right)\in \left(P_{\mathrm{NI}}\cup P_{\mathrm{SP},1}\right)\times \left(\;]\lambda_{-},\lambda_{+}[\;\backslash \;\left\{0,1\right\}\;\right)$, all initial population sizes $X_{0}\in N$ and for all observation horizons n∈N the following assertions hold true:*

*(a)*  *the Hellinger integral can be bounded by the closed-form lower and upper bounds*
  $$C_{\lambda ,X_{0},n}^{(p_{\lambda }^{E},q_{\lambda }^{E}),T}\;\leq \;C_{\lambda ,X_{0},n}^{(p_{\lambda }^{E},q_{\lambda }^{E}),L}\;\leq \;V_{\lambda ,X_{0},n}\;=\;H_{\lambda }(P_{A,n}∥P_{H,n})\;\leq \;C_{\lambda ,X_{0},n}^{(p_{\lambda }^{E},q_{\lambda }^{E}),U}\;\leq \;C_{\lambda ,X_{0},n}^{(p_{\lambda }^{E},q_{\lambda }^{E}),S}\;,$$
*(b)*  $$\begin{array}{ccc} \lim_{n\to \infty }\frac{1}{n}\log\left(V_{\lambda ,X_{0},n}\right) & = & \lim_{n\to \infty }\frac{1}{n}\log\left(C_{\lambda ,X_{0},n}^{(p_{\lambda }^{E},q_{\lambda }^{E}),L}\right)\;=\;\lim_{n\to \infty }\frac{1}{n}\log\left(C_{\lambda ,X_{0},n}^{(p_{\lambda }^{E},q_{\lambda }^{E}),U}\right)\\ & = & \lim_{n\to \infty }\frac{1}{n}\log\left(C_{\lambda ,X_{0},n}^{(p_{\lambda }^{E},q_{\lambda }^{E}),T}\right)\;=\;\lim_{n\to \infty }\frac{1}{n}\log\left(C_{\lambda ,X_{0},n}^{(p_{\lambda }^{E},q_{\lambda }^{E}),S}\right)\;=\;\frac{\alpha_{A}}{\beta_{A}}\cdot x_{0}^{\left(q_{\lambda }^{E}\right)}\;, \end{array}$$

*where the involved closed-form lower bounds are defined by*

(96) $$\begin{array}{ccc} C_{\lambda ,X_{0},n}^{(p_{\lambda }^{E},q_{\lambda }^{E}),L} & := & C_{\lambda ,X_{0},n}^{(p_{\lambda }^{E},q_{\lambda }^{E}),T}\cdot \exp\left\{{\underline{\zeta }}_{n}^{\left(q_{\lambda }^{E}\right)}\cdot X_{0}\;+\;\frac{\alpha_{A}}{\beta_{A}}\cdot {\underline{\vartheta }}_{n}^{\left(q_{\lambda }^{E}\right)}\right\},\;\mathrm{with}\\ C_{\lambda ,X_{0},n}^{(p_{\lambda }^{E},q_{\lambda }^{E}),T} & := & \exp\left\{x_{0}^{\left(q_{\lambda }^{E}\right)}\cdot \left[X_{0}-\frac{\alpha_{A}}{\beta_{A}}\cdot \frac{d^{(q_{\lambda }^{E}),T}}{1-d^{(q_{\lambda }^{E}),T}}\right]\cdot \left(1-{\left(d^{(q_{\lambda }^{E}),T}\right)}^{n}\right)+\frac{\alpha_{A}}{\beta_{A}}x_{0}^{\left(q_{\lambda }^{E}\right)}\cdot n\right\}, \end{array}$$

*and the closed-form upper bounds are defined by*

(97) $$\begin{array}{ccc} C_{\lambda ,X_{0},n}^{(p_{\lambda }^{E},q_{\lambda }^{E}),U} & := & C_{\lambda ,X_{0},n}^{(p_{\lambda }^{E},q_{\lambda }^{E}),S}\cdot \exp\left\{-\;{\bar{\zeta }}_{n}^{\left(q_{\lambda }^{E}\right)}\cdot X_{0}\;-\;\frac{\alpha_{A}}{\beta_{A}}\cdot {\bar{\vartheta }}_{n}^{\left(q_{\lambda }^{E}\right)}\right\},\;\mathrm{with}\\ C_{\lambda ,X_{0},n}^{(p_{\lambda }^{E},q_{\lambda }^{E}),S} & := & \exp\left\{x_{0}^{\left(q_{\lambda }^{E}\right)}\cdot \left[X_{0}-\frac{\alpha_{A}}{\beta_{A}}\cdot \frac{d^{(q_{\lambda }^{E}),S}}{1-d^{(q_{\lambda }^{E}),S}}\right]\cdot \left(1-{\left(d^{(q_{\lambda }^{E}),S}\right)}^{n}\right)+\frac{\alpha_{A}}{\beta_{A}}x_{0}^{\left(q_{\lambda }^{E}\right)}\cdot n\right\}, \end{array}$$

*where in the case λ∈]0,1[*

(98) $$\begin{array}{ccc} {\underline{\zeta }}_{n}^{\left(q_{\lambda }^{E}\right)} & := & \Gamma_{<}^{\left(q_{\lambda }^{E}\right)}\cdot \frac{{\left(d^{(q_{\lambda }^{E}),T}\right)}^{n-1}}{1-d^{(q_{\lambda }^{E}),T}}\cdot \left(1-{\left(d^{(q_{\lambda }^{E}),T}\right)}^{n}\right)\;>\;0\;,\; \end{array}$$

(99) $$\begin{array}{ccc} {\underline{\vartheta }}_{n}^{\left(q_{\lambda }^{E}\right)} & := & \Gamma_{<}^{\left(q_{\lambda }^{E}\right)}\cdot \frac{1-{\left(d^{(q_{\lambda }^{E}),T}\right)}^{n}}{{\left(1-d^{(q_{\lambda }^{E}),T}\right)}^{2}}\cdot \left[1-\frac{d^{(q_{\lambda }^{E}),T}\left(1+{\left(d^{(q_{\lambda }^{E}),T}\right)}^{n}\right)}{1+d^{(q_{\lambda }^{E}),T}}\right]\;>\;0\;, \end{array}$$

(100) $$\begin{array}{ccc} {\bar{\zeta }}_{n}^{\left(q_{\lambda }^{E}\right)} & := & \Gamma_{<}^{\left(q_{\lambda }^{E}\right)}\cdot \left[\frac{{\left(d^{(q_{\lambda }^{E}),S}\right)}^{n}-{\left(d^{(q_{\lambda }^{E}),T}\right)}^{n}}{d^{(q_{\lambda }^{E}),S}-d^{(q_{\lambda }^{E}),T}}-{\left(d^{(q_{\lambda }^{E}),S}\right)}^{n-1}\cdot \frac{1-{\left(d^{(q_{\lambda }^{E}),T}\right)}^{n}}{1-d^{(q_{\lambda }^{E}),T}}\right]\;>\;0\;, \end{array}$$

(101) $$\begin{array}{ccc} {\bar{\vartheta }}_{n}^{\left(q_{\lambda }^{E}\right)} & := & \Gamma_{<}^{\left(q_{\lambda }^{E}\right)}\cdot \frac{d^{(q_{\lambda }^{E}),T}}{1-d^{(q_{\lambda }^{E}),T}}\cdot \left[\frac{1-{\left(d^{(q_{\lambda }^{E}),S}d^{(q_{\lambda }^{E}),T}\right)}^{n}}{1-d^{(q_{\lambda }^{E}),S}d^{(q_{\lambda }^{E}),T}}-\frac{{\left(d^{(q_{\lambda }^{E}),S}\right)}^{n}-{\left(d^{(q_{\lambda }^{E}),T}\right)}^{n}}{d^{(q_{\lambda }^{E}),S}-d^{(q_{\lambda }^{E}),T}}\right]\;>\;0\;,\; \end{array}$$

*and where in the case $\lambda \in \;]\lambda_{-},\lambda_{+}[\;\backslash \left[0,1\right]$*

(102) $$\begin{array}{ccc} {\underline{\zeta }}_{n}^{\left(q_{\lambda }^{E}\right)} & := & \Gamma_{>}^{\left(q_{\lambda }^{E}\right)}\cdot \frac{{\left(d^{(q_{\lambda }^{E}),T}\right)}^{n}-{\left(d^{(q_{\lambda }^{E}),S}\right)}^{2n}}{d^{(q_{\lambda }^{E}),T}-{\left(d^{(q_{\lambda }^{E}),S}\right)}^{2}}\;>\;0\;, \end{array}$$

(103) $$\begin{array}{ccc} {\underline{\vartheta }}_{n}^{\left(q_{\lambda }^{E}\right)} & := & \frac{\Gamma_{>}^{\left(q_{\lambda }^{E}\right)}}{d^{(q_{\lambda }^{E}),T}\;-{\left(d^{(q_{\lambda }^{E}),S}\right)}^{2}}\left[\frac{d^{(q_{\lambda }^{E}),T}\left(1-{\left(d^{(q_{\lambda }^{E}),T}\right)}^{n}\right)}{1-d^{(q_{\lambda }^{E}),T}}-\frac{{\left(d^{(q_{\lambda }^{E}),S}\right)}^{2}\left(1-{\left(d^{(q_{\lambda }^{E}),S}\right)}^{2n}\right)}{1-{\left(d^{(q_{\lambda }^{E}),S}\right)}^{2}}\right]\\ & > & 0\;, \end{array}$$

(104) $$\begin{array}{ccc} {\bar{\zeta }}_{n}^{\left(q_{\lambda }^{E}\right)} & := & \Gamma_{>}^{\left(q_{\lambda }^{E}\right)}\cdot {\left(d^{(q_{\lambda }^{E}),S}\right)}^{n-1}\cdot \left[n\;-\;\frac{1-{\left(d^{(q_{\lambda }^{E}),T}\right)}^{n}}{1-d^{(q_{\lambda }^{E}),T}}\right]\;>\;0\;, \end{array}$$

(105) $$\begin{array}{ccc} {\bar{\vartheta }}_{n}^{\left(q_{\lambda }^{E}\right)} & := & \Gamma_{>}^{\left(q_{\lambda }^{E}\right)}\cdot [\frac{d^{(q_{\lambda }^{E}),S}-d^{(q_{\lambda }^{E}),T}}{{\left(1-d^{(q_{\lambda }^{E}),S}\right)}^{2}\left(1-d^{(q_{\lambda }^{E}),T}\right)}\cdot \left(1-{\left(d^{(q_{\lambda }^{E}),S}\right)}^{n}\right)\\ & & \;+\;\frac{d^{(q_{\lambda }^{E}),T}\left(1-{\left(d^{(q_{\lambda }^{E}),S}d^{(q_{\lambda }^{E}),T}\right)}^{n}\right)}{\left(1-d^{(q_{\lambda }^{E}),T}\right)\left(1-d^{(q_{\lambda }^{E}),S}d^{(q_{\lambda }^{E}),T}\right)}-\frac{{\left(d^{(q_{\lambda }^{E}),S}\right)}^{n}}{1-d^{(q_{\lambda }^{E}),S}}\cdot n]\;>\;0\;.\;\; \end{array}$$

*Notice that $\frac{\alpha_{A}}{\beta_{A}}$ can be equivalently be replaced by $\frac{\alpha_{H}}{\beta_{H}}$ in (96) and in (97).*

A proof of Theorem 5 is given in Appendix A.3.

### 6.3. Explicit Closed-Form Bounds for the Cases $\left(\beta_{A},\beta_{H},\alpha_{A},\alpha_{H},\lambda \right)\in \left(P_{\mathrm{SP}}\backslash P_{\mathrm{SP},1}\right)\times ]0,1[$

To derive (explicit) closed-form lower bounds of the (nonexplicit) recursive lower bounds $B_{\lambda ,X_{0},n}^{L}$ for the Hellinger integral $H_{\lambda }(P_{A,n}∥P_{H,n})$ respectively closed-form upper bounds of the recursive upper bounds $B_{\lambda ,X_{0},n}^{U}$ for all parameters cases $\left(\beta_{A},\beta_{H},\alpha_{A},\alpha_{H},\lambda \right)\in \left(P_{\mathrm{SP}}\backslash P_{\mathrm{SP},1}\right)\times \left(R\backslash \left\{0,1\right\}\right)$, we combine part (b) of Theorem 1, Lemma 2, Lemma 3 together with appropriate parameters $p_{\lambda }^{L}=p^{L}\left(\beta_{A},\beta_{H},\alpha_{A},\alpha_{H},\lambda \right),\;p_{\lambda }^{U}=p^{U}\left(\beta_{A},\beta_{H},\alpha_{A},\alpha_{H},\lambda \right)\geq 0$ and $q_{\lambda }^{L}=q^{L}\left(\beta_{A},\beta_{H},\alpha_{A},\alpha_{H},\lambda \right)$, $q_{\lambda }^{U}=q^{U}\left(\beta_{A},\beta_{H},\alpha_{A},\alpha_{H},\lambda \right)>0$ satisfying (35). Notice that the representations of the lower and upper closed-form sequence-bounds depend on whether $0<q_{\lambda }^{A}<\beta_{\lambda }$, $0<q_{\lambda }^{A}=\beta_{\lambda }$ or $\max\left\{0,\beta_{\lambda }\right\}<q_{\lambda }^{A}<\min\left\{1,e^{\beta_{\lambda }-1}\right\}$ (A∈{L,U}).

Let us start with closed-form *lower* bounds for the case λ∈]0,1[; recall that the choice $p_{\lambda }^{L}=\alpha_{A}^{\lambda }\alpha_{H}^{1-\lambda },\;q_{\lambda }^{L}=\beta_{A}^{\lambda }\beta_{H}^{1-\lambda }$ led to the optimal recursive lower bounds $B_{\lambda ,X_{0},n}^{L}$ of the Hellinger integral (cf. Theorem 1(b) and Section 3.5). Correspondingly, we can derive

**Theorem** **6.** *Let $p_{\lambda }^{L}=\alpha_{A}^{\lambda }\alpha_{H}^{1-\lambda }$ and $q_{\lambda }^{L}=\beta_{A}^{\lambda }\beta_{H}^{1-\lambda }$. Then, the following assertions hold true:*

*(a)*  *For all $\left(\beta_{A},\beta_{H},\alpha_{A},\alpha_{H},\lambda \right)\in \left(P_{\mathrm{SP},2}\cup P_{\mathrm{SP},3a}\cup P_{\mathrm{SP},3b}\cup P_{\mathrm{SP},3c}\right)\times ]0,1[$ (for which particularly $0<q_{\lambda }^{L}<\beta_{\lambda }$, $\beta_{A}\neq \beta_{H}$), all initial population sizes $X_{0}\in N$ and all observation horizons n∈N there holds*
  $$\begin{array}{ccc} C_{\lambda ,X_{0},n}^{(p_{\lambda }^{L},q_{\lambda }^{L}),T}\; & \leq & \;C_{\lambda ,X_{0},n}^{(p_{\lambda }^{L},q_{\lambda }^{L}),L}\;\leq \;B_{\lambda ,X_{0},n}^{L}\;<\;1\;, \end{array}$$
  (106) $$\begin{array}{ccc} \mathrm{where}\;C_{\lambda ,X_{0},n}^{(p_{\lambda }^{L},q_{\lambda }^{L}),L} & := & C_{\lambda ,X_{0},n}^{(p_{\lambda }^{L},q_{\lambda }^{L}),T}\cdot \exp\left\{{\underline{\zeta }}_{n}^{\left(q_{\lambda }^{L}\right)}\cdot X_{0}\;+\;\frac{p_{\lambda }^{L}}{q_{\lambda }^{L}}\cdot {\underline{\vartheta }}_{n}^{\left(q_{\lambda }^{L}\right)}\right\} \end{array}$$
  (107) $$\begin{array}{ccc} \mathrm{with}\;C_{\lambda ,X_{0},n}^{(p_{\lambda }^{L},q_{\lambda }^{L}),T} & := & \exp\{x_{0}^{\left(q_{\lambda }^{L}\right)}\cdot \left[X_{0}-\frac{p_{\lambda }^{L}}{q_{\lambda }^{L}}\cdot \frac{d^{(q_{\lambda }^{L}),T}}{1-d^{(q_{\lambda }^{L}),T}}\right]\cdot \left(1-{\left(d^{(q_{\lambda }^{L}),T}\right)}^{n}\right)\\ & & \;+\;\left(\frac{p_{\lambda }^{L}}{q_{\lambda }^{L}}\cdot \left(\beta_{\lambda }+x_{0}^{\left(q_{\lambda }^{L}\right)}\right)-\alpha_{\lambda }\right)\cdot n\}\;,\\ \mathrm{and}\;\mathrm{with}\;{\underline{\zeta }}_{n}^{\left(q_{\lambda }^{L}\right)} & := & \Gamma_{<}^{\left(q_{\lambda }^{L}\right)}\cdot \frac{{\left(d^{(q_{\lambda }^{L}),T}\right)}^{n-1}}{1-d^{(q_{\lambda }^{L}),T}}\cdot \left(1-{\left(d^{(q_{\lambda }^{L}),T}\right)}^{n}\right)\;>\;0\;, \end{array}$$
  (108) $$\begin{array}{ccc} {\underline{\vartheta }}_{n}^{\left(q_{\lambda }^{L}\right)} & := & \Gamma_{<}^{\left(q_{\lambda }^{L}\right)}\cdot \frac{1-{\left(d^{(q_{\lambda }^{L}),T}\right)}^{n}}{{\left(1-d^{(q_{\lambda }^{L}),T}\right)}^{2}}\cdot \left[1-\frac{d^{(q_{\lambda }^{L}),T}\left(1+{\left(d^{(q_{\lambda }^{L}),T}\right)}^{n}\right)}{1+d^{(q_{\lambda }^{L}),T}}\right]\;>\;0\;.\; \end{array}$$
*(b)*  *For all $\left(\beta_{A},\beta_{H},\alpha_{A},\alpha_{H},\lambda \right)\in \left(P_{\mathrm{SP},4a}\cup P_{\mathrm{SP},4b}\right)\times ]0,1[$ (for which particularly $0<q_{\lambda }^{L}=\beta_{\lambda }$, $\beta_{A}=\beta_{H}$), all initial population sizes $X_{0}\in N$ and all observation horizons n∈N there holds*
  $$C_{\lambda ,X_{0},n}^{(p_{\lambda }^{L},q_{\lambda }^{L}),L}\;:=\;C_{\lambda ,X_{0},n}^{(p_{\lambda }^{L},q_{\lambda }^{L}),T}\;:=\;B_{\lambda ,X_{0},n}^{L}\;=\;\exp\left\{\left(p_{\lambda }^{L}-\alpha_{\lambda }\right)\cdot n\right\}\;<\;1\;.$$
*(c)*  *For all $\left(\beta_{A},\beta_{H},\alpha_{A},\alpha_{H},\lambda \right)\in \left(P_{\mathrm{SP}}\backslash P_{\mathrm{SP},1}\right)\times ]0,1[$ and all initial population sizes $X_{0}\in N$ one gets*
  $$\begin{array}{ccc} \lim_{n\to \infty }\frac{1}{n}\log\left(C_{\lambda ,X_{0},n}^{(p_{\lambda }^{L},q_{\lambda }^{L}),T}\right) & = & \lim_{n\to \infty }\frac{1}{n}\log\left(C_{\lambda ,X_{0},n}^{(p_{\lambda }^{L},q_{\lambda }^{L}),L}\right)\;=\;\lim_{n\to \infty }\frac{1}{n}\log\left(B_{\lambda ,X_{0},n}^{L}\right)\\ & = & \frac{p_{\lambda }^{L}}{q_{\lambda }^{L}}\cdot \left(\beta_{\lambda }+x_{0}^{\left(q_{\lambda }^{L}\right)}\right)-\alpha_{\lambda }\;<\;0\;, \end{array}$$
  *where in the case $\beta_{A}=\beta_{H}$ there holds $q_{\lambda }^{L}=\beta_{\lambda }$ and $x_{0}^{\left(q_{\lambda }^{L}\right)}=0$.*

The proof will be provided in Appendix A.3.

In order to deduce closed-form *upper* bounds for the case λ∈]0,1[, we first recall from the Section 3.6, Section 3.7, Section 3.8, Section 3.9, Section 3.10, Section 3.11, Section 3.12 and Section 3.13, that we have to employ suitable parameters $p_{\lambda }^{U}=p^{U}\left(\beta_{A},\beta_{H},\alpha_{A},\alpha_{H},\lambda \right),\;q_{\lambda }^{U}=q^{U}\left(\beta_{A},\beta_{H},\alpha_{A},\alpha_{H},\lambda \right)$ satisfying (35). Notice that we automatically obtain $p_{\lambda }^{U}\geq p_{\lambda }^{L}=\alpha_{A}^{\lambda }\alpha_{H}^{1-\lambda }>0$. Correspondingly, we obtain

**Theorem** **7.** *For all $\left(\beta_{A},\beta_{H},\alpha_{A},\alpha_{H},\lambda \right)\in \left(P_{\mathrm{SP}}\backslash P_{\mathrm{SP},1}\right)\times ]0,1[$, all coefficients $p_{\lambda }^{U},\;q_{\lambda }^{U}$ which satisfy (35) for all $x\in N_{0}$ and additionally either $0<q_{\lambda }^{U}\leq \beta_{\lambda }$ or $\beta_{\lambda }<q_{\lambda }^{U}<\min\left\{1,e^{\beta_{\lambda }-1}\right\}$, all initial population sizes $X_{0}\in N$ and all observation horizons n∈N the following assertions hold true:*

(109) $$C_{\lambda ,X_{0},n}^{(p_{\lambda }^{U},q_{\lambda }^{U}),S}\;\geq \;C_{\lambda ,X_{0},n}^{(p_{\lambda }^{U},q_{\lambda }^{U}),U}\;\geq \;{\tilde{B}}_{\lambda ,X_{0},n}^{\left(p_{\lambda }^{U},q_{\lambda }^{U}\right)}\;\geq \;B_{\lambda ,X_{0},n}^{U}\;,\;\mathrm{where}$$

*(a)*  *in the case $0<q_{\lambda }^{U}<\beta_{\lambda }$ one has*
  (110) $$\begin{array}{c} C_{\lambda ,X_{0},n}^{(p_{\lambda }^{U},q_{\lambda }^{U}),U}:=C_{\lambda ,X_{0},n}^{(p_{\lambda }^{U},q_{\lambda }^{U}),S}\cdot \exp\left\{-\;{\bar{\zeta }}_{n}^{\left(q_{\lambda }^{U}\right)}\cdot X_{0}\;-\;\frac{p_{\lambda }^{U}}{q_{\lambda }^{U}}\cdot {\bar{\vartheta }}_{n}^{\left(q_{\lambda }^{U}\right)}\right\} \end{array}$$
  (111) $$\begin{array}{c} \mathrm{with}\;C_{\lambda ,X_{0},n}^{(p_{\lambda }^{U},q_{\lambda }^{U}),S}:=\exp\{x_{0}^{\left(q_{\lambda }^{U}\right)}\cdot \left[X_{0}-\frac{p_{\lambda }^{U}}{q_{\lambda }^{U}}\cdot \frac{d^{(q_{\lambda }^{U}),S}}{1-d^{(q_{\lambda }^{U}),S}}\right]\cdot \left(1-{\left(d^{(q_{\lambda }^{U}),S}\right)}^{n}\right)\\ \;+\;\left(\frac{p_{\lambda }^{U}}{q_{\lambda }^{U}}\cdot \left(\beta_{\lambda }+x_{0}^{\left(q_{\lambda }^{U}\right)}\right)-\alpha_{\lambda }\right)\cdot n\}\;,\;\\ {\bar{\zeta }}_{n}^{\left(q_{\lambda }^{U}\right)}:=\Gamma_{<}^{\left(q_{\lambda }^{U}\right)}\cdot \left[\frac{{\left(d^{(q_{\lambda }^{U}),S}\right)}^{n}-{\left(d^{(q_{\lambda }^{U}),T}\right)}^{n}}{d^{(q_{\lambda }^{U}),S}-d^{(q_{\lambda }^{U}),T}}-{\left(d^{(q_{\lambda }^{U}),S}\right)}^{n-1}\cdot \frac{1-{\left(d^{(q_{\lambda }^{U}),T}\right)}^{n}}{1-d^{(q_{\lambda }^{U}),T}}\right]\;>\;0\;, \end{array}$$
  (112) $$\begin{array}{c} {\bar{\vartheta }}_{n}^{\left(q_{\lambda }^{U}\right)}:=\Gamma_{<}^{\left(q_{\lambda }^{U}\right)}\cdot \frac{d^{(q_{\lambda }^{U}),T}}{1-d^{(q_{\lambda }^{U}),T}}\cdot \left[\frac{1-{\left(d^{(q_{\lambda }^{U}),S}d^{(q_{\lambda }^{U}),T}\right)}^{n}}{1-d^{(q_{\lambda }^{U}),S}d^{(q_{\lambda }^{U}),T}}-\frac{{\left(d^{(q_{\lambda }^{U}),S}\right)}^{n}-{\left(d^{(q_{\lambda }^{U}),T}\right)}^{n}}{d^{(q_{\lambda }^{U}),S}-d^{(q_{\lambda }^{U}),T}}\right]\;>\;0\;;\; \end{array}$$
  *furthermore, whenever $p_{\lambda }^{U},\;q_{\lambda }^{U}$ satisfy additionally (47) (such parameters exist particularly in the setups $P_{\mathrm{SP},2}\cup P_{\mathrm{SP},3a}\cup P_{\mathrm{SP},3b}$, cf. Section 3.7, Section 3.8 and Section 3.9), then*
  $$1\;>\;C_{\lambda ,X_{0},n}^{(p_{\lambda }^{U},q_{\lambda }^{U}),S}\;\mathrm{and}\;{\tilde{B}}_{\lambda ,X_{0},n}^{\left(p_{\lambda }^{U},q_{\lambda }^{U}\right)}\;=\;B_{\lambda ,X_{0},n}^{U}\;\forall \;n\in N\;;$$
*(b)*  *in the case $0<q_{\lambda }^{U}=\beta_{\lambda }$ one has*
  $$C_{\lambda ,X_{0},n}^{(p_{\lambda }^{U},q_{\lambda }^{U}),U}\;:=\;C_{\lambda ,X_{0},n}^{(p_{\lambda }^{U},q_{\lambda }^{U}),S}\;:=\;{\tilde{B}}_{\lambda ,X_{0},n}^{\left(p_{\lambda }^{U},q_{\lambda }^{U}\right)}\;=\;\exp\left\{\left(p_{\lambda }^{U}-\alpha_{\lambda }\right)\cdot n\right\}\;;$$
*(c)*  *in the case $\beta_{\lambda }<q_{\lambda }^{U}<\min\left\{1\;,\;e^{\beta_{\lambda }-1}\right\}$ the formulas (109) and (110) remain valid, but with*
  (113) $$\begin{array}{ccc} {\bar{\zeta }}_{n}^{\left(q_{\lambda }^{U}\right)} & := & \Gamma_{>}^{\left(q_{\lambda }^{U}\right)}\cdot {\left(d^{(q_{\lambda }^{U}),S}\right)}^{n-1}\cdot \left[n\;-\;\frac{1-{\left(d^{(q_{\lambda }^{U}),T}\right)}^{n}}{1-d^{(q_{\lambda }^{U}),T}}\right]\;>\;0\;,\; \end{array}$$
  (114) $$\begin{array}{ccc} {\bar{\vartheta }}_{n}^{\left(q_{\lambda }^{U}\right)} & := & \Gamma_{>}^{\left(q_{\lambda }^{U}\right)}\cdot [\frac{d^{(q_{\lambda }^{U}),S}-d^{(q_{\lambda }^{U}),T}}{{\left(1-d^{(q_{\lambda }^{U}),S}\right)}^{2}\left(1-d^{(q_{\lambda }^{U}),T}\right)}\cdot \left(1-{\left(d^{(q_{\lambda }^{U}),S}\right)}^{n}\right)\\ & & \;+\;\frac{d^{(q_{\lambda }^{U}),T}\left(1-{\left(d^{(q_{\lambda }^{U}),S}d^{(q_{\lambda }^{U}),T}\right)}^{n}\right)}{\left(1-d^{(q_{\lambda }^{U}),T}\right)\left(1-d^{(q_{\lambda }^{U}),S}d^{(q_{\lambda }^{U}),T}\right)}-\frac{{\left(d^{(q_{\lambda }^{U}),S}\right)}^{n}}{1-d^{(q_{\lambda }^{U}),S}}\cdot n]\;>\;0\;;\; \end{array}$$
*(d)*  *for all cases (a) to (c) one gets*
  $$\begin{array}{ccc} \lim_{n\to \infty }\frac{1}{n}\log\left(C_{\lambda ,X_{0},n}^{(p_{\lambda }^{U},q_{\lambda }^{U}),S}\right) & = & \lim_{n\to \infty }\frac{1}{n}\log\left(C_{\lambda ,X_{0},n}^{(p_{\lambda }^{U},q_{\lambda }^{U}),U}\right)\;=\;\lim_{n\to \infty }\frac{1}{n}\log\left({\tilde{B}}_{\lambda ,X_{0},n}^{\left(p_{\lambda }^{U},q_{\lambda }^{U}\right)}\right)\\ & = & \frac{p_{\lambda }^{U}}{q_{\lambda }^{U}}\cdot \left(\beta_{\lambda }+x_{0}^{\left(q_{\lambda }^{U}\right)}\right)-\alpha_{\lambda }\;, \end{array}$$
  *where in the case $q_{\lambda }^{U}=\beta_{\lambda }$ there holds $x_{0}^{\left(q_{\lambda }^{U}\right)}=0$.*

This Theorem 7 will be proved in Appendix A.3. Notice that for an inadequate choice of $p_{\lambda }^{U},\;q_{\lambda }^{U}$ it may hold that $\frac{p_{\lambda }^{U}}{q_{\lambda }^{U}}\left(\beta_{\lambda }+x_{0}^{\left(q_{\lambda }^{U}\right)}\right)-\alpha_{\lambda }>0$ in part (d) of Theorem 7.

### 6.4. Explicit Closed-Form Bounds for the Cases $\left(\beta_{A},\beta_{H},\alpha_{A},\alpha_{H},\lambda \right)\in \left(P_{\mathrm{SP}}\backslash P_{\mathrm{SP},1}\right)\times \left(R\backslash \left[0,1\right]\right)$

For λ∈R\[0,1], let us now construct closed-form *lower* bounds of the recursive lower bound components ${\tilde{B}}_{\lambda ,X_{0},n}^{\left(p_{\lambda }^{L},q_{\lambda }^{L}\right)}$, for suitable parameters $p_{\lambda }^{L}\geq 0$ and either $0<q_{\lambda }^{L}\leq \beta_{\lambda }$ or $\max\left\{0,\beta_{\lambda }\right\}<q_{\lambda }^{L}<\min\left\{1,e^{\beta_{\lambda }-1}\right\}$ satisfying (35).

**Theorem** **8.** *For all $\left(\beta_{A},\beta_{H},\alpha_{A},\alpha_{H},\lambda \right)\in \left(P_{\mathrm{SP}}\backslash P_{\mathrm{SP},1}\right)\times \left(R\backslash \left[0,1\right]\right)$, all coefficients $p_{\lambda }^{L}\geq 0,\;q_{\lambda }^{L}>0$ which satisfy (35) for all $x\in N_{0}$ and either $0<q_{\lambda }^{L}\leq \beta_{\lambda }$ or $\max\left\{0,\beta_{\lambda }\right\}<q_{\lambda }^{L}<\min\left\{1,e^{\beta_{\lambda }-1}\right\}$, all initial population sizes $X_{0}\in N$ and all observation horizons n∈N the following assertions hold true:*

(115) $$C_{\lambda ,X_{0},n}^{(p_{\lambda }^{L},q_{\lambda }^{L}),T}\leq \;C_{\lambda ,X_{0},n}^{(p_{\lambda }^{L},q_{\lambda }^{L}),L}\;\leq \;{\tilde{B}}_{\lambda ,X_{0},n}^{\left(p_{\lambda }^{L},q_{\lambda }^{L}\right)}\;\leq \;B_{\lambda ,X_{0},n}^{L}\;,\;\mathrm{where}$$

*(a)*  *in the case $0<q_{\lambda }^{L}<\beta_{\lambda }$ one has*
  (116) $$\begin{array}{c} C_{\lambda ,X_{0},n}^{(p_{\lambda }^{L},q_{\lambda }^{L}),L}:=C_{\lambda ,X_{0},n}^{(p_{\lambda }^{L},q_{\lambda }^{L}),T}\cdot \exp\left\{{\underline{\zeta }}_{n}^{\left(q_{\lambda }^{L}\right)}\cdot X_{0}\;+\;\frac{p_{\lambda }^{L}}{q_{\lambda }^{L}}\cdot {\underline{\vartheta }}_{n}^{\left(q_{\lambda }^{L}\right)}\right\}, \end{array}$$
  (117) $$\begin{array}{c} \mathrm{with}\;C_{\lambda ,X_{0},n}^{(p_{\lambda }^{L},q_{\lambda }^{L}),T}:=\exp\{x_{0}^{\left(q_{\lambda }^{L}\right)}\cdot \left[X_{0}-\frac{p_{\lambda }^{L}}{q_{\lambda }^{L}}\cdot \frac{d^{(q_{\lambda }^{L}),T}}{1-d^{(q_{\lambda }^{L}),T}}\right]\cdot \left(1-{\left(d^{(q_{\lambda }^{L}),T}\right)}^{n}\right)\\ \;+\;\left(\frac{p_{\lambda }^{L}}{q_{\lambda }^{L}}\cdot \left(\beta_{\lambda }+x_{0}^{\left(q_{\lambda }^{L}\right)}\right)-\alpha_{\lambda }\right)\cdot n\}\\ {\underline{\zeta }}_{n}^{\left(q_{\lambda }^{L}\right)}:=\Gamma_{<}^{\left(q_{\lambda }^{L}\right)}\cdot \frac{{\left(d^{(q_{\lambda }^{L}),T}\right)}^{n-1}}{1-d^{(q_{\lambda }^{L}),T}}\cdot \left(1-{\left(d^{(q_{\lambda }^{L}),T}\right)}^{n}\right)\;>\;0\;, \end{array}$$
  (118) $$\begin{array}{c} {\underline{\vartheta }}_{n}^{\left(q_{\lambda }^{L}\right)}:=\Gamma_{<}^{\left(q_{\lambda }^{L}\right)}\cdot \frac{1-{\left(d^{(q_{\lambda }^{L}),T}\right)}^{n}}{{\left(1-d^{(q_{\lambda }^{L}),T}\right)}^{2}}\cdot \left[1-\frac{d^{(q_{\lambda }^{L}),T}\left(1+{\left(d^{(q_{\lambda }^{L}),T}\right)}^{n}\right)}{1+d^{(q_{\lambda }^{L}),T}}\right]\;>\;0\;; \end{array}$$
  *furthermore, whenever $p_{\lambda }^{L},\;q_{\lambda }^{L}$ satisfy additionally (56) (such parameters exist particularly in the setups $P_{\mathrm{SP},2}\cup P_{\mathrm{SP},3a}\cup P_{\mathrm{SP},3b}$, cf. Section 3.17, Section 3.18 and Section 3.19), then*
  $$1\;<\;C_{\lambda ,X_{0},n}^{(p_{\lambda }^{L},q_{\lambda }^{L}),T}\;\mathrm{and}\;{\tilde{B}}_{\lambda ,X_{0},n}^{\left(p_{\lambda }^{L},q_{\lambda }^{L}\right)}\;=\;B_{\lambda ,X_{0},n}^{L}\;\forall \;n\in N\;;$$
*(b)*  *in the case $0<q_{\lambda }^{L}=\beta_{\lambda }$ one has*
  $$C_{\lambda ,X_{0},n}^{(p_{\lambda }^{L},q_{\lambda }^{L}),L}\;:=\;C_{\lambda ,X_{0},n}^{(p_{\lambda }^{L},q_{\lambda }^{L}),T}\;=\;{\tilde{B}}_{\lambda ,X_{0},n}^{\left(p_{\lambda }^{L},q_{\lambda }^{L}\right)}\;=\;\exp\left\{\left(p_{\lambda }^{L}-\alpha_{\lambda }\right)\cdot n\right\}\;;$$
*(c)*  *in the case $\max\left\{0\;,\;\beta_{\lambda }\right\}<q_{\lambda }^{L}<\min\left\{1\;,\;e^{\beta_{\lambda }-1}\right\}$ the formulas (115) and (116) remain valid, but with*
  (119) $$\;{\underline{\zeta }}_{n}^{\left(q_{\lambda }^{L}\right)}:=\Gamma_{>}^{\left(q_{\lambda }^{L}\right)}\cdot \frac{{\left(d^{(q_{\lambda }^{L}),T}\right)}^{n}-{\left(d^{(q_{\lambda }^{L}),S}\right)}^{2n}}{d^{(q_{\lambda }^{L}),T}-{\left(d^{(q_{\lambda }^{L}),S}\right)}^{2}}\;>\;0\;,\;$$
  (120) $$\;{\underline{\vartheta }}_{n}^{\left(q_{\lambda }^{L}\right)}:=\frac{\Gamma_{>}^{\left(q_{\lambda }^{L}\right)}}{d^{(q_{\lambda }^{L}),T}-{\left(d^{(q_{\lambda }^{L}),S}\right)}^{2}}\cdot \left[\frac{d^{(q_{\lambda }^{L}),T}\cdot \left(1-{\left(d^{(q_{\lambda }^{L}),T}\right)}^{n}\right)}{1-d^{(q_{\lambda }^{L}),T}}-\frac{{\left(d^{(q_{\lambda }^{L}),S}\right)}^{2}\cdot \left(1-{\left(d^{(q_{\lambda }^{L}),S}\right)}^{2n}\right)}{1-{\left(d^{(q_{\lambda }^{L}),S}\right)}^{2}}\right]>\;0\;;$$
*(d)*  *for all cases (a) to (c) one gets*
  $$\begin{array}{ccc} \lim_{n\to \infty }\frac{1}{n}\log\left(C_{\lambda ,X_{0},n}^{(p_{\lambda }^{L},q_{\lambda }^{L}),T}\right) & = & \lim_{n\to \infty }\frac{1}{n}\log\left(C_{\lambda ,X_{0},n}^{(p_{\lambda }^{L},q_{\lambda }^{L}),L}\right)\;=\;\lim_{n\to \infty }\frac{1}{n}\log\left({\tilde{B}}_{\lambda ,X_{0},n}^{\left(p_{\lambda }^{L},q_{\lambda }^{L}\right)}\right)\\ & = & \frac{p_{\lambda }^{L}}{q_{\lambda }^{L}}\cdot \left(\beta_{\lambda }+x_{0}^{\left(q_{\lambda }^{L}\right)}\right)-\alpha_{\lambda }\;, \end{array}$$
  *where in the case $q_{\lambda }^{L}=\beta_{\lambda }$ there holds $x_{0}^{\left(q_{\lambda }^{L}\right)}=0$.*

For the proof of Theorem 8, see Appendix A.3. Notice that for an inadequate choice of $p_{\lambda }^{L},\;q_{\lambda }^{L}$ it may hold that $\frac{p_{\lambda }^{L}}{q_{\lambda }^{L}}\left(\beta_{\lambda }+x_{0}^{\left(q_{\lambda }^{U}\right)}\right)-\alpha_{\lambda }<0$ in the last assertion of Theorem 8.

To derive closed-form *upper* bounds of the recursive upper bounds $B_{\lambda ,X_{0},n}^{U}$ of the Hellinger integral in the case λ∈R\[0,1], let us first recall from Section 3.24 that we have to use the parameters $p_{\lambda }^{U}=\alpha_{A}^{\lambda }\alpha_{H}^{1-\lambda }>0$ and $q_{\lambda }^{U}=\beta_{A}^{\lambda }\beta_{H}^{1-\lambda }>0$. Furthermore, in the case $\beta_{A}\neq \beta_{H}$ we obtain from Lemma 1 (setting $q_{\lambda }=q_{\lambda }^{U}$) the assertion that $\max\left\{0,\beta_{\lambda }\right\}<q_{\lambda }^{U}<\min\left\{1,e^{\beta_{\lambda }-1}\right\}$ iff $\lambda \in ]\lambda_{-},\lambda_{+}[\;\backslash \;\left[0,1\right]$(implying that the sequence ${\left(a_{n}^{\left(q_{\lambda }^{U}\right)}\right)}_{n\in N}$ converges). In the case $\beta_{A}=\beta_{H}$ on gets $q_{\lambda }^{U}=\beta_{A}^{\lambda }\beta_{H}^{1-\lambda }=\beta_{A}=\beta_{H}=\beta_{\lambda }$ and therefore (cf. (P2)) $a_{n}^{\left(q_{\lambda }^{U}\right)}=0$ for all n∈N and for all λ∈R\[0,1]. Correspondingly, we deduce

**Theorem** **9.** *Let $p_{\lambda }^{U}=\alpha_{A}^{\lambda }\alpha_{H}^{1-\lambda }$ and $q_{\lambda }^{U}=\beta_{A}^{\lambda }\beta_{H}^{1-\lambda }$. Then, the following assertions hold true:*

*(a)*  *For all $\left(\beta_{A},\beta_{H},\alpha_{A},\alpha_{H},\lambda \right)\in \left(P_{\mathrm{SP},2}\cup P_{\mathrm{SP},3a}\cup P_{\mathrm{SP},3b}\cup P_{\mathrm{SP},3c}\right)\times \left(\;\right]\lambda_{-},\lambda_{+}\left[\;\backslash \left[0,1\right]\;\right)$ (in particular for $\beta_{A}\neq \beta_{H}$), all initial population sizes $X_{0}\in N$ and all observation horizons n∈N there holds*
  $$\infty \;>\;C_{\lambda ,X_{0},n}^{(p_{\lambda }^{U},q_{\lambda }^{U}),S}\;\geq \;C_{\lambda ,X_{0},n}^{(p_{\lambda }^{U},q_{\lambda }^{U}),U}\;\geq \;B_{\lambda ,X_{0},n}^{U}\;>\;1\;,$$
  (121) $$\begin{array}{cc} \mathrm{where} & C_{\lambda ,X_{0},n}^{(p_{\lambda }^{U},q_{\lambda }^{U}),U}:=C_{\lambda ,X_{0},n}^{(p_{\lambda }^{U},q_{\lambda }^{U}),S}\cdot \exp\left\{-\;{\bar{\zeta }}_{n}^{\left(q_{\lambda }^{U}\right)}\cdot X_{0}\;-\;\frac{p_{\lambda }^{U}}{q_{\lambda }^{U}}\cdot {\bar{\vartheta }}_{n}^{\left(q_{\lambda }^{U}\right)}\right\} \end{array}$$
  (122) $$\begin{array}{cc} \mathrm{with} & C_{\lambda ,X_{0},n}^{(p_{\lambda }^{U},q_{\lambda }^{U}),S}:=\exp\{x_{0}^{\left(q_{\lambda }^{U}\right)}\cdot \left[X_{0}-\frac{p_{\lambda }^{U}}{q_{\lambda }^{U}}\cdot \frac{d^{(q_{\lambda }^{U}),T}}{1-d^{(q_{\lambda }^{U}),T}}\right]\cdot \left(1-{\left(d^{(q_{\lambda }^{U}),T}\right)}^{n}\right)\\ & \;+\;\left(\frac{p_{\lambda }^{U}}{q_{\lambda }^{U}}\cdot \left(\beta_{\lambda }+x_{0}^{\left(q_{\lambda }^{U}\right)}\right)-\alpha_{\lambda }\right)\cdot n\;\}\;,\\ & {\bar{\zeta }}_{n}^{\left(q_{\lambda }^{U}\right)}:=\Gamma_{>}^{\left(q_{\lambda }^{U}\right)}\cdot {\left(d^{(q_{\lambda }^{U}),S}\right)}^{n-1}\cdot \left[n\;-\;\frac{1-{\left(d^{(q_{\lambda }^{U}),T}\right)}^{n}}{1-d^{(q_{\lambda }^{U}),T}}\right]\;>\;0\;, \end{array}$$
  (123) $$\begin{array}{c} {\bar{\vartheta }}_{n}^{\left(q_{\lambda }^{U}\right)}:=\Gamma_{>}^{\left(q_{\lambda }^{U}\right)}\cdot [\frac{d^{(q_{\lambda }^{U}),S}-d^{(q_{\lambda }^{U}),T}}{{\left(1-d^{(q_{\lambda }^{U}),S}\right)}^{2}\left(1-d^{(q_{\lambda }^{U}),T}\right)}\cdot \left(1-{\left(d^{(q_{\lambda }^{U}),S}\right)}^{n}\right)\\ \;+\;\frac{d^{(q_{\lambda }^{U}),T}\left(1-{\left(d^{(q_{\lambda }^{U}),S}d^{(q_{\lambda }^{U}),T}\right)}^{n}\right)}{\left(1-d^{(q_{\lambda }^{U}),T}\right)\left(1-d^{(q_{\lambda }^{U}),S}d^{(q_{\lambda }^{U}),T}\right)}-\frac{{\left(d^{(q_{\lambda }^{U}),S}\right)}^{n}}{1-d^{(q_{\lambda }^{U}),S}}\cdot n]\;>\;0\;. \end{array}$$
*(b)*  *For all $\left(\beta_{A},\beta_{H},\alpha_{A},\alpha_{H},\lambda \right)\in \left(P_{\mathrm{SP},4a}\cup P_{\mathrm{SP},4b}\right)\times \left(\;R\backslash \left[0,1\right]\;\right)$ (for which particularly $0<q_{\lambda }^{U}=\beta_{\lambda }$, $\beta_{A}=\beta_{H}$), all initial population sizes $X_{0}\in N$ and all observation horizons n∈N there holds*
  $$C_{\lambda ,X_{0},n}^{(p_{\lambda }^{U},q_{\lambda }^{U}),U}\;:=\;C_{\lambda ,X_{0},n}^{(p_{\lambda }^{U},q_{\lambda }^{U}),S}\;:=\;B_{\lambda ,X_{0},n}^{U}\;=\;\exp\left\{\left(p_{\lambda }^{U}-\alpha_{\lambda }\right)\cdot n\right\}\;>\;1\;.$$
*(c)*  *For all $\left(\beta_{A},\beta_{H},\alpha_{A},\alpha_{H},\lambda \right)\in \left(P_{\mathrm{SP}}\backslash P_{\mathrm{SP},1}\right)\times \left(\;\right]\lambda_{-},\lambda_{+}\left[\;\backslash \left[0,1\right]\;\right)$ and all initial population sizes $X_{0}\in N$ one gets*
  $$\begin{array}{ccc} \lim_{n\to \infty }\frac{1}{n}\log\left(C_{\lambda ,X_{0},n}^{(p_{\lambda }^{U},q_{\lambda }^{U}),S}\right) & = & \lim_{n\to \infty }\frac{1}{n}\log\left(C_{\lambda ,X_{0},n}^{(p_{\lambda }^{U},q_{\lambda }^{U}),U}\right)\;=\;\lim_{n\to \infty }\frac{1}{n}\log\left(B_{\lambda ,X_{0},n}^{U}\right)\\ & = & \frac{p_{\lambda }^{U}}{q_{\lambda }^{U}}\cdot \left(\beta_{\lambda }+x_{0}^{\left(q_{\lambda }^{U}\right)}\right)-\alpha_{\lambda }\;>\;0\;, \end{array}$$
  *where in the case $\beta_{A}=\beta_{H}$ there holds $q_{\lambda }^{U}=\beta_{\lambda }$ and $x_{0}^{\left(q_{\lambda }^{U}\right)}=0$.*

A proof of Theorem 9 is provided in Appendix A.3.

**Remark** **7.** *Substituting $a_{n}^{\left(q\right)}$ by $a_{n}^{(q),T}$ resp. $a_{n}^{(q),S}$ (cf. (78) resp. (79)) in ${\tilde{B}}_{\lambda ,X_{0},n}^{\left(p,q\right)}$ from (42) leads to the “rudimentary” closed-form bounds $C_{\lambda ,X_{0},n}^{(p,q),T}$ resp. $C_{\lambda ,X_{0},n}^{(p,q),S}$, whereas substituting $a_{n}^{\left(q\right)}$ by ${\underline{a}}_{n}^{\left(q\right)}$ resp. ${\bar{a}}_{n}^{\left(q\right)}$ (cf. (92) resp. (94)) in ${\tilde{B}}_{\lambda ,X_{0},n}^{\left(p,q\right)}$ from (42) leads to the “improved” closed-form bounds $C_{\lambda ,X_{0},n}^{(p,q),L}$ resp. $C_{\lambda ,X_{0},n}^{(p,q),U}$ in all the Theorems 5–9.*

### 6.5. Totally Explicit Closed-Form Bounds

The above-mentioned results give closed-form lower bounds $C_{\lambda ,X_{0},n}^{(p,q),L}$, $C_{\lambda ,X_{0},n}^{(p,q),T}$ resp. closed-form upper bounds $C_{\lambda ,X_{0},n}^{(p,q),U}$, $C_{\lambda ,X_{0},n}^{(p,q),S}$ of the Hellinger integrals $H_{\lambda }(P_{A,n}∥P_{H,n})$ for case-dependent choices of p,q. However, these bounds still involve the fixed point $x_{0}^{\left(q\right)}$ which in general has to be calculated implicitly. In order to get “totally” explicit but “slightly” less tight closed-form bounds of $H_{\lambda }(P_{A,n}∥P_{H,n})$, one can proceed as follows:

1. in all the closed-form lower bound formulas of the Theorems 5, 6 and 8–including the definitions (76), (77) and (91)–replace the implicit $x_{0}^{\left(q\right)}$ by a close explicitly known point ${\underline{x}}_{0}^{\left(q\right)}<x_{0}^{\left(q\right)}$;
2. in all closed-form upper bound formulas of the Theorems 5, 7 and 9–including (76), (77) and (91)–replace $x_{0}^{\left(q\right)}$ by a close explicitly known point ${\bar{x}}_{0}^{\left(q\right)}>x_{0}^{\left(q\right)}$.

For instance, one can use the following choices which will be also employed as an auxiliary tool for the diffusion-limit-concerning proof of Lemma A6 in Appendix A.4:

(124) $$\begin{array}{ccc} {\underline{x}}_{0}^{\left(q\right)} & := & \left\{\begin{array}{cc} q^{-1}\cdot e^{-{\underline{\underline{x}}}_{0}^{\left(q\right)}}\cdot \left[\left(1-q\right)-\sqrt{{\left(1-q\right)}^{2}-2\cdot q\cdot e^{{\underline{\underline{x}}}_{0}^{\left(q\right)}}\cdot \left(q-\beta_{\lambda }\right)}\right], & \mathrm{if}\;q\in ]0,\beta_{\lambda }[\;,\\ q^{-1}\cdot \left[\left(1-q\right)-\sqrt{{\left(1-q\right)}^{2}-2\cdot q\cdot \left(q-\beta_{\lambda }\right)}\right], & \;\mathrm{if}\;\max\left\{0,\beta_{\lambda }\right\}<q<\min\left\{1,e^{\beta_{\lambda }-1}\right\}, \end{array}\right. \end{array}$$

(125) $$\begin{array}{c} \;\mathrm{where}\;{\underline{\underline{x}}}_{0}^{\left(q\right)}\;:=\;\left\{\begin{array}{cc} \max\left\{-\beta_{\lambda }\;,\;\frac{q-\beta_{\lambda }}{1-q}\right\}, & \mathrm{if}\;q\in ]0,1[\;,\\ -\beta_{\lambda }, & \mathrm{if}\;q\;\geq \;1, \end{array}\right. \end{array}$$

(126) $$\begin{array}{ccc} {\bar{x}}_{0}^{\left(q\right)} & := & \left\{\begin{array}{cc} q^{-1}\cdot \left[\left(1-q\right)-\sqrt{{\left(1-q\right)}^{2}-2\cdot q\cdot \left(q-\beta_{\lambda }\right)}\right], & \;\mathrm{if}\;q\in ]0,\beta_{\lambda }[\;,\\ \left(1-q\right)-\sqrt{{\left(1-q\right)}^{2}-2\cdot \left(q-\beta_{\lambda }\right)}, & \;\mathrm{if}\;\max\left\{0,\beta_{\lambda }\right\}<q<\min\left\{1,e^{\beta_{\lambda }-1}\right\}\\ & \;\mathrm{and}\;{\left(1-q\right)}^{2}-2\cdot q\cdot \left(q-\beta_{\lambda }\right)\geq 0,\\ {\bar{\bar{x}}}_{0}^{\left(q\right)}\;:=\;-\log\left(q\right) & \;\mathrm{if}\;\max\left\{0,\beta_{\lambda }\right\}<q<\min\left\{1,e^{\beta_{\lambda }-1}\right\}\\ & \;\mathrm{and}\;{\left(1-q\right)}^{2}-2\cdot q\cdot \left(q-\beta_{\lambda }\right)<0. \end{array}\right. \end{array}$$

Behind this choice “lies” the idea that–in contrast to the solution $x_{0}^{\left(q\right)}$ of $\xi_{\lambda }^{\left(q\right)}\left(x\right):=qe^{x}-\beta_{\lambda }=x$–the point ${\underline{x}}_{0}^{\left(q\right)}$ is a solution of (the obviously explicitly solvable) ${\underline{Q}}_{\lambda }^{\left(q\right)}\left(x\right):={\underline{a}}_{\lambda }^{\left(q\right)}x^{2}+{\underline{b}}_{\lambda }^{\left(q\right)}x+{\underline{c}}_{\lambda }^{\left(q\right)}=x$ in both cases $0<q<\beta_{\lambda }$ and $\max\left\{0,\beta_{\lambda }\right\}<q<\min\left\{1,e^{\beta_{\lambda }-1}\right\}$, whereas the point ${\bar{x}}_{0}^{\left(q\right)}$ is a solution of ${\bar{Q}}_{\lambda }^{\left(q\right)}\left(x\right):={\bar{a}}_{\lambda }^{\left(q\right)}x^{2}+{\bar{b}}_{\lambda }^{\left(q\right)}x+{\bar{c}}_{\lambda }^{\left(q\right)}=x$ in the case $0<q<\beta_{\lambda }$ and in the case $\max\left\{0,\beta_{\lambda }\right\}<q<\min\left\{1,e^{\beta_{\lambda }-1}\right\}$ together with ${\left(1-q\right)}^{2}-2\cdot q\cdot \left(q-\beta_{\lambda }\right)\geq 0$. Thereby, ${\underline{Q}}_{\lambda }^{\left(q\right)}\left(\cdot \right)$ and ${\bar{Q}}_{\lambda }^{\left(q\right)}\left(\cdot \right)$ are the lower resp. upper quadratic approximates of $\xi_{\lambda }^{\left(q\right)}\left(\cdot \right)$ satisfying the following constraints:

- for $q\in ]0,\beta_{\lambda }[$ (mostly but not only for λ∈]0,1[) (lower bound):
  $${\underline{Q}}_{\lambda }^{\left(q\right)}\left(0\right)=\xi_{\lambda }^{\left(q\right)}\left(0\right)=q-\beta_{\lambda },\;{\underline{Q}}_{\lambda }^{(q)\;\prime }\left(0\right)=\xi_{\lambda }^{(q)\;\prime }\left(0\right)=q,\;{\underline{Q}}_{\lambda }^{(q)\;\prime \prime }\left(x\right)=\xi_{\lambda }^{(q)\;\prime \prime }\left(y\right)=qe^{y},\;x\in R,$$
  for some explicitly known approximate $y<x_{0}^{\left(q\right)}$(leading to the (tighter) explicit lower approximate ${\underline{x}}_{0}^{\left(q\right)}\in ]y,x_{0}^{\left(q\right)}[$); here, we choose
  $$y\;:=\;{\underline{\underline{x}}}_{0}^{\left(q\right)}\;:=\;\left\{\begin{array}{cc} \max\left\{-\beta_{\lambda }\;,\;\frac{q-\beta_{\lambda }}{1-q}\right\}, & \mathrm{if}\;q\;<\;1,\\ -\beta_{\lambda }, & \mathrm{if}\;q\;\geq \;1; \end{array}\right.$$
- for $q\in ]0,\beta_{\lambda }[$ (mostly but not only for λ∈]0,1[) (upper bound):
  $${\bar{Q}}_{\lambda }^{\left(q\right)}\left(0\right)=\xi_{\lambda }^{\left(q\right)}\left(0\right)=q-\beta_{\lambda },\;{\bar{Q}}_{\lambda }^{(q)\;\prime }\left(0\right)=\xi_{\lambda }^{(q)\;\prime }\left(0\right)=q,\;{\bar{Q}}_{\lambda }^{(q)\;\prime \prime }\left(x\right)=\xi_{\lambda }^{(q)\;\prime \prime }\left(0\right)=q,\;x\in R;$$
- for $\max\left\{0,\beta_{\lambda }\right\}<q<\min\left\{1,e^{\beta_{\lambda }-1}\right\}$ (mostly but not only for λ∈R\[0,1]) (lower bound):
  $${\underline{Q}}_{\lambda }^{\left(q\right)}\left(0\right)=\xi_{\lambda }^{\left(q\right)}\left(0\right)=q-\beta_{\lambda },\;{\underline{Q}}_{\lambda }^{(q)\;\prime }\left(0\right)=\xi_{\lambda }^{(q)\;\prime }\left(0\right)=q,\;{\underline{Q}}_{\lambda }^{(q)\;\prime \prime }\left(x\right)=\xi_{\lambda }^{(q)\;\prime \prime }\left(0\right)=q,\;x\in R;$$
- for $\max\left\{0,\beta_{\lambda }\right\}<q<\min\left\{1,e^{\beta_{\lambda }-1}\right\}$ in combination with ${\left(1-q\right)}^{2}-2\cdot q\cdot \left(q-\beta_{\lambda }\right)\geq 0$ (mostly but not only for λ∈R\[0,1]) (upper bound):
  $${\bar{Q}}_{\lambda }^{\left(q\right)}\left(0\right)=\xi_{\lambda }^{\left(q\right)}\left(0\right)=q-\beta_{\lambda },\;{\bar{Q}}_{\lambda }^{(q)\;\prime }\left(0\right)=\xi_{\lambda }^{(q)\;\prime }\left(0\right)=q,\;{\bar{Q}}_{\lambda }^{(q)\;\prime \prime }\left(x\right)=\xi_{\lambda }^{(q)\;\prime \prime }\left(-\log\left(q\right)\right)=1,\;x\in R.$$

If $\max\left\{0,\beta_{\lambda }\right\}<q<\min\left\{1,e^{\beta_{\lambda }-1}\right\}$ and ${\left(1-q\right)}^{2}-2\cdot q\cdot \left(q-\beta_{\lambda }\right)<0$, then a real-valued solution ${\bar{Q}}_{\lambda }^{\left(q\right)}\left(x\right)=x$ does not exist and we set ${\bar{x}}_{0}^{\left(q\right)}:={\bar{\bar{x}}}_{0}^{\left(q\right)}:=-\log\left(q\right)$, with $\xi_{\lambda }^{(q)\;\prime }\left({\bar{\bar{x}}}_{0}^{\left(q\right)}\right)=1$. The above considerations lead to corresponding unique choices of constants ${\underline{a}}_{\lambda }^{\left(q\right)},\;{\underline{b}}_{\lambda }^{\left(q\right)},\;{\underline{c}}_{\lambda }^{\left(q\right)},\;{\bar{a}}_{\lambda }^{\left(q\right)},\;{\bar{b}}_{\lambda }^{\left(q\right)},\;{\bar{c}}_{\lambda }^{\left(q\right)}$ culminating in

(127) $$\begin{array}{ccc} {\underline{Q}}_{\lambda }^{\left(q\right)}\left(x\right) & := & \left\{\begin{array}{cc} \frac{q}{2}\cdot e^{{\underline{\underline{x}}}_{0}^{\left(q\right)}}\cdot x^{2}\;+\;q\cdot x\;+\;q-\beta_{\lambda }, & \;\mathrm{if}\;0<q<\beta_{\lambda }\;,\\ \frac{q}{2}\cdot x^{2}\;+\;q\cdot x\;+\;q-\beta_{\lambda }, & \;\mathrm{if}\;\max\left\{0,\beta_{\lambda }\right\}<q<\min\left\{1,e^{\beta_{\lambda }-1}\right\}\;, \end{array}\right. \end{array}$$

(128) $$\begin{array}{ccc} {\bar{Q}}_{\lambda }^{\left(q\right)}\left(x\right) & := & \left\{\begin{array}{cc} \frac{q}{2}\cdot x^{2}\;+\;q\cdot x\;+\;q-\beta_{\lambda }, & \;\mathrm{if}\;0<q<\beta_{\lambda }\;,\\ \frac{1}{2}\cdot x^{2}\;+\;q\cdot x\;+\;q-\beta_{\lambda }, & \;\mathrm{if}\;\max\left\{0,\beta_{\lambda }\right\}<q<\min\left\{1,e^{\beta_{\lambda }-1}\right\}\;. \end{array}\right. \end{array}$$

### 6.6. Closed-Form Bounds for Power Divergences of Non-Kullback-Leibler-Information-Divergence Type

Analogously to Section 4 (see especially Section 4.1), for orders λ∈R\{0,1} all the results of the previous Section 6.1, Section 6.2, Section 6.3, Section 6.4 and Section 6.5 carry correspondingly over from closed-form bounds of the Hellinger integrals $H_{\lambda }\left(\cdot ∥\cdot \right)$ to closed-form bounds of the total variation distance V(·||·), by virtue of the relation (cf. (12))

$$2\;\left(1-H_{\frac{1}{2}}(P_{A,n}∥P_{H,n})\right)\;\leq \;V(P_{A,n}∥P_{H,n})\;\leq \;2\;\sqrt{1-{\left(H_{\frac{1}{2}}(P_{A,n}∥P_{H,n})\right)}^{2}}\;,$$

to closed-form bounds of the Renyi divergences $R_{\lambda }\left(\cdot ∥\cdot \right)$, by virtue of the relation (cf. (7))

$$0\leq \;R_{\lambda }\left(P_{A,n}∥P_{H,n}\right)\;=\;\frac{1}{\lambda (\lambda -1)}\;\log H_{\lambda }\left(P_{A,n}∥P_{H,n}\right)\;,\;\mathrm{with}\log\;0:=-\infty ,$$

as well as to closed-form bounds of the power divergences $I_{\lambda }\left(\cdot ∥\cdot \right)$, by virtue of the relation (cf. (2))

$$I_{\lambda }\left(P_{A,n}∥P_{H,n}\right)\;=\;\frac{1-H_{\lambda }(P_{A,n}∥P_{H,n})}{\lambda \cdot (1-\lambda )}\;,\;n\in N\;.$$

For the sake of brevity, the–merely repetitive–exact details are omitted.

### 6.7. Applications to Decision Making

The above-mentioned investigations of the Section 6.1 to Section 6.6 can be applied to the context of Section 2.5 on *dichotomous* decision making on the space of all possible path scenarios (path space) of Poissonian Galton-Watson processes without (with) immigration GW(I) (e.g., in combination with our running-example epidemiological context of Section 2.3). More detailed, for the minimal mean decision loss (Bayes risk) $R_{n}$ defined by (18) we can derive explicit closed-form upper (respectively lower) bounds by using (19) respectively (20) together with the results of the Section 6.1, Section 6.2, Section 6.3, Section 6.4 and Section 6.5 concerning Hellinger integrals of order λ∈]0,1[; we can proceed analogously in the Neyman-Pearson context in order to deduce closed-form bounds of type II error probabilities, by means of (23) and (24). Moreover, in an analogous way we can employ the investigations of Section 6.6 on power divergences in order to obtain closed-form bounds of (i) the corresponding (cf. (21)) *weighted-average* decision risk reduction (weighted-average statistical information measure) about the degree of evidence deg concerning the parameter θ that can be attained by observing the GW(I)-path $X_{n}$ until stage *n*, as well as (ii) the corresponding (cf. (22)) *limit* decision risk reduction (limit statistical information measure). For the sake of brevity, the–merely repetitive–exact details are omitted.

## 7. Hellinger Integrals and Power Divergences of Galton-Watson Type Diffusion Approximations

### 7.1. Branching-Type Diffusion Approximations

One can show that a properly rescaled Galton-Watson process without (respectively with) immigration GW(I) converges weakly to a diffusion process $\tilde{X}:=\left\{{\tilde{X}}_{s}\;,s\in [0,\infty [\right\}$ which is the unique, strong, nonnegative – and in case of $\frac{\eta }{\sigma^{2}}\geq \frac{1}{2}$ strictly positive– solution of the stochastic differential equation (SDE) of the form

(129) $$d{\tilde{X}}_{s}\;=\;\left(\eta \;-\;\kappa \;{\tilde{X}}_{s}\right)\;ds\;+\;\sigma \sqrt{{\tilde{X}}_{s}}\;dW_{s},\;\;s\in [0,\infty \left[,\;{\tilde{X}}_{0}\in \right]0,\infty [\mathrm{given},$$

where η∈[0,∞[, κ∈[0,∞[, σ∈]0,∞[ are constants and $\left\{W_{s}\;,\;s\in [0,\infty [\right\}$ denotes a standard Brownian motion with respect to the underlying probability measure *P*; see e.g., Feller [130], Jirina [131], Lamperti [132,133], Lindvall [134,135], Grimvall [136], Jagers [56], Borovkov [137], Ethier & Kurtz [138], Durrett [139] for the non-immigration case corresponding to η=0, κ≥0, Kawazu & Watanabe [140], Wei & Winnicki [141], Winnicki [64] for the immigration case corresponding to η≠0, κ=0, as well as Sriram [142] for the general case η∈[0,∞[, κ∈R. Feller-type branching processes of the form (129), which are special cases of continuous state branching processes with immigration (see e.g., Kawazu & Watanabe [140], Li [143], as well as Dawson & Li [144] for imbeddings to affine processes) play for instance an important role in the modelling of the term structure of interest rates, cf. the seminal Cox-Ingersoll-Ross CIR model [145] and the vast follow-up literature thereof. Furthermore, (129) is also prominently used as (a special case of) Cox & Ross’s [146] constant elasticity of variance CEV asset price process, as (part of) Heston’s [147] stochastic asset-volatility framework, as a model of neuron activity (see e.g., Lansky & Lanska [148], Giorno et al. [149], Lanska et al. [150], Lansky et al [151], Ditlevsen & Lansky [152], Höpfner [153], Lansky & Ditlevsen [154]), as a time-dynamic description of the nitrous oxide emission rate from the soil surface (see e.g., Pedersen [155]), as well as a model for the individual hazard rate in a survival analysis context (see e.g., Aalen & Gjessing [156]).

Along these lines of branching-type diffusion limits, it makes sense to consider the solutions of two SDEs (129) with different fixed parameter sets $\left(\eta ,\kappa_{A},\sigma \right)$ and $\left(\eta ,\kappa_{H},\sigma \right)$, determine for each of them a corresponding approximating GW(I), investigate the Hellinger integral between the laws of these two GW(I), and finally calculate the limit of the Hellinger integral (bounds) as the GW(I) approach their SDE solutions. Notice that for technicality reasons (which will be explained below), the constants η and σ ought to be independent of A, H in our current context.

In order to make the above-mentioned limit procedure rigorous, it is reasonable to work with appropriate approximations such that in each convergence step *m* one faces the setup $P_{\mathrm{NI}}\cup P_{\mathrm{SP},1}$ (i.e., the non-immigration or the equal-fraction case), where the corresponding Hellinger integral can be calculated exactly in a recursive way, as stated in Theorem 1. Let us explain the details in the following.

Consider a sequence of GW(I) ${\left(X^{\left(m\right)}\right)}_{m\in N}$ with probability laws $P_{•}^{\left(m\right)}$ on a measurable space (Ω,F), where as above the subscript • stands for either the hypothesis H or the alternative A. Analogously to (1), we use for each fixed step m∈N the representation $X^{\left(m\right)}:=\left\{X_{\ell }^{\left(m\right)},\;\ell \in N\right\}$ with

(130) $$X_{\ell }^{\left(m\right)}\;:=\;\sum_{j=1}^{X_{\ell -1}^{\left(m\right)}}Y_{\ell -1,j}^{\left(m\right)}+{\tilde{Y}}_{\ell }^{\left(m\right)},\;\ell \in N,\;X_{0}^{\left(m\right)}\in N\mathrm{given},$$

where under the law $P_{•}^{\left(m\right)}$

- the collection $Y^{\left(m\right)}:=\left\{Y_{i,j}^{\left(m\right)},\;i\in N_{0},\;j\in N\right\}$ consists of i.i.d. random variables which are Poisson distributed with parameter $\beta_{•}^{\left(m\right)}>0$,
- the collection ${\tilde{Y}}^{\left(m\right)}:=\left\{{\tilde{Y}}_{i}^{\left(m\right)},\;i\in N\right\}$ consists of i.i.d. random variables which are Poisson distributed with parameter $\alpha_{•}^{\left(m\right)}\geq 0$,
- $Y^{\left(m\right)}$ and ${\tilde{Y}}^{\left(m\right)}$ are independent.

From arbitrary drift-parameters η∈[0,∞[, $\kappa_{•}\in [0,\infty [$, and diffusion-term-parameter σ>0, we construct the offspring-distribution-parameter and the immigration-distribution parameter of the sequence ${\left(X_{\ell }^{\left(m\right)}\right)}_{\ell \in N}$ by

(131) $$\beta_{•}^{\left(m\right)}\;:=\;1-\frac{\kappa_{•}}{\sigma^{2}m}\;\mathrm{and}\;\alpha_{•}^{\left(m\right)}\;:=\;\beta_{•}^{\left(m\right)}\cdot \frac{\eta }{\sigma^{2}}.$$

Here and henceforth, we always assume that the approximation step *m* is large enough to ensure that $\beta_{•}^{\left(m\right)}\in ]0,1]$ and at least one of $\beta_{A}^{\left(m\right)}$, $\beta_{H}^{\left(m\right)}$ is strictly less than 1; this will be abbreviated by $m\in \bar{N}$. Let us point out that – as mentioned above–our choice entails the best-to-handle setup $P_{\mathrm{NI}}\cup P_{\mathrm{SP},1}$ (which does not happen if instead of η one uses $\eta_{•}$ with $\eta_{A}\neq \eta_{H}$). Based on the GW(I) $X^{\left(m\right)}$, let us construct the *continuous-time* branching process ${\tilde{X}}^{\left(m\right)}:=\left\{{\tilde{X}}_{s}^{\left(m\right)},\;s\in [0,\infty [\right\}$ by

(132) $${\tilde{X}}_{s}^{\left(m\right)}\;:=\;\frac{1}{m}\;X_{\left\lfloor \sigma^{2}ms\right\rfloor }^{\left(m\right)}\;,$$

living on the state space $E^{\left(m\right)}:=\frac{1}{m}N_{0}$. Notice that ${\tilde{X}}^{\left(m\right)}$ is constant on each time-interval $[\frac{k}{\sigma^{2}m}\;,\;\frac{k+1}{\sigma^{2}m}[$ and takes at $s=\frac{k}{\sigma^{2}m}$ the value $\frac{1}{m}X_{k}^{\left(m\right)}$ of the *k*-th GW(I) generation size, divided by *m*, i.e., it “jumps” with the jump-size $\frac{1}{m}\left(X_{k}^{\left(m\right)}-X_{k-1}^{\left(m\right)}\right)$ which is equal to the $\frac{1}{m}$-fold difference to the previous generation size. From (132) one can immediately see the necessity of having σ to be independent of A, H because for the required law-equivalence in (the corresponding version of) (13) both models at stake have to “live” on the same time-scale $\tau_{s}^{\left(m\right)}:=\left\lfloor \sigma {}^{2}ms\right\rfloor$. For this setup, one obtains the following convergenc result:

**Theorem** **10.** *Let η∈[0,∞[, $\kappa_{•}\in [0,\infty [$, σ∈]0,∞[ and ${\tilde{X}}^{\left(m\right)}$ be as defined in (130) to (132). Furthermore, let us suppose that $\lim_{m\to \infty }\frac{1}{m}\;X_{0}^{\left(m\right)}={\tilde{X}}_{0}>0$ and denote by d([0,∞[,[0,∞[) the space of right-continuous functions f:[0,∞[↦[0,∞[ with left limits. Then the sequence of processes ${\left({\tilde{X}}^{\left(m\right)}\right)}_{m\in \bar{N}}$ convergences in distribution in d([0,∞[,[0,∞[) to a diffusion process $\tilde{X}$ which is the unique strong, nonnegative–and in case of $\frac{\eta }{\sigma^{2}}\geq \frac{1}{2}$ strictly positive–solution of the SDE*

(133) $$d{\tilde{X}}_{s}\;=\;\left(\eta \;-\;\kappa_{•}\;{\tilde{X}}_{s}\right)\;ds\;+\;\sigma \sqrt{{\tilde{X}}_{s}}\;dW_{s}^{•},\;s\in [0,\infty \left[,\;{\tilde{X}}_{0}\in \right]0,\infty [\mathrm{given},$$

*where $\left\{W_{s}^{•},\;s\in [0,\infty [\right\}$ denotes a standard Brownian motion with respect to the limit probability measure ${\tilde{P}}_{•}$.*

**Remark** **8.** *Notice that the condition $\frac{\eta }{\sigma^{2}}\geq \frac{1}{2}$ can be interpreted in our approximation setup (131) as $\alpha_{•}^{\left(m\right)}\geq \beta_{•}^{\left(m\right)}/2$, which quantifies the intuitively reasonable indication that if the probability $P_{•}\left[{\tilde{Y}}_{\ell }^{\left(m\right)}=0\right]=e^{-\alpha_{•}^{\left(m\right)}}$ of having no immigration is small enough relative to the probability $P_{•}\left[Y_{\ell -1,k}^{\left(m\right)}=0\right]=e^{-\beta_{•}^{\left(m\right)}}$ of having no offspring ($m\in \bar{N}$), then the limiting diffusion $\tilde{X}$ never hits zero almost surely.*

The corresponding proof of Theorem 10–which is outlined in Appendix A.4–is an adaption of the proof of Theorem 9.1.3 in Ethier & Kurtz [138] which deals with drift-parameters η=0, $\kappa_{•}=0$ in the SDE (133) whose solution is approached on a σ–independent time scale by a sequence of (critical) Galton-Watson processes without immigration but with general offspring distribution with mean 1 and variance σ. Notice that due to (131) the latter is inconsistent with our Poissonian setup, but this is compensated by our chosen σ–dependent time scale. Other limit investigations for (133) involving offspring/immigration distributions and parametrizations which are also incompatible to ours, are e.g., treated in Sriram [142].

As illustration of our proposed approach, let us give the following

**Figure 4:**
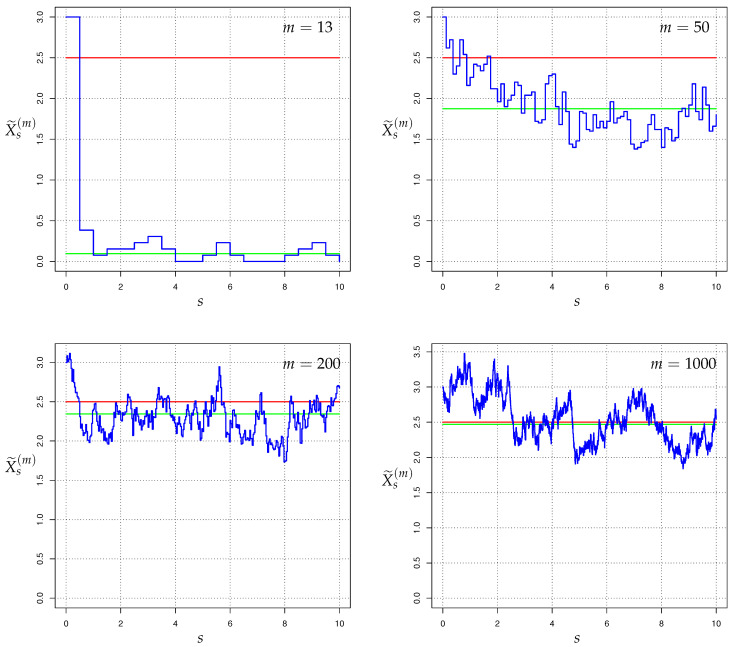
Simulation of the process ${\tilde{X}}_{s}^{\left(m\right)}$ for the approximation steps m∈{13,50,200,1000} in the parameter setup $\left(\eta ,\kappa_{•},\sigma \right)=\left(5,2,0.4\right)$ and with initial starting value ${\tilde{X}}_{0}=3$.

**Example** **3.** *Consider the parameter setup $\left(\eta ,\kappa_{•},\sigma \right)=\left(5,2,0.4\right)$ and initial generation size ${\tilde{X}}_{0}=3$. Figure 4 shows the diffusion-approximation ${\tilde{X}}_{s}^{\left(m\right)}$ (blue) of the corresponding solution ${\tilde{X}}_{s}$ of the SDE (133) up to the time horizon T=10, for the approximation steps m∈{13,50,200,1000}. Notice that in this setup there holds $\bar{N}=\left\{k\in N:\;k\geq 13\right\}$ (recall that $\bar{N}$ is the subset of the positive integers such that $\beta_{•}^{\left(m\right)}=1-\frac{\kappa_{•}}{\sigma^{2}\cdot m}>0$). The “long-term mean” of the limit process ${\tilde{X}}_{s}$ is $\frac{\eta }{\kappa_{•}}=2.5$ and is indicated as red line. The “long-term mean” of the approximations ${\tilde{X}}_{s}^{\left(m\right)}$ is equal to $\frac{\alpha_{•}^{\left(m\right)}}{1-\beta_{•}^{\left(m\right)}}=\frac{\eta }{\kappa_{•}}-\frac{\eta }{\sigma^{2}\cdot m}=2.5-31.25/m$ and is displayed as green line.*

### 7.2. Bounds of Hellinger Integrals for Diffusion Approximations

For each approximation step *m* and each observation horizon t∈[0,∞[, let us now investigate the behaviour of the Hellinger integrals $H_{\lambda }\left(P_{A,t}^{(m),CdA}∥P_{H,t}^{(m),CdA}\right)$, where $P_{•,t}^{(m),CdA}$ denotes the canonical law (under H resp. A) of the *continuous-time diffusion approximation*${\tilde{X}}^{\left(m\right)}$ (cf. (132)), restricted to [0,t]. It is easy to see that $H_{\lambda }\left(P_{A,t}^{(m),CdA}∥P_{H,t}^{(m),CdA}\right)$ coincides with $H_{\lambda }\left(P_{A,\left\lfloor \sigma^{2}mt\right\rfloor }^{\left(m\right)}∥P_{H,\left\lfloor \sigma^{2}mt\right\rfloor }^{\left(m\right)}\right)$ of the law restrictions of the GW(I) generations sizes ${\left(X_{\ell }^{\left(m\right)}\right)}_{\ell \in \{0,\ldots ,\left\lfloor \sigma^{2}mt\right\rfloor \}}$, where $\frac{\left\lfloor \sigma^{2}mt\right\rfloor }{\sigma^{2}m}$ can be interpreted as the last “jump-time” of ${\tilde{X}}^{\left(m\right)}$ before *t*. These Hellinger integrals obey the results of

- the Propositions 2 and 3 (for η=0) respectively the Propositions 4 and 5 (for η∈]0,∞[), as far as recursively computable exact values are concerned,
- Theorem 5 as far as closed-form bounds are concerned; recall that the current setup is of type $P_{\mathrm{NI}}\cup P_{\mathrm{SP},1}$, and thus we can use the simplifications proposed in the Remark 7(a).

In order to obtain the desired Hellinger integral limits $\lim_{m\to \infty }H_{\lambda }\left(P_{A,\left\lfloor \sigma^{2}mt\right\rfloor }^{\left(m\right)}∥P_{H,\left\lfloor \sigma^{2}mt\right\rfloor }^{\left(m\right)}\right)$, one faces several technical problems which will be described in the following. To begin with, for fixed $m\in \bar{N}$ we apply the Propositions 2(b), 3(b), 4(b), 5(b) to the current setup $\left(\beta_{A}^{\left(m\right)},\beta_{H}^{\left(m\right)},\alpha_{A}^{\left(m\right)},\alpha_{H}^{\left(m\right)}\right)\in P_{\mathrm{NI}}\cup P_{\mathrm{SP},1}$ with

$$\beta_{•}^{\left(m\right)}\;:=\;\beta_{•}\left(m,\kappa_{•},\sigma^{2}\right)\;:=\;1-\frac{\kappa_{•}}{\sigma^{2}m}\;\mathrm{and}\;\alpha_{•}^{\left(m\right)}\;:=\;\alpha_{•}\left(m,\kappa_{•},\sigma^{2},\eta \right)\;:=\;\beta_{•}^{\left(m\right)}\cdot \frac{\eta }{\sigma^{2}}\;\left(\mathrm{cf}.(131)\right).$$

Notice that η=0 corresponds to the no-immigration (NI) case and that $\frac{\alpha_{•}^{\left(m\right)}}{\beta_{•}^{\left(m\right)}}=\frac{\eta }{\sigma^{2}}$. Accordingly, we set $\alpha_{\lambda }^{\left(m\right)}:=\lambda \cdot \alpha_{A}^{\left(m\right)}+\left(1-\lambda \right)\cdot \alpha_{H}^{\left(m\right)},\;\beta_{\lambda }^{\left(m\right)}:=\lambda \cdot \beta_{A}^{\left(m\right)}+\left(1-\lambda \right)\cdot \beta_{H}^{\left(m\right)}$. By using

(134) $$q_{\lambda }^{\left(m\right)}\;:=\;q\left(m,\kappa_{•},\sigma^{2},\lambda \right)\;:=\;{\left(\beta_{A}^{\left(m\right)}\right)}^{\lambda }{\left(\beta_{H}^{\left(m\right)}\right)}^{1-\lambda }\;,\;\lambda \in R\backslash \left\{0,1\right\},$$

as well as the connected sequence ${\left(a_{n}^{\left(m\right)}\right)}_{n\in N}:={\left(a_{n}^{\left(q_{\lambda }^{\left(m\right)}\right)}\right)}_{n\in N}$ we arrive at the

**Corollary** **13.** *For all $\left(\beta_{A}^{\left(m\right)},\beta_{H}^{\left(m\right)},\alpha_{A}^{\left(m\right)},\alpha_{H}^{\left(m\right)},\lambda \right)\in \left(P_{\mathrm{NI}}\cup P_{\mathrm{SP},1}\right)\times \left(R\backslash \left\{0,1\right\}\right)$ and all population sizes $X_{0}^{\left(m\right)}\in N$ there holds*

(135) $$h_{\lambda }^{\left(m\right)}\;:=\;H_{\lambda }\left(P_{A,\left\lfloor \sigma^{2}mt\right\rfloor }^{\left(m\right)}∥P_{H,\left\lfloor \sigma^{2}mt\right\rfloor }^{\left(m\right)}\right)\;=\;\exp\left\{a_{\left\lfloor \sigma^{2}mt\right\rfloor }^{\left(q_{\lambda }^{\left(m\right)}\right)}\cdot X_{0}^{\left(m\right)}\;+\;\frac{\eta }{\sigma^{2}}\sum_{k=1}^{\left\lfloor \sigma^{2}mt\right\rfloor }a_{k}^{\left(q_{\lambda }^{\left(m\right)}\right)}\right\}$$

*with η=0 in the NI case.*

In the following, we employ the SDE-parameter constellations (which are consistent with (131) in combination with our requirement to work here only on $\left(P_{\mathrm{NI}}\cup P_{\mathrm{SP},1}\right)$)

(136) $$\begin{array}{ccc} {\tilde{P}}_{NI} & := & \left\{\left(\kappa_{A},\kappa_{H},\eta \right),\;\eta =0,\;\kappa_{A}\in [0,\infty [\;,\;\kappa_{H}\in [0,\infty [\;,\;\kappa_{A}\neq \kappa_{H}\right\}, \end{array}$$

(137) $$\begin{array}{ccc} {\tilde{P}}_{SP,1} & := & \left\{\left(\kappa_{A},\kappa_{H},\eta \right),\;\eta >0,\;\kappa_{A}\in [0,\infty [\;,\;\kappa_{H}\in [0,\infty [\;,\;\kappa_{A}\neq \kappa_{H}\right\}\;. \end{array}$$

Due to the–not in closed-form representable–recursive nature of the sequences ${\left(a_{n}^{\left(q\right)}\right)}_{n\in N}$ defined by (36), the calculation of $\lim_{m\to \infty }h_{\lambda }^{\left(m\right)}$ in (135) seems to be not (straightforwardly) tractable; after all, one “has to move along” a *sequence* of recursions (roughly speaking) since $\left\lfloor \sigma^{2}mt\right\rfloor \to \infty$ as *m* tends to infinity. One way to “circumvent” such technical problems is to compute instead of the limit $\lim_{m\to \infty }h_{\lambda }^{\left(m\right)}$ of the (exact values of the) Hellinger integrals $h_{\lambda }^{\left(m\right)}$, the limits of the corresponding (explicit) closed-form lower resp. upper bounds adapted from Theorem 5. In order to achieve this, one first needs a preparatory step, due to the fact that the sequence ${\left(a_{\left\lfloor \sigma^{2}mt\right\rfloor }^{\left(q_{\lambda }^{\left(m\right)}\right)}\right)}_{m\in \bar{N}}$ (and hence its bounds leading to closed-form expressions) does not necessarily converge for all λ∈R\[0,1]; roughly, this can be conjectured from the Propositions 3(c) and 5(c) in combination with $\left\lfloor \sigma^{2}mt\right\rfloor \to \infty$. Correspondingly, for our “sequence-of-recursions” context equipped with the diffusion-limit’s drift-parameter constellations $\left(\kappa_{A},\kappa_{H},\eta \right)$ we have to derive a “convergence interval” $\left[{\tilde{\lambda }}_{-},{\tilde{\lambda }}_{+}\right]\backslash \left[0,1\right]$ which replaces the single-recursion-concerning $\left[\lambda_{-},\lambda_{+}\right]\backslash \left[0,1\right]$ (cf. Lemma 1). This amounts to

**Proposition** **15.** *For all $\left(\kappa_{A},\kappa_{H},\eta \right)\in {\tilde{P}}_{\mathrm{NI}}\cup {\tilde{P}}_{\mathrm{SP},1}$ define*

(138) $$0\;>\;{\tilde{\lambda }}_{-}\;:=\;\left\{\begin{array}{cc} -\;\infty , & \mathrm{if}\;\kappa_{A}<\kappa_{H}\;,\\ -\;\frac{\kappa_{H}^{2}}{\kappa_{A}^{2}-\kappa_{H}^{2}}, & \mathrm{if}\;\kappa_{A}>\kappa_{H}\;, \end{array}\right.\;\mathrm{and}\;1\;<\;{\tilde{\lambda }}_{+}\;:=\;\left\{\begin{array}{cc} \frac{\kappa_{H}^{2}}{\kappa_{H}^{2}-\kappa_{A}^{2}}, & \mathrm{if}\;\kappa_{A}<\kappa_{H}\;,\\ \infty , & \mathrm{if}\;\kappa_{A}>\kappa_{H}\;. \end{array}\right.$$

*Then, for all $\left(\kappa_{A},\kappa_{H},\eta ,\lambda \right)\in \left({\tilde{P}}_{\mathrm{NI}}\;\cup \;{\tilde{P}}_{\mathrm{SP},1}\right)\;\times \;]{\tilde{\lambda }}_{-},{\tilde{\lambda }}_{+}[\;\backslash \;\left[0,1\right]$ there holds for all sufficiently large $m\in \bar{N}$*

(139) $$q_{\lambda }^{\left(m\right)}\;:=\;{\left(1-\frac{\kappa_{A}}{\sigma^{2}m}\right)}^{\lambda }{\left(1-\frac{\kappa_{H}}{\sigma^{2}m}\right)}^{1-\lambda }\;<\;\min\left\{1\;,\;e^{\beta_{\lambda }^{\left(m\right)}-1}\right\}\;,$$

*and thus the sequence ${\left(a_{n}^{\left(q_{\lambda }^{\left(m\right)}\right)}\right)}_{n\in N}$ converges to the fixed point $x_{0}^{\left(m\right)}\in \;]0,-\log\left(q_{\lambda }^{\left(m\right)}\right)[$.*

This will be proved in Appendix A.4.

We are now in the position to determine bounds of the Hellinger integral limits $\lim_{m\to \infty }H_{\lambda }\left(P_{A,\left\lfloor \sigma^{2}mt\right\rfloor }^{\left(m\right)}∥P_{H,\left\lfloor \sigma^{2}mt\right\rfloor }^{\left(m\right)}\right)$ in form of *m*-limits of appropriate versions of closed-form bounds from Section 6. For the sake of brevity, let us henceforth use the abbreviations $x_{0}^{\left(m\right)}:=x_{0}^{\left(q_{\lambda }^{\left(m\right)}\right)}$, $\Gamma_{<}^{\left(m\right)}:=\Gamma_{<}^{\left(q_{\lambda }^{\left(m\right)}\right)}=\frac{q_{\lambda }^{\left(m\right)}}{2}\cdot e^{x_{0}^{\left(m\right)}}\cdot {\left(x_{0}^{\left(m\right)}\right)}^{2}$, $\Gamma_{>}^{\left(m\right)}:=\Gamma_{>}^{\left(q_{\lambda }^{\left(m\right)}\right)}=\frac{q_{\lambda }^{\left(m\right)}}{2}\cdot {\left(x_{0}^{\left(m\right)}\right)}^{2}$, $d^{(m),S}\;:=\;d^{(q_{\lambda }^{\left(m\right)}),S}\;=\;\frac{x_{0}^{\left(m\right)}-\left(q_{\lambda }^{\left(m\right)}-\beta_{\lambda }^{\left(m\right)}\right)}{x_{0}^{\left(m\right)}}$ and $d^{(m),T}\;:=\;d^{(q_{\lambda }^{\left(m\right)}),T}\;=\;q_{\lambda }^{\left(m\right)}\cdot e^{x_{0}^{\left(m\right)}}.$ By the above considerations, the Theorem 5 (together with Remark 7(a)) adapts to the current setup as follows:

**Corollary** **14.** *(a) For all $\left(\kappa_{A},\kappa_{H},\eta ,\lambda \right)\in \left({\tilde{P}}_{NI}\cup {\tilde{P}}_{SP,1}\right)\times ]0,1[$, all t∈[0,∞[, all approximation steps $m\in \bar{N}$ and all initial population sizes $X_{0}^{\left(m\right)}\in N$ the Hellinger integral can be bounded by*

(140) $$\begin{array}{ccc} C_{\lambda ,X_{0}^{\left(m\right)},t}^{(m),L} & := & \exp\{x_{0}^{\left(m\right)}\cdot \left[X_{0}^{\left(m\right)}-\frac{\eta }{\sigma^{2}}\frac{d^{(m),T}}{1-d^{(m),T}}\right]\left(1-{\left(d^{(m),T}\right)}^{\left\lfloor \sigma^{2}mt\right\rfloor }\right)\;+\;x_{0}^{\left(m\right)}\frac{\eta }{\sigma^{2}}\cdot \left\lfloor \sigma^{2}mt\right\rfloor \\ & & \;\;+\;{\underline{\zeta }}_{\left\lfloor \sigma^{2}mt\right\rfloor }^{\left(m\right)}\cdot X_{0}^{\left(m\right)}\;+\;\frac{\eta }{\sigma^{2}}\cdot {\underline{\vartheta }}_{\left\lfloor \sigma^{2}mt\right\rfloor }^{\left(m\right)}\} \end{array}$$

(141) $$\begin{array}{cc} \leq & H_{\lambda }\left(P_{A,\left\lfloor \sigma^{2}mt\right\rfloor }^{\left(m\right)}∥P_{H,\left\lfloor \sigma^{2}mt\right\rfloor }^{\left(m\right)}\right)\\ \leq & \exp\{x_{0}^{\left(m\right)}\cdot \left[X_{0}^{\left(m\right)}-\frac{\eta }{\sigma^{2}}\frac{d^{(m),S}}{1-d^{(m),S}}\right]\left(1-{\left(d^{(m),S}\right)}^{\left\lfloor \sigma^{2}mt\right\rfloor }\right)\;+\;x_{0}^{\left(m\right)}\frac{\eta }{\sigma^{2}}\cdot \left\lfloor \sigma^{2}mt\right\rfloor \\ & \;\;-\;{\bar{\zeta }}_{\left\lfloor \sigma^{2}mt\right\rfloor }^{\left(m\right)}\cdot X_{0}^{\left(m\right)}\;-\;\frac{\eta }{\sigma^{2}}\cdot {\bar{\vartheta }}_{\left\lfloor \sigma^{2}mt\right\rfloor }^{\left(m\right)}\}\;=:\;C_{\lambda ,X_{0}^{\left(m\right)},t}^{(m),U}\;, \end{array}$$

*where we define analogously to (98) to (101)*

(142) $$\begin{array}{ccc} {\underline{\zeta }}_{n}^{\left(m\right)} & := & \Gamma_{<}^{\left(m\right)}\cdot \frac{{\left(d^{(m),T}\right)}^{n-1}}{1-d^{(m),T}}\cdot \left(1-{\left(d^{(m),T}\right)}^{n}\right)\;>\;0\;, \end{array}$$

(143) $$\begin{array}{ccc} {\underline{\vartheta }}_{n}^{\left(m\right)} & := & \Gamma_{<}^{\left(m\right)}\cdot \frac{1-{\left(d^{(m),T}\right)}^{n}}{{\left(1-d^{(m),T}\right)}^{2}}\cdot \left[1-\frac{d^{(m),T}\left(1+{\left(d^{(m),T}\right)}^{n}\right)}{1+d^{(m),T}}\right]\;>\;0\;, \end{array}$$

(144) $$\begin{array}{ccc} {\bar{\zeta }}_{n}^{\left(m\right)} & := & \Gamma_{<}^{\left(m\right)}\cdot \left[\frac{{\left(d^{(m),S}\right)}^{n}-{\left(d^{(m),T}\right)}^{n}}{d^{(m),S}-d^{(m),T}}-{\left(d^{(m),S}\right)}^{n-1}\cdot \frac{1-{\left(d^{(m),T}\right)}^{n}}{1-d^{(m),T}}\right]\;>\;0\;, \end{array}$$

(145) $$\begin{array}{ccc} {\bar{\vartheta }}_{n}^{\left(m\right)} & := & \Gamma_{<}^{\left(m\right)}\cdot \frac{d^{(m),T}}{1-d^{(m),T}}\cdot \left[\frac{1-{\left(d^{(m),S}d^{(m),T}\right)}^{n}}{1-d^{(m),S}d^{(m),T}}-\frac{{\left(d^{(m),S}\right)}^{n}-{\left(d^{(m),T}\right)}^{n}}{d^{(m),S}-d^{(m),T}}\right]\;>\;0\;.\; \end{array}$$

*Notice that (140) and (141) simplify significantly for $\left(\kappa_{A},\kappa_{H},\eta ,\lambda \right)\in {\tilde{P}}_{NI}\times ]0,1[$ for which η=0 holds.*

*(b) For all $\left(\kappa_{A},\kappa_{H},\eta ,\lambda \right)\in \left({\tilde{P}}_{\mathrm{NI}}\cup {\tilde{P}}_{\mathrm{SP},1}\right)\;\times \;]{\tilde{\lambda }}_{-},{\tilde{\lambda }}_{+}[\;\backslash \;\left[0,1\right]$ and all initial population sizes $X_{0}^{\left(m\right)}\in N$ the Hellinger integral bounds (140) and (141) are valid for all sufficiently large $m\in \bar{N}$, where the expressions (142) to (145) have to be replaced by*

(146) $$\begin{array}{ccc} {\underline{\zeta }}_{n}^{\left(m\right)} & := & \Gamma_{>}^{\left(m\right)}\cdot \frac{{\left(d^{(m),T}\right)}^{n}-{\left(d^{(m),S}\right)}^{2n}}{d^{(m),T}-{\left(d^{(m),S}\right)}^{2}}\;>\;0\;, \end{array}$$

(147) $$\begin{array}{ccc} {\underline{\vartheta }}_{n}^{\left(m\right)} & := & \frac{\Gamma_{>}^{\left(m\right)}}{d^{(m),T}-{\left(d^{(m),S}\right)}^{2}}\cdot \left[\frac{d^{(m),T}\cdot \left(1-{\left(d^{(m),T}\right)}^{n}\right)}{1-d^{(m),T}}-\frac{{\left(d^{(m),S}\right)}^{2}\cdot \left(1-{\left(d^{(m),S}\right)}^{2n}\right)}{1-{\left(d^{(m),S}\right)}^{2}}\right]\;>\;0\;,\\ {\bar{\zeta }}_{n}^{\left(m\right)} & := & \Gamma_{>}^{\left(m\right)}\cdot {\left(d^{(m),S}\right)}^{n-1}\cdot \left[n\;-\;\frac{1-{\left(d^{(m),T}\right)}^{n}}{1-d^{(m),T}}\right]\;>\;0\;, \end{array}$$

(148) $$\begin{array}{ccc} {\bar{\vartheta }}_{n}^{\left(m\right)} & := & \Gamma_{>}^{\left(m\right)}\cdot [\frac{d^{(m),S}-d^{(m),T}}{{\left(1-d^{(m),S}\right)}^{2}\left(1-d^{(m),T}\right)}\cdot \left(1-{\left(d^{(m),S}\right)}^{n}\right) \end{array}$$

(149) $$\begin{array}{c} \;+\;\frac{d^{(m),T}\left(1-{\left(d^{(m),S}d^{(m),T}\right)}^{n}\right)}{\left(1-d^{(m),T}\right)\left(1-d^{(m),S}d^{(m),T}\right)}-\frac{{\left(d^{(m),S}\right)}^{n}}{1-d^{(m),S}}\cdot n]\;.\; \end{array}$$

Let us finally present the desired assertions on the limits of the bounds given in Corollary 14 as the approximation step *m* tends to infinity, by employing for $\lambda \in ]{\tilde{\lambda }}_{-},{\tilde{\lambda }}_{+}[\;⊋\left[0,1\right]$ the quantities

(150) $$\kappa_{\lambda }\;:=\;\lambda \kappa_{A}+\left(1-\lambda \right)\kappa_{H}\;\mathrm{as}\mathrm{well}\mathrm{as}\;\Lambda_{\;\lambda }\;:=\;\sqrt{\lambda \kappa_{A}^{2}+\left(1-\lambda \right)\kappa_{H}^{2}}\;,$$

for which the following relations hold:

(151) $$\begin{array}{cc} \Lambda_{\lambda }\;>\kappa_{\lambda }\;>\;0, & \;\mathrm{for}\;\lambda \in \;]0,1[,\; \end{array}$$

(152) $$\begin{array}{cc} 0\;<\;\Lambda_{\lambda }\;<\kappa_{\lambda }, & \;\mathrm{for}\;\lambda \in \;]{\tilde{\lambda }}_{-},{\tilde{\lambda }}_{+}[\backslash \left[0,1\right]\;. \end{array}$$

**Theorem** **11.** *Let the initial SDE-value ${\tilde{X}}_{0}\in ]0,\infty [$ be arbitrary but fixed, and suppose that $\lim_{m\to \infty }\frac{1}{m}\;X_{0}^{\left(m\right)}={\tilde{X}}_{0}$. Then, for all $\left(\kappa_{A},\kappa_{H},\eta ,\lambda \right)\in \left({\tilde{P}}_{NI}\cup {\tilde{P}}_{SP,1}\right)\;\times \;]{\tilde{\lambda }}_{-}\;,\;{\tilde{\lambda }}_{+}[\;\backslash \;\left\{0,1\right\}$ and all t∈[0,∞[ the Hellinger integral limit can be bounded by*

(153) $$\begin{array}{ccc} D_{\lambda ,{\tilde{X}}_{0},t}^{L} & := & \exp\{-\frac{\Lambda_{\;\lambda }-\kappa_{\lambda }}{\sigma^{2}}\left[{\tilde{X}}_{0}-\frac{\eta }{\Lambda_{\;\lambda }}\right]\left(1-e^{-\Lambda_{\;\lambda }\cdot t}\right)-\frac{\eta }{\sigma^{2}}\left(\Lambda_{\;\lambda }-\kappa_{\lambda }\right)\cdot t\\ & & \;\;+\;L_{\lambda }^{\left(1\right)}\left(t\right)\cdot {\tilde{X}}_{0}\;+\;\frac{\eta }{\sigma^{2}}\cdot L_{\lambda }^{\left(2\right)}\left(t\right)\}\; \end{array}$$

(154) $$\begin{array}{cc} \leq & \lim_{m\to \infty }H_{\lambda }\left(P_{A,\left\lfloor \sigma^{2}mt\right\rfloor }^{\left(m\right)}∥P_{H,\left\lfloor \sigma^{2}mt\right\rfloor }^{\left(m\right)}\right)\\ \leq & \exp\{-\frac{\Lambda_{\;\lambda }-\kappa_{\lambda }}{\sigma^{2}}\left[{\tilde{X}}_{0}-\frac{\eta }{\frac{1}{2}\left(\Lambda_{\;\lambda }+\kappa_{\lambda }\right)}\right]\left(1-e^{-\frac{1}{2}\left(\Lambda_{\;\lambda }+\kappa_{\lambda }\right)\cdot t}\right)-\frac{\eta }{\sigma^{2}}\left(\Lambda_{\;\lambda }-\kappa_{\lambda }\right)\cdot t\;\\ & \;\;-\;U_{\lambda }^{\left(1\right)}\left(t\right)\cdot {\tilde{X}}_{0}\;-\;\frac{\eta }{\sigma^{2}}\cdot U_{\lambda }^{\left(2\right)}\left(t\right)\}\;=:\;d_{\lambda ,{\tilde{X}}_{0},t}^{U}\;, \end{array}$$

*where for the (sub)case of all λ∈]0,1[ and all t≥0*

(155) $$\begin{array}{ccc} L_{\lambda }^{\left(1\right)}\left(t\right) & := & \frac{{\left(\Lambda_{\;\lambda }-\kappa_{\lambda }\right)}^{2}}{2\sigma^{2}\cdot \Lambda_{\;\lambda }}\cdot e^{-\Lambda_{\;\lambda }\cdot t}\cdot \left(1-e^{-\Lambda_{\;\lambda }\cdot t}\right)\;, \end{array}$$

(156) $$\begin{array}{ccc} L_{\lambda }^{\left(2\right)}\left(t\right) & := & \frac{1}{4}\cdot {\left(\frac{\Lambda_{\;\lambda }-\kappa_{\lambda }}{\Lambda_{\;\lambda }}\right)}^{2}\cdot {\left(1-e^{-\Lambda_{\;\lambda }\cdot t}\right)}^{2}\;, \end{array}$$

(157) $$\begin{array}{ccc} U_{\lambda }^{\left(1\right)}\left(t\right) & := & \frac{{\left(\Lambda_{\;\lambda }-\kappa_{\lambda }\right)}^{2}}{\sigma^{2}}\cdot \left[\frac{e^{-\frac{1}{2}\left(\Lambda_{\;\lambda }+\kappa_{\lambda }\right)\cdot t}-e^{-\Lambda_{\;\lambda }\cdot t}}{\Lambda_{\;\lambda }-\kappa_{\lambda }}-\frac{e^{-\frac{1}{2}\left(\Lambda_{\;\lambda }+\kappa_{\lambda }\right)\cdot t}\left(1-e^{-\Lambda_{\;\lambda }\cdot t}\right)}{2\cdot \Lambda_{\;\lambda }}\right]\;,\; \end{array}$$

(158) $$\begin{array}{ccc} U_{\lambda }^{\left(2\right)}\left(t\right) & := & \frac{{\left(\Lambda_{\;\lambda }-\kappa_{\lambda }\right)}^{2}}{\Lambda_{\;\lambda }}\cdot \left[\frac{1-e^{-\frac{1}{2}\left(3\Lambda_{\;\lambda }+\kappa_{\lambda }\right)\cdot t}}{3\Lambda_{\;\lambda }+\kappa_{\lambda }}+\frac{e^{-\Lambda_{\;\lambda }\cdot t}-e^{-\frac{1}{2}\left(\Lambda_{\;\lambda }+\kappa_{\lambda }\right)\cdot t}}{\Lambda_{\;\lambda }-\kappa_{\lambda }}\right]\;,\; \end{array}$$

*and for the remaining (sub)case of all $\lambda \in ]{\tilde{\lambda }}_{-},{\tilde{\lambda }}_{+}[\;\backslash \;\left[0,1\right]$ and all t≥0*

(159) $$\begin{array}{ccc} L_{\lambda }^{\left(1\right)}\left(t\right) & := & \frac{{\left(\Lambda_{\;\lambda }-\kappa_{\lambda }\right)}^{2}}{2\sigma^{2}\cdot \kappa_{\lambda }}\cdot e^{-\Lambda_{\;\lambda }\cdot t}\cdot \left(1-e^{-\kappa_{\lambda }\cdot t}\right)\;, \end{array}$$

(160) $$\begin{array}{ccc} L_{\lambda }^{\left(2\right)}\left(t\right) & := & \frac{{\left(\Lambda_{\lambda }-\kappa_{\lambda }\right)}^{2}}{2\cdot \kappa_{\lambda }}\cdot \left[\frac{1-e^{-\Lambda_{\lambda }\cdot t}}{\Lambda_{\lambda }}-\frac{1-e^{-(\Lambda_{\lambda }+\kappa_{\lambda })\cdot t}}{\Lambda_{\lambda }+\kappa_{\lambda }}\right]\;, \end{array}$$

(161) $$\begin{array}{ccc} U_{\lambda }^{\left(1\right)}\left(t\right) & := & \frac{{\left(\Lambda_{\;\lambda }-\kappa_{\lambda }\right)}^{2}}{2\cdot \sigma^{2}}\cdot e^{-\frac{1}{2}\left(\Lambda_{\lambda }+\kappa_{\lambda }\right)\cdot t}\cdot \left[t\;-\;\frac{1-e^{-\Lambda_{\lambda }\cdot t}}{\Lambda_{\lambda }}\right]\;, \end{array}$$

(162) $$\begin{array}{ccc} U_{\lambda }^{\left(2\right)}\left(t\right) & := & {\left(\Lambda_{\lambda }-\kappa_{\lambda }\right)}^{2}\cdot \left[\frac{\left(\Lambda_{\lambda }-\kappa_{\lambda }\right)\left(1-e^{-\frac{1}{2}\left(\Lambda_{\lambda }+\kappa_{\lambda }\right)\cdot t}\right)}{\Lambda_{\lambda }\cdot {\left(\Lambda_{\lambda }+\kappa_{\lambda }\right)}^{2}}+\;\frac{1-e^{-\frac{1}{2}\left(3\Lambda_{\lambda }+\kappa_{\lambda }\right)\cdot t}}{\Lambda_{\lambda }\cdot \left(3\Lambda_{\lambda }+\kappa_{\lambda }\right)}-\frac{e^{-\frac{1}{2}\left(\Lambda_{\lambda }+\kappa_{\lambda }\right)\cdot t}}{\Lambda_{\lambda }+\kappa_{\lambda }}\cdot t\right]\;. \end{array}$$

*Notice that the components $L_{\lambda }^{\left(i\right)}\left(t\right)$ and $U_{\lambda }^{\left(i\right)}\left(t\right)$(for i=1,2 and in both cases λ∈]0,1[ and $\lambda \in ]{\tilde{\lambda }}_{-},{\tilde{\lambda }}_{+}[\;\backslash \;\left[0,1\right]$) are strictly positive for t>0 and do not depend on the parameter η. Furthermore, the bounds $d_{\lambda ,{\tilde{X}}_{0},t}^{L}$ and $d_{\lambda ,{\tilde{X}}_{0},t}^{U}$ simplify significantly in the case $\left(\kappa_{A},\kappa_{H},\eta \right)\in {\tilde{P}}_{NI}$, for which η=0 holds.*

This will be proved in Appendix A.4. For the time-asymptotics, we obtain the

**Corollary** **15.** *Let the initial SDE-value ${\tilde{X}}_{0}\in ]0,\infty [$ be arbitrary but fixed, and suppose that $\lim_{m\to \infty }\frac{1}{m}\;X_{0}^{\left(m\right)}={\tilde{X}}_{0}$. Then:*

*(a) For all $\left(\kappa_{A},\kappa_{H},\eta ,\lambda \right)\in {\tilde{P}}_{NI}\times ]{\tilde{\lambda }}_{-},{\tilde{\lambda }}_{+}[\;\backslash \left\{0,1\right\}$ the Hellinger integral limit converges to*

$$\lim_{t\to \infty }\;\lim_{m\to \infty }\log\left(H_{\lambda }\left(P_{A,\left\lfloor \sigma^{2}mt\right\rfloor }^{\left(m\right)}∥P_{H,\left\lfloor \sigma^{2}mt\right\rfloor }^{\left(m\right)}\right)\right)\;=\;-\;\frac{{\tilde{X}}_{0}}{\sigma^{2}}\cdot \left(\Lambda_{\lambda }-\kappa_{\lambda }\right)\;\left\{\begin{array}{cc} <0, & \mathrm{for}\;\lambda \in ]0,1[\;,\\ >0, & \mathrm{for}\;\lambda \in ]{\tilde{\lambda }}_{-},{\tilde{\lambda }}_{+}[\;\backslash \;\left[0,1\right]\;. \end{array}\right.$$

*(b) For all $\left(\kappa_{A},\kappa_{H},\eta ,\lambda \right)\in {\tilde{P}}_{SP,1}\times ]{\tilde{\lambda }}_{-},{\tilde{\lambda }}_{+}[\;\backslash \left\{0,1\right\}$ the Hellinger integral limit possesses the asymptotical behaviour*

$$\lim_{t\to \infty }\frac{1}{t}\log\left(\lim_{m\to \infty }H_{\lambda }\left(P_{A,\left\lfloor \sigma^{2}mt\right\rfloor }^{\left(m\right)}∥P_{H,\left\lfloor \sigma^{2}mt\right\rfloor }^{\left(m\right)}\right)\right)\;=\;-\;\frac{\eta }{\sigma^{2}}\cdot \left(\Lambda_{\lambda }-\kappa_{\lambda }\right)\;\left\{\begin{array}{cc} <0, & \mathrm{for}\;\lambda \in ]0,1[\;,\\ >0, & \mathrm{for}\;\lambda \in ]{\tilde{\lambda }}_{-},{\tilde{\lambda }}_{+}[\;\backslash \;\left[0,1\right]\;. \end{array}\right.$$

The assertions of Corollary 15 follow immediately by inspecting the expressions in the exponential of (153) and (154) in combination with (155) to (162).

### 7.3. Bounds of Power Divergences for Diffusion Approximations

Analogously to Section 4 (see especially Section 4.1), for orders λ∈R\{0,1} all the results of the previous Section 7.2 carry correspondingly over from (limits of) bounds of the Hellinger integrals $H_{\lambda }\left(P_{A,\left\lfloor \sigma^{2}mt\right\rfloor }^{\left(m\right)}∥P_{H,\left\lfloor \sigma^{2}mt\right\rfloor }^{\left(m\right)}\right)$ to (limits of) bounds of the total variation distance $V\left(P_{A,\left\lfloor \sigma^{2}mt\right\rfloor }^{\left(m\right)}∥P_{H,\left\lfloor \sigma^{2}mt\right\rfloor }^{\left(m\right)}\right)$ (by virtue of (12)), to (limits of) bounds of the Renyi divergences $R_{\lambda }\left(P_{A,\left\lfloor \sigma^{2}mt\right\rfloor }^{\left(m\right)}∥P_{H,\left\lfloor \sigma^{2}mt\right\rfloor }^{\left(m\right)}\right)$ (by virtue of (7)) as well as to (limits of) bounds of the power divergences $I_{\lambda }\left(P_{A,\left\lfloor \sigma^{2}mt\right\rfloor }^{\left(m\right)}∥P_{H,\left\lfloor \sigma^{2}mt\right\rfloor }^{\left(m\right)}\right)$ (by virtue of (2)). For the sake of brevity, the–merely repetitive–exact details are omitted. Moreover, by combining the outcoming results on the above-mentioned power divergences with parts of the Bayesian-decision-making context of Section 2.5, we obtain corresponding assertions on (i) the (cf. (21)) *weighted-average* decision risk reduction (weighted-average statistical information measure) about the degree of evidence deg concerning the parameter θ that can be attained by observing the GWI-path $X_{n}$ until stage *n*, as well as (ii) the (cf. (22)) *limit* decision risk reduction (limit statistical information measure).

In the following, let us concentrate on the derivation of the Kullback-Leibler information divergence KL (relative entropy) within the current diffusion-limit framework. Notice that altogether we face two limit procedures simultaneously: by the first limit $\lim_{\lambda ↑1}I_{\lambda }\left(P_{A,\left\lfloor \sigma^{2}mt\right\rfloor }^{\left(m\right)}∥P_{H,\left\lfloor \sigma^{2}mt\right\rfloor }^{\left(m\right)}\right)$ we obtain the KL $I\left(P_{A,\left\lfloor \sigma^{2}mt\right\rfloor }^{\left(m\right)}∥P_{H,\left\lfloor \sigma^{2}mt\right\rfloor }^{\left(m\right)}\right)$ for every fixed approximation step $m\in \bar{N}$; on the other hand, for each fixed λ∈]0,1[, the second limit $\lim_{m\to \infty }I_{\lambda }\left(P_{A,\left\lfloor \sigma^{2}mt\right\rfloor }^{\left(m\right)}∥P_{H,\left\lfloor \sigma^{2}mt\right\rfloor }^{\left(m\right)}\right)$ describes the limit of the power divergence – as the sequence of rescaled and continuously interpolated GW(I)’s ${\left({\left({\tilde{X}}_{s}^{\left(m\right)}\right)}_{s\in [0,\infty [}\right)}_{m\in \bar{N}}$(equipped with probability law $P_{A,\left\lfloor \sigma^{2}mt\right\rfloor }^{\left(m\right)}$ resp. $P_{H,\left\lfloor \sigma^{2}mt\right\rfloor }^{\left(m\right)}$ up to time $\left\lfloor \sigma^{2}mt\right\rfloor$) converges weakly to the continuous-time CIR-type diffusion process ${\left({\tilde{X}}_{s}\right)}_{s\in [0,\infty [}$ (with probability law ${\tilde{P}}_{A,t}$ resp. ${\tilde{P}}_{H,t}$ up to time *t*). In Appendix A.4 we shall prove that these two limits can be interchanged:

**Theorem** **12.** *Let the initial SDE-value ${\tilde{X}}_{0}\in ]0,\infty [$ be arbitrary but fixed, and suppose that $\lim_{m\to \infty }\frac{1}{m}\;X_{0}^{\left(m\right)}={\tilde{X}}_{0}$. Then, for all $\left(\kappa_{A},\kappa_{H},\eta \right)\in {\tilde{P}}_{NI}\cup {\tilde{P}}_{SP,1}$ and all t∈[0,∞[, one gets the Kullback-Leibler information divergence (relative entropy) convergences*

(163) $$\begin{array}{c} \lim_{m\to \infty }I\left(P_{A,\left\lfloor \sigma^{2}mt\right\rfloor }^{\left(m\right)}∥P_{H,\left\lfloor \sigma^{2}mt\right\rfloor }^{\left(m\right)}\right)\;=\;\lim_{m\to \infty }\lim_{\lambda ↗1}I_{\lambda }\left(P_{A,\left\lfloor \sigma^{2}mt\right\rfloor }^{\left(m\right)}∥P_{H,\left\lfloor \sigma^{2}mt\right\rfloor }^{\left(m\right)}\right)\\ =\left\{\begin{array}{cc} \frac{{\left(\kappa_{A}-\kappa_{H}\right)}^{2}}{2\sigma^{2}\cdot \kappa_{A}}\cdot \left[\left({\tilde{X}}_{0}-\frac{\eta }{\kappa_{A}}\right)\cdot \left(1-e^{-\kappa_{A}\cdot t}\right)+\eta \cdot t\right]\;, & \mathrm{if}\;\;\kappa_{A}>0,\\ \frac{\kappa_{H}^{2}}{2\sigma^{2}}\cdot \left[\frac{\eta }{2}\cdot t^{2}\;+\;{\tilde{X}}_{0}\cdot t\right]\;, & \mathrm{if}\;\;\kappa_{A}=0, \end{array}\right.\\ =\;\;\lim_{\lambda ↗1}\lim_{m\to \infty }I_{\lambda }\left(P_{A,\left\lfloor \sigma^{2}mt\right\rfloor }^{\left(m\right)}∥P_{H,\left\lfloor \sigma^{2}mt\right\rfloor }^{\left(m\right)}\right)\;. \end{array}$$

This immediately leads to the following

**Corollary** **16.** *Let the initial SDE-value ${\tilde{X}}_{0}\in ]0,\infty [$ be arbitrary but fixed, and suppose that $\lim_{m\to \infty }\frac{1}{m}\;X_{0}^{\left(m\right)}={\tilde{X}}_{0}$. Then, the KL limit (163) possesses the following time-asymptotical behaviour:*

*(a) For all $\left(\kappa_{A},\kappa_{H},\eta \right)\in {\tilde{P}}_{NI}$ (i.e., η=0) one gets*

$$\begin{array}{ccc} \left(i\right)\; & \mathrm{in}\;\mathrm{the}\;\mathrm{case}\;\kappa_{A}>0 & \;\lim_{t\to \infty }\lim_{m\to \infty }I\left(P_{A,\left\lfloor \sigma^{2}mt\right\rfloor }^{\left(m\right)}∥P_{H,\left\lfloor \sigma^{2}mt\right\rfloor }^{\left(m\right)}\right)\;=\;\frac{{\tilde{X}}_{0}\cdot {\left(\kappa_{A}-\kappa_{H}\right)}^{2}}{2\sigma^{2}\cdot \kappa_{A}}\;,\;\\ \left(\mathrm{ii}\right)\; & \mathrm{in}\;\mathrm{the}\;\mathrm{case}\;\kappa_{A}=0 & \;\lim_{t\to \infty }\lim_{m\to \infty }\frac{1}{t}\cdot I\left(P_{A,\left\lfloor \sigma^{2}mt\right\rfloor }^{\left(m\right)}∥P_{H,\left\lfloor \sigma^{2}mt\right\rfloor }^{\left(m\right)}\right)\;=\;\frac{{\tilde{X}}_{0}\cdot \kappa_{H}^{2}}{4\sigma^{2}}\;. \end{array}$$

*(b) For all $\left(\kappa_{A},\kappa_{H},\eta \right)\in {\tilde{P}}_{SP,1}$ (i.e., η>0) one gets*

$$\begin{array}{ccc} \left(i\right)\; & \mathrm{in}\;\mathrm{the}\;\mathrm{case}\;\kappa_{A}>0 & \;\lim_{t\to \infty }\lim_{m\to \infty }\frac{1}{t}\cdot I\left(P_{A,\left\lfloor \sigma^{2}mt\right\rfloor }^{\left(m\right)}∥P_{H,\left\lfloor \sigma^{2}mt\right\rfloor }^{\left(m\right)}\right)\;=\;\frac{\eta \cdot {\left(\kappa_{A}-\kappa_{H}\right)}^{2}}{2\sigma^{2}\cdot \kappa_{A}}\;,\;\\ \left(\mathrm{ii}\right)\; & \mathrm{in}\;\mathrm{the}\;\mathrm{case}\;\kappa_{A}=0 & \;\lim_{t\to \infty }\lim_{m\to \infty }\frac{1}{t^{2}}\cdot I\left(P_{A,\left\lfloor \sigma^{2}mt\right\rfloor }^{\left(m\right)}∥P_{H,\left\lfloor \sigma^{2}mt\right\rfloor }^{\left(m\right)}\right)\;=\;\frac{\eta \cdot \kappa_{H}^{2}}{4\sigma^{2}}\;. \end{array}$$

**Remark** **9.** *In Appendix A.4 we shall see that the proof of the last (limit-interchange concerning) equality in (163) relies heavily on the use of the extra terms $L_{\lambda }^{\left(1\right)}\left(t\right),\;L_{\lambda }^{\left(2\right)}\left(t\right),\;U_{\lambda }^{\left(1\right)}\left(t\right),\;U_{\lambda }^{\left(2\right)}\left(t\right)$ in (153) and (154). Recall that these terms ultimately stem from (manipulations of) the corresponding parts of the “improved closed-form bounds” in Theorem 5, which were derived by using the linear inhomogeneous difference equations ${\underline{a}}_{n}^{\left(q\right)}$ resp. ${\bar{a}}_{n}^{\left(q\right)}$ (cf. (92) resp. (94)) instead of the linear homogeneous difference equations $a_{n}^{(q),T}$ resp. $a_{n}^{(q),S}$ (cf. (78) resp. (79)) as explicit approximates of the sequence $a_{n}^{\left(q\right)}$. Not only this fact shows the importance of this more tedious approach.*

Interesting comparisons of the above-mentioned results in Section 7.2 and Section 7.3 with corresponding information measures of the solutions of the SDE (129) themselves (rather their branching approximations), can be found in Kammerer [157].

### 7.4. Applications to Decision Making

Analogously to Section 6.7, the above-mentioned investigations of the Section 7.1, Section 7.2 and Section 7.3 can be applied to the context of Section 2.5 on *dichotomous* decision making about GW(I)-type diffusion approximations of solutions of the stochastic differential Equation (129). For the sake of brevity, the–merely repetitive–exact details are omitted.

## Appendix A. Proofs and Auxiliary Lemmas

### Appendix A.1. Proofs and Auxiliary Lemmas for Section 3

**Lemma** **A1.**
*For all real numbers x,y,z>0 and all λ∈R one has*

$$x^{\lambda }y^{1-\lambda }\;-\;\left(\lambda \;x\;z^{\lambda -1}\;+\;\left(1-\lambda \right)\;y\;z^{\lambda }\right)\;\left\{\begin{array}{cc} \leq \;0, & \mathrm{for}\;\lambda \in ]0,1[\;,\\ =\;0, & \mathrm{for}\;\lambda \in \{0,1\}\;,\\ \geq \;0, & \mathrm{for}\;\lambda \in R\backslash [0,1]\;, \end{array}\right.$$

*with equality in the cases λ∈R\{0,1} iff $\frac{x}{y}=z$.*

**Proof of Lemma** **A1.** For fixed $\tilde{x}:=xz^{\lambda -1}>0$, $\tilde{y}:=yz^{\lambda }>0$ with $\tilde{x}\neq \tilde{y}$ we inspect the function *g* on R defined by $g\left(\lambda \right):=\;{\tilde{x}}^{\lambda }{\tilde{y}}^{1-\lambda }-\left(\lambda \tilde{x}+\left(1-\lambda \right)\tilde{y}\right)$ which satisfies g(0)=g(1)=0, $g^{\prime }\left(0\right)=\tilde{y}\log\left(\tilde{x}/\tilde{y}\right)-\left(\tilde{x}-\tilde{y}\right)\;<\;\tilde{y}\left(\left(\tilde{x}/\tilde{y}\right)-1\right)-\left(\tilde{x}-\tilde{y}\right)\;=\;0$ and which is strictly convex. Thus, the assertion follows immediately by taking into account the obvious case $\tilde{x}=\tilde{y}$. □

**Proof of** **Properties 1.** Property (P9) is trivially valid. To show (P1) we assume $0<q<\beta_{\lambda }$, which implies $a_{1}^{\left(q\right)}=\xi_{\lambda }^{\left(q\right)}\left(0\right)=q-\beta_{\lambda }<0$. By induction, ${\left(a_{n}\right)}_{n\in N}$ is strictly negative and strictly decreasing. As stated in (P9), the function $\xi_{\lambda }^{\left(q\right)}$ is strictly increasing, strictly convex and converges to $-\beta_{\lambda }$ for x→−∞. Thus, it hits the straight line id(x)=x once and only once on the negative real line at $x_{0}^{\left(q\right)}\in ]-\beta_{\lambda },0[$ (cf. (44)). This implies that the sequence ${\left(a_{n}^{\left(q\right)}\right)}_{n\in N}$ converges to $x_{0}^{\left(q\right)}\in ]-\beta_{\lambda },q-\beta_{\lambda }[$. Property (P2) follows immediately. In order to prove (P3), let us fix $q>\max\{0,\beta_{\lambda }\}$, implying $a_{1}^{\left(q\right)}=\xi_{\lambda }^{\left(q\right)}\left(0\right)=q-\beta_{\lambda }>0$; notice that in this setup, the special choice q=1 implies $\min\left\{1,e^{\beta_{\lambda }-1}\right\}=e^{\beta_{\lambda }-1}<q$. By induction, ${\left(a_{n}^{\left(q\right)}\right)}_{n\in N}$ is strictly positive and strictly increasing. Since $\lim_{x\to \infty }\xi_{\lambda }^{\left(q\right)}\left(x\right)=\infty$, the function $\xi_{\lambda }^{\left(q\right)}$ does not necessarily hit the straight line id(x)=x on the positive real line. In fact, due to strict convexity (cf. (P9)), this is excluded if $\xi_{\lambda }^{(q)\prime }\left(0\right)=q\geq 1$. Suppose that q<1. To prove that there exists a positive solution of the equation $\xi_{\lambda }^{\left(q\right)}\left(x\right)=x$ it is sufficient to show that the unique global minimum of the strict convex function $h_{\lambda }^{\left(q\right)}\left(x\right):=\xi_{\lambda }^{\left(q\right)}\left(x\right)-x$ is taken at some point $x_{0}\in ]0,\infty [$ and that $h_{\lambda }^{\left(q\right)}\left(x_{0}\right)\leq 0$. It holds $h_{\lambda }^{(q)\prime }\left(x\right)=q\cdot e^{x}-1$, and therefore $h_{\lambda }^{(q)\prime }\left(x\right)=0$ iff $x=x_{0}=-\log q$. We have $h_{\lambda }^{\left(q\right)}\left(-\log q\right)=1-\beta_{\lambda }+\log q$, which is less or equal to zero iff $q\leq e^{\beta_{\lambda }-1}$. It remains to show that for $q>\beta_{\lambda }$ and $q>\min\left\{1\;,\;e^{\beta_{\lambda }-1}\right\}$ the sequence ${\left(a_{n}^{\left(q\right)}\right)}_{n\in N}$ grows faster than exponentially, i.e., there do not exist constants $c_{1},c_{2}\in R$ such that $a_{n}^{\left(q\right)}\leq e^{c_{1}+c_{2}n}$ for all n∈N. We already know that (in the current case) $a_{n}^{\left(q\right)}\overset{n\to \infty }{⟶}\infty$. Notice that it is sufficient to verify ${lim sup}_{n\to \infty }\left(\log\left(a_{n+1}^{\left(q\right)}\right)-\log\left(a_{n}^{\left(q\right)}\right)\right)=\infty$. For the case $\beta_{\lambda }\geq 0$ the latter is obtained by

$$\begin{array}{ccc} \log\left(a_{n+1}^{\left(q\right)}\right)-\log\left(a_{n}^{\left(q\right)}\right) & = & \log\left(\left(q-\beta_{\lambda }\right)e^{a_{n}^{\left(q\right)}}+\beta_{\lambda }\left(e^{a_{n}^{\left(q\right)}}-1\right)\right)-\log\left(qe^{a_{n-1}^{\left(q\right)}}-\beta_{\lambda }\right)\\ & \geq & \left(\log\left(q-\beta_{\lambda }\right)-\log\left(q\right)\right)+\left(qe^{a_{n-1}^{\left(q\right)}}-\beta_{\lambda }-a_{n-1}^{\left(q\right)}\right)\;\overset{a_{n-1}^{\left(q\right)}\to \infty }{⟶}\;\infty \;. \end{array}$$

An analogous consideration works out for the case $\beta_{\lambda }<0$. Property (P4) is trivial, and (P5) to (P8) are direct implications of the already proven properties (P1) to (P4). □

**Proof of Lemma** **1.** (a) Let $\beta_{A}>0$, $\beta_{H}>0$ with $\beta_{A}\neq \beta_{H}$, λ∈R\]0,1[, $\beta_{\lambda }:=\lambda \beta_{A}+\left(1-\lambda \right)\beta_{H}$ and $q_{\lambda }:=\beta_{A}^{\lambda }\beta_{H}^{1-\lambda }>\max\left\{0,\beta_{\lambda }\right\}$ (cf. Lemma A1). Below, we follow the lines of Linkov & Lunyova [53], appropriately adapted to our context. We have to find those λ∈R\]0,1[ for which the following two conditions hold:

(i) $q_{\lambda }\leq 1$, i.e., $\xi_{\lambda }^{(q_{\lambda })\;\prime }\left(0\right)\leq 1$,
(ii) $q_{\lambda }\leq e^{\beta_{\lambda }-1}$ (cf.(P3a)), which is equivalent with the existence of a–positive, if (i) is satisfied,–solution of the equation $\xi_{\lambda }^{\left(q_{\lambda }\right)}\left(x\right)=x$.

Notice that the case $q_{\lambda }=1$, λ∈R\[0,1], cannot appear in (i), provided that (ii) holds (since due to Lemma A1$e^{\beta_{\lambda }-1}<e^{q_{\lambda }-1}=1$). For (i), it is easy to check that we have to require

(A1) $$\lambda \;\left\{\begin{array}{cc} < & \frac{\log(\beta_{H})}{\log(\beta_{H}/\beta_{A})},\;\mathrm{if}\;\beta_{A}>\beta_{H},\\ > & \frac{\log(\beta_{H})}{\log(\beta_{H}/\beta_{A})},\;\mathrm{if}\;\beta_{A}<\beta_{H}. \end{array}\right.$$

To proceed, straightforward analysis leads to $-\log\left(q_{\lambda }\right)=\arg\min_{x\in R}\left\{\xi_{\lambda }^{\left(q_{\lambda }\right)}\left(x\right)-x\right\}$. To check (ii), we first notice that $q_{\lambda }\leq e^{\beta_{\lambda }-1}$ iff $\xi_{\lambda }^{\left(q_{\lambda }\right)}\left(x\right)-x\leq 0$ for some x∈R. Hence, we calculate

(A2) $$\begin{array}{c} \xi_{\lambda }^{\left(q_{\lambda }\right)}\left(-\log(q_{\lambda })\right)+\log\left(q_{\lambda }\right)\;\leq \;0\;⟺\;1-\lambda \left(\beta_{A}-\beta_{H}\right)-\beta_{H}+\lambda \log\left(\frac{\beta_{A}}{\beta_{H}}\right)+\log\left(\beta_{H}\right)\;\leq \;0\\ ⟺\;\lambda \cdot \left[\beta_{H}\left(1-\frac{\beta_{A}}{\beta_{H}}\right)+\log\left(\frac{\beta_{A}}{\beta_{H}}\right)\right]\;\leq \;\beta_{H}-1-\log\left(\beta_{H}\right). \end{array}$$

In order to isolate λ in (A2), one has to find out for which $\left(\beta_{A},\beta_{H}\right)$ the term in the square bracket is positive resp. zero resp. negative. To achieve this, we aim for the substitutions $x:=\beta_{A}/\beta_{H}$, $\beta =\beta_{H}$ and thus study first the auxiliary function $h_{\beta }\left(x\right):=\log\left(x\right)-\beta \left(x-1\right)$, x>0, with fixed parameters β>0. Straightforwardly, we obtain $h_{\beta }^{\prime }\left(x\right)=x^{-1}-\beta$ and $h_{\beta }^{\prime \prime }\left(x\right)=-x^{-2}$. Thus, the function $h_{\beta }\left(\cdot \right)$ is strictly concave and attains a maximum at $x=\beta^{-1}$. Since additionally $h_{\beta }\left(1\right)=0$ and $h_{\beta }^{\prime }\left(1\right)=1-\beta$, there exists a second solution z(β)≠1 of the equation $h_{\beta }\left(x\right)=0$ iff β≠1. Thus, one gets

- for β=1: for all x>0 there holds $h_{\beta }\left(x\right)\leq 0$, with equality iff $x=\beta^{-1}$,
- for β<1: $h_{\beta }\left(x\right)\geq 0$ iff x∈[1,z(β)], with equality iff x∈{1,z(β)} (notice that z(β)>1),
- for β>1: $h_{\beta }\left(x\right)\geq 0$ iff x∈[z(β),1], with equality iff x∈{z(β),1} (notice that z(β)<1).

Suppose that λ<0. **Case 1:** If $\beta_{H}=1$, then condition (ii) is not satisfied whenever $\beta_{A}\neq \beta_{H}$, since the right side of (A2) is equal to zero and the left side is strictly greater than zero. Hence, $\lambda_{-}=0$. **Case 2:** Let $\beta_{H}>1$. If $\beta_{A}<\beta_{H}$, then condition (i) is not satisfied and hence $\lambda_{-}=0$. If $\beta_{A}>\beta_{H}$, then condition (i) is satisfied iff $\lambda <\overset{˘}{\overset{˘}{\lambda }}:=\overset{˘}{\overset{˘}{\lambda }}\left(\beta_{A},\beta_{H}\right):=\frac{\log(\beta_{H})}{\log(\beta_{H}/\beta_{A})}<0$. On the other hand, incorporating the discussion of the function $h_{\beta }\left(\cdot \right)$, we see that $h_{\beta_{H}}\left(\frac{\beta_{A}}{\beta_{H}}\right)<0$. Thus, (A2) implies that condition (ii) is satisfied when $\lambda \geq \overset{˘}{\lambda }:=\overset{˘}{\lambda }\left(\beta_{A},\beta_{H}\right):=\frac{\beta_{H}-1-\log\left(\beta_{H}\right)}{\beta_{H}-\beta_{A}+\log\left(\frac{\beta_{A}}{\beta_{H}}\right)}$. We claim that $\overset{˘}{\overset{˘}{\lambda }}<\overset{˘}{\lambda }$ and conclude that the conditions (i) and (ii) are not fulfilled jointly, which leads to $\lambda_{-}=0$. To see this, we notice that due to $1<\beta_{H}<\beta_{A}$ we get $\log\left(\beta_{A}\right)/\left(\beta_{A}-1\right)<\log\left(\beta_{H}\right)/\left(\beta_{H}-1\right)$ and thus

(A3) $$\begin{array}{cc} & \log\left(\beta_{A}\right)\left(\beta_{H}-1\right)\;<\;\log\left(\beta_{H}\right)\left(\beta_{A}-1\right)\\ ⟺ & \beta_{H}\log\left(\beta_{H}\right)-\beta_{A}\log\left(\beta_{H}\right)\;<\;\beta_{H}\log\left(\beta_{H}\right)-\beta_{H}\log\left(\beta_{A}\right)-\log\left(\beta_{H}\right)+\log\left(\beta_{A}\right)\\ ⟺ & \log\left(\beta_{H}\right)\left(\beta_{H}-\beta_{A}\right)+\log\left(\beta_{H}\right)\log\left(\frac{\beta_{A}}{\beta_{H}}\right)\;<\;\log\left(\frac{\beta_{H}}{\beta_{A}}\right)\left(\beta_{H}-1\right)+\log\left(\beta_{H}\right)\log\left(\frac{\beta_{A}}{\beta_{H}}\right)\\ ⟺ & \frac{\log(\beta_{H})}{\log\left(\frac{\beta_{H}}{\beta_{A}}\right)}\;<\;\frac{\beta_{H}-1-\log\left(\beta_{H}\right)}{\beta_{H}-\beta_{A}+\log\left(\frac{\beta_{A}}{\beta_{H}}\right)}\;⟺\;\overset{˘}{\overset{˘}{\lambda }}\;<\;\overset{˘}{\lambda }\;. \end{array}$$

**Case 3:** Let $\beta_{H}<1$. For this, one gets $h_{\beta_{H}}\left(\frac{\beta_{A}}{\beta_{H}}\right)\geq 0$ for $\beta_{A}\in ]\beta_{H},\beta_{H}z\left(\beta_{H}\right)]$. Hence, condition (ii) is satisfied if either $\beta_{A}\in ]\beta_{H},\beta_{H}z\left(\beta_{H}\right)]$, or $\beta_{A}\notin ]\beta_{H},\beta_{H}z\left(\beta_{H}\right)]$ and $\lambda \geq \overset{˘}{\lambda }$. If $\beta_{A}>\beta_{H}z\left(\beta_{H}\right)$, then condition (i) is trivially satisfied for all λ<0. In the case $\beta_{A}<\beta_{H}$, condition (i) is satisfied whenever $\lambda >\overset{˘}{\overset{˘}{\lambda }}$. Notice that since $0<\beta_{A}<\beta_{H}<1$, an analogous consideration as in (A3) leads to $\overset{˘}{\overset{˘}{\lambda }}<\overset{˘}{\lambda }$. This implies that $\lambda_{-}=\overset{˘}{\lambda }$. The last case $\beta_{A}\in ]\beta_{H},\beta_{H}z\left(\beta_{H}\right)]$ is easy to handle: since $\frac{\log(\beta_{H})}{\log(\beta_{H}/\beta_{A})}>0$ as well as $z_{\beta_{H}}\left(\frac{\beta_{A}}{\beta_{H}}\right)>0$, both conditions (i) and (ii) hold trivially. The representation of $\lambda_{+}$ follows straightforwardly from the $\lambda_{-}$-result and the skew symmetry (8), by employing $1-\overset{˘}{\lambda }\left(\beta_{H},\beta_{A}\right)=\overset{˘}{\lambda }\left(\beta_{A},\beta_{H}\right)$. Alternatively, one can proceed analogously to the $\lambda_{-}$-case. Part (b) is much easier to prove: if $\beta_{•}:=\beta_{A}=\beta_{H}>0$, then for all λ∈R\[0,1] one gets $q_{\lambda }=\beta_{A}^{\lambda }\beta_{H}^{1-\lambda }=\beta_{•}$ as well as $\beta_{\lambda }=\beta_{•}$. Hence, Properties 1 (P2) implies that $a_{n}^{\left(q_{\lambda }\right)}\equiv 0$ and thus it is convergent, independently of the choice λ∈R\[0,1]. □

**Proof of Formula** **(51).** For the parameter constellation in Section 3.10, we employ as upper bound for $\phi_{\lambda }\left(x\right)$ ($x\in N_{0}$) the function

$$\bar{\phi_{\lambda }}\left(x\right):=\left\{\begin{array}{cc} \phi_{\lambda }\left(0\right), & \mathrm{if}x=0,\\ 0, & \mathrm{if}x>0. \end{array}\right.$$

Notice that this method is rather crude, and gives in the other cases treated in the Section 3.7, Section 3.8 and Section 3.9 worse bounds than those derived there. Since λ∈]0,1[ and $\alpha_{A}\neq \alpha_{H}$, one has $\phi_{\lambda }\left(0\right)<0$. In order to derive an upper bound of the Hellinger integral, we first set $\bar{\epsilon }:=1-e^{\phi_{\lambda }\left(0\right)}\in ]0,1[$. Hence, for all n∈N\{1} we obtain the auxiliary expression

$$\begin{array}{c} \sum_{x_{n-1}=0}^{\infty }\frac{{\left[\varphi_{\lambda }\left(x_{n-2}\right)\right]}^{x_{n-1}}}{x_{n-1}!}\cdot \exp\left\{\phi_{\lambda }\left(x_{n-1}\right)\right\}\;\leq \;\sum_{x_{n-1}=0}^{\infty }\frac{{\left[\varphi_{\lambda }\left(x_{n-2}\right)\right]}^{x_{n-1}}}{x_{n-1}!}\cdot \exp\left\{\bar{\phi_{\lambda }}\left(x_{n-1}\right)\right\}\\ =\;\exp\left\{\varphi_{\lambda }\left(x_{n-2}\right)\right\}-\bar{\epsilon }\;=\;\exp\left\{\varphi_{\lambda }\left(x_{n-2}\right)\right\}\cdot \left[1-\bar{\epsilon }\cdot \exp\left\{-\varphi_{\lambda }\left(x_{n-2}\right)\right\}\right]\;. \end{array}$$

Moreover, since $\beta_{A}\neq \beta_{H}$, one gets $\lim_{x\to \infty }\phi_{\lambda }\left(x\right)=-\infty$ (cf. Properties 3 (P20) and Lemma A1). This–together with the nonnegativity of $\varphi_{\lambda }\left(\cdot \right)$–implies

$$\underset{x\in N_{0}}{\sup}\left\{\exp\left\{\phi_{\lambda }\left(x\right)\right\}\cdot \left[1-\bar{\epsilon }\cdot \exp\left\{-\varphi_{\lambda }\left(x\right)\right\}\right]\right\}\;=:\;\bar{\delta }\in ]0,1[\;.$$

Incorporating these considerations as well as the formulas (27) to (32), we get for n=1 the relation $H_{\lambda }\left(P_{A,n}∥P_{H,n}\right)=\exp\left\{\phi_{\lambda }\left(x_{0}\right)\right\}\leq 1$(with equality iff $x_{0}=x^{*}=\frac{\alpha_{A}-\alpha_{H}}{\beta_{H}-\beta_{A}}$), and–as a continuation of formula (29)– for all n∈N\{1}(recall that $\vec{x}:=\left(x_{0},x_{1},\ldots \right)\in \Omega$)

(A4) $$\begin{array}{c} H_{\lambda }\left(P_{A,n}∥P_{H,n}\right)\;=\;\sum_{x_{1}=0}^{\infty }\cdots \sum_{x_{n}=0}^{\infty }\prod_{k=1}^{n}Z_{n,k}^{\left(\lambda \right)}\left(\vec{x}\right)\\ =\;\sum_{x_{1}=0}^{\infty }\cdots \sum_{x_{n-1}=0}^{\infty }\prod_{k=1}^{n-1}Z_{n,k}^{\left(\lambda \right)}\left(\vec{x}\right)\\ \;\cdot \exp\left\{{\left(f_{A}\left(x_{n-1}\right)\right)}^{\lambda }{\left(f_{H}\left(x_{n-1}\right)\right)}^{\left(1-\lambda \right)}-\left(\lambda f_{A}\left(x_{n-1}\right)+\left(1-\lambda \right)f_{H}\left(x_{n-1}\right)\right)\right\}\\ =\;\sum_{x_{1}=0}^{\infty }\cdots \sum_{x_{n-2}=0}^{\infty }\prod_{k=1}^{n-2}Z_{n,k}^{\left(\lambda \right)}\left(\vec{x}\right)\cdot \exp\left\{-f_{\lambda }\left(x_{n-2}\right)\right\}\sum_{x_{n-1}=0}^{\infty }\frac{{\left[\varphi_{\lambda }\left(x_{n-2}\right)\right]}^{x_{n-1}}}{x_{n-1}!}\cdot \exp\left\{\phi_{\lambda }\left(x_{n-1}\right)\right\}\\ \leq \;\sum_{x_{1}=0}^{\infty }\cdots \sum_{x_{n-2}=0}^{\infty }\prod_{k=1}^{n-2}Z_{n,k}^{\left(\lambda \right)}\left(\vec{x}\right)\cdot \exp\left\{\phi_{\lambda }\left(x_{n-2}\right)\right\}\cdot \left[1-\bar{\epsilon }\cdot \exp\left\{-\varphi_{\lambda }\left(x_{n-2}\right)\right\}\right]\\ \leq \;\bar{\delta }\cdot \sum_{x_{1}=0}^{\infty }\cdots \sum_{x_{n-2}=0}^{\infty }\prod_{k=1}^{n-2}Z_{n,k}^{\left(\lambda \right)}\left(\vec{x}\right)\;\leq \cdots \leq \;{\bar{\delta }}^{\left\lfloor n/2\right\rfloor }\;. \end{array}$$

Hence, $H_{\lambda }\left(P_{A,n}∥P_{H,n}\right)<1$ for (at least) all n∈N\{1}, and $\lim_{n\to \infty }H_{\lambda }\left(P_{A,n}∥P_{H,n}\right)=0$. □

Notice that the above proof method of formula (51) does not work for the parameter setup in Section 3.11, because there one gets $\bar{\delta }=\sup_{x\in N_{0}}\left\{\exp\left\{\phi_{\lambda }\left(x\right)\right\}\cdot \left[1-\bar{\epsilon }\cdot \exp\left\{-\varphi_{\lambda }\left(x\right)\right\}\right]\right\}=1$.

**Proof of Proposition** **9.** In the setup $\left(\beta_{A},\beta_{H},\alpha_{A},\alpha_{H},\lambda \right)\in P_{\mathrm{SP},4a}\times ]0,1[$ we require $\beta_{•}:=\beta_{A}=\beta_{H}<1$. As a linear upper bound for $\phi_{\lambda }\left(\cdot \right)$, we employ the tangent line at y≥0 (cf. (52))

(A5) $$\phi_{\lambda ,y}^{\tan}\left(x\right)\;:=\;\left(p_{y}-\alpha_{\lambda }\right)+\left(q_{y}-\beta_{•}\right)\cdot x:=\left(p_{\lambda ,y}^{\tan}-\alpha_{\lambda }\right)+\left(q_{\lambda ,y}^{\tan}-\beta_{\lambda }\right)\cdot x\;:=\;\left(\phi_{\lambda }\left(y\right)-y\cdot \phi_{\lambda }^{\prime }\left(y\right)\right)+\phi_{\lambda }^{\prime }\left(y\right)\cdot x\;.$$

Since in the current setup $P_{\mathrm{SP},4a}$ the function $\phi_{\lambda }\left(\cdot \right)$ is strictly increasing, the slope $\phi_{\lambda }^{\prime }\left(y\right)$ of the tangent line at *y* is positive. Thus we have $q_{y}>\beta_{\lambda }$ and Properties 1 (P3) implies that the sequence ${\left(a_{n}^{\left(q_{y}\right)}\right)}_{n\in N}$ is strictly increasing and converges to $x_{0}^{\left(q_{y}\right)}\in ]0,-\log\left(q_{y}\right)]$ iff $q_{y}\leq \min\left\{1,e^{\beta_{•}-1}\right\}=e^{\beta_{•}-1}<1$ (cf. (P3a)), where $x_{0}^{\left(q_{y}\right)}$ is the smallest solution of the equation $\xi_{\lambda }^{\left(q_{y}\right)}\left(x\right)=q_{y}\cdot e^{x}-\beta_{•}=x$. Since $q_{y}↘\beta_{•}$ for y→∞ (cf. Properties 3 (P18)) and additionally $e^{\beta_{•}-1}>\beta_{•}$, there exists a large enough y≥0 such that the sequence ${\left(a_{n}^{\left(q_{y}\right)}\right)}_{n\in N}$ converges. If this *y* is also large enough to additionally guarantee h(y)<0 for

$$h\left(y\right)\;:=\;\lim_{n\to \infty }\frac{1}{n}\log\left({\tilde{B}}_{\lambda ,X_{0},n}^{\left(p_{y},q_{y}\right)}\right)\;=\;p_{y}\cdot e^{x_{0}^{\left(q_{y}\right)}}-\alpha_{\lambda }\;,$$

then one can conclude that $\lim_{n\to \infty }H_{\lambda }(P_{A,n}∥P_{H,n})=0$. As a first step, for verifying h(y)<0 we look for an upper bound ${\bar{x}}_{0}^{\left(q_{y}\right)}$ for the fixed point $x_{0}^{\left(q_{y}\right)}$ where the latter exists for $y\geq y_{1}$ (say). Notice that

(A6) $${\bar{Q}}_{\lambda }^{\left(q_{y}\right)}\left(x\right)\;:=\;\frac{1}{2}x^{2}\;+\;q_{y}x\;+\;q_{y}-\beta_{•}\;\geq \;q_{y}\cdot e^{x}\;-\;\beta_{•}\;=\;\xi_{\lambda }^{\left(q_{y}\right)}\left(x\right)\;,$$

since ${\bar{Q}}_{\lambda }^{\left(q_{y}\right)}\left(0\right)=\xi_{\lambda }^{\left(q_{y}\right)}\left(0\right)$, ${\bar{Q}}_{\lambda }^{(q_{y})\;\prime }\left(0\right)=\xi_{\lambda }^{(q_{y})\;\prime }\left(0\right)$ and ${\bar{Q}}_{\lambda }^{(q_{y})\;\prime \prime }\left(x\right)\geq \xi_{\lambda }^{(q_{y})\;\prime \prime }\left(x\right)$ for $x\in [0,-\log\left(q_{y}\right)]$. For sufficiently large $y\geq y_{2}\geq y_{1}$ (say), we easily obtain the smaller solution of ${\bar{Q}}_{\lambda }^{\left(q_{y}\right)}\left(x\right)=x$ as

(A7) $${\bar{x}}_{0}^{\left(q_{y}\right)}=\left(1-q_{y}\right)-\sqrt{{\left(1-q_{y}\right)}^{2}-2\left(q_{y}-\beta_{•}\right)}=\left(1-\phi_{\lambda }^{\prime }\left(y\right)-\beta_{•}\right)-\sqrt{{\left(1-\phi_{\lambda }^{\prime }\left(y\right)-\beta_{•}\right)}^{2}-2\phi_{\lambda }^{\prime }\left(y\right)}\geq x_{0}^{\left(q_{y}\right)}$$

where the expression in the root is positive since $q_{y}↘\beta_{•}$ for y→∞. We now have

(A8) $$h\left(y\right)\;=\;p_{y}\cdot e^{x_{0}^{\left(q_{y}\right)}}-\alpha_{\lambda }\;\leq \;p_{y}\cdot e^{{\bar{x}}_{0}^{\left(q_{y}\right)}}-\alpha_{\lambda }\;=:\;\bar{h}\left(y\right)\;,\;\forall \;y\geq y_{2}\;.$$

Hence, it suffices to show that $\bar{h}\left(y\right)<0$ for some $y\geq y_{2}$. We recall from Properties 3 (P15), (P17) and (P19) that

(A9) $$\begin{array}{ccc} \phi_{\lambda }\left(y\right) & = & {\left(\alpha_{A}+\beta_{•}\cdot y\right)}^{\lambda }{\left(\alpha_{H}+\beta_{•}\cdot y\right)}^{1-\lambda }-\lambda \left(\alpha_{A}+\beta_{•}\cdot y\right)-\left(1-\lambda \right)\left(\alpha_{H}+\beta_{•}\cdot y\right)\;<\;0,\\ \phi_{\lambda }^{\prime }\left(y\right) & = & \lambda \cdot \beta_{•}\cdot {\left(\frac{\alpha_{A}+\beta_{•}\cdot y}{\alpha_{H}+\beta_{•}\cdot y}\right)}^{\lambda -1}\;+\;\left(1-\lambda \right)\cdot \beta_{•}\cdot {\left(\frac{\alpha_{A}+\beta_{•}\cdot y}{\alpha_{H}+\beta_{•}\cdot y}\right)}^{\lambda }\;-\;\beta_{•}\;>\;0\;\mathrm{and}\mathrm{that}\\ \phi_{\lambda }^{\prime \prime }\left(y\right) & = & -\;{\left(\frac{\alpha_{A}+\beta_{•}\cdot y}{\alpha_{H}+\beta_{•}\cdot y}\right)}^{\lambda }\cdot \frac{\lambda \left(1-\lambda \right)\cdot \beta_{•}^{2}\cdot {\left(\alpha_{A}-\alpha_{H}\right)}^{2}}{{\left(\alpha_{A}+\beta_{•}\cdot y\right)}^{2}\left(\alpha_{H}+\beta_{•}\cdot y\right)}\;<\;0\;, \end{array}$$

which immediately implies $\lim_{y\to \infty }\phi_{\lambda }\left(y\right)=\lim_{y\to \infty }\phi_{\lambda }^{\prime }\left(y\right)=\lim_{y\to \infty }\phi_{\lambda }^{\prime \prime }\left(y\right)=0$ and with l’Hospital’s rule

(A10) $$\begin{array}{ccc} \lim_{y\to \infty }y\cdot \phi_{\lambda }\left(y\right) & = & \lim_{y\to \infty }-y^{2}\cdot \phi_{\lambda }^{\prime }\left(y\right)\;=\;\lim_{y\to \infty }\frac{y^{3}}{2}\cdot \phi_{\lambda }^{\prime \prime }\left(y\right)\\ & = & -\frac{1}{2}\lim_{y\to \infty }{\left(\frac{\alpha_{A}+\beta_{•}\cdot y}{\alpha_{H}+\beta_{•}\cdot y}\right)}^{\lambda }\cdot \frac{\lambda \left(1-\lambda \right)\cdot \beta_{•}^{2}\cdot {\left(\alpha_{A}-\alpha_{H}\right)}^{2}}{{\left(\alpha_{A}/y+\beta_{•}\right)}^{2}\left(\alpha_{H}/y+\beta_{•}\right)}\;=\;-\frac{1}{2}\lambda \left(1-\lambda \right)\cdot \frac{{\left(\alpha_{A}-\alpha_{H}\right)}^{2}}{\beta_{•}}\;. \end{array}$$

The formulas (A5), (A7) and (A9) imply the limits $\lim_{y\to \infty }p_{y}=\alpha_{\lambda }$, $\lim_{y\to \infty }q_{y}=\beta_{•}$, $\lim_{y\to \infty }{\bar{x}}_{0}^{\left(q_{y}\right)}=0$. Notice that $p_{y}<\alpha_{\lambda }$ holds trivially for all y≥0 since the intercept $\left(p_{y}\;-\;\alpha_{\lambda }\right)$ of the tangent line $\phi_{\lambda ,y}^{\tan}\left(\cdot \right)$ is negative. Incorporating (A8) we therefore obtain $\lim_{y\to \infty }h\left(y\right)\leq \lim_{y\to \infty }\bar{h}\left(y\right)=0$. As mentioned before, for the proof it is sufficient to show that $\bar{h}\left(y\right)<0$ for some $y\geq y_{2}$. This holds true if $\lim_{y\to \infty }y\cdot \bar{h}\left(y\right)<0$. To verify this, notice first that from (A5), (A7) and (A8) we get

(A11) $${\bar{h}}^{\prime }\left(y\right)\;=\;-p_{y}\cdot e^{{\bar{x}}_{0}^{\left(q_{y}\right)}}\cdot \phi_{\lambda }^{\prime \prime }\left(y\right)\cdot \left[1-\frac{2-\phi_{\lambda }^{\prime }\left(y\right)-\beta_{•}}{\sqrt{{\left(1-q_{y}\right)}^{2}-2\left(q_{y}-\beta_{•}\right)}}\right]\;-\;y\cdot \phi_{\lambda }^{\prime \prime }\left(y\right)\cdot e^{{\bar{x}}_{0}^{\left(q_{y}\right)}}\;\overset{y\to \infty }{⟶}\;0.$$

Finally we obtain with (A10)

$$\begin{array}{ccc} \lim_{y\to \infty }y\cdot \bar{h}\left(y\right) & = & -\;\lim_{y\to \infty }y^{2}\cdot {\bar{h}}^{\prime }\left(y\right)\\ & = & \lim_{y\to \infty }p_{y}\cdot e^{{\bar{x}}_{0}^{\left(q_{y}\right)}}\cdot y^{2}\cdot \phi_{\lambda }^{\prime \prime }\left(y\right)\cdot \left[1-\frac{2-\phi_{\lambda }^{\prime }\left(y\right)-\beta_{•}}{\sqrt{{\left(1-q_{y}\right)}^{2}-2\left(q_{y}-\beta_{•}\right)}}\right]\;+\;y^{3}\cdot \phi_{\lambda }^{\prime \prime }\left(y\right)\cdot e^{{\bar{x}}_{0}^{\left(q_{y}\right)}}\\ & = & 0\;-\;\lambda \left(1-\lambda \right)\cdot \frac{{\left(\alpha_{A}-\alpha_{H}\right)}^{2}}{\beta_{•}}\;<\;0\;.\;\;\;□ \end{array}$$

**Proof of Corollary** **1.** Part (a) follows directly from Proposition 1 (a),(b) and the limit $\lim_{n\to \infty }H_{\lambda }(P_{A,n}∥P_{H,n})=0$ in the respective part (c) of the Propositions 7, 8, 9 as well as from (51). To prove part (b), according to (26) we have to verify ${lim inf}_{\lambda ↗1}\left\{{lim inf}_{n\to \infty }H_{\lambda }\left(P_{A,n}∥P_{H,n}\right)\right\}\;=\;1$. From part (c) of Proposition 2 we see that this is satisfied iff $\lim_{\lambda ↑1}x_{0}^{\left(q_{\lambda }^{E}\right)}=0$. Recall that for fixed λ∈]0,1[ we have $\beta_{\lambda }=\lambda \beta_{A}+\left(1-\lambda \right)\beta_{H}>0$, $q_{\lambda }^{E}=\beta_{A}^{\lambda }\beta_{H}^{1-\lambda }<\beta_{\lambda }$ (cf. Lemma A1) and from Properties 1 (P1) the unique negative solution $x_{0}^{\left(q_{\lambda }^{E}\right)}\in ]-\beta_{\lambda },q_{\lambda }^{E}-\beta_{\lambda }[$ of $\xi_{\lambda }^{\left(q_{\lambda }^{E}\right)}\left(x\right)=q_{\lambda }^{E}e^{x}-\beta_{\lambda }=x$ (cf. (44)). Due to the continuity and boundedness of the map $\lambda \mapsto x_{0}^{\left(q_{\lambda }^{E}\right)}$ (for λ∈[0,1]) one gets that $\lim_{\lambda ↗1}x_{0}^{\left(q_{\lambda }^{E}\right)}$ exists and is the smallest nonpositive solution of $\beta_{A}e^{x}-\beta_{A}=x$. From this, the part (b) as well as the non-contiguity in part (c) follow immediately. The other part of (c) is a direct consequence of Proposition 1 (a),(b) and Proposition 2 (c). □

**Proof of Formula** **(59)**. One can proceed similarly to the proof of formula (51) above. Recall $H_{\lambda }(P_{A,1}∥P_{H,1})=\exp\left\{\phi_{\lambda }\left(X_{0}\right)\right\}>1$ for $X_{0}\in N$(cf. (28), Lemma A1 and $f_{A}\left(X_{0}\right)\neq f_{H}\left(X_{0}\right)$ for all $X_{0}\in N$). For $\left(\beta_{A},\beta_{H},\alpha_{A},\alpha_{H},\lambda \right)\in P_{\mathrm{SP},2}\times \left(R\backslash \left[0,1\right]\right)$ one gets $\phi_{\lambda }\left(0\right)=0,\;\phi_{\lambda }\left(1\right)>0$, and we define for x≥0

$$\underline{\phi_{\lambda }}\left(x\right):=\left\{\begin{array}{cc} \phi_{\lambda }\left(1\right)\;, & \mathrm{if}x=1,\\ 0, & \mathrm{if}x\neq 1. \end{array}\right.$$

By means of the choice $\underline{\epsilon }:=\varphi_{\lambda }\left(1\right)\cdot \left(e^{\phi_{\lambda }\left(1\right)}-1\right)>0$, we obtain for all n∈N\{1}

$$\begin{array}{c} \sum_{x_{n-1}=0}^{\infty }\frac{{\left[\varphi_{\lambda }\left(x_{n-2}\right)\right]}^{x_{n-1}}}{x_{n-1}!}\cdot \exp\left\{\phi_{\lambda }\left(x_{n-1}\right)\right\}\;\geq \;\sum_{x_{n-1}=0}^{\infty }\frac{{\left[\varphi_{\lambda }\left(x_{n-2}\right)\right]}^{x_{n-1}}}{x_{n-1}!}\cdot \exp\left\{\underline{\phi_{\lambda }}\left(x_{n-1}\right)\right\}\\ =\;\exp\left\{\varphi_{\lambda }\left(x_{n-2}\right)\right\}+\underline{\epsilon }\;=\;\exp\left\{\varphi_{\lambda }\left(x_{n-2}\right)\right\}\cdot \left[1+\underline{\epsilon }\cdot \exp\left\{-\varphi_{\lambda }\left(x_{n-2}\right)\right\}\right]\;. \end{array}$$

Incorporating

$$\underset{x\in N_{0}}{\inf}\left\{\exp\left\{\phi_{\lambda }\left(x\right)\right\}\cdot \left[1+\underline{\epsilon }\cdot \exp\left\{-\varphi_{\lambda }\left(x\right)\right\}\right]\right\}\;=:\;\underline{\delta }\;>\;1\;,$$

one can show analogously to (A4) that

$$H_{\lambda }\left(P_{A,n}∥P_{H,n}\right)\;\geq \;\cdots \;\geq \;{\underline{\delta }}^{\left\lfloor n/2\right\rfloor }\;\overset{n\to \infty }{⟶}\;\infty .\;\;\;□$$

**Proof of the Formulas** **(61), (63) and (64).** In the following, we slightly adapt the above-mentioned proof of formula (59). Let us define

$$\underline{\phi_{\lambda }}\left(x\right):=\left\{\begin{array}{cc} \phi_{\lambda }\left(0\right)\;, & \mathrm{if}x=0,\\ 0, & \mathrm{if}x>0. \end{array}\right.$$

In all respective subcases one clearly has $\underline{\phi_{\lambda }}\left(0\right)=\phi_{\lambda }\left(0\right)>0$. With $\underline{\epsilon }:=e^{\phi_{\lambda }\left(0\right)}-1>0$ we obtain for all n∈N\{1}

$$\begin{array}{c} \sum_{x_{n-1}=0}^{\infty }\frac{{\left[\varphi_{\lambda }\left(x_{n-2}\right)\right]}^{x_{n-1}}}{x_{n-1}!}\cdot \exp\left\{\phi_{\lambda }\left(x_{n-1}\right)\right\}\;\geq \;\sum_{x_{n-1}=0}^{\infty }\frac{{\left[\varphi_{\lambda }\left(x_{n-2}\right)\right]}^{x_{n-1}}}{x_{n-1}!}\cdot \exp\left\{\underline{\phi_{\lambda }}\left(x_{n-1}\right)\right\}\\ =\;\exp\left\{\varphi_{\lambda }\left(x_{n-2}\right)\right\}+\underline{\epsilon }\;=\;\exp\left\{\varphi_{\lambda }\left(x_{n-2}\right)\right\}\cdot \left[1+\underline{\epsilon }\cdot \exp\left\{-\varphi_{\lambda }\left(x_{n-2}\right)\right\}\right]\;. \end{array}$$

By employing

(A12) $$\underset{x\in N_{0}}{\inf}\left\{\exp\left\{\phi_{\lambda }\left(x\right)\right\}\cdot \left[1+\underline{\epsilon }\cdot \exp\left\{-\varphi_{\lambda }\left(x\right)\right\}\right]\right\}\;=:\;\underline{\delta }>\;1\;,$$

one can show analogously to (A4) that

$$H_{\lambda }\left(P_{A,n}∥P_{H,n}\right)\;\geq \;\cdots \;\geq \;{\underline{\delta }}^{\left\lfloor n/2\right\rfloor }\;\overset{n\to \infty }{⟶}\;\infty .$$

Notice that this method does not work for the parameter cases $P_{\mathrm{SP},4a}\cup P_{\mathrm{SP},4b}$, since there the infimum in (A12) is equal to one. □

**Proof of Proposition** **13.** In the setup $\left(\beta_{A},\beta_{H},\alpha_{A},\alpha_{H},\lambda \right)\in P_{\mathrm{SP},4a}\times \left(R\backslash \left[0,1\right]\right)$ we require $\beta_{•}:=\beta_{A}=\beta_{H}<1$. As in the proof of Proposition 9, we stick to the tangent line $\phi_{\lambda ,y}^{\tan}\left(\cdot \right)$ at y≥0 (cf. (52)) as a linear lower bound for $\phi_{\lambda }\left(\cdot \right)$, i.e., we use the function

(A13) $$\phi_{\lambda ,y}^{\tan}\left(x\right)\;:=\;\left(p_{y}-\alpha_{\lambda }\right)+\left(q_{y}-\beta_{•}\right)\cdot x\;:=\;\left(p_{\lambda ,y}^{\tan}-\alpha_{\lambda }\right)+\left(q_{\lambda ,y}^{\tan}-\beta_{\lambda }\right)\cdot x\;:=\;\left(\phi_{\lambda }\left(y\right)-y\cdot \phi_{\lambda }^{\prime }\left(y\right)\right)\;+\;\phi_{\lambda }^{\prime }\left(y\right)\cdot x\;.$$

As already mentioned in Section 3.21, on $P_{\mathrm{SP},4a}$ the function $\phi_{\lambda }\left(\cdot \right)$ is strictly decreasing and converges to 0. Thus, for all y≥0 the slope $\phi_{\lambda }^{\prime }\left(y\right)$ of the tangent line at *y* is negative, which implies that $q_{y}<\beta_{\lambda }=\beta_{•}$. For λ∈R\[0,1] there clearly may hold $q_{y}<0$ for some y∈R. However, there exists a sufficiently large $y_{1}>0$ such that $q_{y}>0$ for all $y>y_{1}$, since $\lim_{y\to \infty }\phi_{\lambda }^{\prime }\left(y\right)=0$ and hence $q_{y}↗\beta_{•}>0$ for y→∞. Thus, let us suppose that $y>y_{1}$. Then, the sequence ${\left(a_{n}^{\left(q_{y}\right)}\right)}_{n\in N}$ is strictly negative, strictly decreasing and converges to $x_{0}^{\left(q_{y}\right)}\in ]-\beta_{•},q_{y}-\beta_{•}[$ (cf. Properties 1 (P1)). If there is some $y\geq y_{1}$ such that h(y)>0 with

$$h\left(y\right)\;:=\;\lim_{n\to \infty }\frac{1}{n}\log\left({\tilde{B}}_{\lambda ,X_{0},n}^{\left(p_{y},q_{y}\right)}\right)\;=\;p_{y}\cdot e^{x_{0}^{\left(q_{y}\right)}}-\alpha_{\lambda }\;,$$

then one can conclude that $\lim_{n\to \infty }H_{\lambda }(P_{A,n}∥P_{H,n})=\infty$. Let us at first consider the case $\alpha_{\lambda }\geq 0$. By employing $p_{y}↘\alpha_{\lambda }$ for y→∞, one gets $p_{y}>0$ for all y≥0. Analogously to the proof of Proposition 9, we now look for a lower bound ${\underline{x}}_{0}^{\left(q_{y}\right)}$ of the fixed point $x_{0}^{\left(q_{y}\right)}$. Notice that $x_{0}^{\left(q_{y}\right)}>-\beta_{•}$ implies

(A14) $${\underline{Q}}_{\lambda }^{\left(q_{y}\right)}\left(x\right)\;:=\;\frac{e^{-\beta_{•}}}{2}\cdot q_{y}\cdot x^{2}\;+\;q_{y}\cdot x\;+\;q_{y}-\beta_{•}\;\leq \;q_{y}\cdot e^{x}\;-\;\beta_{•}\;=\;\xi_{\lambda }^{\left(q_{y}\right)}\left(x\right)\;,$$

since ${\underline{Q}}_{\lambda }^{\left(q_{y}\right)}\left(0\right)=\xi_{\lambda }^{\left(q_{y}\right)}\left(0\right)<0$, ${\underline{Q}}_{\lambda }^{(q_{y})\;\prime }\left(0\right)=\xi_{\lambda }^{(q_{y})\;\prime }\left(0\right)>0$ and $0<{\underline{Q}}_{\lambda }^{(q_{y})\;\prime \prime }\left(x\right)<\xi_{\lambda }^{(q_{y})\;\prime \prime }\left(x\right)$ for $x\in ]-\beta_{•},0]$. Thus, the negative solution ${\underline{x}}_{0}^{\left(q_{y}\right)}$ of the equation ${\underline{Q}}_{\lambda }^{\left(q_{y}\right)}\left(x\right)=x$ (which definitely exists) implies that there holds ${\underline{x}}_{0}^{\left(q_{y}\right)}\leq x_{0}^{\left(q_{y}\right)}$. We easily obtain

(A15) $$\begin{array}{ccc} {\underline{x}}_{0}^{\left(q_{y}\right)} & = & \frac{e^{\beta_{•}}}{q_{y}}\left[\left(1-q_{y}\right)\;-\;\sqrt{{\left(1-q_{y}\right)}^{2}-2e^{-\beta_{•}}q_{y}\left(q_{y}-\beta_{•}\right)}\right]\\ & = & \frac{e^{\beta_{•}}}{\phi_{\lambda }^{\prime }\left(y\right)+\beta_{•}}\left[\left(1-\phi_{\lambda }^{\prime }\left(y\right)-\beta_{•}\right)\;-\;\sqrt{{\left(1-\phi_{\lambda }^{\prime }\left(y\right)-\beta_{•}\right)}^{2}-2\cdot e^{-\beta_{•}}q_{y}\cdot \phi_{\lambda }^{\prime }\left(y\right)}\right]\;<\;0.\;\; \end{array}$$

Since

(A16) $$h\left(y\right)\;=\;p_{y}\cdot e^{x_{0}^{\left(q_{y}\right)}}-\alpha_{\lambda }\;\geq \;p_{y}\cdot e^{{\underline{x}}_{0}^{\left(q_{y}\right)}}-\alpha_{\lambda }\;=:\;\underline{h}\left(y\right)\;,$$

it is sufficient to show $\underline{h}\left(y\right)>0$ for some $y>y_{1}$. We recall from Properties 3 (P15), (P17) and (P19) that

(A17) $$\begin{array}{ccc} \phi_{\lambda }\left(y\right) & = & {\left(\alpha_{A}+\beta_{•}\cdot y\right)}^{\lambda }{\left(\alpha_{H}+\beta_{•}\cdot y\right)}^{1-\lambda }-\lambda \left(\alpha_{A}+\beta_{•}\cdot y\right)-\left(1-\lambda \right)\left(\alpha_{H}+\beta_{•}\cdot y\right)\;>\;0,\\ \phi_{\lambda }^{\prime }\left(y\right) & = & \lambda \cdot \beta_{•}\cdot {\left(\frac{\alpha_{A}+\beta_{•}\cdot y}{\alpha_{H}+\beta_{•}\cdot y}\right)}^{\lambda -1}\;+\;\left(1-\lambda \right)\cdot \beta_{•}\cdot {\left(\frac{\alpha_{A}+\beta_{•}\cdot y}{\alpha_{H}+\beta_{•}\cdot y}\right)}^{\lambda }\;-\;\beta_{•}\;<\;0\;\mathrm{and}\\ \phi_{\lambda }^{\prime \prime }\left(y\right) & = & -\;{\left(\frac{\alpha_{A}+\beta_{•}\cdot y}{\alpha_{H}+\beta_{•}\cdot y}\right)}^{\lambda }\cdot \frac{\lambda \left(1-\lambda \right)\cdot \beta_{•}^{2}\cdot {\left(\alpha_{A}-\alpha_{H}\right)}^{2}}{{\left(\alpha_{A}+\beta_{•}\cdot y\right)}^{2}\left(\alpha_{H}+\beta_{•}\cdot y\right)}\;>\;0, \end{array}$$

which immediately implies $\lim_{y\to \infty }\phi_{\lambda }\left(y\right)=\lim_{y\to \infty }\phi_{\lambda }^{\prime }\left(y\right)=\lim_{y\to \infty }\phi_{\lambda }^{\prime \prime }\left(y\right)=0$, and by means of l’Hospital’s rule

(A18) $$\begin{array}{c} \lim_{y\to \infty }y\cdot \phi_{\lambda }\left(y\right)=\lim_{y\to \infty }-y^{2}\cdot \phi_{\lambda }^{\prime }\left(y\right)\;=\;\lim_{y\to \infty }\frac{y^{3}}{2}\cdot \phi_{\lambda }^{\prime \prime }\left(y\right)\\ =-\frac{1}{2}\lim_{y\to \infty }{\left(\frac{\alpha_{A}+\beta_{•}\cdot y}{\alpha_{H}+\beta_{•}\cdot y}\right)}^{\lambda }\cdot \frac{\lambda \left(1-\lambda \right)\cdot \beta_{•}^{2}\cdot {\left(\alpha_{A}-\alpha_{H}\right)}^{2}}{{\left(\alpha_{A}/y+\beta_{•}\right)}^{2}\left(\alpha_{H}/y+\beta_{•}\right)}\;=\;-\frac{1}{2}\lambda \left(1-\lambda \right)\cdot \frac{{\left(\alpha_{A}-\alpha_{H}\right)}^{2}}{\beta_{•}}\;.\;\;\;\; \end{array}$$

The Formulas (A13), (A15), (A17) imply the limits $\lim_{y\to \infty }p_{y}=\alpha_{\lambda }$, $\lim_{y\to \infty }q_{y}=\beta_{•}$ and $\lim_{y\to \infty }{\underline{x}}_{0}^{\left(q_{y}\right)}=0$ iff $\beta_{•}\leq 1$. The latter is due to the fact that for $\beta_{•}>1$ one gets with (A15) $\lim_{y\to \infty }{\underline{x}}_{0}^{\left(q_{y}\right)}=\frac{e^{\beta_{•}}}{\beta_{•}}\left[\left(1-\beta_{•}\right)-\sqrt{{\left(1-\beta_{•}\right)}^{2}}\right]=\frac{e^{\beta_{•}}}{\beta_{•}}\left[2-2\beta_{•}\right]\neq 0$. In the following, let us assume $\beta_{•}<1$ (the reason why we exclude the case $\beta_{•}=1$ is explained below). One gets $\lim_{y\to \infty }h\left(y\right)\geq \lim_{y\to \infty }\underline{h}\left(y\right)=0$. Since we have to prove that $\underline{h}\left(y\right)>0$ for some $y>y_{1}$, it is sufficient to show that $\lim_{y\to \infty }y\cdot \underline{h}\left(y\right)>0$. To verify the latter, we first derive with l’Hospital’s rule and with (A17), (A18)

(A19) $$\begin{array}{cc} \; & \lim_{y\to \infty }y\cdot \left(1-e^{{\underline{x}}_{0}^{\left(q_{y}\right)}}\right)\;=\;\lim_{y\to \infty }y^{2}\cdot e^{{\underline{x}}_{0}^{\left(q_{y}\right)}}\cdot \left(\frac{\partial }{\partial y}{\underline{x}}_{0}^{\left(q_{y}\right)}\right)\\ \; & =\;\lim_{y\to \infty }\{y^{2}\cdot \frac{-e^{\beta_{•}}\cdot \phi_{\lambda }^{\prime \prime }\left(y\right)}{{\left(\phi_{\lambda }^{\prime }\left(y\right)+\beta_{•}\right)}^{2}}\cdot \left[\left(1-q_{y}\right)\;-\;\sqrt{{\left(1-q_{y}\right)}^{2}-2e^{-\beta_{•}}q_{y}\left(q_{y}-\beta_{•}\right)}\right]\\ \; & \;+\;\frac{e^{\beta_{•}}}{q_{y}}\cdot \left[-y^{2}\cdot \phi_{\lambda }^{\prime \prime }\left(y\right)-\frac{-2y^{2}\phi_{\lambda }^{\prime \prime }\left(y\right)\left(1-q_{y}\right)-2y^{2}\phi_{\lambda }^{\prime \prime }\left(y\right)e^{-\beta_{•}}q_{y}-2y^{2}\phi_{\lambda }^{\prime \prime }\left(y\right)e^{-\beta_{•}}\phi_{\lambda }^{\prime }\left(y\right)}{2\cdot \sqrt{{\left(1-q_{y}\right)}^{2}-2e^{-\beta_{•}}q_{y}\left(q_{y}-\beta_{•}\right)}}\right]\}\\ \; & =\;0\;. \end{array}$$

Notice that without further examination this limit would not necessarily hold for $\beta_{•}=1$, since then the denominator in (A19) converges to zero. With (A13), (A16), (A18) and (A19) we finally obtain

(A20) $$\begin{array}{ccc} \lim_{y\to \infty }y\cdot \underline{h}\left(y\right) & = & \lim_{y\to \infty }\left\{\left(y\cdot \phi_{\lambda }\left(y\right)-y^{2}\cdot \phi_{\lambda }^{\prime }\left(y\right)\right)\cdot e^{{\underline{x}}_{0}^{\left(q_{y}\right)}}-y\cdot \left(1-e^{{\underline{x}}_{0}^{\left(q_{y}\right)}}\right)\alpha_{\lambda }\right\}\\ & = & -\lambda \left(1-\lambda \right)\frac{{\left(\alpha_{A}-\alpha_{H}\right)}^{2}}{\beta_{•}}\;>\;0\;. \end{array}$$

Let us now consider the case $\alpha_{\lambda }<0$. The proof works out almost completely analogous to the case $\alpha_{\lambda }\geq 0$. We indicate the main differences. Since $p_{y}↘\alpha_{\lambda }<0$ and $q_{y}↗\beta_{•}\in ]0,1[$ for y→∞, there is a sufficiently large $y_{2}>y_{1}$, such that $p_{y}<0$ and $q_{y}>0$. Thus,

$${\bar{Q}}_{\lambda }^{\left(q_{y}\right)}\left(x\right)\;:=\;\frac{q_{y}}{2}\cdot x^{2}\;+\;q_{y}\cdot x\;+\;q_{y}-\beta_{•}\;\geq \;\xi_{\lambda }^{\left(q_{y}\right)}\left(x\right)\;=\;q_{y}e^{x}-\beta_{•}\;\mathrm{for}\;x\in ]-\infty ,0].$$

The corresponding (existing) smaller solution of ${\bar{Q}}_{\lambda }^{\left(q_{y}\right)}\left(x\right)=x$ is

$${\bar{x}}_{0}^{\left(q_{y}\right)}\;=\;\frac{1}{q_{y}}\left[\left(1-q_{y}\right)\;-\;\sqrt{{\left(1-q_{y}\right)}^{2}-2q_{y}\left(q_{y}-\beta_{•}\right)}\right]\;,$$

having the same form as the solution (A15) with $e^{-\beta_{•}}$ substituted by 1. Notice that there clearly holds $x_{0}^{\left(q_{y}\right)}<{\bar{x}}_{0}^{\left(q_{y}\right)}<0$. However, since $p_{y}<0$, we now get $h\left(y\right)=p_{y}\cdot e^{x_{0}^{\left(q_{y}\right)}}-\alpha_{\lambda }\geq p_{y}\cdot e^{{\bar{x}}_{0}^{\left(q_{y}\right)}}-\alpha_{\lambda }=:\underline{h}\left(y\right)$, as in (A16). Since all calculations (A17) to (A20) remain valid (with $e^{-\beta_{•}}$ substituted by 1), this proof is finished. □

### Appendix A.2. Proofs and Auxiliary Lemmas for Section 5

We start with two lemmas which will be useful for the proof of Theorem 3. They deal with the sequence ${\left(a_{n}^{\left(q_{\lambda }\right)}\right)}_{n\in N}$ from (36).

**Lemma** **A2.**
*For arbitrarily fixed parameter constellation $\left(\beta_{A},\beta_{H},\alpha_{A},\alpha_{H},\lambda \right)\in P\times ]0,1[$, suppose that $q_{\lambda }>0$ and $\lim_{\lambda ↗1}\;q_{\lambda }\;=\;\beta_{A}$ holds. Then one gets the limit*

(A21) $$\forall \;n\in N:\;\lim_{\lambda ↗1}\;a_{n}^{\left(q_{\lambda }\right)}\;=0.$$

**Proof.** This can be easily seen by induction: for n=1 there clearly holds

$$\lim_{\lambda ↗1}a_{1}^{\left(q_{\lambda }\right)}\;=\;\lim_{\lambda ↗1}\left(q_{\lambda }-\beta_{\lambda }\right)\;=\;\beta_{A}-\beta_{A}\;=\;0\;.$$

Assume now that $\lim_{\lambda ↗1}\;a_{k}^{\left(q_{\lambda }\right)}\;=0$ holds for all k∈N, k≤n−1, then

$$\;\lim_{\lambda ↗1}a_{n}^{\left(q_{\lambda }\right)}\;=\;\lim_{\lambda ↗1}\left(q_{\lambda }\cdot e^{a_{n-1}^{\left(q_{\lambda }\right)}}-\beta_{\lambda }\right)\;=\;\beta_{A}\cdot 1-\beta_{A}\;=\;0\;.\;□$$

**Lemma** **A3.**
*In addition to the assumptions of Lemma A2, suppose that $\lambda \mapsto q_{\lambda }$ is continuously differentiable on ]0,1[ and that the limit $l:=\lim_{\lambda ↗1}\frac{\partial \;q_{\lambda }}{\partial \lambda }$ is finite. Then, for all n∈N one obtains*

(A22) $$\lim_{\lambda ↗1}\frac{\partial \;a_{n}^{\left(q_{\lambda }\right)}}{\partial \lambda }\;=\;u_{n}\;:=\;\left\{\begin{array}{cc} \frac{l+\beta_{H}-\beta_{A}}{1-\beta_{A}}\cdot \left(1-{\left(\beta_{A}\right)}^{n}\right)\;, & \mathrm{if}\;\beta_{A}\neq 1,\\ \; & \;\\ n\cdot \left(l+\beta_{H}-1\right)\;, & \mathrm{if}\;\beta_{A}=1, \end{array}\right.$$

*which is the unique solution of the linear recursion equation*

(A23) $$u_{n}\;=\;l+\beta_{H}-\beta_{A}\;+\;\beta_{A}\cdot u_{n-1}\;,\;u_{0}\;=\;0\;.$$

*Furthermore, for all n∈N there holds*

$$\sum_{k=1}^{n}\lim_{\lambda ↗1}\frac{\partial \;a_{k}^{\left(q_{\lambda }\right)}}{\partial \lambda }\;=\;\sum_{k=1}^{n}u_{k}\;=\;\left\{\begin{array}{cc} \frac{l+\beta_{H}-\beta_{A}}{1-\beta_{A}}\cdot \left[n-\frac{\beta_{A}}{1-\beta_{A}}\left(1-{\left(\beta_{A}\right)}^{n}\right)\right]\;, & \mathrm{if}\;\beta_{A}\neq 1,\\ \; & \;\\ \frac{n\cdot (n+1)}{2}\cdot \left(l+\beta_{H}-1\right)\;, & \mathrm{if}\;\beta_{A}=1. \end{array}\right.$$

**Proof.** Clearly, $u_{n}$ defined by (A22) is the unique solution of (A23). We prove by induction that $\lim_{\lambda ↗1}\frac{\partial \;a_{n}^{\left(q_{\lambda }\right)}}{\partial \lambda }\;=\;u_{n}$ holds. For n=1 one gets

$$\lim_{\lambda ↗1}\frac{\partial \;a_{1}^{\left(q_{\lambda }\right)}}{\partial \lambda }\;=\;\lim_{\lambda ↗1}\frac{\partial \;(q_{\lambda }-\beta_{\lambda })}{\partial \lambda }\;=\;l-\left(\beta_{A}-\beta_{H}\right)\;=\;u_{1}.$$

Suppose now that (A22) holds for all k∈N, k≤n−1. Then, by incorporating (A21) we obtain

$$\begin{array}{ccc} \;\lim_{\lambda ↗1}\frac{\partial \;a_{n}^{\left(q_{\lambda }\right)}}{\partial \lambda } & = & \lim_{\lambda ↗1}\frac{\partial }{\partial \lambda }\left(q_{\lambda }\cdot e^{a_{n-1}^{\left(q_{\lambda }\right)}}-\beta_{\lambda }\right)\;=\;\lim_{\lambda ↗1}e^{a_{n-1}^{\left(q_{\lambda }\right)}}\cdot \left(\frac{\partial \;q_{\lambda }}{\partial \lambda }+q_{\lambda }\frac{\partial \;a_{n-1}^{\left(q_{\lambda }\right)}}{\partial \lambda }\right)-\left(\beta_{A}-\beta_{H}\right)\\ & = & l-\left(\beta_{A}-\beta_{H}\right)+\beta_{A}\cdot u_{n-1}\;=\;u_{n}. \end{array}$$

The remaining assertions follow immediately. □

We are now ready to give the

**Proof of Theorem** **3.** (a) Recall that for the setup $\left(\beta_{A},\beta_{H},\alpha_{A},\alpha_{H}\right)\in \left(P_{\mathrm{NI}}\cup P_{\mathrm{SP},1}\right)$ we chose the intercept as $p_{\lambda }:=p_{\lambda }^{E}:=\alpha_{A}^{\lambda }\alpha_{H}^{1-\lambda }$ and the slope as $q_{\lambda }:=q_{\lambda }^{E}:=\beta_{A}^{\lambda }\beta_{H}^{1-\lambda }$, which in (39) lead to the exact value $V_{\lambda ,X_{0},n}$ of the Hellinger integral. Because of $\frac{p_{\lambda }}{q_{\lambda }}\beta_{\lambda }-\alpha_{\lambda }=0$ as well as $\lim_{\lambda ↗1}q_{\lambda }=\beta_{A}$, we obtain by using (38) and Lemma A2 for all $X_{0}\in N$ and for all n∈N

$$\lim_{\lambda ↗1}V_{\lambda ,X_{0},n}\;:=\;\lim_{\lambda ↗1}\exp\left\{a_{n}^{\left(q_{\lambda }\right)}\cdot X_{0}+\sum_{k=1}^{n}b_{k}^{\left(p_{\lambda },q_{\lambda }\right)}\right\}\;=\;\lim_{\lambda ↗1}\exp\left\{a_{n}^{\left(q_{\lambda }\right)}\cdot X_{0}+\frac{\alpha_{A}}{\beta_{A}}\sum_{k=1}^{n}a_{k}^{\left(q_{\lambda }\right)}\right\}\;=\;1,$$

which leads by (68) to

(A24) $$\begin{array}{ccc} I(P_{A,n}∥P_{H,n}) & = & \lim_{\lambda ↗1}\;\frac{1-H_{\lambda }(P_{A,n}∥P_{H,n})}{\lambda \cdot (1-\lambda )}\;=\;\lim_{\lambda ↗1}\;\frac{1-V_{\lambda ,X_{0},n}}{\lambda \cdot (1-\lambda )}\\ & = & \lim_{\lambda ↗1}\frac{-V_{\lambda ,X_{0},n}}{1-2\lambda }\cdot \frac{\partial }{\partial \lambda }\left[a_{n}^{\left(q_{\lambda }\right)}\cdot X_{0}+\frac{p_{\lambda }}{q_{\lambda }}\sum_{k=1}^{n}a_{k}^{\left(q_{\lambda }\right)}\right]\\ & = & \lim_{\lambda ↗1}\left[\frac{\partial \;a_{n}^{\left(q_{\lambda }\right)}}{\partial \lambda }\cdot X_{0}+\left(\frac{\partial }{\partial \lambda }\frac{p_{\lambda }}{q_{\lambda }}\right)\cdot \sum_{k=1}^{n}a_{k}^{\left(q_{\lambda }\right)}+\frac{p_{\lambda }}{q_{\lambda }}\cdot \sum_{k=1}^{n}\frac{\partial \;a_{k}^{\left(q_{\lambda }\right)}}{\partial \lambda }\right]. \end{array}$$

For further analysis, we use the obvious derivatives

(A25) $$\frac{\partial \;p_{\lambda }}{\partial \lambda }\;=\;p_{\lambda }\;\log\left(\frac{\alpha_{A}}{\alpha_{H}}\right),\;\frac{\partial }{\partial \lambda }\frac{p_{\lambda }}{q_{\lambda }}\;=\;\frac{p_{\lambda }}{q_{\lambda }}\;\log\left(\frac{\alpha_{A}\beta_{H}}{\alpha_{H}\beta_{A}}\right),\;\frac{\partial \;q_{\lambda }}{\partial \lambda }\;=\;q_{\lambda }\;\log\left(\frac{\beta_{A}}{\beta_{H}}\right),$$

where the subcase $\left(\beta_{A},\beta_{H},\alpha_{A},\alpha_{H}\right)\in P_{\mathrm{NI}}$ (with $p_{\lambda }\equiv 0$) is consistently covered. From (A25) and Lemma A3 we deduce

$$\lim_{\lambda ↗1}\frac{\partial \;a_{n}^{\left(q_{\lambda }\right)}}{\partial \lambda }\cdot X_{0}\;=\;\left\{\begin{array}{cc} \left(\beta_{A}\log\left(\frac{\beta_{A}}{\beta_{H}}\right)-\left(\beta_{A}-\beta_{H}\right)\right)\cdot \frac{1-{\left(\beta_{A}\right)}^{n}}{1-\beta_{A}}\cdot X_{0}, & \mathrm{if}\beta_{A}\neq 1,\\ n\cdot \left(\beta_{A}\log\left(\frac{\beta_{A}}{\beta_{H}}\right)-\left(\beta_{A}-\beta_{H}\right)\right)\cdot X_{0}, & \mathrm{if}\beta_{A}=1, \end{array}\right.$$

and by means of (A21)

$$\forall \;n\in N:\;\lim_{\lambda ↗1}\left[\left(\frac{\partial }{\partial \lambda }\frac{p_{\lambda }}{q_{\lambda }}\right)\cdot \sum_{k=1}^{n}a_{k}^{\left(q_{\lambda }\right)}\right]\;=\;0.$$

For the last expression in (A24) we again apply Lemma A3 to end up with

(A26) $$\lim_{\lambda ↗1}\frac{p_{\lambda }}{q_{\lambda }}\cdot \sum_{k=1}^{n}\frac{\partial }{\partial \lambda }a_{k}^{\left(q_{\lambda }\right)}\;=\;\left\{\begin{array}{cc} \frac{\alpha_{A}\cdot \left[\beta_{A}\log\left(\frac{\beta_{A}}{\beta_{H}}\right)-\left(\beta_{A}-\beta_{H}\right)\right]}{\beta_{A}\left(1-\beta_{A}\right)}\cdot \left[n-\frac{\beta_{A}}{1-\beta_{A}}\left(1-{\left(\beta_{A}\right)}^{n}\right)\right], & \mathrm{if}\beta_{A}\neq 1,\\ n\cdot \left(n+1\right)\frac{\alpha_{A}}{2\beta_{A}}\cdot \left[\beta_{A}\log\left(\frac{\beta_{A}}{\beta_{H}}\right)-\left(\beta_{A}-\beta_{H}\right)\right], & \mathrm{if}\beta_{A}=1, \end{array}\right.$$

which finishes the proof of part (a). To show part (b), for the corresponding setup $\left(\beta_{A},\beta_{H},\alpha_{A},\alpha_{H}\right)$$\in P_{\mathrm{SP}}\backslash P_{\mathrm{SP},1}$ let us first choose – according to (45) in Section 3.4—the intercept as $p_{\lambda }:=p_{\lambda }^{L}:=\alpha_{A}^{\lambda }\alpha_{H}^{1-\lambda }$ and the slope as $q_{\lambda }:=q_{\lambda }^{L}:=\beta_{A}^{\lambda }\beta_{H}^{1-\lambda }$, which in part (b) of Proposition 6 lead to the lower bounds $B_{\lambda ,X_{0},n}^{L}$ of the Hellinger integral. This is formally the same choice as in part (a) satisfying $\lim_{\lambda ↗1}p_{\lambda }=\alpha_{A}$, $\lim_{\lambda ↗1}q_{\lambda }=\beta_{A}$ but in contrast to (a) we now have $\frac{p_{\lambda }}{q_{\lambda }}\;\beta_{\lambda }-\alpha_{\lambda }\neq 0$ but nevertheless

$$\lim_{\lambda ↗1}\frac{p_{\lambda }}{q_{\lambda }}\;\beta_{\lambda }-\alpha_{\lambda }\;=\;0.$$

From this, (38), part (b) of Proposition 6 and Lemma A2 we obtain

(A27) $$\lim_{\lambda ↗1}B_{\lambda ,X_{0},n}^{L}\;=\;\lim_{\lambda ↗1}\exp\left\{a_{n}^{\left(q_{\lambda }\right)}\cdot X_{0}+\frac{p_{\lambda }}{q_{\lambda }}\;\sum_{k=1}^{n}a_{k}^{\left(q_{\lambda }\right)}+n\cdot \left(\frac{p_{\lambda }}{q_{\lambda }}\beta_{\lambda }-\alpha_{\lambda }\right)\right\}\;=\;1$$

and hence

(A28) $$\begin{array}{ccc} \;I(P_{A,n}∥P_{H,n}) & \leq & \;\lim_{\lambda ↗1}\frac{1-B_{\lambda ,X_{0},n}^{L}}{\lambda \cdot (1-\lambda )}=\lim_{\lambda ↗1}\frac{-B_{\lambda ,X_{0},n}^{L}}{1-2\lambda }\cdot \frac{\partial }{\partial \lambda }\left[a_{n}^{\left(q_{\lambda }\right)}X_{0}+\frac{p_{\lambda }}{q_{\lambda }}\sum_{k=1}^{n}a_{k}^{\left(q_{\lambda }\right)}+n\left(\frac{p_{\lambda }}{q_{\lambda }}\;\beta_{\lambda }-\alpha_{\lambda }\right)\right]\\ \; & = & \;\lim_{\lambda ↗1}\left[\frac{\partial \;a_{n}^{\left(q_{\lambda }\right)}}{\partial \lambda }X_{0}+\left(\frac{\partial }{\partial \lambda }\frac{p_{\lambda }}{q_{\lambda }}\right)\sum_{k=1}^{n}a_{k}^{\left(q_{\lambda }\right)}+\frac{p_{\lambda }}{q_{\lambda }}\sum_{k=1}^{n}\frac{\partial \;a_{k}^{\left(q_{\lambda }\right)}}{\partial \lambda }+n\frac{\partial }{\partial \lambda }\left(\frac{p_{\lambda }}{q_{\lambda }}\beta_{\lambda }-\alpha_{\lambda }\right)\right]\;. \end{array}$$

In the current setup, the first three expressions in (A28) can be evaluated in exactly the same way as in (A25) to (A26), and for the last expression one has the limit

$$\begin{array}{ccc} \frac{\partial }{\partial \lambda }\left(\frac{p_{\lambda }}{q_{\lambda }}\;\beta_{\lambda }-\alpha_{\lambda }\right) & = & \frac{p_{\lambda }}{q_{\lambda }}\;\log\left(\frac{\alpha_{A}\beta_{H}}{\alpha_{H}\beta_{A}}\right)\cdot \beta_{\lambda }\;+\;\frac{p_{\lambda }}{q_{\lambda }}\cdot \left(\beta_{A}-\beta_{H}\right)\;-\;\left(\alpha_{A}-\alpha_{H}\right)\\ & \overset{\lambda ↗1}{⟶} & \alpha_{A}\left[\log\left(\frac{\alpha_{A}\beta_{H}}{\alpha_{H}\beta_{A}}\right)-\frac{\beta_{H}}{\beta_{A}}\right]+\alpha_{H}\;, \end{array}$$

which finishes the proof of part (b). □

**Proof of Theorem** **4.** Let us fix $\left(\beta_{A},\beta_{H},\alpha_{A},\alpha_{H}\right)\in P_{\mathrm{SP}}\backslash P_{\mathrm{SP},1}$, $X_{0}\in N$, n∈N and y∈[0,∞[. The lower bound $E_{y,X_{0},n}^{L,tan}$ of the Kullback-Leibler information divergence (relative entropy) is derived by using $\phi_{\lambda }^{U}\equiv \phi_{\lambda ,y}^{\tan}$ (cf. (52)), which corresponds to the tangent line of $\phi_{\lambda }$ at *y*, as a linear upper bound for $\phi_{\lambda }$ (λ∈]0,1[). More precisely, one gets $\phi_{\lambda }^{U}\left(x\right):=\left(p_{\lambda }^{U}-\alpha_{\lambda }\right)+\left(q_{\lambda }^{U}-\beta_{\lambda }\right)\;x$ (x∈[0,∞[) with $p_{\lambda }:=p_{\lambda }\left(y\right):=\phi_{\lambda }\left(y\right)-y\phi_{\lambda }^{\prime }\left(y\right)+\alpha_{\lambda }$ and $q_{\lambda }:=q_{\lambda }\left(y\right):=\phi_{\lambda }^{\prime }\left(y\right)+\beta_{\lambda }$, implying $q_{\lambda }>0$ because of Properties 3 (P17). Analogously to (A27) and (A28), we obtain from (38) and (40) the convergence $\lim_{\lambda ↗1}B_{\lambda ,X_{0},n}^{U}=1$ and thus

(A29) $$I(P_{A,n}∥P_{H,n})\;\geq \;\lim_{\lambda ↗1}\left[\frac{\partial \;a_{n}^{\left(q_{\lambda }\right)}}{\partial \lambda }X_{0}+\left(\frac{\partial }{\partial \lambda }\frac{p_{\lambda }}{q_{\lambda }}\right)\sum_{k=1}^{n}a_{k}^{\left(q_{\lambda }\right)}+\frac{p_{\lambda }}{q_{\lambda }}\sum_{k=1}^{n}\frac{\partial \;a_{k}^{\left(q_{\lambda }\right)}}{\partial \lambda }+n\frac{\partial }{\partial \lambda }\left(\frac{p_{\lambda }}{q_{\lambda }}\beta_{\lambda }-\alpha_{\lambda }\right)\right].$$

As before, we compute the involved derivatives. From (30) to (32) as well as (P17) we get

(A30) $$\begin{array}{ccc} \;\frac{\partial p_{\lambda }}{\partial \lambda } & \;= & \;{\left(\frac{f_{A}\left(y\right)}{f_{H}\left(y\right)}\right)}^{\lambda }f_{H}\left(y\right)\log\left(\frac{f_{A}\left(y\right)}{f_{H}\left(y\right)}\right)-\beta_{A}y{\left(\frac{f_{A}\left(y\right)}{f_{H}\left(y\right)}\right)}^{\lambda -1}\;-\lambda \beta_{A}y{\left(\frac{f_{A}\left(y\right)}{f_{H}\left(y\right)}\right)}^{\lambda -1}\;\log\left(\frac{f_{A}\left(y\right)}{f_{H}\left(y\right)}\right)\\ & & \;+\;\beta_{H}y{\left(\frac{f_{A}\left(y\right)}{f_{H}\left(y\right)}\right)}^{\lambda }-\left(1-\lambda \right)\beta_{H}y{\left(\frac{f_{A}\left(y\right)}{f_{H}\left(y\right)}\right)}^{\lambda }\log\left(\frac{f_{A}\left(y\right)}{f_{H}\left(y\right)}\right)\\ & \;\overset{\lambda ↗1}{⟶} & \;\alpha_{A}\log\left(\frac{f_{A}\left(y\right)}{f_{H}\left(y\right)}\right)+\frac{y\cdot (\alpha_{A}\beta_{H}-\alpha_{H}\beta_{A})}{f_{H}\left(y\right)}\;, \end{array}$$

and

(A31) $$\begin{array}{ccc} \frac{\partial q_{\lambda }}{\partial \lambda } & = & \beta_{A}{\left(\frac{f_{A}\left(y\right)}{f_{H}\left(y\right)}\right)}^{\lambda -1}+\lambda \beta_{A}{\left(\frac{f_{A}\left(y\right)}{f_{H}\left(y\right)}\right)}^{\lambda -1}\log\left(\frac{f_{A}\left(y\right)}{f_{H}\left(y\right)}\right)-\beta_{H}{\left(\frac{f_{A}\left(y\right)}{f_{H}\left(y\right)}\right)}^{\lambda }\\ & & +\;\left(1-\lambda \right)\beta_{H}{\left(\frac{f_{A}\left(y\right)}{f_{H}\left(y\right)}\right)}^{\lambda }\log\left(\frac{f_{A}\left(y\right)}{f_{H}\left(y\right)}\right)\\ & \overset{\lambda ↗1}{⟶} & \beta_{A}\left(1+\log\left(\frac{f_{A}\left(y\right)}{f_{H}\left(y\right)}\right)\right)-\beta_{H}\frac{f_{A}\left(y\right)}{f_{H}\left(y\right)}\;=:\;l. \end{array}$$

Combining these two limits we get

(A32) $$\begin{array}{ccc} \frac{\partial }{\partial \lambda }\left(\frac{p_{\lambda }}{q_{\lambda }}\beta_{\lambda }-\alpha_{\lambda }\right) & = & \frac{q_{\lambda }\left(\frac{\partial p_{\lambda }}{\partial \lambda }\right)-p_{\lambda }\left(\frac{\partial q_{\lambda }}{\partial \lambda }\right)}{{\left(q_{\lambda }\right)}^{2}}\cdot \beta_{\lambda }+\frac{p_{\lambda }}{q_{\lambda }}\cdot \left(\beta_{A}-\beta_{H}\right)-\left(\alpha_{A}-\alpha_{H}\right)\\ & \overset{\lambda ↗1}{⟶} & \left[\frac{y\cdot (\alpha_{A}\beta_{H}-\alpha_{H}\beta_{A})}{f_{H}\left(y\right)}-\alpha_{A}\left(1-\frac{\beta_{H}f_{A}\left(y\right)}{\beta_{A}f_{H}\left(y\right)}\right)\right]+\alpha_{H}-\frac{\alpha_{A}\beta_{H}}{\beta_{A}}.\\ & = & \left(\alpha_{H}-\alpha_{A}\frac{\beta_{H}}{\beta_{A}}\right)\left(1-\frac{f_{A}\left(y\right)}{f_{H}\left(y\right)}\right). \end{array}$$

The above calculation also implies that $\lim_{\lambda ↗1}\left(\frac{\partial }{\partial \lambda }\frac{p_{\lambda }}{q_{\lambda }}\right)$ is finite and thus $\lim_{\lambda ↗1}\left(\frac{\partial }{\partial \lambda }\frac{p_{\lambda }}{q_{\lambda }}\right)\sum_{k=1}^{n}a_{k}^{\left(q_{\lambda }\right)}=0$ by means of Lemma A2. The proof of $I(P_{A,n}∥P_{H,n})\geq E_{y,X_{0},n}^{L,\tan}$ is finished by using Lemma A3 with *l* defined in (A31) and by plugging the limits (A30) to (A32) in (A29). To derive the lower bound $E_{k,X_{0},n}^{L,\sec}$ (cf. (73)) for fixed $k\in N_{0}$, we use as a linear upper bound $\phi_{\lambda }^{U}$ for $\phi_{\lambda }\left(\cdot \right)$ (λ∈]0,1[) the secant line $\phi_{\lambda ,k}^{\sec}$ (cf. (53)) of $\phi_{\lambda }$ across its arguments *k* and k+1, corresponding to the choices $p_{\lambda }:=p_{\lambda ,k}^{\sec}=\left(k+1\right)\cdot \phi_{\lambda }\left(k\right)-k\cdot \phi_{\lambda }\left(k+1\right)+\alpha_{\lambda }$ and $q_{\lambda }:=q_{\lambda ,k}^{\sec}:=\phi_{\lambda }\left(k+1\right)-\phi_{\lambda }\left(k\right)+\beta_{\lambda }$, implying $q_{\lambda }>0$ because of Properties 3 (P18). As a side remark, notice that this $\phi_{\lambda }^{U}\left(x\right)$ may become positive for some x∈[0,∞[ (which is not always consistent with Goal (G1) for fixed λ, but leads to a tractable limit bound as λ tends to 1). Analogously to (A27) and (A28) we get again $\lim_{\lambda ↗1}B_{\lambda ,X_{0},n}^{U}=1$, which leads to the lower bound given in (A29) with appropriately plugged-in quantities. As in the above proof of the lower bound $E_{y,X_{0},n}^{L,tan}$, the inequality $I(P_{A,n}∥P_{H,n})\geq E_{k,X_{0},n}^{L,\sec}$ follows straightforwardly from Lemma A2, Lemma A3 and the three limits

$$\begin{array}{c} \;\frac{\partial p_{\lambda }}{\partial \lambda }\;=\;{\left(\frac{f_{A}\left(k\right)}{f_{H}\left(k\right)}\right)}^{\lambda }f_{H}\left(k\right)\cdot \left(k\;+\;1\right)\log\left(\frac{f_{A}\left(k\right)}{f_{H}\left(k\right)}\right)-{\left(\frac{f_{A}\left(k\;+\;1\right)}{f_{H}\left(k\;+\;1\right)}\right)}^{\lambda }f_{H}\left(k\;+\;1\right)\cdot k\log\left(\frac{f_{A}\left(k\;+\;1\right)}{f_{H}\left(k\;+\;1\right)}\right)\\ \;\overset{\lambda ↗1}{⟶}f_{A}\left(k\right)\left(k\;+\;1\right)\log\left(\frac{f_{A}\left(k\right)}{f_{H}\left(k\right)}\right)-f_{A}\left(k\;+\;1\right)k\log\left(\frac{f_{A}\left(k\;+\;1\right)}{f_{H}\left(k\;+\;1\right)}\right),\\ \;\frac{\partial q_{\lambda }}{\partial \lambda }\;=\;{\left(\frac{f_{A}\left(k\;+\;1\right)}{f_{H}\left(k\;+\;1\right)}\right)}^{\lambda }f_{H}\left(k\;+\;1\right)\log\left(\frac{f_{A}\left(k\;+\;1\right)}{f_{H}\left(k\;+\;1\right)}\right)-{\left(\frac{f_{A}\left(k\right)}{f_{H}\left(k\right)}\right)}^{\lambda }f_{H}\left(k\right)\log\left(\frac{f_{A}\left(k\right)}{f_{H}\left(k\right)}\right)\\ \;\overset{\lambda ↗1}{⟶}f_{A}\left(k\;+\;1\right)\log\left(\frac{f_{A}\left(k\;+\;1\right)}{f_{H}\left(k\;+\;1\right)}\right)-f_{A}\left(k\right)\log\left(\frac{f_{A}\left(k\right)}{f_{H}\left(k\right)}\right)\;=:\;l\;,\;\;\mathrm{and}\\ \;\frac{\partial }{\partial \lambda }\left(\frac{p_{\lambda }}{q_{\lambda }}\beta_{\lambda }-\alpha_{\lambda }\right)\;=\;\frac{q_{\lambda }\left(\frac{\partial p_{\lambda }}{\partial \lambda }\right)-p_{\lambda }\left(\frac{\partial q_{\lambda }}{\partial \lambda }\right)}{{\left(q_{\lambda }\right)}^{2}}\cdot \beta_{\lambda }+\frac{p_{\lambda }}{q_{\lambda }}\cdot \left(\beta_{A}-\beta_{H}\right)-\left(\alpha_{A}-\alpha_{H}\right)\\ \;\overset{\lambda ↗1}{⟶}f_{A}\left(k\right)\log\left(\frac{f_{A}\left(k\right)}{f_{H}\left(k\right)}\right)\left(k\;+\;1+\frac{\alpha_{A}}{\beta_{A}}\right)-f_{A}\left(k\;+\;1\right)\log\left(\frac{f_{A}\left(k\;+\;1\right)}{f_{H}\left(k\;+\;1\right)}\right)\left(k+\frac{\alpha_{A}}{\beta_{A}}\right)-\frac{\alpha_{A}\beta_{H}}{\beta_{A}}+\alpha_{H}. \end{array}$$

To construct the third lower bound $E_{X_{0},n}^{L,hor}$ (cf. (74)), we start by using the horizontal line $\phi_{\lambda }^{\mathrm{hor}}\left(\cdot \right)$ (cf. (54)) as an upper bound of $\phi_{\lambda }$. For each fixed λ∈]0,1[, it is defined by the intercept $\sup_{x\in N_{0}}\phi_{\lambda }\left(x\right)$. On $P_{\mathrm{SP},3a}\cup P_{\mathrm{SP},3b}$, this supremum is achieved at the finite integer point $z_{\lambda }^{*}:=\arg\max_{x\in N_{0}}\phi_{\lambda }\left(x\right)$ (since $\lim_{x\to \infty }\phi_{\lambda }\left(x\right)=-\infty$) and there holds $\phi_{\lambda }\left(z_{\lambda }^{*}\right)<0$ which leads with the parameters $q_{\lambda }=\beta_{\lambda }$, $p_{\lambda }=\phi_{\lambda }\left(z_{\lambda }^{*}\right)+\alpha_{\lambda }$ to the Hellinger integral upper bound $B_{\lambda ,X_{0},n}^{U}=\exp\left\{\phi_{\lambda }\left(z_{\lambda }^{*}\right)\cdot n\right\}<1$ (cf. Remark 1 (b)). We strive for computing the limit $\lim_{\lambda ↗1}\frac{1-B_{\lambda ,X_{0},n}^{U}}{\lambda (1-\lambda )}$, which is not straightforward to solve since in general it seems to be intractable to express $z_{\lambda }^{*}$ explicitly in terms of λ. To circumvent this problem, we notice that it is sufficient to determine $z_{\lambda }^{*}$ in a small ϵ–environment ]1−ϵ,1[. To accomplish this, we incorporate $\lim_{\lambda ↗1}\phi_{\lambda }\left(x\right)=0$ for all x∈[0,∞[ and calculate by using l’Hospital’s rule

$$\lim_{\lambda ↗1}\frac{\phi_{\lambda }\left(x\right)}{1-\lambda }\;=\;\left(\alpha_{A}+\beta_{A}x\right)\left[-\log\left(\frac{\alpha_{A}+\beta_{A}x}{\alpha_{H}+\beta_{H}x}\right)+1\right]-\left(\alpha_{H}+\beta_{H}x\right).$$

Accordingly, let us define $z^{*}:=\arg\max_{x\in N_{0}}\left\{\left(\alpha_{A}+\beta_{A}x\right)\left[-\log\left(\frac{\alpha_{A}+\beta_{A}x}{\alpha_{H}+\beta_{H}x}\right)+1\right]-\left(\alpha_{H}+\beta_{H}x\right)\right\}$ (note that the maximum exists since $\lim_{x\to \infty }\left\{\left(\alpha_{A}+\beta_{A}x\right)\left[-\log\left(\frac{\alpha_{A}+\beta_{A}x}{\alpha_{H}+\beta_{H}x}\right)+1\right]-\left(\alpha_{H}+\beta_{H}x\right)\right\}$=−∞). Due to continuity of the function $\left(\lambda ,x\right)\mapsto \frac{\phi_{\lambda }\left(x\right)}{1-\lambda }$, there exists an ϵ>0 such that for all λ∈]1−ϵ,1[ there holds $z_{\lambda }^{*}=z^{*}$. Applying these considerations, we get with l’Hospital’s rule

(A33) $$I(P_{A,n}∥P_{H,n})\;\geq \;\lim_{\lambda ↗1}\frac{1-\exp\left\{\phi_{\lambda }\left(z^{*}\right)\cdot n\right\}}{\lambda (1-\lambda )}\;=\;\left[f_{A}\left(z^{*}\right)\cdot \left[\log\left(\frac{f_{A}\left(z^{*}\right)}{f_{H}\left(z^{*}\right)}\right)-1\right]+f_{H}\left(z^{*}\right)\right]\cdot \;n\;\geq \;0.$$

In fact, for the current parameter constellation $P_{\mathrm{SP},3a}\cup P_{\mathrm{SP},3b}$ we have $\phi_{\lambda }\left(x\right)<0$ for all λ∈]0,1[ and all $x\in N_{0}$ which implies $f_{A}\left(z^{*}\right)\neq f_{H}\left(z^{*}\right)$ by Lemma A1; thus, we even get $E_{X_{0},n}^{L,hor}>0$ for all n∈N by virtue of the inequality $-\log\left(\frac{f_{H}\left(z^{*}\right)}{f_{A}\left(z^{*}\right)}\right)>-\frac{f_{H}\left(z^{*}\right)}{f_{A}\left(z^{*}\right)}+1$. For the case $P_{\mathrm{SP},2}$, the above-mentioned procedure leads to $z_{\lambda }^{*}=0=z^{*}$ (λ∈]0,1[) which implies $\phi_{\lambda }\left(z_{\lambda }^{*}\right)=0$, $B_{\lambda ,X_{0},n}^{U}\equiv 1$ and thus the trivial lower bound $E_{X_{0},n}^{L,hor}=\lim_{\lambda ↗1}\frac{1-B_{\lambda ,X_{0},n}^{U}}{\lambda (1-\lambda )}=0$ follows for all n∈N. In contrast, for the case $P_{\mathrm{SP},3c}$ one gets $z_{\lambda }^{*}=\frac{\alpha_{A}-\alpha_{H}}{\beta_{H}-\beta_{A}}=z^{*}$ (λ∈]0,1[) which nevertheless also implies $\phi_{\lambda }\left(z_{\lambda }^{*}\right)=0$ and hence $E_{X_{0},n}^{L,hor}\equiv 0$. On $P_{\mathrm{SP},4}$, we have $\sup_{x\in N_{0}}\phi_{\lambda }\left(x\right)=\lim_{x\to \infty }\phi_{\lambda }\left(x\right)=0$ and hence we set $E_{X_{0},n}^{L,hor}\equiv 0$. To show the strict positivity $E_{X_{0},n}^{L}>0$ in the parameter case $P_{\mathrm{SP},2}$, we inspect the bound $E_{0,X_{0},n}^{L,sec}$. With $\alpha :=\alpha_{•}:=\alpha_{A}=\alpha_{H}$ (the bullet will be omitted in this proof) and the auxiliary variable $x:=\frac{\beta_{H}}{\beta_{A}}>0$, the definition (73) respectively its special case (76) rewrites for all n∈N as

(A34) $$E_{0,X_{0},n}^{L,sec}\;:=\;E_{0,X_{0},n}^{L,sec}\left(x\right)\;:=\;\left\{\begin{array}{cc} \left[-\left(\alpha +\beta_{A}\right)\cdot \log\left(\frac{\alpha +\beta_{A}x}{\alpha +\beta_{A}}\right)+\beta_{A}\left(x-1\right)\right]\cdot \frac{1-{\left(\beta_{A}\right)}^{n}}{1-\beta_{A}}\cdot \left[X_{0}-\frac{\alpha }{1-\beta_{A}}\right] & \;\\ +\;[\frac{\alpha }{\beta_{A}\left(1-\beta_{A}\right)}\left(-\left(\alpha +\beta_{A}\right)\cdot \log\left(\frac{\alpha +\beta_{A}x}{\alpha +\beta_{A}}\right)+\beta_{A}\left(x-1\right)\right) & \;\\ \;+\frac{\alpha }{\beta_{A}}\left(\alpha +\beta_{A}\right)\cdot \log\left(\frac{\alpha +\beta_{A}x}{\alpha +\beta_{A}}\right)-\alpha \left(x-1\right)]\cdot n\;, & \;\mathrm{if}\;\beta_{A}\neq 1,\;\\ \left[-\left(\alpha +1\right)\cdot \log\left(\frac{\alpha +x}{\alpha +1}\right)+x-1\right]\cdot \left[\frac{\alpha }{2}\cdot n^{2}+\left(X_{0}+\frac{\alpha }{2}\right)\cdot n\right] & \;\\ +\left[\left(\alpha +1\right)\cdot \log\left(\frac{\alpha +x}{\alpha +1}\right)-x+1\right]\cdot \alpha \cdot n\;, & \;\mathrm{if}\;\beta_{A}=1. \end{array}\right.$$

To prove that $E_{0,X_{0},n}^{L,sec}>0$ for all $X_{0}\in N$ and all n∈N it suffices to show that $E_{0,X_{0},n}^{L,sec}\left(1\right)=\left(\frac{\partial }{\partial x}E_{0,X_{0},n}^{L,sec}\right)\left(1\right)=0$ and $\left(\frac{\partial^{2}}{\partial x^{2}}E_{0,X_{0},n}^{L,sec}\right)\left(x\right)>0$ for all x∈]0,∞[\{1}. The assertion $E_{0,X_{0},n}^{L,sec}\left(1\right)=0$ is trivial from (A34). Moreover, we obtain

$$\left(\frac{\partial }{\partial x}E_{0,X_{0},n}^{L,sec}\right)\left(x\right)\;=\;\left\{\begin{array}{cc} \beta_{A}\cdot \left[1-\frac{\alpha +\beta_{A}}{\alpha +\beta_{A}x}\right]\cdot \frac{1-{\left(\beta_{A}\right)}^{n}}{1-\beta_{A}}\cdot \left[X_{0}-\frac{\alpha }{1-\beta_{A}}\right] & \;\\ +\;\alpha \cdot \left(1-\frac{\alpha +\beta_{A}}{\alpha +\beta_{A}x}\right)\cdot \frac{\beta_{A}}{1-\beta_{A}}\cdot n\;, & \mathrm{if}\;\beta_{A}\neq 1,\;\\ \left[1-\frac{\alpha +1}{\alpha +x}\right]\cdot \left[\frac{\alpha }{2}\cdot n^{2}+\left(X_{0}-\frac{\alpha }{2}\right)\cdot n\right]\;, & \mathrm{if}\;\beta_{A}=1, \end{array}\right.$$

which immediately yields $\left(\frac{\partial }{\partial x}E_{0,X_{0},n}^{L,sec}\right)\left(1\right)=0$. For the second derivative we get

(A35) $$\left(\frac{\partial^{2}}{\partial x^{2}}E_{0,X_{0},n}^{L,sec}\right)\left(x\right)\;=\;\left\{\begin{array}{cc} \frac{\left(\alpha +\beta_{A}\right)\cdot \beta_{A}^{2}}{{\left(\alpha +\beta_{A}x\right)}^{2}}\cdot \frac{1-{\left(\beta_{A}\right)}^{n}}{1-\beta_{A}}\cdot \left[X_{0}-\frac{\alpha }{1-\beta_{A}}\right] & \;\\ +\;\alpha \frac{\alpha +\beta_{A}}{{\left(\alpha +\beta_{A}x\right)}^{2}}\cdot \frac{\beta_{A}^{2}}{1-\beta_{A}}\cdot n\;>0, & \mathrm{if}\;\beta_{A}\neq 1,\;\\ \frac{\alpha +1}{{\left(\alpha +x\right)}^{2}}\cdot \left[\frac{\alpha }{2}\cdot n^{2}+\left(X_{0}-\frac{\alpha }{2}\right)\cdot n\right]\;>0, & \mathrm{if}\;\beta_{A}=1, \end{array}\right.$$

where the strict positivity of $E_{0,X_{0},n}^{L,sec}$ in the case $\beta_{A}\neq 1$ follows immediately by replacing $X_{0}$ with 0 and by using the obvious relation $\frac{1}{1-\beta_{A}}\cdot \left[n-\frac{1-\beta_{A}^{n}}{1-\beta_{A}}\right]=\frac{1}{1-\beta_{A}}\sum_{k=0}^{n-1}\left(1-\beta_{A}^{k}\right)>0$. The strict positivity in the case $\beta_{A}=1$ is trivial by inspection. For the constellation $P_{\mathrm{SP},4}$ with parameters $\beta :=\beta_{•}:=\beta_{A}=\beta_{H}$, $\alpha_{A}\neq \alpha_{H}$, the strict positivity of $E_{X_{0},n}^{L}>0$ follows by showing that $E_{y,X_{0},n}^{L,tan}$ converges from above to zero as *y* tends to infinity. This is done by proving $\lim_{y\to \infty }y\cdot E_{y,X_{0},n}^{L,tan}\in ]0,\infty [$. To see this, let us first observe that by l’Hospital’s rule we get

$$\lim_{y\to \infty }y\cdot \log\left(\frac{\alpha_{A}+\beta y}{\alpha_{H}+\beta y}\right)\;=\;\frac{\alpha_{A}-\alpha_{H}}{\beta }\;\mathrm{as}\mathrm{well}\mathrm{as}\;\lim_{y\to \infty }y\cdot \left(1-\frac{\alpha_{A}+\beta y}{\alpha_{H}+\beta y}\right)\;=\;-\frac{\alpha_{A}-\alpha_{H}}{\beta }\;.$$

From this and (72), we obtain $\lim_{y\to \infty }y\cdot E_{y,X_{0},n}^{L,tan}=\frac{{\left(\alpha_{A}-\alpha_{H}\right)}^{2}}{\beta }\cdot n\;>\;0$ in both cases β≠1 and β=1. Finally, for the parameter case $P_{\mathrm{SP},3c}$ we consider the bound $E_{y^{*},X_{0},n}^{L,tan}$, with $y^{*}=\frac{\alpha_{A}-\alpha_{H}}{\beta_{H}-\beta_{A}}$. Since $\alpha_{A}+\beta_{A}y^{*}=\alpha_{H}+\beta_{H}y^{*}$, it is easy to see that $E_{y^{*},X_{0},n}^{L,tan}=0$ for all n∈N. However, the condition $\left(\frac{\partial }{\partial y}E_{y,X_{0},n}^{L,tan}\right)\left(y^{*}\right)\neq 0$ implies that $\sup_{y\geq 0}E_{y,X_{0},n}^{L,tan}>0$. The explicit form (75) of this condition follows from

$$\left(\frac{\partial }{\partial y}E_{y,X_{0},n}^{L,tan}\right)\left(y\right)\;=\;\left\{\begin{array}{cc} \frac{{\left(\alpha_{A}\beta_{H}-\alpha_{H}\beta_{A}\right)}^{2}}{f_{A}\left(y\right){\left(f_{H}\left(y\right)\right)}^{2}}\cdot \frac{1-{\left(\beta_{A}\right)}^{n}}{1-\beta_{A}}\cdot \left[X_{0}-\frac{\alpha_{A}}{1-\beta_{A}}\right]\\ +\;\frac{\alpha_{A}\beta_{H}-\alpha_{H}\beta_{A}}{{\left(f_{H}\left(y\right)\right)}^{2}}\cdot \left[\frac{\alpha_{A}}{\beta_{A}\left(1-\beta_{A}\right)f_{A}\left(y\right)}-\frac{\alpha_{A}\beta_{H}-\alpha_{H}\beta_{A}}{\beta_{A}}\right]\cdot n\;, & \mathrm{if}\;\beta_{A}\neq 1,\;\\ \frac{{\left(\alpha_{A}\beta_{H}-\alpha_{H}\right)}^{2}}{f_{A}\left(y\right){\left(f_{H}\left(y\right)\right)}^{2}}\cdot \left[\frac{\alpha_{A}}{2}\cdot n^{2}+\left(X_{0}+\frac{\alpha_{A}}{2}\right)\cdot n\right]\;-\;\frac{{\left(\alpha_{A}\beta_{H}-\alpha_{H}\right)}^{2}}{{\left(f_{H}\left(y\right)\right)}^{2}}\cdot n\;, & \mathrm{if}\;\beta_{A}=1, \end{array}\right.$$

y≥0, by using the particular choice $y=y^{*}$ together with $f_{A}\left(y^{*}\right)=f_{H}\left(y^{*}\right)=-\frac{\alpha_{A}\beta_{H}-\alpha_{H}\beta_{A}}{\beta_{A}-\beta_{H}}$. □

### Appendix A.3. Proofs and Auxiliary Lemmas for Section 6

**Proof of Lemma** **2.** A closed-form representation of a sequence ${\left({\tilde{a}}_{n}\right)}_{n\in N_{0}}$ defined in (83) to (85) is given by the formula

(A36) $${\tilde{a}}_{n}\;=\;\sum_{k=0}^{n-1}\left(c+\rho_{k}\right)d^{n-1-k}.$$

This can be seen by induction: from (83) we obtain with ${\tilde{a}}_{0}=0$ for the first element ${\tilde{a}}_{1}=c+\rho_{0}=\sum_{k=0}^{0}\left(c+\rho_{k}\right)d^{-k}$. Supposing that (A36) holds for the *n*-th element, the induction step is

$${\tilde{a}}_{n+1}\;=\;c+d\cdot {\tilde{a}}_{n}+\rho_{n}\;=\;c+d\cdot \sum_{k=0}^{n-1}\left(c+\rho_{k}\right)d^{n-1-k}+\rho_{n}\;=\;\sum_{k=0}^{n}\left(c+\rho_{k}\right)d^{n-k}\;.$$

In order to obtain the explicit representation of ${\tilde{a}}_{n}$, we consider first the case 0≤ν<ϰ<d and $\rho_{n}=K_{1}\cdot \varkappa^{n}+K_{2}\cdot \nu^{n}$, which leads to

(A37) $$\begin{array}{ccc} {\tilde{a}}_{n} & = & d^{n-1}\sum_{k=0}^{n-1}\left(c\cdot d^{-k}+K_{1}\cdot {\left(\frac{\varkappa }{d}\right)}^{k}+K_{2}\cdot {\left(\frac{\nu }{d}\right)}^{k}\right)\\ & = & d^{n-1}\cdot \left[c\cdot \frac{1-d^{-n}}{1-d^{-1}}+K_{1}\cdot \frac{1-{\left(\frac{\varkappa }{d}\right)}^{n}}{1-\frac{\varkappa }{d}}+K_{2}\cdot \frac{1-{\left(\frac{\nu }{d}\right)}^{n}}{1-\frac{\nu }{d}}\right]\\ & = & \frac{c}{1-d}\left(1-d^{n}\right)+K_{1}\cdot \frac{d^{n}-\varkappa^{n}}{d-\varkappa }+K_{2}\cdot \frac{d^{n}-\nu^{n}}{d-\nu }. \end{array}$$

Hence, for the corresponding sum we get

(A38) $$\begin{array}{ccc} \sum_{k=1}^{n}{\tilde{a}}_{k} & = & \sum_{k=1}^{n}\left[\frac{c}{1-d}+\left(\frac{K_{1}}{d-\varkappa }+\frac{K_{2}}{d-\nu }-\frac{c}{1-d}\right)\cdot d^{k}-\frac{K_{1}}{d-\varkappa }\cdot \varkappa^{k}-\frac{K_{2}}{d-\nu }\cdot \nu^{k}\right]\\ & = & \frac{c}{1-d}\cdot n+\left(\frac{K_{1}}{d-\varkappa }+\frac{K_{2}}{d-\nu }-\frac{c}{1-d}\right)\cdot \frac{d\cdot (1-d^{n})}{1-d}-\frac{K_{1}\cdot \varkappa \cdot \left(1-\varkappa^{n}\right)}{\left(d-\varkappa )(1-\varkappa \right)}-\frac{K_{2}\cdot \nu \cdot \left(1-\nu^{n}\right)}{\left(d-\nu )(1-\nu \right)}. \end{array}$$

Consider now the case 0≤ν<ϰ=d. Then some expressions in (A37) and (A38) have a zero denominator. In this case, the evaluation of (A36) becomes

(A39) $$\begin{array}{ccc} {\tilde{a}}_{n} & = & d^{n-1}\sum_{k=0}^{n-1}\left(c\cdot d^{-k}+K_{1}+K_{2}\cdot {\left(\frac{\nu }{d}\right)}^{k}\right)=d^{n-1}\cdot \left[c\cdot \frac{1-d^{-n}}{1-d^{-1}}+K_{1}\cdot n+K_{2}\cdot \frac{1-{\left(\frac{\nu }{d}\right)}^{n}}{1-\frac{\nu }{d}}\right]\\ & = & \frac{c}{1-d}\left(1-d^{n}\right)+K_{1}\cdot n\cdot d^{n-1}+K_{2}\cdot \frac{d^{n}-\nu^{n}}{d-\nu }. \end{array}$$

Before we calculate the corresponding sum $\sum_{k=1}^{n}{\tilde{a}}_{k}$, we notice that

$$\sum_{k=1}^{n}k\cdot d^{k-1}\;=\;\sum_{k=1}^{n}\frac{\partial }{\partial d}d^{k}\;=\;\frac{\partial }{\partial d}\sum_{k=1}^{n}d^{k}\;=\;\frac{\partial }{\partial d}\left(\frac{d\cdot (1-d^{n})}{1-d}\right)\;=\;\frac{1-n\cdot d^{n}\left(1-d\right)-d^{n}}{{\left(1-d\right)}^{2}}\;.$$

Using this fact, we obtain

$$\begin{array}{c} \;\sum_{k=1}^{n}{\tilde{a}}_{k}\;=\;\sum_{k=1}^{n}\left[\frac{c}{1-d}\left(1-d^{k}\right)+K_{1}\cdot k\cdot d^{k-1}+K_{2}\cdot \frac{d^{k}-\nu^{k}}{d-\nu }\right]\\ =\frac{c}{1-d}\cdot n+\sum_{k=1}^{n}\left(\frac{K_{2}}{d-\nu }-\frac{c}{1-d}\right)d^{k}+K_{1}\sum_{k=1}^{n}k\cdot d^{k-1}-\frac{K_{2}}{d-\nu }\sum_{k=1}^{n}\nu^{k}\\ =\left(\frac{K_{2}}{d-\nu }-\frac{c}{1-d}\right)\frac{d\cdot (1-d^{n})}{1-d}+K_{1}\cdot \frac{1-n\cdot d^{n}\left(1-d\right)-d^{n}}{{\left(1-d\right)}^{2}}-\frac{K_{2}\cdot \nu \left(1-\nu^{n}\right)}{\left(d-\nu )(1-\nu \right)}+\frac{c}{1-d}\cdot n\\ =\left(\frac{K_{1}}{d(1-d)}+\frac{K_{2}}{d-\nu }-\frac{c}{1-d}\right)\frac{d\cdot (1-d^{n})}{1-d}-\frac{K_{2}\cdot \nu \left(1-\nu^{n}\right)}{\left(d-\nu )(1-\nu \right)}+\left(\frac{c}{1-d}-\frac{K_{1}\cdot d^{n}}{1-d}\right)\cdot n.\;\;\;□ \end{array}$$

**Proof of Lemma** **3.** (a) In this case we have $0<q<\beta_{\lambda }$. To prove part (i), we consider the function $\xi_{\lambda }^{\left(q\right)}\left(\cdot \right)$ on $\left[x_{0}^{\left(q\right)},0\right]$, the range of the sequence ${\left(a_{n}^{\left(q\right)}\right)}_{n\in N}$ (recall Properties 1 (P1)). For tackling the left-hand inequality in (i), we compare $\xi_{\lambda }^{\left(q\right)}\left(x\right)\;=\;q\cdot e^{x}-\beta_{\lambda }$ with the quadratic function

(A40) $${\underline{\Upsilon }}_{\lambda }^{\left(q\right)}\left(x\right)\;:=\;\frac{q}{2}\;e^{x_{0}^{\left(q\right)}}\cdot x^{2}+qe^{x_{0}^{\left(q\right)}}\left(1-x_{0}^{\left(q\right)}\right)\cdot x+x_{0}^{\left(q\right)}\left(1-qe^{x_{0}^{\left(q\right)}}+\frac{q}{2}e^{x_{0}^{\left(q\right)}}x_{0}^{\left(q\right)}\right).$$

Clearly, one has the relations ${\underline{\Upsilon }}_{\lambda }^{\left(q\right)}\left(x_{0}^{\left(q\right)}\right)=x_{0}^{\left(q\right)}=\xi_{\lambda }^{\left(q\right)}\left(x_{0}^{\left(q\right)}\right)$, ${\underline{\Upsilon }}_{\lambda }^{(q)\;\prime }\left(x_{0}^{\left(q\right)}\right)=q\cdot e^{x_{0}^{\left(q\right)}}=\xi_{\lambda }^{(q)\;\prime }\left(x_{0}^{\left(q\right)}\right)$, and ${\underline{\Upsilon }}_{\lambda }^{(q)\;\prime \prime }\left(x\right)<\xi_{\lambda }^{(q)\;\prime \prime }\left(x\right)$ for all $x\in ]x_{0}^{\left(q\right)},0]$. Hence, ${\underline{\Upsilon }}_{\lambda }^{\left(q\right)}\left(\cdot \right)$ is on $]x_{0}^{\left(q\right)},0]$ a strict lower functional bound of $\xi_{\lambda }^{\left(q\right)}\left(\cdot \right)$. We are now ready to prove the left-hand inequality in (i) by induction. For n=1, we easily see that ${\underline{a}}_{1}^{\left(q\right)}<a_{1}^{\left(q\right)}$ iff $x_{0}^{\left(q\right)}\left(1-qe^{x_{0}^{\left(q\right)}}+\frac{q}{2}e^{x_{0}^{\left(q\right)}}x_{0}^{\left(q\right)}\right)<q-\beta_{\lambda }$ iff ${\underline{\Upsilon }}_{\lambda }^{\left(q\right)}\left(0\right)<\xi_{\lambda }^{\left(q\right)}\left(0\right)$, and the latter is obviously true. Let us assume that ${\underline{a}}_{n}^{\left(q\right)}\leq a_{n}^{\left(q\right)}$ holds. From this, (93), (78) and (80) we obtain

$$\begin{array}{c} 0\;<\;{\underline{\rho }}_{n}^{\left(q\right)}\;=\;\frac{q}{2}\;e^{x_{0}^{\left(q\right)}}{\left(x_{0}^{\left(q\right)}\cdot {\left(q\cdot e^{x_{0}^{\left(q\right)}}\right)}^{n}\;\right)}^{2}\;=\;\frac{q}{2}\;e^{x_{0}^{\left(q\right)}}{\left(a_{n}^{(q),T}-x_{0}^{\left(q\right)}\right)}^{2}\\ <\;\frac{q}{2}\;e^{x_{0}^{\left(q\right)}}{\left(a_{n}^{\left(q\right)}-x_{0}^{\left(q\right)}\right)}^{2}\;=\;{\underline{\Upsilon }}_{\lambda }^{\left(q\right)}\left(a_{n}^{\left(q\right)}\right)-d^{(q),T}\cdot a_{n}^{\left(q\right)}-x_{0}^{\left(q\right)}\cdot \left(1-d^{(q),T}\right)\\ <\;\xi_{\lambda }^{\left(q\right)}\left(a_{n}^{\left(q\right)}\right)-d^{(q),T}\cdot a_{n}^{\left(q\right)}-x_{0}^{\left(q\right)}\cdot \left(1-d^{(q),T}\right)\\ <\;a_{n+1}^{\left(q\right)}-d^{(q),T}\cdot {\underline{a}}_{n}^{\left(q\right)}-x_{0}^{\left(q\right)}\cdot \left(1-d^{(q),T}\right)\;=\;a_{n+1}^{\left(q\right)}-\xi_{\lambda }^{(q),T}\left({\underline{a}}_{n}^{\left(q\right)}\right)\;. \end{array}$$

Thus, there holds ${\underline{a}}_{n+1}^{\left(q\right)}<a_{n+1}^{\left(q\right)}$. For the right-hand inequality in (i), we proceed analogously:

(A41) $${\bar{\Upsilon }}_{\lambda }^{\left(q\right)}\left(x\right)\;:=\;\frac{q}{2}\;e^{x_{0}^{\left(q\right)}}\cdot x^{2}\;+\;\left(1-\frac{q}{2}\;e^{x_{0}^{\left(q\right)}}x_{0}^{\left(q\right)}-\frac{q-\beta_{\lambda }}{x_{0}^{\left(q\right)}}\right)\cdot x\;+\;q-\beta_{\lambda }$$

satisfies ${\bar{\Upsilon }}_{\lambda }^{\left(q\right)}\left(x_{0}^{\left(q\right)}\right)=x_{0}^{\left(q\right)}=\xi_{\lambda }^{\left(q\right)}\left(x_{0}^{\left(q\right)}\right)$, ${\bar{\Upsilon }}_{\lambda }^{\left(q\right)}\left(0\right)=q-\beta_{\lambda }=\xi_{\lambda }^{\left(q\right)}\left(0\right)$ as well as ${\bar{\Upsilon }}_{\lambda }^{(q)\;\prime \prime }\left(x\right)<\xi_{\lambda }^{(q)\;\prime \prime }\left(x\right)$ for all $x\in ]x_{0}^{\left(q\right)},0]$. Hence, ${\bar{\Upsilon }}_{\lambda }^{\left(q\right)}\left(\cdot \right)$ is on $]x_{0}^{\left(q\right)},0]$ a strict upper functional bound of $\xi_{\lambda }^{\left(q\right)}\left(\cdot \right)$. Let us first observe the obvious relation ${\bar{a}}_{1}^{\left(q\right)}=q-\beta_{\lambda }=a_{1}^{\left(q\right)}<0$, and assume that ${\bar{a}}_{n}^{\left(q\right)}\geq a_{n}^{\left(q\right)}$ (n∈N) holds. From this, (95), (79), and (80) we obtain the desired inequality ${\bar{a}}_{n+1}^{\left(q\right)}>a_{n+1}^{\left(q\right)}$ by

$$\begin{array}{c} 0>{\bar{\rho }}_{n}^{\left(q\right)}=-\Gamma_{<}^{\left(q\right)}{\left(d^{(q),T}\right)}^{n}\cdot \frac{a_{n}^{(q),S}}{x_{0}^{\left(q\right)}}\;=\;\frac{q}{2}\;e^{x_{0}^{\left(q\right)}}\left(a_{n}^{(q),T}-x_{0}^{\left(q\right)}\right)\cdot a_{n}^{(q),S}\\ \geq \frac{q}{2}\;e^{x_{0}^{\left(q\right)}}\left(a_{n}^{\left(q\right)}-x_{0}^{\left(q\right)}\right)\cdot a_{n}^{\left(q\right)}\;=\;{\bar{\Upsilon }}_{\lambda }^{\left(q\right)}\left(a_{n}^{\left(q\right)}\right)-d^{(q),S}\cdot a_{n}^{\left(q\right)}-\left(q-\beta_{\lambda }\right)\\ >\;\xi_{\lambda }^{\left(q\right)}\left(a_{n}^{\left(q\right)}\right)-d^{(q),S}\cdot a_{n}^{\left(q\right)}-\left(q-\beta_{\lambda }\right)\;\geq \;a_{n+1}^{\left(q\right)}-d^{(q),S}\cdot {\bar{a}}_{n}^{\left(q\right)}-\left(q-\beta_{\lambda }\right)=\;a_{n+1}^{\left(q\right)}-\xi_{\lambda }^{(q),S}\left({\bar{a}}_{n}^{\left(q\right)}\right)\;. \end{array}$$

The explicit representations of the sequences ${\left(a_{n}^{\left(q\right)}\right)}_{n\in N}$, ${\left({\underline{a}}_{n}^{\left(q\right)}\right)}_{n\in N}$ and ${\left({\bar{a}}_{n}^{\left(q\right)}\right)}_{n\in N}$ follow from (86) by incorporating the appropriate constants mentioned in the prelude of Lemma 3. With (83) to (85) and (86) we immediately achieve ${\underline{a}}_{n}^{\left(q\right)}>a_{n}^{(q),T}$ for all n∈N. Analogously, for all n≥2, we get ${\bar{\rho }}_{n-1}<0$, which implies that ${\bar{a}}_{n}^{\left(q\right)}<a_{n}^{(q),S}$ for all n≥2. For n=1 one obtains ${\bar{\rho }}_{0}=0$ as well as ${\bar{a}}_{1}^{\left(q\right)}=a_{1}^{(q),S}=a_{1}^{\left(q\right)}=q-\beta_{\lambda }$. For the second part (ii), we employ the representation (A36) which leads to

$$\begin{array}{cc} & {\underline{a}}_{n}^{\left(q\right)}\;=\;\sum_{k=0}^{n-1}{\left(d^{(q),T}\right)}^{n-1-k}\cdot \left({\underline{\rho }}_{k}^{\left(q\right)}+x_{0}^{\left(q\right)}\cdot \left(1-d^{(q),T}\right)\right)\;\\ \mathrm{as}\mathrm{well}\mathrm{as}\; & {\bar{a}}_{n}^{\left(q\right)}\;=\;\sum_{k=0}^{n-1}{\left(d^{(q),S}\right)}^{n-1-k}\cdot \left({\bar{\rho }}_{k}^{\left(q\right)}+\left(q-\beta_{\lambda }\right)\right)\;. \end{array}$$

The strict decreasingness of both sequences follows from

$${\underline{\rho }}_{k}^{\left(q\right)}+x_{0}^{\left(q\right)}\left(1-d^{(q),T}\right)\;=\;\frac{qe^{x_{0}^{\left(q\right)}}}{2}{\left(x_{0}^{\left(q\right)}\right)}^{2}{\left(d^{(q),T}\right)}^{2n}+x_{0}^{\left(q\right)}\left(1-d^{(q),T}\right)\;\leq \;{\underline{\Upsilon }}_{\lambda }^{\left(q\right)}\left(0\right)\;<\;\xi_{\lambda }^{\left(q\right)}\left(0\right)\;=\;q-\beta_{\lambda }\;<\;0\;$$

and from the fact that ${\bar{\rho }}_{k}^{\left(q\right)}\leq 0$ for all $k\in N_{0}$ and $q<\beta_{\lambda }$. Part (iii) follows directly from (i), since $d^{(q),T},\;d^{(q),S}\in ]0,1[$. Let us now prove part (b), where $\max\left\{0,\beta_{\lambda }\right\}<q<\min\left\{1\;,\;e^{\beta_{\lambda }-1}\right\}$ is assumed. To tackle part (i), we compare $\xi_{\lambda }^{\left(q\right)}\left(x\right)\;=\;q\cdot e^{x}-\beta_{\lambda }$ with the quadratic function

(A42) $${\underline{\underline{\upsilon }}}_{\lambda }^{\left(q\right)}\left(x\right)\;:=\;\frac{q}{2}\cdot x^{2}+q\cdot \left(e^{x_{0}^{\left(q\right)}}-x_{0}^{\left(q\right)}\right)\cdot x+x_{0}^{\left(q\right)}\left(1-qe^{x_{0}^{\left(q\right)}}+\frac{q}{2}x_{0}^{\left(q\right)}\right)\;>\;0$$

on the interval $\left[0,x_{0}^{\left(q\right)}\right]$. Clearly, we have ${\underline{\underline{\upsilon }}}_{\lambda }^{\left(q\right)}\left(x_{0}^{\left(q\right)}\right)=\xi_{\lambda }^{\left(q\right)}\left(x_{0}^{\left(q\right)}\right)=x_{0}^{\left(q\right)}$, $\;{\underline{\underline{\upsilon }}}_{\lambda }^{(q)\;\prime }\left(x_{0}^{\left(q\right)}\right)=\xi_{\lambda }^{(q)\;\prime }\left(x_{0}^{\left(q\right)}\right)=qe^{x_{0}^{\left(q\right)}}$ and $0<{\underline{\underline{\upsilon }}}_{\lambda }^{(q)\;\prime \prime }\left(x\right)<\xi_{\lambda }^{(q)\;\prime \prime }\left(x\right)$ for all $x\in ]0,x_{0}^{\left(q\right)}]$. Thus, ${\underline{\underline{\upsilon }}}_{\lambda }^{\left(q\right)}\left(\cdot \right)$ constitutes a positive functional lower bound for $\xi_{\lambda }^{\left(q\right)}\left(\cdot \right)$ on $\left[0,x_{0}^{\left(q\right)}\right]$. Let us now prove the left-hand inequality of (i) by induction: for n=1 we get ${\underline{a}}_{1}^{\left(q\right)}={\underline{\underline{\upsilon }}}_{\lambda }^{\left(q\right)}\left(0\right)<\xi_{\lambda }^{\left(q\right)}\left(0\right)=a_{1}^{\left(q\right)}$. Moreover, by assuming ${\underline{a}}_{n}^{\left(q\right)}\leq a_{n}^{\left(q\right)}$ for n∈N, we obtain with the above-mentioned considerations and (93), (80) and (82)

$$\begin{array}{c} 0\;<\;{\underline{\rho }}_{n}^{\left(q\right)}\;=\;\Gamma_{>}^{\left(q\right)}{\left(d^{(q),S}\right)}^{2n}\;=\;\frac{q}{2}\cdot {\left(a_{n}^{(q),S}-x_{0}^{\left(q\right)}\right)}^{2}\;<\;\frac{q}{2}\cdot {\left(a_{n}^{\left(q\right)}-x_{0}^{\left(q\right)}\right)}^{2}\\ =\;\frac{q}{2}{\left(a_{n}^{\left(q\right)}\right)}^{2}+q\cdot \left(e^{x_{0}^{\left(q\right)}}-x_{0}^{\left(q\right)}\right)\cdot a_{n}^{\left(q\right)}+x_{0}^{\left(q\right)}\cdot \left(1-qe^{x_{0}^{\left(q\right)}}+\frac{q}{2}x_{0}^{\left(q\right)}\right)-d^{(q),T}a_{n}^{\left(q\right)}-c^{(q),T}\\ =\;{\underline{\underline{\upsilon }}}_{\lambda }^{\left(q\right)}\left(a_{n}^{\left(q\right)}\right)-d^{(q),T}a_{n}^{\left(q\right)}-c^{(q),T}\;<\;\xi_{\lambda }^{\left(q\right)}\left(a_{n}^{\left(q\right)}\right)-d^{(q),T}a_{n}^{\left(q\right)}-c^{(q),T}\\ <\;a_{n+1}^{\left(q\right)}-d^{(q),T}{\underline{a}}_{n}^{\left(q\right)}-c^{(q),T}\;=\;a_{n+1}^{\left(q\right)}-\xi_{\lambda }^{(q),T}\left({\underline{a}}_{n}^{\left(q\right)}\right)\;. \end{array}$$

Hence, ${\underline{a}}_{n+1}^{\left(q\right)}<a_{n+1}^{\left(q\right)}$. For the right-hand inequality in part (i), we define the quadratic function

(A43) $${\bar{\bar{\upsilon }}}_{\lambda }^{\left(q\right)}\left(x\right)\;:=\;\frac{q}{2}\cdot x^{2}+\left(1-\frac{q}{2}x_{0}^{\left(q\right)}-\frac{q-\beta_{\lambda }}{x_{0}^{\left(q\right)}}\right)\cdot x+q-\beta_{\lambda }\;,$$

which is a functional upper bound for $\xi_{\lambda }^{\left(q\right)}\left(\cdot \right)$ on the interval $\left[0,x_{0}^{\left(q\right)}\right]$ since there holds ${\bar{\bar{\upsilon }}}_{\lambda }^{\left(q\right)}\left(0\right)=\xi_{\lambda }^{\left(q\right)}\left(0\right)=q-\beta_{\lambda }$, ${\bar{\bar{\upsilon }}}_{\lambda }^{\left(q\right)}\left(x_{0}^{\left(q\right)}\right)=\xi_{\lambda }^{\left(q\right)}\left(x_{0}^{\left(q\right)}\right)=x_{0}^{\left(q\right)}$ and additionally ${\bar{\bar{\upsilon }}}_{\lambda }^{(q)\;\prime \prime }\left(x\right)=q<qe^{x}=\xi_{\lambda }^{(q)\;\prime \prime }\left(x\right)$ on $]0,x_{0}^{\left(q\right)}[$. Obviously, ${\bar{a}}_{1}^{\left(q\right)}=q-\beta_{\lambda }=a_{1}^{\left(q\right)}$. By assuming ${\bar{a}}_{n}^{\left(q\right)}\geq a_{n}^{\left(q\right)}$ for n∈N, we obtain with (80), (82) and (95)

(A44) $$\begin{array}{c} 0\;>\;{\bar{\rho }}_{n}^{\left(q\right)}\;=\;-\;\Gamma_{>}^{\left(q\right)}\cdot {\left(d^{(q),S}\right)}^{n}\cdot \left(1-{\left(d^{(q),T}\right)}^{n}\right)\;=\;-\frac{q}{2}\cdot \left(x_{0}-a_{n}^{(q),S}\right)\cdot a_{n}^{(q),T}\\ >\;-\frac{q}{2}\cdot \left(x_{0}-a_{n}^{\left(q\right)}\right)\cdot a_{n}^{\left(q\right)}\;=\;{\bar{\bar{\upsilon }}}_{\lambda }^{\left(q\right)}\left(a_{n}^{\left(q\right)}\right)-\frac{x_{0}^{\left(q\right)}-\left(q-\beta_{\lambda }\right)}{x_{0}^{\left(q\right)}}\cdot a_{n}^{\left(q\right)}-\left(q-\beta_{\lambda }\right)\\ >\;\xi_{\lambda }^{\left(q\right)}\left(a_{n}^{\left(q\right)}\right)-d^{(q),S}a_{n}^{\left(q\right)}-c^{(q),S}\;>\;\xi_{\lambda }^{\left(q\right)}\left(a_{n}^{\left(q\right)}\right)-d^{(q),S}{\bar{a}}_{n}^{(q),S}-c^{(q),S}=\;a_{n+1}^{\left(q\right)}-\xi_{\lambda }^{(q),S}\left({\bar{a}}_{n}^{\left(q\right)}\right)\;,\;\; \end{array}$$

which implies ${\bar{a}}_{n+1}^{\left(q\right)}>a_{n+1}^{\left(q\right)}$. The explicit representations of the sequences ${\left({\underline{a}}_{n}^{\left(q\right)}\right)}_{n\in N}$ and ${\left({\bar{a}}_{n}^{\left(q\right)}\right)}_{n\in N}$ follow from (86) by employing the appropriate constants mentioned in the prelude of Lemma 3. By means of (83) to (85) and (86), we directly get ${\underline{a}}_{n}^{\left(q\right)}>a_{n}^{(q),T}$ for all n∈N, whereas ${\bar{a}}_{n}^{\left(q\right)}<a_{n}^{(q),S}$ holds only for all n≥2, since ${\bar{\rho }}_{0}=0$ implies that ${\bar{a}}_{1}^{\left(q\right)}=a_{1}^{(q),S}=a_{1}^{\left(q\right)}=q-\beta_{\lambda }$. The second part (ii) can be proved in the same way as part (ii) of (a), by employing the representation (A36). For the lower bound one has

$${\underline{a}}_{n}^{\left(q\right)}\;=\;\sum_{k=0}^{n-1}{\left(d^{(q),T}\right)}^{n-1-k}\cdot \left[c^{(q),T}+{\underline{\rho }}_{k}^{\left(q\right)}\right]\;,\;\mathrm{with}\;c^{(q),T}>0\;\mathrm{and}\;{\underline{\rho }}_{k}^{\left(q\right)}>0.$$

For the upper bound we get

$${\bar{a}}_{n}^{\left(q\right)}\;=\;\sum_{k=0}^{n-1}{\left(d^{(q),S}\right)}^{n-1-k}\cdot \left[c^{(q),S}+{\bar{\rho }}_{k}^{\left(q\right)}\right],$$

hence it is enough to show $c^{(q),S}+{\bar{\rho }}_{n}^{\left(q\right)}>0$ for all $n\in N_{0}$. Considering the first two lines of calculation (A44) and incorporating $c^{(q),S}=q-\beta_{\lambda }$, this can be seen from

$$c^{(q),S}+{\bar{\rho }}_{n}^{\left(q\right)}\;>\;{\bar{\bar{\upsilon }}}_{\lambda }^{\left(q\right)}\left(a_{n}^{\left(q\right)}\right)-\frac{x_{0}^{\left(q\right)}-\left(q-\beta_{\lambda }\right)}{x_{0}^{\left(q\right)}}\cdot a_{n}^{\left(q\right)}\;=\;{\bar{\bar{\upsilon }}}_{\lambda }^{\left(q\right)}\left(a_{n}^{\left(q\right)}\right)-d^{(q),S}\cdot a_{n}^{\left(q\right)}\;>\;0\;,$$

because on $\left[0,x_{0}^{\left(q\right)}\right]$ there holds $d^{(q),S}\cdot x\;<\;x\;<\;{\bar{\bar{\upsilon }}}_{\lambda }^{\left(q\right)}\left(x\right)$. The last part (iii) can be easily deduced from (i) together with $\lim_{n\to \infty }n\cdot {\left(d^{(q),S}\right)}^{n-1}=0$. □

The proofs of all Theorems 5–9 are mainly based on the following

**Lemma** **A4.**
*Recall the quantity ${\tilde{B}}_{\lambda ,X_{0},n}^{\left(p,q\right)}$ from (42) for general p≥0,q>0 (notice that we do not consider parameters p<0, q≤0 in Section 6) as well as the constants $d^{(q),T},\;d^{(q),S}$ and $\Gamma_{<}^{\left(q\right)},\;\Gamma_{>}^{\left(q\right)}$ defined in (76), (77) and (91). For all $\left(\beta_{A},\beta_{H},\alpha_{A},\alpha_{H},\lambda \right)\in P\times R\backslash \left\{0,1\right\}$, all initial population sizes $X_{0}\in N$ and all observation horizons n∈N there holds*

(a) *in the case p≥0 and $0<q<\beta_{\lambda }$*
  (A45) $$\begin{array}{ccc} {\tilde{B}}_{\lambda ,X_{0},n}^{\left(p,q\right)} & \geq & \exp\{x_{0}^{\left(q\right)}\cdot \left[X_{0}-\frac{p}{q}\cdot \frac{d^{(q),T}}{1-d^{(q),T}}\right]\cdot \left(1-{\left(d^{(q),T}\right)}^{n}\right)\;+\;\left(\frac{p}{q}\cdot \left(\beta_{\lambda }+x_{0}^{\left(q\right)}\right)-\alpha_{\lambda }\right)\cdot n\\ & & \;+\;{\underline{\zeta }}_{n}^{\left(q\right)}\cdot X_{0}\;+\;\frac{p}{q}\cdot {\underline{\vartheta }}_{n}^{\left(q\right)}\}\;=:\;C_{\lambda ,X_{0},n}^{(p,q),L}\;, \end{array}$$
  (A46) $$\begin{array}{ccc} {\tilde{B}}_{\lambda ,X_{0},n}^{\left(p,q\right)} & \leq & \exp\{x_{0}^{\left(q\right)}\cdot \left[X_{0}-\frac{p}{q}\cdot \frac{d^{(q),S}}{1-d^{(q),S}}\right]\cdot \left(1-{\left(d^{(q),S}\right)}^{n}\right)\;+\;\left(\frac{p}{q}\cdot \left(\beta_{\lambda }+x_{0}^{\left(q\right)}\right)-\alpha_{\lambda }\right)\cdot n\\ & & \;-\;{\bar{\zeta }}_{n}^{\left(q\right)}\cdot X_{0}\;-\;\frac{p}{q}\cdot {\bar{\vartheta }}_{n}^{\left(q\right)}\}\;=:\;C_{\lambda ,X_{0},n}^{(p,q),U}\;, \end{array}$$
  (A47) $$\begin{array}{cc} \mathrm{where} & {\underline{\zeta }}_{n}^{\left(q\right)}:=\Gamma_{<}^{\left(q\right)}\cdot \frac{{\left(d^{(q),T}\right)}^{n-1}}{1-d^{(q),T}}\cdot \left(1-{\left(d^{(q),T}\right)}^{n}\right)\;>\;0\;, \end{array}$$
  (A48) $$\begin{array}{ccc} {\underline{\vartheta }}_{n}^{\left(q\right)} & := & \Gamma_{<}^{\left(q\right)}\cdot \frac{1-{\left(d^{(q),T}\right)}^{n}}{{\left(1-d^{(q),T}\right)}^{2}}\cdot \left[1-\frac{d^{(q),T}\left(1+{\left(d^{(q),T}\right)}^{n}\right)}{1+d^{(q),T}}\right]\;>\;0\;, \end{array}$$
  (A49) $$\begin{array}{ccc} {\bar{\zeta }}_{n}^{\left(q\right)} & := & \Gamma_{<}^{\left(q\right)}\cdot \left[\frac{{\left(d^{(q),S}\right)}^{n}-{\left(d^{(q),T}\right)}^{n}}{d^{(q),S}-d^{(q),T}}-{\left(d^{(q),S}\right)}^{n-1}\cdot \frac{1-{\left(d^{(q),T}\right)}^{n}}{1-d^{(q),T}}\right]\;>\;0\;, \end{array}$$
  (A50) $$\begin{array}{ccc} {\bar{\vartheta }}_{n}^{\left(q\right)} & := & \Gamma_{<}^{\left(q\right)}\cdot \frac{d^{(q),T}}{1-d^{(q),T}}\cdot \left[\frac{1-{\left(d^{(q),S}d^{(q),T}\right)}^{n}}{1-d^{(q),S}d^{(q),T}}-\frac{{\left(d^{(q),S}\right)}^{n}-{\left(d^{(q),T}\right)}^{n}}{d^{(q),S}-d^{(q),T}}\right]\;>\;0\;.\; \end{array}$$
(b) *in the case p≥0 and $0<q=\beta_{\lambda }$*
  $${\tilde{B}}_{\lambda ,X_{0},n}^{\left(p,q\right)}\;=\;\exp\left\{\left(\frac{p}{q}\cdot \left(\beta_{\lambda }+x_{0}^{\left(q\right)}\right)-\alpha_{\lambda }\right)\cdot n\right\}\;=\;\exp\left\{\left(p-\alpha_{\lambda }\right)\cdot n\right\}\;.$$
(c) *in the case p≥0 and $\max\left\{0\;,\;\beta_{\lambda }\right\}<q<\min\left\{1\;,\;e^{\beta_{\lambda }-1}\right\}$ the bounds $C_{\lambda ,X_{0},n}^{(p,q),L}$ and $C_{\lambda ,X_{0},n}^{(p,q),U}$ from (96) and (97) remain valid, but with*
  (A51) $$\begin{array}{ccc} {\underline{\zeta }}_{n}^{\left(q\right)} & := & \Gamma_{>}^{\left(q\right)}\cdot \frac{{\left(d^{(q),T}\right)}^{n}-{\left(d^{(q),S}\right)}^{2n}}{d^{(q),T}-{\left(d^{(q),S}\right)}^{2}}\;>\;0\;, \end{array}$$
  (A52) $$\begin{array}{ccc} {\underline{\vartheta }}_{n}^{\left(q\right)} & := & \frac{\Gamma_{>}^{\left(q\right)}}{d^{(q),T}-{\left(d^{(q),S}\right)}^{2}}\cdot \left[\frac{d^{(q),T}\cdot \left(1-{\left(d^{(q),T}\right)}^{n}\right)}{1-d^{(q),T}}-\frac{{\left(d^{(q),S}\right)}^{2}\cdot \left(1-{\left(d^{(q),S}\right)}^{2n}\right)}{1-{\left(d^{(q),S}\right)}^{2}}\right]\;>\;0\;, \end{array}$$
  (A53) $$\begin{array}{ccc} {\bar{\zeta }}_{n}^{\left(q\right)} & := & \Gamma_{>}^{\left(q\right)}\cdot {\left(d^{(q),S}\right)}^{n-1}\cdot \left[n\;-\;\frac{1-{\left(d^{(q),T}\right)}^{n}}{1-d^{(q),T}}\right]\;>\;0\;, \end{array}$$
  (A54) $$\begin{array}{ccc} {\bar{\vartheta }}_{n}^{\left(q\right)} & := & \Gamma_{>}^{\left(q\right)}\cdot [\frac{d^{(q),S}-d^{(q),T}}{{\left(1-d^{(q),S}\right)}^{2}\left(1-d^{(q),T}\right)}\cdot \left(1-{\left(d^{(q),S}\right)}^{n}\right)\\ & & \;+\;\frac{d^{(q),T}\left(1-{\left(d^{(q),S}d^{(q),T}\right)}^{n}\right)}{\left(1-d^{(q),T}\right)\left(1-d^{(q),S}d^{(q),T}\right)}-\frac{{\left(d^{(q),S}\right)}^{n}}{1-d^{(q),S}}\cdot n]\;.\; \end{array}$$
(d) *for the special choices $p:=p_{\lambda }^{E}:=\alpha_{A}^{\lambda }\alpha_{H}^{1-\lambda }>0,\;q:=q_{\lambda }^{E}:=\beta_{A}^{\lambda }\beta_{H}^{1-\lambda }>0$ in the parameter setup $\left(\beta_{A},\beta_{H},\alpha_{A},\alpha_{H},\lambda \right)\in \left(P_{\mathrm{NI}}\cup P_{\mathrm{SP},1}\right)\times \;]\lambda_{-},\lambda_{+}[\;\backslash \left\{0,1\right\}$ we obtain*
  $$\lim_{n\to \infty }\frac{1}{n}\log\left(V_{\lambda ,X_{0},n}\right)\;=\;\lim_{n\to \infty }\frac{1}{n}\log\left(C_{\lambda ,X_{0},n}^{(p_{\lambda }^{E},q_{\lambda }^{E}),L}\right)\;=\;\lim_{n\to \infty }\frac{1}{n}\log\left(C_{\lambda ,X_{0},n}^{(p_{\lambda }^{E},q_{\lambda }^{E}),U}\right)\;=\;\frac{\alpha_{A}}{\beta_{A}}\cdot x_{0}^{\left(q_{\lambda }^{E}\right)}\;.$$
(e) *for all general p≥0 with either $0<q<\beta_{\lambda }$ or $\max\left\{0,\beta_{\lambda }\right\}<q<\min\left\{1\;,\;e^{\beta_{\lambda }-1}\right\}$ we get*
  $$\lim_{n\to \infty }\frac{1}{n}\log\left({\tilde{B}}_{\lambda ,X_{0},n}^{\left(p,q\right)}\right)\;=\;\lim_{n\to \infty }\frac{1}{n}\log\left(C_{\lambda ,X_{0},n}^{(p,q),L}\right)\;=\;\lim_{n\to \infty }\frac{1}{n}\log\left(C_{\lambda ,X_{0},n}^{(p,q),U}\right)\;=\;\frac{p}{q}\cdot \left(\beta_{\lambda }+x_{0}^{\left(q\right)}\right)-\alpha_{\lambda }\;.$$

**Proof of Lemma** **A4.** The closed-form bounds $C_{\lambda ,X_{0},n}^{(p,q),L}$ and $C_{\lambda ,X_{0},n}^{(p,q),U}$ are obtained by substituting in the representation (42) (for ${\tilde{B}}_{\lambda ,X_{0},n}^{\left(p,q\right)}$, cf. Theorem 1) the recursive sequence member $a_{n}^{\left(q\right)}$ by the explicit sequence member ${\underline{a}}_{n}^{\left(q\right)}$ respectively ${\bar{a}}_{n}^{\left(q\right)}$. From the definitions of these sequences (92) to (95) and from (83) to (85) one can see that we basically have to evaluate the term

(A55) $$\exp\left\{\left({\tilde{a}}_{n}^{hom}+{\tilde{c}}_{n}\right)\cdot X_{0}\;+\;\frac{p}{q}\cdot \sum_{k=1}^{n}\left({\tilde{a}}_{k}^{hom}+{\tilde{c}}_{k}\right)\;+\;\left(\frac{p}{q}\cdot \beta_{\lambda }-\alpha_{\lambda }\right)\cdot n\right\}\;,$$

where ${\tilde{a}}_{n}^{hom}+{\tilde{c}}_{n}={\tilde{a}}_{n}$ is either interpreted as the lower approximate ${\underline{a}}_{n}^{\left(q\right)}$ or as the upper approximate ${\bar{a}}_{n}^{\left(q\right)}$. After rearranging and incorporating that $\frac{c^{(q),S}}{1-d^{(q),S}}=\frac{c^{(q),T}}{1-d^{(q),T}}=x_{0}^{\left(q\right)}$ in both approximate cases, we obtain with the help of (86), (87) for the expression (A55) in the case 0≤ν<ϰ<d

(A56) $$\begin{array}{c} \exp\{x_{0}^{\left(q\right)}\cdot \left(1-d^{n}\right)\cdot \left[X_{0}-\frac{p}{q}\cdot \frac{d}{1-d}\right]\;+\;\left(\frac{p}{q}\cdot \left(\beta_{\lambda }+x_{0}^{\left(q\right)}\right)-\alpha_{\lambda }\right)\cdot n\\ \;+\;\left[K_{1}\cdot \frac{d^{n}-\varkappa^{n}}{d-\varkappa }\;+\;K_{2}\cdot \frac{d^{n}-\nu^{n}}{d-\nu }\right]\cdot X_{0}\\ \;+\;\frac{p}{q}\cdot \left[\left(\frac{K_{1}}{d-\varkappa }+\frac{K_{2}}{d-\nu }\right)\cdot \frac{d\cdot \left(1-d^{n}\right)}{1-d}-\frac{K_{1}\cdot \varkappa \cdot \left(1-\varkappa^{n}\right)}{\left(d-\varkappa )(1-\varkappa \right)}-\frac{K_{2}\cdot \nu \cdot \left(1-\nu^{n}\right)}{\left(d-\nu )(1-\nu \right)}\right]\}.\;\; \end{array}$$

In the other case 0≤ν<ϰ=d, the application of (88), (89) turns (A55) into

(A57) $$\begin{array}{c} \exp\{x_{0}^{\left(q\right)}\cdot \left(1-d^{n}\right)\cdot \left[X_{0}-\frac{p}{q}\cdot \frac{d}{1-d}\right]\;+\;\left(\frac{p}{q}\cdot \left(\beta_{\lambda }+x_{0}^{\left(q\right)}\right)-\alpha_{\lambda }\right)\cdot n\\ \;+\;\left[K_{1}\cdot n\cdot d^{n-1}+K_{2}\cdot \frac{d^{n}-\nu^{n}}{d-\nu }\right]\cdot X_{0}\\ \;+\;\frac{p}{q}\cdot \left[\left(\frac{K_{1}}{d(1-d)}+\frac{K_{2}}{d-\nu }\right)\cdot \frac{d\cdot \left(1-d^{n}\right)}{1-d}-\frac{K_{2}\cdot \nu \cdot \left(1-\nu^{n}\right)}{\left(d-\nu )(1-\nu \right)}-\frac{K_{1}\cdot d^{n}}{1-d}\cdot n\right]\}.\;\; \end{array}$$

After these preparatory considerations let us now begin with elaboration of the details. (a) Let $0<q<\beta_{\lambda }$. We obtain a closed-form lower bound for ${\tilde{B}}_{\lambda ,X_{0},n}^{\left(p,q\right)}$ by employing the parameters $c\hat{=}c^{(q),T}$, $d\hat{=}d^{(q),T}$, $K_{2}=\nu =0$, $K_{1}=\Gamma_{<}^{\left(q\right)},$ and $\varkappa ={\left(d^{(q),T}\right)}^{2}$, cf. (93) in combination with (85). Since $\varkappa <d^{(q),T}$, we have to plug in these parameters into (A56). The representations of ${\underline{\zeta }}_{n}^{\left(q\right)}$ and ${\underline{\vartheta }}_{n}^{\left(q\right)}$ in (A47) and (A48) follow immediately. For a closed-form upper bound, we employ the parameters $c\hat{=}c^{(q),S}$, $d\hat{=}d^{(q),S}$, $-K_{1}=K_{2}=\Gamma_{<}^{\left(q\right)}$, $\varkappa =d^{(q),T}$ and $\nu =d^{(q),S}d^{(q),T}$ (in particular, $\varkappa <d^{(q),S}$ implying that we have to use (A56)). From this, (A49) can be deduced directly; the representation (A50) comes from the expressions in the squared brackets in the last line of (A56) and from

$$\begin{array}{c} -\left(\frac{\Gamma_{<}^{\left(q\right)}}{d^{(q),S}-d^{(q),T}}-\frac{\Gamma_{<}^{\left(q\right)}}{d^{(q),S}-d^{(q),S}d^{(q),T}}\right)\cdot \frac{d^{(q),S}\cdot \left(1-{\left(d^{(q),S}\right)}^{n}\right)}{1-d^{(q),S}}+\frac{\Gamma_{<}^{\left(q\right)}\cdot d^{(q),T}\cdot \left(1-{\left(d^{(q),T}\right)}^{n}\right)}{\left(d^{(q),S}-d^{(q),T}\right)\left(1-d^{(q),T}\right)}\\ \;-\;\frac{\Gamma_{<}^{\left(q\right)}\cdot d^{(q),S}d^{(q),T}\cdot \left(1-{\left(d^{(q),S}d^{(q),T}\right)}^{n}\right)}{\left(d^{(q),S}-d^{(q),S}d^{(q),T}\right)\left(1-d^{(q),S}d^{(q),T}\right)}\\ =\;-\;\frac{\Gamma_{<}^{\left(q\right)}\cdot d^{(q),T}\left(1-d^{(q),S}\right)}{d^{(q),S}\left(d^{(q),S}-d^{(q),T}\right)\left(1-d^{(q),T}\right)}\cdot \frac{d^{(q),S}\cdot \left(1-{\left(d^{(q),S}\right)}^{n}\right)}{1-d^{(q),S}}+\frac{\Gamma_{<}^{\left(q\right)}\cdot d^{(q),T}\cdot \left(1-{\left(d^{(q),T}\right)}^{n}\right)}{\left(d^{(q),S}-d^{(q),T}\right)\left(1-d^{(q),T}\right)}\\ \;-\;\frac{\Gamma_{<}^{\left(q\right)}\cdot d^{(q),T}\cdot \left(1-{\left(d^{(q),S}d^{(q),T}\right)}^{n}\right)}{\left(1-d^{(q),T}\right)\left(1-d^{(q),S}d^{(q),T}\right)}\\ =\;-\;\frac{\Gamma_{<}^{\left(q\right)}\cdot d^{(q),T}}{1-d^{(q),T}}\cdot \left[\frac{1-{\left(d^{(q),S}d^{(q),T}\right)}^{n}}{1-d^{(q),S}d^{(q),T}}+\frac{1-{\left(d^{(q),S}\right)}^{n}}{d^{(q),S}-d^{(q),T}}-\frac{1-{\left(d^{(q),T}\right)}^{n}}{d^{(q),S}-d^{(q),T}}\right]\\ =\;-\;\frac{\Gamma_{<}^{\left(q\right)}\cdot d^{(q),T}}{1-d^{(q),T}}\cdot \left[\frac{1-{\left(d^{(q),S}d^{(q),T}\right)}^{n}}{1-d^{(q),S}d^{(q),T}}-\frac{{\left(d^{(q),S}\right)}^{n}-{\left(d^{(q),T}\right)}^{n}}{d^{(q),S}-d^{(q),T}}\right]\;=\;-{\bar{\vartheta }}_{n}^{\left(q\right)}\;.\; \end{array}$$

Part (b) has already been mentioned in Remark 1 (b) and is due to the fact that for $0<q=\beta_{\lambda }$, the sequence ${\left(a_{n}^{\left(q\right)}\right)}_{n\in N}$ is itself explicitly representable by $a_{n}^{\left(q\right)}=0$ for all n∈N (cf. Properties 1 (P2)). Plugging this into (42) gives the desired result. (c) Let us now consider $\max\left\{0,\beta_{\lambda }\right\}<q<\min\left\{1,e^{\beta_{\lambda }-1}\right\}$. For a closed-form lower bound for ${\tilde{B}}_{\lambda ,X_{0},n}^{\left(p,q\right)}$ we have to employ the parameters $c\hat{=}c^{(q),T}$, $d\hat{=}d^{(q),T}$, $K_{2}=\nu =0$, $K_{1}=\Gamma_{>}^{\left(q\right)}$ and $\varkappa ={\left(d^{(q),S}\right)}^{2}$, cf. (93) in combination with (85). The representations of ${\underline{\zeta }}_{n}^{\left(q\right)}$ and ${\underline{\vartheta }}_{n}^{\left(q\right)}$ in (A51) and (A52) follow immediately from (A56). For a closed-form upper bound, we use the parameters $c\hat{=}c^{(q),S}$, $d\hat{=}d^{(q),S}$, $-K_{1}=K_{2}=\Gamma_{>}^{\left(q\right)}$, $\varkappa =d^{(q),S}$ and $\nu =d^{(q),S}d^{(q),T}$. Notice that in this case we stick to the representation (A57). The formula (104) is obviously valid, and (105) is implied by

$$\begin{array}{cc} & \left(\frac{-\Gamma_{>}^{\left(q\right)}}{d^{(q),S}\left(1-d^{(q),S}\right)}+\frac{\Gamma_{>}^{\left(q\right)}}{d^{(q),S}-d^{(q),S}d^{(q),T}}\right)\cdot \frac{d^{(q),S}\cdot \left(1-{\left(d^{(q),S}\right)}^{n}\right)}{1-d^{(q),S}}\\ = & -\;\Gamma_{>}^{\left(q\right)}\cdot \frac{d^{(q),S}-d^{(q),T}}{{\left(1-d^{(q),S}\right)}^{2}\left(1-d^{(q),T}\right)}\cdot \left(1-{\left(d^{(q),S}\right)}^{n}\right)\;. \end{array}$$

The parts (d) and (e) are trivial by incorporating that in all respective cases one has $d^{(q),S}\in ]0,1[$, $d^{(q),T}\in ]0,1[$ and $\lim_{n\to \infty }n\cdot d^{(q),S}=0$. □

**Proof of Theorem** **5.** (a) For λ∈]0,1[, we get $0<q_{\lambda }^{E}<\beta_{\lambda }$ and the assertion follows by applying part (a) of Lemma A4. Notice that in the current subcase $P_{\mathrm{NI}}\cup P_{\mathrm{SP},1}$ there holds $\frac{p_{\lambda }^{E}}{q_{\lambda }^{E}}\beta_{\lambda }-\alpha_{\lambda }=0$ as well as $\frac{p_{\lambda }^{E}}{q_{\lambda }^{E}}=\frac{\alpha_{A}}{\beta_{A}}=\frac{\alpha_{H}}{\beta_{H}}$. For the case λ∈R\[0,1], one gets from Lemma A1 that $\max\left\{0,\beta_{\lambda }\right\}<q_{\lambda }^{E}$, and there holds $q_{\lambda }^{E}<\min\left\{1,e^{\beta_{\lambda }-1}\right\}$ iff $\lambda \in ]\lambda_{-},\lambda_{+}[\;\backslash \left[0,1\right]$, cf. Lemma 1. Thus, an application of part (c) of Lemma A4 proves the desired result. The assertion (b) is equivalent to part (d) of Lemma A4. □

**Proof of Theorem** **6.** The assertions follow immediately from (A45), Lemma A4(b),(e), Proposition 6(d) as well as the incorporation of the fact that for λ∈]0,1[ there holds $q_{\lambda }^{L}=\beta_{A}^{\lambda }\beta_{H}^{1-\lambda }<\beta_{\lambda }$ in the case $\left(\beta_{A},\beta_{H},\alpha_{A},\alpha_{H}\right)\in \left(P_{\mathrm{SP}}\backslash \left(P_{\mathrm{SP},1}\cup P_{\mathrm{SP},4}\right)\right)$ (i.e., $\beta_{A}\neq \beta_{H}$) respectively $q_{\lambda }^{L}=\beta_{\lambda }$ in the case $\left(\beta_{A},\beta_{H},\alpha_{A},\alpha_{H}\right)\in P_{\mathrm{SP},4}$ (i.e., $\beta_{A}=\beta_{H}$). □

**Proof of Theorem** **7.** This can be deduced from (A46), from the parts (b), (c) and (e) of Lemma A4 as well as the incorporation of $p_{\lambda }^{U}\geq \alpha_{A}^{\lambda }\alpha_{H}^{1-\lambda }>0$ for λ∈]0,1[. Notice that an inadequate choice of $p_{\lambda }^{U},\;q_{\lambda }^{U}$ may lead to $\frac{p_{\lambda }^{U}}{q_{\lambda }^{U}}\left(\beta_{\lambda }+x_{0}^{\left(q_{\lambda }^{U}\right)}\right)-\alpha_{\lambda }>0$. □

**Proof of Theorem** **8.** The assertions follow immediately from (A45) and from the parts (b), (c) and (e) of Lemma A4. Notice that an inadequate choice of $p_{\lambda }^{L},\;q_{\lambda }^{L}$ may lead to $\frac{p_{\lambda }^{L}}{q_{\lambda }^{L}}\left(\beta_{\lambda }+x_{0}^{\left(q_{\lambda }^{U}\right)}\right)-\alpha_{\lambda }<0$. □

**Proof of Theorem** **9.** Let $p_{\lambda }^{U}=\alpha_{A}^{\lambda }\alpha_{H}^{1-\lambda }>\max\left\{0,\alpha_{\lambda }\right\}$ and $q_{\lambda }^{U}=\beta_{A}^{\lambda }\beta_{H}^{1-\lambda }>\max\left\{0,\beta_{\lambda }\right\}$. Since $q_{\lambda }^{U}<\min\left\{1,e^{\beta_{\lambda }-1}\right\}$ iff $\lambda \in ]\lambda_{-},\lambda_{+}[\;\backslash \left[0,1\right]$ (cf. Lemma 1 for $q_{\lambda }:=q_{\lambda }^{U})$), this theorem follows from (A46) of Lemma A4, from the parts (b), (e) of Lemma A4 and from part (d) of Proposition 14. □

### Appendix A.4. Proofs and Auxiliary Lemmas for Section 7

**Proof of Theorem** **10.** As already mentioned above, one can adapt the proof of Theorem 9.1.3 in Ethier & Kurtz [138] who deal with drift-parameters η=0, $\kappa_{•}=0$, and the different setup of *σ–independent time-scale* and a sequence of *critical* Galton-Watson processes *without immigration* with *general* offspring distribution. For the sake of brevity, we basically outline here only the main differences to their proof; for similar limit investigations involving offspring/immigration distributions and parametrizations which are incompatble to ours, see e.g., Sriram [142]. As a first step, let us define the generator

$$A_{•}f\left(x\right)\;:=\;\left(\eta -\kappa_{•}\cdot x\right)\cdot f^{\prime }\left(x\right)+\frac{\sigma^{2}}{2}\cdot x\cdot f^{\prime \prime }\left(x\right),\;f\in C_{c}^{\infty }\left([0,\infty )\right)\;,$$

which corresponds to the diffusion process $\tilde{X}$ governed by (133). In connection with (130), we study

$$T_{•}^{\left(m\right)}f\left(x\right)\;:=\;EP_{•}\left[f\left(\frac{1}{m}\left(\sum_{k=1}^{mx}Y_{0,k}^{\left(m\right)}+{\tilde{Y}}_{0}^{\left(m\right)}\right)\right)\right],\;x\in E^{\left(m\right)}:=\frac{1}{m}N_{0},\;f\in C_{c}^{\infty }(\left[0,\infty \right),$$

where the $Y_{0,k}^{\left(m\right)}$, ${\tilde{Y}}_{0}^{\left(m\right)}$ are independent and (Poisson-$\beta_{•}^{\left(m\right)}$ respectively Poisson-$\alpha_{•}^{\left(m\right)}$) distributed as the members of the collection $Y^{\left(m\right)}$ respectively ${\tilde{Y}}^{\left(m\right)}$. By the Theorems 8.2.1 and 1.6.5 as well as Corollary 4.8.9 of [138] it is sufficient to show

(A58) $$\lim_{m\to \infty }\underset{x\in E^{\left(m\right)}}{\sup}\left|\sigma^{2}m\left(T_{•}^{\left(m\right)}f\left(x\right)-f\left(x\right)\right)-A_{•}f\left(x\right)\right|\;=\;0,\;\;f\in C_{c}^{\infty }\left([0,\infty )\right)\;.$$

But (A58) follows mainly from the next

**Lemma** **A5.**
*Let*

$$S_{n}^{\left(m\right)}\;:=\;\frac{1}{\sqrt{n}}\left(\sum_{k=1}^{n}\left(Y_{0,k}^{\left(m\right)}-\beta_{•}^{\left(m\right)}\right)+{\tilde{Y}}_{0}^{\left(m\right)}-\alpha_{•}^{\left(m\right)}\right)\;,\;n\in N,\;m\in \bar{N},$$

*with the usual convention $S_{0}^{\left(m\right)}:=0$. Then for all $m\in \bar{N}$, $x\in E^{\left(m\right)}$ and all $f\in C_{c}^{\infty }\left([0,\infty )\right)$*

(A59) $$\begin{array}{c} \epsilon^{\left(m\right)}\left(x\right)\;:=\;EP_{•}\left[\int_{0}^{1}{\left(S_{mx}^{\left(m\right)}\right)}^{2}x\left(1-v\right)\left(f^{\prime \prime }\left(\beta_{•}^{\left(m\right)}x+\frac{\alpha_{•}^{\left(m\right)}}{m}+v\sqrt{\frac{x}{m}}S_{mx}^{\left(m\right)}\right)-f^{\prime \prime }\left(x\right)\right)dv\right]\\ =\;\frac{1}{\sigma^{2}}\cdot \left[\sigma^{2}m\cdot \left(T_{•}^{\left(m\right)}f\left(x\right)-f\left(x\right)\right)-A_{•}f\left(x\right)\right]\;+\;R^{\left(m\right)},\;\mathrm{where}\;\lim_{m\to \infty }R^{\left(m\right)}=0. \end{array}$$

**Proof of Lemma** **A5.** Let us fix $f\in C_{c}^{\infty }\left([0,\infty )\right)$. From the involved Poissonian expectations it is easy to see that

$$\lim_{m\to \infty }\left|\sigma^{2}m\left(T_{•}^{\left(m\right)}f\left(0\right)-f\left(0\right)\right)-A_{•}f\left(0\right)\right|\;=\;0\;,$$

and thus (A49) holds for x=0. Accordingly, we next consider the case $x\in E^{\left(m\right)}\backslash \left\{0\right\}$, with fixed $m\in \bar{N}$. From $EP_{•}\left[{\left(S_{mx}^{\left(m\right)}\right)}^{2}\right]=\beta_{•}^{\left(m\right)}+\frac{\alpha_{•}^{\left(m\right)}}{mx}$ we obtain

(A60) $$EP_{•}\left[{\left(S_{mx}^{\left(m\right)}\right)}^{2}xf^{\prime \prime }\left(x\right)\int_{0}^{1}\left(1-v\right)dv\right]\;=\;\frac{1}{2}\left(\beta_{•}^{\left(m\right)}\cdot x+\frac{\alpha_{•}^{\left(m\right)}}{m}\right)f^{\prime \prime }\left(x\right)\;=:\;a_{mx}\;\frac{f^{\prime \prime }\left(x\right)}{2}\;=:\;a\;\frac{f^{\prime \prime }\left(x\right)}{2}\;.$$

Furthermore, with $\;b_{mx}:=\;b:=a+\sqrt{x/m}\cdot S_{mx}^{\left(m\right)}=\frac{1}{m}\left(\sum_{k=1}^{mx}Y_{0,k}^{\left(m\right)}+{\tilde{Y}}_{0}^{\left(m\right)}\right)$ we get on $\left\{S_{mx}^{\left(m\right)}\neq 0\right\}$

(A61) $$\int_{0}^{1}f^{\prime \prime }\left(\beta_{•}^{\left(m\right)}x+\frac{\alpha_{•}^{\left(m\right)}}{m}+v\sqrt{\frac{x}{m}}\;S_{mx}^{\left(m\right)}\right)dv\;=\;\sqrt{\frac{m}{x}}\cdot \frac{1}{S_{mx}^{\left(m\right)}}\int_{a}^{b}f^{\prime \prime }\left(y\right)dy\;=\;\sqrt{\frac{m}{x}}\cdot \frac{f^{\prime }\left(b\right)-f^{\prime }\left(a\right)}{S_{mx}^{\left(m\right)}}$$

as well as

(A62) $$\begin{array}{c} \int_{0}^{1}vf^{\prime \prime }\left(\beta_{•}^{\left(m\right)}x+\frac{\alpha_{•}^{\left(m\right)}}{m}+v\sqrt{\frac{x}{m}}\;S_{mx}^{\left(m\right)}\right)dv\;=\;\frac{m}{x{\left(S_{mx}^{\left(m\right)}\right)}^{2}}\left[\int_{a}^{b}yf^{\prime \prime }\left(y\right)\;dy-a\int_{a}^{b}f^{\prime \prime }\left(y\right)\;dy\right]\\ =\;\sqrt{\frac{m}{x}}\cdot \frac{f^{\prime }\left(b\right)}{S_{mx}^{\left(m\right)}}\;+\;\frac{m}{x}\cdot \frac{f(a)-f(b)}{{\left(S_{mx}^{\left(m\right)}\right)}^{2}}\;. \end{array}$$

With our choice $\beta_{•}^{\left(m\right)}=1-\frac{\kappa_{•}}{\sigma^{2}m}$ and $\alpha_{•}^{\left(m\right)}=\beta_{•}^{\left(m\right)}\cdot \frac{\eta }{\sigma^{2}}$, a Taylor expansion of *f* at *x* gives

(A63) $$\begin{array}{c} f\left(a\right)\;=\;f\left(x\right)\;+\;\frac{1}{\sigma^{2}m}\cdot f^{\prime }\left(x\right)\left(\beta_{•}^{\left(m\right)}\cdot \eta -\kappa_{•}\cdot x\right)\;+\;o\left(\frac{1}{m}\right), \end{array}$$

where for the case η=κ=0 we use the convention $o\left(\frac{1}{m}\right)\equiv 0$. Combining (A60) to (A63) and the centering $EP_{•}\left[S_{mx}^{\left(m\right)}\right]=0$, the left hand side of Equation (A59) becomes

$$\begin{array}{cc} & EP_{•}\left[\int_{0}^{1}{\left(S_{mx}^{\left(m\right)}\right)}^{2}x\left(1-v\right)\left(f^{\prime \prime }\left(\beta_{•}^{\left(m\right)}x+\frac{\alpha_{•}^{\left(m\right)}}{m}+v\sqrt{\frac{x}{m}}\;S_{mx}^{\left(m\right)}\right)-f^{\prime \prime }\left(x\right)\right)dv\right]\\ = & EP_{•}\left[\sqrt{mx}\cdot S_{mx}^{\left(m\right)}\cdot \left(f^{\prime }\left(b\right)-f^{\prime }\left(a\right)\right)\right]\;-\;EP_{•}\left[\sqrt{mx}\cdot S_{mx}^{\left(m\right)}\cdot f^{\prime }\left(b\right)+m\cdot \left(f\left(a\right)-f\left(b\right)\right)\right]\\ & -\;\frac{1}{2}\left(\beta_{•}^{\left(m\right)}\cdot x+\frac{\alpha_{•}^{\left(m\right)}}{m}\right)\cdot f^{\prime \prime }\left(x\right)\\ = & m\cdot \left(EP_{•}\left[f(b)\right]-f\left(a\right)\right)\;-\;\frac{1}{2}\left(\beta_{•}^{\left(m\right)}\cdot x+\frac{\alpha_{•}^{\left(m\right)}}{m}\right)\cdot f^{\prime \prime }\left(x\right)\\ = & m\cdot \left\{EP_{•}\left[f\left(\frac{1}{m}\left(\sum_{k=1}^{mx}Y_{0,k}^{\left(m\right)}+{\tilde{Y}}_{0}\right)\right)\right]-f\left(x\right)\right\}-\frac{1}{\sigma^{2}}A_{•}f\left(x\right)\\ & +\frac{1}{\sigma^{2}}\left[\left(\eta -\kappa_{•}\cdot x\right)-\beta_{•}^{\left(m\right)}\cdot \eta +\kappa_{•}\cdot x\right]\cdot f^{\prime }\left(x\right)+\frac{x}{2}\left[1-\beta_{•}^{\left(m\right)}-\frac{\alpha_{•}^{\left(m\right)}}{m}\right]\cdot f^{\prime \prime }\left(x\right)-m\cdot o\left(\frac{1}{m}\right) \end{array}$$

which immediately leads to the right hand side of (A59). □

To proceed with the proof of Theorem 10, we obtain for $m\geq 2\kappa_{•}/\sigma^{2}$ the inequality $\beta_{•}^{\left(m\right)}\geq 1/2$ and accordingly for all v∈]0,1[, $x\in E^{\left(m\right)}$

$$\beta_{•}^{\left(m\right)}x+\frac{\alpha_{•}^{\left(m\right)}}{m}+v\sqrt{\frac{x}{m}}\;S_{mx}^{\left(m\right)}=\left(1-v\right)\cdot x\cdot \beta_{•}^{\left(m\right)}+\left(1-v\right)\frac{\alpha_{•}^{\left(m\right)}}{m}+v\left(\sum_{k=1}^{mx}Y_{0,k}^{\left(m\right)}+{\tilde{Y}}_{0}\right)\;\geq \;x\cdot \frac{1-v}{2}\;.$$

Suppose that the support of *f* is contained in the interval [0,c]. Correspondingly, for v≤1−2c/x the integrand in $\epsilon^{\left(m\right)}\left(x\right)$ is zero and hence with (A64) we obtain the bounds

$$\begin{array}{cc} & \left|\int_{0}^{1}{\left(S_{mx}^{\left(m\right)}\right)}^{2}x\left(1-v\right)\left(f^{\prime \prime }\left(\beta_{•}^{\left(m\right)}x+\frac{\alpha_{•}^{\left(m\right)}}{m}+v\sqrt{\frac{x}{m}}\;S_{mx}^{\left(m\right)}\right)-f^{\prime \prime }\left(x\right)\right)dv\right|\\ \leq & \int_{0\vee (1-2c/x)}^{1}{\left(S_{mx}^{\left(m\right)}\right)}^{2}x\left(1-v\right)\cdot 2{∥f^{\prime \prime }∥}_{\infty }dv\;\leq \;x\cdot {\left(S_{mx}^{\left(m\right)}\right)}^{2}{\left(1\wedge \frac{2c}{x}\right)}^{2}{∥f^{\prime \prime }∥}_{\infty }. \end{array}$$

From this, one can deduce $\lim_{m\to \infty }\sup_{x\in E^{\left(m\right)}}\epsilon^{\left(m\right)}\left(x\right)=0$–and thus (A58) – in the same manner as at the end of the proof of Theorem 9.1.3 in [138] (by means of the dominated convergence theorem).

**Proof of Proposition** **15.** Let $\left(\kappa_{A},\kappa_{H},\eta \right)\in {\tilde{P}}_{NI}\cup {\tilde{P}}_{SP,1}$ be fixed. We have to find those orders λ∈R\[0,1] which satisfy for all sufficiently large $m\in \bar{N}$

(A64) $$q_{\lambda }^{\left(m\right)}\;=\;{\left(1-\frac{\kappa_{A}}{\sigma^{2}m}\right)}^{\lambda }{\left(1-\frac{\kappa_{H}}{\sigma^{2}m}\right)}^{1-\lambda }\;<\;\min\left\{1\;,\;e^{\beta_{\lambda }^{\left(m\right)}-1}\right\}.$$

In order to achieve this, we interpret $q_{\lambda }^{\left(m\right)}=q_{\lambda }\left(\frac{1}{m}\right)$ in terms of the function

(A65) $$q_{\lambda }\left(x\right)\;:=\;{\left(1-\frac{\kappa_{A}}{\sigma^{2}}\cdot x\right)}^{\lambda }{\left(1-\frac{\kappa_{H}}{\sigma^{2}}\cdot x\right)}^{1-\lambda }\;,\;x\in ]-\epsilon ,\epsilon [\;,$$

for some small enough ϵ>0 such that (A65) is well-defined. Since $\beta_{\lambda }^{\left(m\right)}-1=-\frac{\kappa_{\lambda }}{\sigma^{2}\cdot m}=-\frac{\kappa_{\lambda }}{\sigma^{2}}\cdot x=-\frac{\lambda \kappa_{A}+\left(1-\lambda \right)\kappa_{H}}{\sigma^{2}}\cdot x$, for the verification of (A64) it suffices to show

(A66) $$\begin{array}{ccc} \lim_{x↘0}\frac{1-q_{\lambda }\left(x\right)}{x} & > & 0\;, \end{array}$$

(A67) $$\begin{array}{ccc} \mathrm{and}\;\lim_{x↘0}\frac{e^{-\frac{\kappa_{\lambda }}{\sigma^{2}}\cdot x}-q_{\lambda }\left(x\right)}{x^{2}} & > & 0\;. \end{array}$$

By l’Hospital’s rule, one gets $\lim_{x↘0}\frac{1-q_{\lambda }\left(x\right)}{x}\;=\;\frac{\lambda \kappa_{A}+\left(1-\lambda \right)\kappa_{H}}{\sigma^{2}}\;=\;\frac{\kappa_{\lambda }}{\sigma^{2}}$ and hence

(A68) $$\begin{array}{ccc} \left(\text{A66}\right) & ⟺ & \left\{\begin{array}{ccc} \lambda \;<\;\frac{\kappa_{H}}{\kappa_{H}-\kappa_{A}}, & \mathrm{if} & \kappa_{A}\;<\;\kappa_{H}\;,\\ \lambda \;>\;-\;\frac{\kappa_{H}}{\kappa_{A}-\kappa_{H}}, & \mathrm{if} & \kappa_{A}\;>\;\kappa_{H}\;. \end{array}\right. \end{array}$$

To find a condition that guarantees (A67), we use l’Hospital’s rule twice to deduce

$$\lim_{x↘0}\frac{e^{-\frac{\kappa_{\lambda }}{\sigma^{2}}\cdot x}-q_{\lambda }\left(x\right)}{x^{2}}\;=\;\frac{1}{2\sigma^{4}}\left[\kappa_{\lambda }^{2}-\lambda \left(\lambda -1\right){\left(\kappa_{A}-\kappa_{H}\right)}^{2}\right]\;=\;\frac{1}{2\sigma^{4}}\left[\lambda \kappa_{A}^{2}+\left(1-\lambda \right)\kappa_{H}^{2}\right]\;$$

and hence we obtain

(A69) $$\begin{array}{ccc} \left(\text{A67}\right) & ⟺ & \left\{\begin{array}{ccc} \lambda \;<\;\frac{\kappa_{H}^{2}}{\kappa_{H}^{2}-\kappa_{A}^{2}}, & \mathrm{if} & \kappa_{A}\;<\;\kappa_{H},\\ \lambda \;>\;-\;\frac{\kappa_{H}^{2}}{\kappa_{A}^{2}-\kappa_{H}^{2}}, & \mathrm{if} & \kappa_{A}\;>\;\kappa_{H}. \end{array}\right. \end{array}$$

To compare both the lower and upper bounds in (A68) and (A69), let us calculate

(A70) $$\frac{\kappa_{H}^{2}}{\kappa_{H}^{2}-\kappa_{A}^{2}}-\frac{\kappa_{H}}{\kappa_{H}-\kappa_{A}}\;=\;-\;\frac{\kappa_{A}\kappa_{H}}{\left(\kappa_{H}-\kappa_{A}\right)\left(\kappa_{H}+\kappa_{A}\right)}\;\left\{\begin{array}{ccc} <\;0, & \mathrm{if} & \kappa_{A}\;<\;\kappa_{H},\\ >\;0, & \mathrm{if} & \kappa_{A}\;>\;\kappa_{H}. \end{array}\right.$$

Incorporating this, we observe that both conditions (A66) and (A67) are satisfied simultaneously iff

$$\begin{array}{ccc} \lambda & < & \min\left\{\frac{\kappa_{H}}{\kappa_{H}-\kappa_{A}}\;,\;\frac{\kappa_{H}^{2}}{\kappa_{H}^{2}-\kappa_{A}^{2}}\right\}\;\;=\;\;\frac{\kappa_{H}^{2}}{\kappa_{H}^{2}-\kappa_{A}^{2}}\;\mathrm{if}\;\kappa_{A}\;<\;\kappa_{H},\\ \lambda & > & \max\left\{-\frac{\kappa_{H}}{\kappa_{A}-\kappa_{H}}\;,\;-\frac{\kappa_{H}^{2}}{\kappa_{A}^{2}-\kappa_{H}^{2}}\right\}\;=\;-\frac{\kappa_{H}^{2}}{\kappa_{A}^{2}-\kappa_{H}^{2}}\;\mathrm{if}\;\kappa_{A}\;>\;\kappa_{H}, \end{array}$$

which finishes the proof. □

The following lemma is the main tool for the proof of Theorem 11.

**Lemma** **A6.**
*Let $\left(\kappa_{A},\kappa_{H},\eta ,\lambda \right)\in \left({\tilde{P}}_{NI}\cup {\tilde{P}}_{SP,1}\right)\times \left(\;\right]{\tilde{\lambda }}_{-},{\tilde{\lambda }}_{+}\left[\;\backslash \{0,1\}\;\right)$. By using the quantities $\kappa_{\lambda }:=\lambda \kappa_{A}+\left(1-\lambda \right)\kappa_{H}$ and $\Lambda_{\;\lambda }:=\sqrt{\lambda \kappa_{A}^{2}+\left(1-\lambda \right)\kappa_{H}^{2}}$ from (150) (which is well-defined, cf. (138)), one gets for all t>0*

$$\begin{array}{cc} \left(a\right) & \;\lim_{m\to \infty }m\cdot \left(1-q_{\lambda }^{\left(m\right)}\right)\;=\;\lim_{m\to \infty }m\cdot \left(1-\beta_{\lambda }^{\left(m\right)}\right)\;=\;\frac{\kappa_{\lambda }}{\sigma^{2}}\;.\\ \left(b\right) & \;\lim_{m\to \infty }m^{2}\cdot a_{1}^{\left(m\right)}\;=\;\lim_{m\to \infty }m^{2}\cdot \left(q_{\lambda }^{\left(m\right)}-\beta_{\lambda }^{\left(m\right)}\right)\;=\;-\frac{\lambda \left(1-\lambda \right){\left(\kappa_{A}-\kappa_{H}\right)}^{2}}{2\sigma^{4}}\;=\;-\frac{\Lambda_{\;\lambda }^{2}-\kappa_{\lambda }^{2}}{2\sigma^{4}}\;.\;\\ \left(c\right) & \;\lim_{m\to \infty }m\cdot x_{0}^{\left(m\right)}\;=\;-\frac{\Lambda_{\;\lambda }-\kappa_{\lambda }}{\sigma^{2}}\;\left\{\begin{array}{cc} <0, & \mathrm{if}\;\lambda \in ]0,1[,\\ \;\\ >0, & \mathrm{if}\;\lambda \in ]{\tilde{\lambda }}_{-},{\tilde{\lambda }}_{+}[\backslash \left[0,1\right]. \end{array}\right.\\ \left(d\right) & \;\lim_{m\to \infty }m^{2}\cdot \Gamma_{<}^{\left(m\right)}\;=\;\lim_{m\to \infty }m^{2}\cdot \Gamma_{>}^{\left(m\right)}\;=\;\frac{{\left(\Lambda_{\;\lambda }-\kappa_{\lambda }\right)}^{2}}{2\sigma^{4}}\;>\;0\;.\\ \left(e\right) & \;\lim_{m\to \infty }m\cdot \left(1-d^{(m),S}\right)\;=\;\frac{\Lambda_{\;\lambda }+\kappa_{\lambda }}{2\sigma^{2}}\;>\;0\;.\\ \left(f\right) & \;\lim_{m\to \infty }m\cdot \left(1-d^{(m),T}\right)\;=\;\frac{\Lambda_{\;\lambda }}{\sigma^{2}}\;>\;0\;\;.\\ \left(g\right) & \;\lim_{m\to \infty }m\cdot \left(1-d^{(m),S}d^{(m),T}\right)\;=\;\frac{3\Lambda_{\;\lambda }+\kappa_{\lambda }}{2\sigma^{2}}\;>\;0\;.\\ \left(h\right) & \;\lim_{m\to \infty }{\left(d^{(m),S}\right)}^{\sigma^{2}mt}\;=\;\exp\left\{-\frac{\Lambda_{\;\lambda }+\kappa_{\lambda }}{2}\cdot t\right\}\;<\;1\;.\\ \left(i\right) & \;\lim_{m\to \infty }{\left(d^{(m),T}\right)}^{\sigma^{2}mt}\;=\;\exp\left\{-\Lambda_{\;\lambda }\cdot t\right\}\;<\;1\;.\\ \left(j\right) & \;\lim_{m\to \infty }{\left(d^{(m),S}d^{(m),T}\right)}^{\sigma^{2}mt}\;=\;\exp\left\{-\frac{3\Lambda_{\;\lambda }+\kappa_{\lambda }}{2}\cdot t\right\}\;<\;1\;.\\ \left(k\right) & \;\mathrm{for}\;\lambda \in ]0,1[,\;\mathrm{there}\;\mathrm{holds}\;\mathrm{for}\;\mathrm{the}\;\mathrm{respective}\;\mathrm{quantities}\;\mathrm{defined}\;\mathrm{in}\;(142)\;\mathrm{to}\;(145)\\ & \;\lim_{m\to \infty }m\cdot {\underline{\zeta }}_{\left\lfloor \sigma^{2}mt\right\rfloor }^{\left(m\right)}\;=\;\frac{{\left(\Lambda_{\;\lambda }-\kappa_{\lambda }\right)}^{2}}{2\sigma^{2}\cdot \Lambda_{\;\lambda }}\cdot e^{-\Lambda_{\;\lambda }\cdot t}\cdot \left(1-e^{-\Lambda_{\;\lambda }\cdot t}\right)\;>\;0\;,\\ & \;\lim_{m\to \infty }{\underline{\vartheta }}_{\left\lfloor \sigma^{2}mt\right\rfloor }^{\left(m\right)}\;=\;\frac{1}{4}\cdot {\left(\frac{\Lambda_{\;\lambda }-\kappa_{\lambda }}{\Lambda_{\;\lambda }}\right)}^{2}\cdot {\left(1-e^{-\Lambda_{\;\lambda }\cdot t}\right)}^{2}\;>\;0\;,\\ & \;\lim_{m\to \infty }m\cdot {\bar{\zeta }}_{\left\lfloor \sigma^{2}mt\right\rfloor }^{\left(m\right)}\;=\;\frac{{\left(\Lambda_{\;\lambda }-\kappa_{\lambda }\right)}^{2}}{\sigma^{2}}\cdot \left[\frac{e^{-\frac{1}{2}\left(\Lambda_{\;\lambda }+\kappa_{\lambda }\right)\cdot t}-e^{-\Lambda_{\;\lambda }\cdot t}}{\Lambda_{\;\lambda }-\kappa_{\lambda }}-\frac{e^{-\frac{1}{2}\left(\Lambda_{\;\lambda }+\kappa_{\lambda }\right)\cdot t}\left(1-e^{-\Lambda_{\;\lambda }\cdot t}\right)}{2\cdot \Lambda_{\;\lambda }}\right]\;>\;0\;,\\ & \;\lim_{m\to \infty }{\bar{\vartheta }}_{\left\lfloor \sigma^{2}mt\right\rfloor }^{\left(m\right)}\;=\;\frac{{\left(\Lambda_{\;\lambda }-\kappa_{\lambda }\right)}^{2}}{\Lambda_{\;\lambda }}\cdot \left[\frac{1-e^{-\frac{1}{2}\left(3\Lambda_{\;\lambda }+\kappa_{\lambda }\right)\cdot t}}{3\Lambda_{\;\lambda }+\kappa_{\lambda }}+\frac{e^{-\Lambda_{\;\lambda }\cdot t}-e^{-\frac{1}{2}\left(\Lambda_{\;\lambda }+\kappa_{\lambda }\right)\cdot t}}{\Lambda_{\;\lambda }-\kappa_{\lambda }}\right]\;>\;0\;.\\ \left(l\right) & \;\mathrm{for}\;\lambda \in ]{\tilde{\lambda }}_{-},{\tilde{\lambda }}_{+}[\backslash \left[0,1\right],\;\mathrm{there}\;\mathrm{holds}\;\mathrm{for}\;\mathrm{the}\;\mathrm{respective}\;\mathrm{quantities}\;\mathrm{defined}\;\mathrm{in}\;(146)\;\mathrm{to}\;(149)\\ & \;\lim_{m\to \infty }m\cdot {\underline{\zeta }}_{\left\lfloor \sigma^{2}mt\right\rfloor }^{\left(m\right)}\;=\;\frac{{\left(\Lambda_{\;\lambda }-\kappa_{\lambda }\right)}^{2}}{2\sigma^{2}\cdot \kappa_{\lambda }}\cdot e^{-\Lambda_{\;\lambda }\cdot t}\cdot \left(1-e^{-\kappa_{\lambda }\cdot t}\right)\;>\;0\;,\\ & \;\lim_{m\to \infty }{\underline{\vartheta }}_{\left\lfloor \sigma^{2}mt\right\rfloor }^{\left(m\right)}\;=\;\frac{{\left(\Lambda_{\lambda }-\kappa_{\lambda }\right)}^{2}}{2\cdot \kappa_{\lambda }}\cdot \left[\frac{1-e^{-\Lambda_{\lambda }\cdot t}}{\Lambda_{\lambda }}-\frac{1-e^{-(\Lambda_{\lambda }+\kappa_{\lambda })\cdot t}}{\Lambda_{\lambda }+\kappa_{\lambda }}\right]\;>\;0\;,\\ & \;\lim_{m\to \infty }m\cdot {\bar{\zeta }}_{\left\lfloor \sigma^{2}mt\right\rfloor }^{\left(m\right)}\;=\;\frac{{\left(\Lambda_{\;\lambda }-\kappa_{\lambda }\right)}^{2}}{2\cdot \sigma^{2}}\cdot e^{-\frac{1}{2}\left(\Lambda_{\lambda }+\kappa_{\lambda }\right)\cdot t}\cdot \left[t\;-\;\frac{1-e^{-\Lambda_{\lambda }\cdot t}}{\Lambda_{\lambda }}\right]\;>\;0\;,\\ & \;\lim_{m\to \infty }{\bar{\vartheta }}_{\left\lfloor \sigma^{2}mt\right\rfloor }^{\left(m\right)}\;=\;{\left(\Lambda_{\lambda }-\kappa_{\lambda }\right)}^{2}\cdot [\frac{\left(\Lambda_{\lambda }-\kappa_{\lambda }\right)\left(1-e^{-\frac{1}{2}\left(\Lambda_{\lambda }+\kappa_{\lambda }\right)\cdot t}\right)}{\Lambda_{\lambda }\cdot {\left(\Lambda_{\lambda }+\kappa_{\lambda }\right)}^{2}}\\ & \;+\;\frac{1-e^{-\frac{1}{2}\left(3\Lambda_{\lambda }+\kappa_{\lambda }\right)\cdot t}}{\Lambda_{\lambda }\cdot \left(3\Lambda_{\lambda }+\kappa_{\lambda }\right)}-\frac{e^{-\frac{1}{2}\left(\Lambda_{\lambda }+\kappa_{\lambda }\right)\cdot t}}{\Lambda_{\lambda }+\kappa_{\lambda }}\cdot t]\;>\;0\;.\; \end{array}$$

**Proof of Lemma** **A6.** For each of the assertions (a) to (l), we will make use of l’Hospital’s rule. To begin with, we obtain for arbitrary μ,ν∈R

(A71) $$\begin{array}{c} \;\lim_{m\to \infty }m\left[1-{\left(\beta_{A}^{\left(m\right)}\right)}^{\mu }{\left(\beta_{H}^{\left(m\right)}\right)}^{\nu }\right]\;=\;\lim_{m\to \infty }m^{2}\left[\mu \cdot {\left(\beta_{A}^{\left(m\right)}\right)}^{\mu -1}{\left(\beta_{H}^{\left(m\right)}\right)}^{\nu }\frac{\kappa_{A}}{\sigma^{2}\;m^{2}}+\nu \cdot {\left(\beta_{A}^{\left(m\right)}\right)}^{\mu }{\left(\beta_{H}^{\left(m\right)}\right)}^{\nu -1}\frac{\kappa_{H}}{\sigma^{2}\;m^{2}}\right]\\ \;=\;\mu \;\frac{\kappa_{A}}{\sigma^{2}}+\nu \;\frac{\kappa_{H}}{\sigma^{2}}\;. \end{array}$$

From this, the first part of (a) follows immediately and the second part is a direct consequence of the definition of $\beta_{\lambda }^{\left(m\right)}$. Part (b) can be deduced from (A71):

$$\begin{array}{ccc} \lim_{m\to \infty }m^{2}\cdot a_{1}^{\left(m\right)} & = & \lim_{m\to \infty }\frac{m}{2\sigma^{2}}\cdot [\lambda \cdot \kappa_{A}\left(1-{\left(\beta_{A}^{\left(m\right)}\right)}^{\lambda -1}{\left(\beta_{H}^{\left(m\right)}\right)}^{1-\lambda }\right)\\ & & +\;\left(1-\lambda \right)\cdot \kappa_{H}\left(1-{\left(\beta_{A}^{\left(m\right)}\right)}^{\lambda }{\left(\beta_{H}^{\left(m\right)}\right)}^{-\lambda }\right)]\;=\;-\frac{\lambda \left(1-\lambda \right){\left(\kappa_{A}-\kappa_{H}\right)}^{2}}{2\sigma^{4}}\;=\;-\frac{\Lambda_{\lambda }^{2}-\kappa_{\lambda }^{2}}{2\sigma^{4}}\;. \end{array}$$

For the proof of (c), we rely on the inequalities ${\underline{x}}_{0}^{\left(m\right)}\leq x_{0}^{\left(m\right)}\leq {\bar{x}}_{0}^{\left(m\right)}$ (m∈N), where ${\underline{x}}_{0}^{\left(m\right)}$ and ${\bar{x}}_{0}^{\left(m\right)}$ are the obvious notational adaptions of (124) and (126), respectively. Notice that ${\underline{x}}_{0}^{\left(m\right)}$ and ${\bar{x}}_{0}^{\left(m\right)}$ are solutions of the (again adapted) quadratic equations ${\underline{Q}}_{\lambda }^{\left(m\right)}\left(x\right)=x$ resp. ${\bar{Q}}_{\lambda }^{\left(m\right)}\left(x\right)=x$ (cf. (127) and (128)). These solutions clearly exist in the case λ∈]0,1[. For sufficiently large approximations steps m∈N, these solutions also exist in the case $\lambda \in ]{\tilde{\lambda }}_{-},{\tilde{\lambda }}_{+}[\backslash \left[0,1\right]$ since (138) together with parts (a) and (b) imply

$$\lim_{m\to \infty }{\left(m\cdot (1-q_{\lambda }^{\left(m\right)})\right)}^{2}-2\cdot q_{\lambda }^{\left(m\right)}\cdot m^{2}\cdot a_{1}^{\left(m\right)}\;=\;\sigma^{-2}\cdot \left[\lambda \kappa_{A}^{2}+\left(1-\lambda \right)\kappa_{H}^{2}\right]\;>\;0,\;\mathrm{for}\;\lambda \in ]{\tilde{\lambda }}_{-},{\tilde{\lambda }}_{+}[\backslash \left[0,1\right].$$

To prove part (c), we show that the limits of ${\underline{x}}_{0}^{\left(m\right)}$ and ${\bar{x}}_{0}^{\left(m\right)}$ coincide. Assume first that λ∈]0,1[. Using (a) and (b), we obtain together with the obvious limit $\lim_{m\to \infty }q_{\lambda }^{\left(m\right)}=1$

(A72) $$\begin{array}{ccc} \lim_{m\to \infty }m\cdot {\bar{x}}_{0}^{\left(m\right)} & = & \lim_{m\to \infty }{\left(q_{\lambda }^{\left(m\right)}\right)}^{-1}\cdot \left[m\cdot \left(1-q_{\lambda }^{\left(m\right)}\right)-\sqrt{{\left(m\cdot (1-q_{\lambda }^{\left(m\right)})\right)}^{2}-2\cdot q_{\lambda }^{\left(m\right)}\cdot m^{2}\cdot a_{1}^{\left(m\right)}}\right]\\ & = & \frac{\kappa_{\lambda }}{\sigma^{2}}\;-\;\sqrt{{\left(\frac{\kappa_{\lambda }}{\sigma^{2}}\right)}^{2}+\frac{\Lambda_{\;\lambda }^{2}-\kappa_{\lambda }^{2}}{\sigma^{4}}}\;=\;-\frac{\Lambda_{\lambda }-\kappa_{\lambda }}{\sigma^{2}}\;. \end{array}$$

Let ${\underline{\underline{x}}}_{0}^{\left(m\right)}$ be the adapted version of the auxiliary fixed-point lower bound defined in (125). By incorporating $\lim_{m\to \infty }\beta_{\lambda }^{\left(m\right)}=1$ we obtain with (a) and (b)

$$\lim_{m\to \infty }{\underline{\underline{x}}}_{0}^{\left(m\right)}\;=\;\lim_{m\to \infty }\max\left\{-\beta_{\lambda }^{\left(m\right)}\;,\;\frac{q_{\lambda }^{\left(m\right)}-\beta_{\lambda }^{\left(m\right)}}{1-q_{\lambda }^{\left(m\right)}}\right\}\;=\;\lim_{m\to \infty }\frac{1}{m}\cdot \frac{m^{2}\cdot a_{1}^{\left(m\right)}}{m\cdot \left(1-q_{\lambda }^{\left(m\right)}\right)}\;=\;0,$$

which implies

(A73) $$\begin{array}{ccc} \lim_{m\to \infty }m\cdot {\underline{x}}_{0}^{\left(m\right)} & = & \lim_{m\to \infty }\frac{e^{-{\underline{\underline{x}}}_{0}^{\left(m\right)}}}{q_{\lambda }^{\left(m\right)}}\cdot \left[m\cdot \left(1-q_{\lambda }^{\left(m\right)}\right)-\sqrt{{\left(m\cdot (1-q_{\lambda }^{\left(m\right)})\right)}^{2}-2\cdot e^{{\underline{\underline{x}}}_{0}^{\left(m\right)}}q_{\lambda }^{\left(m\right)}\cdot m^{2}\cdot a_{1}^{\left(m\right)}}\right]\\ & = & \frac{\kappa_{\lambda }}{\sigma^{2}}\;-\;\sqrt{{\left(\frac{\kappa_{\lambda }}{\sigma^{2}}\right)}^{2}+\frac{\Lambda_{\;\lambda }^{2}-\kappa_{\lambda }^{2}}{\sigma^{4}}}\;=\;-\frac{\Lambda_{\lambda }-\kappa_{\lambda }}{\sigma^{2}}\;. \end{array}$$

Combining (A72) and (A73), the desired result (c) follows for λ∈]0,1[. Assume now that $\lambda \in ]{\tilde{\lambda }}_{-},{\tilde{\lambda }}_{+}[\backslash \left[0,1\right]$. In this case the approximates ${\underline{x}}_{0}^{\left(m\right)}$ and ${\bar{x}}_{0}^{\left(m\right)}$ have a different form, given in (124) and (126). However, the calculations work out in the same way: with parts (a) and (b) we get

$$\begin{array}{ccc} \lim_{m\to \infty }m\cdot {\underline{x}}_{0}^{\left(m\right)} & = & \lim_{m\to \infty }\frac{1}{q_{\lambda }^{\left(m\right)}}\cdot \left[m\cdot \left(1-q_{\lambda }^{\left(m\right)}\right)-\sqrt{{\left(m\cdot (1-q_{\lambda }^{\left(m\right)})\right)}^{2}-2\cdot q_{\lambda }^{\left(m\right)}\cdot m^{2}\cdot a_{1}^{\left(m\right)}}\right]\\ & = & \frac{\kappa_{\lambda }}{\sigma^{2}}\;-\;\sqrt{{\left(\frac{\kappa_{\lambda }}{\sigma^{2}}\right)}^{2}+\frac{\Lambda_{\;\lambda }^{2}-\kappa_{\lambda }^{2}}{\sigma^{4}}}\;=\;-\frac{\Lambda_{\lambda }-\kappa_{\lambda }}{\sigma^{2}}\;, \end{array}$$

as well as

$$\begin{array}{ccc} \lim_{m\to \infty }m\cdot {\bar{x}}_{0}^{\left(m\right)} & = & \lim_{m\to \infty }m\cdot \left(1-q_{\lambda }^{\left(m\right)}\right)-\sqrt{{\left(m\cdot (1-q_{\lambda }^{\left(m\right)})\right)}^{2}-2\cdot m^{2}\cdot a_{1}^{\left(m\right)}}\\ & = & \frac{\kappa_{\lambda }}{\sigma^{2}}\;-\;\sqrt{{\left(\frac{\kappa_{\lambda }}{\sigma^{2}}\right)}^{2}+\frac{\Lambda_{\;\lambda }^{2}-\kappa_{\lambda }^{2}}{\sigma^{4}}}\;=\;-\frac{\Lambda_{\lambda }-\kappa_{\lambda }}{\sigma^{2}}\;, \end{array}$$

which finally finishes the proof of part (c). Assertion (d) is a direct consequence of (c). Since the representations of the parameters $c^{(m),S},$$d^{(m),S},$$c^{(m),T},$$d^{(m),T}$ are the same in both cases λ∈]0,1[ and $\lambda \in ]{\tilde{\lambda }}_{-},{\tilde{\lambda }}_{+}[\backslash \left[0,1\right]$, the following considerations hold generally. Part (e) follows from (b) and (c) by

$$\lim_{m\to \infty }m\cdot \left(1-d^{(m),S}\right)\;=\;\lim_{m\to \infty }\frac{m^{2}\cdot a_{1}^{\left(m\right)}}{m\cdot x_{0}^{\left(m\right)}}\;=\;\frac{\Lambda_{\;\lambda }+\kappa_{\lambda }}{2\sigma^{2}}\;>\;0\;.$$

Notice that this term is positive since on $]{\tilde{\lambda }}_{-},{\tilde{\lambda }}_{+}[\;\backslash \left\{0,1\right\}$ there holds $\kappa_{\lambda }>0$ as well as $\Lambda_{\lambda }>0$, cf. (A70). To prove (f), we apply the general limit $\;\lim_{x\to 0}\frac{e^{x}-1}{x}\;=\;1$ and get with (a), (c)

$$\lim_{m\to \infty }m\cdot \left(1-d^{(m),T}\right)\;=\;\lim_{m\to \infty }\left(m\cdot \left(1-q_{\lambda }^{\left(m\right)}\right)-q_{\lambda }^{\left(m\right)}\cdot m\cdot x_{0}^{\left(m\right)}\cdot \frac{e^{x_{0}^{\left(m\right)}}-1}{x_{0}^{\left(m\right)}}\right)\;=\;\frac{\Lambda_{\;\lambda }}{\sigma^{2}}\;.$$

The limit (g) can be obtained from (e) and (f):

$$\lim_{m\to \infty }m\cdot \left(1-d^{(m),S}d^{(m),T}\right)\;=\;\lim_{m\to \infty }\left\{m\cdot \left(1-d^{(m),S}\right)+d^{(m),S}\cdot m\cdot \left(1-d^{(m),T}\right)\right\}\;=\;\frac{3\Lambda_{\lambda }+\kappa_{\lambda }}{2\sigma^{2}}\;.$$

The assertions (h) resp. (i) resp. (j) follow from (e) resp. (f) resp. (g) by using the general relation $\lim_{m\to \infty }{\left(1+\frac{x_{m}}{m}\right)}^{m}=\exp\left\{\lim_{m\to \infty }x_{m}\right\}$. To get the last two parts (k) and (l), we make repeatedly use of the results (a) to (j) and combine them with the formulas (142) to (149) of Corollary 14. More detailed, for λ∈]0,1[(and thus $q_{\lambda }^{\left(m\right)}<\beta_{\lambda }^{\left(m\right)}$) we obtain

$$\begin{array}{ccc} m\cdot {\underline{\zeta }}_{\left\lfloor \sigma^{2}mt\right\rfloor }^{\left(m\right)} & = & m^{2}\cdot \Gamma_{<}^{\left(m\right)}\cdot \frac{{\left(d^{(m),T}\right)}^{\left\lfloor \sigma^{2}mt\right\rfloor -1}}{m\cdot \left(1-d^{(m),T}\right)}\cdot \left(1-{\left(d^{(m),T}\right)}^{\left\lfloor \sigma^{2}mt\right\rfloor }\right)\\ & \overset{m\to \infty }{⟶} & \frac{{\left(\Lambda_{\;\lambda }-\kappa_{\lambda }\right)}^{2}}{2\sigma^{2}\cdot \Lambda_{\;\lambda }}\cdot e^{-\Lambda_{\;\lambda }\cdot t}\cdot \left(1-e^{-\Lambda_{\;\lambda }\cdot t}\right)\;>\;0\;,\\ {\underline{\vartheta }}_{\left\lfloor \sigma^{2}mt\right\rfloor }^{\left(m\right)} & = & m^{2}\cdot \Gamma_{<}^{\left(m\right)}\cdot \frac{1-{\left(d^{(m),T}\right)}^{\left\lfloor \sigma^{2}mt\right\rfloor }}{{\left(m\cdot \left(1-d^{(m),T}\right)\right)}^{2}}\cdot \left[1-\frac{d^{(m),T}\left(1+{\left(d^{(m),T}\right)}^{\left\lfloor \sigma^{2}mt\right\rfloor }\right)}{1+d^{(m),T}}\right]\\ & \overset{m\to \infty }{⟶} & \frac{1}{4}\cdot {\left(\frac{\Lambda_{\;\lambda }-\kappa_{\lambda }}{\Lambda_{\;\lambda }}\right)}^{2}\cdot {\left(1-e^{-\Lambda_{\;\lambda }\cdot t}\right)}^{2}\;>\;0\;, \end{array}$$

$$\begin{array}{ccc} m\cdot {\bar{\zeta }}_{\left\lfloor \sigma^{2}mt\right\rfloor }^{\left(m\right)} & = & m^{2}\cdot \Gamma_{<}^{\left(m\right)}\cdot [\frac{{\left(d^{(m),S}\right)}^{\left\lfloor \sigma^{2}mt\right\rfloor }-{\left(d^{(m),T}\right)}^{\left\lfloor \sigma^{2}mt\right\rfloor }}{m\cdot \left(1-d^{(m),T}\right)-m\cdot \left(1-d^{(m),S}\right)}\\ & & \;-\;{\left(d^{(m),S}\right)}^{\left\lfloor \sigma^{2}mt\right\rfloor -1}\cdot \frac{1-{\left(d^{(m),T}\right)}^{\left\lfloor \sigma^{2}mt\right\rfloor }}{m\cdot \left(1-d^{(m),T}\right)}]\\ & \overset{m\to \infty }{⟶} & \frac{{\left(\Lambda_{\;\lambda }-\kappa_{\lambda }\right)}^{2}}{\sigma^{2}}\cdot \left[\frac{e^{-\frac{1}{2}\left(\Lambda_{\;\lambda }+\kappa_{\lambda }\right)\cdot t}-e^{-\Lambda_{\;\lambda }\cdot t}}{\Lambda_{\;\lambda }-\kappa_{\lambda }}-\frac{e^{-\frac{1}{2}\left(\Lambda_{\;\lambda }+\kappa_{\lambda }\right)\cdot t}\left(1-e^{-\Lambda_{\;\lambda }\cdot t}\right)}{2\cdot \Lambda_{\;\lambda }}\right]\;>\;0\;, \end{array}$$

$$\begin{array}{ccc} {\bar{\vartheta }}_{\left\lfloor \sigma^{2}mt\right\rfloor }^{\left(m\right)} & = & \frac{m^{2}\cdot \Gamma_{<}^{\left(m\right)}\cdot d^{(m),T}}{m\cdot \left(1-d^{(m),T}\right)}\cdot \left[\frac{1-{\left(d^{(m),S}d^{(m),T}\right)}^{\left\lfloor \sigma^{2}mt\right\rfloor }}{m\cdot \left(1-d^{(m),S}d^{(m),T}\right)}-\frac{{\left(d^{(m),S}\right)}^{\left\lfloor \sigma^{2}mt\right\rfloor }-{\left(d^{(m),T}\right)}^{\left\lfloor \sigma^{2}mt\right\rfloor }}{m\cdot \left(1-d^{(m),T}\right)-m\cdot \left(1-d^{(m),S}\right)}\right]\\ & \overset{m\to \infty }{⟶} & \frac{{\left(\Lambda_{\;\lambda }-\kappa_{\lambda }\right)}^{2}}{\Lambda_{\;\lambda }}\cdot \left[\frac{1-e^{-\frac{1}{2}\left(3\Lambda_{\;\lambda }+\kappa_{\lambda }\right)\cdot t}}{3\Lambda_{\;\lambda }+\kappa_{\lambda }}+\frac{e^{-\Lambda_{\;\lambda }\cdot t}-e^{-\frac{1}{2}\left(\Lambda_{\;\lambda }+\kappa_{\lambda }\right)\cdot t}}{\Lambda_{\;\lambda }-\kappa_{\lambda }}\right]\;>\;0\;. \end{array}$$

For $\lambda \in ]{\tilde{\lambda }}_{-},{\tilde{\lambda }}_{+}[\backslash \left[0,1\right]$(and thus $q_{\lambda }^{\left(m\right)}>\beta_{\lambda }^{\left(m\right)}$) we get

$$\begin{array}{ccc} m\cdot {\underline{\zeta }}_{\left\lfloor \sigma^{2}mt\right\rfloor }^{\left(m\right)} & = & m^{2}\cdot \Gamma_{>}^{\left(m\right)}\cdot \frac{{\left(d^{(m),T}\right)}^{\left\lfloor \sigma^{2}mt\right\rfloor }-{\left(d^{(m),S}\right)}^{2\cdot \left\lfloor \sigma^{2}mt\right\rfloor }}{m\cdot \left(1-d^{(m),S}\right)\left(1+d^{(m),S}\right)-m\cdot \left(1-d^{(m),T}\right)}\\ & \overset{m\to \infty }{⟶} & \frac{{\left(\Lambda_{\;\lambda }-\kappa_{\lambda }\right)}^{2}}{2\sigma^{2}\cdot \kappa_{\lambda }}\cdot e^{-\Lambda_{\;\lambda }\cdot t}\cdot \left(1-e^{-\kappa_{\lambda }\cdot t}\right)\;>\;0\;,\\ {\underline{\vartheta }}_{\left\lfloor \sigma^{2}mt\right\rfloor }^{\left(m\right)} & = & \frac{m^{2}\cdot \Gamma_{>}^{\left(m\right)}}{m\cdot \left(1-d^{(m),S}\right)\left(1+d^{(m),S}\right)-m\cdot \left(1-d^{(m),T}\right)}\\ & & \cdot \left[\frac{d^{(m),T}\cdot \left(1-{\left(d^{(m),T}\right)}^{\left\lfloor \sigma^{2}mt\right\rfloor }\right)}{m\cdot \left(1-d^{(m),T}\right)}-\frac{{\left(d^{(m),S}\right)}^{2}\cdot \left(1-{\left(d^{(m),S}\right)}^{2\cdot \left\lfloor \sigma^{2}mt\right\rfloor }\right)}{m\cdot \left(1-d^{(m),S}\right)\left(1+d^{(m),S}\right)}\right]\\ & \overset{m\to \infty }{⟶} & \frac{{\left(\Lambda_{\lambda }-\kappa_{\lambda }\right)}^{2}}{2\cdot \kappa_{\lambda }}\cdot \left[\frac{1-e^{-\Lambda_{\lambda }\cdot t}}{\Lambda_{\lambda }}-\frac{1-e^{-(\Lambda_{\lambda }+\kappa_{\lambda })\cdot t}}{\Lambda_{\lambda }+\kappa_{\lambda }}\right]\;>\;0\;,\\ m\cdot {\bar{\zeta }}_{\left\lfloor \sigma^{2}mt\right\rfloor }^{\left(m\right)} & = & m^{2}\cdot \Gamma_{>}^{\left(m\right)}\cdot {\left(d^{(m),S}\right)}^{\left\lfloor \sigma^{2}mt\right\rfloor -1}\cdot \left[\frac{\left\lfloor \sigma^{2}mt\right\rfloor }{m}\;-\;\frac{1-{\left(d^{(m),T}\right)}^{\left\lfloor \sigma^{2}mt\right\rfloor }}{m\cdot \left(1-d^{(m),T}\right)}\right]\\ & \overset{m\to \infty }{⟶} & \frac{{\left(\Lambda_{\;\lambda }-\kappa_{\lambda }\right)}^{2}}{2\cdot \sigma^{2}}\cdot e^{-\frac{1}{2}\left(\Lambda_{\lambda }+\kappa_{\lambda }\right)\cdot t}\cdot \left[t\;-\;\frac{1-e^{-\Lambda_{\lambda }\cdot t}}{\Lambda_{\lambda }}\right]\;>\;0\;, \end{array}$$

$$\begin{array}{ccc} {\bar{\vartheta }}_{\left\lfloor \sigma^{2}mt\right\rfloor }^{\left(m\right)} & = & m^{2}\cdot \Gamma_{>}^{\left(m\right)}\cdot [\frac{m\cdot \left(1-d^{(m),T}\right)-m\cdot \left(1-d^{(m),S}\right)}{m^{2}\cdot {\left(1-d^{(m),S}\right)}^{2}\cdot m\cdot \left(1-d^{(m),T}\right)}\cdot \left(1-{\left(d^{(m),S}\right)}^{\left\lfloor \sigma^{2}mt\right\rfloor }\right)\\ & & \;+\;\frac{d^{(m),T}\left(1-{\left(d^{(m),S}d^{(m),T}\right)}^{\left\lfloor \sigma^{2}mt\right\rfloor }\right)}{m\cdot \left(1-d^{(m),T}\right)\cdot m\cdot \left(1-d^{(m),S}d^{(m),T}\right)}-\frac{{\left(d^{(m),S}\right)}^{\left\lfloor \sigma^{2}mt\right\rfloor }}{m\cdot \left(1-d^{(m),S}\right)}\cdot \frac{\left\lfloor \sigma^{2}mt\right\rfloor }{m}]\\ & \;\overset{m\to \infty }{⟶} & {\left(\Lambda_{\lambda }-\kappa_{\lambda }\right)}^{2}\cdot \left[\frac{\left(\Lambda_{\lambda }-\kappa_{\lambda }\right)\left(1-e^{-\frac{1}{2}\left(\Lambda_{\lambda }+\kappa_{\lambda }\right)\cdot t}\right)}{\Lambda_{\lambda }\cdot {\left(\Lambda_{\lambda }+\kappa_{\lambda }\right)}^{2}}+\frac{1-e^{-\frac{1}{2}\left(3\Lambda_{\lambda }+\kappa_{\lambda }\right)\cdot t}}{\Lambda_{\lambda }\cdot \left(3\Lambda_{\lambda }+\kappa_{\lambda }\right)}-\frac{e^{-\frac{1}{2}\left(\Lambda_{\lambda }+\kappa_{\lambda }\right)\cdot t}}{\Lambda_{\lambda }+\kappa_{\lambda }}\cdot t\right]>0.□ \end{array}$$

**Proof of Theorem** **11.** It suffices to compute the limits of the bounds given in Corollary 14 as *m* tends to infinity. This is done by applying Lemma A6 which provides corresponding limits of all quantities of interest. Accordingly, for all t>0 the lower bound (153) in the case λ∈]0,1[ can be obtained from (140), (142) and (143) by

$$\begin{array}{c} \lim_{m\to \infty }\exp\{x_{0}^{\left(m\right)}\cdot \left[X_{0}^{\left(m\right)}-\frac{\eta }{\sigma^{2}}\cdot \frac{d^{(m),T}}{1-d^{(m),T}}\right]\left(1-{\left(d^{(m),T}\right)}^{\left\lfloor \sigma^{2}mt\right\rfloor }\right)\\ \;+\;x_{0}^{\left(m\right)}\frac{\eta }{\sigma^{2}}\cdot \left\lfloor \sigma^{2}mt\right\rfloor \;+\;{\underline{\zeta }}_{\left\lfloor \sigma^{2}mt\right\rfloor }^{\left(m\right)}\cdot X_{0}^{\left(m\right)}\;+\;{\underline{\vartheta }}_{\left\lfloor \sigma^{2}mt\right\rfloor }^{\left(m\right)}\}\\ =\lim_{m\to \infty }\exp\{m\cdot x_{0}^{\left(m\right)}\cdot \left[\frac{X_{0}^{\left(m\right)}}{m}-\frac{\eta }{\sigma^{2}}\cdot \frac{d^{(m),T}}{m\cdot \left(1-d^{(m),T}\right)}\right]\left(1-{\left(d^{(m),T}\right)}^{\left\lfloor \sigma^{2}mt\right\rfloor }\right)\\ \;+\;m\cdot x_{0}^{\left(m\right)}\frac{\eta }{\sigma^{2}}\cdot \frac{\left\lfloor \sigma^{2}mt\right\rfloor }{m}\;+\;m\cdot {\underline{\zeta }}_{\left\lfloor \sigma^{2}mt\right\rfloor }^{\left(m\right)}\cdot \frac{X_{0}^{\left(m\right)}}{m}\;+\;{\underline{\vartheta }}_{\left\lfloor \sigma^{2}mt\right\rfloor }^{\left(m\right)}\}\\ =\exp\{-\frac{\Lambda_{\;\lambda }-\kappa_{\lambda }}{\sigma^{2}}\cdot \left[{\tilde{X}}_{0}-\frac{\eta }{\sigma^{2}}\cdot \frac{\sigma^{2}}{\Lambda_{\;\lambda }}\right]\left(1-e^{-\Lambda_{\;\lambda }t}\right)-\frac{\Lambda_{\;\lambda }-\kappa_{\lambda }}{\sigma^{2}}\cdot \frac{\eta }{\sigma^{2}}\cdot \sigma^{2}t\\ \;+\;\frac{{\left(\Lambda_{\;\lambda }-\kappa_{\lambda }\right)}^{2}}{2\sigma^{2}\cdot \Lambda_{\;\lambda }}\cdot e^{-\Lambda_{\;\lambda }\cdot t}\cdot \left(1-e^{-\Lambda_{\;\lambda }\cdot t}\right)\cdot {\tilde{X}}_{0}+\frac{\eta }{4\sigma^{2}}\cdot {\left(\frac{\Lambda_{\;\lambda }-\kappa_{\lambda }}{\Lambda_{\;\lambda }}\right)}^{2}\cdot {\left(1-e^{-\Lambda_{\;\lambda }\cdot t}\right)}^{2}\}\\ =\exp\left\{-\frac{\Lambda_{\;\lambda }-\kappa_{\lambda }}{\sigma^{2}}\left[{\tilde{X}}_{0}-\frac{\eta }{\Lambda_{\;\lambda }}\right]\left(1-e^{-\Lambda_{\;\lambda }\cdot t}\right)-\frac{\eta }{\sigma^{2}}\left(\Lambda_{\;\lambda }-\kappa_{\lambda }\right)\cdot t\;+\;L_{\lambda }^{\left(1\right)}\left(t\right)\cdot {\tilde{X}}_{0}\;+\;\frac{\eta }{\sigma^{2}}\cdot L_{\lambda }^{\left(2\right)}\left(t\right)\right\}. \end{array}$$

For all t>0, the upper bound (154) in the case λ∈]0,1[ follows analogously from (141), (144), (145) by

$$\begin{array}{c} \lim_{m\to \infty }\exp\{x_{0}^{\left(m\right)}\cdot \left[X_{0}^{\left(m\right)}-\frac{\eta }{\sigma^{2}}\cdot \frac{d^{(m),S}}{1-d^{(m),S}}\right]\left(1-{\left(d^{(m),S}\right)}^{\left\lfloor \sigma^{2}mt\right\rfloor }\right)\\ \;+\;x_{0}^{\left(m\right)}\frac{\eta }{\sigma^{2}}\cdot \left\lfloor \sigma^{2}mt\right\rfloor \;-\;{\bar{\zeta }}_{\left\lfloor \sigma^{2}mt\right\rfloor }^{\left(m\right)}\cdot X_{0}^{\left(m\right)}\;-\;{\bar{\vartheta }}_{\left\lfloor \sigma^{2}mt\right\rfloor }^{\left(m\right)}\}\\ =\lim_{m\to \infty }\exp\{m\cdot x_{0}^{\left(m\right)}\cdot \left[\frac{X_{0}^{\left(m\right)}}{m}-\frac{\eta }{\sigma^{2}}\cdot \frac{d^{(m),S}}{m\cdot \left(1-d^{(m),S}\right)}\right]\left(1-{\left(d^{(m),S}\right)}^{\left\lfloor \sigma^{2}mt\right\rfloor }\right)\\ \;+\;m\cdot x_{0}^{\left(m\right)}\frac{\eta }{\sigma^{2}}\cdot \frac{\left\lfloor \sigma^{2}mt\right\rfloor }{m}\;-\;m\cdot {\bar{\zeta }}_{\left\lfloor \sigma^{2}mt\right\rfloor }^{\left(m\right)}\cdot \frac{X_{0}^{\left(m\right)}}{m}\;-\;{\bar{\vartheta }}_{\left\lfloor \sigma^{2}mt\right\rfloor }^{\left(m\right)}\}\\ =\exp\{-\frac{\Lambda_{\;\lambda }-\kappa_{\lambda }}{\sigma^{2}}\left[{\tilde{X}}_{0}-\frac{\eta }{\sigma^{2}}\cdot \frac{2\sigma^{2}}{\Lambda_{\;\lambda }+\kappa_{\lambda }}\right]\left(1-\left(e^{-\frac{1}{2}\left(\Lambda_{\;\lambda }+\kappa_{\lambda }\right)t}\right)\right)-\frac{\Lambda_{\;\lambda }-\kappa_{\lambda }}{\sigma^{2}}\cdot \frac{\eta }{\sigma^{2}}\cdot \sigma^{2}t\\ \;-\;\frac{{\left(\Lambda_{\;\lambda }-\kappa_{\lambda }\right)}^{2}}{\sigma^{2}}\cdot \left[\frac{e^{-\frac{1}{2}\left(\Lambda_{\;\lambda }+\kappa_{\lambda }\right)\cdot t}-e^{-\Lambda_{\;\lambda }\cdot t}}{\Lambda_{\;\lambda }-\kappa_{\lambda }}-\frac{e^{-\frac{1}{2}\left(\Lambda_{\;\lambda }+\kappa_{\lambda }\right)\cdot t}\left(1-e^{-\Lambda_{\;\lambda }\cdot t}\right)}{2\cdot \Lambda_{\;\lambda }}\right]\cdot {\tilde{X}}_{0}\\ \;-\;\frac{\eta }{\sigma^{2}}\frac{{\left(\Lambda_{\;\lambda }-\kappa_{\lambda }\right)}^{2}}{\Lambda_{\;\lambda }}\cdot \left[\frac{1-e^{-\frac{1}{2}\left(3\Lambda_{\;\lambda }+\kappa_{\lambda }\right)\cdot t}}{3\Lambda_{\;\lambda }+\kappa_{\lambda }}+\frac{e^{-\Lambda_{\;\lambda }\cdot t}-e^{-\frac{1}{2}\left(\Lambda_{\;\lambda }+\kappa_{\lambda }\right)\cdot t}}{\Lambda_{\;\lambda }-\kappa_{\lambda }}\right]\}\\ =\exp\{-\frac{\Lambda_{\;\lambda }-\kappa_{\lambda }}{\sigma^{2}}\left[{\tilde{X}}_{0}-\frac{\eta }{\frac{1}{2}\left(\Lambda_{\;\lambda }+\kappa_{\lambda }\right)}\right]\left(1-e^{-\frac{1}{2}\left(\Lambda_{\;\lambda }+\kappa_{\lambda }\right)\cdot t}\right)-\frac{\eta }{\sigma^{2}}\left(\Lambda_{\;\lambda }-\kappa_{\lambda }\right)\cdot t\\ \;-\;U_{\lambda }^{\left(1\right)}\left(t\right)\cdot {\tilde{X}}_{0}\;-\;\frac{\eta }{\sigma^{2}}\cdot U_{\lambda }^{\left(2\right)}\left(t\right)\}.\; \end{array}$$

In the case $\lambda \in ]{\tilde{\lambda }}_{-},{\tilde{\lambda }}_{+}[\backslash \left[0,1\right]$, the lower bound as well as the upper bound of the Hellinger integral limit is obtained analogously, by taking into account that the quantities ${\underline{\zeta }}_{n}^{\left(m\right)},{\underline{\vartheta }}_{n}^{\left(m\right)},{\bar{\zeta }}_{n}^{\left(m\right)},{\bar{\vartheta }}_{n}^{\left(m\right)}$ now have the form (146) to (149) instead of (142) to (145). Thus, the functions $L_{\lambda }^{\left(1\right)}\left(t\right),U_{\lambda }^{\left(1\right)}\left(t\right),L_{\lambda }^{\left(2\right)}\left(t\right),U_{\lambda }^{\left(2\right)}\left(t\right)$ are obtained by employing the limits of part (l) of Lemma A6 instead of part (k). □

The next Lemma (and parts of its proof) will be useful for the verification of Theorem 12:

**Lemma** **A7.**
*Recall the bounds on the Hellinger integral m–limit given in (153) and (154) of Theorem 11, in terms of $L_{\lambda }^{\left(i\right)}\left(t\right)$ and $U_{\lambda }^{\left(i\right)}\left(t\right)$ (i=1,2) defined by (155) to (158). Correspondingly, one gets the following λ–limits for all t∈[0,∞[:*

(a) *for all $\kappa_{A}\in ]0,\infty [$ and all $\kappa_{H}\in [0,\infty [$ with $\kappa_{A}\neq \kappa_{H}$*
  (A74) $$\lim_{\lambda ↗1}\frac{\partial L_{\lambda }^{\left(1\right)}\left(t\right)}{\partial \lambda }\;=\;\lim_{\lambda ↗1}\frac{\partial L_{\lambda }^{\left(2\right)}\left(t\right)}{\partial \lambda }\;=\;\lim_{\lambda ↗1}\frac{\partial \;U_{\lambda }^{\left(1\right)}\left(t\right)}{\partial \lambda }\;=\;\lim_{\lambda ↗1}\frac{\partial \;U_{\lambda }^{\left(2\right)}\left(t\right)}{\partial \lambda }\;=\;0\;.$$
(b) *for $\kappa_{A}=0$ and all $\kappa_{H}\in ]0,\infty [$*
  (A75) $$\begin{array}{c} \lim_{\lambda ↗1}\frac{\partial L_{\lambda }^{\left(1\right)}\left(t\right)}{\partial \lambda }\;=\;-\frac{\kappa_{H}^{2}\cdot t}{2\sigma^{2}}\;, \end{array}$$
  (A76) $$\begin{array}{c} \lim_{\lambda ↗1}\frac{\partial L_{\lambda }^{\left(2\right)}\left(t\right)}{\partial \lambda }\;=\;-\frac{\kappa_{H}^{2}\cdot t^{2}}{4}\;, \end{array}$$
  (A77) $$\begin{array}{c} \lim_{\lambda ↗1}\frac{\partial \;U_{\lambda }^{\left(1\right)}\left(t\right)}{\partial \lambda }\;=\;\lim_{\lambda ↗1}\frac{\partial \;U_{\lambda }^{\left(2\right)}\left(t\right)}{\partial \lambda }\;=\;0\;. \end{array}$$

**Proof of Lemma** **A7.** For all $\kappa_{A},\kappa_{H}\in [0,\infty [$ with $\kappa_{A}\neq \kappa_{H}$ one can deduce from (150) as well as (155) to (158) the following derivatives:

(A78) $$\begin{array}{c} \;\frac{\partial L_{\lambda }^{\left(1\right)}\left(t\right)}{\partial \lambda }\;=\;\frac{1}{2\sigma^{2}}\{\frac{t}{2}{\left(\frac{\Lambda_{\;\lambda }-\kappa_{\lambda }}{\Lambda_{\;\lambda }}\right)}^{2}\left(\kappa_{A}^{2}-\kappa_{H}^{2}\right)\left[2e^{-2\Lambda_{\;\lambda }t}-e^{-\Lambda_{\;\lambda }t}\right]\\ \;+\;e^{-\Lambda_{\;\lambda }t}\frac{1-e^{-\Lambda_{\;\lambda }t}}{\Lambda_{\;\lambda }}\left[\frac{\Lambda_{\;\lambda }-\kappa_{\lambda }}{\Lambda_{\;\lambda }}\left(\kappa_{A}^{2}-\kappa_{H}^{2}-2\Lambda_{\;\lambda }\left(\kappa_{A}-\kappa_{H}\right)\right)-{\left(\frac{\Lambda_{\;\lambda }-\kappa_{\lambda }}{\Lambda_{\;\lambda }}\right)}^{2}\frac{\kappa_{A}^{2}-\kappa_{H}^{2}}{2}\right]\}\;, \end{array}$$

(A79) $$\begin{array}{c} \;\frac{\partial L_{\lambda }^{\left(2\right)}\left(t\right)}{\partial \lambda }=\frac{1}{4}\{\frac{\Lambda_{\;\lambda }-\kappa_{\lambda }}{\Lambda_{\;\lambda }}\cdot {\left(\frac{1-e^{-\Lambda_{\;\lambda }t}}{\Lambda_{\;\lambda }}\right)}^{2}\cdot \left(\kappa_{A}^{2}-\kappa_{H}^{2}-2\Lambda_{\;\lambda }\left(\kappa_{A}-\kappa_{H}\right)-\frac{\Lambda_{\;\lambda }-\kappa_{\lambda }}{\Lambda_{\;\lambda }}\left(\kappa_{A}^{2}-\kappa_{H}^{2}\right)\right)\\ \;+\;t\cdot e^{-\Lambda_{\;\lambda }t}\cdot {\left(\frac{\Lambda_{\;\lambda }-\kappa_{\lambda }}{\Lambda_{\;\lambda }}\right)}^{2}\cdot \frac{1-e^{-\Lambda_{\;\lambda }t}}{\Lambda_{\;\lambda }}\cdot \left(\kappa_{A}^{2}-\kappa_{H}^{2}\right)\}\;, \end{array}$$

(A80) $$\begin{array}{c} \;\frac{\partial \;U_{\lambda }^{\left(1\right)}\left(t\right)}{\partial \lambda }\;=\;\frac{1}{\sigma^{2}}\{\frac{\Lambda_{\;\lambda }-\kappa_{\lambda }}{2\Lambda_{\;\lambda }}\left[t\;e^{-\Lambda_{\;\lambda }t}\left(\kappa_{A}^{2}-\kappa_{H}^{2}\right)-\frac{t}{2}\;e^{-\frac{1}{2}\left(\Lambda_{\;\lambda }+\kappa_{\lambda }\right)t}\left(\kappa_{A}^{2}-\kappa_{H}^{2}+2\Lambda_{\;\lambda }\left(\kappa_{A}-\kappa_{H}\right)\right)\right]\\ \;-\;\frac{e^{-\frac{1}{2}\left(\Lambda_{\;\lambda }+\kappa_{\lambda }\right)t}-e^{-\Lambda_{\;\lambda }t}}{2\Lambda_{\;\lambda }}\cdot \left(\kappa_{A}^{2}-\kappa_{H}^{2}-2\Lambda_{\;\lambda }\left(\kappa_{A}-\kappa_{H}\right)\right)\\ \;+{\left(\frac{\Lambda_{\;\lambda }-\kappa_{\lambda }}{2\Lambda_{\;\lambda }}\right)}^{2}[\frac{t}{2}\;e^{-\frac{1}{2}\left(\Lambda_{\;\lambda }+\kappa_{\lambda }\right)t}\left(\kappa_{A}^{2}-\kappa_{H}^{2}+2\Lambda_{\;\lambda }\left(\kappa_{A}-\kappa_{H}\right)\right)\\ \;-\frac{t}{2}e^{-\frac{1}{2}\left(3\Lambda_{\;\lambda }+\kappa_{\lambda }\right)t}\left(3\left(\kappa_{A}^{2}-\kappa_{H}^{2}\right)+2\Lambda_{\;\lambda }\left(\kappa_{A}-\kappa_{H}\right)\right)\\ \;+e^{-\frac{1}{2}\left(\Lambda_{\;\lambda }+\kappa_{\lambda }\right)t}\cdot \frac{1-e^{-\Lambda_{\;\lambda }t}}{\Lambda_{\;\lambda }}\cdot \left(\kappa_{A}^{2}-\kappa_{H}^{2}\right)]\\ \;+\frac{\Lambda_{\;\lambda }-\kappa_{\lambda }}{\Lambda_{\;\lambda }}\left(\kappa_{A}^{2}-\kappa_{H}^{2}-2\Lambda_{\;\lambda }\left(\kappa_{A}-\kappa_{H}\right)\right)\left[\frac{e^{-\frac{1}{2}\left(\Lambda_{\;\lambda }+\kappa_{\lambda }\right)t}-e^{-\Lambda_{\;\lambda }t}}{\Lambda_{\;\lambda }-\kappa_{\lambda }}-\frac{e^{-\frac{1}{2}\left(\Lambda_{\;\lambda }+\kappa_{\lambda }\right)t}\left(1-e^{-\Lambda_{\;\lambda }t}\right)}{2\Lambda_{\lambda }}\right]\}\;, \end{array}$$

(A81) $$\begin{array}{ccc} \frac{\partial \;U_{\lambda }^{\left(2\right)}\left(t\right)}{\partial \lambda } & = & \frac{{\left(\Lambda_{\;\lambda }-\kappa_{\lambda }\right)}^{2}}{\Lambda_{\;\lambda }\left(3\Lambda_{\;\lambda }+\kappa_{\lambda }\right)}[\frac{t}{2}\;e^{-\frac{1}{2}\left(3\Lambda_{\;\lambda }+\kappa_{\lambda }\right)t}\left(3\frac{\kappa_{A}^{2}-\kappa_{H}^{2}}{2\Lambda_{\;\lambda }}+\kappa_{A}-\kappa_{H}\right)\\ & & \;-\;\frac{1-e^{-\frac{1}{2}\left(3\Lambda_{\;\lambda }+\kappa_{\lambda }\right)t}}{3\Lambda_{\;\lambda }+\kappa_{\lambda }}\cdot \left(3\;\frac{\kappa_{A}^{2}-\kappa_{H}^{2}}{2\Lambda_{\;\lambda }}+\kappa_{A}-\kappa_{H}\right)]\\ & & \;+\;\frac{\Lambda_{\;\lambda }-\kappa_{\lambda }}{\Lambda_{\;\lambda }}\left[\frac{t}{2}\;e^{-\frac{1}{2}\left(\Lambda_{\;\lambda }+\kappa_{\lambda }\right)t}\left(\frac{\kappa_{A}^{2}-\kappa_{H}^{2}}{2\Lambda_{\;\lambda }}+\kappa_{A}-\kappa_{H}\right)-t\;e^{-\Lambda_{\;\lambda }t}\;\frac{\kappa_{A}^{2}-\kappa_{H}^{2}}{2\Lambda_{\;\lambda }}\right]\\ & & \;+\;\frac{e^{-\frac{1}{2}\left(\Lambda_{\;\lambda }+\kappa_{\lambda }\right)t}-e^{-\Lambda_{\;\lambda }t}}{\Lambda_{\;\lambda }}\left(\frac{\kappa_{A}^{2}-\kappa_{H}^{2}}{2\Lambda_{\;\lambda }}-\kappa_{A}+\kappa_{H}\right)\\ & & \;+\;\left[2\left(\frac{\kappa_{A}^{2}-\kappa_{H}^{2}}{2\Lambda_{\;\lambda }}-\kappa_{A}+\kappa_{H}\right)-\frac{\Lambda_{\;\lambda }-\kappa_{\lambda }}{\Lambda_{\;\lambda }^{2}}\cdot \frac{\kappa_{A}^{2}-\kappa_{H}^{2}}{2}\right]\\ & & \;\cdot \;\frac{1}{\Lambda_{\;\lambda }}\left[\frac{\Lambda_{\;\lambda }-\kappa_{\lambda }}{3\Lambda_{\;\lambda }+\kappa_{\lambda }}\left(1-e^{-\frac{1}{2}\left(3\Lambda_{\;\lambda }+\kappa_{\lambda }\right)t}\right)-e^{-\frac{1}{2}\left(\Lambda_{\;\lambda }+\kappa_{\lambda }\right)t}+e^{-\Lambda_{\;\lambda }t}\right]\;. \end{array}$$

If $\kappa_{A}\in ]0,\infty [$ and $\kappa_{H}\in [0,\infty [$ with $\kappa_{A}\neq \kappa_{H}$, then one gets $\lim_{\lambda ↗1}\Lambda_{\;\lambda }=\lim_{\lambda ↗1}\kappa_{\lambda }=\kappa_{A}>0$ which implies (A74) from (A78) to (A81). For the proof of part (b), let us correspondingly assume $\kappa_{A}=0$ and $\kappa_{H}\in ]0,\infty [$, which by (150) leads to $\kappa_{\lambda }=\kappa_{H}\cdot \left(1-\lambda \right)$, $\Lambda_{\;\lambda }=\kappa_{H}\cdot \sqrt{1-\lambda }$ and the convergences $\lim_{\lambda ↗1}\Lambda_{\;\lambda }=\lim_{\lambda ↗1}\kappa_{\lambda }=0$. From this, the assertions (A75), (A76), (A77) follow in a straightforward manner from (A78), (A79), (A80) – respectively – by using (parts of) the obvious relations

(A82) $$\lim_{\lambda ↗1}\frac{\kappa_{\lambda }}{\Lambda_{\;\lambda }}=0,\;\lim_{\lambda ↗1}\frac{\Lambda_{\;\lambda }\pm \kappa_{\lambda }}{\Lambda_{\;\lambda }}\;=\;\lim_{\lambda ↗1}\frac{\Lambda_{\;\lambda }-\kappa_{\lambda }}{\Lambda_{\;\lambda }+\kappa_{\lambda }}\;=\;1\;,$$

(A83) $$\lim_{\lambda ↗1}\frac{1-e^{-c_{\lambda }\cdot t}}{c_{\lambda }}\;=\;t\;\mathrm{for}\;\mathrm{all}\;c_{\lambda }\in \left\{\Lambda_{\;\lambda },\frac{\Lambda_{\;\lambda }+\kappa_{\lambda }}{2},\frac{3\;\Lambda_{\;\lambda }+\kappa_{\lambda }}{2}\right\}\;.$$

In order to get the last assertion in (A77), we make use of the following limits

(A84) $$\lim_{\lambda ↗1}\frac{1}{\Lambda_{\;\lambda }-\kappa_{\lambda }}-\frac{3}{3\Lambda_{\;\lambda }+\kappa_{\lambda }}\;=\;\lim_{\lambda ↗1}\frac{4\;\kappa_{H}}{\left(\kappa_{H}-\kappa_{H}\cdot \sqrt{1-\lambda }\right)\cdot \left(3\;\kappa_{H}+\kappa_{H}\cdot \sqrt{1-\lambda }\right)}\;=\;\frac{4}{3\;\kappa_{H}}\;$$

and

(A85) $$\lim_{\lambda ↗1}\frac{1}{\Lambda_{\;\lambda }}\left[\frac{1-e^{-\frac{1}{2}\left(3\Lambda_{\;\lambda }+\kappa_{\lambda }\right)t}}{3\Lambda_{\;\lambda }+\kappa_{\lambda }}-\frac{1-e^{-\Lambda_{\;\lambda }t}}{\Lambda_{\;\lambda }-\kappa_{\lambda }}+\frac{1-e^{-\frac{1}{2}\left(\Lambda_{\;\lambda }+\kappa_{\lambda }\right)t}}{\Lambda_{\;\lambda }-\kappa_{\lambda }}\right]\;=\;0\;.$$

To see (A85), let us first observe that the involved limit can be rewritten as

(A86) $$\begin{array}{c} \lim_{\lambda ↗1}\{\frac{1}{\Lambda_{\;\lambda }\left(\Lambda_{\;\lambda }-\kappa_{\lambda }\right)}\left[\frac{1}{3}-\frac{1}{3}\;e^{-\frac{1}{2}\left(3\Lambda_{\;\lambda }+\kappa_{\lambda }\right)t}+e^{-\Lambda_{\;\lambda }t}-e^{-\frac{1}{2}\left(\Lambda_{\;\lambda }+\kappa_{\lambda }\right)t}\right] \end{array}$$

(A87) $$\begin{array}{c} \;+\;\frac{1-e^{-\frac{1}{2}\left(3\Lambda_{\;\lambda }+\kappa_{\lambda }\right)t}}{\Lambda_{\;\lambda }}\left[\frac{1}{3\Lambda_{\;\lambda }+\kappa_{\lambda }}-\frac{1}{3(\Lambda_{\;\lambda }-\kappa_{\lambda })}\right]\}\;. \end{array}$$

Substituting $x:=\sqrt{1-\lambda }$ and applying l’Hospital’s rule twice, we get for the first limit (A86)

$$\begin{array}{c} \;\;\;\lim_{x↘0}\frac{\frac{1}{3}-\frac{1}{3}\;e^{-\frac{\kappa_{H}t}{2}\left(3x+x^{2}\right)}+e^{-\kappa_{H}tx}-e^{-\frac{\kappa_{H}t}{2}\left(x+x^{2}\right)}}{\kappa_{H}^{2}\cdot \left(x^{2}-x^{3}\right)}\\ \;=\lim_{x↘0}\frac{\frac{\kappa_{H}t}{6}\left(3+2x\right)\;e^{-\frac{\kappa_{H}t}{2}\left(3x+x^{2}\right)}-\kappa_{H}\;t\;e^{-\kappa_{H}tx}+\frac{\kappa_{H}t}{2}\;\left(1+2x\right)\;e^{-\frac{\kappa_{H}t}{2}\left(x+x^{2}\right)}}{\kappa_{H}^{2}\cdot \left(2x-3x^{2}\right)}\\ \;=\lim_{x↘0}\frac{\left[-\frac{\kappa_{H}^{2}t^{2}}{12}{\left(3+2x\right)}^{2}+\frac{\kappa_{H}t}{3}\right]e^{-\frac{\kappa_{H}t}{2}\left(3x+x^{2}\right)}+\kappa_{H}^{2}\;t^{2}\;e^{-\kappa_{H}tx}\;-\;\left[\frac{\kappa_{H}^{2}t^{2}}{4}{\left(1+2x\right)}^{2}-\kappa_{H}\;t\right]e^{-\frac{\kappa_{H}t}{2}\left(x+x^{2}\right)}}{\kappa_{H}^{2}\cdot \left(2-6x\right)}\\ \;=\frac{1}{2\kappa_{H}^{2}}\left[-\frac{3\kappa_{H}^{2}t^{2}}{4}+\frac{\kappa_{H}t}{3}+\kappa_{H}^{2}t^{2}-\frac{\kappa_{H}^{2}t^{2}}{4}+\kappa_{H}t\right]\;=\;\frac{2t}{3\;\kappa_{H}}. \end{array}$$

The second limit (A87) becomes

(A88) $$\begin{array}{c} \lim_{\lambda ↗1}\frac{1-e^{-\frac{1}{2}\left(3\Lambda_{\;\lambda }+\kappa_{\lambda }\right)t}}{3\Lambda_{\;\lambda }+\kappa_{\lambda }}\cdot \frac{3\Lambda_{\;\lambda }+\kappa_{\lambda }}{\Lambda_{\;\lambda }}\cdot \frac{-4\kappa_{H}}{\left(3\kappa_{H}+\sqrt{1-\lambda }\kappa_{H}\right)\left(3\kappa_{H}-3\sqrt{1-\lambda }\kappa_{H}\right)} \end{array}$$

and consequently (A85) follows. To proceed with the proof of (A77), we rearrange

(A89) $$\begin{array}{c} \;\lim_{\lambda ↗1}\frac{\partial \;U_{\lambda }^{\left(2\right)}\left(t\right)}{\partial \lambda }\;=\;\lim_{\lambda ↗1}\{{\left(\frac{\Lambda_{\;\lambda }-\kappa_{\lambda }}{\Lambda_{\;\lambda }}\right)}^{2}[\frac{\Lambda_{\;\lambda }}{3\Lambda_{\;\lambda }+\kappa_{\lambda }}\left(\frac{t}{2}\;e^{-\frac{1}{2}\left(3\Lambda_{\;\lambda }+\kappa_{\lambda }\right)t}\left(-\frac{3\kappa_{H}^{2}}{2\Lambda_{\;\lambda }}-\kappa_{H}\right)\right)\\ \;-\;\frac{\Lambda_{\;\lambda }}{3\Lambda_{\;\lambda }+\kappa_{\lambda }}\cdot \frac{1-e^{-\frac{1}{2}\left(3\Lambda_{\;\lambda }+\kappa_{\lambda }\right)t}}{3\Lambda_{\;\lambda }+\kappa_{\lambda }}\left(-\frac{3\kappa_{H}^{2}}{2\Lambda_{\;\lambda }}-\kappa_{H}\right)\;+\;\frac{\Lambda_{\;\lambda }}{\Lambda_{\;\lambda }-\kappa_{\lambda }}\frac{e^{-\frac{1}{2}\left(\Lambda_{\;\lambda }+\kappa_{\lambda }\right)t}-e^{-\Lambda_{\;\lambda }t}}{\Lambda_{\;\lambda }-\kappa_{\lambda }}\left(-\frac{\kappa_{H}^{2}}{2\Lambda_{\;\lambda }}+\kappa_{H}\right)\\ \;-\;\frac{\Lambda_{\;\lambda }}{\Lambda_{\;\lambda }-\kappa_{\lambda }}\left(-\frac{t}{2}\;e^{-\frac{1}{2}\left(\Lambda_{\;\lambda }+\kappa_{\lambda }\right)t}\left(-\frac{\kappa_{H}^{2}}{2\Lambda_{\;\lambda }}-\kappa_{H}\right)-t\;e^{-\Lambda_{\;\lambda }t}\frac{\kappa_{H}^{2}}{2\Lambda_{\;\lambda }}\right)]\\ \;+\left[\frac{\Lambda_{\;\lambda }-\kappa_{\lambda }}{\Lambda_{\;\lambda }}\left(-\kappa_{H}^{2}+2\Lambda_{\;\lambda }\kappa_{H}\right)+{\left(\frac{\Lambda_{\;\lambda }-\kappa_{\lambda }}{\Lambda_{\;\lambda }}\right)}^{2}\frac{\kappa_{H}^{2}}{2}\right]\cdot \left[\frac{1-e^{-\frac{1}{2}\left(3\Lambda_{\;\lambda }+\kappa_{\lambda }\right)t}}{\Lambda_{\;\lambda }\left(3\Lambda_{\;\lambda }+\kappa_{\lambda }\right)}-\frac{e^{-\frac{1}{2}\left(\Lambda_{\;\lambda }+\kappa_{\lambda }\right)t}-e^{-\Lambda_{\;\lambda }t}}{\Lambda_{\;\lambda }\left(\Lambda_{\;\lambda }-\kappa_{\lambda }\right)}\right]\}\\ =\;\lim_{\lambda ↗1}\{{\left(\frac{\Lambda_{\;\lambda }-\kappa_{\lambda }}{\Lambda_{\;\lambda }}\right)}^{2}[\frac{\kappa_{H}^{2}\;t}{4}\left(-\frac{3\;e^{-\frac{1}{2}\left(3\Lambda_{\;\lambda }+\kappa_{\lambda }\right)t}}{3\Lambda_{\;\lambda }+\kappa_{\lambda }}-\frac{e^{-\frac{1}{2}\left(\Lambda_{\;\lambda }+\kappa_{\lambda }\right)t}}{\Lambda_{\;\lambda }-\kappa_{\lambda }}+\frac{2\;e^{-\Lambda_{\;\lambda }t}}{\Lambda_{\;\lambda }-\kappa_{\lambda }}\right) \end{array}$$

(A90) $$\begin{array}{c} +\;\frac{\kappa_{H}^{2}}{2}\left(\frac{3\left(1-e^{-\frac{1}{2}\left(3\Lambda_{\;\lambda }+\kappa_{\lambda }\right)t}\right)}{{\left(3\Lambda_{\;\lambda }+\kappa_{\lambda }\right)}^{2}}-\frac{1-e^{-\Lambda_{\;\lambda }t}}{{\left(\Lambda_{\;\lambda }-\kappa_{\lambda }\right)}^{2}}+\frac{1-e^{-\frac{1}{2}\left(\Lambda_{\;\lambda }+\kappa_{\lambda }\right)t}}{{\left(\Lambda_{\;\lambda }-\kappa_{\lambda }\right)}^{2}}\right)\; \end{array}$$

(A91) $$\begin{array}{c} \;+\;\kappa_{H}(-\frac{\Lambda_{\;\lambda }}{3\Lambda_{\;\lambda }+\kappa_{\lambda }}\cdot \frac{t\;e^{-\frac{1}{2}\left(3\Lambda_{\;\lambda }+\kappa_{\lambda }\right)t}}{2}+\frac{\Lambda_{\;\lambda }}{3\Lambda_{\;\lambda }+\kappa_{\lambda }}\cdot \frac{1-e^{-\frac{1}{2}\left(3\Lambda_{\;\lambda }+\kappa_{\lambda }\right)t}}{3\Lambda_{\;\lambda }+\kappa_{\lambda }}-\frac{\Lambda_{\;\lambda }}{\Lambda_{\;\lambda }-\kappa_{\lambda }}\cdot \frac{t\;e^{-\frac{1}{2}\left(\Lambda_{\;\lambda }+\kappa_{\lambda }\right)t}}{2}\\ \;+\;\frac{\Lambda_{\;\lambda }}{\Lambda_{\;\lambda }-\kappa_{\lambda }}\cdot \frac{1-e^{-\Lambda_{\;\lambda }t}}{\Lambda_{\;\lambda }-\kappa_{\lambda }}-\frac{\Lambda_{\;\lambda }}{\Lambda_{\;\lambda }-\kappa_{\lambda }}\cdot \frac{1-e^{-\frac{1}{2}\left(\Lambda_{\;\lambda }+\kappa_{\lambda }\right)t}}{\Lambda_{\;\lambda }-\kappa_{\lambda }})]\\ \;+\left[\frac{\Lambda_{\;\lambda }-\kappa_{\lambda }}{\Lambda_{\;\lambda }}\left(-\kappa_{H}^{2}+2\Lambda_{\;\lambda }\kappa_{H}\right)+{\left(\frac{\Lambda_{\;\lambda }-\kappa_{\lambda }}{\Lambda_{\;\lambda }}\right)}^{2}\frac{\kappa_{H}^{2}}{2}\right]\cdot \left[\frac{1-e^{-\frac{1}{2}\left(3\Lambda_{\;\lambda }+\kappa_{\lambda }\right)t}}{\Lambda_{\;\lambda }\left(3\Lambda_{\;\lambda }+\kappa_{\lambda }\right)}-\frac{e^{-\frac{1}{2}\left(\Lambda_{\;\lambda }+\kappa_{\lambda }\right)t}-e^{-\Lambda_{\;\lambda }t}}{\Lambda_{\;\lambda }\left(\Lambda_{\;\lambda }-\kappa_{\lambda }\right)}\right]\}. \end{array}$$

By means of (A82) to (A84), the limit of the expression after the squared brackets in (A89) becomes

(A92) $$\lim_{\lambda ↗1}\{\frac{\kappa_{H}^{2}\;t}{4}\left[\frac{1-e^{-\frac{1}{2}\left(\Lambda_{\;\lambda }+\kappa_{\lambda }\right)t}}{\Lambda_{\;\lambda }-\kappa_{\lambda }}-2\;\frac{1-e^{-\Lambda_{\;\lambda }t}}{\Lambda_{\;\lambda }-\kappa_{\lambda }}+3\;\frac{1-e^{-\frac{1}{2}\left(3\Lambda_{\;\lambda }+\kappa_{\lambda }\right)t}}{3\Lambda_{\;\lambda }+\kappa_{\lambda }}+\frac{1}{\Lambda_{\;\lambda }-\kappa_{\lambda }}-\frac{3}{3\Lambda_{\;\lambda }+\kappa_{\lambda }}\right]=\frac{\kappa_{H}\;t}{3}\;,$$

and the limit of the expression in (A90) becomes with (A85)

(A93) $$\begin{array}{c} \lim_{\lambda ↗1}\{\frac{\Lambda_{\;\lambda }}{\Lambda_{\;\lambda }-\kappa_{\lambda }}\cdot \frac{\kappa_{H}^{2}}{2\Lambda_{\;\lambda }}\cdot \left[\frac{1-e^{-\frac{1}{2}\left(3\Lambda_{\;\lambda }+\kappa_{\lambda }\right)t}}{3\Lambda_{\;\lambda }+\kappa_{\lambda }}-\frac{1-e^{-\Lambda_{\;\lambda }t}}{\Lambda_{\;\lambda }-\kappa_{\lambda }}+\frac{1-e^{-\frac{1}{2}\left(\Lambda_{\;\lambda }+\kappa_{\lambda }\right)t}}{\Lambda_{\;\lambda }-\kappa_{\lambda }}\right]\\ \;-\;\frac{\kappa_{H}^{2}}{2}\cdot \frac{1-e^{-\frac{1}{2}\left(3\Lambda_{\;\lambda }+\kappa_{\lambda }\right)t}}{3\Lambda_{\;\lambda }+\kappa_{\lambda }}\cdot \left[\frac{1}{\Lambda_{\;\lambda }-\kappa_{\lambda }}-\frac{3}{3\Lambda_{\;\lambda }+\kappa_{\lambda }}\right]\;=\;-\frac{\kappa_{H}t}{3}\;. \end{array}$$

By putting (A91)–(A93) together with (A85) we finally end up with

$$\lim_{\lambda ↗1}\frac{\partial \;U_{\lambda }^{\left(2\right)}\left(t\right)}{\partial \lambda }\;=\;\left[\frac{\kappa_{H}t}{3}-\frac{\kappa_{H}t}{3}\right]+\kappa_{H}\left(-\frac{t}{6}+\frac{t}{6}-\frac{t}{2}+t-\frac{t}{2}\right)+\left[-\kappa_{H}^{2}+\frac{\kappa_{H}^{2}}{2}\right]\cdot 0\;=\;0\;,$$

which finishes the proof of Lemma A7. □

**Proof of Theorem** **12.** Recall from (131) the approximative Poisson offspring-distribution parameter $\beta_{•}^{\left(m\right)}:=1-\frac{\kappa_{•}}{\sigma^{2}m}$ and Poisson immigration-distribution parameter $\alpha_{•}^{\left(m\right)}:=\beta_{•}^{\left(m\right)}\cdot \frac{\eta }{\sigma^{2}}$, which is a special case of $\left(\beta_{A}^{\left(m\right)}\;,\beta_{H}^{\left(m\right)}\;,\alpha_{A}^{\left(m\right)}\;,\alpha_{H}^{\left(m\right)}\;\right)\in P_{\mathrm{NI}}\cup P_{\mathrm{SP},1}$. Let us first calculate $\lim_{m\to \infty }I\left(P_{A,\left\lfloor \sigma^{2}mt\right\rfloor }^{\left(m\right)}∥P_{H,\left\lfloor \sigma^{2}mt\right\rfloor }^{\left(m\right)}\right)$ by starting from Theorem 3(a). Correspondingly, we evaluate for all $\kappa_{A}\geq 0$, $\kappa_{H}\geq 0$ with $\kappa_{A}\neq \kappa_{H}$ by a twofold application of l’Hospital’s rule

(A94) $$\begin{array}{c} \;\lim_{m\to \infty }m^{2}\cdot \left[\beta_{A}^{\left(m\right)}\cdot \left(\log\left(\frac{\beta_{A}^{\left(m\right)}}{\beta_{H}^{\left(m\right)}}\right)-1\right)+\beta_{H}^{\left(m\right)}\right]=\lim_{m\to \infty }\frac{-m}{2\sigma^{2}}\left[\kappa_{A}\log\left(\frac{\beta_{A}^{\left(m\right)}}{\beta_{H}^{\left(m\right)}}\right)+\kappa_{H}\left(1-\frac{\beta_{A}^{\left(m\right)}}{\beta_{H}^{\left(m\right)}}\right)\right]\\ \;=\;\frac{1}{2\sigma^{4}}\cdot \lim_{m\to \infty }\frac{\beta_{H}^{\left(m\right)}\cdot \kappa_{A}-\beta_{A}^{\left(m\right)}\cdot \kappa_{H}}{{\left(\beta_{H}^{\left(m\right)}\right)}^{2}}\cdot \left(\kappa_{A}\cdot \frac{\beta_{H}^{\left(m\right)}}{\beta_{A}^{\left(m\right)}}-\kappa_{H}\right)\;=\;\frac{{\left(\kappa_{A}-\kappa_{H}\right)}^{2}}{2\sigma^{4}}\;. \end{array}$$

Additionally there holds

(A95) $$\lim_{m\to \infty }m\cdot \left(1-\beta_{A}^{\left(m\right)}\right)=\frac{\kappa_{A}}{\sigma^{2}}\;\mathrm{and}\;\lim_{m\to \infty }{\left(\beta_{A}^{\left(m\right)}\right)}^{\left\lfloor \sigma^{2}mt\right\rfloor }=\lim_{m\to \infty }{\left[{\left(1-\frac{\kappa_{A}}{\sigma^{2}m}\right)}^{m}\right]}^{\left\lfloor \sigma^{2}mt\right\rfloor /m}=e^{-\kappa_{A}\cdot t}\;.$$

For $\kappa_{A}>0$, we apply the upper part of formula (69) as well as (A94) and (A95) to derive

$$\begin{array}{c} \lim_{m\to \infty }I_{\lambda }\left(P_{A,\left\lfloor \sigma^{2}mt\right\rfloor }^{\left(m\right)}∥P_{H,\left\lfloor \sigma^{2}mt\right\rfloor }^{\left(m\right)}\right)\;=\;\lim_{m\to \infty }\left[\frac{m^{2}\cdot \left[\beta_{A}^{\left(m\right)}\cdot \left(\log\left(\frac{\beta_{A}^{\left(m\right)}}{\beta_{H}^{\left(m\right)}}\right)-1\right)+\beta_{H}^{\left(m\right)}\right]}{m\cdot (1-\beta_{A}^{\left(m\right)})}\right.\\ \;\cdot \left[\frac{X_{0}^{\left(m\right)}}{m}-\frac{\alpha_{A}^{\left(m\right)}}{m\cdot (1-\beta_{A}^{\left(m\right)})}\right]\cdot \left(1-{\left(\beta_{A}^{\left(m\right)}\right)}^{\left\lfloor \sigma^{2}mt\right\rfloor }\right)\\ \;+\frac{\alpha_{A}^{\left(m\right)}}{\beta_{A}^{\left(m\right)}\cdot m\cdot \left(1-\beta_{A}^{\left(m\right)}\right)}\cdot m^{2}\cdot \left[\beta_{A}^{\left(m\right)}\cdot \left(\log\left(\frac{\beta_{A}^{\left(m\right)}}{\beta_{H}^{\left(m\right)}}\right)-1\right)+\beta_{H}^{\left(m\right)}\right]\cdot \left.\frac{\left\lfloor \sigma^{2}mt\right\rfloor }{m}\right]\\ =\;\frac{{\left(\kappa_{A}-\kappa_{H}\right)}^{2}}{2\sigma^{2}\cdot \kappa_{A}}\cdot \left[\left({\tilde{X}}_{0}-\frac{\eta }{\kappa_{A}}\right)\cdot \left(1-e^{-\kappa_{A}\cdot t}\right)+\eta \cdot t\right]. \end{array}$$

For $\kappa_{A}=0$ (and thus $\kappa_{H}>0$, $\beta_{A}^{\left(m\right)}\equiv 1$, $\alpha_{A}^{\left(m\right)}\equiv \eta /\sigma^{2}$), we apply the lower part of formula (69) as well as (A94) and (A95) to obtain

$$\begin{array}{c} \lim_{m\to \infty }I_{\lambda }\left(P_{A,\left\lfloor \sigma^{2}mt\right\rfloor }^{\left(m\right)}∥P_{H,\left\lfloor \sigma^{2}mt\right\rfloor }^{\left(m\right)}\right)\;=\;\{\lim_{m\to \infty }m^{2}\cdot \left[\beta_{H}^{\left(m\right)}-\log\beta_{H}^{\left(m\right)}-1\right]\\ \cdot \left[\frac{\eta }{2\sigma^{2}}\cdot \frac{{\left(\left\lfloor \sigma^{2}mt\right\rfloor \right)}^{2}}{m^{2}}\;+\;\left(\frac{X_{0}^{\left(m\right)}}{m}+\frac{\eta }{2\sigma^{2}\cdot m}\right)\cdot \frac{\left\lfloor \sigma^{2}mt\right\rfloor }{m}\right]\}\;=\;\frac{\kappa_{H}^{2}}{2\sigma^{2}}\cdot \left[\frac{\eta }{2}\cdot t^{2}\;+\;{\tilde{X}}_{0}\cdot t\right]. \end{array}$$

Let us now calculate the “converse” double limit

$$\lim_{\lambda ↗1}\lim_{m\to \infty }I_{\lambda }\left(P_{A,\left\lfloor \sigma^{2}mt\right\rfloor }^{\left(m\right)}∥P_{H,\left\lfloor \sigma^{2}mt\right\rfloor }^{\left(m\right)}\right)\;=\;\lim_{\lambda ↗1}\lim_{m\to \infty }\frac{1-H_{\lambda }\left(P_{A,\left\lfloor \sigma^{2}mt\right\rfloor }^{\left(m\right)}∥P_{H,\left\lfloor \sigma^{2}mt\right\rfloor }^{\left(m\right)}\right)}{\lambda \cdot (1-\lambda )}\;.$$

This will be achieved by evaluating for each t>0 the two limits

(A96) $$\lim_{\lambda ↗1}\frac{1-d_{\lambda ,{\tilde{X}}_{0},t}^{L}}{\lambda \cdot (1-\lambda )}\;\mathrm{and}\;\lim_{\lambda ↗1}\frac{1-d_{\lambda ,{\tilde{X}}_{0},t}^{U}}{\lambda \cdot (1-\lambda )}$$

which will turn out to coincide; the involved lower and upper bound $d_{\lambda ,{\tilde{X}}_{0},t}^{L}$, $d_{\lambda ,{\tilde{X}}_{0},t}^{U}$ defined by (153) and (154) satisfy $\lim_{\lambda ↗1}d_{\lambda ,{\tilde{X}}_{0},t}^{L}=\lim_{\lambda ↗1}d_{\lambda ,{\tilde{X}}_{0},t}^{U}=1$ as an easy consequence of the limits (cf. 150)

(A97) $$\lim_{\lambda ↗1}\Lambda_{\;\lambda }=\kappa_{A}\geq 0\;\mathrm{and}\;\lim_{\lambda ↗1}\kappa_{\lambda }=\kappa_{A}\geq 0\;,$$

as well as the formulas (A82) and (A83) for the case $\kappa_{A}=0$. Accordingly, we compute

(A98) $$\begin{array}{c} \lim_{\lambda ↗1}\frac{1-d_{\lambda ,{\tilde{X}}_{0},t}^{L}}{\lambda \cdot (1-\lambda )}\;=\;\lim_{\lambda ↗1}\frac{-d_{\lambda ,{\tilde{X}}_{0},t}^{L}}{1-2\lambda }\;\frac{\partial }{\partial \lambda }[-\frac{\Lambda_{\;\lambda }-\kappa_{\lambda }}{\sigma^{2}}\cdot \left[{\tilde{X}}_{0}-\frac{\eta }{\Lambda_{\;\lambda }}\right]\cdot \left(1-e^{-\Lambda_{\;\lambda }\cdot t}\right)-\frac{\eta }{\sigma^{2}}\cdot \left(\Lambda_{\;\lambda }-\kappa_{\lambda }\right)\cdot t\\ \;+\;L_{\lambda }^{\left(1\right)}\left(t\right)\cdot {\tilde{X}}_{0}+\frac{\eta }{\sigma^{2}}\cdot L_{\lambda }^{\left(2\right)}\left(t\right)]\\ =\lim_{\lambda ↗1}\{-\frac{\Lambda_{\;\lambda }-\kappa_{\lambda }}{\sigma^{2}}\left[\left({\tilde{X}}_{0}-\frac{\eta }{\Lambda_{\;\lambda }}\right)\cdot te^{-\Lambda_{\;\lambda }\cdot t}\cdot \frac{\partial \;\Lambda_{\;\lambda }}{\partial \lambda }+\left(1-e^{-\Lambda_{\;\lambda }\cdot t}\right)\cdot \frac{\eta }{\Lambda_{\;\lambda }^{2}}\cdot \frac{\partial \;\Lambda_{\;\lambda }}{\partial \lambda }\right]\\ \;-\;\frac{1}{\sigma^{2}}\cdot \frac{\partial }{\partial \lambda }\left(\Lambda_{\;\lambda }-\kappa_{\lambda }\right)\cdot \left({\tilde{X}}_{0}-\frac{\eta }{\Lambda_{\;\lambda }}\right)\cdot \left(1-e^{-\Lambda_{\;\lambda }\cdot t}\right)-\frac{\eta \;t}{\sigma^{2}}\cdot \frac{\partial }{\partial \lambda }\left(\Lambda_{\;\lambda }-\kappa_{\lambda }\right)\\ \;+\;{\tilde{X}}_{0}\;\frac{\partial L_{\lambda }^{\left(1\right)}\left(t\right)}{\partial \lambda }\;+\;\frac{\eta }{\sigma^{2}}\;\frac{\partial L_{\lambda }^{\left(2\right)}\left(t\right)}{\partial \lambda }\}\;,\;\mathrm{with} \end{array}$$

(A99) $$\frac{\partial \;\Lambda_{\;\lambda }}{\partial \lambda }\;=\;\frac{\kappa_{A}^{2}-\kappa_{H}^{2}}{2\;\Lambda_{\;\lambda }}\;\mathrm{and}\;\frac{\partial \;\kappa_{\lambda }}{\partial \lambda }\;=\;\kappa_{A}-\kappa_{H}\;.$$

For the case $\kappa_{A}>0$, one can combine this with (A97) and (A74) to end up with

(A100) $$\lim_{\lambda ↗1}\frac{1-d_{\lambda ,{\tilde{X}}_{0},t}^{L}}{\lambda \cdot (1-\lambda )}\;=\;\frac{{\left(\kappa_{A}-\kappa_{H}\right)}^{2}}{2\sigma^{2}\cdot \kappa_{A}}\cdot \left[\left({\tilde{X}}_{0}-\frac{\eta }{\kappa_{A}}\right)\cdot \left(1-e^{-\kappa_{A}\cdot t}\right)+\eta \cdot t\right].$$

For the case $\kappa_{A}=0$, we continue the calculation (A98) by rearranging terms and by employing the Formulas (A75), (A76), (A82) and (A83) as well as the obvious relation $\frac{1}{\Lambda }-\frac{\Lambda -\kappa_{\lambda }}{\Lambda^{2}}=\frac{1}{\kappa_{H}}$ and obtain

(A101) $$\begin{array}{c} \;\lim_{\lambda ↗1}\frac{1-d_{\lambda ,{\tilde{X}}_{0},t}^{L}}{\lambda \cdot (1-\lambda )}\;=\;\lim_{\lambda ↗1}\{\frac{\kappa_{H}^{2}\cdot {\tilde{X}}_{0}}{2\sigma^{2}}\left[\frac{\Lambda_{\;\lambda }-\kappa_{\lambda }}{\Lambda_{\;\lambda }}\cdot t\cdot e^{-\Lambda_{\;\lambda }t}+\frac{1-e^{-\Lambda_{\;\lambda }t}}{\Lambda_{\;\lambda }}\right]\\ \;+\;\frac{\eta \cdot \kappa_{H}^{2}\cdot t}{2\sigma^{2}}\left[\frac{1}{\Lambda_{\;\lambda }}-\frac{\Lambda_{\;\lambda }-\kappa_{\lambda }}{\Lambda_{\;\lambda }^{2}}+\frac{\Lambda_{\;\lambda }-\kappa_{\lambda }}{\Lambda_{\;\lambda }}\cdot \frac{1-e^{-\Lambda_{\;\lambda }t}}{\Lambda_{\;\lambda }}\right]-\;\frac{\eta \cdot \kappa_{H}^{2}}{2\sigma^{2}}\cdot \frac{1-e^{-\Lambda_{\;\lambda }t}}{\Lambda_{\;\lambda }}\left[\frac{1}{\Lambda_{\;\lambda }}-\frac{\Lambda_{\;\lambda }-\kappa_{\lambda }}{\Lambda_{\;\lambda }^{2}}\right]\\ \;-\;\frac{\kappa_{H}\cdot {\tilde{X}}_{0}}{\sigma^{2}}\left(1-e^{-\Lambda_{\;\lambda }t}\right)\;+\;\frac{\eta \cdot \kappa_{H}}{\sigma^{2}}\left[\frac{1-e^{-\Lambda_{\;\lambda }t}}{\Lambda_{\;\lambda }}-t\right]+\;\frac{\partial L_{\lambda }^{\left(1\right)}\left(t\right)}{\partial \lambda }\cdot {\tilde{X}}_{0}\;+\;\frac{\eta }{\sigma^{2}}\cdot \frac{\partial L_{\lambda }^{\left(2\right)}\left(t\right)}{\partial \lambda }\}\\ =\frac{\kappa_{H}^{2}\;{\tilde{X}}_{0}\;t}{\sigma^{2}}+\frac{\eta \;\kappa_{H}^{2}\;t}{2\sigma^{2}}\left[\frac{1}{\kappa_{H}}+t\right]-\frac{\eta \;\kappa_{H}\;t}{2\sigma^{2}}-\frac{\kappa_{H}^{2}\;{\tilde{X}}_{0}\;t}{2\sigma^{2}}-\frac{\eta \;\kappa_{H}^{2}\;t^{2}}{4\sigma^{2}}\;=\;\frac{\kappa_{H}^{2}}{2\sigma^{2}}\cdot \left[\frac{\eta }{2}\cdot t^{2}+{\tilde{X}}_{0}\cdot t\right]. \end{array}$$

Let us now turn to the second limit (A96) for which we compute analogously to (A98)

(A102) $$\begin{array}{c} \lim_{\lambda ↗1}\frac{1-d_{\lambda ,{\tilde{X}}_{0},t}^{U}}{\lambda \cdot (1-\lambda )}\;=\;\lim_{\lambda ↗1}\frac{-d_{\lambda ,{\tilde{X}}_{0},t}^{U}}{1-2\lambda }\;\frac{\partial }{\partial \lambda }[-\frac{\Lambda_{\;\lambda }-\kappa_{\lambda }}{\sigma^{2}}\cdot \left[{\tilde{X}}_{0}-\frac{\eta }{\frac{1}{2}\left(\Lambda_{\;\lambda }+\kappa_{\lambda }\right)}\right]\cdot \left(1-e^{-\frac{1}{2}\left(\Lambda_{\;\lambda }+\kappa_{\lambda }\right)\cdot t}\right)\\ \;-\frac{\eta }{\sigma^{2}}\cdot \left(\Lambda_{\;\lambda }-\kappa_{\lambda }\right)\cdot t-U_{\lambda }^{\left(1\right)}\left(t\right)\cdot {\tilde{X}}_{0}-\frac{\eta }{\sigma^{2}}\cdot U_{\lambda }^{\left(2\right)}\left(t\right)]\\ =\lim_{\lambda ↗1}\{-\frac{\Lambda_{\;\lambda }-\kappa_{\lambda }}{\sigma^{2}}[\left({\tilde{X}}_{0}-\frac{\eta }{\frac{1}{2}\left(\Lambda_{\;\lambda }+\kappa_{\lambda }\right)}\right)\cdot \frac{t}{2}\cdot e^{-\frac{1}{2}\left(\Lambda_{\;\lambda }+\kappa_{\lambda }\right)\cdot t}\;\frac{\partial }{\partial \lambda }\left(\Lambda_{\;\lambda }+\kappa_{\lambda }\right)\\ \;+\;\left(1-e^{-\frac{1}{2}\left(\Lambda_{\;\lambda }+\kappa_{\lambda }\right)\cdot t}\right)\cdot \frac{2\cdot \eta }{{\left(\Lambda_{\;\lambda }+\kappa_{\lambda }\right)}^{2}}\cdot \frac{\partial }{\partial \lambda }\left(\Lambda_{\;\lambda }+\kappa_{\lambda }\right)]\\ \;-\;\frac{1}{\sigma^{2}}\cdot \frac{\partial }{\partial \lambda }\left(\Lambda_{\;\lambda }-\kappa_{\lambda }\right)\cdot \left({\tilde{X}}_{0}-\frac{\eta }{\frac{1}{2}\left(\Lambda_{\;\lambda }+\kappa_{\lambda }\right)}\right)\cdot \left(1-e^{-\frac{1}{2}\left(\Lambda_{\;\lambda }+\kappa_{\lambda }\right)\cdot t}\right)-\frac{\eta \;t}{\sigma^{2}}\cdot \frac{\partial }{\partial \lambda }\left(\Lambda_{\;\lambda }-\kappa_{\lambda }\right)\\ \;-\;\frac{\partial \;U_{\lambda }^{\left(1\right)}\left(t\right)}{\partial \lambda }\cdot {\tilde{X}}_{0}\;-\;\frac{\eta }{\sigma^{2}}\frac{\partial \;U_{\lambda }^{\left(2\right)}\left(t\right)}{\partial \lambda }\}\;. \end{array}$$

For the case $\kappa_{A}>0$, one can combine this with (A97), (A99) and (A74) to end up with

(A103) $$\lim_{\lambda ↗1}\frac{1-d_{\lambda ,{\tilde{X}}_{0},t}^{U}}{\lambda \cdot (1-\lambda )}\;=\;\frac{{\left(\kappa_{A}-\kappa_{H}\right)}^{2}}{2\sigma^{2}\cdot \kappa_{A}}\cdot \left[\left({\tilde{X}}_{0}-\frac{\eta }{\kappa_{A}}\right)\cdot \left(1-e^{-\kappa_{A}\cdot t}\right)+\eta \cdot t\right].$$

For the case $\kappa_{A}=0$, we continue the calculation of (A102) by rearranging terms and by employing the formulas (A77), (A82) and (A83) as well as the obvious relation $\lim_{\lambda ↗1}\frac{1}{\Lambda_{\;\lambda }}-\frac{\Lambda_{\;\lambda }-\kappa_{\lambda }}{\Lambda_{\;\lambda }\left(\Lambda_{\;\lambda }+\kappa_{\lambda }\right)}\;=\;\frac{2}{\kappa_{H}}$ to obtain

(A104) $$\begin{array}{c} \lim_{\lambda ↗1}\frac{1-d_{\lambda ,{\tilde{X}}_{0},t}^{U}}{\lambda \cdot (1-\lambda )}\;=\;\lim_{\lambda ↗1}\{\frac{t\cdot {\tilde{X}}_{0}}{4\sigma^{2}}\cdot \frac{\Lambda_{\;\lambda }-\kappa_{\lambda }}{\Lambda_{\;\lambda }}\cdot e^{-\frac{1}{2}\left(\Lambda_{\;\lambda }+\kappa_{\lambda }\right)\cdot t}\left(\kappa_{H}^{2}+2\Lambda_{\;\lambda }\kappa_{H}\right)\;\\ +\;\frac{{\tilde{X}}_{0}}{2\sigma^{2}}\cdot \frac{1-e^{-\frac{1}{2}\left(\Lambda_{\;\lambda }+\kappa_{\lambda }\right)\cdot t}}{\Lambda_{\;\lambda }}\left(\kappa_{H}^{2}-2\Lambda_{\;\lambda }\kappa_{H}\right)-\;\frac{\eta \cdot t}{\sigma^{2}}[\kappa_{H}\left(1+e^{-\frac{1}{2}\left(\Lambda_{\;\lambda }+\kappa_{\lambda }\right)\cdot t}\;\frac{\Lambda_{\;\lambda }-\kappa_{\lambda }}{\Lambda_{\;\lambda }+\kappa_{\lambda }}\right)\\ -\;\frac{\kappa_{H}^{2}}{2}\cdot \left(\frac{1}{\Lambda_{\;\lambda }}-\frac{\Lambda_{\;\lambda }-\kappa_{\lambda }}{\Lambda_{\;\lambda }\left(\Lambda_{\;\lambda }+\kappa_{\lambda }\right)}+\frac{\Lambda_{\;\lambda }-\kappa_{\lambda }}{\Lambda_{\;\lambda }+\kappa_{\lambda }}\cdot \frac{1-e^{-\frac{1}{2}\left(\Lambda_{\;\lambda }+\kappa_{\lambda }\right)\cdot t}}{\Lambda_{\;\lambda }}\right)]\\ +\;\frac{2\eta }{\sigma^{2}}\cdot \frac{1-e^{-\frac{1}{2}\left(\Lambda_{\;\lambda }+\kappa_{\lambda }\right)\cdot t}}{\Lambda_{\;\lambda }+\kappa_{\lambda }}\left[\kappa_{H}\left(1+\frac{\Lambda_{\;\lambda }-\kappa_{\lambda }}{\Lambda_{\;\lambda }+\kappa_{\lambda }}\right)-\frac{\kappa_{H}^{2}}{2}\left(\frac{1}{\Lambda_{\;\lambda }}-\frac{\Lambda_{\;\lambda }-\kappa_{\lambda }}{\Lambda_{\;\lambda }\left(\Lambda_{\;\lambda }+\kappa_{\lambda }\right)}\right)\right]\\ -\;\frac{\partial \;U_{\lambda }^{\left(1\right)}\left(t\right)}{\partial \lambda }\cdot {\tilde{X}}_{0}\;-\;\frac{\eta }{\sigma^{2}}\frac{\partial \;U_{\lambda }^{\left(2\right)}\left(t\right)}{\partial \lambda }\}\\ =\;\frac{\kappa_{H}^{2}\;t\;{\tilde{X}}_{0}}{4\sigma^{2}}+\frac{\kappa_{H}^{2}\;t\;{\tilde{X}}_{0}}{4\sigma^{2}}-\frac{\eta \;t}{\sigma^{2}}\left[2\kappa_{H}-\kappa_{H}-\frac{\kappa_{H}^{2}\;t}{4}\right]+\frac{\eta \;t}{\sigma^{2}}\left[2\kappa_{H}-\kappa_{H}\right]\;=\;\frac{\kappa_{H}^{2}}{2\sigma^{2}}\left[\frac{\eta }{2}\cdot t^{2}+{\tilde{X}}_{0}\cdot t\right]. \end{array}$$

Since (A100) coincides with (A103) and (A101) coincides with (A104), we have finished the proof. □

## References

[1] Liese F., Vajda I. (1987). Convex Statistical Distances.

[2] Read T.R.C., Cressie N.A.C. (1988). Goodness-of-Fit Statistics for Discrete Multivariate Data.

[3] Vajda I. (1989). Theory of Statistical Inference and Information.

[4] Csiszár I., Shields P.C. (2004). Information Theory and Statistics: A Tutorial.

[5] Stummer W. (2004). Exponentials, Diffusions, Finance, Entropy and Information.

[6] Pardo L. (2006). Statistical Inference Based on Divergence Measures.

[7] Liese F., Miescke K.J. (2008). Statistical Decision Theory: Estimation, Testing, and Selection.

[8] Basu A., Shioya H., Park C. (2011). Statistical Inference: The Minimum Distance Approach.

[9] Voinov V., Nikulin M., Balakrishnan N. (2013). Chi-Squared Goodness of Fit Tests with Applications.

[10] Liese F., Vajda I. (2006). On divergences and informations in statistics and information theory. IEEE Trans. Inform. Theory.

[11] Vajda I., van der Meulen E.C., Karian Z.A., Dudewicz E.J. (2010). Goodness-of-fit criteria based on observations quantized by hypothetical and empirical percentiles. Handbook of Fitting Statistical Distributions with R.

[12] Stummer W., Vajda I. (2012). On Bregman distances and divergences of probability measures. IEEE Trans. Inform. Theory.

[13] Kißlinger A.-L., Stummer W., Agostinelli C., Basu A., Filzmoser P., Mukherjee D. (2016). Robust statistical engineering by means of scaled Bregman distances. Recent Advances in Robust Statistics–Theory and Applications.

[14] Broniatowski M., Stummer W., Nielsen F. (2019). Some universal insights on divergences for statistics, machine learning and artificial intelligence. Geometric Structures of Information.

[15] Stummer W., Vajda I. (2007). Optimal statistical decisions about some alternative financial models. J. Econom..

[16] Stummer W., Lao W. (2012). Limits of Bayesian decision related quantities of binomial asset price models. Kybernetika.

[17] Csiszar I. (1963). Eine informationstheoretische Ungleichung und ihre Anwendung auf den Beweis der Ergodizität von Markoffschen Ketten. Publ. Math. Inst. Hungar. Acad. Sci..

[18] Ali M.S., Silvey D. (1966). A general class of coefficients of divergence of one distribution from another. J. Roy. Statist. Soc. B.

[19] Morimoto T. (1963). Markov processes and the H-theorem. J. Phys. Soc. Jpn.

[20] van Erven T., Harremoes P. (2014). Renyi divergence and Kullback-Leibler divergence. IEEE Trans. Inf. Theory.

[21] Newman C.M. (1973). On the orthogonality of independent increment processes. Topics in Probability Theory.

[22] Liese F. (1982). Hellinger integrals of Gaussian processes with independent increments. Stochastics.

[23] Memin J., Shiryayev A.N. (1985). Distance de Hellinger-Kakutani des lois correspondant a deux processus a accroissements indépendants. Probab. Theory Relat. Fields.

[24] Jacod J., Shiryaev A.N. (1987). Limit Theorems for Stochastic Processes.

[25] Linkov Y.N., Shevlyakov Y.A. (1998). Large deviation theorems in the hypotheses testing problems for processes with independent increments. Theory Stoch. Process.

[26] Liese F. (1985). Hellinger integrals, error probabilities and contiguity of Gaussian processes with independent increments and Poisson processes. J. Inf. Process. Cybern..

[27] Kabanov Y.M., Liptser R.S., Shiryaev A.N. (1986). On the variation distance for probability measures defined on a filtered space. Probab. Theory Relat. Fields.

[28] Liese F. (1986). Hellinger integrals of diffusion processes. Statistics.

[29] Vajda I. (1990). Distances and discrimination rates for stochastic processes. Stoch. Process. Appl..

[30] Stummer W. (1993). The Novikov and entropy conditions of multidimensional diffusion processes with singular drift. Probab. Theory Relat. Fields.

[31] Stummer W. (1999). On a statistical information measure of diffusion processes. Stat. Decis..

[32] Stummer W. (2001). On a statistical information measure for a generalized Samuelson-Black-Scholes model. Stat. Decis..

[33] Bartoszynski R., Le Cam L.M., Neyman J. (1967). Branching processes and the theory of epidemics. Proceedings of the Fifth Berkeley Symposium on Mathematical Statistics and Probability, Vol. IV.

[34] Ludwig D. (1975). Qualitative behaviour of stochastic epidemics. Math. Biosci..

[35] Becker N.G. (1976). Estimation for an epidemic model. Biometrics.

[36] Becker N.G. (1977). Estimation for discrete time branching processes with applications to epidemics. Biometrics.

[37] Metz J.A.J. (1978). The epidemic in a closed population with all susceptibles equally vulnerable; some results for large susceptible populations and small initial infections. Acta Biotheor..

[38] Heyde C.C. (1979). On assessing the potential severity of an outbreak of a rare infectious disease. Austral. J. Stat..

[39] Von Bahr B., Martin-Löf A. (1980). Threshold limit theorems for some epidemic processes. Adv. Appl. Prob..

[40] Ball F. (1983). The threshold behaviour of epidemic models. J. Appl. Prob..

[41] Jacob C. (2010). Branching processes: Their role in epidemics. Int. J. Environ. Res. Public Health.

[42] Barbour A.D., Reinert G. (2013). Approximating the epidemic curve. Electron. J. Probab..

[43] Britton T., Pardoux E., Britton T., Pardoux E. (2019). Stochastic epidemics in a homogeneous community. Stochastic Epidemic Models.

[44] Dion J.P., Gauthier G., Latour A. (1995). Branching processes with immigration and integer-valued time series. Serdica Math. J..

[45] Grunwald G.K., Hyndman R.J., Tedesco L., Tweedie R.L. (2000). Non-Gaussian conditional linear AR(1) models. Aust. N. Z. J. Stat..

[46] Kedem B., Fokianos K. (2002). An Regression Models for Time Series Analysis.

[47] Held L., Höhle M., Hofmann M. (2005). A statistical framework for the analysis of multivariate infectious disease surveillance counts. Stat. Model..

[48] Weiss C.H. (2018). An Introduction to Discrete-Valued Time Series.

[49] Feigin P.D., Passy U. (1981). The geometric programming dual to the extinction probability problem in simple branching processes. Ann. Probab..

[50] Mordecki E. (1994). Asymptotic mixed normality and Hellinger processes. Stoch. Stoch. Rep..

[51] Sriram T.N., Vidyashankar A.N. (2000). Minimum Hellinger distance estimation for supercritical Galton-Watson processes. Stat. Probab. Lett..

[52] Guttorp P. (1991). Statistical Inference for Branching Processes.

[53] Linkov Y.N., Lunyova L.A. (1996). Large deviation theorems in the hypothesis testing problems for the Galton-Watson processes with immigration. Theory Stoch. Process.

[54] Heathcote C.R. (1965). A branching process allowing immigration. J. R. Stat. Soc. B.

[55] Athreya K.B., Ney P.E. (1972). Branching Processes.

[56] Jagers P. (1975). Branching Processes with Biological Applications.

[57] Asmussen S., Hering H. (1983). Branching Processes.

[58] Haccou P., Jagers P., Vatutin V.A. (2005). Branching Processes: Variation, Growth, and Extinction of Populations.

[59] Heyde C.C., Seneta E. (1972). Estimation theory for growth and immigration rates in a multiplicative process. J. Appl. Probab..

[60] Basawa I.V., Rao B.L.S. (1980). Statistical Inference of Stochastic Processes.

[61] Basawa I.V., Scott D.J. (1983). Asymptotic Optimal Inference for Non-Ergodic Models.

[62] Sankaranarayanan G. (1989). Branching Processes and Its Estimation Theory.

[63] Wei C.Z., Winnicki J. (1990). Estimation of the means in the branching process with immigration. Ann. Stat..

[64] Winnicki J. (1991). Estimation of the variances in the branching process with immigration. Probab. Theory Relat. Fields.

[65] Yanev N.M., Ahsanullah M., Yanev G.P. (2008). Statistical inference for branching processes. Records and Branching Processes.

[66] Harris T.E. (1963). The Theory of Branching Processes.

[67] Gauthier G., Latour A. (1994). Convergence forte des estimateurs des parametres d’un processus GENAR(p). Ann. Sci. Math. Que..

[68] Latour A. (1998). Existence and stochastic structure of a non-negative integer-valued autoregressive process. J. Time Ser. Anal..

[69] Rydberg T.H., Shephard N. (2000). BIN models for trade-by-trade data. Modelling the number of trades in a fixed interval of time. Econometric Society World Congress.

[70] Brandt P.T., Williams J.T. (2001). A linear Poisson autoregressive model: The Poisson AR(p) model. Polit. Anal..

[71] Heinen A. (2003). Modelling time series count data: An autoregressive conditional Poisson model. Core Discussion Paper.

[72] Held L., Hofmann M., Höhle M., Schmid V. (2006). A two-component model for counts of infectious diseases. Biostatistics.

[73] Finkenstädt B.F., Bjornstad O.N., Grenfell B.T. (2002). A stochastic model for extinction and recurrence of epidemics: Estimation and inference for measles outbreak. Biostatistics.

[74] Ferland R., Latour A., Oraichi D. (2006). Integer-valued GARCH process. J. Time Ser. Anal..

[75] Weiß C.H. (2009). Modelling time series of counts with overdispersion. Stat. Methods Appl..

[76] Weiß C.H. (2010). The INARCH(1) model for overdispersed time series of counts. Comm. Stat. Sim. Comp..

[77] Weiß C.H. (2010). INARCH(1) processes: Higher-order moments and jumps. Stat. Probab. Lett..

[78] Weiß C.H., Testik M.C. (2012). Detection of abrupt changes in count data time series: Cumulative sum derivations for INARCH(1) models. J. Qual. Technol..

[79] Kaslow R.A., Evans A.S., Evans A.S., Kaslow R.A. (1997). Epidemiologic concepts and methods. Viral Infections of Humans.

[80] Osterholm M.T., Hedberg C.W., Bennett J.E., Dolin R., Blaser M.J. (2015). Epidemiologic principles. Mandell, Douglas, and Bennett’s Principles and Practice of Infectious Diseases.

[81] Grassly N.C., Fraser C. (2008). Mathematical models of infectious disease transmission. Nat. Rev..

[82] Keeling M.J., Rohani P. (2008). Modeling Infectious Diseases in Humans and Animals.

[83] Yan P., Brauer F., van den Driessche P., Wu J. (2008). Distribution theory stochastic processes and infectious disease modelling. Mathematical Epidemiology.

[84] Yan P., Chowell G. (2019). Quantitative Methods for Investigating Infectious Disease Outbreaks.

[85] Britton T. (2010). Stochastic epidemic models: A survey. Math. Biosc..

[86] Diekmann O., Heesterbeek H., Britton T. (2013). Mathematical Tools for Understanding Infectious Disease Dynamics.

[87] Cummings D.A.T., Lessler J., Nelson K.E., Masters Williams C. (2014). Infectious disease dynamics. Infectious Disease Epidemiology: Theory and Practice.

[88] Just W., Callender H., Drew LaMar M., Toporikova N., Robeva R.S. (2015). Transmission of infectious diseases: Data, models and simulations. Algebraic and Discrete Mathematical Methods of Modern Biology.

[89] Britton T., Giardina F. (2016). Introduction to statistical inference for infectious diseases. J. Soc. Franc. Stat..

[90] Fine P.E.M. (2003). The interval between successive cases of an infectious disease. Am. J. Epidemiol..

[91] Svensson A. (2007). A note on generation times in epidemic models. Math. Biosci..

[92] Svensson A. (2015). The influence of assumptions on generation time distributions in epidemic models. Math. Biosci..

[93] Wallinga J., Lipsitch M. (2007). How generation intervals shape the relationship between growth rates and reproductive numbers. Proc. R. Soc. B.

[94] Forsberg White L., Pagano M. (2008). A likelihood-based method for real-time estimation of the serial interval and reproductive number of an epidemic. Stat. Med..

[95] Nishiura H. (2010). Time variations in the generation time of an infectious disease: Implications for sampling to appropriately quantify transmission potential. Math. Biosci..

[96] Scalia Tomba G., Svensson A., Asikainen T., Giesecke J. (2010). Some model based considerations on observing generation times for communicable diseases. Math. Biosci..

[97] Trichereau J., Verret C., Mayet A., Manet G. (2012). Estimation of the reproductive number for A(H1N1) pdm09 influenza among the French armed forces, September 2009–March 2010. J. Infect..

[98] Vink M.A., Bootsma M.C.J., Wallinga J. (2014). Serial intervals of respiratory infectious diseases: A systematic review and analysis. Am. J. Epidemiol..

[99] Champredon D., Dushoff J. (2015). Intrinsic and realized generation intervals in infectious-disease transmission. Proc. R. Soc. B.

[100] An der Heiden M., Hamouda O. (2020). Schätzung der aktuellen Entwicklung der SARS-CoV-2-Epidemie in Deutschland— Nowcasting. Epid. Bull..

[101] Ferretti L., Wymant C., Kendall M., Zhao L., Nurtay A., Abeler-Dörner L., Parker M., Bonsall D., Fraser C. (2020). Quantifying SARS-CoV-2 transmission suggests epidemic control with digital contact tracing. Science.

[102] Ganyani T., Kremer C., Chen D., Torneri A., Faes C., Wallinga J., Hens N. (2020). Estimating the generation interval for COVID-19 based on symptom onset data. medRxiv Prepr..

[103] Li M., Liu K., Song Y., Wang M., Wu J. (2020). Serial interval and generation interval for respectively the imported and local infectors estimated using reported contact-tracing data of COVID-19 in China. medRxiv Prepr..

[104] Nishiura H., Linton N.M., Akhmetzhanov A.R. (2020). Serial interval of novel coronavirus (COVID-19) infections. medRxiv Prepr..

[105] Park M., Cook A.R., Lim J.J., Sun X., Dickens B.L. (2020). A systematic review of COVID-19 epidemiology based on current evidence. J. Clin. Med..

[106] Spouge J.L. (2019). An accurate approximation for the expected site frequency spectrum in a Galton-Watson process under an infinite sites mutation model. Theor. Popul. Biol..

[107] Taneyhill D.E., Dunn A.M., Hatcher M.J. (1999). The Galton-Watson branching process as a quantitative tool in parasitology. Parasitol. Today.

[108] Parnes D. (2017). Analyzing the contagion effect of foreclosures as a branching process: A close look at the years that follow the Great Recession. J. Account. Financ..

[109] Le Cam L. (1986). Asymptotic Methods in Statistical Decision Theory.

[110] Heyde C.C., Johnstone I.M. (1979). On asymptotic posterior normality for stochastic processes. J. R. Stat. Soc. B.

[111] Johnson R.A., Susarla V., van Ryzin J. (1979). Bayesian non-parametric estimation for age-dependent branching processes. Stoch. Proc. Appl..

[112] Scott D. (1987). On posterior asymptotic normality and asymptotic normality of estimators for the Galton-Watson process. J. R. Stat. Soc. B.

[113] Yanev N.M., Tsokos C.P. (1999). Decision-theoretic estimation of the offspring mean in mortal branching processes. Comm. Stat. Stoch. Models.

[114] Mendoza M., Gutierrez-Pena E. (2000). Bayesian conjugate analysis of the Galton-Watson process. Test.

[115] Feicht R., Stummer W., De La Grandville O. (2011). An explicit nonstationary stochastic growth model. Economic Growth and Development (Frontiers of Economics and Globalization, Vol. 11).

[116] Dorn F., Fuest C., Göttert M., Krolage C., Lautenbacher S., Link S., Peichl A., Reif M., Sauer S., Stöckli M. (2020). Die volkswirtschaftlichen Kosten des Corona-Shutdown für Deutschland: Eine Szenarienrechnung. ifo Schnelldienst.

[117] Dorn F., Khailaie S., Stöckli M., Binder S., Lange B., Peichl A., Vanella P., Wollmershäuser T., Fuest C., Meyer-Hermann M. (2020). Das gemeinsame Interesse von Gesundheit und Wirtschaft: Eine Szenarienrechnung zur Eindämmung der Corona-Pandemie. ifo Schnelld. Dig..

[118] Kißlinger A.-L., Stummer W. (2018). A new toolkit for robust distributional change detection. Appl. Stoch. Models Bus. Ind..

[119] Dehning J., Zierenberg J., Spitzner F.P., Wibral M., Neto J.P., Wilczek M., Priesemann V. (2020). Inferring change points in the spread of COVID-19 reveals the effectiveness of interventions. Science.

[120] Friesen M. (2003). Statistical surveillance. Optimality and methods. Int. Stat. Review.

[121] Friesen M., Andersson E., Schiöler L. (2009). Robust outbreak surveillance of epidemics in Sweden. Stat. Med..

[122] Brauner J.M., Mindermann S., Sharma M., Stephenson A.B., Gavenciak T., Johnston D., Salvatier J., Leech G., Besiroglu T., Altman G. (2020). The effectiveness and perceived burden of nonpharmaceutical interventions against COVID-19 transmission: A modelling study with 41 countries. medRxiv Prepr..

[123] Österreicher F., Vajda I. (1993). Statistical information and discrimination. IEEE Trans. Inform. Theory.

[124] De Groot M.H. (1962). Uncertainty, information and sequential experiments. Ann. Math. Statist..

[125] Krafft O., Plachky D. (1970). Bounds for the power of likelihood ratio tests and their asymptotic properties. Ann. Math. Stat..

[126] Basawa I.V., Scott D.J. (1976). Efficient tests for branching processes. Biometrika.

[127] Feigin P.D. (1978). The efficiency criteria problem for stochastic processes. Stoch. Proc. Appl..

[128] Sweeting T.J. (1978). On efficient tests for branching processes. Biometrika.

[129] Linkov Y.N. (2005). Lectures in Mathematical Statistics, Parts 1 and 2.

[130] Feller W., Neyman J. (1951). Diffusion processes in genetics. Proceedings of the Second Berkeley Symposium on Mathematical Statistics and Probability.

[131] Jirina M. (1969). On Feller’s branching diffusion process. Časopis Pěst. Mat..

[132] Lamperti J., Le Cam L.M., Neyman J. (1967). Limiting distributions for branching processes. Proceedings of the Fifth Berkeley Symposium on Mathematical Statistics and Probability, Vol. II, Part 2.

[133] Lamperti J. (1967). The limit of a sequence of branching processes. Z. Wahrscheinlichkeitstheorie Verw. Geb..

[134] Lindvall T. (1972). Convergence of critical Galton-Watson branching processes. J. Appl. Prob..

[135] Lindvall T. (1974). Limit theorems for some functionals of certain Galton-Watson branching processes. Adv. Appl. Prob..

[136] Grimvall A. (1974). On the convergence of sequences of branching processes. Ann. Probab..

[137] Borovkov K.A. (1986). On the convergence of branching processes to a diffusion process. Theor. Probab. Appl..

[138] Ethier S.N., Kurtz T.G. (1986). Markov Processes: Characterization and Convergence.

[139] Durrett R. (1996). Stochastic Calculus.

[140] Kawazu K., Watanabe S. (1971). Branching processes with immigration and related limit theorems. Theor. Probab. Appl..

[141] Wei C.Z., Winnicki J. (1989). Some asymptotic results for the branching process with immigration. Stoch. Process. Appl..

[142] Sriram T.N. (1994). Invalidity of bootstrap for critical branching processes with immigration. Ann. Stat..

[143] Li Z. (2006). Branching processes with immigration and related topics. Front. Math. China.

[144] Dawson D.A., Li Z. (2006). Skew convolution semigroups and affine Markov processes. Ann. Probab..

[145] Cox J.C., Ingersoll J.E., Ross S.A. (1985). A theory of the term structure of interest rates. Econometrica.

[146] Cox J.C., Ross S.A. (1976). The valuation of options for alternative processes. J. Finan. Econ..

[147] Heston S.L. (1993). A closed-form solution for options with stochastic volatilities with applications to bond and currency options. Rev. Finan. Stud..

[148] Lansky P., Lanska V. (1987). Diffusion approximation of the neuronal model with synaptic reversal potentials. Biol. Cybern..

[149] Giorno V., Lansky P., Nobile A.G., Ricciardi L.M. (1988). Diffusion approximation and first-passage-time problem for a model neuron. Biol. Cybern..

[150] Lanska V., Lansky P., Smith C.E. (1994). Synaptic transmission in a diffusion model for neuron activity. J. Theor. Biol..

[151] Lansky P., Sacerdote L., Tomassetti F. (1995). On the comparison of Feller and Ornstein-Uhlenbeck models for neural activity. Biol. Cybern..

[152] Ditlevsen S., Lansky P. (2006). Estimation of the input parameters in the Feller neuronal model. Phys. Rev. E.

[153] Höpfner R. (2007). On a set of data for the membrane potential in a neuron. Math. Biosci..

[154] Lansky P., Ditlevsen S. (2008). A review of the methods for signal estimation in stochastic diffusion leaky integrate-and-fire neuronal models. Biol. Cybern..

[155] Pedersen A.R. (2000). Estimating the nitrous oxide emission rate from the soil surface by means of a diffusion model. Scand. J. Stat. Theory Appl..

[156] Aalen O.O., Gjessing H.K. (2004). Survival models based on the Ornstein-Uhlenbeck process. Lifetime Data Anal..

[157] Kammerer N.B. (2011). Generalized-Relative-Entropy Type Distances Between Some Branching Processes and Their Diffusion Limits. Ph.D. Thesis.

